# River channel conveyance capacity adjusts to modes of climate variability

**DOI:** 10.1038/s41598-019-48782-1

**Published:** 2019-09-02

**Authors:** L. J. Slater, A. Khouakhi, R. L. Wilby

**Affiliations:** 1 School of Geography and the Environment, University of Oxford, Oxford, UK; 2 School of Architecture, Building and Civil Engineering, Loughborough University, Loughborough, UK; 3 Geography and Environment, Loughborough University, Loughborough, UK

**Keywords:** Hydrology, Hydrology, Geomorphology

## Abstract

River networks are typically treated as conduits of fixed discharge conveyance capacity in flood models and engineering design, despite knowledge that alluvial channel networks adjust their geometry, conveyance, planform, extent and drainage density over time in response to shifts in the magnitude and frequency of streamflows and sediment supply. Consistent relationships between modes of climate variability conducive to wetter-/drier-than-average conditions and changes in channel conveyance have never been established, hindering geomorphological prediction over interannual to multidecadal timescales. This paper explores the relationship between river channel conveyance/geometry and three modes of climate variability (the El Niño–Southern Oscillation, Atlantic Multidecadal Oscillation, and Arctic Oscillation) using two-, five- and ten-year medians of channel measurements, streamflow, precipitation and climate indices over seven decades in 67 United States rivers. We find that in two thirds of these rivers, channel capacity undergoes coherent phases of expansion/contraction in response to shifts in catchment precipitation and streamflow, driven by climate modes with different periodicities. Understanding the sensitivity of channel conveyance to climate modes would enable better river management, engineering design, and flood predictability over interannual to multidecadal timescales.

## Introduction

**Figure 1:**
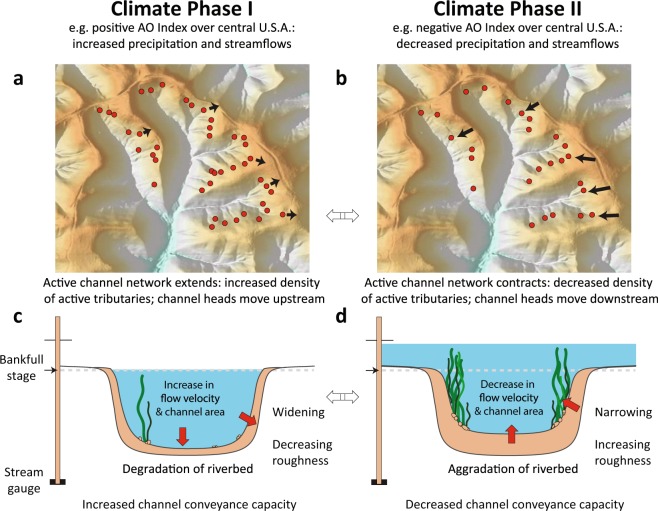
Schematic illustrating the hypothesised response of an idealized catchment and river channel cross-section during a wet (left column) and a dry (right column) phase of a mode of climate variability (over multiple years). (**a**) Extension and (**b**) contraction of the active fluvial network is symbolized by channel heads (red circles: only a selection is represented). During wet periods (left column), the length of the channel network may extend^8,10^ as channel heads^68^ migrate upstream and the active drainage network becomes more dense in response to heightened runoff; the reverse is true during dry periods. Below, a conceptual illustration of single-thread river channel cross-section indicates (**c**) increased conveyance during wet years and (**d**) decreased conveyance during dry years. Under constant sediment supply, enhanced runoff may result in more geomorphically effective (sediment-displacing) flow, thereby increasing channel cross-sectional conveyance capacity due to increased flow velocities and erosion of the river channel boundaries.

Alluvial river channels are self-formed by the sediment-laden flow they convey downstream, adjusting their geometry^1–4^, conveyance^5,6^, planform^7^, network extent^8^ and drainage density^9,10^ dynamically over time to reflect prevailing streamflow regimes^11,12^ and any changes in the sediment supply generated upstream^13^. Morphological responses may include subtle shifts in cross-sectional stream channel geometry^6,14^ or widespread landscape transitions^15^, involving progressive or abrupt change^16^ over daily to millennial timescales. Thus, the longitudinal expansion and contraction of active stream networks^8–10,17^ as well as shifts in channel conveyance (i.e. the ability of the channel to convey flow, measured in m^3^/s) and cross-sectional geometry^11,18^ are expected to reflect the occurrence of wetter- or drier-than-average climatic conditions that alter the volumes of precipitation, runoff, streamflow, and sediment supplied to alluvial channels^8^ (Fig. 1).

Identifying the modes of climate variability that affect regional precipitation patterns and streamflow^19–26^ is the first step in understanding the potential for associated channel adjustments. In the USA, links between weather and large-scale climate variability are relatively well established but vary regionally^22–26^. Patterns with interannual to interdecadal periodicity, such as the El Niño-Southern Oscillation (ENSO), the Pacific-North America (PNA) pattern^20,21^, or Pacific Decadal Oscillation (PDO)^27^ exert a strong influence on rainfall, streamflow, and sediment supply^27^. Other prominent modes that display no particular periodicity, such as the Arctic Oscillation (AO, which exhibits similarity with the North Atlantic Oscillation Index) also exert strong regional influences on temperature, precipitation extremes and streamflow^23,28^. Over multidecadal timescales across the USA, findings differ: some studies have found that climate anomalies, river flows^19^ and flood events^29^ are linked to slow variations of sea surface temperature driven by the Atlantic Multidecadal Oscillation (AMO); others indicate that the fraction of stream gages exhibiting a significant relation between flood magnitude/frequency and the AMO is no greater than would be expected by chance^30^. Uncovering relationships between local climate and large-scale climate modes is challenging, because associations are notoriously non-stationary^31^, reflect interactions between multiple modes^32^ and are influenced by emerging anthropogenic climate change signals^33^.

Although it is recognized that climate patterns have consistent effects on streamflow distributions in different regions of the world^25,26,34,35^, that alluvial river channels adjust to reflect changing streamflow and sediment regimes^1,4,11,36^, and that channel morphology can be influenced by large-scale climate modes^27,37^, a systematic association between large-scale climate patterns and changes in channel conveyance has not been established in observational records. The influence of climate variability on the hydrological expansion and contraction of active river networks (Fig. 1a,b) has been assessed using field campaigns^9,10,38^ over timescales of individual rainstorms and seasons. However, elucidating the extent to which climate patterns may drive the conveyance of river channels at the scale of river cross-sections (Fig. 1c,d) requires dense, repeat transect measurements in fixed locations over interannual to multidecadal timescales.

To investigate channel geometry adjustment to patterns of climate variability we use hydrometric measurements gathered by the U.S. Geological Survey (USGS) between 1950 and 2017^39^. Channel transect measurements are made at regular (monthly-seasonal) intervals to update streamflow rating curves, but can also be used to monitor changes in channel form^3,6,14,27,40–43^. One important caveat with channel transect measurements is that they are purposely located in stable sites along the river, thus detectable responses in channel form to climate modes are likely to be conservative. Here, we identified a subset of 67 gaging sites with exceptional temporal density of transects and consistent measurement location over time (predominantly in the eastern USA, where most of the long-term records are located; Supplementary Figs S5–S205). Time series of cross-sectional channel conveyance capacity (m^3^/s), cross-sectional flow area (m^2^), mean cross-sectional flow velocity (m/s), channel width (m), and riverbed elevation (m) were computed using residuals from rating relationships^6,44^. We excluded sites with abrupt artificial shifts in flow over the period of record. Additionally, because channels may exhibit gradual positive/negative trends in conveyance capacity over time^6^ (e.g. due to changes in sediment delivery from climatic and anthropogenic changes upstream, such as glacial melt, logging, or urbanisation), the data were detrended to remove such signals (and thus avoid spurious correlations with the climate indices) by computing standardised residuals of a linear regression model (see Supplementary Fig. S1). In practice, this means that some of the river channels may have become considerably wider/narrower, deeper/shallower over the seven decades, but we focus solely on the annual to multi-decadal fluctuations to assess whether they reflect climate variability. Thus, we question whether the prevailing assumption of stationarity (or quasi-equilibrium^13^) in channel form (i.e. the notion that channels reflect stationary, time-averaged distributions of streamflow) is reasonable, and appropriate for broader applications such as global flood modelling (e.g.^45^) and engineering design.

We selected the Oceanic Niño Index (ONI, reflective of ENSO), AO index (AOI), and AMO index (AMOI) because of their known influence on USA precipitation patterns, as well as their different periodicities which could manifest in the data – ranging from interannual (ONI) to multidecadal (AMOI), or no particular (AOI) periodicity. Climate index time series were obtained from the National Oceanic and Atmospheric Administration (NOAA)^46^, gridded precipitation from PRISM^47,48^, and mean daily streamflow from the USGS National Water Information System (NWIS)^49^. All data were aggregated (medians of channel form, precipitation, streamflow, and climate indices) over common non-overlapping two-, five- and ten-year blocks starting in 1950 (thus testing the sensitivity of channel response over different block lengths, acknowledging that temporal aggregation could dampen the climate signals). These temporal aggregates provide more robust estimates of average channel form than individual field measurements by reducing the influence of noise/gaps in the data and accounting for lag times (e.g.,^50^) in the response of channels to changing streamflow and sediment regimes. The use of identical aggregation periods for all variables provides a common baseline to assess whether climate variability (i.e. the occurrence of *wetter- or drier-than-average phases*) is reflected in average conditions of precipitation, streamflow, and thereafter, channel morphology. Standardised anomalies of precipitation and streamflow aggregates were computed at each site by subtracting the long-term mean and dividing by the standard deviation, for comparability with the standardised channel geometry and climate indices (Supplementary Figs S5–S205).

## Results

**Figure 2:**
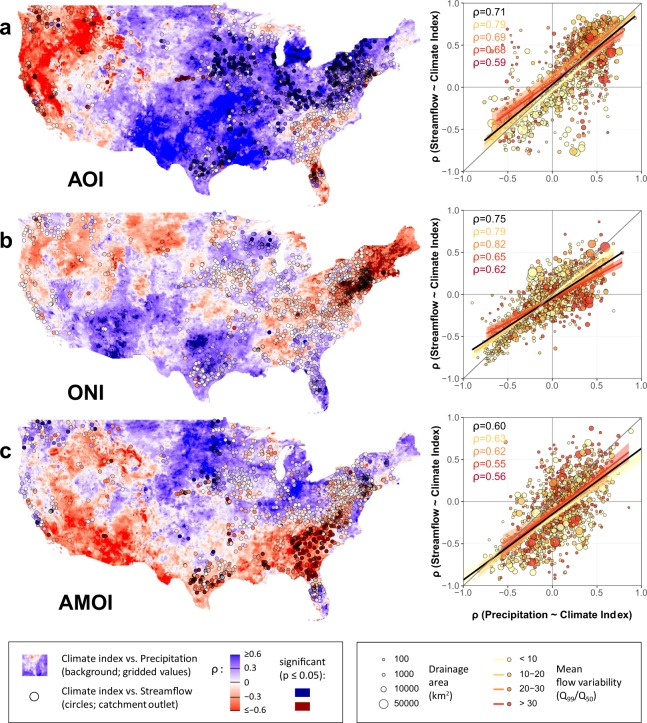
Strength of the relationship between three climate indices and precipitation/streamflow at 1263 stream gages, 1950–2017, using 5-year median values. Rows indicate three indices: (**a**) AOI, (**b**) ONI, (**c**) AMOI. Left column: Strength of correlation (Spearman’s ρ) between the climate indices and precipitation (background) or between the same climate indices and streamflow (circles). Red shades indicate negative relationship, blue positive relationship, white insignificant relationship. Right column: Correlations at every site (using the catchment-averaged precipitation and the streamflow at the gage). The strength of the association (ρ and linear regression with confidence intervals) is shown for all data (black), and for four groups reflecting streamflow variability (yellow to red, where red has the highest flow variability). Flow variability (or flashiness) is measured by the ratio of the 99^th^ percentile of streamflow (high flow, Q_99_), to the 50^th^ percentile of streamflow (median flow, Q_50_), over the entire period of record. Basin drainage area is indicated by circle size.

We first assess the strength of the relationship between the three climate indices and catchment-averaged precipitation at 1263 gaging sites over seven decades. Marked spatial variations can be seen across the USA (Fig. 2): precipitation correlates positively with AOI in the central and south-western USA (Spearman’s ρ > 0.6 in parts of the Midwest and Southwest), but negatively in the western USA (ρ < −0.3 along parts of the West Coast). In the north-eastern USA, precipitation correlates positively with AOI but negatively with ONI. The AMOI correlates positively with rainfall over most of the low-lying parts of the northern USA and negatively over much of the southern USA (ρ often < −0.3 from southern Texas to South Carolina but excluding Florida). These continental relationships between climate indices and precipitation/streamflow echo well-known spatial patterns (such as the prevalence of warmer and wetter conditions during a positive AOI in the central USA) but may depart from previous work due to different study periods^23,25^ and streamflow thresholds^26^. Additionally, the temporal aggregates (five-year periods in Fig. 2) typically include a mix of both positive and negative phases of the different climate modes and, therefore, are only indicative of *average* conditions.

Broadly, we find the influence of climate variability on precipitation patterns is reflected by streamflow. Differences among basins in the response of streamflow to climate variability are likely a function of catchment characteristics such as basin area, evapotranspiration, land cover^51^/vegetation, anthropogenic stresses^52^ such as flow abstraction/augmentation and groundwater exploitation, fraction of flow from snow/ice melt, and geological conditions (Fig. 2). Catchments with more subdued flow regimes may reflect climate variability and changes in wetness better than smaller, flashier catchments^53^ over these timescales (Fig. 2). However, the extent of scatter in the underlying data suggests that across the USA other drivers, such as human impacts, modulate fluvial sensitivity to climate.

Next we consider the extent to which aspects of river channel *form* reflect these climate-driven variations in precipitation and streamflow over inter- to multi-decadal time periods. At sites where the precipitation and streamflow rates are positively (negatively) correlated with a particular mode of climate variability, we expect that a positive phase of the climate mode may result in a larger (smaller) channel conveyance capacity. We hypothesize, following equilibrium theory^12,13,54^, that channel conveyance capacity may increase – on average – in wetter years (although channels may also aggrade during individual floods, but there are insufficient data to evaluate the role of sediment here). Variability in channel response (increased/decreased conveyance) is expected to arise from natural variability in river systems, anthropogenic impacts upstream, and/or uncertainties/error in the underlying channel geometry measurements.

**Figure 3:**
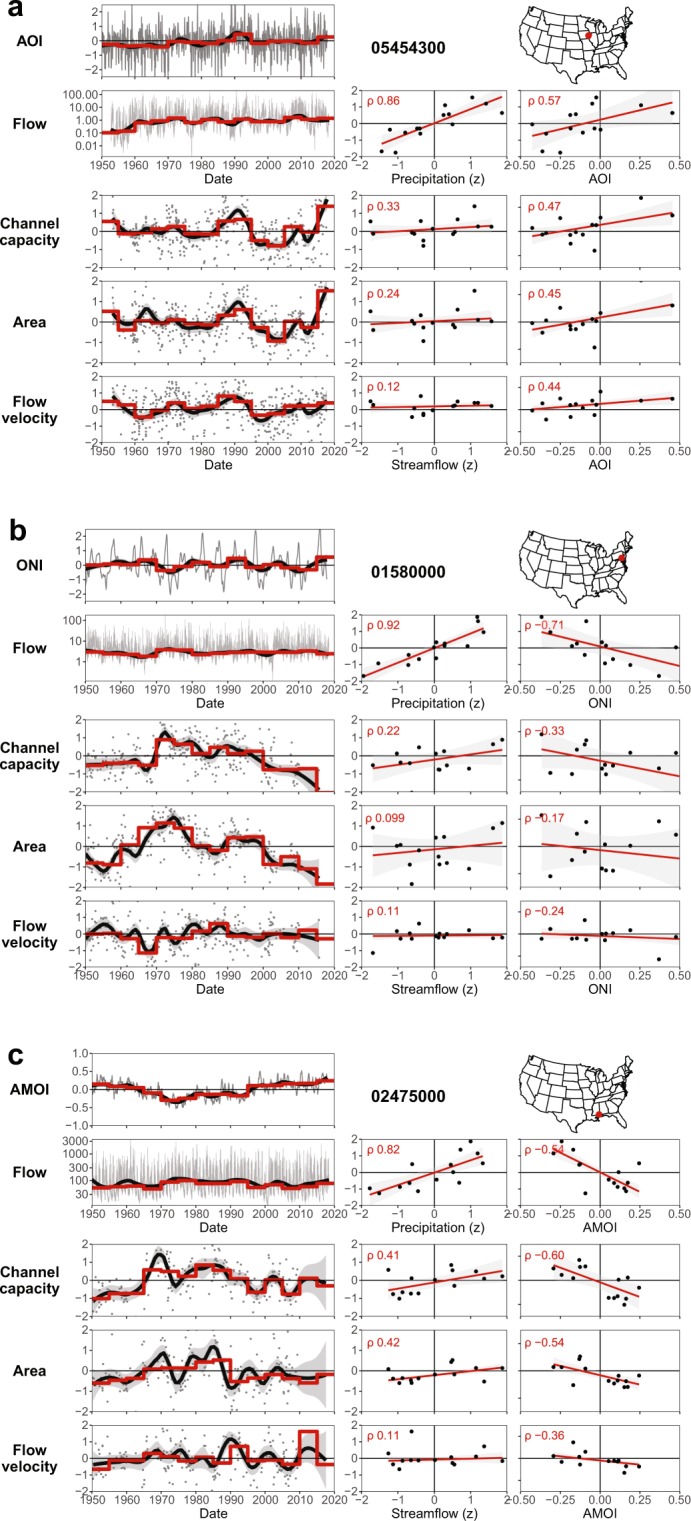
Temporal variations in climate indices, precipitation, streamflow, and river channel conveyance/geometry at three illustrative sites: (**a**) site 05454300: Clear Creek near Coralville, Iowa (AOI), (**b**) site 01580000: Deer creek at Rocks, Maryland (ONI); (**c**) site 02475000: Leaf River near McLain, Mississippi (AMOI). All graph units are standardised anomalies (*z*), except for the streamflow time series (m^3^/s) and Date. Within each panel, the time series indicate, from top to bottom: the climate index (*z*), streamflow (m^3^/s), the cross-sectional channel conveyance capacity (‘Channel capacity’, *z*), the cross-sectional flow area (‘Area’, *z*), and the cross-sectional average flow velocity (‘Flow velocity’, *z*). Grey circles indicate field measurements (*z*) at different points in time (see Methods). Five-year median values (red lines) reflect average channel form, reducing the effects of noise and accounting for lag times between the climate index and the response of the river system. Scatterplots indicate the relationship between: (row 2) precipitation/flow and climate index/flow; (row 3): streamflow/channel capacity and climate index/channel capacity; (row 4); streamflow/area and climate index/area; (row 5); streamflow/flow velocity and climate index/flow velocity. Red lines indicate the corresponding linear regressions (standard error is in grey), with the Spearman’s rank correlation coefficient (ρ) in red for the five-year median values. Equivalent graphs (including five- and ten-year aggregates) are provided for all sites in the Supplementary information, including graphs of channel width and riverbed elevation.

We provide a detailed illustration of the relationship between the climate indices, precipitation, streamflow, and channel capacity/geometry at three sites, chosen for their data quality and contrasting response to different modes (Fig. 3). At Clear Creek (Fig. 3a) over seven decades, precipitation and streamflow records are positively correlated (ρ = 0.86), as are the AOI and streamflow (ρ = 0.57). The normalized river channel conveyance capacity exhibits phases of expansion and contraction after the removal of any long-term trend. Despite considerable variability in the underlying stream measurements, the AOI and channel conveyance capacity (as well as area and mean flow velocity) are positively correlated (ρ = 0.47). At Deer Creek and Leaf River (Fig. 3b,c), in contrast, we find a negative relationship between the ONI/AMOI and streamflow (ρ = −0.71/ρ = −0.54 respectively), which is also reflected in the channel capacity adjustments (ρ = −0.33/ρ = −0.60, respectively). The association between the climate index and channel geometry can sometimes be stronger than the relationship between the streamflow and channel geometry (Fig. 3; Supplementary Figs S5–S205), suggesting that discharge only partly explains changes in channel form. In other words, the climate modes may also be a proxy for other direct/indirect determinants of channel response, such as sediment delivery^55^ or variability in riparian/catchment vegetation^5,56^. To obtain a broader picture of channel response – acknowledging that the quality and reliability of measurements and computed relationships may vary across sites – we pool the data across all 67 rivers (their location is shown in Supplementary Figs S5–S205).

**Figure 4:**
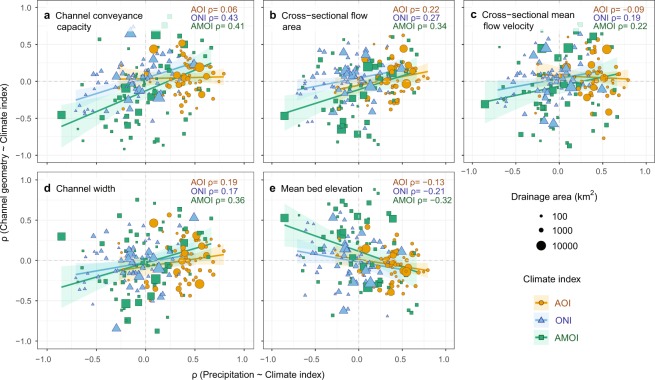
Relationships between climate indices and precipitation reflected in the channel geometry at each of 67 sites. Five-year median values of all variables (as in Figs 2 and 3) are used to compute the Spearman’s rank correlation coefficient (ρ) (i) between each climate index and precipitation (x-axes) and (ii) between the climate index and channel geometry (y-axes; from (**a**–**e**): channel conveyance capacity, cross-sectional flow area, cross-sectional mean flow velocity, channel width, and mean riverbed elevation). Note: Supplementary Figs S2 and S3 show the same plot with *two- and ten-year* aggregation windows. Climate indices are indicated as shapes and colours: orange circles (AOI), blue triangles (ONI), green squares (AMOI), with size of shapes indicating the drainage area. Linear regression is shown for each index with confidence intervals in transparent colours. Note: Supplementary Fig. S4 shows the same plot but with the correlation between *streamflow* and the climate index on the x-axis.

We assess the imprint of climate variability on alluvial channel morphology at each site by considering whether the correlation (ρ) between the climate index and precipitation displays the same sign as the correlation between the same climate index and the channel geometry. Despite considerable variability and uncertainty/noise in the underlying measurements, broadly, a positive (negative) correlation between the indices and precipitation emerges between the same indices and channel conveyance capacity at 63% of sites (based on 201 combinations of 67 sites and 3 climate indices; Fig. 4a). Split by mode, this represents 67% of sites for AMO, 57% for AO, and 66% for ONI. For comparison, repeating the same analysis with the non-detrended data indicates that the relationship holds in 70% of sites (67% AMO, 78% AO and 66% ONI). Hence, detrending reduces the number of sites with a detectable AO imprint (as expected, because AO displays a positive monotonic trend, Fig. 3). Thus, overall, the imprint of the modes is detectable with varying levels of strength in about two-thirds of the sites.

An association between channel form and the climate indices is also evident for the cross-sectional flow area, cross-sectional mean flow velocity, channel width, and mean bed elevation (Fig. 4b–e), although at any given site, the flow velocity, width, and depth do not necessarily exhibit the same direction of change, due to differences in channel adjustment. All three modes appear to associate with channel form, suggesting that the importance of large-scale climate patterns in driving channel form depends less on their periodicity than on the strength of their influence on regional rainfall/streamflow regimes (Fig. 4). The clustering of orange circles in Fig. 4 indicates that most sampled sites are in regions where there is a positive correlation between the AOI and precipitation (see Supplementary Figs S5–S205). Further research is needed to determine which catchment characteristics modulate the strength of this imprint; preliminary statistical tests indicate no significant differences in the slopes of the relationships shown in Fig. 4 when data are stratified by basin area or mean annual precipitation.

Overall, the response of river channels to large-scale modes of climate variability suggests that river networks may be viewed as dynamic, breathing systems that expand and contract over interannual to multidecadal timescales in synchrony with regional climate. Detecting robust morphological signals within the noise of transect data is technically demanding. However, this preliminary exploration provides evidence that channel form may well respond to modes of climate variability: wetter- or drier-than-average conditions are often associated with periodic increases/decreases in channel geometry and conveyance. The existence of links between climate indices and channel hydro-morphology (but also with river network properties such as active channel length, density, or fractal dimension) could be further evaluated using long-term measurements from remotely sensed data (e.g. satellite imagery). However, we do not expect channel conveyance to systematically increase everywhere in response to increased flow and caution that fluvial adjustments reflect the complex interplay of nonstationary anthropogenic and climatic influences within each basin, including feedback mechanisms^57^. This variable behaviour typically precludes straightforward generalizations about channel response without assessing long term channel trajectories, local sediment yield conditions^58^, and landcover history on a site-by-site basis.

Our findings have implications for flood modelling, estimates of flood recurrence, and the design of riverine infrastructure in a changing climate. In flood models, channel geometry and conveyance are typically considered stationary and are recognised as one of the greatest sources of model uncertainty^45^. Since channel conveyance capacity directly influences bankfull flood recurrence intervals^6^, this work suggests that the assumption of channel stationarity may lead to systematic over- or under-prediction of the frequency of out-of-bank flow (i.e. flood return periods, duration, and inundation extent). Additionally, in engineering, if flood designs are based on data gathered during periods where climate modes favoured decreased channel conveyance there is a danger that surveyed channel dimensions and flood conveyance are underestimated over the longer-term. The existence of potential periodicities in river channel conveyance implies that flood models, infrastructure, and flood recurrence estimates should be designed to incorporate the temporal variability in channel dimensions, particularly in regions with rapidly changing hydroclimatic regimes and river morphodynamics. In due course, it may even be possible to assign quality markers to individual streamflow gauging stations according to the sensitivity of their cross-sections to climate variability.

## Detailed Methods

### Channel measurements

This work is based on an analysis of 9,736 individual river cross-sectional channel surveys (transects) taken over a period of almost 70 years at 67 alluvial USGS stream gages. The methods used for preparing and filtering USGS stream field measurements are similar to^6,59^ but are detailed below for clarity. USGS field stream measurements between 1950-01-01 and 2017-12-31 were downloaded from the USGS NWIS^39^. These are transect measurements of channel streamflow (*Q*, m^3^/s), cross-sectional flow area (*A*, m^2^), channel width (*W*, m), and average cross-sectional flow velocity (*V*, m/s), for different values of stage, i.e. gage height (*G*). In addition, we also consider the relative mean river bed elevation (*B*), which is computed as: *B* = *G* − (*A*/*W*). We converted all channel measurements to metric units and then removed any measurements made in icy conditions (i.e. where the control_type_cd contained any of the following strings: “ICE”, “CICE”, “SICE”, “AICE”, “IceAnchor”, “IceShore”, or “IceCover”). We removed any transect measurements with negative values of *Q*, *W*, or *A*, as well as any measurements that were infinite or incomplete (i.e. there had to be a measurement of *Q*, *A*, *W*, and *V* for the entire transect to be retained in the dataset).

To detect potential measurement inaccuracies, we computed a dummy variable *Q*/(*AV*) then removed any streamflow measurements where this value was greater than 1.1 or less than 0.9 (i.e. retaining only sites with measurement errors less than 10%). For each site we kept the single channel number (variable “chan_nu”) with the greatest number of measurements, to increase confidence in measurement location. To limit the influence of gaps in data, we calculated data completeness (defined here as the ratio of years with data to the total time span of the data, in years) over the available period for each site. We retained only active sites (where transects were still made after 2016) with at least 80% completeness, and at least 45 years of data. After all these filtering steps and in addition to those described below, the remaining 67 sites had a mean of 312 field channel measurements each (between 109 and 603) with a mean of 94% completeness over 59 years (min = 47, max = 68 years) over the entire period (1950 to 2017).

### Estimating the channel conveyance capacity and geometry

We quantify how channel conveyance and geometry vary over time using the residuals of the relationships between log-transformed estimates of channel dimensions and gage height. The approach is similar to^44,60^ and simplified from our earlier work^6,59^ because here we only require standardised residuals and not dimensional estimates of channel form. The method removes the influence of gage height on the relationship between the channel dimensions and time^60^. Values of *Q*, *A*, *V, W*, and *B* are transformed using the natural logarithm. We fit a Loess (locally weighted scatterplot smoothing) curve with a span of alpha = 0.25 (found to be appropriate for all sites by examining all plots of the estimated channel measurements) to the relationships between *G*-*lnQ*, *G*-*lnA, G*-*lnV, G*-*lnW, and G*-*lnB* (all Loess fits are shown in Supplementary Figs S5–S205). The Loess fit represents time-averaged values of *lnQ*, *lnA, lnV, lnW, and lnB* for every value of *G*. We compute the regression residuals (i.e. the observed minus the predicted values of the relationship) and transform the residuals back to dimensional units using the antilog (exp). These residuals provide an indication of variations in channels dimensions over time. However, to compare changes in channel form with precipitation, streamflow, and the channel indices, we must still detrend the data as described next.

### Anthropogenic influences and variable detrending

Very few USGS stream gages can be defined as ‘pristine’ (i.e. have not experienced any human impacts on their sediment and/or streamflow regimes). Thus, restricting the data to pristine sites would have limited the analysis considerably given the strictness of our filtering criteria. Further, this analysis seeks to assess whether climate signals can be detected broadly across many USA sites – not just in pristine, unpopulated catchments (which are of lesser relevance to risk management). We remove any systematic long-term trajectories in the channel data (e.g., due to progressive shifts in sediment delivery from upstream) to minimize any potentially spurious correlations with the climate indices. For instance, long-term trends in channel geometry due to anthropogenic influences might coincide with an upward trend in the AOI. Channel measurements are thus detrended by extracting the standardised residuals from the linear regression between the dimensional residuals and the date, using the MASS^61^ package in R. The effects of detrending are shown in Supplementary Fig. S1: after detrending, the relationship between channel geometry and streamflow/AMOI is still present, but not as strong as before.

### Verification of measurement location

Channel measurements were filtered to retain only the most reliable measurements made consistently at the same gaging location over time, by evaluating the scatterplots of *G*-*lnQ*, *G*-*lnA, G*-*lnV, G*-*lnW, and G*-*lnB* (all exclusions are indicated as grey circles in Supplementary Figs S5–S205). Typically, this meant removing measurements made at very high or very low stage, if these measurements were made in different locations (indicated by, for example, an abrupt shift in channel width or flow area).

### Streamflow and climate data

Mean daily streamflow data were downloaded for all sites from the USGS NWIS^49^ using the dataRetrieval^62^ package in R. Monthly time series of the climate indices were downloaded from the NOAA Climate Prediction Center for the same period, 1950-01-01 to 2017-12-31^46^. The monthly time series originate from data that were computed as follows: AMOI^32^ timeseries were calculated from the Kaplan SST dataset which is updated monthly, and series reflect North Atlantic temperatures. The daily AO index (AOI^63^) was constructed by projecting the daily (00Z) 1000 mb height anomalies poleward of 20°N onto the loading pattern of the AO. ONI^64^ was computed as 3 month running mean of ERSST.v5 SST anomalies in the Niño 3.4 region (5°N-5°S, 120–170°W), based on centred 30-year base periods updated every 5 years.

Precipitation data were obtained from the PRISM climate group^47,48^ using the prism^65^ package in R. Catchment-averaged values of monthly precipitation were computed using basin boundaries from USGS NHDPlus shapefiles (version 1).

### Choice of sites (channel gaging locations)

We investigate 67 sites that have a high number and regular temporal density of transect measurements made consistently over five to seven decades at the same location. We sought to retain as many sites as possible. However, most gaging sites have either a sparse/irregular temporal density of measurements or inconsistent/unreliable transect location. Every page in Supplementary Figs S5–S205 indicates one site and one climate index. We used metadata from the USGS Annual Water Data reports to obtain information about any changes in gage location at all the sites. Sites with a change in gage location since 1950 were removed. Any minor changes in gage datum (i.e. at the same location) were corrected following ref.^66^. Any sites where there were doubts about the accuracy of channel measurements (e.g. due to potential changes in measurement location or gage datum) were discarded. To further ascertain measurement location, we checked that channel measurements (of *Q, A*, *W*, *V*, *B*) were made within a similar range over the entire measurement period (1950–2017). Any unreliable sites were systematically discarded, as we prioritized having fewer sites with highest quality measurements over a greater number of sites with higher uncertainty.

### Variable standardisation, aggregation, and correlation

We computed standardised anomalies of the streamflow and precipitation aggregates (by subtracting the sample mean and dividing by the sample standard deviation)^67^, to facilitate comparisons with the climate indices and among sites with different physical and climate characteristics. The mean values of Q_50_ and of Q_99_/Q_50_ were computed using the 50^th^ and 99^th^ percentiles of the daily streamflow distribution using all available data (where Q_99_ indicates the flow that is exceeded 1% of the time). Time series of estimated cross-sectional channel geometry, climate indices, streamflow, and precipitation data were all aggregated using the median value over two-, five- and ten-year periods starting on 1950-01-01. Spearman’s rank correlation coefficients (ρ) were computed between variables (e.g. precipitation and channel geometry) using two-, five- and ten-year median values calculated over identical timeframes. Across all sites (201 combinations), the mean sample size on which these correlations are based is 31 values (two-year aggregates), 13.1 (five-year) 6.7 (ten-year).

## Supplementary information

Supplemental Information

## Data Availability

Climate indices are available from NOAA at https://www.esrl.noaa.gov/psd/data/climateindices/list/. USGS stream measurements are available at https://waterdata.usgs.gov/nwis/measurements and streamflow measurements from http://waterdata.usgs.gov/nwis/dv/?referred_module=sw. Precipitation data are available from PRISM climate group at http://www.prism.oregonstate.edu/. Summary statistics and graphs for all sites are provided in the Supplementary information. All codes were written in the open-source programming language R.
