# Supplementary material for: River channel conveyance capacity adjusts to modes of climate variability
Source: Sci Rep. 2019 Sep 2;9:12619. doi: 10.1038/s41598-019-48782-1 (PMC6718627; doi:10.1038/s41598-019-48782-1)
Supplement: Supplementary file 1 — Supplemental Information [file 41598_2019_48782_MOESM1_ESM.pdf]

Supplementary information

River channel conveyance capacity adjusts to modes of climate variability

L. J. Slater, A. Khouakhi, R. L. Wilby

This file contains:

**Supplementary Fig. S1**<br>(1 page)	Relationships between the AMOI, precipitation, streamflow, and channel geometry at site 02192000, shown before and after detrending the channel transect data.
**Supplementary Figs. S2-S4**<br>(3 pages)	Figure 4 from the main text is reproduced:<br>- using 2-year aggregate time periods (instead of 5-year time periods)<br>- using 10-year aggregate time periods (instead of 5-year time periods)<br>- using Spearman correlations between the climate indices and streamflow (instead of precipitation)
**Supplementary Figs. S5-S205**<br>(201 pages)	Each page indicates one site and one climate index (there are 67 sites x 3 indices).<br><br>For every page, graphs indicate (columns numbered from left to right):<br><br>**Column 1:**<br>Top row: Thin grey line indicates time series of the relevant climate index (in monthly anomalies). Thick dark grey line indicates LOESS (locally weighted scatterplot smoothing) fit, with 95% confidence intervals in lighter grey shade. Thick red stepped line indicates 5-year median values.<br>Channel measurement scatter plots (5 rows): Graphs indicate raw (grey) channel measurements that were discarded because the measurement location was considered unreliable. Filtered (retained) channel measurements are indicated in red (see **Methods** for a description of filtering procedure). From top to bottom, the graphs indicate stage (x-axis, meters) vs channel conveyance capacity (Q, m^3/s), channel flow area (A, m^2), mean cross-sectional flow velocity (V, m/s), width (W, m), and bed elevation (BE, m) (on y-axes).<br><br>**Column 2:**<br>Top row: Map of site location (USGS stream gage indicated as red circle on the map). USGS site number is in bold font beneath the map of the USA.<br>Channel measurement scatter plots (5 rows): Same as in column 1, except the channel measurements have been log-transformed and a LOESS curve is fit (alpha = 0.2 for all sites) to compute residuals for further analysis (see **Methods** for description of residual analysis).<br><br>**Column 3:**<br>Top row: Thin grey line indicates time series (Date, x-axis) of daily streamflow (y-axis, shown in m^3/s). Thick dark grey line indicates LOESS

	(Locally weighted scatterplot smoothing) fit, with 95% confidence intervals. Thick stepped red line indicates 5-year median values.<br>Channel measurement time series: Time series (Date, x-axis) of channel geometry (y-axes: channel conveyance capacity, channel flow area, mean flow velocity, width, and bed elevation) as detrended standardised anomalies (z). Dark grey line indicates LOESS (locally weighted scatterplot smoothing) fit with 95% confidence intervals. Thick red line indicates 5-year median values.<br><br>**Column 4:**<br>Top row: Scatter plot of precipitation (x-axis, in standardised anomalies, z) vs streamflow (y-axis, z). Red circles indicate 5-year median values of both variables; blue circles indicate 10-year median values for comparison. Red line indicates linear regression for 5-year median values with 95% confidence intervals. Blue line indicates linear regression for 10-year median values with 95% confidence intervals. Spearman's correlation coefficient (ρ) is shown on the scatterplot for both the 5- (red, top) and 10- (blue, bottom) year median values.<br>Channel measurement scatterplots: Scatterplots indicating the relationships between the five- (red) and ten- (blue) year median streamflow median (x-axis) and channel geometry median values (y-axes). Red line indicates linear regression for 5-year median values with 95% confidence intervals. Blue line indicates linear regression for 10-year median values with 95% confidence intervals. Correlation coefficient (ρ) is shown for both the 5- (red) and 10- (blue) year median values on the scatterplot.<br><br>**Column 5:**<br>Top row: Scatterplot indicating the relationship between the five- (red) and ten- (blue) year median climate index (x-axis, z) and streamflow median value (y-axis, z). Symbols/colours are as described for column 4.<br>Channel measurement scatterplots: Scatterplots indicating the relationships between the five- (red) and ten-(blue) year median values of channel geometry (y-axes) vs the relevant climate index (x-axis). Symbols/colours/regression are as described above for column 4.
**Supplementary Table T1**<br>(4 pages)	For every site we provide summary statistics for the relevant climate indices and channel characteristics. Description of each variable is provided above the table.

Before detrending:

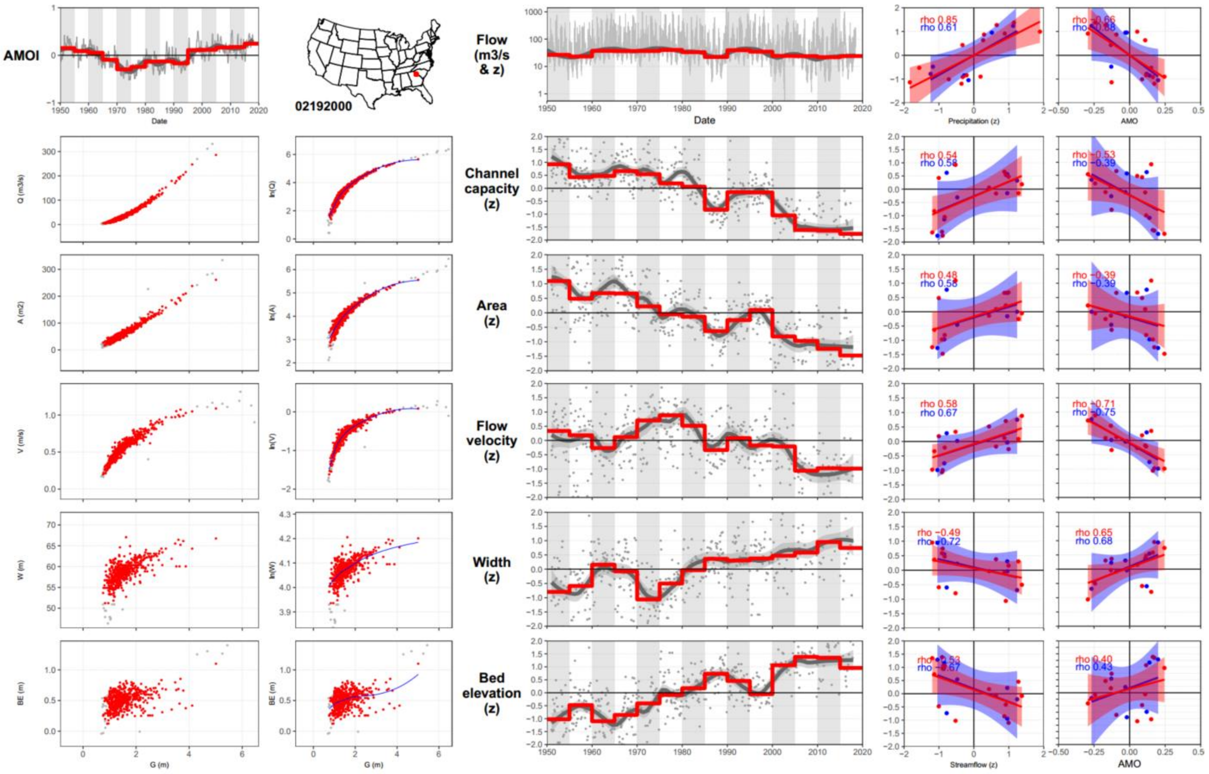

After detrending:

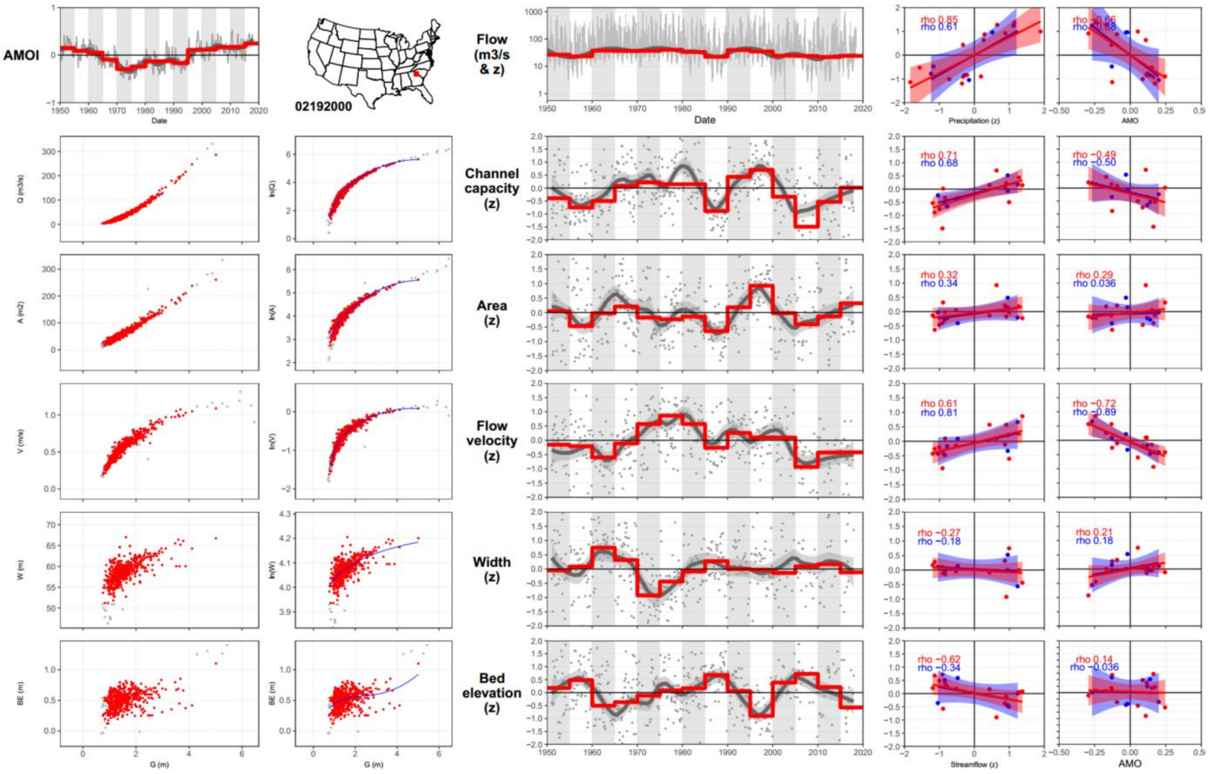

Supplementary Figure S1 | Relationships between the AMOI, precipitation, streamflow, and channel geometry at site 02192000, shown <u>before and after detrending</u> the channel transect data. See page 1 for the detailed caption to the above panels (same caption as for **Supp. Figs. 5-205**).

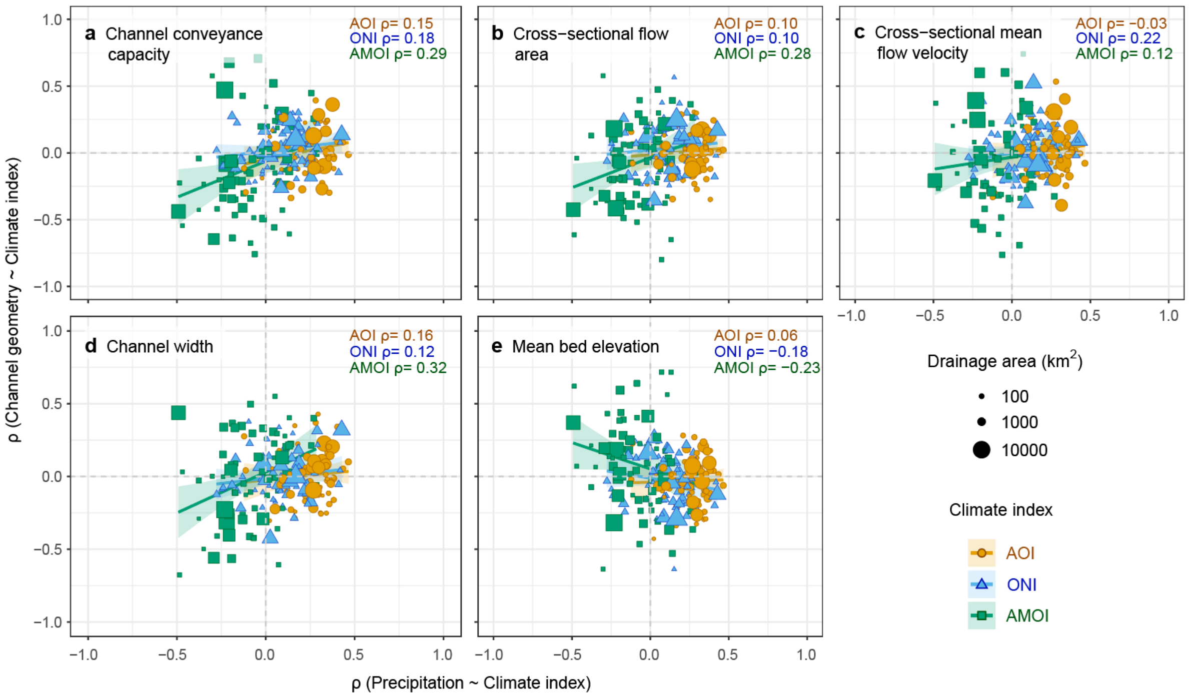

Supplementary Figure S2 | Same plot as Figure 4 in the main text, but the Spearman's rank correlation coefficients (*ρ*) on the x and y axes are computed using <u>2-year windows</u> (instead of 5-year windows).

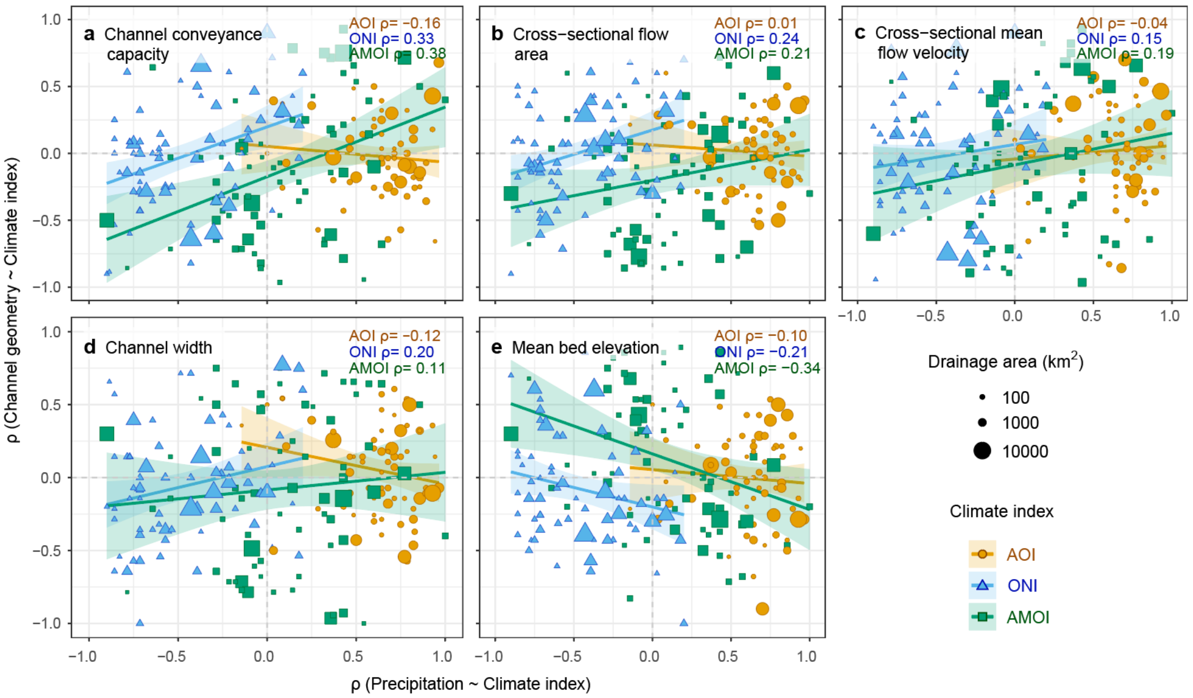

Supplementary Figure S3 | Same plot as Figure 4 in the main text, but the Spearman's rank correlation coefficients (*ρ*) on the x and y axes are computed using <u>10-year windows</u> (instead of 5-year windows).

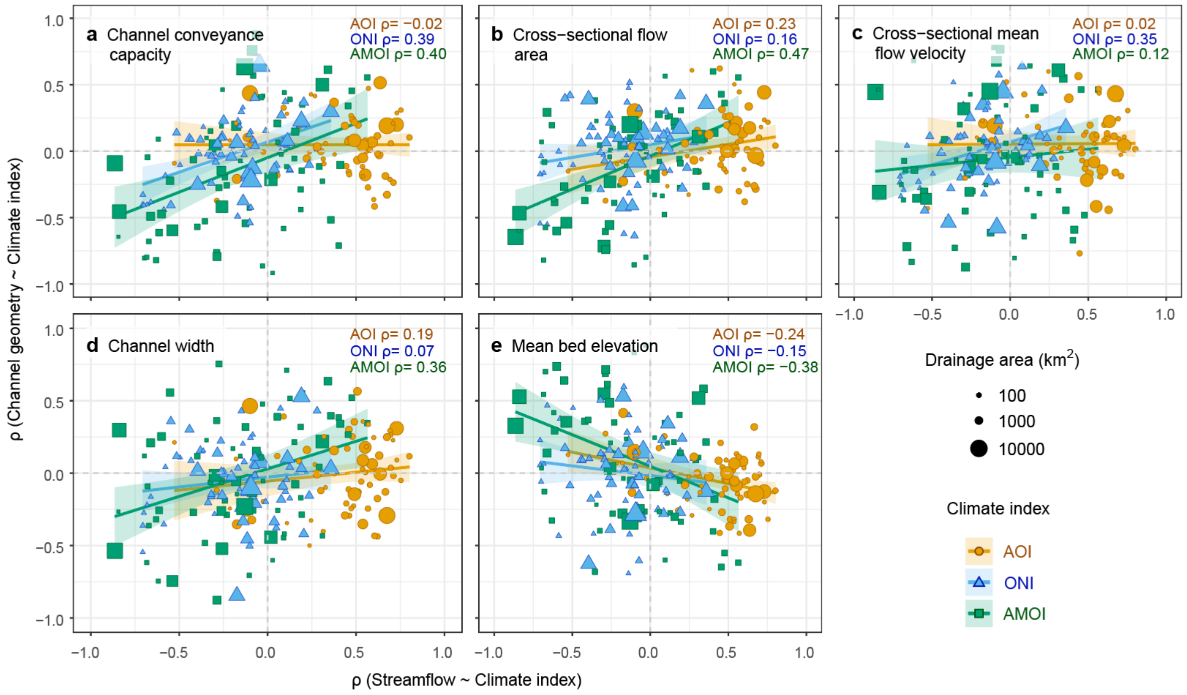

Supplementary Figure S4 | Same plot as Figure 4 in the main text, but showing the Spearman's rank correlation coefficient (*ρ*) between each climate index and <u>streamflow</u> on the x-axis (instead of precipitation).

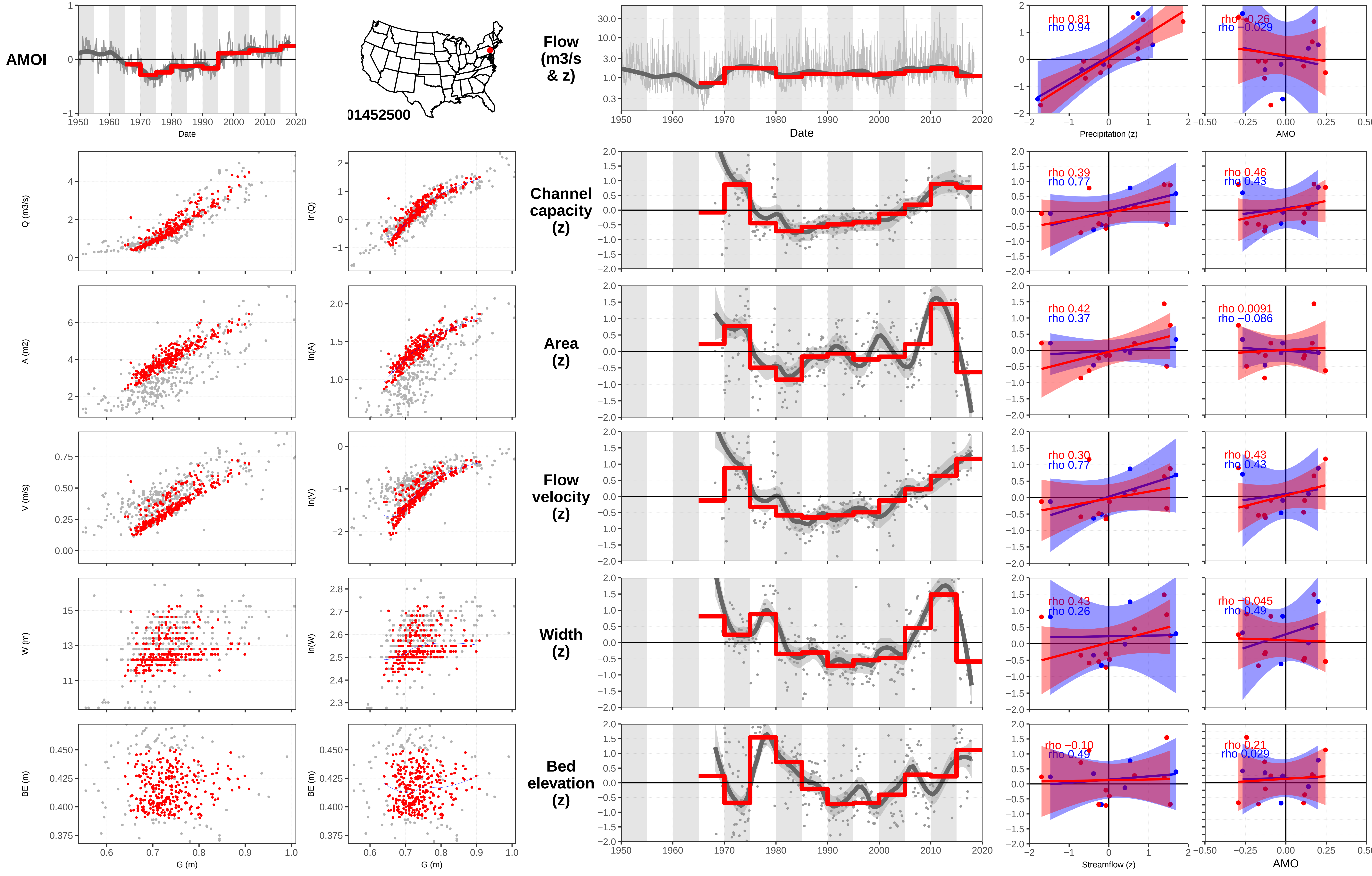

AMOI
01452500
Flow (m3/s & z)
Channel capacity (z)
Area (z)
Flow velocity (z)
Width (z)
Bed elevation (z)
Date
Precipitation (z)
AMO
Streamflow (z)
Q (m3/s)
ln(Q)
A (m2)
ln(A)
V (m/s)
ln(V)
W (m)
ln(W)
BE (m)
G (m)
rho 0.81
rho 0.94
rho −0.26
rho −0.029
rho 0.39
rho 0.77
rho 0.46
rho 0.43
rho 0.42
rho 0.37
rho 0.0091
rho −0.086
rho 0.30
rho 0.77
rho 0.43
rho 0.43
rho 0.43
rho 0.26
rho −0.045
rho 0.49
rho −0.10
rho 0.49
rho 0.21
rho 0.029

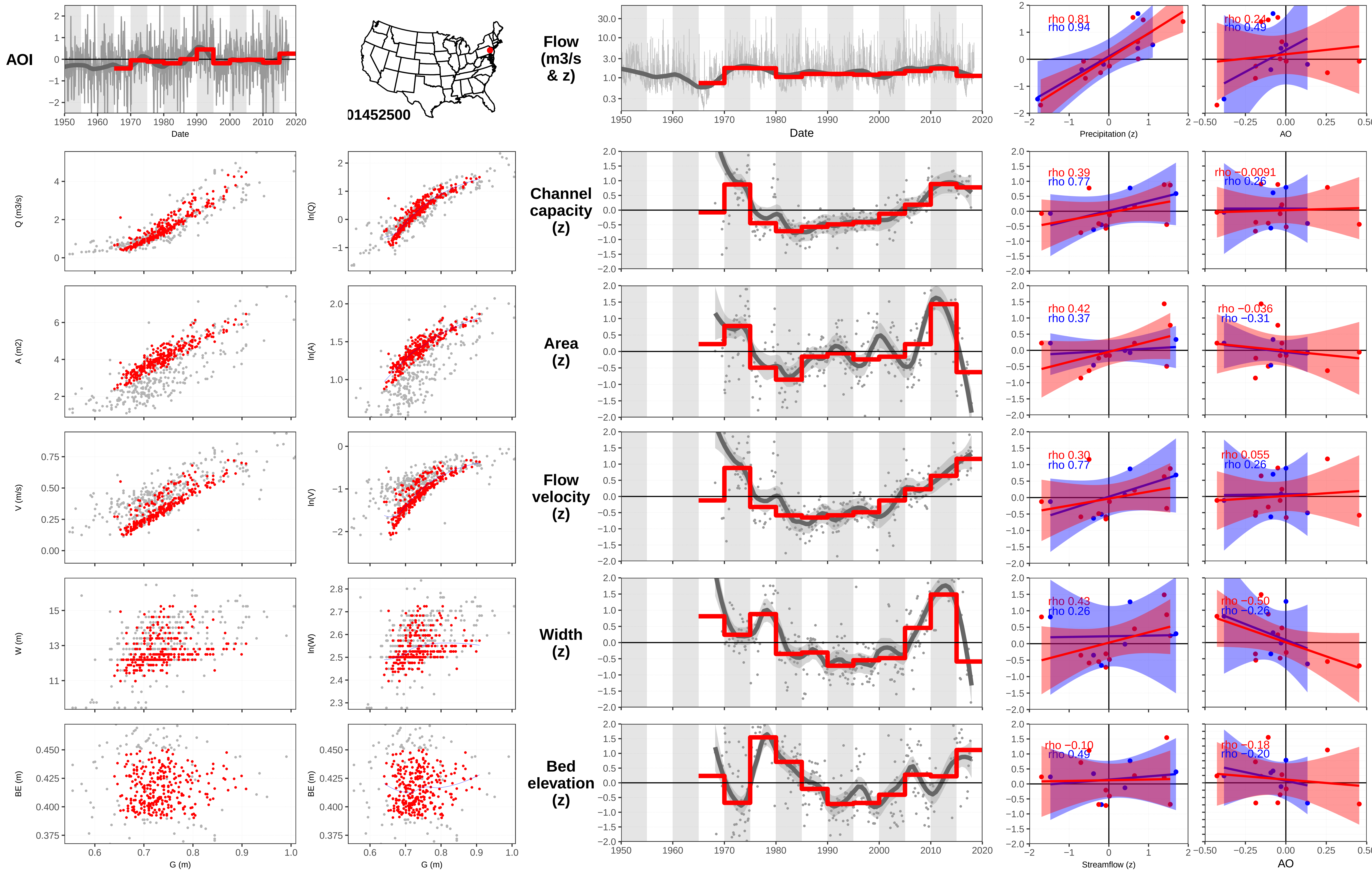

AOI
01452500
Flow (m3/s & z)
Channel capacity (z)
Area (z)
Flow velocity (z)
Width (z)
Bed elevation (z)
Date
Precipitation (z)
Streamflow (z)
AO
G (m)
Q (m3/s)
ln(Q)
A (m2)
ln(A)
V (m/s)
ln(V)
W (m)
ln(W)
BE (m)
rho 0.81
rho 0.94
rho 0.24
rho 0.49
rho 0.39
rho 0.77
rho −0.0091
rho 0.26
rho 0.42
rho 0.37
rho −0.036
rho −0.31
rho 0.30
rho 0.77
rho 0.055
rho 0.26
rho 0.43
rho 0.26
rho −0.50
rho −0.26
rho −0.10
rho 0.49
rho −0.18
rho −0.20

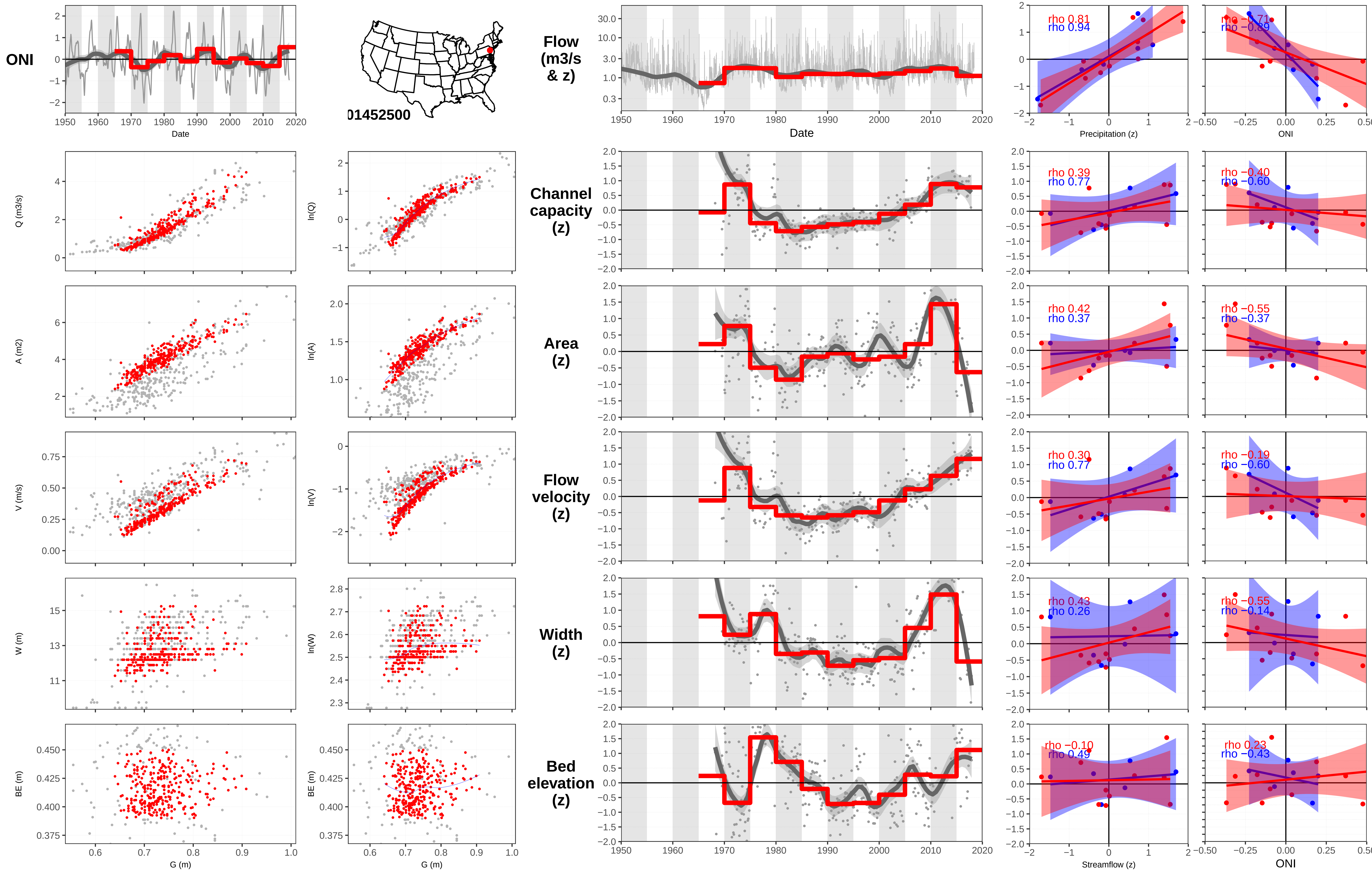

ONI
01452500
Flow (m3/s & z)
Channel capacity (z)
Area (z)
Flow velocity (z)
Width (z)
Bed elevation (z)
Date
Precipitation (z)
Streamflow (z)
ONI
G (m)
Q (m3/s)
ln(Q)
A (m2)
ln(A)
V (m/s)
ln(V)
W (m)
ln(W)
BE (m)
rho 0.81
rho 0.94
rho −0.71
rho −0.89
rho 0.39
rho 0.77
rho −0.40
rho −0.60
rho 0.42
rho 0.37
rho −0.55
rho −0.37
rho 0.30
rho 0.77
rho −0.19
rho −0.60
rho 0.43
rho 0.26
rho −0.55
rho −0.14
rho −0.10
rho 0.49
rho 0.23
rho −0.43

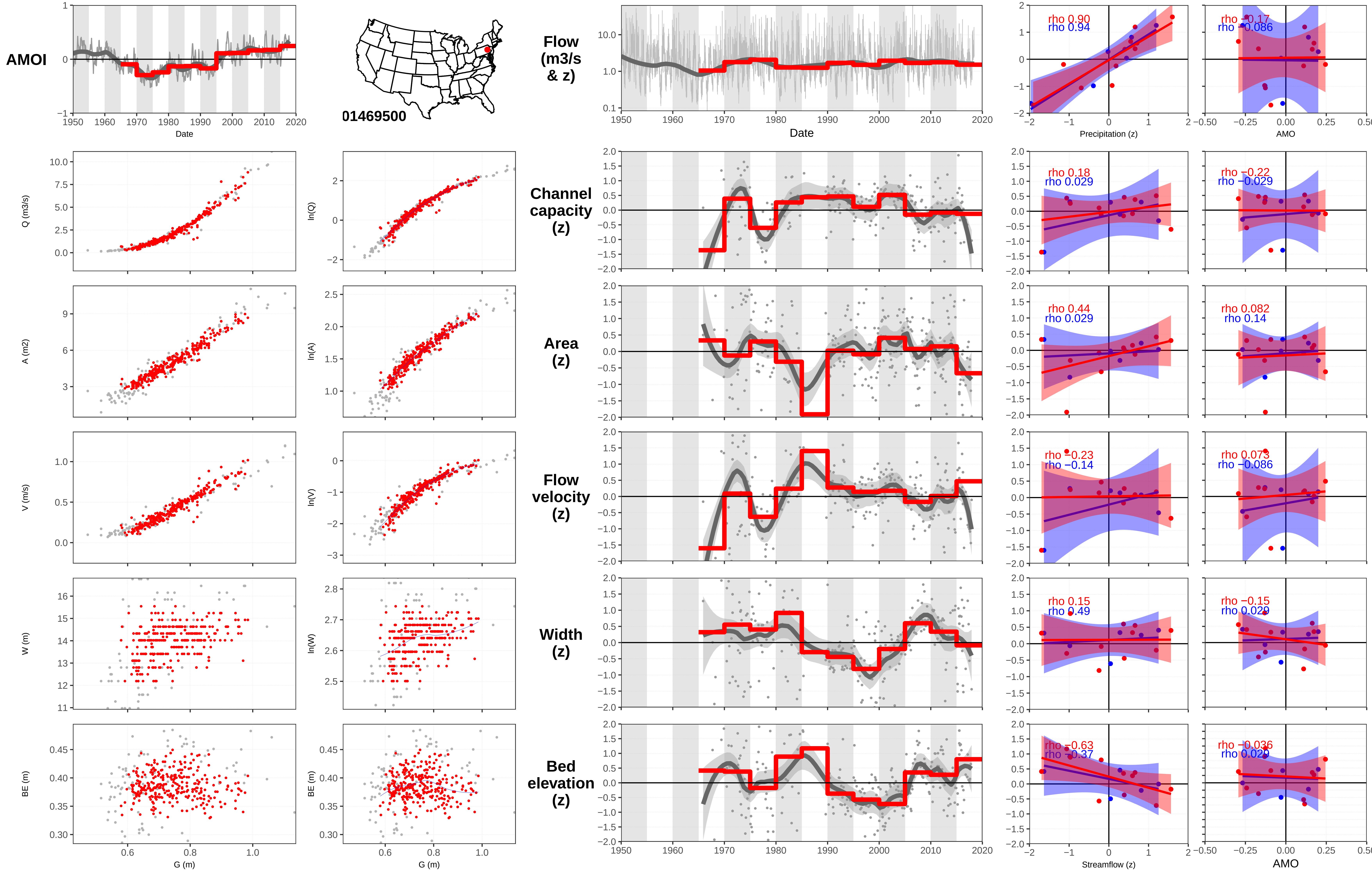

AMOI
01469500
Flow (m3/s & z)
Channel capacity (z)
Area (z)
Flow velocity (z)
Width (z)
Bed elevation (z)
Date
Precipitation (z)
AMO
Streamflow (z)
G (m)
Q (m3/s)
ln(Q)
A (m2)
ln(A)
V (m/s)
ln(V)
W (m)
ln(W)
BE (m)
rho 0.90
rho 0.94
rho −0.17
rho −0.086
rho 0.18
rho 0.029
rho −0.22
rho −0.029
rho 0.44
rho 0.029
rho 0.082
rho 0.14
rho −0.23
rho −0.14
rho 0.073
rho −0.086
rho 0.15
rho 0.49
rho −0.15
rho 0.029
rho −0.63
rho −0.37
rho −0.036
rho 0.029

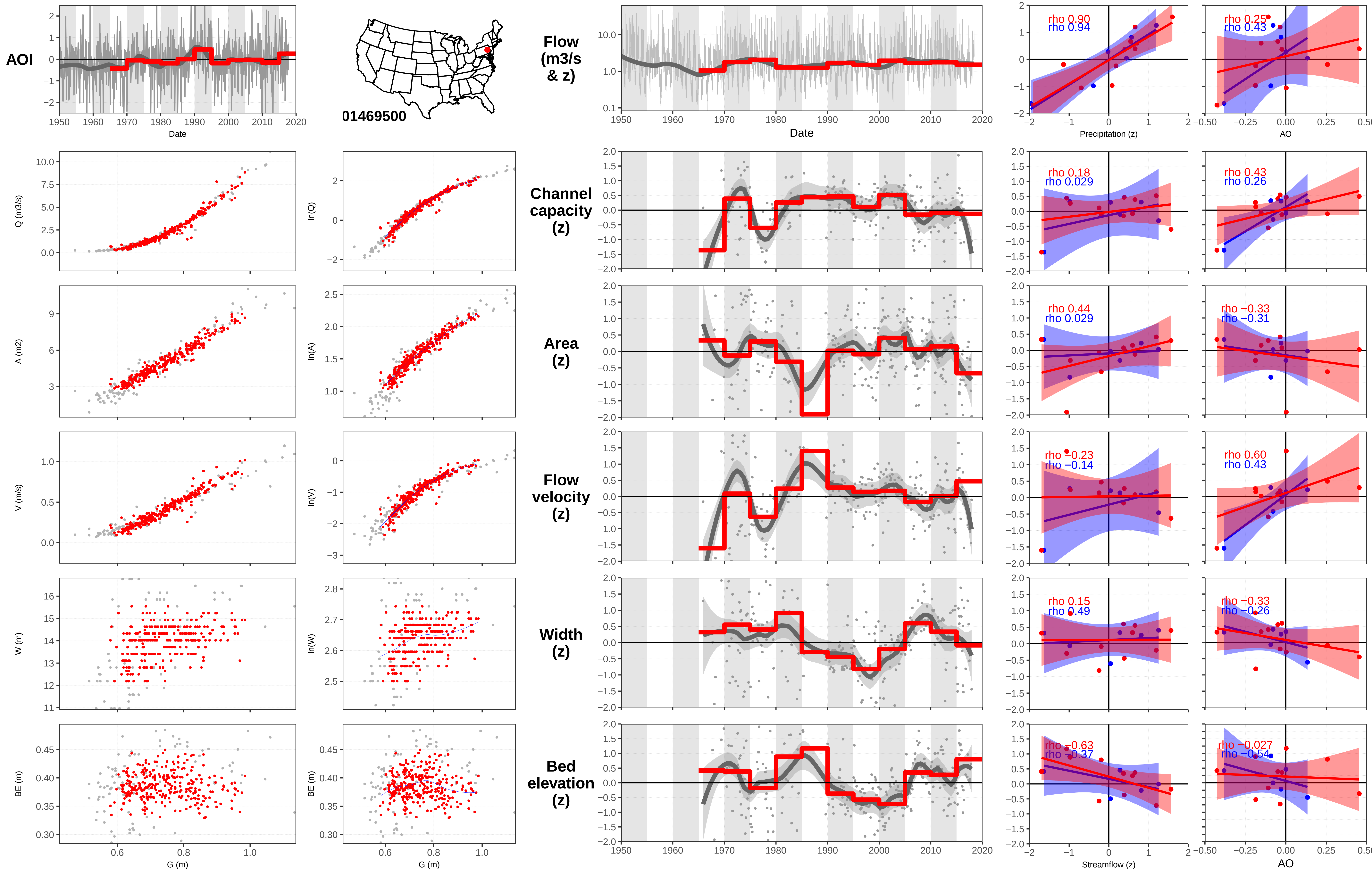

AOI
01469500
Flow (m3/s & z)
Channel capacity (z)
Area (z)
Flow velocity (z)
Width (z)
Bed elevation (z)
Date
Precipitation (z)
AO
Streamflow (z)
Q (m3/s)
ln(Q)
A (m2)
ln(A)
V (m/s)
ln(V)
W (m)
ln(W)
BE (m)
G (m)
rho 0.90
rho 0.94
rho 0.25
rho 0.43
rho 0.18
rho 0.029
rho 0.43
rho 0.26
rho 0.44
rho 0.029
rho −0.33
rho −0.31
rho −0.23
rho −0.14
rho 0.60
rho 0.43
rho 0.15
rho 0.49
rho −0.33
rho −0.26
rho −0.63
rho −0.37
rho −0.027
rho −0.54

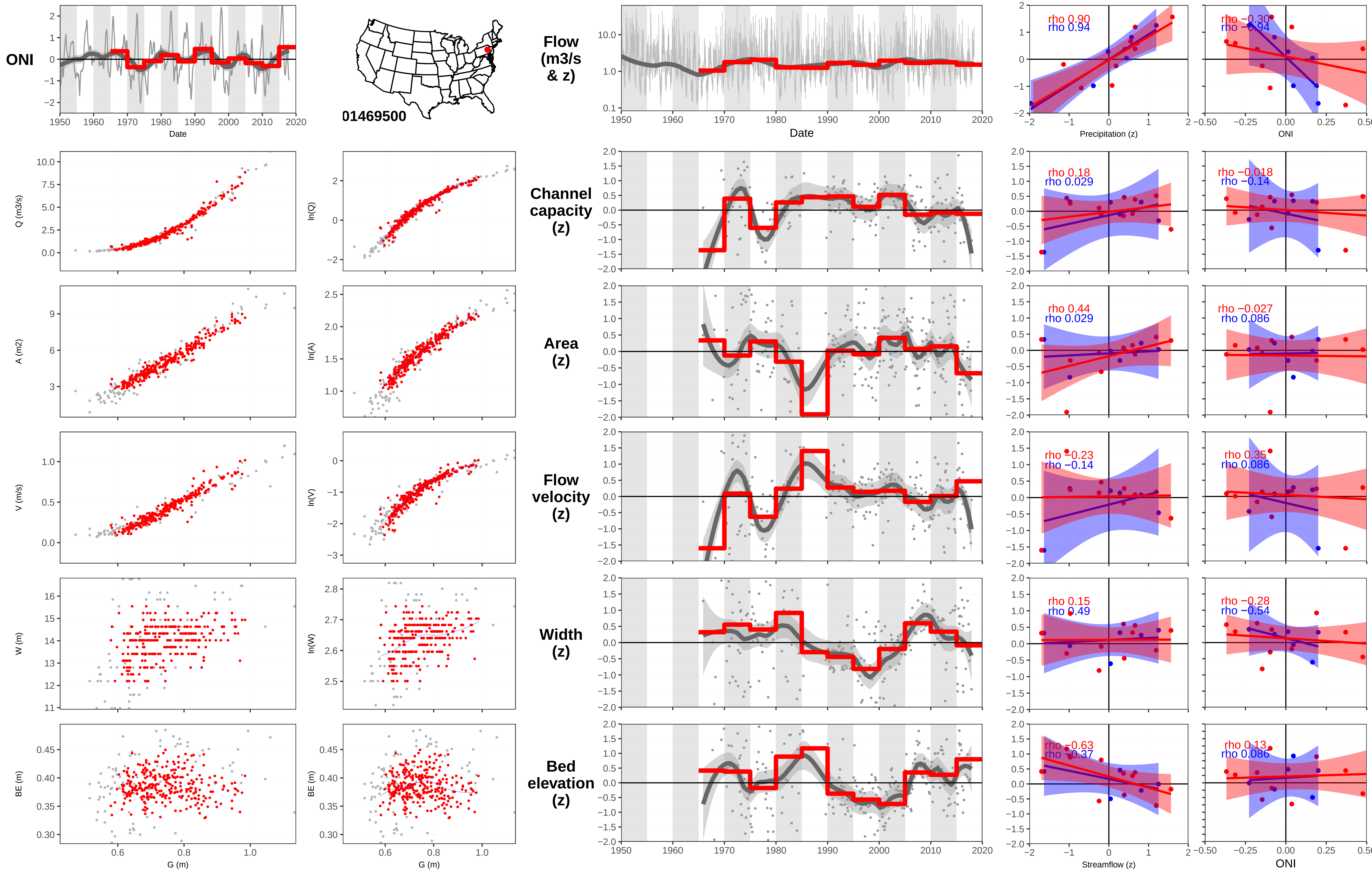

ONI
01469500
Flow (m3/s & z)
Channel capacity (z)
Area (z)
Flow velocity (z)
Width (z)
Bed elevation (z)
Date
Precipitation (z)
Streamflow (z)
ONI
Q (m3/s)
ln(Q)
A (m2)
ln(A)
V (m/s)
ln(V)
W (m)
ln(W)
BE (m)
G (m)
rho 0.90
rho 0.94
rho −0.30
rho −0.94
rho 0.18
rho 0.029
rho −0.018
rho −0.14
rho 0.44
rho 0.029
rho −0.027
rho 0.086
rho −0.23
rho −0.14
rho 0.35
rho 0.086
rho 0.15
rho 0.49
rho −0.28
rho −0.54
rho −0.63
rho −0.37
rho 0.13
rho 0.086

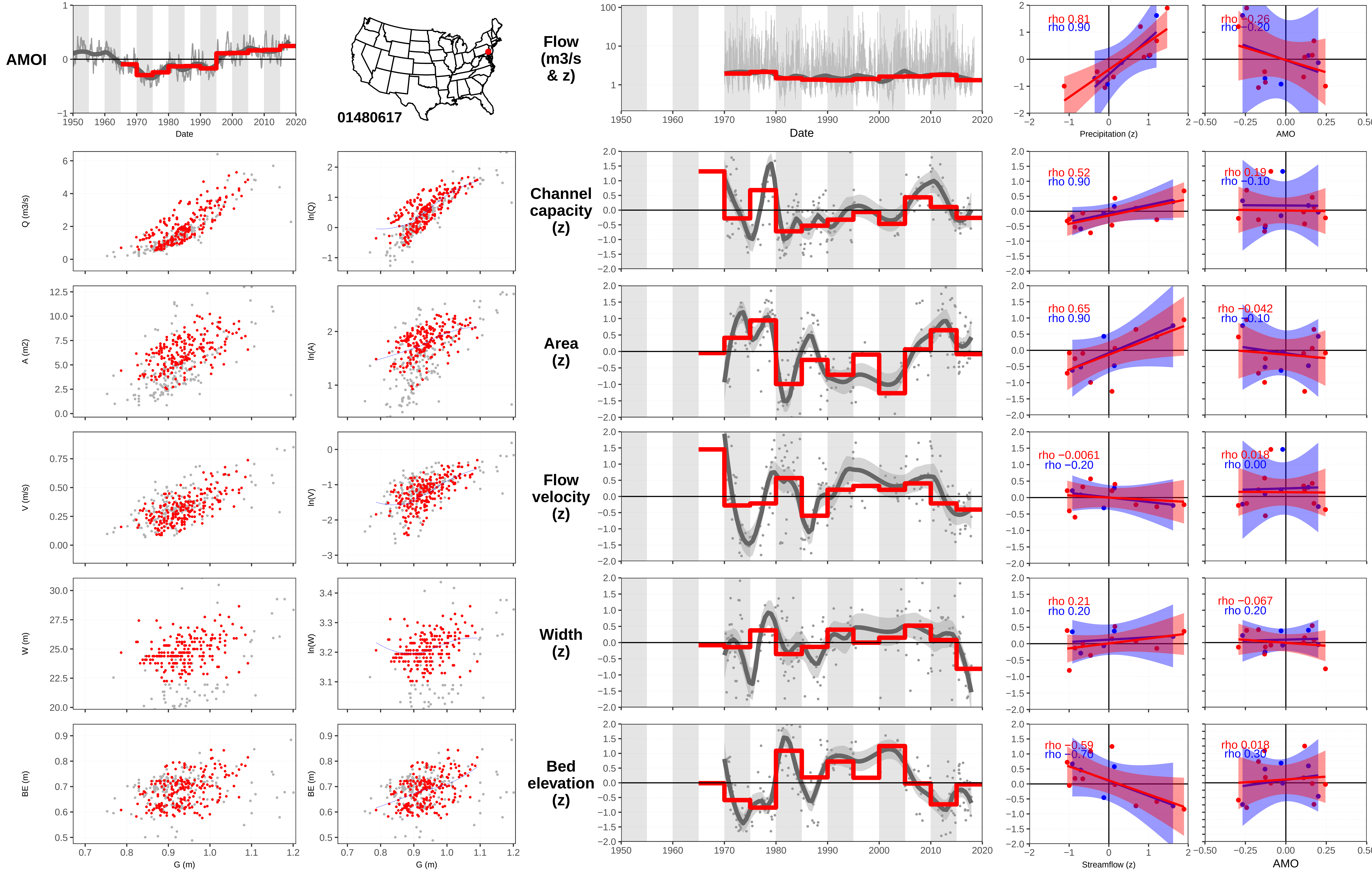
AMOI
01480617
Flow (m3/s & z)
Channel capacity (z)
Area (z)
Flow velocity (z)
Width (z)
Bed elevation (z)
Date
Precipitation (z)
Streamflow (z)
AMO
G (m)
Q (m3/s)
ln(Q)
A (m2)
ln(A)
V (m/s)
ln(V)
W (m)
ln(W)
BE (m)
rho 0.81
rho 0.90
rho −0.26
rho −0.20
rho 0.52
rho 0.90
rho 0.19
rho −0.10
rho 0.65
rho 0.90
rho −0.042
rho −0.10
rho −0.0061
rho −0.20
rho 0.018
rho 0.00
rho 0.21
rho 0.20
rho −0.067
rho 0.20
rho −0.59
rho −0.70
rho 0.018
rho 0.30

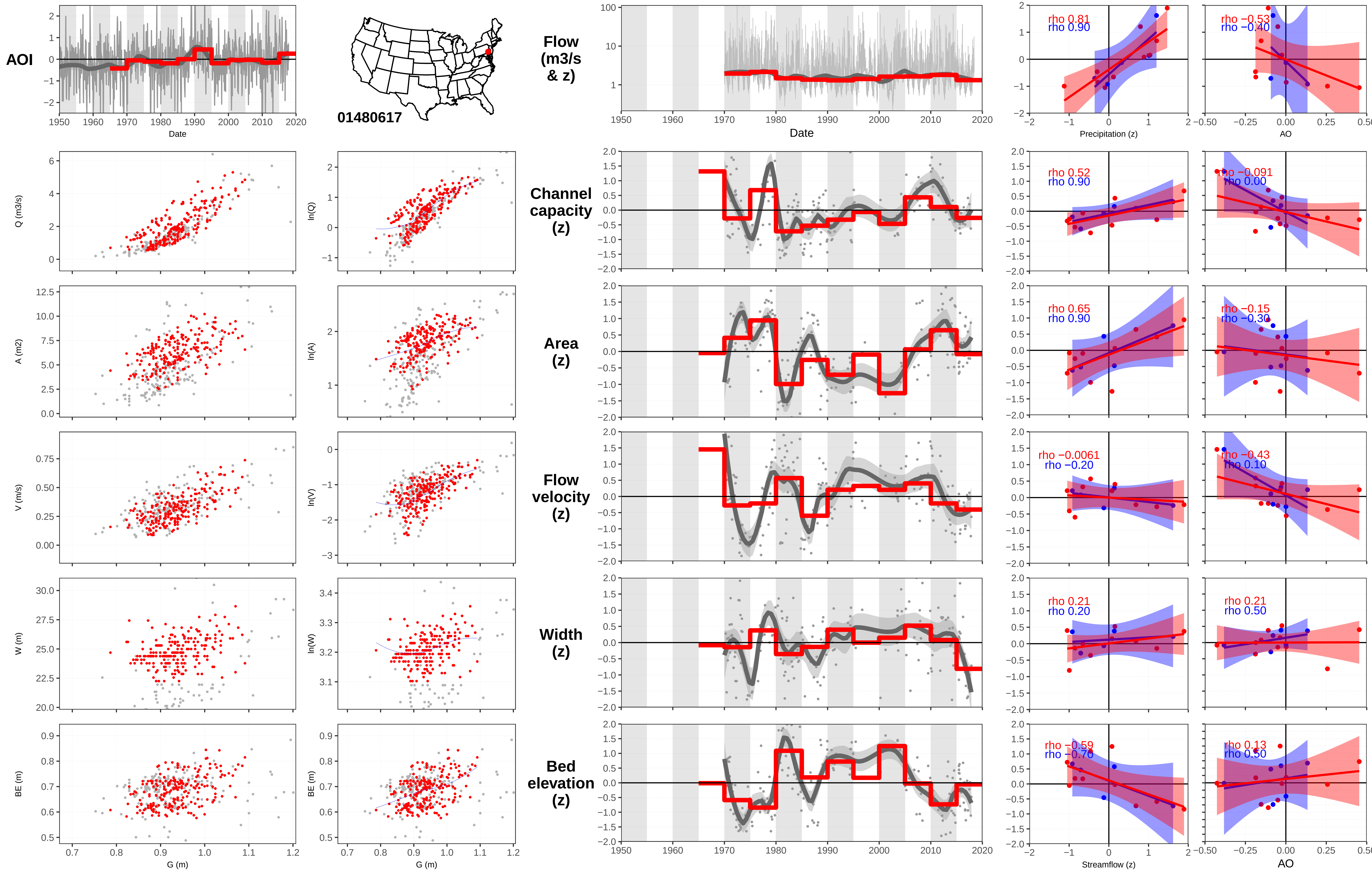

AOI
01480617
Flow (m3/s & z)
Date
Precipitation (z)
AO
rho 0.81
rho 0.90
rho −0.53
rho −0.40
Channel capacity (z)
Q (m3/s)
ln(Q)
rho 0.52
rho 0.90
rho −0.091
rho 0.00
Area (z)
A (m2)
ln(A)
rho 0.65
rho 0.90
rho −0.15
rho −0.30
Flow velocity (z)
V (m/s)
ln(V)
rho −0.0061
rho −0.20
rho −0.43
rho 0.10
Width (z)
W (m)
ln(W)
rho 0.21
rho 0.20
rho 0.21
rho 0.50
Bed elevation (z)
BE (m)
rho −0.59
rho −0.70
rho 0.13
rho 0.50
G (m)
Streamflow (z)

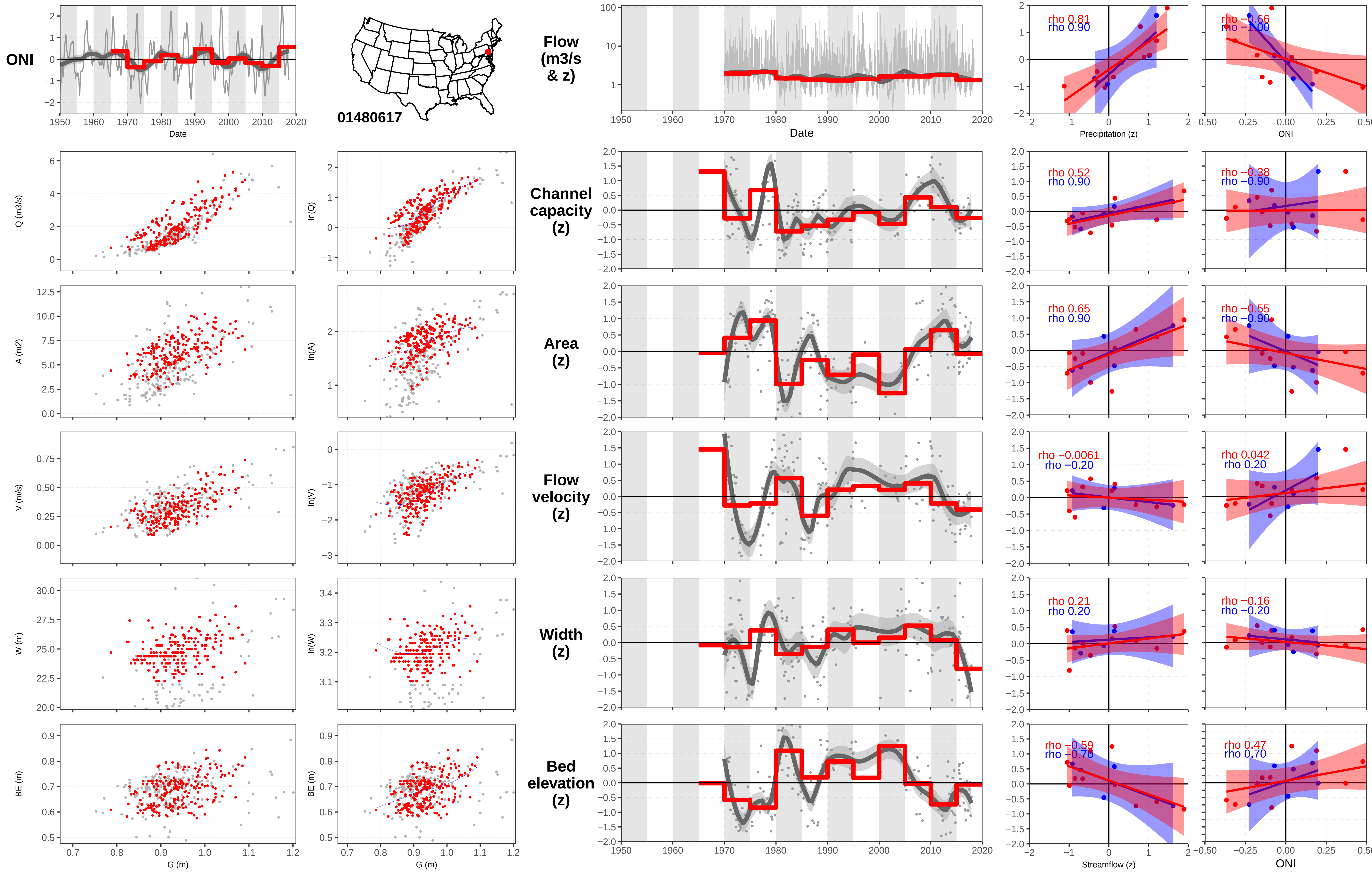
ONI
01480617
Flow (m3/s & z)
Channel capacity (z)
Area (z)
Flow velocity (z)
Width (z)
Bed elevation (z)
Date
Precipitation (z)
ONI
Streamflow (z)
G (m)
Q (m3/s)
ln(Q)
A (m2)
ln(A)
V (m/s)
ln(V)
W (m)
ln(W)
BE (m)
rho 0.81
rho 0.90
rho −0.66
rho −1.00
rho 0.52
rho 0.90
rho −0.38
rho −0.90
rho 0.65
rho 0.90
rho −0.55
rho −0.90
rho −0.0061
rho −0.20
rho 0.042
rho 0.20
rho 0.21
rho 0.20
rho −0.16
rho −0.20
rho −0.59
rho −0.70
rho 0.47
rho 0.70

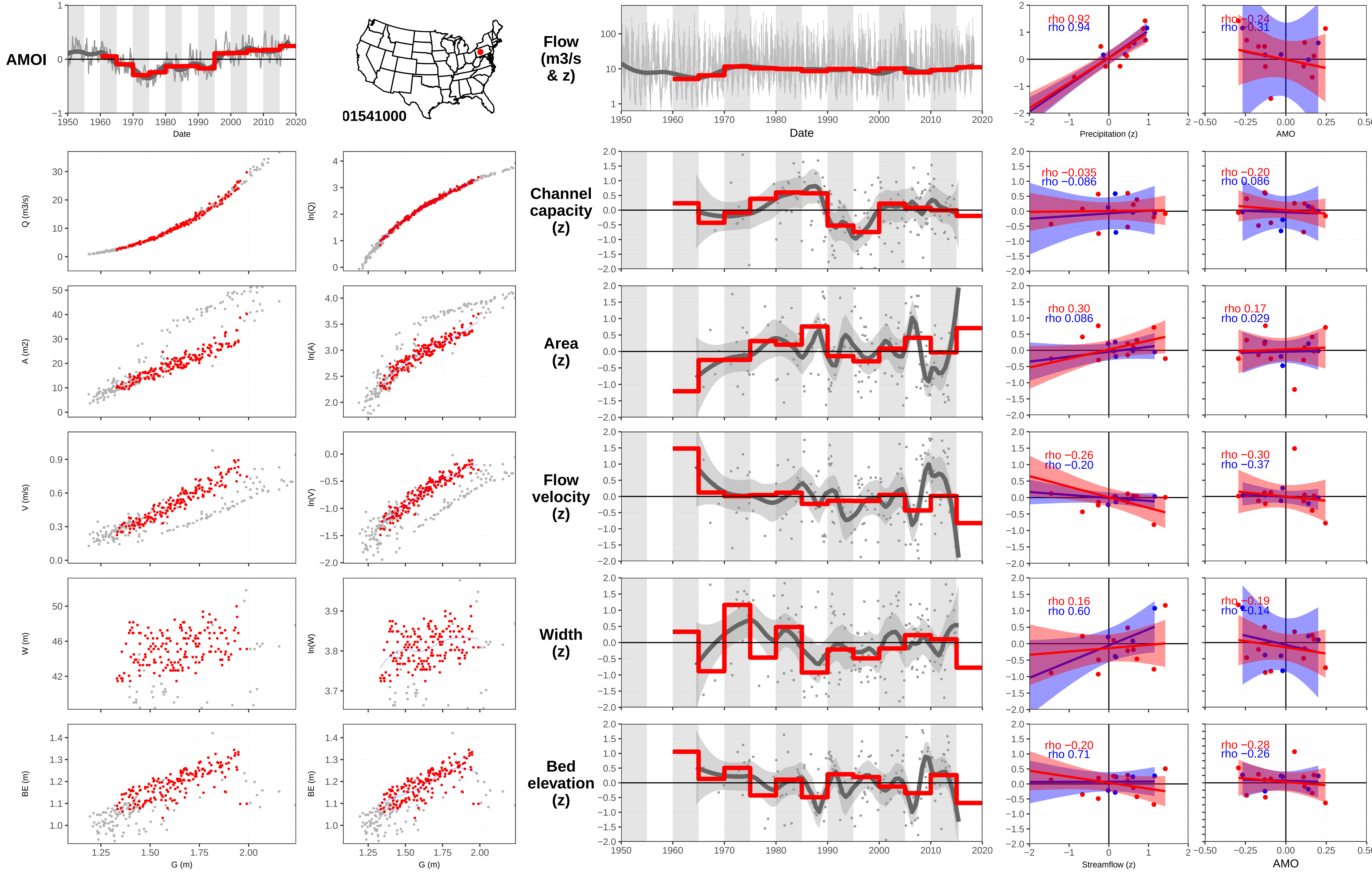
AMOI
01541000
Flow (m3/s & z)
Channel capacity (z)
Area (z)
Flow velocity (z)
Width (z)
Bed elevation (z)
Date
Precipitation (z)
AMO
Streamflow (z)
G (m)
Q (m3/s)
ln(Q)
A (m2)
ln(A)
V (m/s)
ln(V)
W (m)
ln(W)
BE (m)
rho 0.92
rho 0.94
rho −0.24
rho −0.31
rho −0.035
rho −0.086
rho −0.20
rho 0.086
rho 0.30
rho 0.086
rho 0.17
rho 0.029
rho −0.26
rho −0.20
rho −0.30
rho −0.37
rho 0.16
rho 0.60
rho −0.19
rho −0.14
rho −0.20
rho 0.71
rho −0.28
rho −0.26

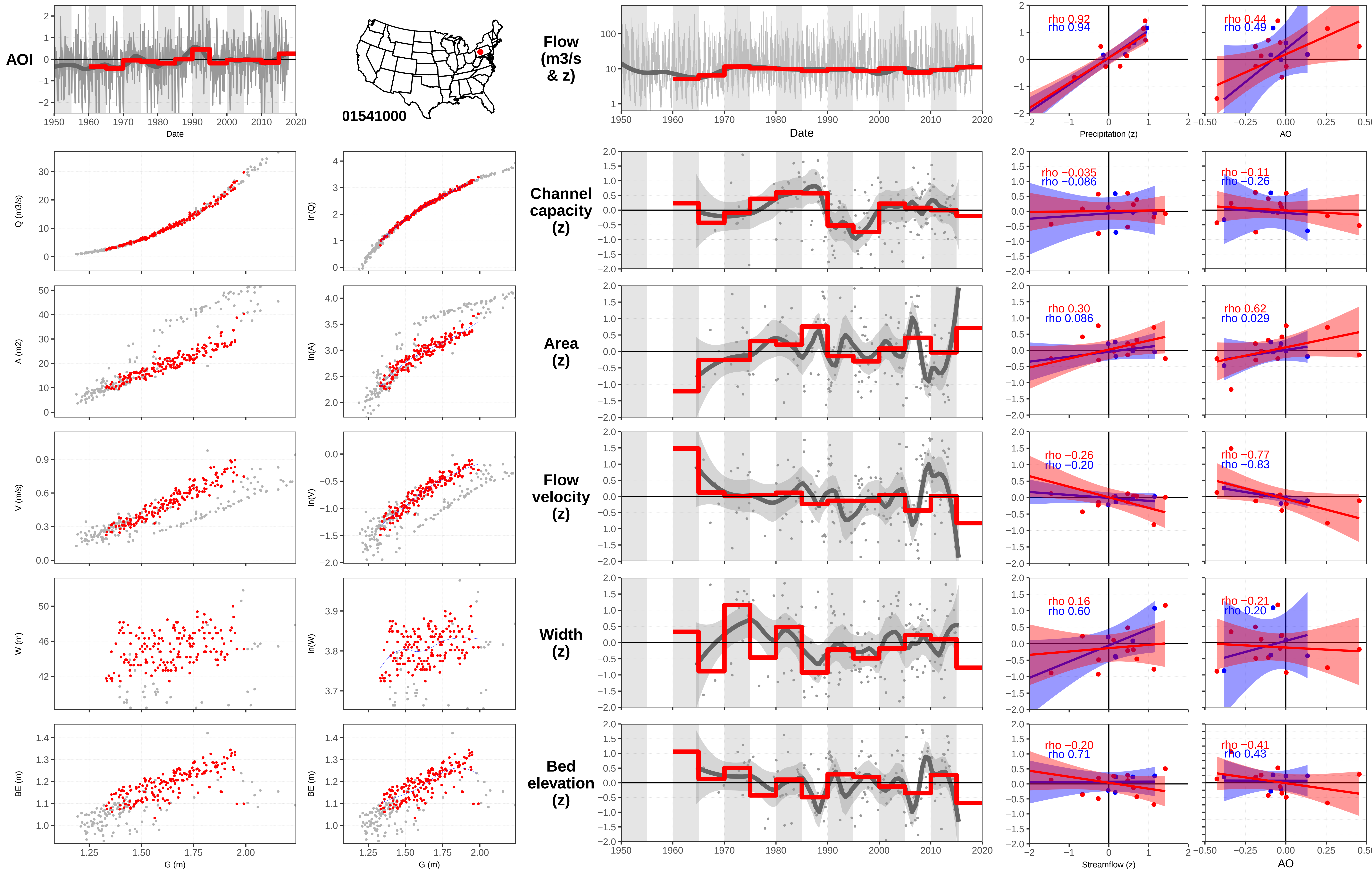

AOI
01541000
Flow (m3/s & z)
Channel capacity (z)
Area (z)
Flow velocity (z)
Width (z)
Bed elevation (z)
Date
Precipitation (z)
AO
Streamflow (z)
G (m)
Q (m3/s)
ln(Q)
A (m2)
ln(A)
V (m/s)
ln(V)
W (m)
ln(W)
BE (m)
rho 0.92
rho 0.94
rho 0.44
rho 0.49
rho −0.035
rho −0.086
rho −0.11
rho −0.26
rho 0.30
rho 0.086
rho 0.62
rho 0.029
rho −0.26
rho −0.20
rho −0.77
rho −0.83
rho 0.16
rho 0.60
rho −0.21
rho 0.20
rho −0.20
rho 0.71
rho −0.41
rho 0.43

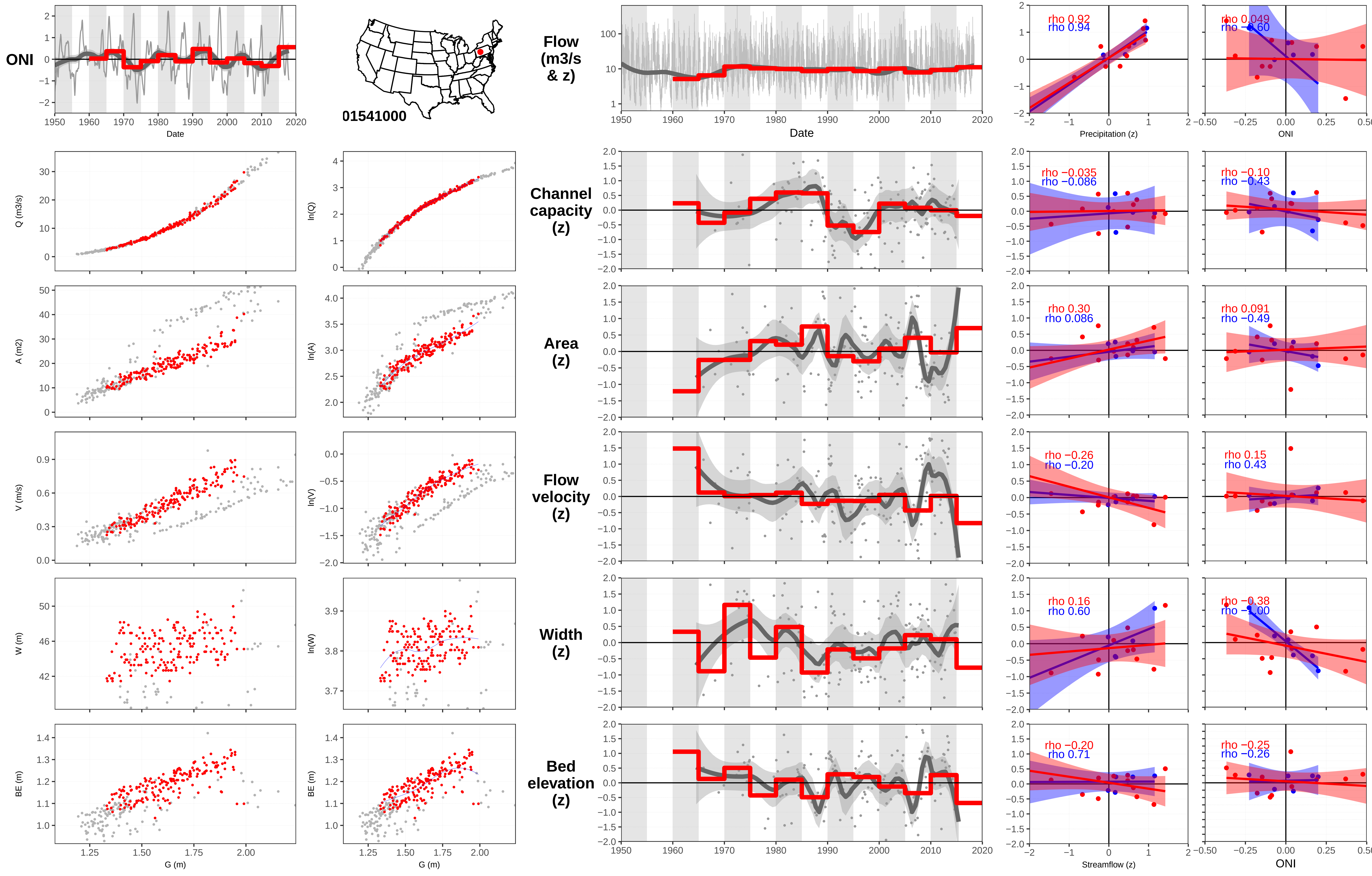

ONI
Date
01541000
Flow (m3/s & z)
Date
rho 0.92
rho 0.94
Precipitation (z)
rho 0.049
rho −0.60
ONI
Q (m3/s)
ln(Q)
Channel capacity (z)
rho −0.035
rho −0.086
rho −0.10
rho −0.43
A (m2)
ln(A)
Area (z)
rho 0.30
rho 0.086
rho 0.091
rho −0.49
V (m/s)
ln(V)
Flow velocity (z)
rho −0.26
rho −0.20
rho 0.15
rho 0.43
W (m)
ln(W)
Width (z)
rho 0.16
rho 0.60
rho −0.38
rho −1.00
BE (m)
BE (m)
Bed elevation (z)
rho −0.20
rho 0.71
rho −0.25
rho −0.26
G (m)
G (m)
Streamflow (z)
ONI

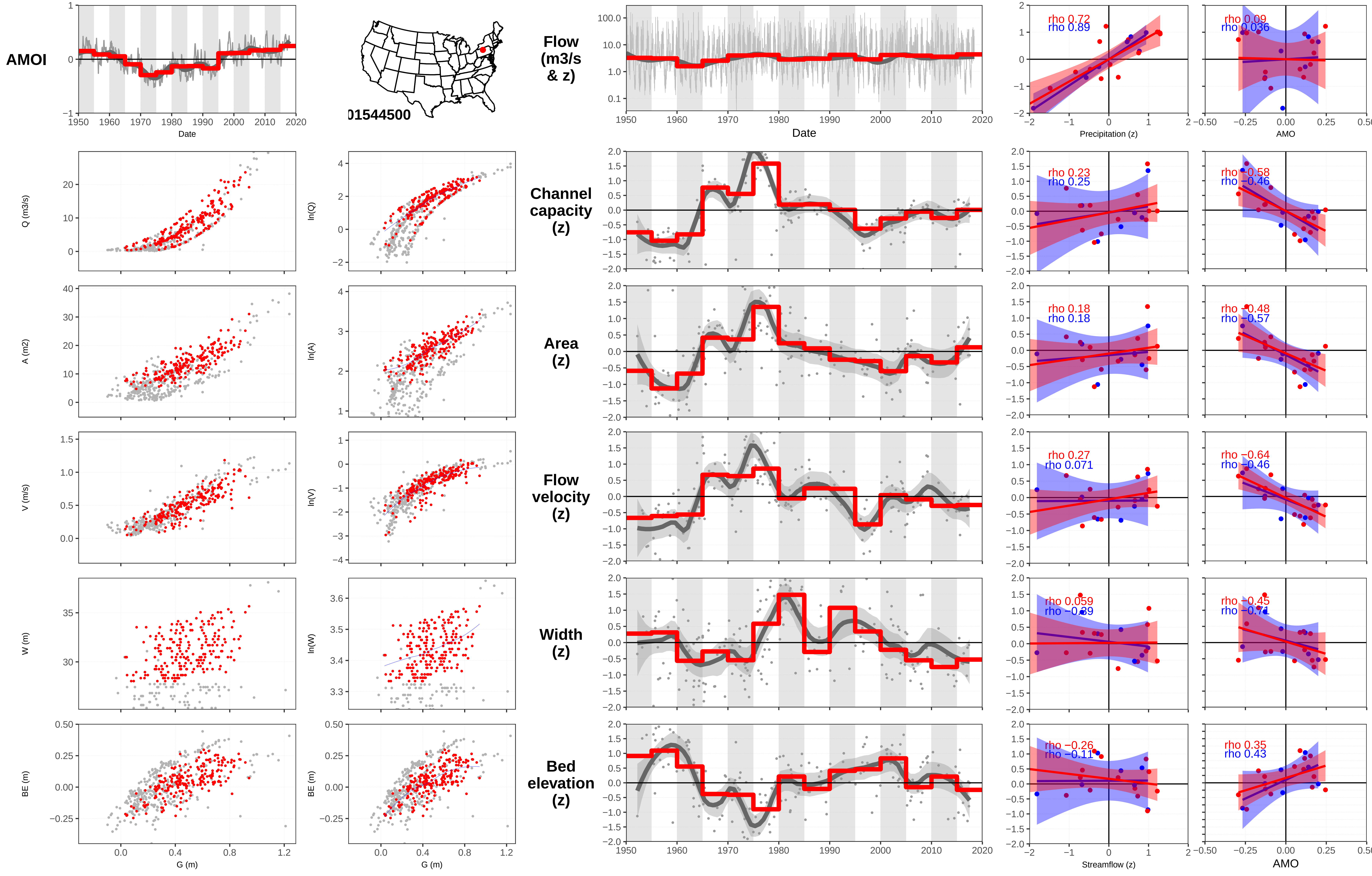
AMOI
Date
01544500
Flow (m3/s & z)
Date
rho 0.72
rho 0.89
Precipitation (z)
rho 0.09
rho 0.036
AMO
Q (m3/s)
ln(Q)
Channel capacity (z)
rho 0.23
rho 0.25
rho −0.58
rho −0.46
A (m2)
ln(A)
Area (z)
rho 0.18
rho 0.18
rho −0.48
rho −0.57
V (m/s)
ln(V)
Flow velocity (z)
rho 0.27
rho 0.071
rho −0.64
rho −0.46
W (m)
ln(W)
Width (z)
rho 0.059
rho −0.39
rho −0.45
rho −0.71
BE (m)
BE (m)
Bed elevation (z)
rho −0.26
rho −0.11
rho 0.35
rho 0.43
G (m)
G (m)
Streamflow (z)
AMO

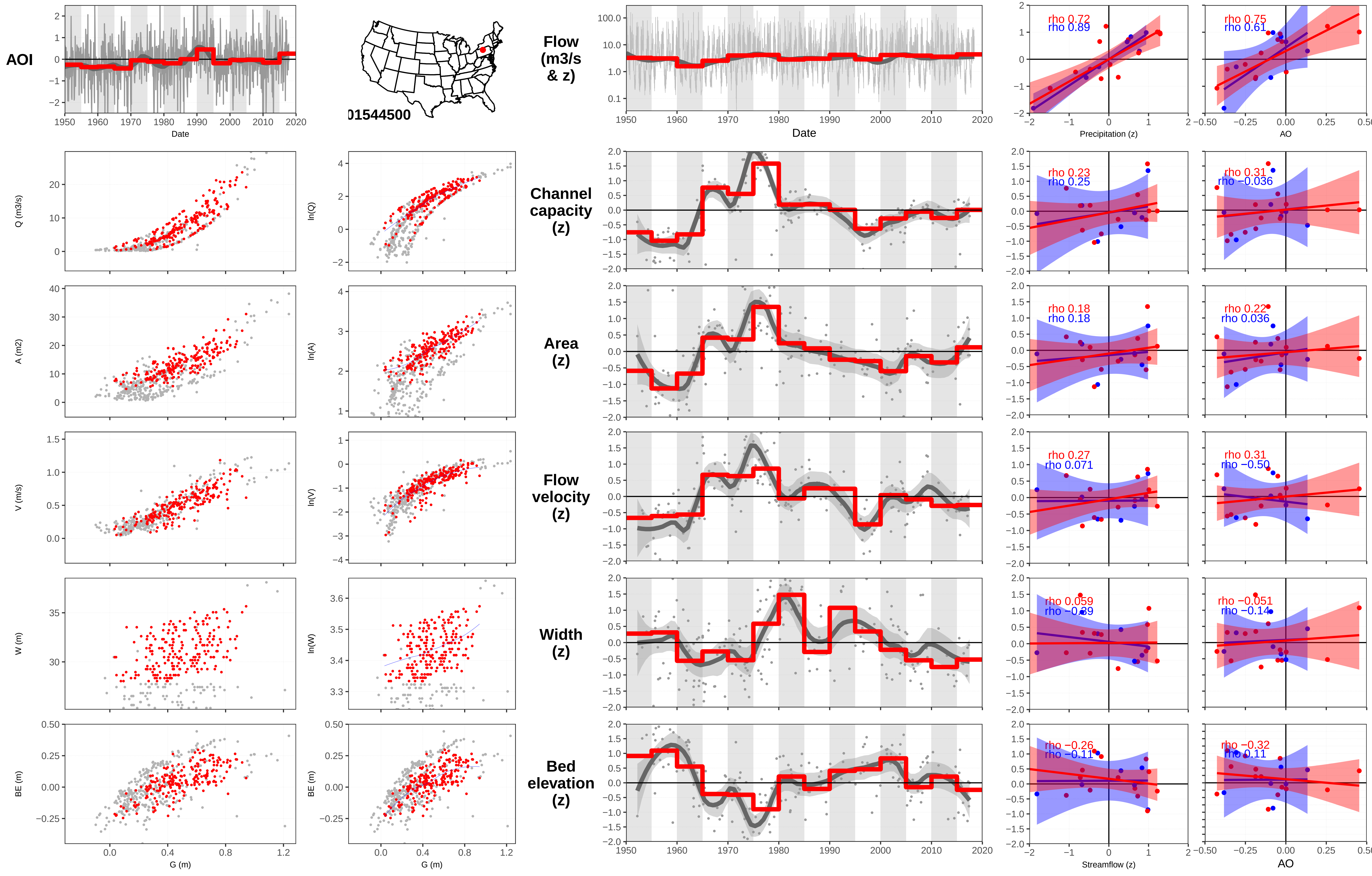
AOI
01544500
Flow (m3/s & z)
Channel capacity (z)
Area (z)
Flow velocity (z)
Width (z)
Bed elevation (z)
Date
Precipitation (z)
AO
Streamflow (z)
G (m)
Q (m3/s)
ln(Q)
A (m2)
ln(A)
V (m/s)
ln(V)
W (m)
ln(W)
BE (m)
rho 0.72
rho 0.89
rho 0.75
rho 0.61
rho 0.23
rho 0.25
rho 0.31
rho −0.036
rho 0.18
rho 0.18
rho 0.22
rho 0.036
rho 0.27
rho 0.071
rho 0.31
rho −0.50
rho 0.059
rho −0.39
rho −0.051
rho −0.14
rho −0.26
rho −0.11
rho −0.32
rho 0.11

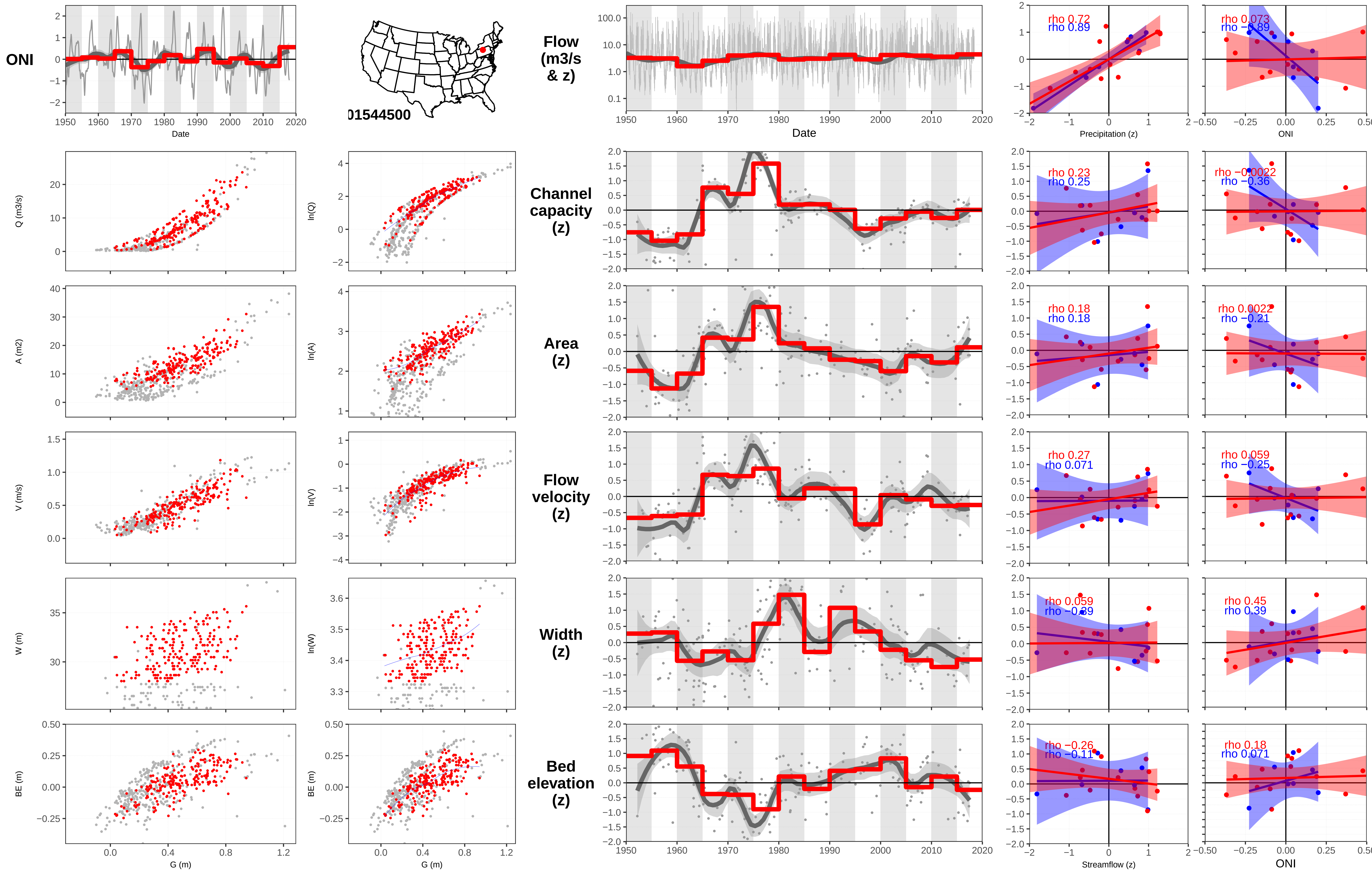
ONI
01544500
Flow (m3/s & z)
Channel capacity (z)
Area (z)
Flow velocity (z)
Width (z)
Bed elevation (z)
Date
Precipitation (z)
ONI
Streamflow (z)
Q (m3/s)
ln(Q)
A (m2)
ln(A)
V (m/s)
ln(V)
W (m)
ln(W)
BE (m)
G (m)
rho 0.72
rho 0.89
rho 0.073
rho −0.89
rho 0.23
rho 0.25
rho −0.0022
rho −0.36
rho 0.18
rho 0.18
rho 0.0022
rho −0.21
rho 0.27
rho 0.071
rho 0.059
rho −0.25
rho 0.059
rho −0.39
rho 0.45
rho 0.39
rho −0.26
rho −0.11
rho 0.18
rho 0.071

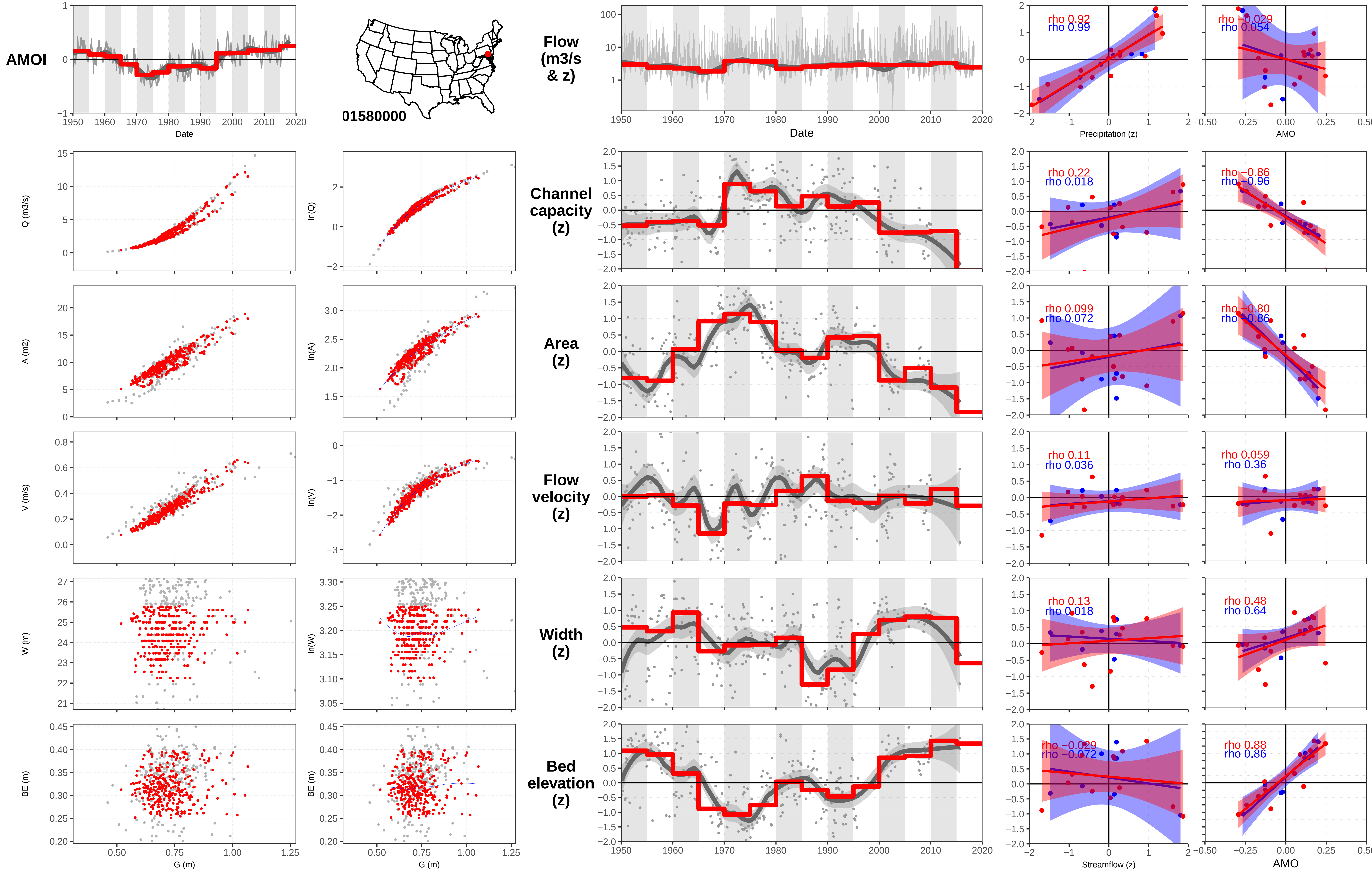
AMOI
Date
01580000
Flow (m3/s & z)
Date
Precipitation (z)
AMO
rho 0.92
rho 0.99
rho −0.029
rho 0.054
Q (m3/s)
ln(Q)
Channel capacity (z)
rho 0.22
rho 0.018
rho −0.86
rho −0.96
A (m2)
ln(A)
Area (z)
rho 0.099
rho 0.072
rho −0.80
rho −0.86
V (m/s)
ln(V)
Flow velocity (z)
rho 0.11
rho 0.036
rho 0.059
rho 0.36
W (m)
ln(W)
Width (z)
rho 0.13
rho 0.018
rho 0.48
rho 0.64
BE (m)
Bed elevation (z)
rho −0.029
rho −0.072
rho 0.88
rho 0.86
G (m)
G (m)
Streamflow (z)
AMO

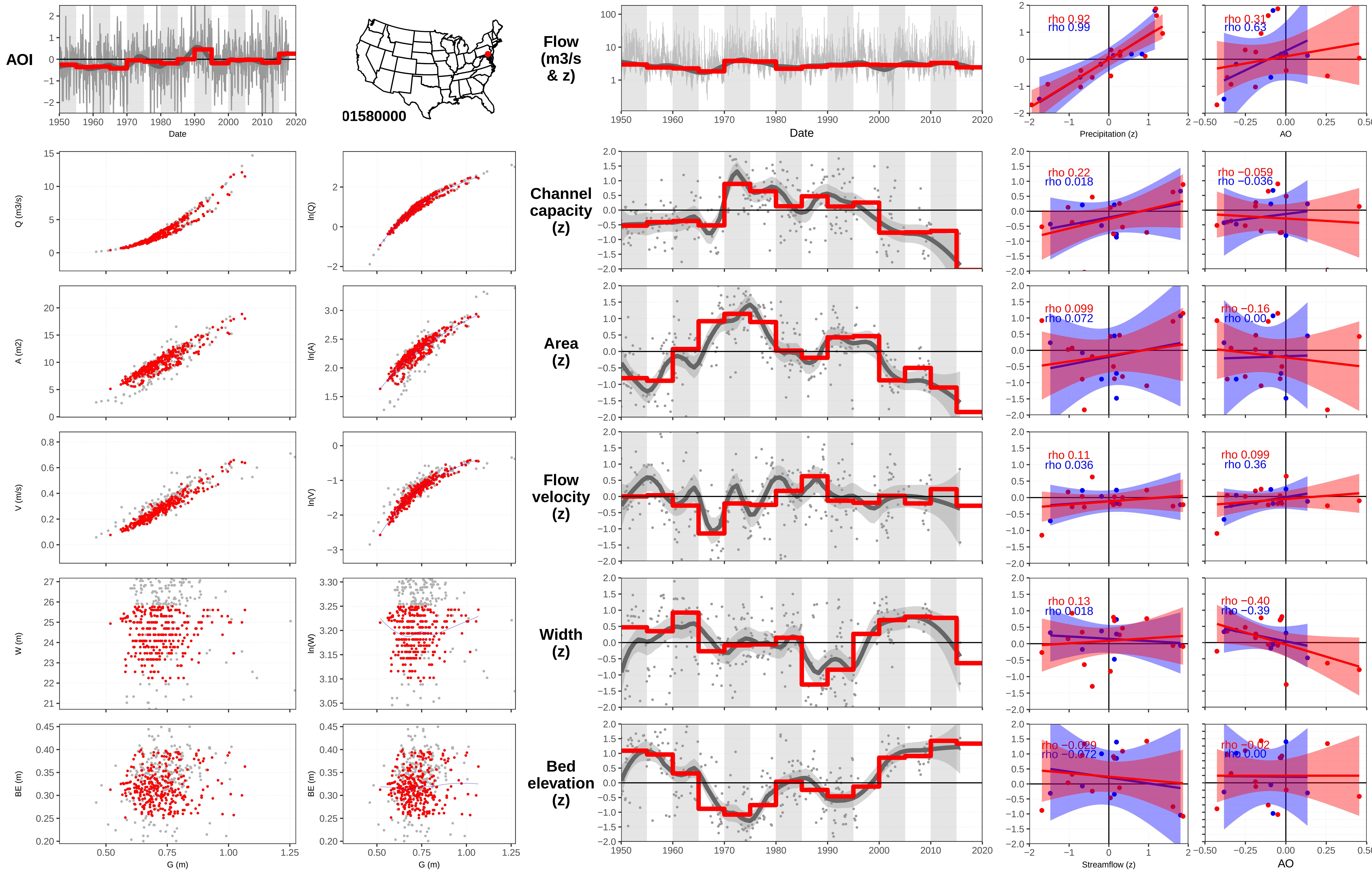

AOI
01580000
Flow (m3/s & z)
Channel capacity (z)
Area (z)
Flow velocity (z)
Width (z)
Bed elevation (z)
Date
Precipitation (z)
Streamflow (z)
AO
Q (m3/s)
ln(Q)
A (m2)
ln(A)
V (m/s)
ln(V)
W (m)
ln(W)
BE (m)
G (m)
rho 0.92
rho 0.99
rho 0.31
rho 0.63
rho 0.22
rho 0.018
rho −0.059
rho −0.036
rho 0.099
rho 0.072
rho −0.16
rho 0.00
rho 0.11
rho 0.036
rho 0.099
rho 0.36
rho 0.13
rho 0.018
rho −0.40
rho −0.39
rho −0.029
rho −0.072
rho −0.02
rho 0.00

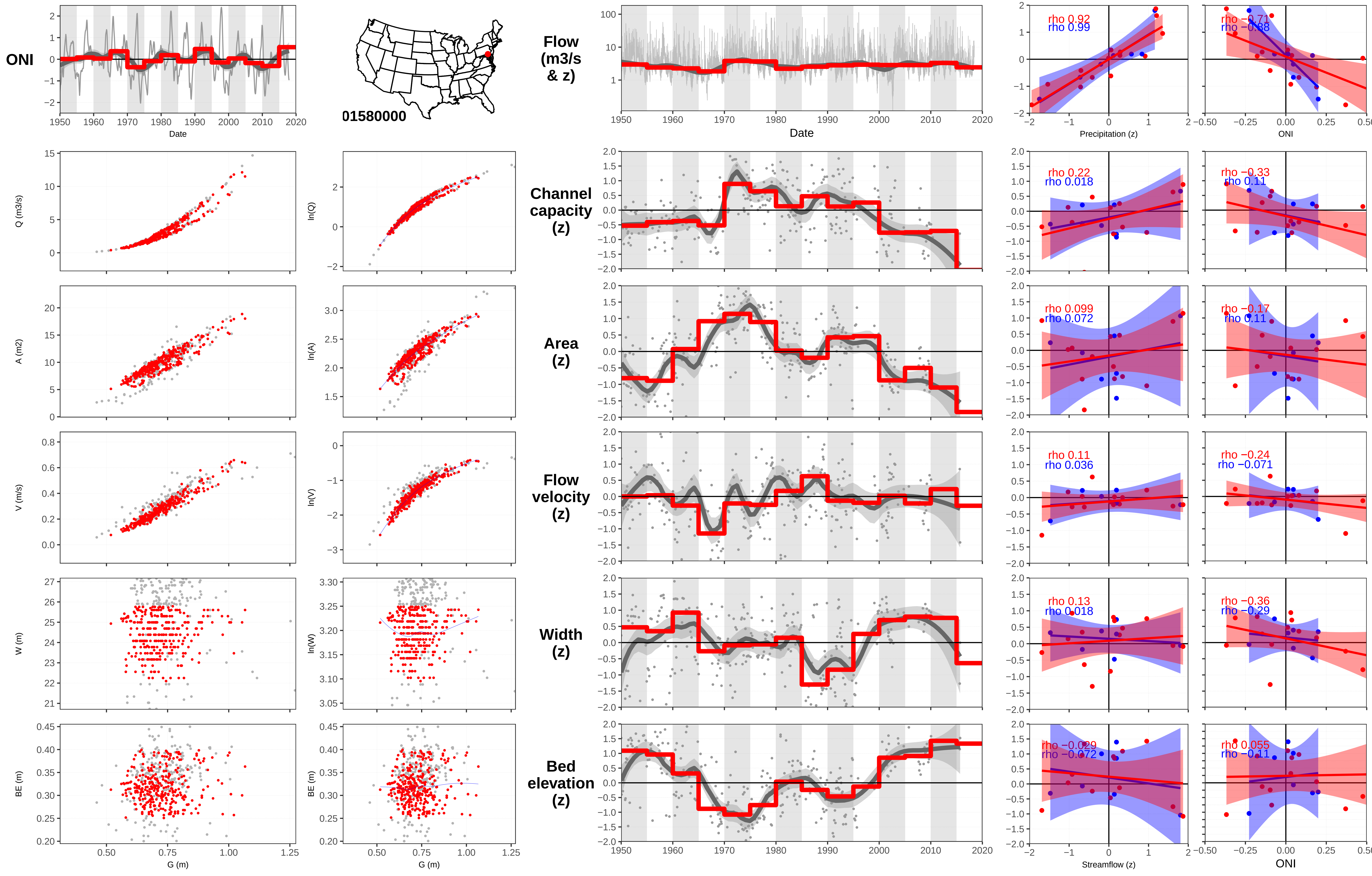
ONI
01580000
Flow (m3/s & z)
Channel capacity (z)
Area (z)
Flow velocity (z)
Width (z)
Bed elevation (z)
Date
Precipitation (z)
Streamflow (z)
ONI
G (m)
Q (m3/s)
ln(Q)
A (m2)
ln(A)
V (m/s)
ln(V)
W (m)
ln(W)
BE (m)
rho 0.92
rho 0.99
rho −0.71
rho −0.88
rho 0.22
rho 0.018
rho −0.33
rho 0.11
rho 0.099
rho 0.072
rho −0.17
rho 0.11
rho 0.11
rho 0.036
rho −0.24
rho −0.071
rho 0.13
rho 0.018
rho −0.36
rho −0.29
rho −0.029
rho −0.072
rho 0.055
rho −0.11

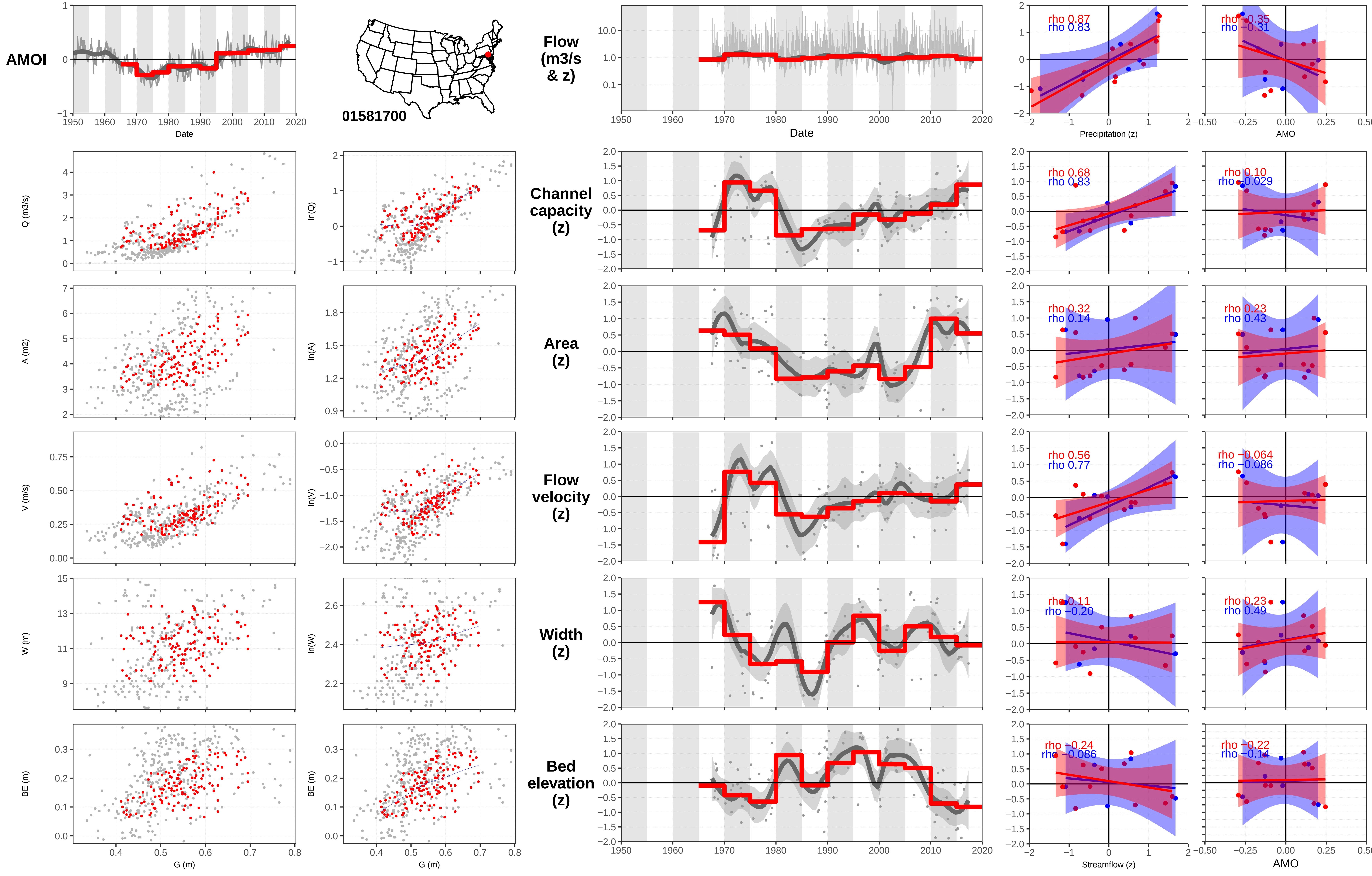

AMOI
01581700
Flow (m3/s & z)
Channel capacity (z)
Area (z)
Flow velocity (z)
Width (z)
Bed elevation (z)
Date
Precipitation (z)
AMO
Streamflow (z)
G (m)
Q (m3/s)
ln(Q)
A (m2)
ln(A)
V (m/s)
ln(V)
W (m)
ln(W)
BE (m)
rho 0.87
rho 0.83
rho −0.35
rho −0.31
rho 0.68
rho 0.83
rho 0.10
rho −0.029
rho 0.32
rho 0.14
rho 0.23
rho 0.43
rho 0.56
rho 0.77
rho −0.064
rho −0.086
rho 0.11
rho −0.20
rho 0.23
rho 0.49
rho −0.24
rho −0.086
rho −0.22
rho −0.14

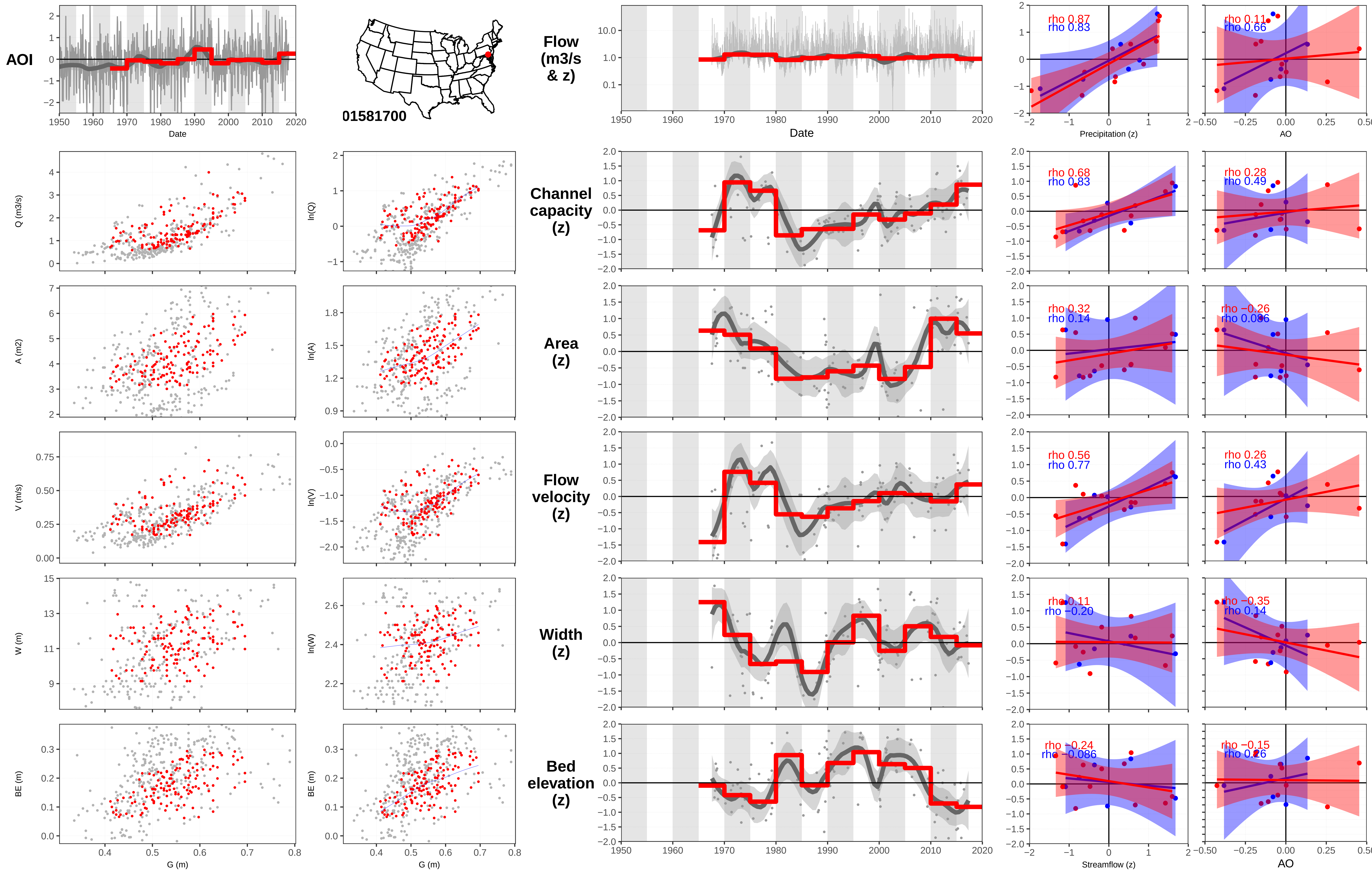

AOI
Date
01581700
Flow (m3/s & z)
Date
rho 0.87
rho 0.83
Precipitation (z)
rho 0.66
AO
Q (m3/s)
ln(Q)
Channel capacity (z)
rho 0.68
rho 0.83
rho 0.28
rho 0.49
A (m2)
ln(A)
Area (z)
rho 0.32
rho 0.14
rho −0.26
V (m/s)
ln(V)
Flow velocity (z)
rho 0.56
rho 0.77
rho 0.26
rho 0.43
W (m)
ln(W)
Width (z)
rho −0.20
rho −0.35
rho 0.14
BE (m)
BE (m)
Bed elevation (z)
rho −0.24
rho −0.086
rho −0.15
rho 0.26
G (m)
G (m)
Streamflow (z)
AO

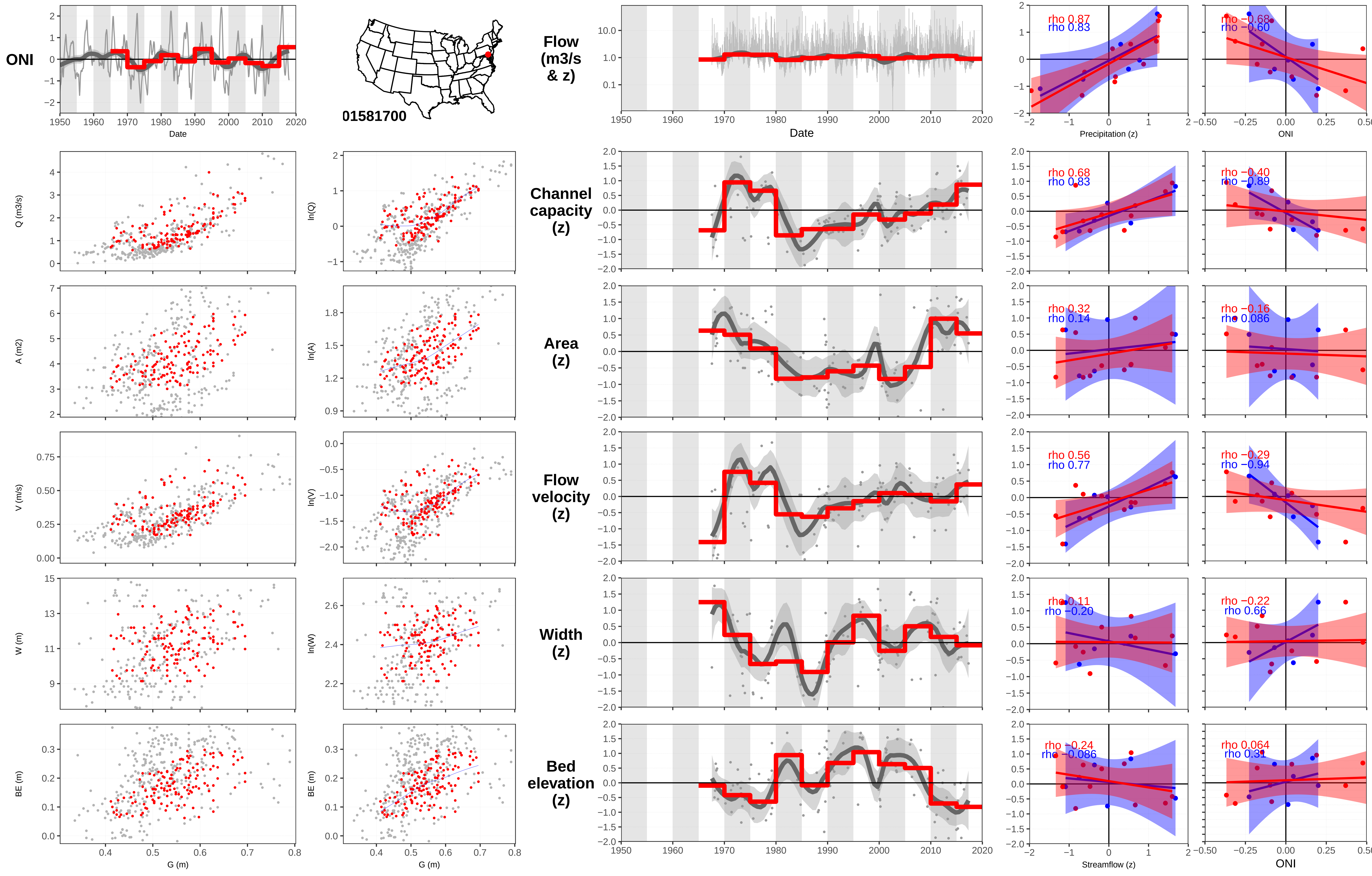
ONI
01581700
Flow (m3/s & z)
Channel capacity (z)
Area (z)
Flow velocity (z)
Width (z)
Bed elevation (z)
Date
Precipitation (z)
ONI
Streamflow (z)
G (m)
Q (m3/s)
ln(Q)
A (m2)
ln(A)
V (m/s)
ln(V)
W (m)
ln(W)
BE (m)
rho 0.87
rho 0.83
rho −0.68
rho −0.60
rho 0.68
rho 0.83
rho −0.40
rho −0.89
rho 0.32
rho 0.14
rho −0.16
rho 0.086
rho 0.56
rho 0.77
rho −0.29
rho −0.94
rho 0.11
rho −0.20
rho −0.22
rho 0.66
rho −0.24
rho −0.086
rho 0.064
rho 0.31

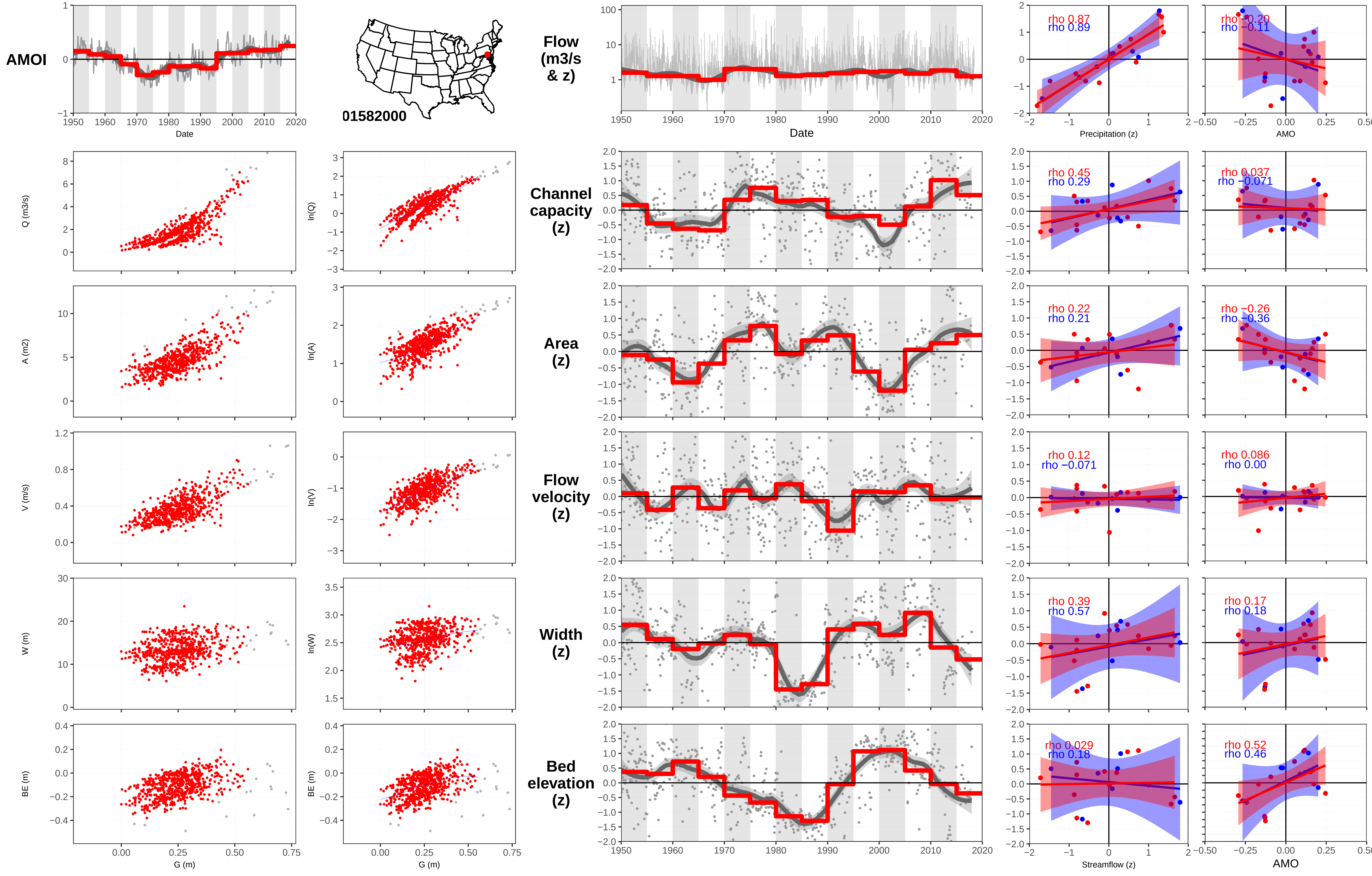

AMOI
01582000
Flow (m3/s & z)
Channel capacity (z)
Area (z)
Flow velocity (z)
Width (z)
Bed elevation (z)
Date
Precipitation (z)
AMO
Streamflow (z)
G (m)
Q (m3/s)
ln(Q)
A (m2)
ln(A)
V (m/s)
ln(V)
W (m)
ln(W)
BE (m)
rho 0.87
rho 0.89
rho −0.20
rho −0.11
rho 0.45
rho 0.29
rho 0.037
rho −0.071
rho 0.22
rho 0.21
rho −0.26
rho −0.36
rho 0.12
rho −0.071
rho 0.086
rho 0.00
rho 0.39
rho 0.57
rho 0.17
rho 0.18
rho 0.029
rho 0.18
rho 0.52
rho 0.46

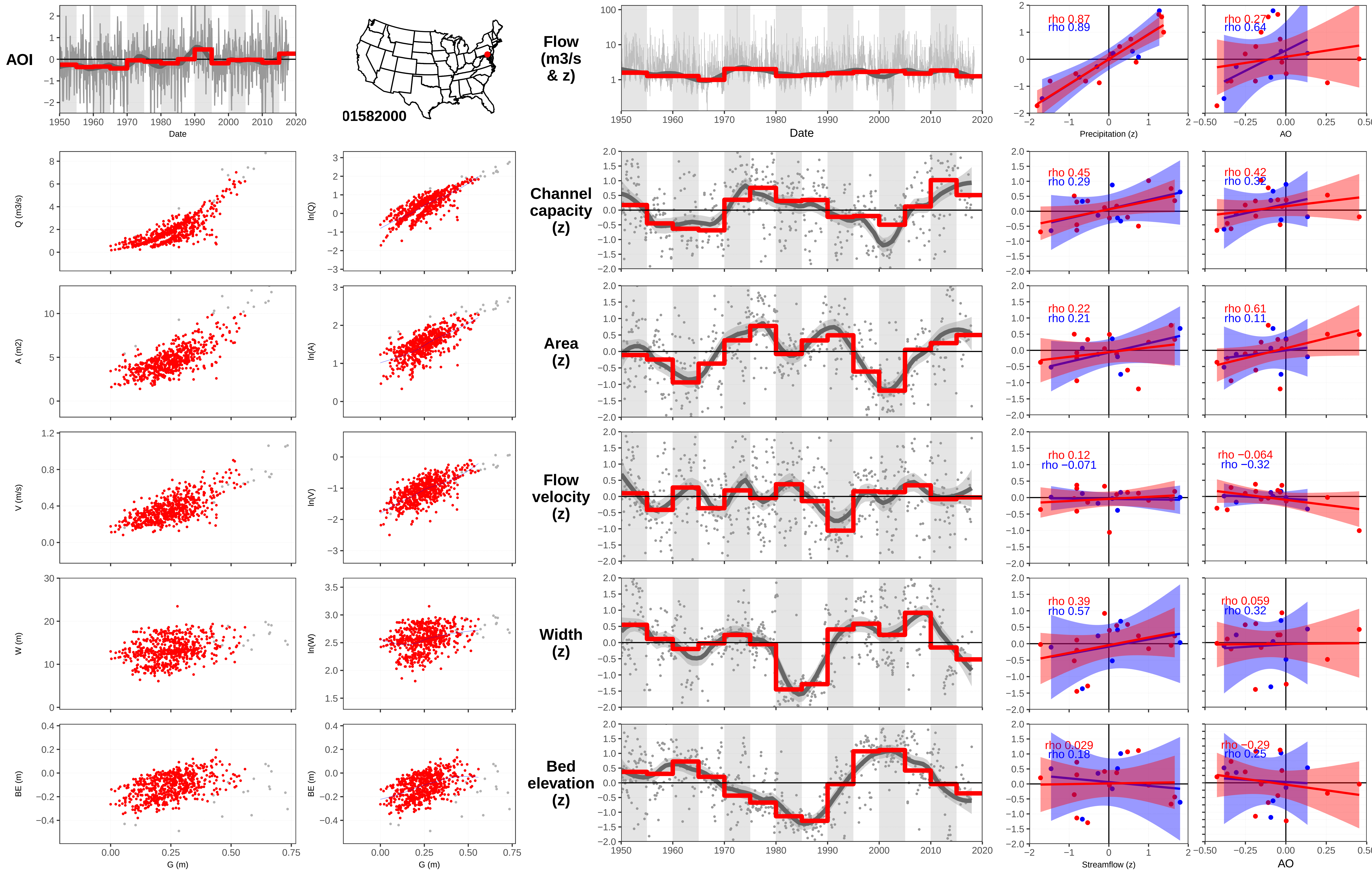

AOI
01582000
Flow (m3/s & z)
Channel capacity (z)
Area (z)
Flow velocity (z)
Width (z)
Bed elevation (z)
Date
Precipitation (z)
Streamflow (z)
AO
G (m)
Q (m3/s)
ln(Q)
A (m2)
ln(A)
V (m/s)
ln(V)
W (m)
ln(W)
BE (m)
rho 0.87
rho 0.89
rho 0.27
rho 0.64
rho 0.45
rho 0.29
rho 0.42
rho 0.32
rho 0.22
rho 0.21
rho 0.61
rho 0.11
rho 0.12
rho −0.071
rho −0.064
rho −0.32
rho 0.39
rho 0.57
rho 0.059
rho 0.32
rho 0.029
rho 0.18
rho −0.29
rho 0.25

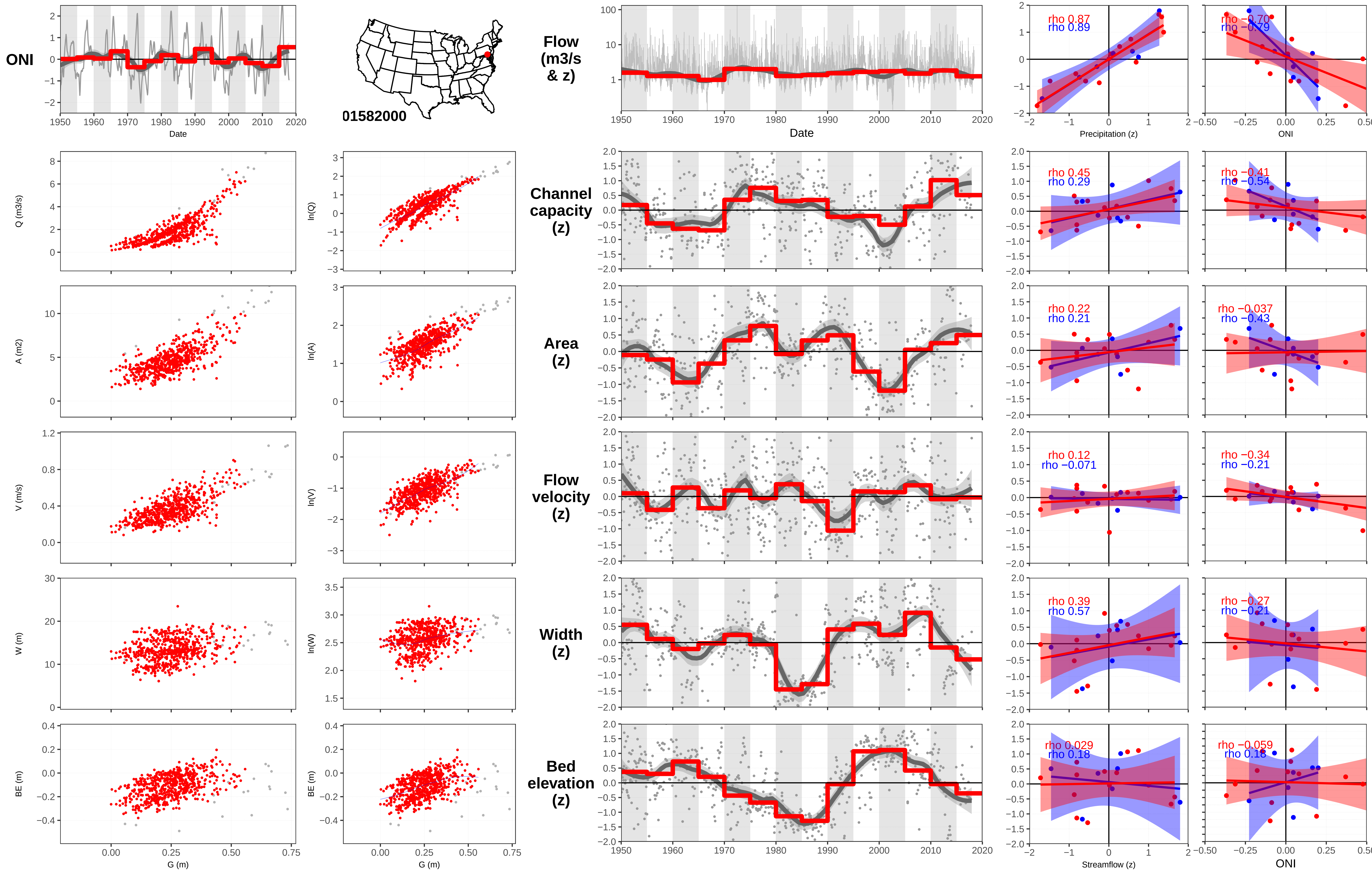

ONI
01582000
Flow (m3/s & z)
Channel capacity (z)
Area (z)
Flow velocity (z)
Width (z)
Bed elevation (z)
Date
Precipitation (z)
Streamflow (z)
ONI
G (m)
Q (m3/s)
ln(Q)
A (m2)
ln(A)
V (m/s)
ln(V)
W (m)
ln(W)
BE (m)
rho 0.87
rho 0.89
rho −0.70
rho −0.79
rho 0.45
rho 0.29
rho −0.41
rho −0.54
rho 0.22
rho 0.21
rho −0.037
rho −0.43
rho 0.12
rho −0.071
rho −0.34
rho −0.21
rho 0.39
rho 0.57
rho −0.27
rho −0.21
rho 0.029
rho 0.18
rho −0.059
rho 0.18

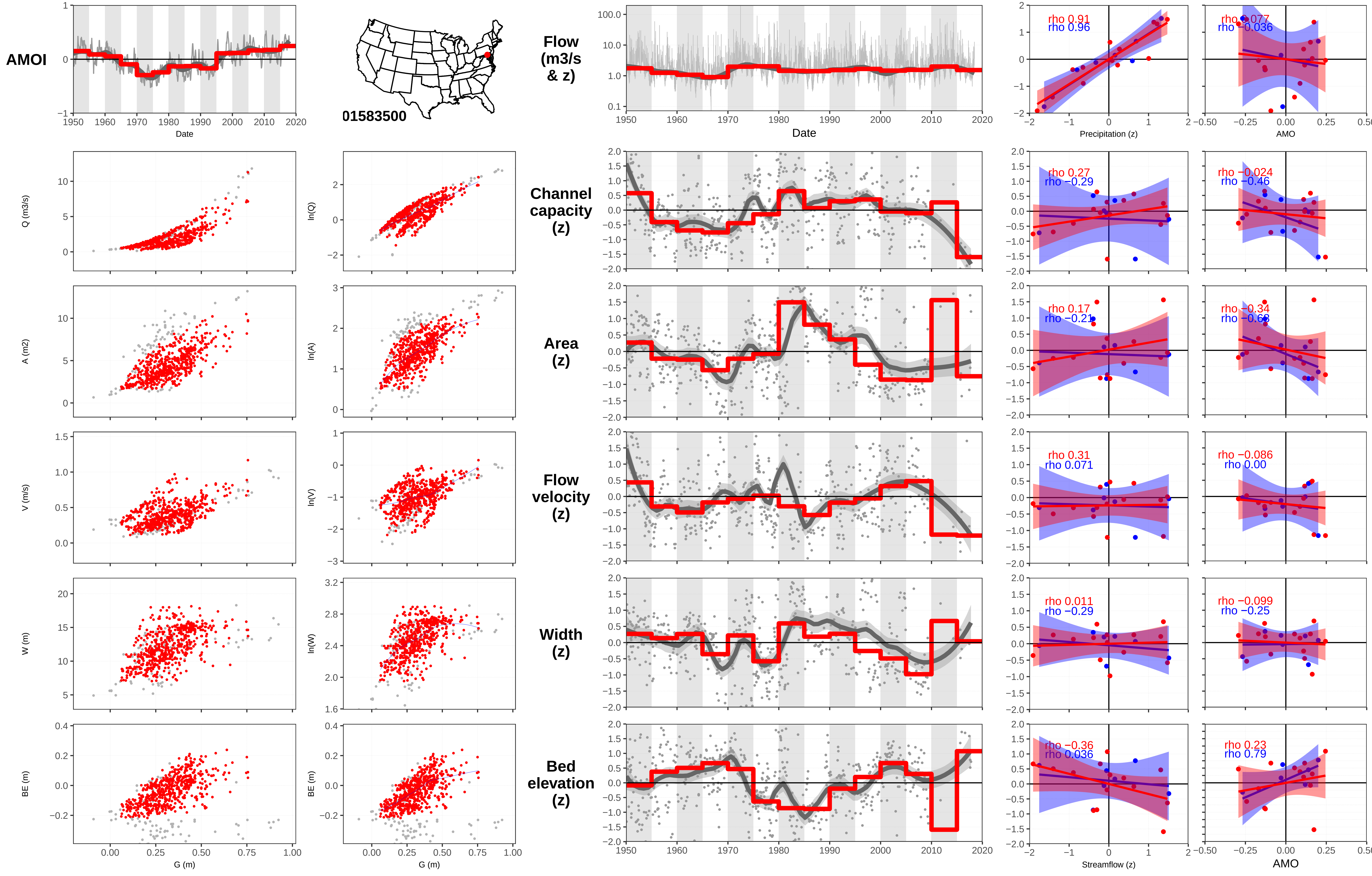

AMOI
01583500
Flow (m3/s & z)
Channel capacity (z)
Area (z)
Flow velocity (z)
Width (z)
Bed elevation (z)
Date
Precipitation (z)
AMO
Streamflow (z)
G (m)
Q (m3/s)
ln(Q)
A (m2)
ln(A)
V (m/s)
ln(V)
W (m)
ln(W)
BE (m)
rho 0.91
rho 0.96
rho 0.077
rho −0.036
rho 0.27
rho −0.29
rho −0.024
rho −0.46
rho 0.17
rho −0.21
rho −0.34
rho −0.68
rho 0.31
rho 0.071
rho −0.086
rho 0.00
rho 0.011
rho −0.29
rho −0.099
rho −0.25
rho −0.36
rho 0.036
rho 0.23
rho 0.79

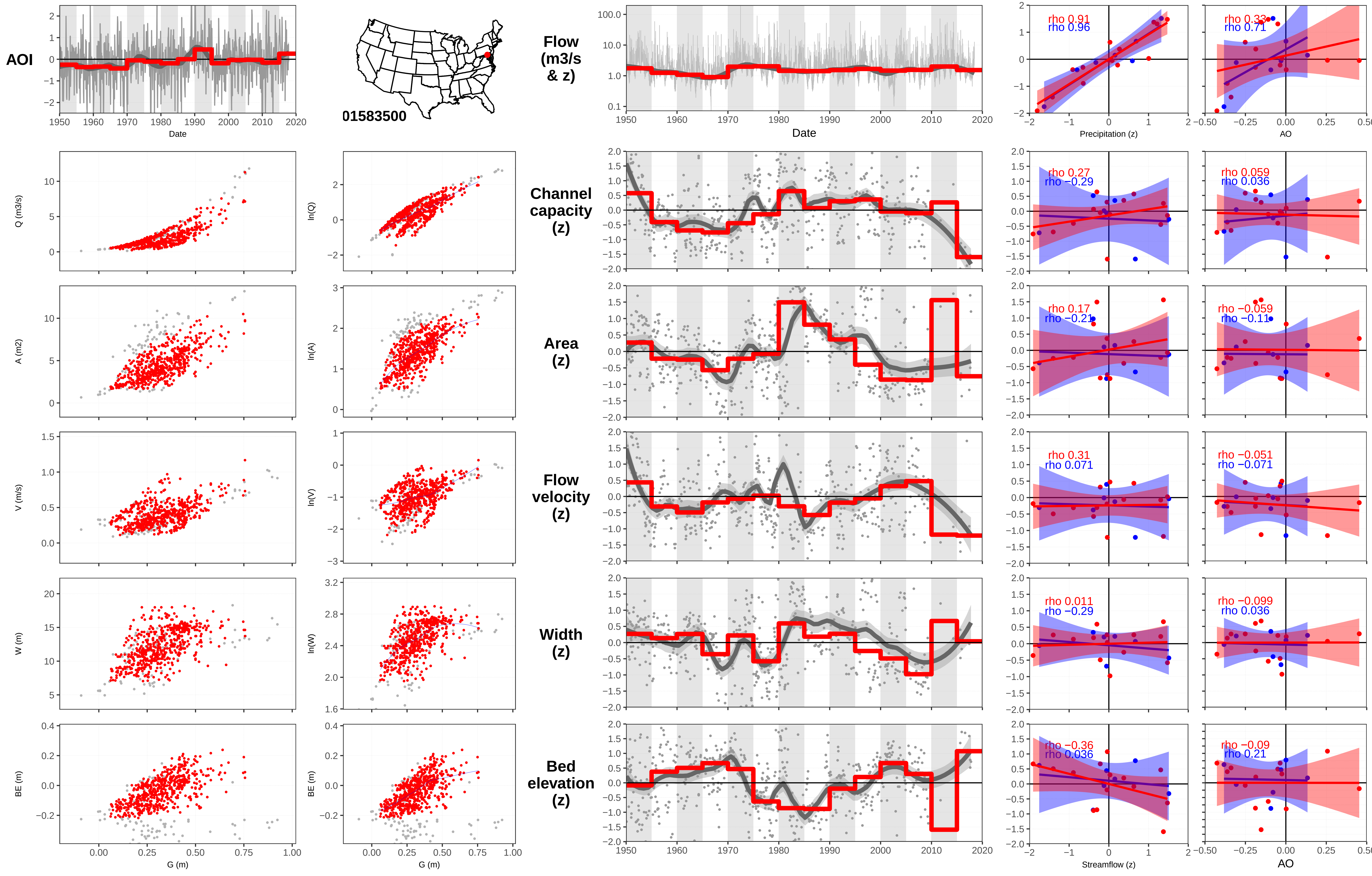

AOI
01583500
Flow (m3/s & z)
Channel capacity (z)
Area (z)
Flow velocity (z)
Width (z)
Bed elevation (z)
Date
Precipitation (z)
AO
Streamflow (z)
G (m)
Q (m3/s)
ln(Q)
A (m2)
ln(A)
V (m/s)
ln(V)
W (m)
ln(W)
BE (m)
rho 0.91
rho 0.96
rho 0.33
rho 0.71
rho 0.27
rho −0.29
rho 0.059
rho 0.036
rho 0.17
rho −0.21
rho −0.059
rho −0.11
rho 0.31
rho 0.071
rho −0.051
rho −0.071
rho 0.011
rho −0.29
rho −0.099
rho 0.036
rho −0.36
rho 0.036
rho −0.09
rho 0.21

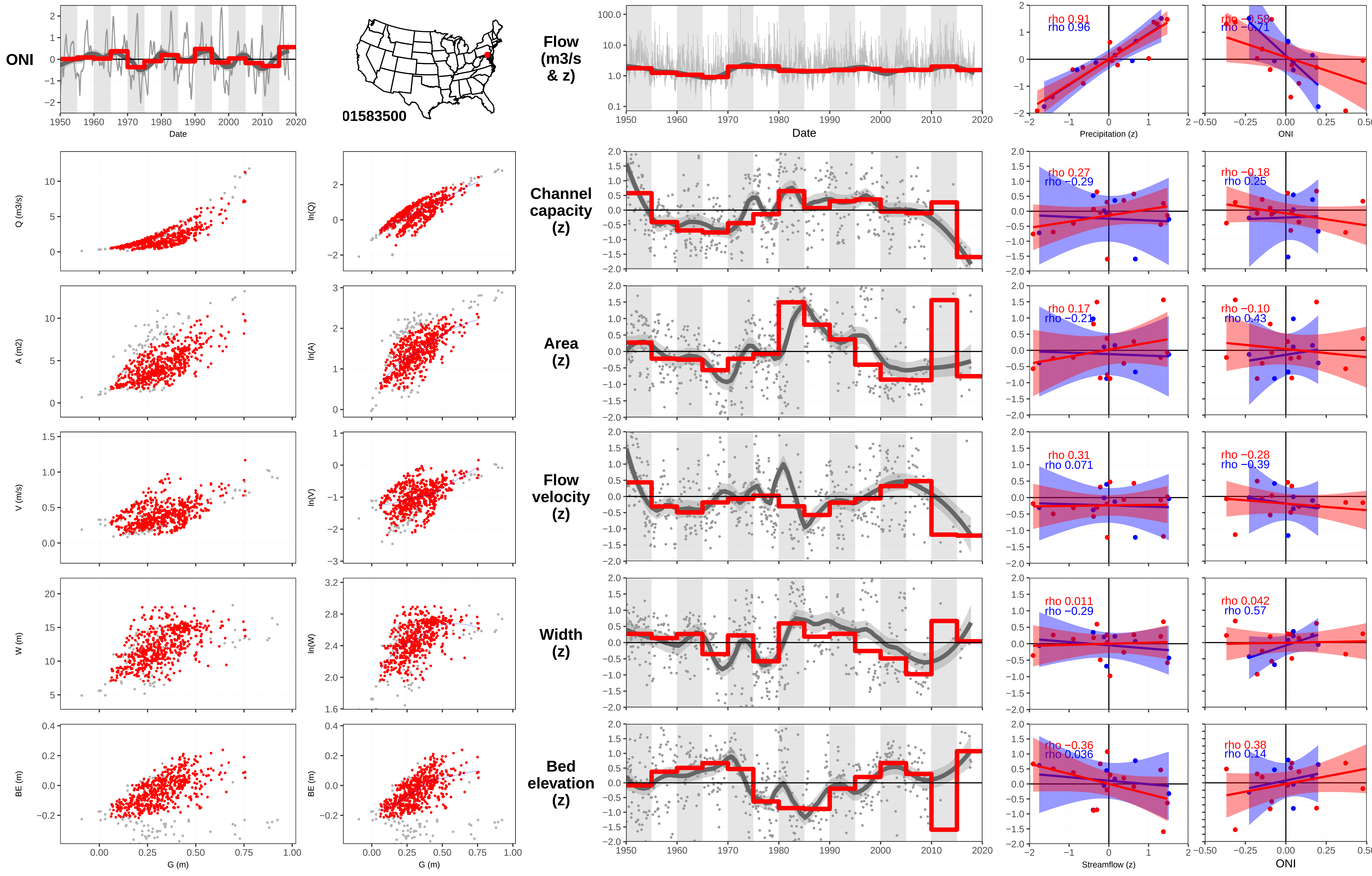

ONI
01583500
Flow (m3/s & z)
Channel capacity (z)
Area (z)
Flow velocity (z)
Width (z)
Bed elevation (z)
Date
Precipitation (z)
Streamflow (z)
ONI
G (m)
Q (m3/s)
ln(Q)
A (m2)
ln(A)
V (m/s)
ln(V)
W (m)
ln(W)
BE (m)
rho 0.91
rho 0.96
rho −0.58
rho −0.71
rho 0.27
rho −0.29
rho −0.18
rho 0.25
rho 0.17
rho −0.21
rho −0.10
rho 0.43
rho 0.31
rho 0.071
rho −0.28
rho −0.39
rho 0.011
rho −0.29
rho 0.042
rho 0.57
rho −0.36
rho 0.036
rho 0.38
rho 0.14

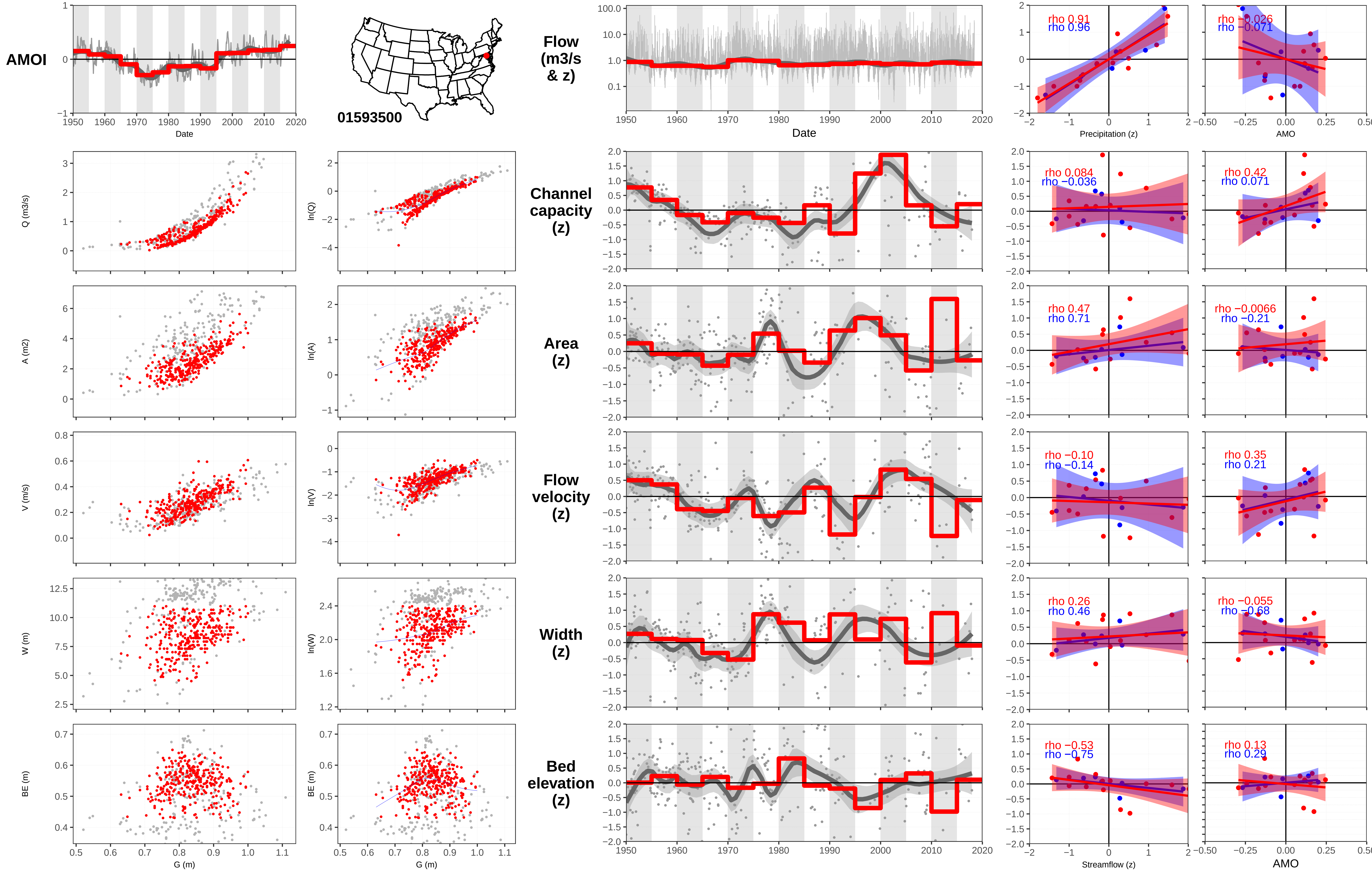
AMOI
01593500
Flow (m3/s & z)
Channel capacity (z)
Area (z)
Flow velocity (z)
Width (z)
Bed elevation (z)
Date
Precipitation (z)
AMO
Streamflow (z)
G (m)
Q (m3/s)
ln(Q)
A (m2)
ln(A)
V (m/s)
ln(V)
W (m)
ln(W)
BE (m)
rho 0.91
rho 0.96
rho −0.026
rho −0.071
rho 0.084
rho −0.036
rho 0.42
rho 0.071
rho 0.47
rho 0.71
rho −0.0066
rho −0.21
rho −0.10
rho −0.14
rho 0.35
rho 0.21
rho 0.26
rho 0.46
rho −0.055
rho −0.68
rho −0.53
rho −0.75
rho 0.13
rho 0.29

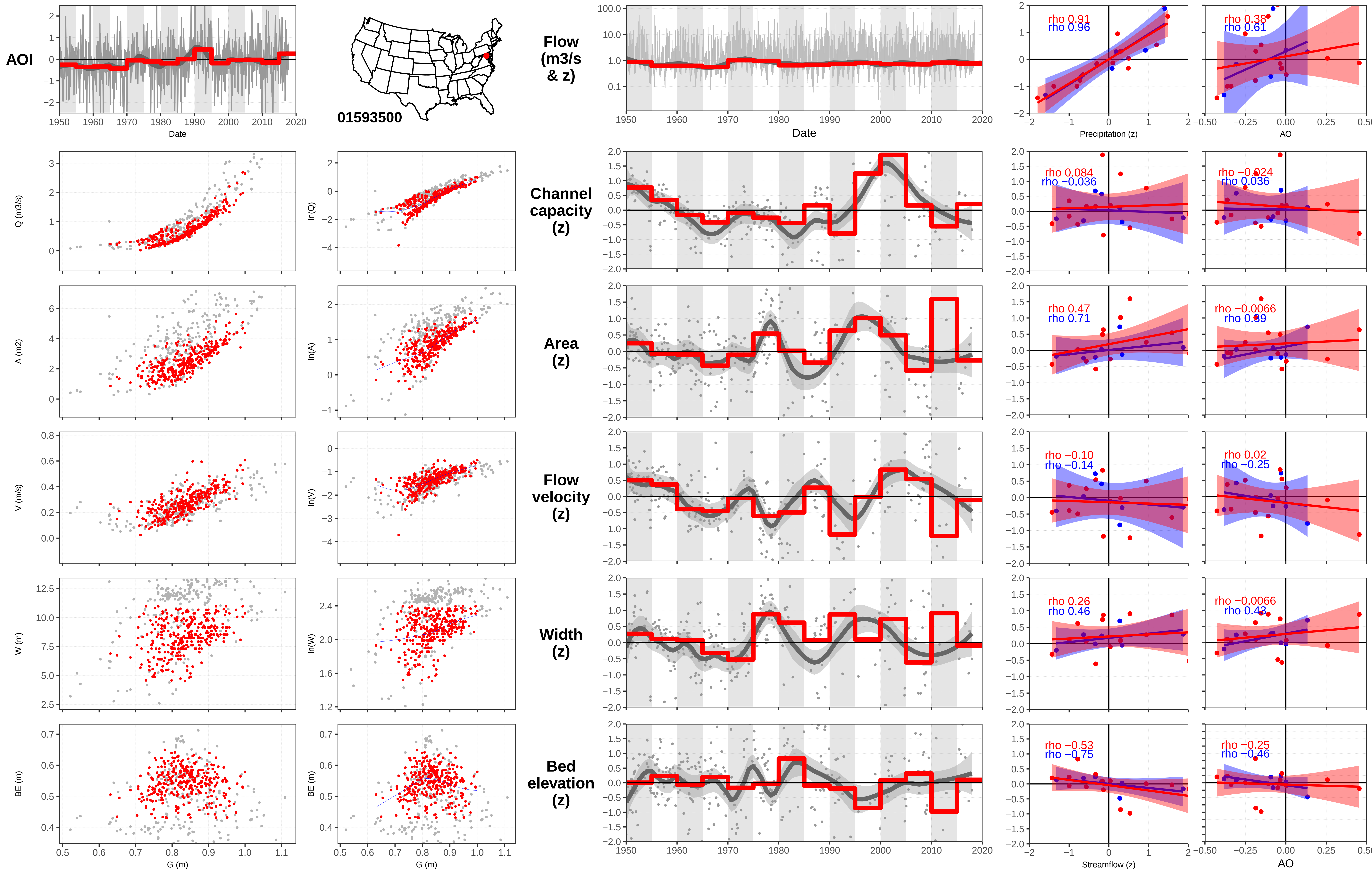

AOI
01593500
Date
Flow (m3/s & z)
Channel capacity (z)
Area (z)
Flow velocity (z)
Width (z)
Bed elevation (z)
Q (m3/s)
ln(Q)
A (m2)
ln(A)
V (m/s)
ln(V)
W (m)
ln(W)
BE (m)
G (m)
Precipitation (z)
Streamflow (z)
AO
rho 0.91
rho 0.96
rho 0.38
rho 0.61
rho 0.084
rho −0.036
rho −0.024
rho 0.036
rho 0.47
rho 0.71
rho −0.0066
rho 0.39
rho −0.10
rho −0.14
rho 0.02
rho −0.25
rho 0.26
rho 0.46
rho −0.0066
rho 0.43
rho −0.53
rho −0.75
rho −0.25
rho −0.46

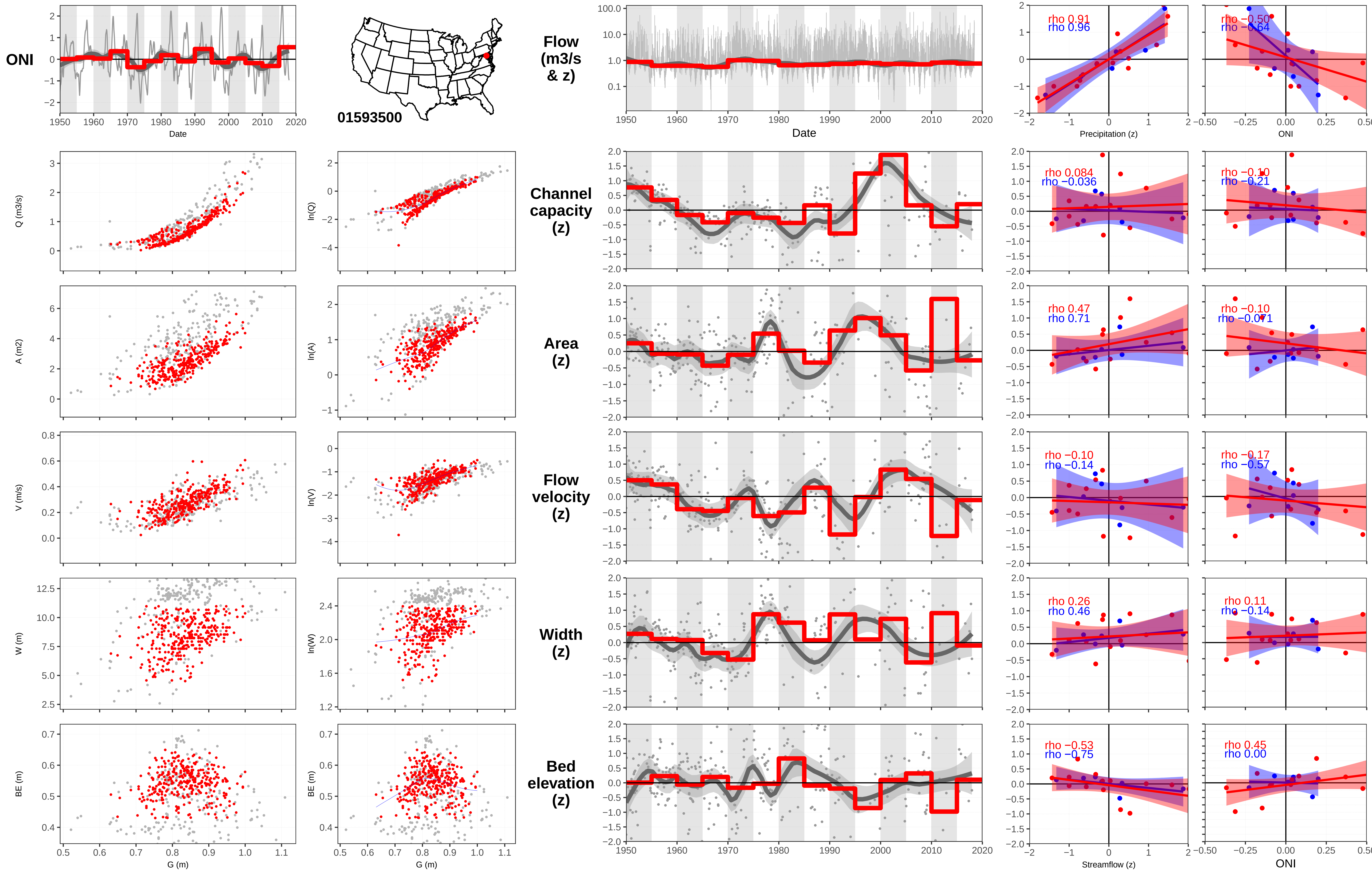

ONI
01593500
Flow (m3/s & z)
Channel capacity (z)
Area (z)
Flow velocity (z)
Width (z)
Bed elevation (z)
Date
Precipitation (z)
ONI
Streamflow (z)
G (m)
Q (m3/s)
ln(Q)
A (m2)
ln(A)
V (m/s)
ln(V)
W (m)
ln(W)
BE (m)
rho 0.91
rho 0.96
rho −0.50
rho −0.64
rho 0.084
rho −0.036
rho −0.10
rho −0.21
rho 0.47
rho 0.71
rho −0.10
rho −0.071
rho −0.10
rho −0.14
rho −0.17
rho −0.57
rho 0.26
rho 0.46
rho 0.11
rho −0.14
rho −0.53
rho −0.75
rho 0.45
rho 0.00

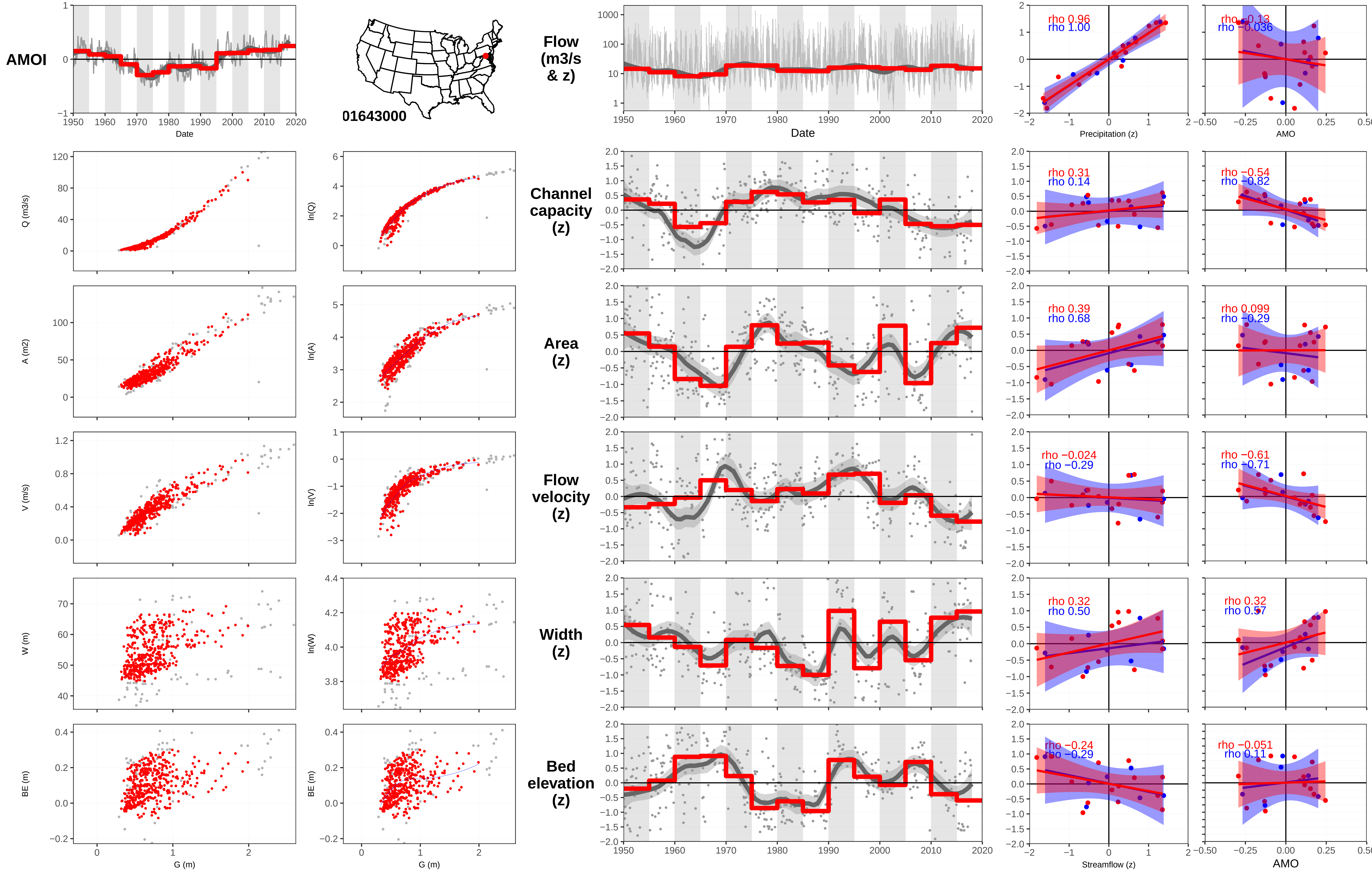

AMOI
01643000
Flow (m3/s & z)
Channel capacity (z)
Area (z)
Flow velocity (z)
Width (z)
Bed elevation (z)
Date
Precipitation (z)
AMO
Streamflow (z)
G (m)
Q (m3/s)
ln(Q)
A (m2)
ln(A)
V (m/s)
ln(V)
W (m)
ln(W)
BE (m)
rho 0.96
rho 1.00
rho −0.13
rho −0.036
rho 0.31
rho 0.14
rho −0.54
rho −0.82
rho 0.39
rho 0.68
rho 0.099
rho −0.29
rho −0.024
rho −0.29
rho −0.61
rho −0.71
rho 0.32
rho 0.50
rho 0.32
rho 0.57
rho −0.24
rho −0.29
rho −0.051
rho 0.11

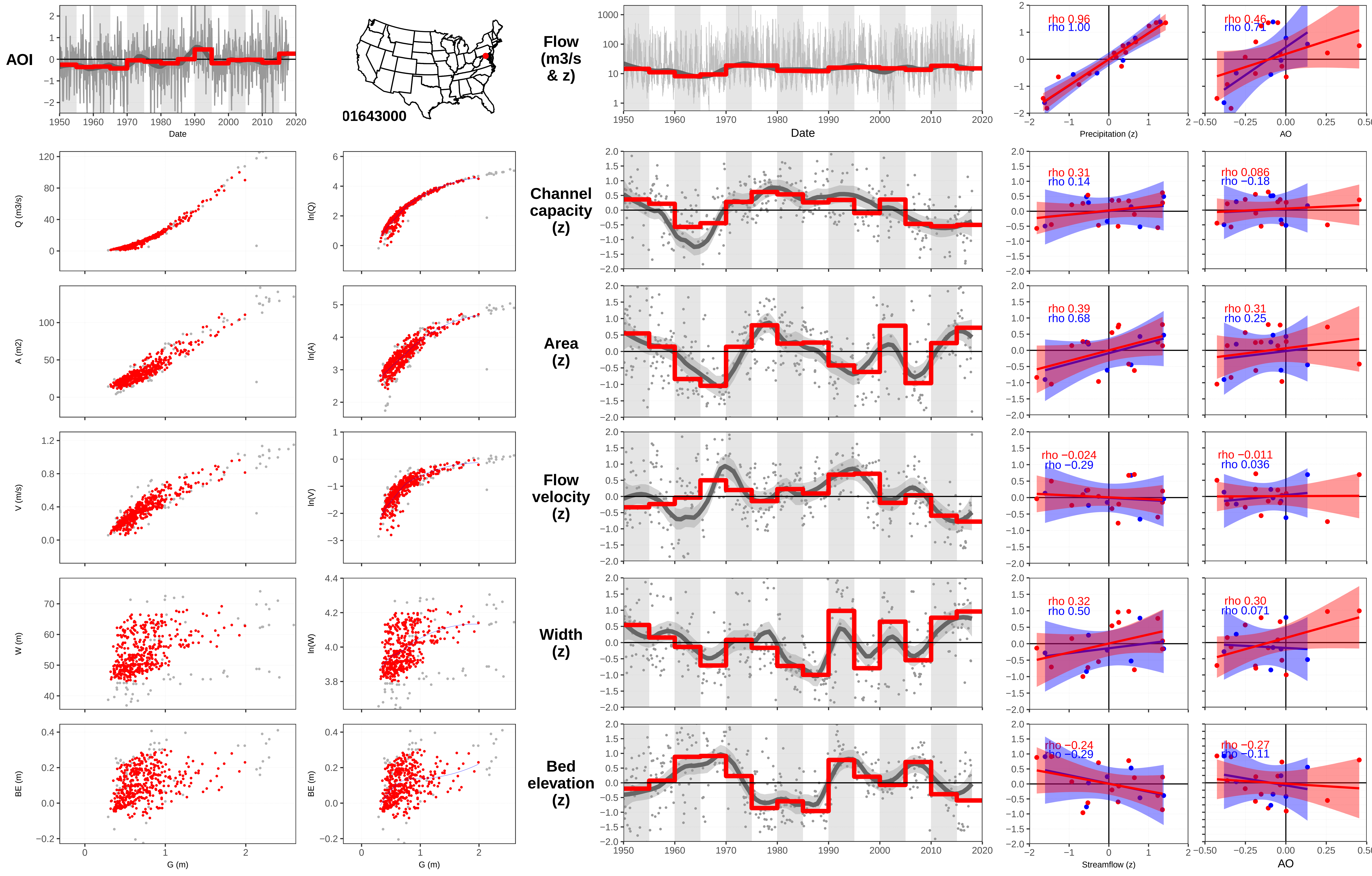

AOI
Date
01643000
Flow (m3/s & z)
Date
rho 0.96
rho 1.00
Precipitation (z)
rho 0.46
rho 0.71
AO
Q (m3/s)
ln(Q)
Channel capacity (z)
rho 0.31
rho 0.14
rho 0.086
rho −0.18
A (m2)
ln(A)
Area (z)
rho 0.39
rho 0.68
rho 0.31
rho 0.25
V (m/s)
ln(V)
Flow velocity (z)
rho −0.024
rho −0.29
rho −0.011
rho 0.036
W (m)
ln(W)
Width (z)
rho 0.32
rho 0.50
rho 0.30
rho 0.071
BE (m)
BE (m)
Bed elevation (z)
rho −0.24
rho −0.29
rho −0.27
rho −0.11
G (m)
G (m)
Streamflow (z)
AO

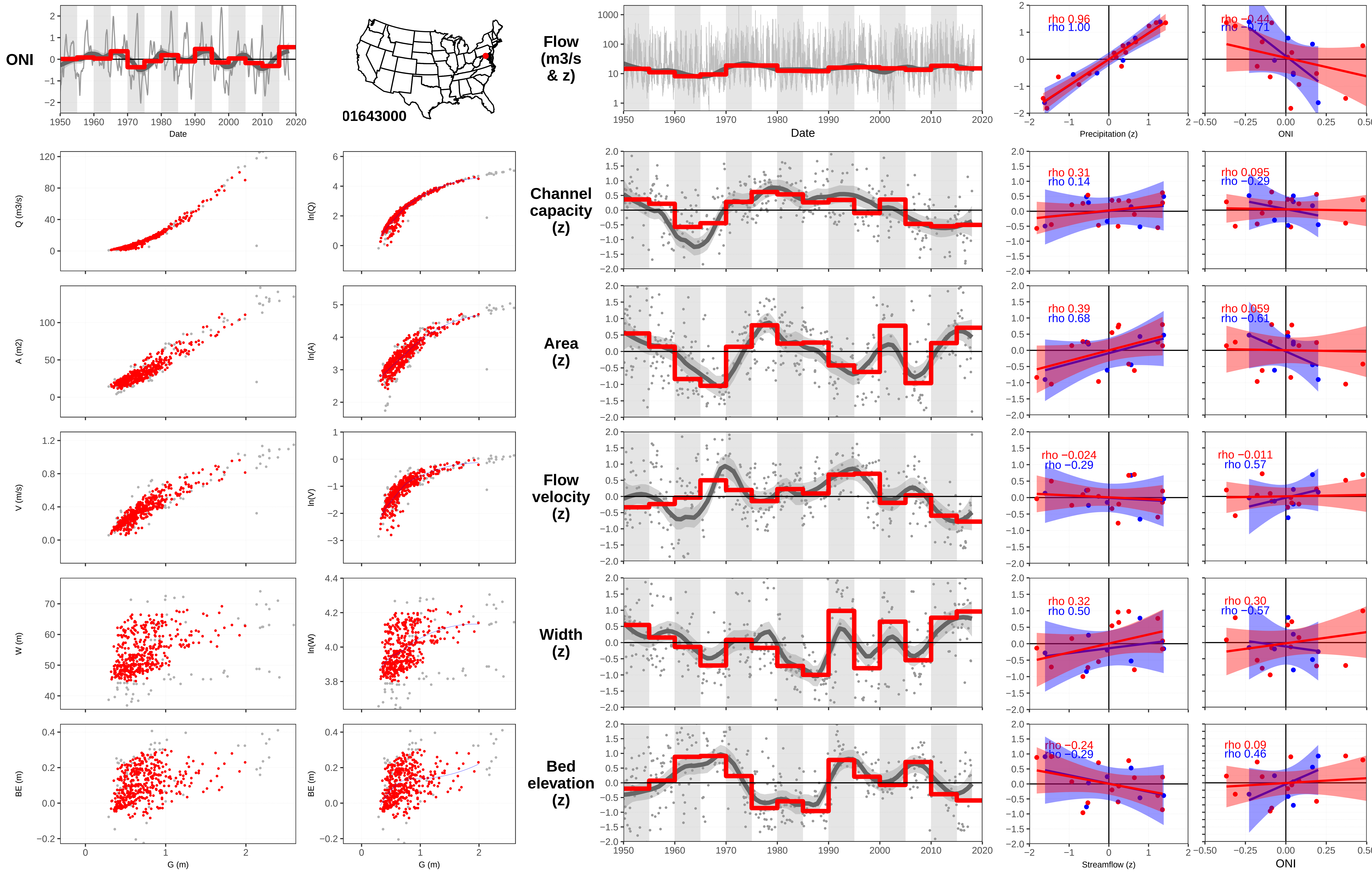

ONI
01643000
Flow (m3/s & z)
Channel capacity (z)
Area (z)
Flow velocity (z)
Width (z)
Bed elevation (z)
Date
Precipitation (z)
ONI
Streamflow (z)
G (m)
Q (m3/s)
ln(Q)
A (m2)
ln(A)
V (m/s)
ln(V)
W (m)
ln(W)
BE (m)
rho 0.96
rho 1.00
rho −0.44
rho −0.71
rho 0.31
rho 0.14
rho 0.095
rho −0.29
rho 0.39
rho 0.68
rho 0.059
rho −0.61
rho −0.024
rho −0.29
rho −0.011
rho 0.57
rho 0.32
rho 0.50
rho 0.30
rho −0.57
rho −0.24
rho −0.29
rho 0.09
rho 0.46

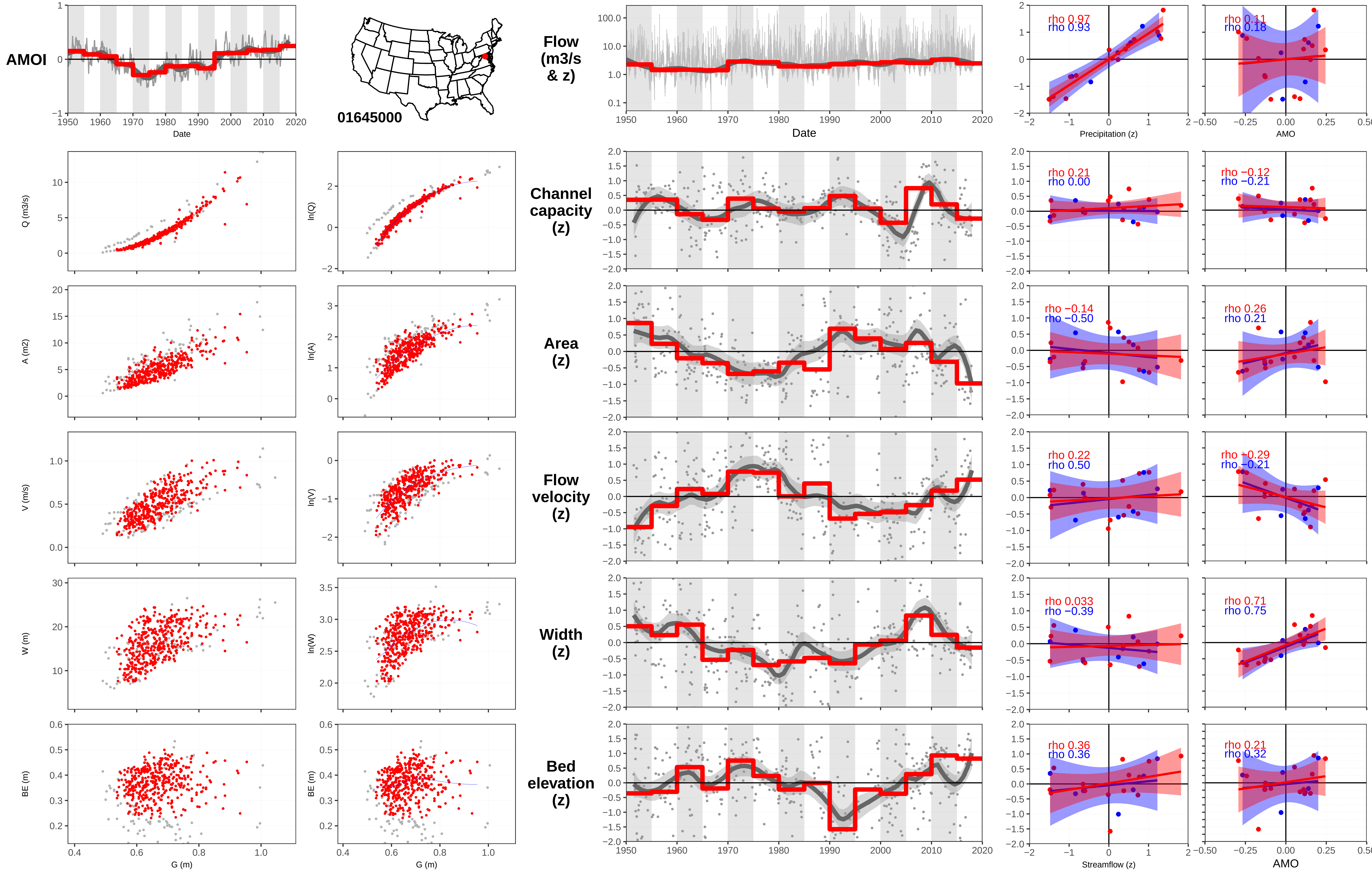
AMOI
01645000
Flow (m3/s & z)
Channel capacity (z)
Area (z)
Flow velocity (z)
Width (z)
Bed elevation (z)
Date
Precipitation (z)
Streamflow (z)
AMO
Q (m3/s)
ln(Q)
A (m2)
ln(A)
V (m/s)
ln(V)
W (m)
ln(W)
BE (m)
G (m)
rho 0.97
rho 0.93
rho 0.11
rho 0.18
rho 0.21
rho 0.00
rho −0.12
rho −0.21
rho −0.14
rho −0.50
rho 0.26
rho 0.21
rho 0.22
rho 0.50
rho −0.29
rho −0.21
rho 0.033
rho −0.39
rho 0.71
rho 0.75
rho 0.36
rho 0.36
rho 0.21
rho 0.32

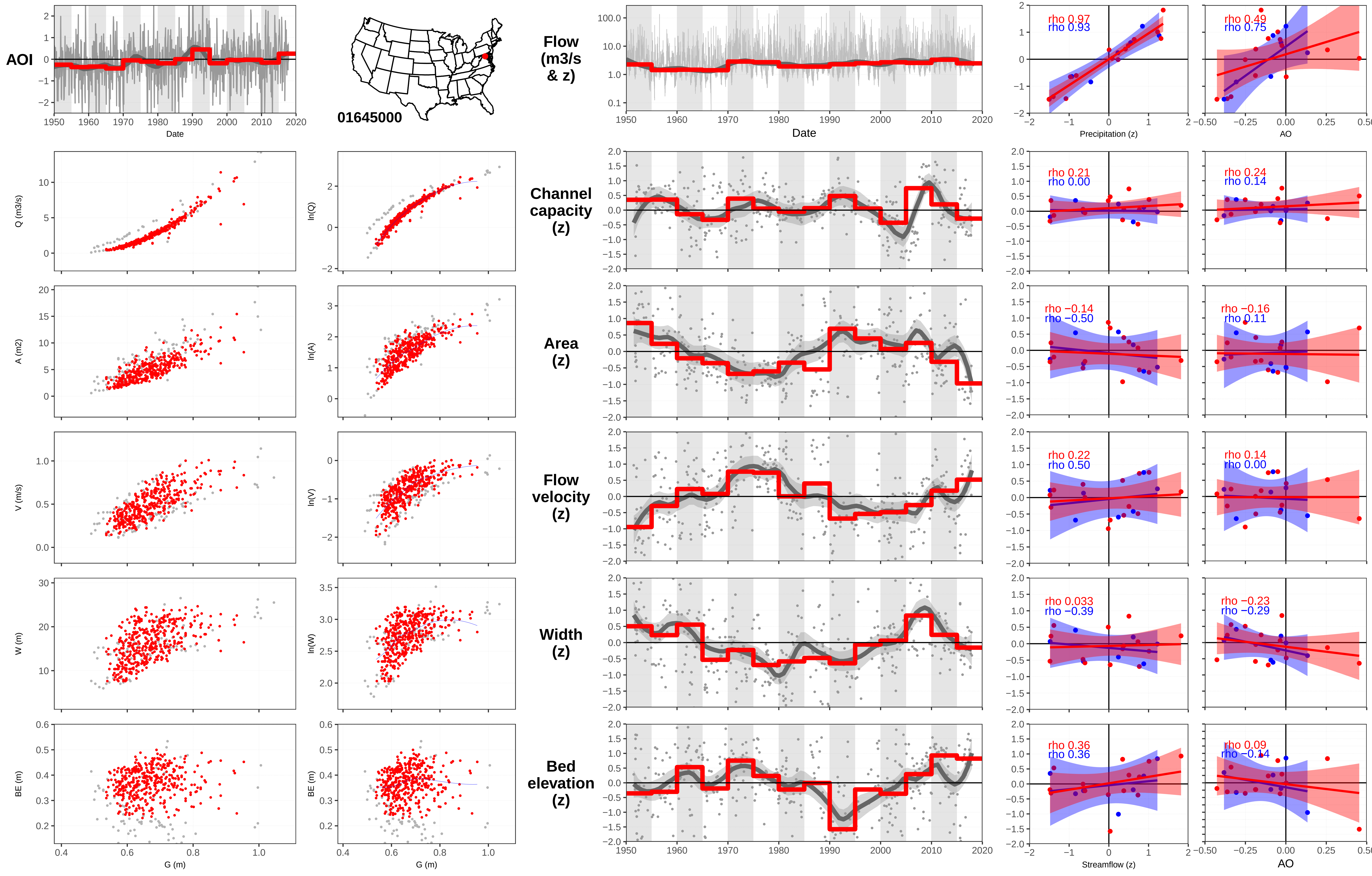

AOI
01645000
Flow (m3/s & z)
Channel capacity (z)
Area (z)
Flow velocity (z)
Width (z)
Bed elevation (z)
Date
Precipitation (z)
Streamflow (z)
AO
Q (m3/s)
ln(Q)
A (m2)
ln(A)
V (m/s)
ln(V)
W (m)
ln(W)
BE (m)
G (m)
rho 0.97
rho 0.93
rho 0.49
rho 0.75
rho 0.21
rho 0.00
rho 0.24
rho 0.14
rho −0.14
rho −0.50
rho −0.16
rho 0.11
rho 0.22
rho 0.50
rho 0.14
rho 0.00
rho 0.033
rho −0.39
rho −0.23
rho −0.29
rho 0.36
rho 0.36
rho 0.09
rho −0.14

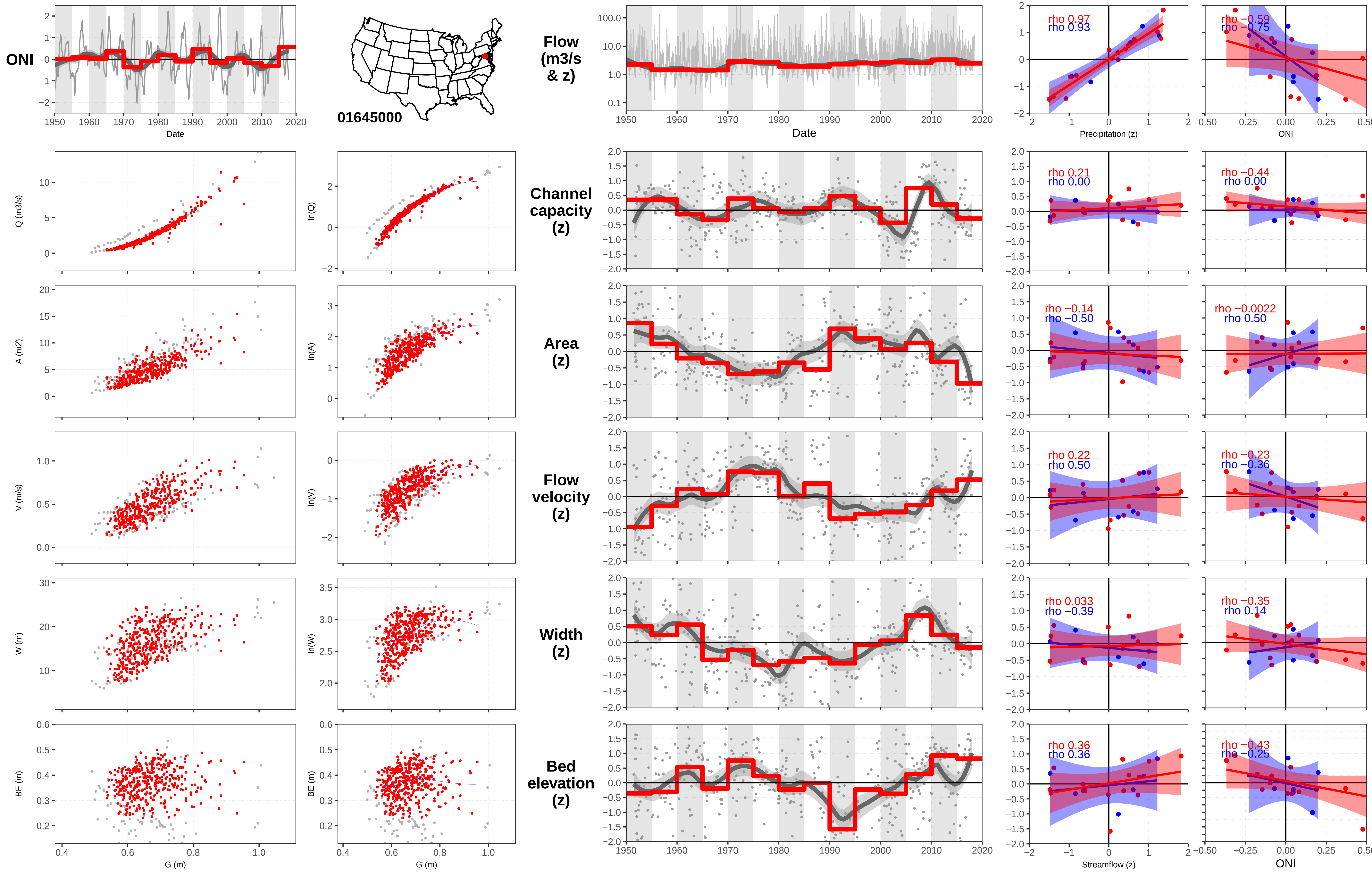
ONI
01645000
Flow (m3/s & z)
Channel capacity (z)
Area (z)
Flow velocity (z)
Width (z)
Bed elevation (z)
Date
Precipitation (z)
Streamflow (z)
ONI
G (m)
Q (m3/s)
ln(Q)
A (m2)
ln(A)
V (m/s)
ln(V)
W (m)
ln(W)
BE (m)
rho 0.97
rho 0.93
rho −0.59
rho −0.75
rho 0.21
rho 0.00
rho −0.44
rho 0.00
rho −0.14
rho −0.50
rho −0.0022
rho 0.50
rho 0.22
rho 0.50
rho −0.23
rho −0.36
rho 0.033
rho −0.39
rho −0.35
rho 0.14
rho 0.36
rho 0.36
rho −0.43
rho −0.25

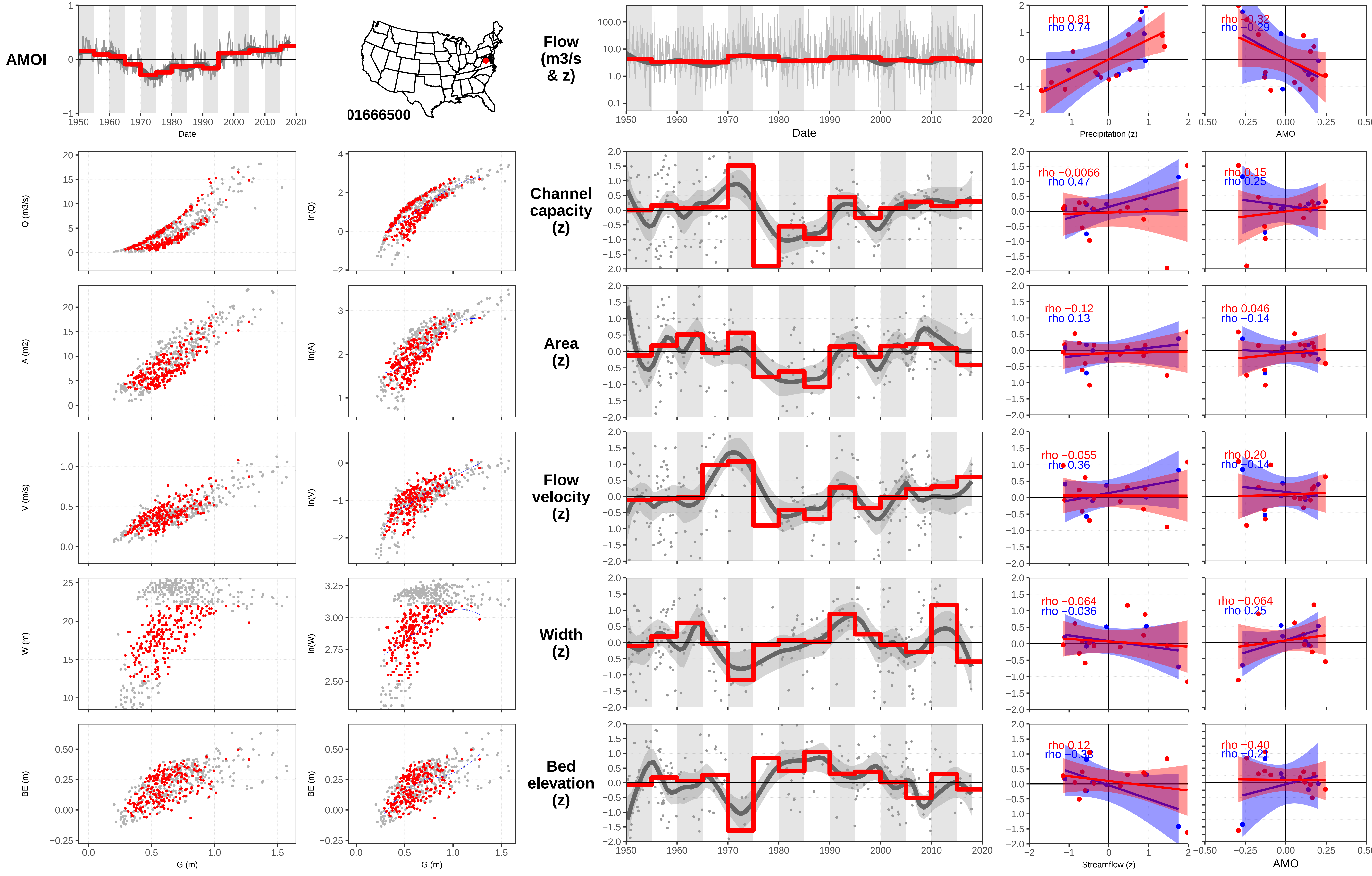
AMOI
Date
01666500
Flow (m3/s & z)
Date
rho 0.81
rho 0.74
Precipitation (z)
rho −0.32
rho −0.29
AMO
Q (m3/s)
ln(Q)
Channel capacity (z)
rho −0.0066
rho 0.47
rho 0.15
rho 0.25
A (m2)
ln(A)
Area (z)
rho −0.12
rho 0.13
rho 0.046
rho −0.14
V (m/s)
ln(V)
Flow velocity (z)
rho −0.055
rho 0.36
rho 0.20
rho −0.14
W (m)
ln(W)
Width (z)
rho −0.064
rho −0.036
rho −0.064
rho 0.25
BE (m)
BE (m)
Bed elevation (z)
rho 0.12
rho −0.38
rho −0.40
rho −0.21
G (m)
G (m)
Streamflow (z)
AMO

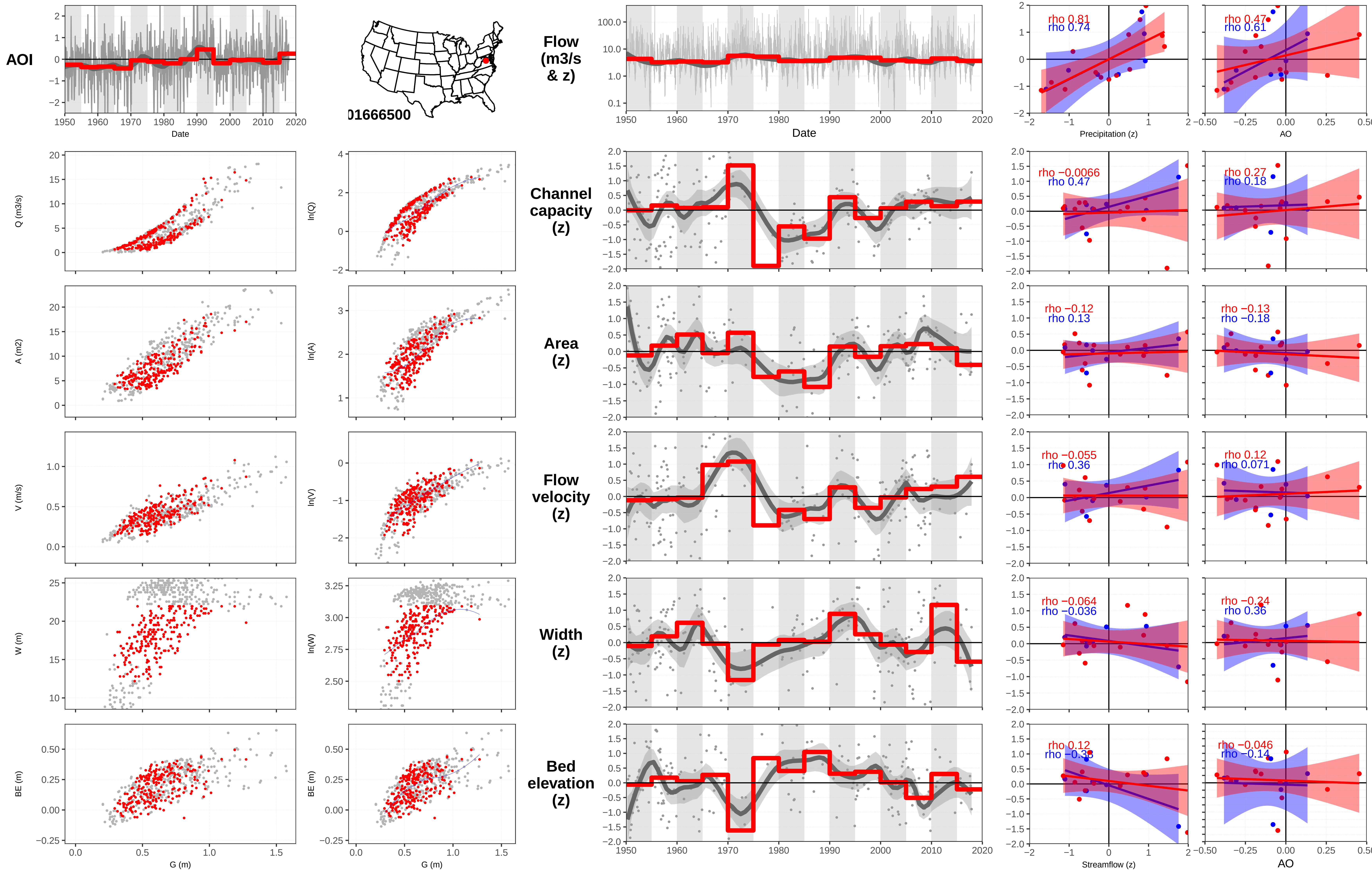
AOI
01666500
Flow (m3/s & z)
Channel capacity (z)
Area (z)
Flow velocity (z)
Width (z)
Bed elevation (z)
Date
G (m)
Q (m3/s)
ln(Q)
A (m2)
ln(A)
V (m/s)
ln(V)
W (m)
ln(W)
BE (m)
Precipitation (z)
Streamflow (z)
AO
rho 0.81
rho 0.74
rho 0.47
rho 0.61
rho −0.0066
rho 0.47
rho 0.27
rho 0.18
rho −0.12
rho 0.13
rho −0.13
rho −0.18
rho −0.055
rho 0.36
rho 0.12
rho 0.071
rho −0.064
rho −0.036
rho −0.24
rho 0.36
rho 0.12
rho −0.38
rho −0.046
rho −0.14

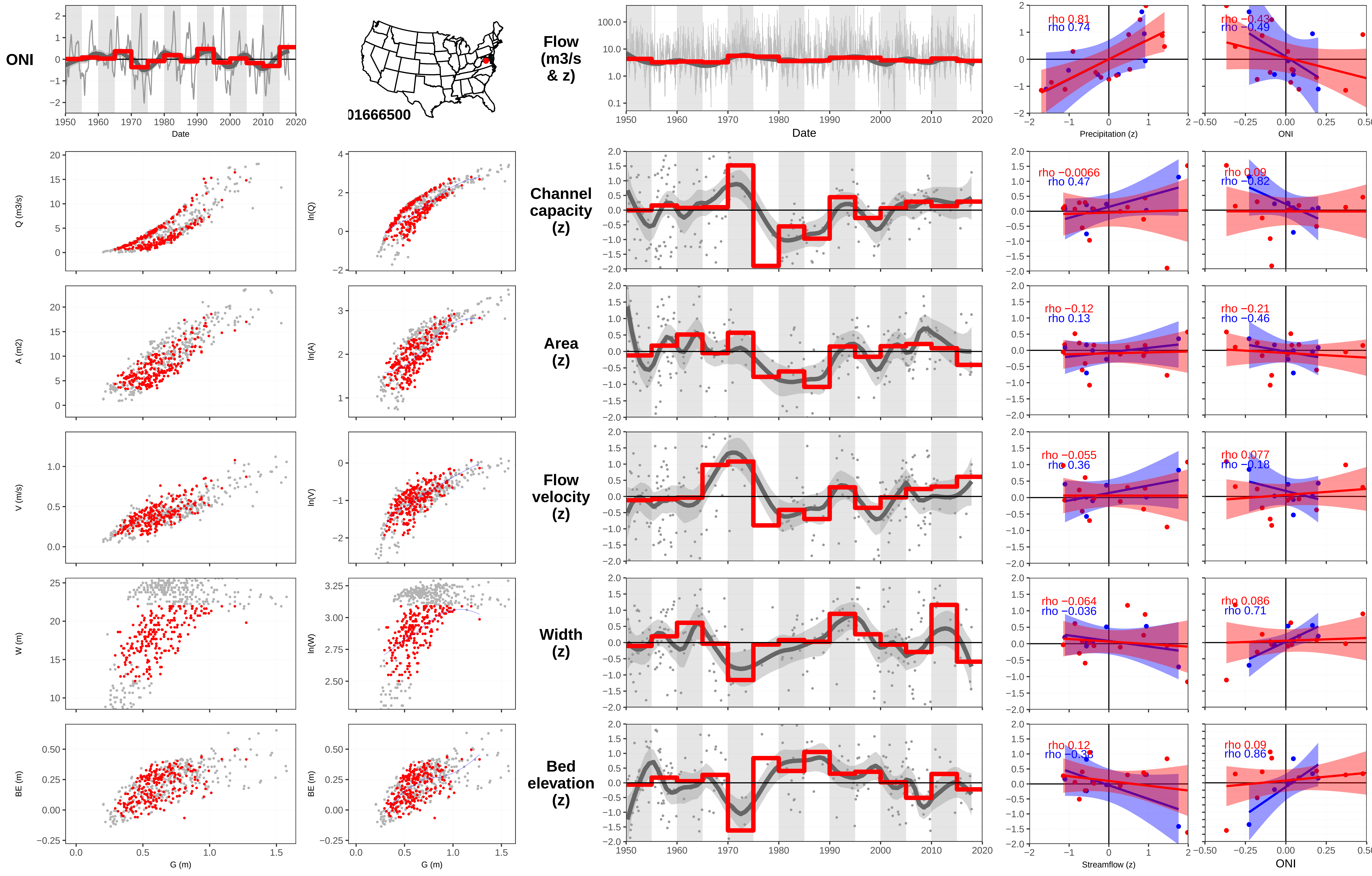
ONI
01666500
Flow (m3/s & z)
Channel capacity (z)
Area (z)
Flow velocity (z)
Width (z)
Bed elevation (z)
Date
Precipitation (z)
ONI
Streamflow (z)
G (m)
Q (m3/s)
ln(Q)
A (m2)
ln(A)
V (m/s)
ln(V)
W (m)
ln(W)
BE (m)
rho 0.81
rho 0.74
rho −0.43
rho −0.49
rho −0.0066
rho 0.47
rho 0.09
rho −0.82
rho −0.12
rho 0.13
rho −0.21
rho −0.46
rho −0.055
rho 0.36
rho 0.077
rho −0.18
rho −0.064
rho −0.036
rho 0.086
rho 0.71
rho 0.12
rho −0.38
rho 0.09
rho 0.86

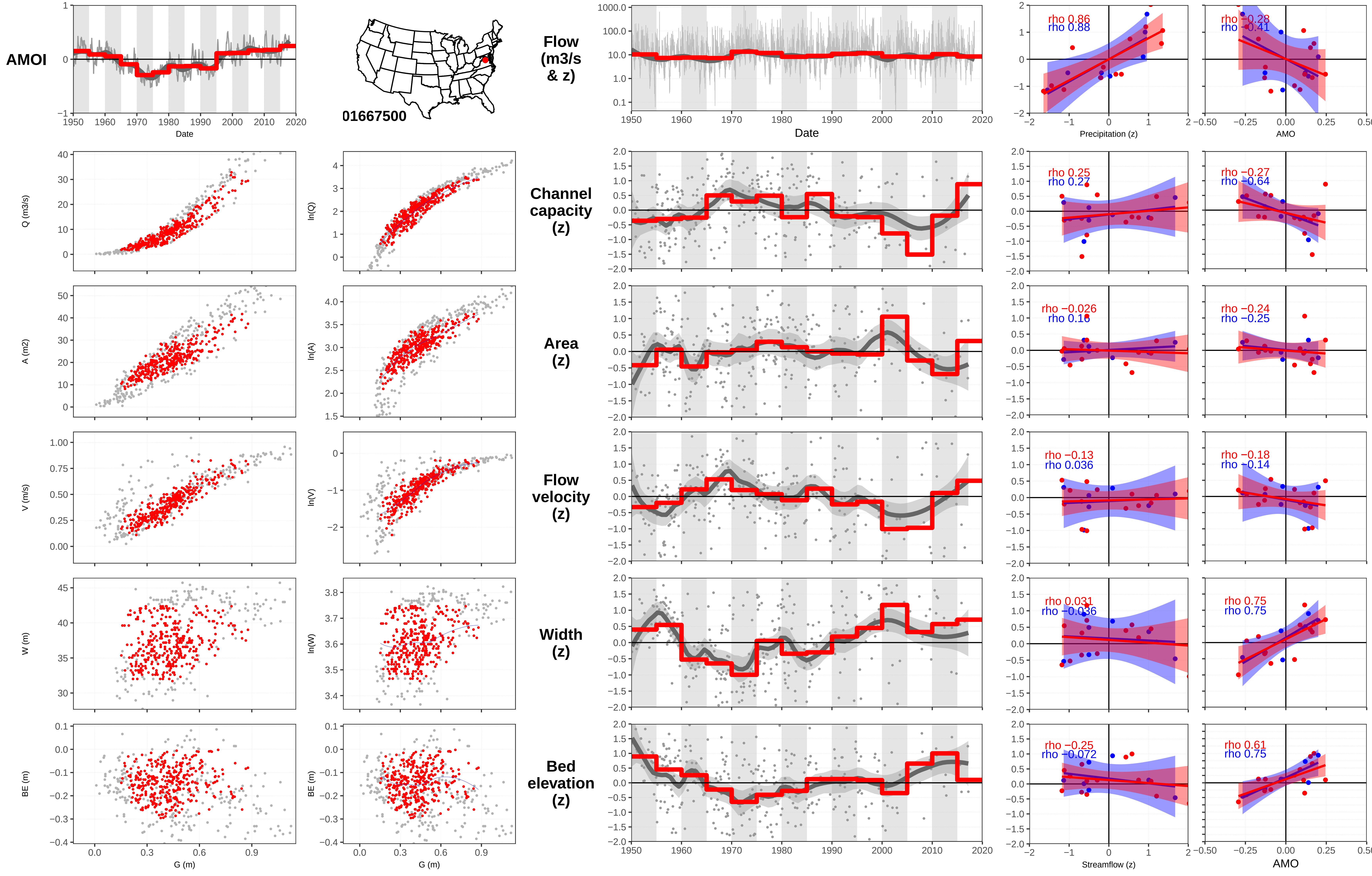
AMOI
01667500
Flow (m3/s & z)
Channel capacity (z)
Area (z)
Flow velocity (z)
Width (z)
Bed elevation (z)
Date
Precipitation (z)
AMO
Streamflow (z)
G (m)
Q (m3/s)
ln(Q)
A (m2)
ln(A)
V (m/s)
ln(V)
W (m)
ln(W)
BE (m)
rho 0.86
rho 0.88
rho −0.28
rho −0.41
rho 0.25
rho 0.27
rho −0.27
rho −0.64
rho −0.026
rho 0.16
rho −0.24
rho −0.25
rho −0.13
rho 0.036
rho −0.18
rho −0.14
rho 0.031
rho −0.036
rho 0.75
rho 0.75
rho −0.25
rho −0.072
rho 0.61
rho 0.75

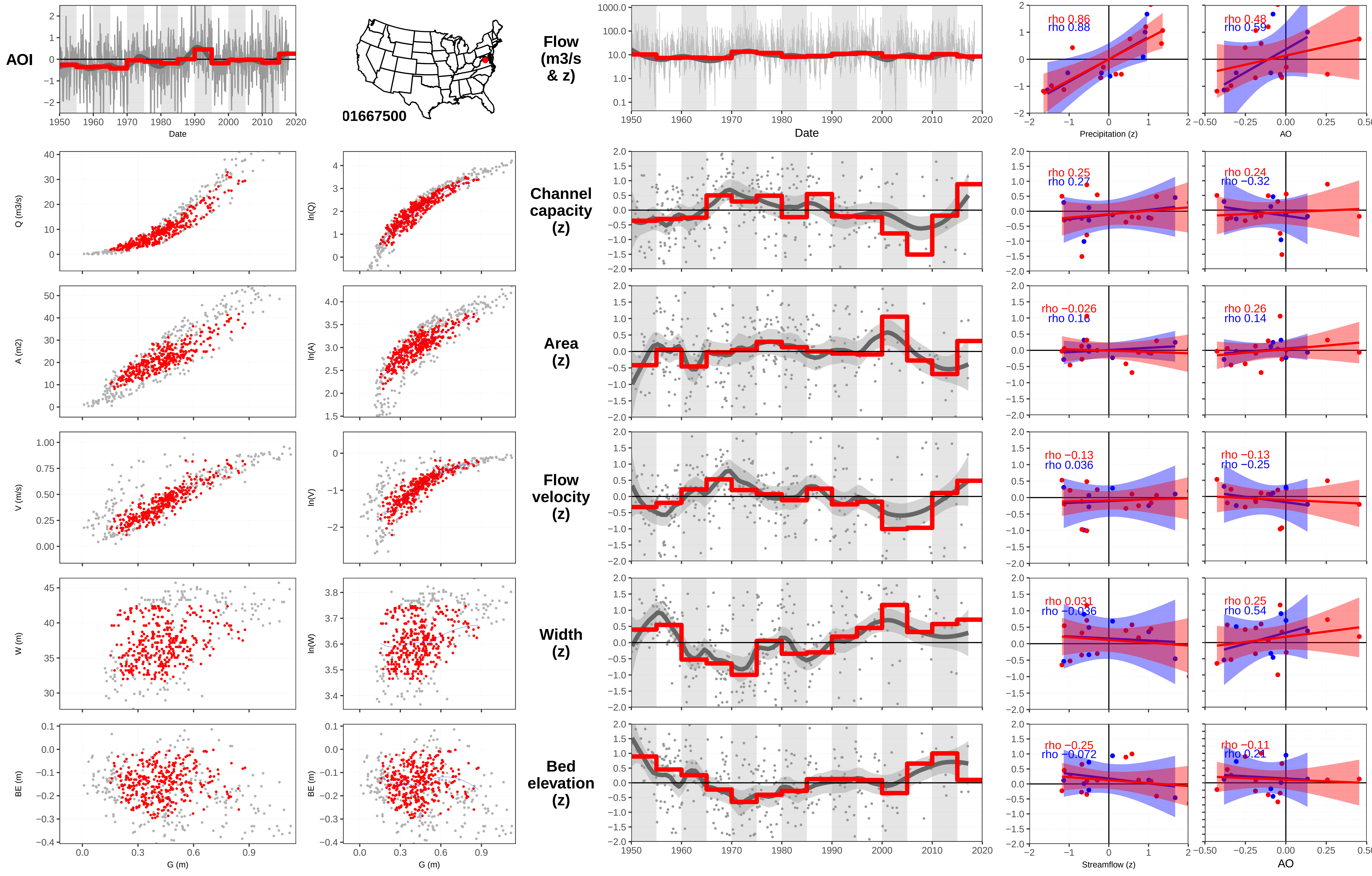
AOI
01667500
Flow (m3/s & z)
Channel capacity (z)
Area (z)
Flow velocity (z)
Width (z)
Bed elevation (z)
Date
Precipitation (z)
AO
Streamflow (z)
Q (m3/s)
ln(Q)
A (m2)
ln(A)
V (m/s)
ln(V)
W (m)
ln(W)
BE (m)
G (m)
rho 0.86
rho 0.88
rho 0.48
rho 0.59
rho 0.25
rho 0.27
rho 0.24
rho −0.32
rho −0.026
rho 0.16
rho 0.26
rho 0.14
rho −0.13
rho 0.036
rho −0.13
rho −0.25
rho 0.031
rho −0.036
rho 0.25
rho 0.54
rho −0.25
rho −0.072
rho −0.11
rho 0.21

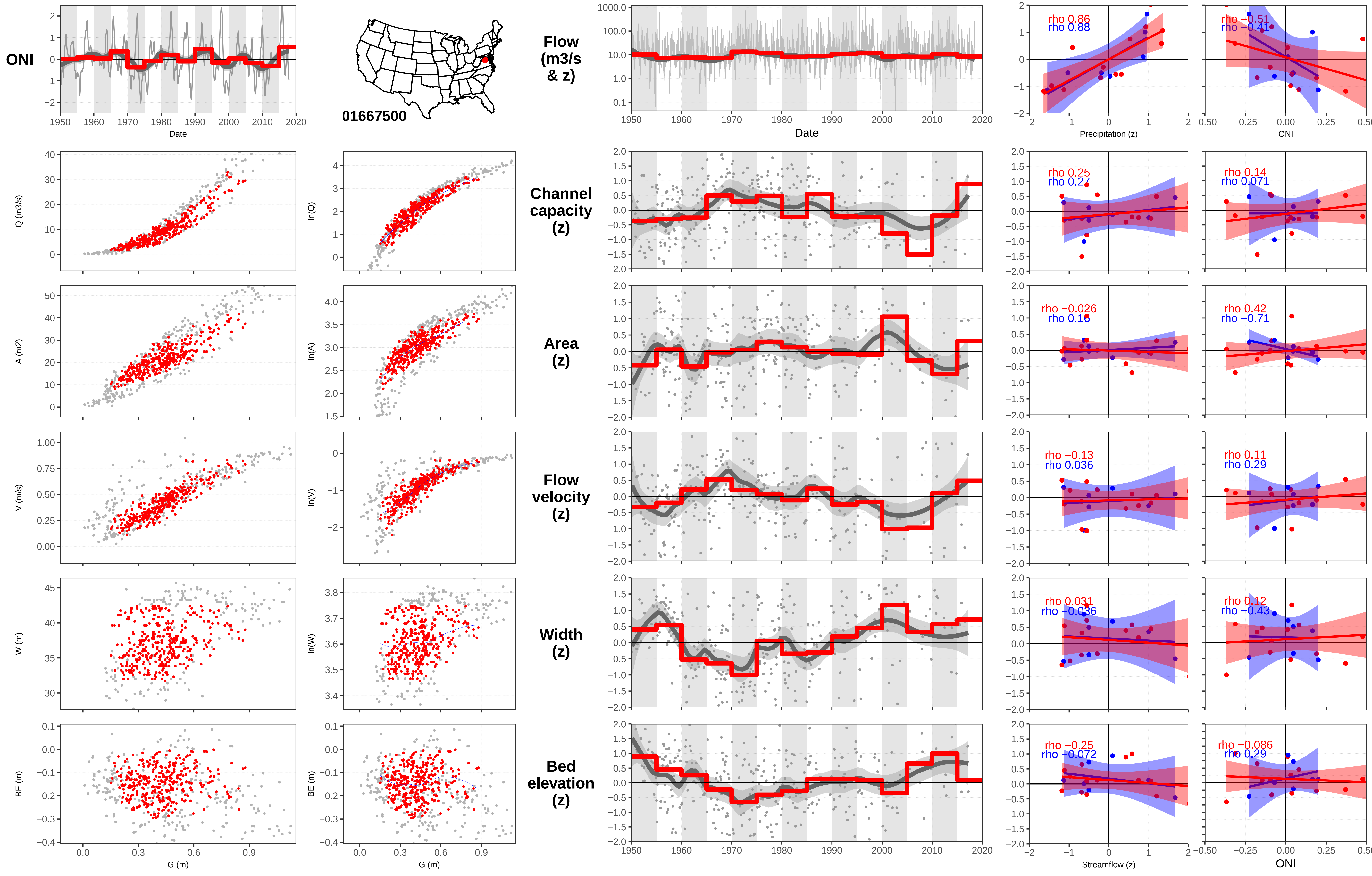

ONI
01667500
Flow (m3/s & z)
Channel capacity (z)
Area (z)
Flow velocity (z)
Width (z)
Bed elevation (z)
Date
Precipitation (z)
Streamflow (z)
ONI
G (m)
Q (m3/s)
ln(Q)
A (m2)
ln(A)
V (m/s)
ln(V)
W (m)
ln(W)
BE (m)
rho 0.86
rho 0.88
rho −0.51
rho −0.41
rho 0.25
rho 0.27
rho 0.14
rho 0.071
rho −0.026
rho 0.16
rho 0.42
rho −0.71
rho −0.13
rho 0.036
rho 0.11
rho 0.29
rho 0.031
rho −0.036
rho 0.12
rho −0.43
rho −0.25
rho −0.072
rho −0.086
rho 0.29

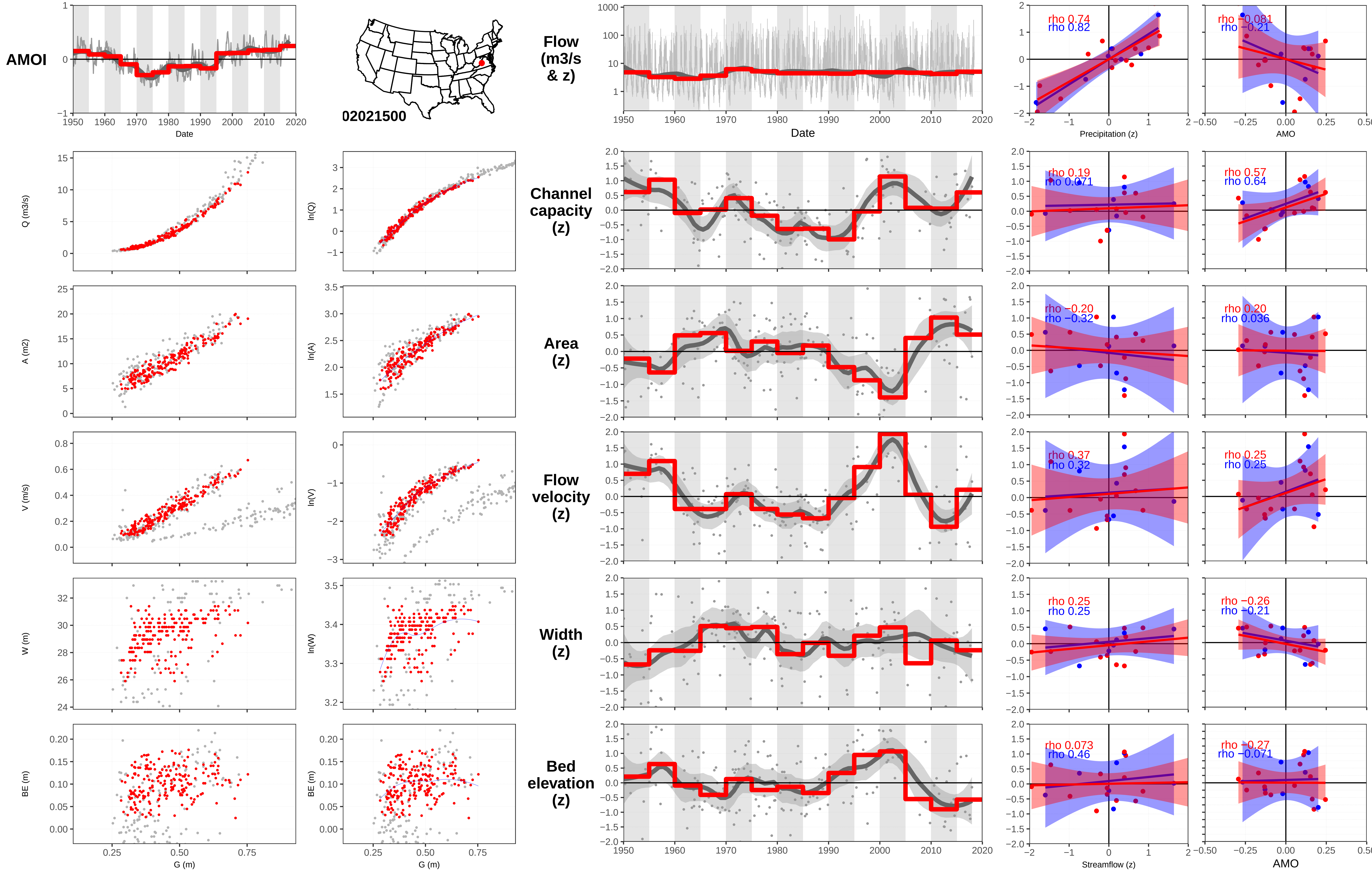

AMOI
02021500
Flow (m3/s & z)
Channel capacity (z)
Area (z)
Flow velocity (z)
Width (z)
Bed elevation (z)
Date
Precipitation (z)
Streamflow (z)
AMO
G (m)
Q (m3/s)
ln(Q)
A (m2)
ln(A)
V (m/s)
ln(V)
W (m)
ln(W)
BE (m)
rho 0.74
rho 0.82
rho −0.081
rho −0.21
rho 0.19
rho 0.071
rho 0.57
rho 0.64
rho −0.20
rho −0.32
rho 0.20
rho 0.036
rho 0.37
rho 0.32
rho 0.25
rho 0.25
rho 0.25
rho 0.25
rho −0.26
rho −0.21
rho 0.073
rho 0.46
rho −0.27
rho −0.071

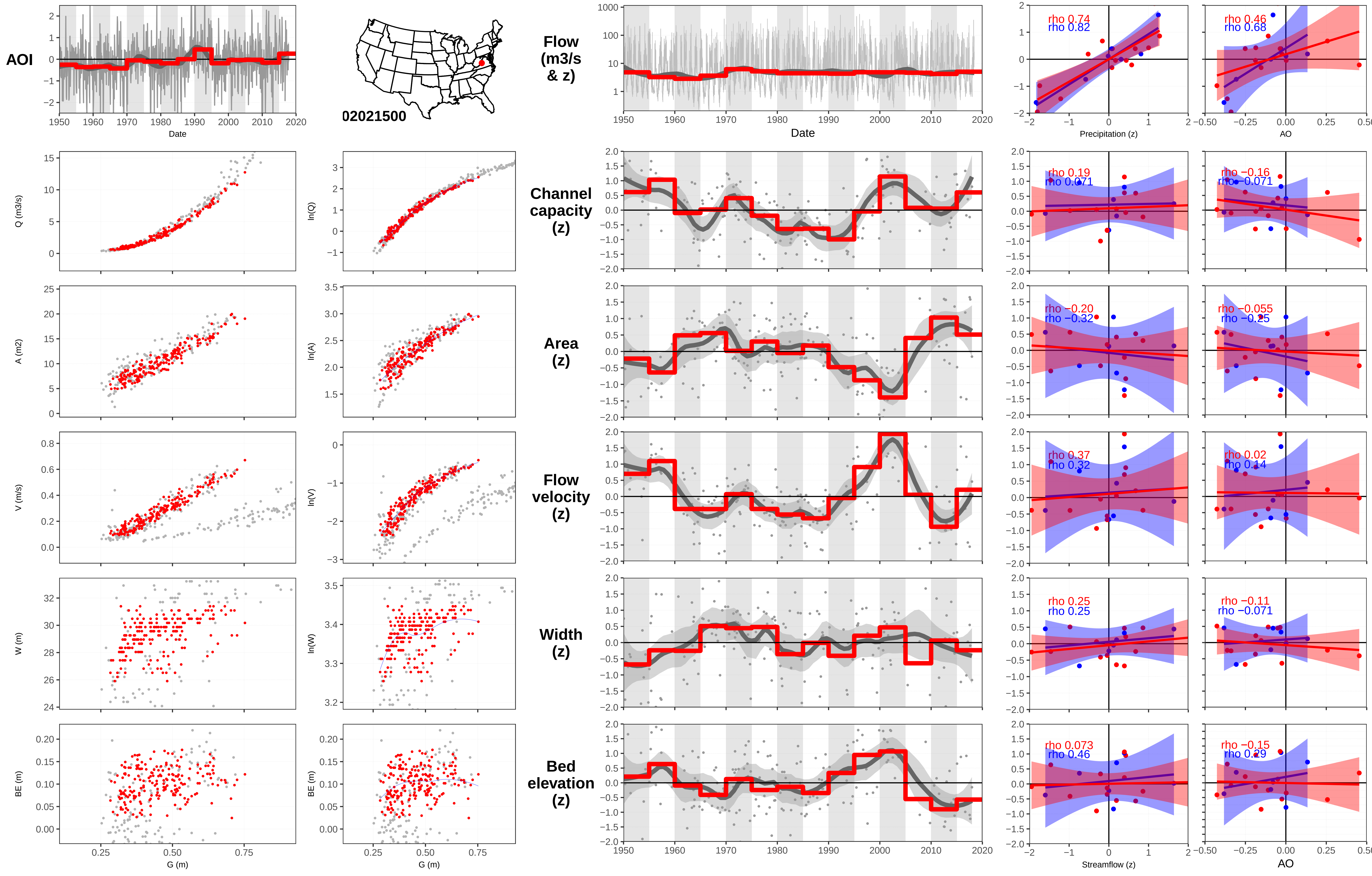

AOI
02021500
Flow (m3/s & z)
Channel capacity (z)
Area (z)
Flow velocity (z)
Width (z)
Bed elevation (z)
Date
Precipitation (z)
AO
Streamflow (z)
G (m)
Q (m3/s)
ln(Q)
A (m2)
ln(A)
V (m/s)
ln(V)
W (m)
ln(W)
BE (m)
rho 0.74
rho 0.82
rho 0.46
rho 0.68
rho 0.19
rho 0.071
rho −0.16
rho −0.071
rho −0.20
rho −0.32
rho −0.055
rho −0.25
rho 0.37
rho 0.32
rho 0.02
rho 0.14
rho 0.25
rho 0.25
rho −0.11
rho −0.071
rho 0.073
rho 0.46
rho −0.15
rho 0.29

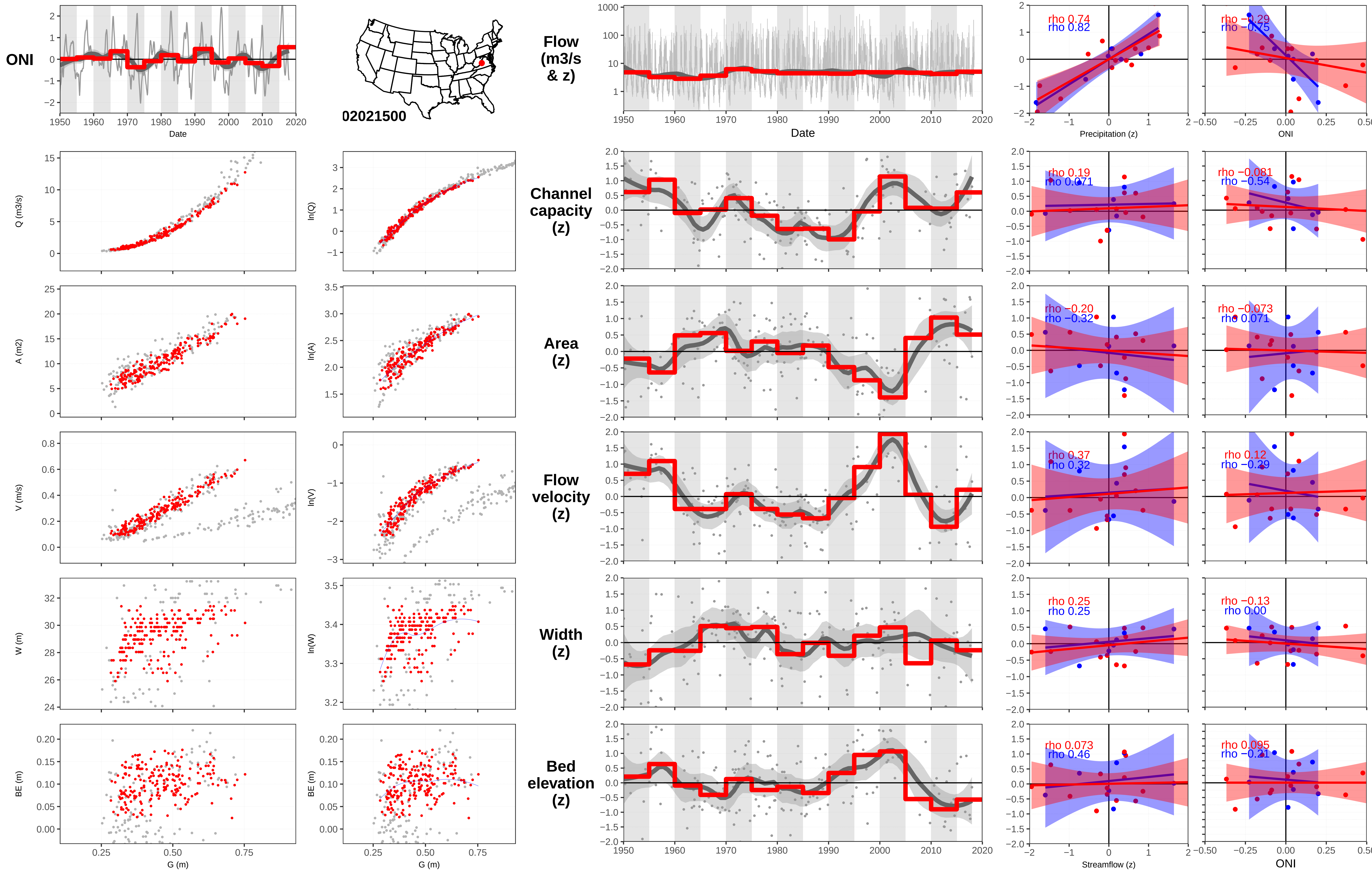

ONI
02021500
Flow (m3/s & z)
Date
Precipitation (z)
ONI
rho 0.74
rho 0.82
rho −0.29
rho −0.75
Channel capacity (z)
Q (m3/s)
ln(Q)
rho 0.19
rho 0.071
rho −0.081
rho −0.54
Area (z)
A (m2)
ln(A)
rho −0.20
rho −0.32
rho −0.073
rho 0.071
Flow velocity (z)
V (m/s)
ln(V)
rho 0.37
rho 0.32
rho 0.12
rho −0.29
Width (z)
W (m)
ln(W)
rho 0.25
rho 0.25
rho −0.13
rho 0.00
Bed elevation (z)
BE (m)
rho 0.073
rho 0.46
rho 0.095
rho −0.21
G (m)
G (m)
Streamflow (z)
ONI

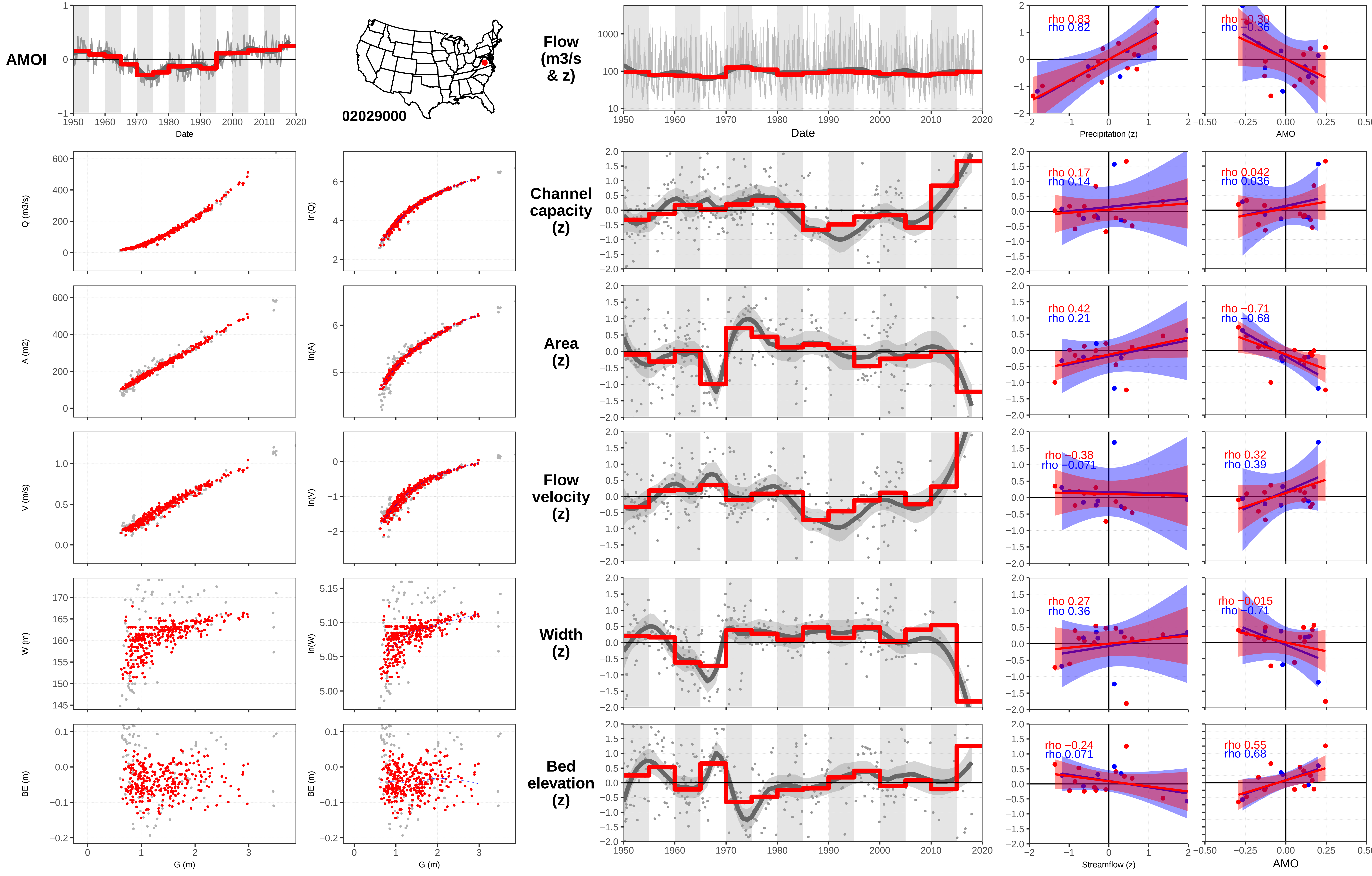

AMOI
02029000
Flow (m3/s & z)
Channel capacity (z)
Area (z)
Flow velocity (z)
Width (z)
Bed elevation (z)
Date
Precipitation (z)
AMO
Streamflow (z)
G (m)
Q (m3/s)
ln(Q)
A (m2)
ln(A)
V (m/s)
ln(V)
W (m)
ln(W)
BE (m)
rho 0.83
rho 0.82
rho −0.30
rho −0.36
rho 0.17
rho 0.14
rho 0.042
rho 0.036
rho 0.42
rho 0.21
rho −0.71
rho −0.68
rho −0.38
rho −0.071
rho 0.32
rho 0.39
rho 0.27
rho 0.36
rho −0.015
rho −0.71
rho −0.24
rho 0.071
rho 0.55
rho 0.68

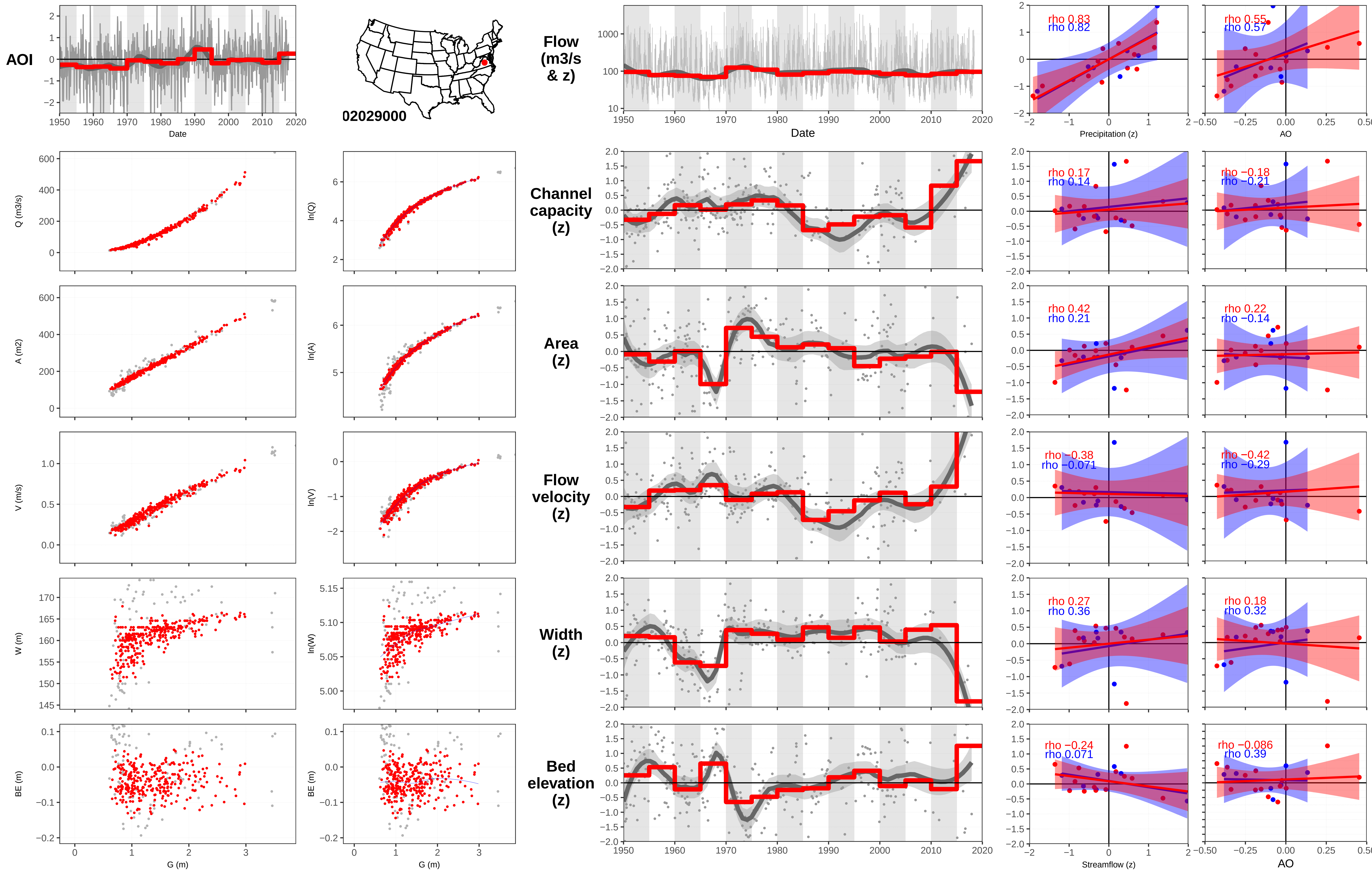
AOI
02029000
Flow (m3/s & z)
Channel capacity (z)
Area (z)
Flow velocity (z)
Width (z)
Bed elevation (z)
Date
Precipitation (z)
AO
Streamflow (z)
G (m)
Q (m3/s)
ln(Q)
A (m2)
ln(A)
V (m/s)
ln(V)
W (m)
ln(W)
BE (m)
rho 0.83
rho 0.82
rho 0.55
rho 0.57
rho 0.17
rho 0.14
rho −0.18
rho −0.21
rho 0.42
rho 0.21
rho 0.22
rho −0.14
rho −0.38
rho −0.071
rho −0.42
rho −0.29
rho 0.27
rho 0.36
rho 0.18
rho 0.32
rho −0.24
rho 0.071
rho −0.086
rho 0.39

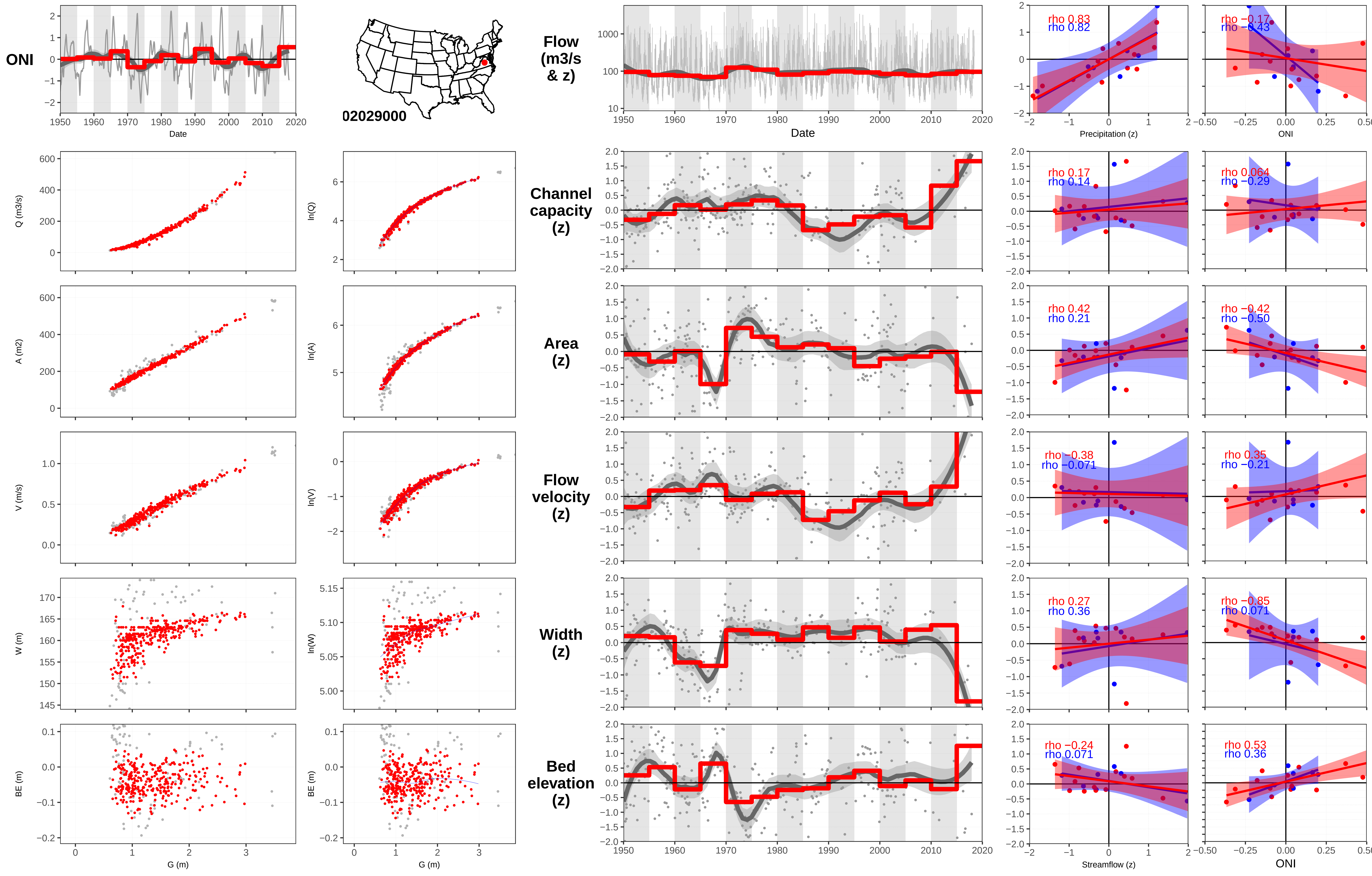

ONI
Date
02029000
Flow (m3/s & z)
Date
rho 0.83
rho 0.82
Precipitation (z)
rho −0.17
rho −0.43
ONI
Q (m3/s)
ln(Q)
Channel capacity (z)
rho 0.17
rho 0.14
rho 0.064
rho −0.29
A (m2)
ln(A)
Area (z)
rho 0.42
rho 0.21
rho −0.42
rho −0.50
V (m/s)
ln(V)
Flow velocity (z)
rho −0.38
rho −0.071
rho 0.35
rho −0.21
W (m)
ln(W)
Width (z)
rho 0.27
rho 0.36
rho −0.85
rho 0.071
BE (m)
BE (m)
Bed elevation (z)
rho −0.24
rho 0.071
rho 0.53
rho 0.36
G (m)
G (m)
Streamflow (z)
ONI

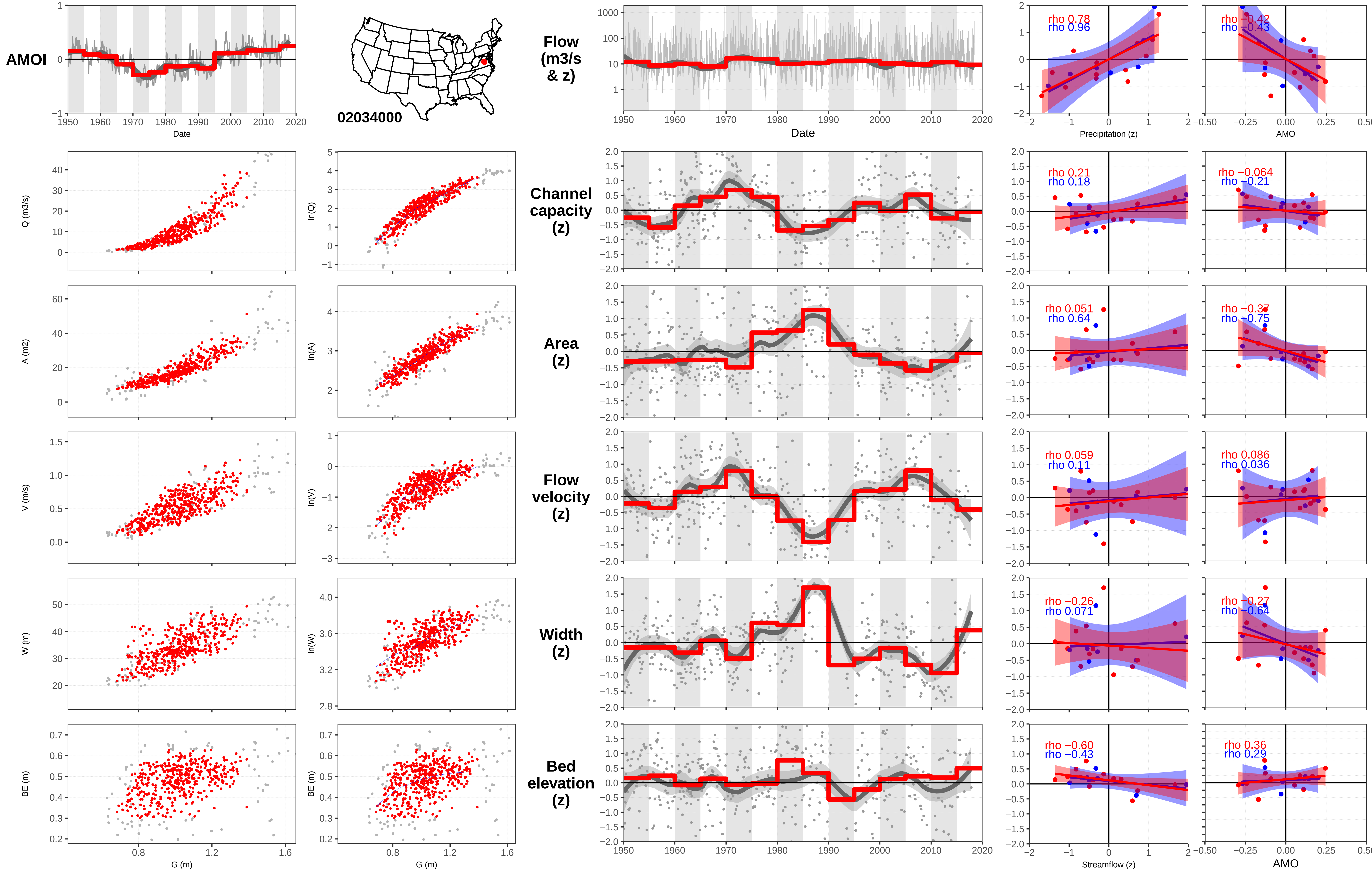

AMOI
02034000
Flow (m3/s & z)
Channel capacity (z)
Area (z)
Flow velocity (z)
Width (z)
Bed elevation (z)
Date
Precipitation (z)
AMO
Streamflow (z)
G (m)
Q (m3/s)
ln(Q)
A (m2)
ln(A)
V (m/s)
ln(V)
W (m)
ln(W)
BE (m)
rho 0.78
rho 0.96
rho −0.42
rho −0.43
rho 0.21
rho 0.18
rho −0.064
rho −0.21
rho 0.051
rho 0.64
rho −0.37
rho −0.75
rho 0.059
rho 0.11
rho 0.086
rho 0.036
rho −0.26
rho 0.071
rho −0.27
rho −0.64
rho −0.60
rho −0.43
rho 0.36
rho 0.29

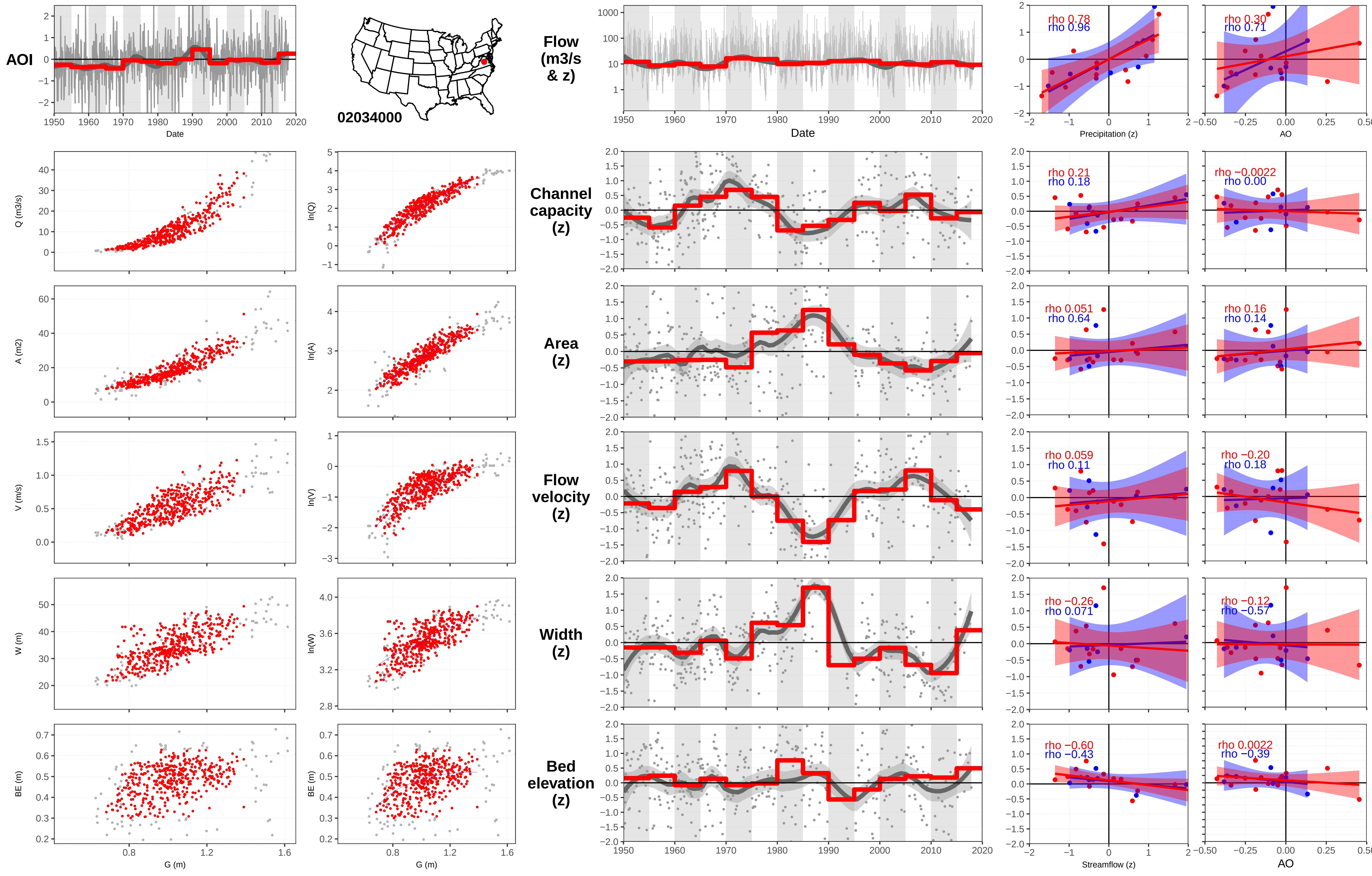

AOI
02034000
Flow (m3/s & z)
Channel capacity (z)
Area (z)
Flow velocity (z)
Width (z)
Bed elevation (z)
Date
Precipitation (z)
AO
Streamflow (z)
G (m)
Q (m3/s)
ln(Q)
A (m2)
ln(A)
V (m/s)
ln(V)
W (m)
ln(W)
BE (m)
rho 0.78
rho 0.96
rho 0.30
rho 0.71
rho 0.21
rho 0.18
rho −0.0022
rho 0.00
rho 0.051
rho 0.64
rho 0.16
rho 0.14
rho 0.059
rho 0.11
rho −0.20
rho 0.18
rho −0.26
rho 0.071
rho −0.12
rho −0.57
rho −0.60
rho −0.43
rho 0.0022
rho −0.39

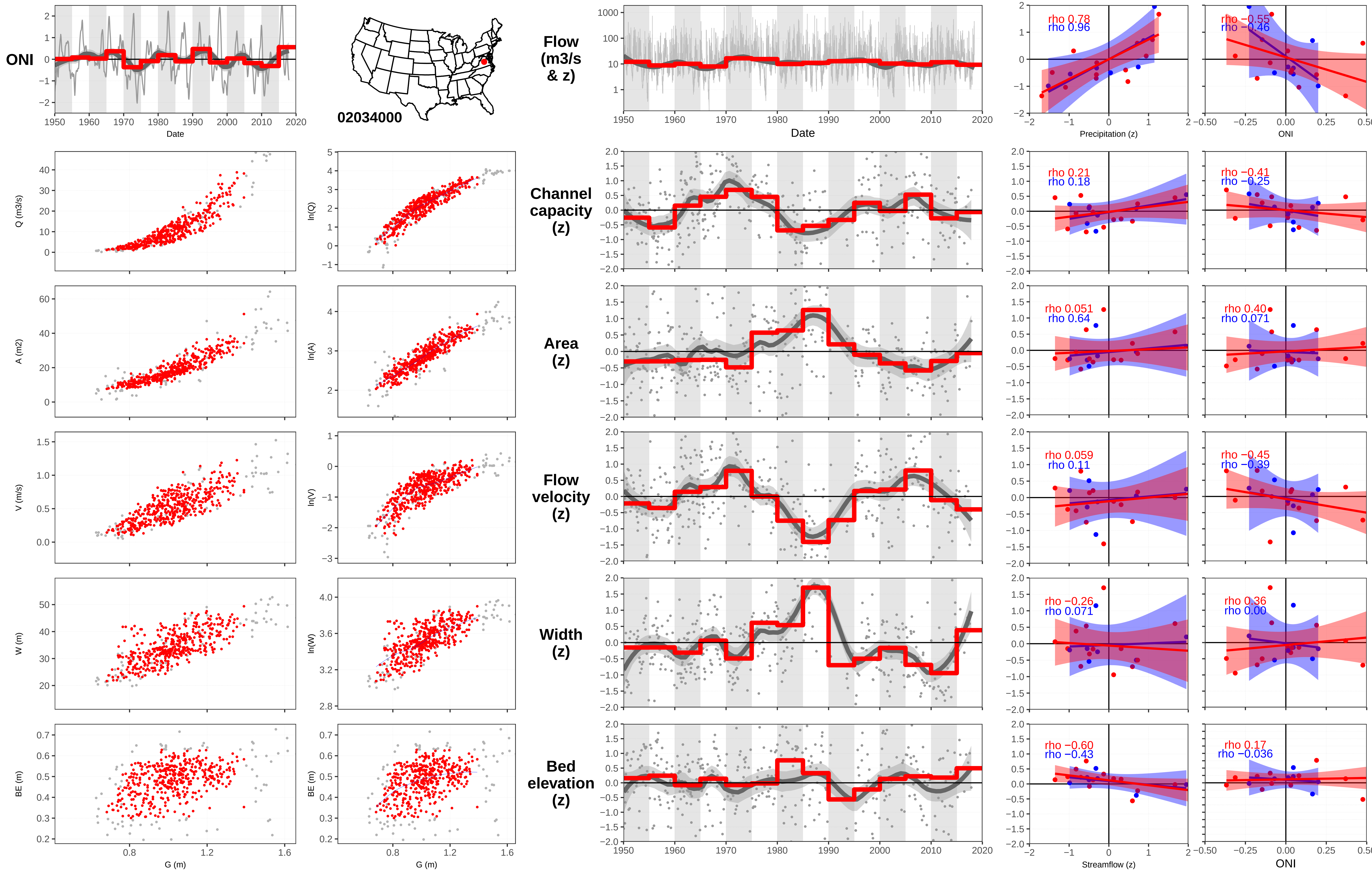
ONI
02034000
Flow (m3/s & z)
Channel capacity (z)
Area (z)
Flow velocity (z)
Width (z)
Bed elevation (z)
Date
Precipitation (z)
Streamflow (z)
ONI
G (m)
Q (m3/s)
ln(Q)
A (m2)
ln(A)
V (m/s)
ln(V)
W (m)
ln(W)
BE (m)
rho 0.78
rho 0.96
rho −0.55
rho −0.46
rho 0.21
rho 0.18
rho −0.41
rho −0.25
rho 0.051
rho 0.64
rho 0.40
rho 0.071
rho 0.059
rho 0.11
rho −0.45
rho −0.39
rho −0.26
rho 0.071
rho 0.36
rho 0.00
rho −0.60
rho −0.43
rho 0.17
rho −0.036

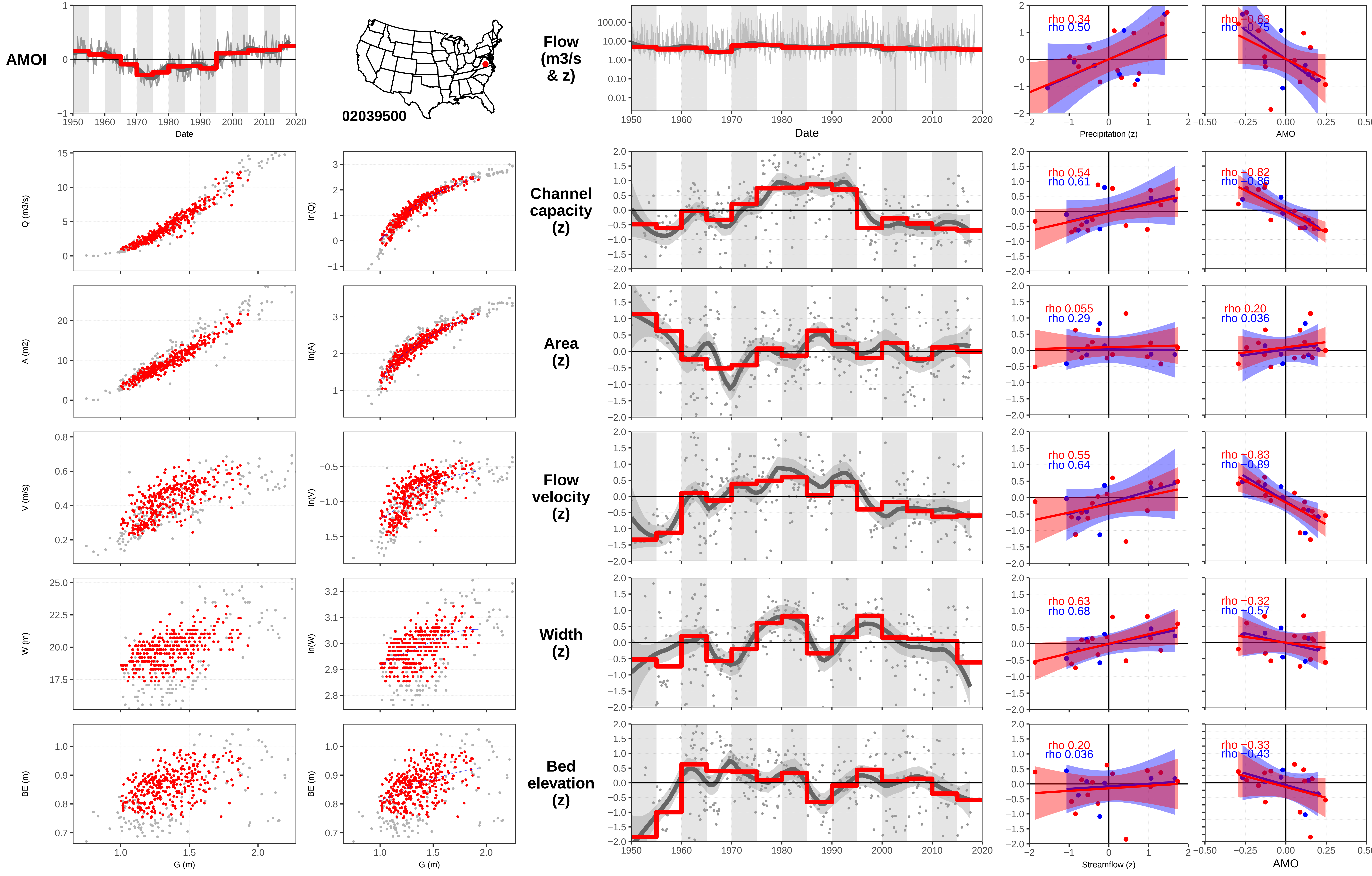

AMOI
Date
02039500
Flow (m3/s & z)
Date
Precipitation (z)
AMO
rho 0.34
rho 0.50
rho −0.63
rho −0.75
Q (m3/s)
ln(Q)
Channel capacity (z)
rho 0.54
rho 0.61
rho −0.82
rho −0.86
A (m2)
ln(A)
Area (z)
rho 0.055
rho 0.29
rho 0.20
rho 0.036
V (m/s)
ln(V)
Flow velocity (z)
rho 0.55
rho 0.64
rho −0.83
rho −0.89
W (m)
ln(W)
Width (z)
rho 0.63
rho 0.68
rho −0.32
rho −0.57
BE (m)
Bed elevation (z)
rho 0.20
rho 0.036
rho −0.33
rho −0.43
G (m)
Streamflow (z)
AMO

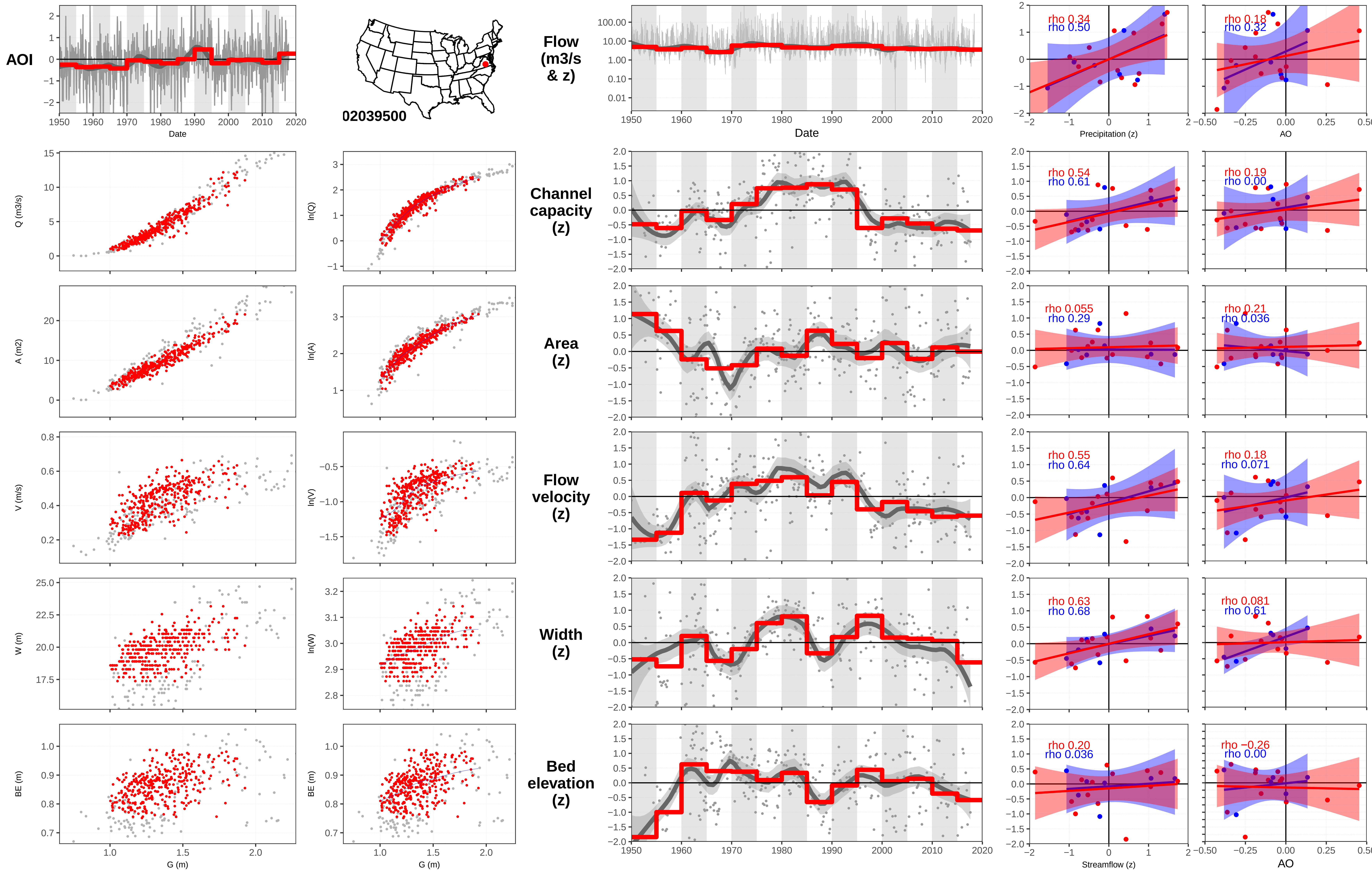

AOI
02039500
Flow (m3/s & z)
Channel capacity (z)
Area (z)
Flow velocity (z)
Width (z)
Bed elevation (z)
Date
Precipitation (z)
AO
Streamflow (z)
G (m)
Q (m3/s)
ln(Q)
A (m2)
ln(A)
V (m/s)
ln(V)
W (m)
ln(W)
BE (m)
rho 0.34
rho 0.50
rho 0.18
rho 0.32
rho 0.54
rho 0.61
rho 0.19
rho 0.00
rho 0.055
rho 0.29
rho 0.21
rho 0.036
rho 0.55
rho 0.64
rho 0.18
rho 0.071
rho 0.63
rho 0.68
rho 0.081
rho 0.61
rho 0.20
rho 0.036
rho −0.26
rho 0.00

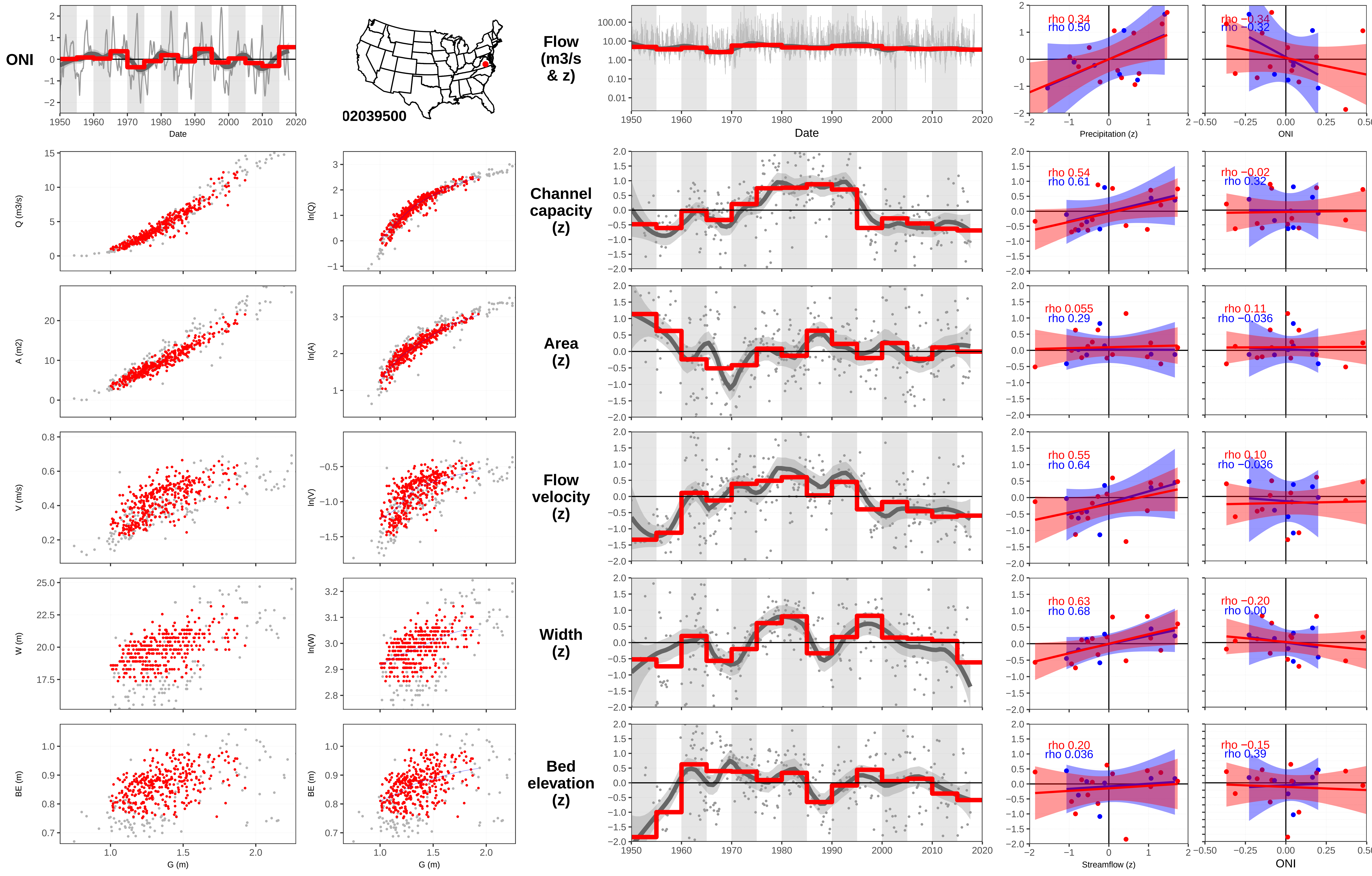

ONI
Date
02039500
Flow (m3/s & z)
Date
rho 0.34
rho 0.50
Precipitation (z)
rho −0.34
rho −0.32
ONI
Q (m3/s)
ln(Q)
Channel capacity (z)
rho 0.54
rho 0.61
rho −0.02
rho 0.32
A (m2)
ln(A)
Area (z)
rho 0.055
rho 0.29
rho 0.11
rho −0.036
V (m/s)
ln(V)
Flow velocity (z)
rho 0.55
rho 0.64
rho 0.10
rho −0.036
W (m)
ln(W)
Width (z)
rho 0.63
rho 0.68
rho −0.20
rho 0.00
BE (m)
BE (m)
Bed elevation (z)
rho 0.20
rho 0.036
rho −0.15
rho 0.39
G (m)
G (m)
Streamflow (z)
ONI

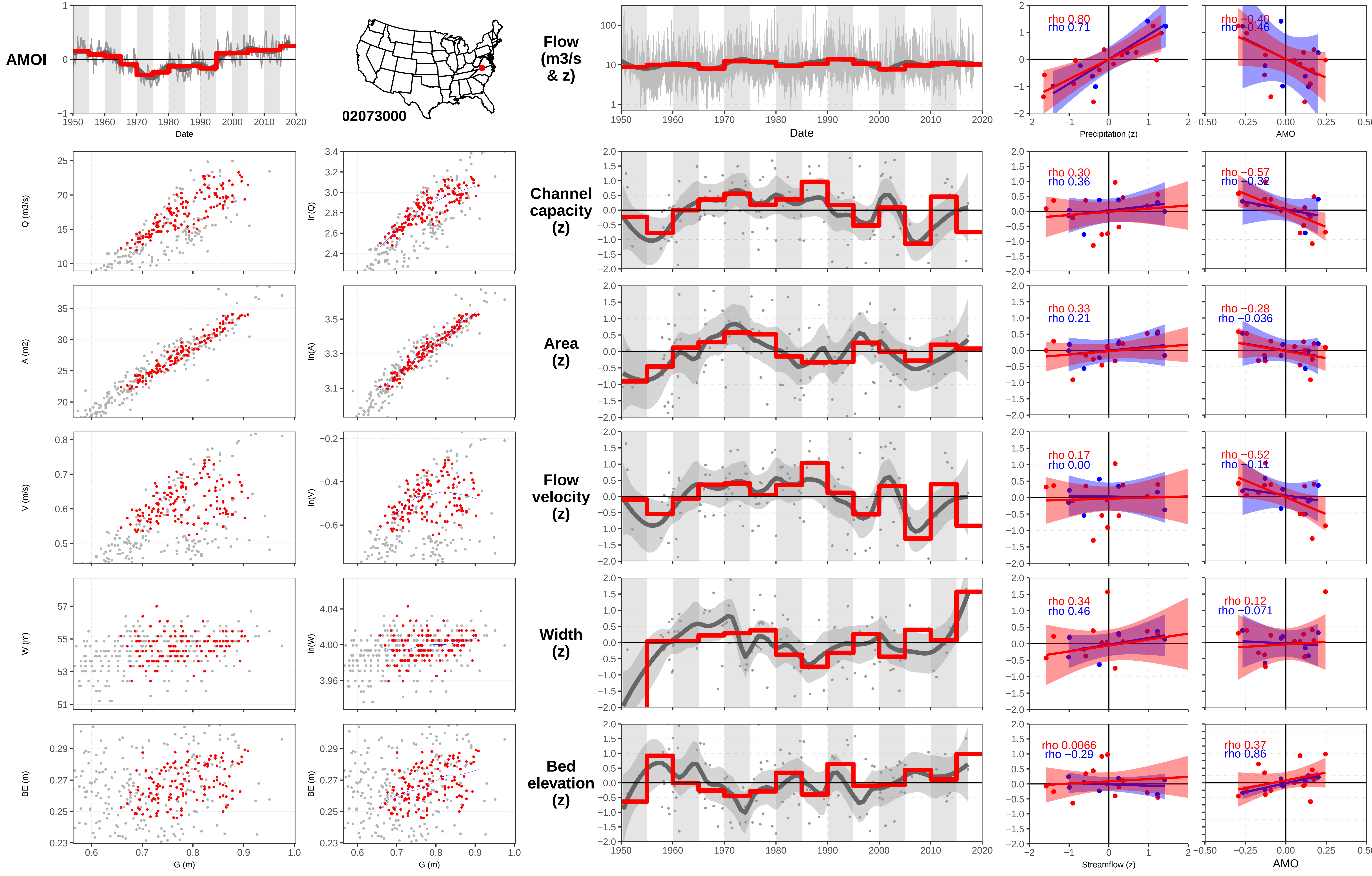

AMOI
02073000
Flow (m3/s & z)
Channel capacity (z)
Area (z)
Flow velocity (z)
Width (z)
Bed elevation (z)
Date
Precipitation (z)
AMO
Streamflow (z)
G (m)
Q (m3/s)
ln(Q)
A (m2)
ln(A)
V (m/s)
ln(V)
W (m)
ln(W)
BE (m)
rho 0.80
rho 0.71
rho −0.40
rho −0.46
rho 0.30
rho 0.36
rho −0.57
rho −0.32
rho 0.33
rho 0.21
rho −0.28
rho −0.036
rho 0.17
rho 0.00
rho −0.52
rho −0.11
rho 0.34
rho 0.46
rho 0.12
rho −0.071
rho 0.0066
rho −0.29
rho 0.37
rho 0.86

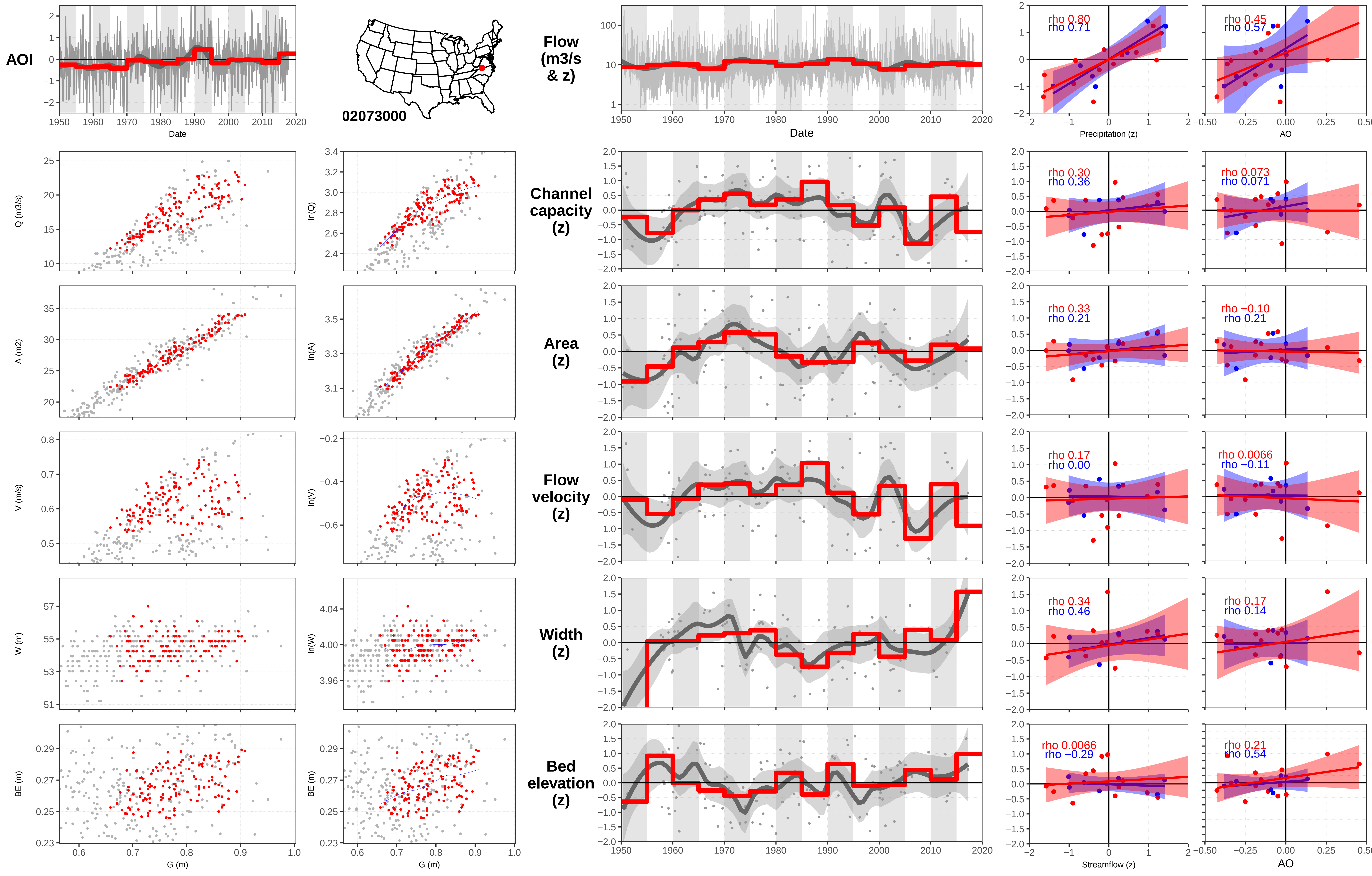

AOI
02073000
Flow (m3/s & z)
Channel capacity (z)
Area (z)
Flow velocity (z)
Width (z)
Bed elevation (z)
Date
Precipitation (z)
AO
Streamflow (z)
G (m)
Q (m3/s)
ln(Q)
A (m2)
ln(A)
V (m/s)
ln(V)
W (m)
ln(W)
BE (m)
rho 0.80
rho 0.71
rho 0.45
rho 0.57
rho 0.30
rho 0.36
rho 0.073
rho 0.071
rho 0.33
rho 0.21
rho −0.10
rho 0.21
rho 0.17
rho 0.00
rho 0.0066
rho −0.11
rho 0.34
rho 0.46
rho 0.17
rho 0.14
rho 0.0066
rho −0.29
rho 0.21
rho 0.54

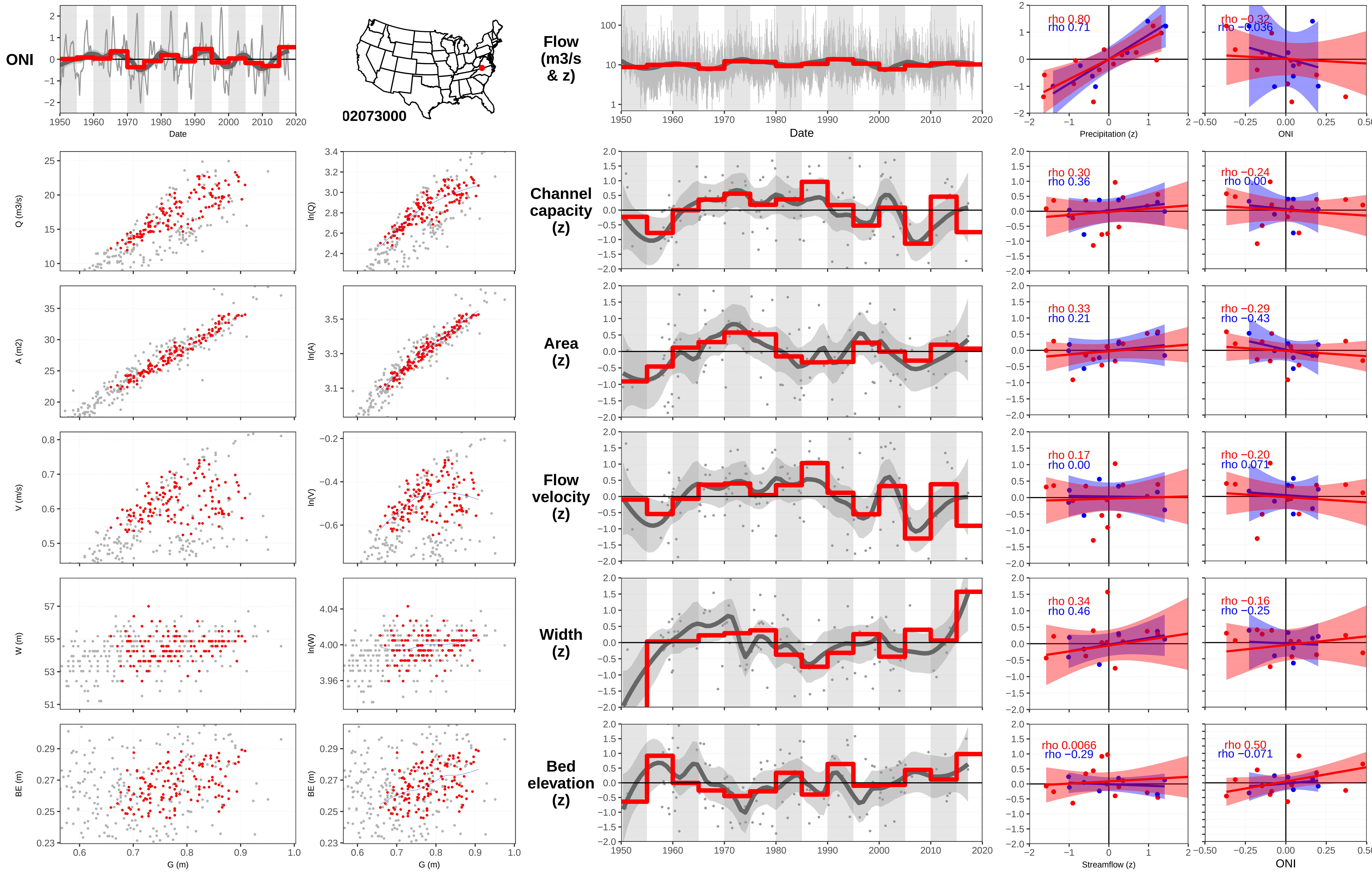

ONI
02073000
Flow (m3/s & z)
Channel capacity (z)
Area (z)
Flow velocity (z)
Width (z)
Bed elevation (z)
Date
Precipitation (z)
Streamflow (z)
ONI
Q (m3/s)
ln(Q)
A (m2)
ln(A)
V (m/s)
ln(V)
W (m)
ln(W)
BE (m)
G (m)
rho 0.80
rho 0.71
rho −0.32
rho −0.036
rho 0.30
rho 0.36
rho −0.24
rho 0.00
rho 0.33
rho 0.21
rho −0.29
rho −0.43
rho 0.17
rho 0.00
rho −0.20
rho 0.071
rho 0.34
rho 0.46
rho −0.16
rho −0.25
rho 0.0066
rho −0.29
rho 0.50
rho −0.071

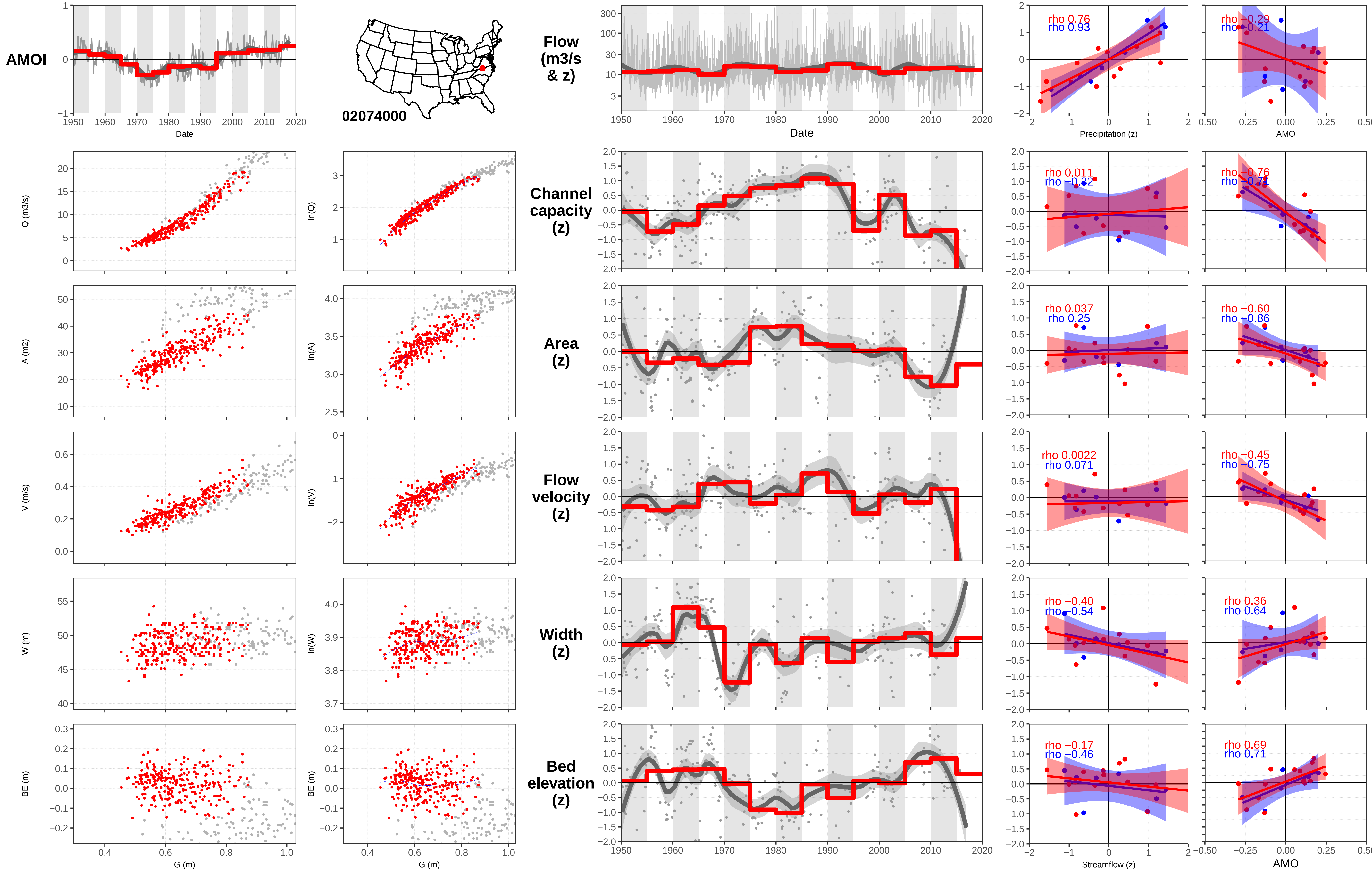
AMOI
Date
02074000
Flow (m3/s & z)
Date
rho 0.76
rho 0.93
Precipitation (z)
rho −0.29
rho −0.21
AMO
Q (m3/s)
ln(Q)
Channel capacity (z)
rho 0.011
rho −0.32
rho −0.76
rho −0.71
A (m2)
ln(A)
Area (z)
rho 0.037
rho 0.25
rho −0.60
rho −0.86
V (m/s)
ln(V)
Flow velocity (z)
rho 0.0022
rho 0.071
rho −0.45
rho −0.75
W (m)
ln(W)
Width (z)
rho −0.40
rho −0.54
rho 0.36
rho 0.64
BE (m)
Bed elevation (z)
rho −0.17
rho −0.46
rho 0.69
rho 0.71
G (m)
Streamflow (z)
AMO

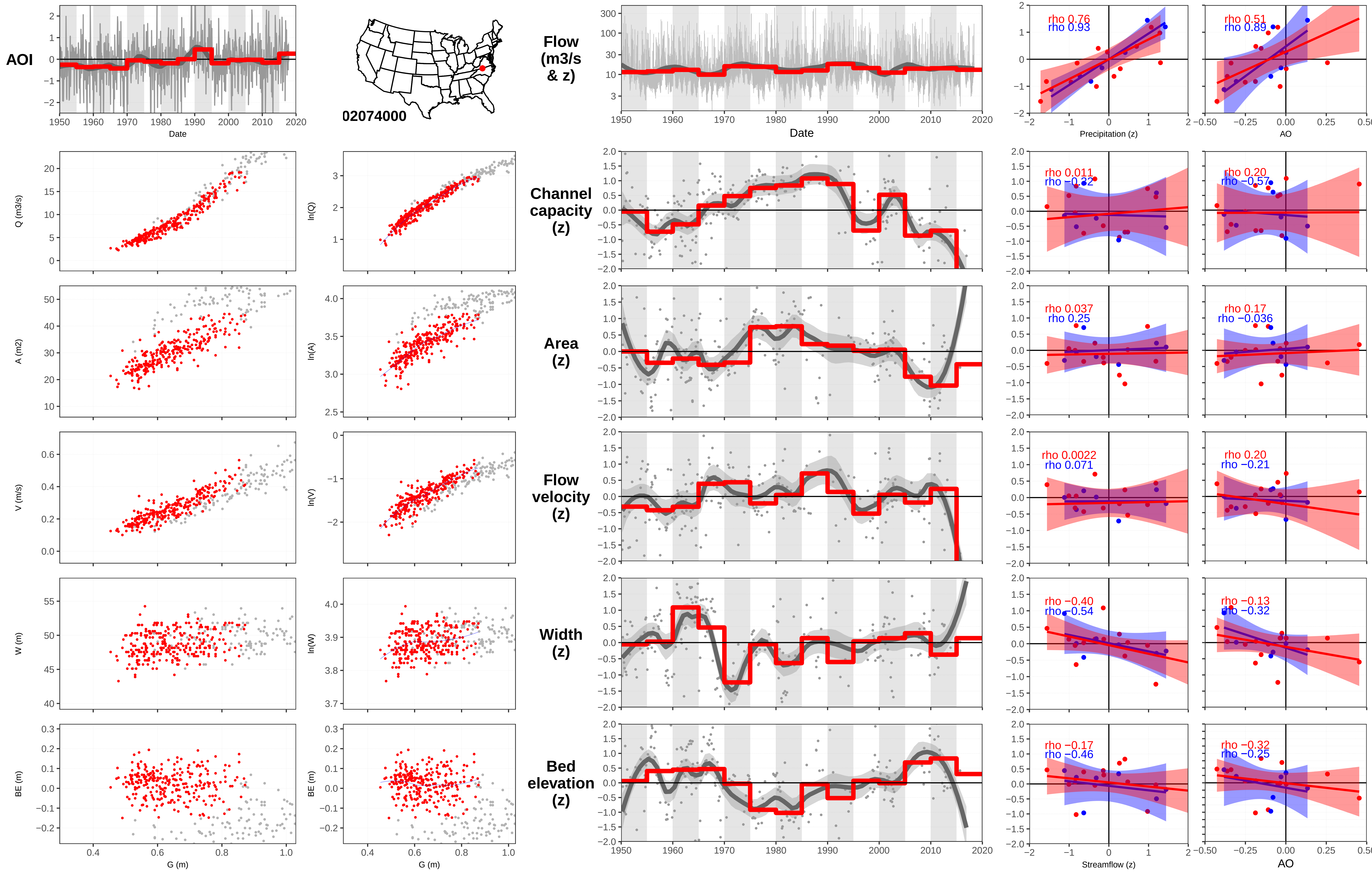

AOI
02074000
Flow (m3/s & z)
Channel capacity (z)
Area (z)
Flow velocity (z)
Width (z)
Bed elevation (z)
Date
Precipitation (z)
Streamflow (z)
AO
G (m)
Q (m3/s)
ln(Q)
A (m2)
ln(A)
V (m/s)
ln(V)
W (m)
ln(W)
BE (m)
rho 0.76
rho 0.93
rho 0.51
rho 0.89
rho 0.011
rho −0.32
rho 0.20
rho −0.57
rho 0.037
rho 0.25
rho 0.17
rho −0.036
rho 0.0022
rho 0.071
rho 0.20
rho −0.21
rho −0.40
rho −0.54
rho −0.13
rho −0.32
rho −0.17
rho −0.46
rho −0.32
rho −0.25

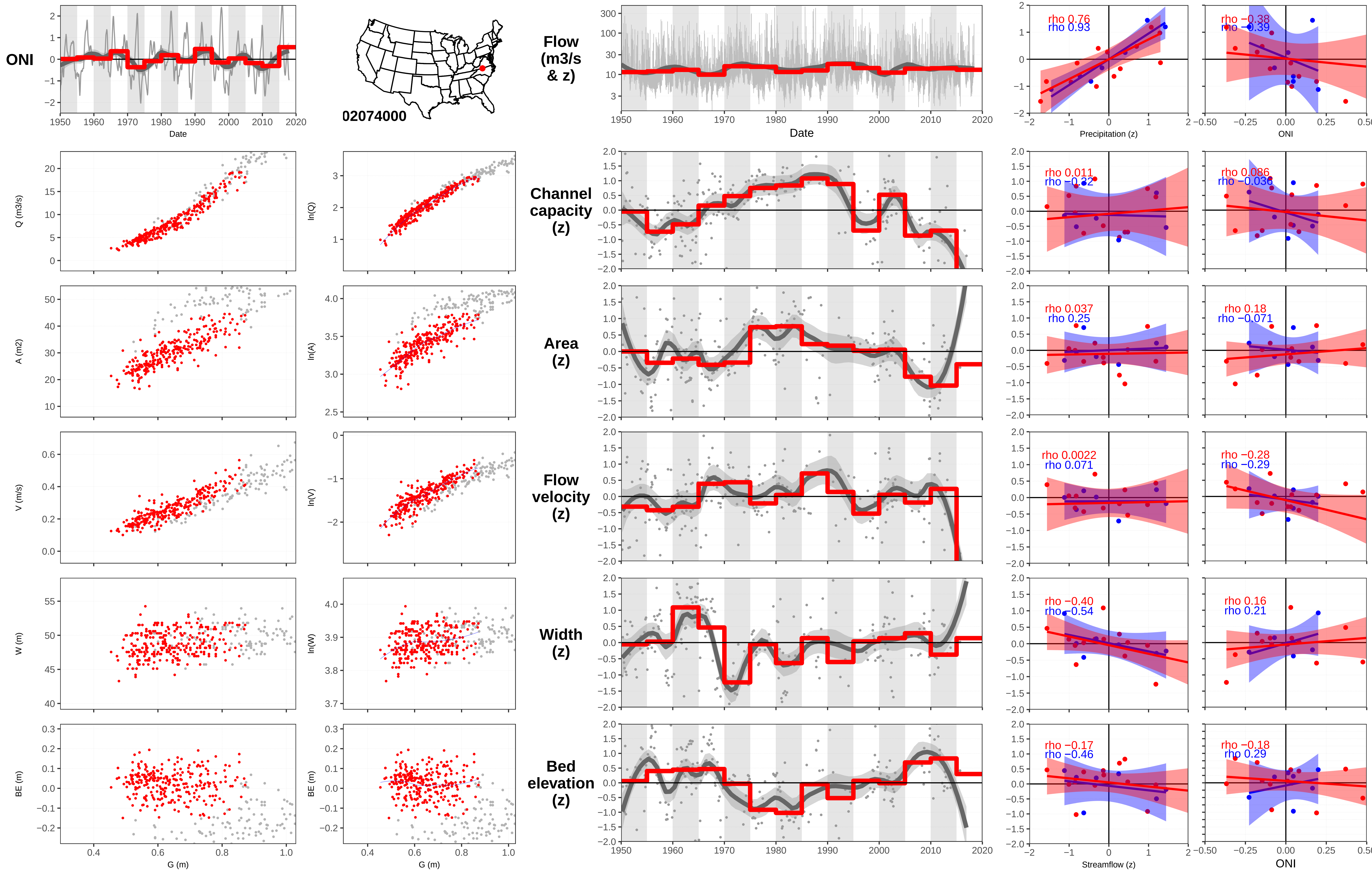

ONI
02074000
Flow (m3/s & z)
Channel capacity (z)
Area (z)
Flow velocity (z)
Width (z)
Bed elevation (z)
Date
Precipitation (z)
Streamflow (z)
ONI
Q (m3/s)
ln(Q)
A (m2)
ln(A)
V (m/s)
ln(V)
W (m)
ln(W)
BE (m)
G (m)
rho 0.76
rho 0.93
rho −0.38
rho −0.39
rho 0.011
rho −0.32
rho 0.086
rho −0.036
rho 0.037
rho 0.25
rho 0.18
rho −0.071
rho 0.0022
rho 0.071
rho −0.28
rho −0.29
rho −0.40
rho −0.54
rho 0.16
rho 0.21
rho −0.17
rho −0.46
rho −0.18
rho 0.29

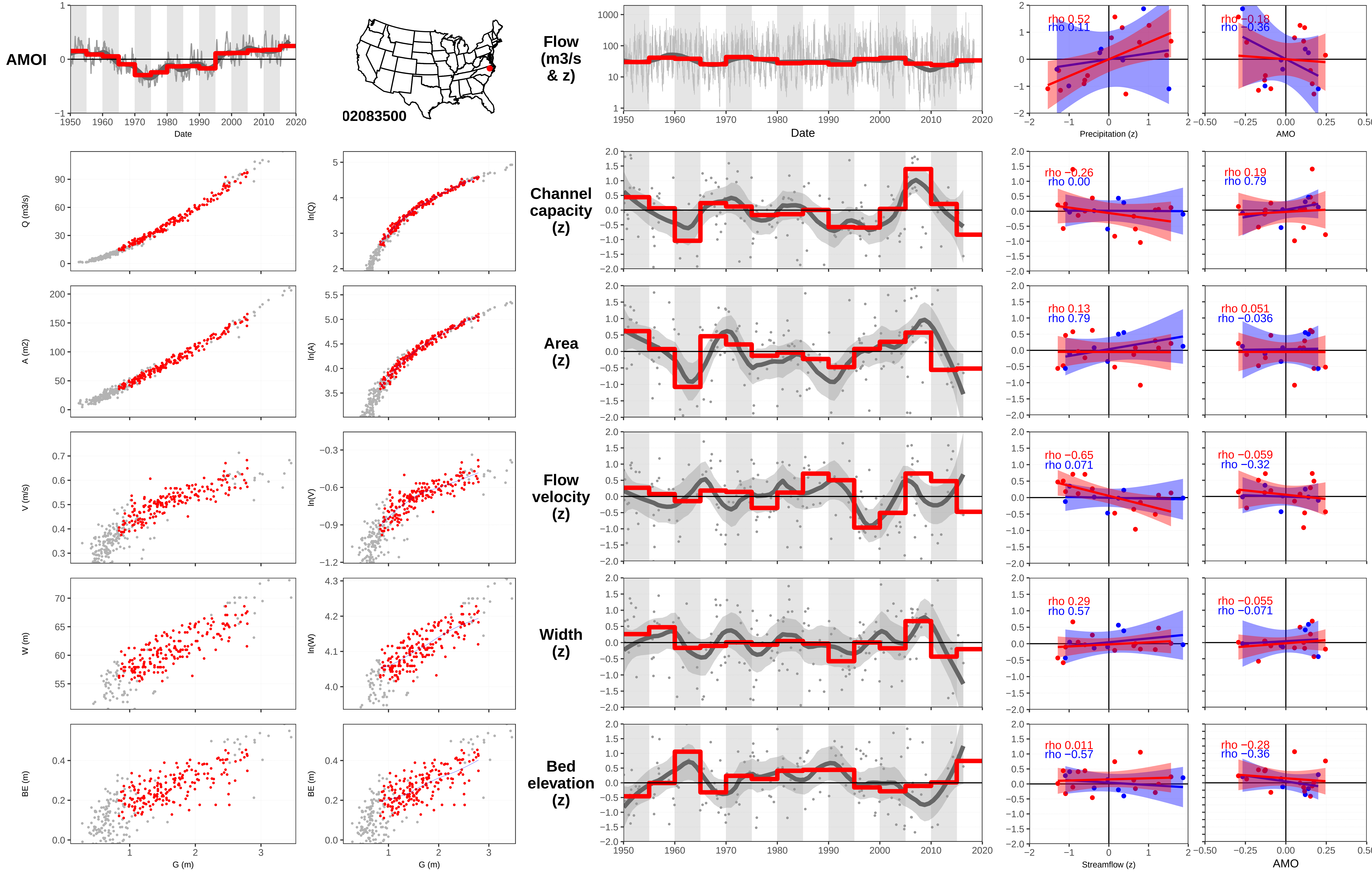
AMOI
Date
02083500
Flow (m3/s & z)
Date
rho 0.52
rho 0.11
Precipitation (z)
rho −0.18
rho −0.36
AMO
Q (m3/s)
ln(Q)
Channel capacity (z)
rho −0.26
rho 0.00
rho 0.19
rho 0.79
A (m2)
ln(A)
Area (z)
rho 0.13
rho 0.79
rho 0.051
rho −0.036
V (m/s)
ln(V)
Flow velocity (z)
rho −0.65
rho 0.071
rho −0.059
rho −0.32
W (m)
ln(W)
Width (z)
rho 0.29
rho 0.57
rho −0.055
rho −0.071
BE (m)
G (m)
Bed elevation (z)
rho 0.011
rho −0.57
rho −0.28
rho −0.36
Streamflow (z)
AMO

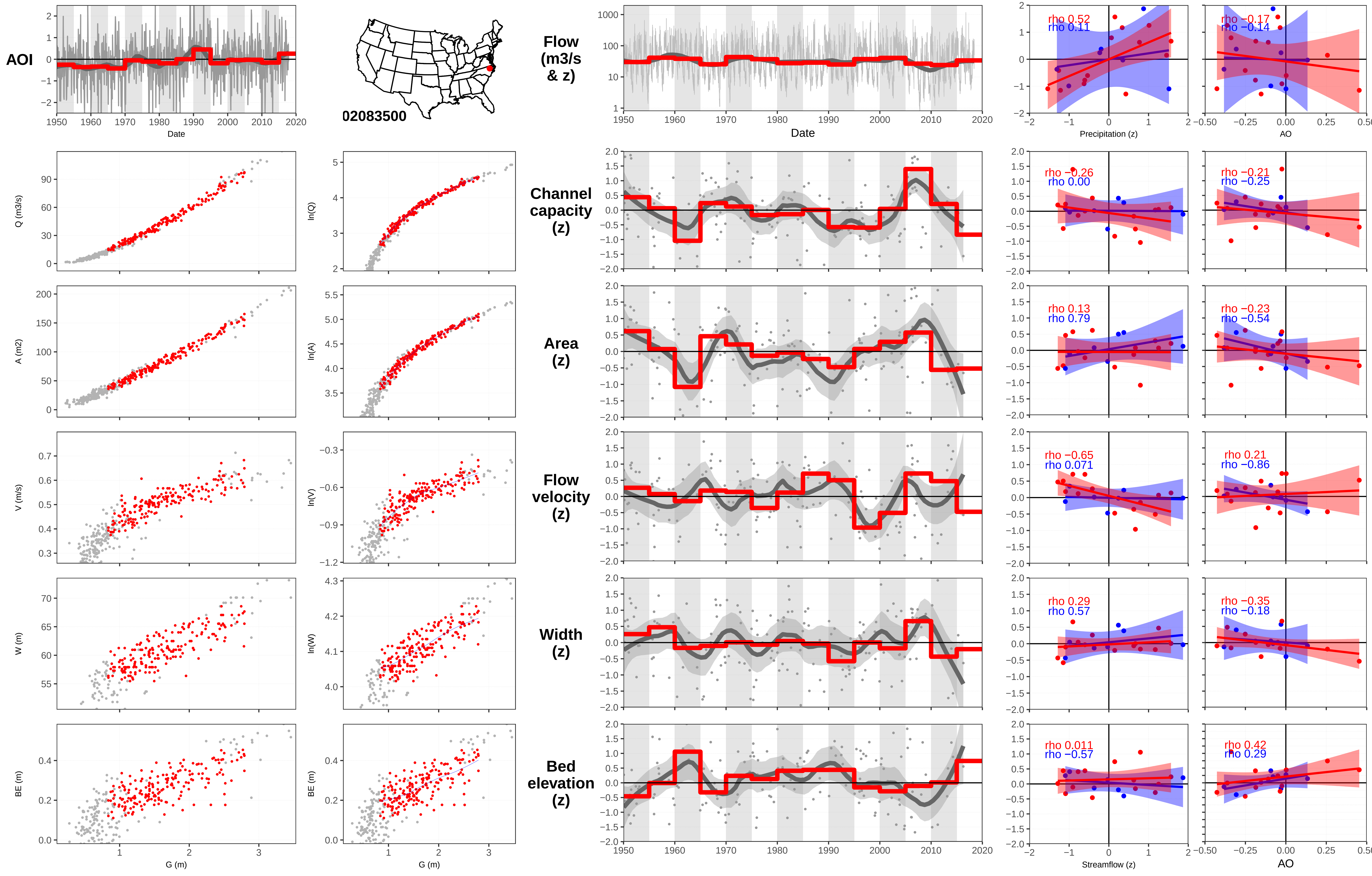

AOI
02083500
Flow (m3/s & z)
Channel capacity (z)
Area (z)
Flow velocity (z)
Width (z)
Bed elevation (z)
Date
Precipitation (z)
AO
Streamflow (z)
G (m)
Q (m3/s)
ln(Q)
A (m2)
ln(A)
V (m/s)
ln(V)
W (m)
ln(W)
BE (m)
rho 0.52
rho 0.11
rho −0.17
rho −0.14
rho −0.26
rho 0.00
rho −0.21
rho −0.25
rho 0.13
rho 0.79
rho −0.23
rho −0.54
rho −0.65
rho 0.071
rho 0.21
rho −0.86
rho 0.29
rho 0.57
rho −0.35
rho −0.18
rho 0.011
rho −0.57
rho 0.42
rho 0.29

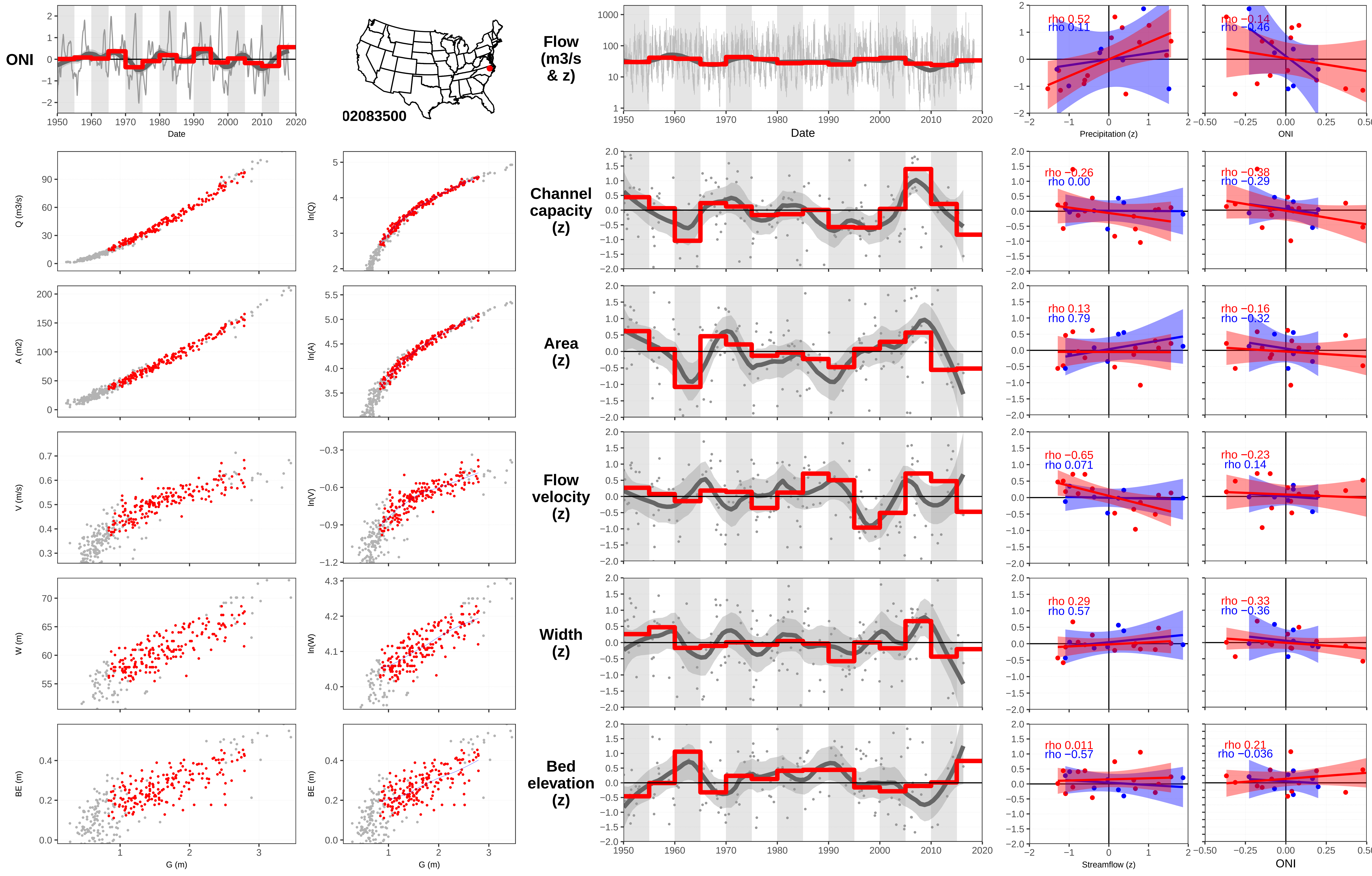

ONI
02083500
Flow (m3/s & z)
Date
Precipitation (z)
ONI
rho 0.52
rho 0.11
rho −0.14
rho −0.46
Channel capacity (z)
rho −0.26
rho 0.00
rho −0.38
rho −0.29
Q (m3/s)
ln(Q)
Area (z)
rho 0.13
rho 0.79
rho −0.16
rho −0.32
A (m2)
ln(A)
Flow velocity (z)
rho −0.65
rho 0.071
rho −0.23
rho 0.14
V (m/s)
ln(V)
Width (z)
rho 0.29
rho 0.57
rho −0.33
rho −0.36
W (m)
ln(W)
Bed elevation (z)
rho 0.011
rho −0.57
rho 0.21
rho −0.036
BE (m)
G (m)
Streamflow (z)

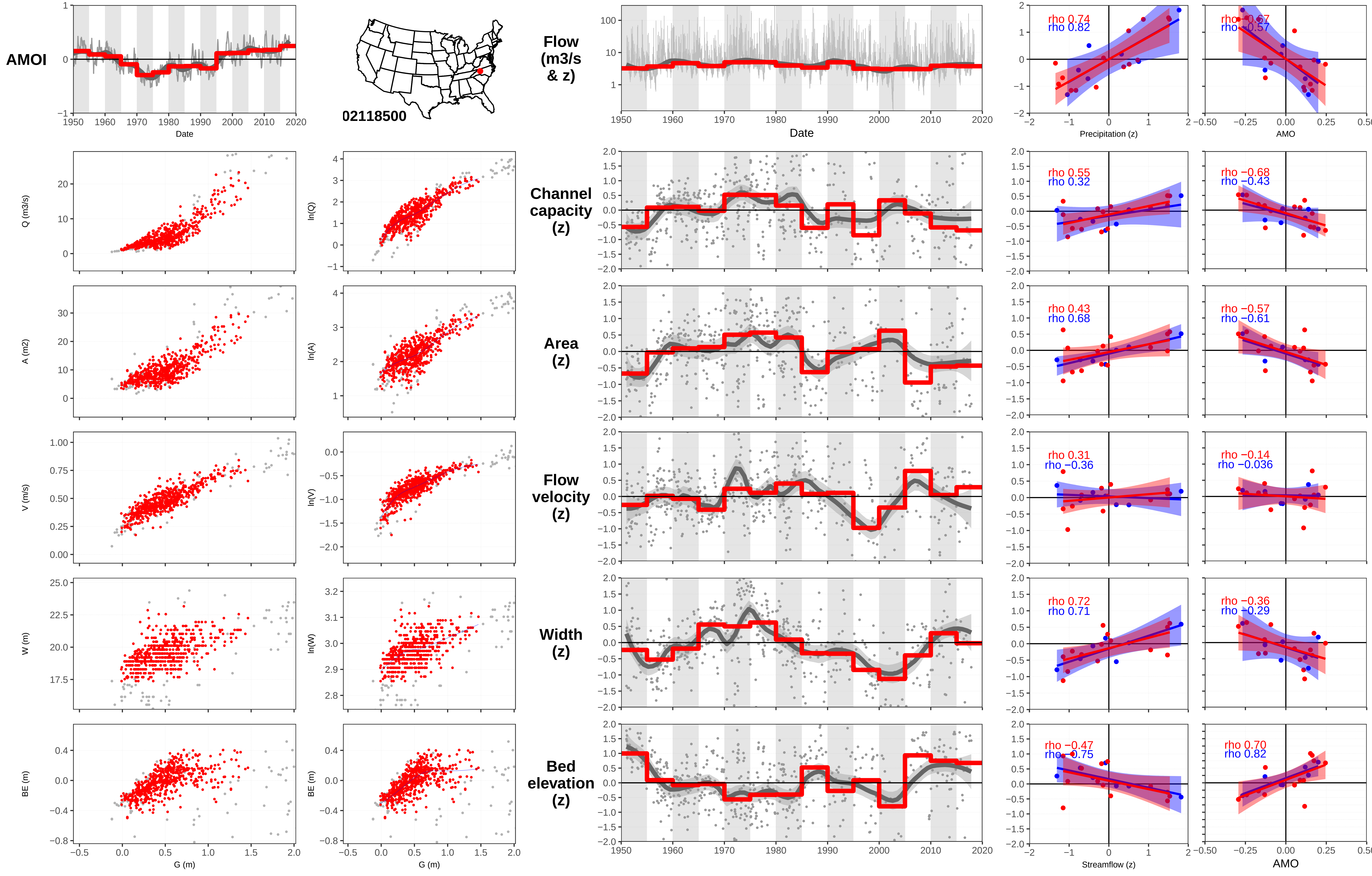

AMOI
02118500
Flow (m3/s & z)
Date
Precipitation (z)
AMO
rho 0.74
rho 0.82
rho −0.57
rho −0.57
Q (m3/s)
ln(Q)
Channel capacity (z)
rho 0.55
rho 0.32
rho −0.68
rho −0.43
A (m2)
ln(A)
Area (z)
rho 0.43
rho 0.68
rho −0.57
rho −0.61
V (m/s)
ln(V)
Flow velocity (z)
rho 0.31
rho −0.36
rho −0.14
rho −0.036
W (m)
ln(W)
Width (z)
rho 0.72
rho 0.71
rho −0.36
rho −0.29
BE (m)
Bed elevation (z)
rho −0.47
rho −0.75
rho 0.70
rho 0.82
G (m)
Streamflow (z)
AMO

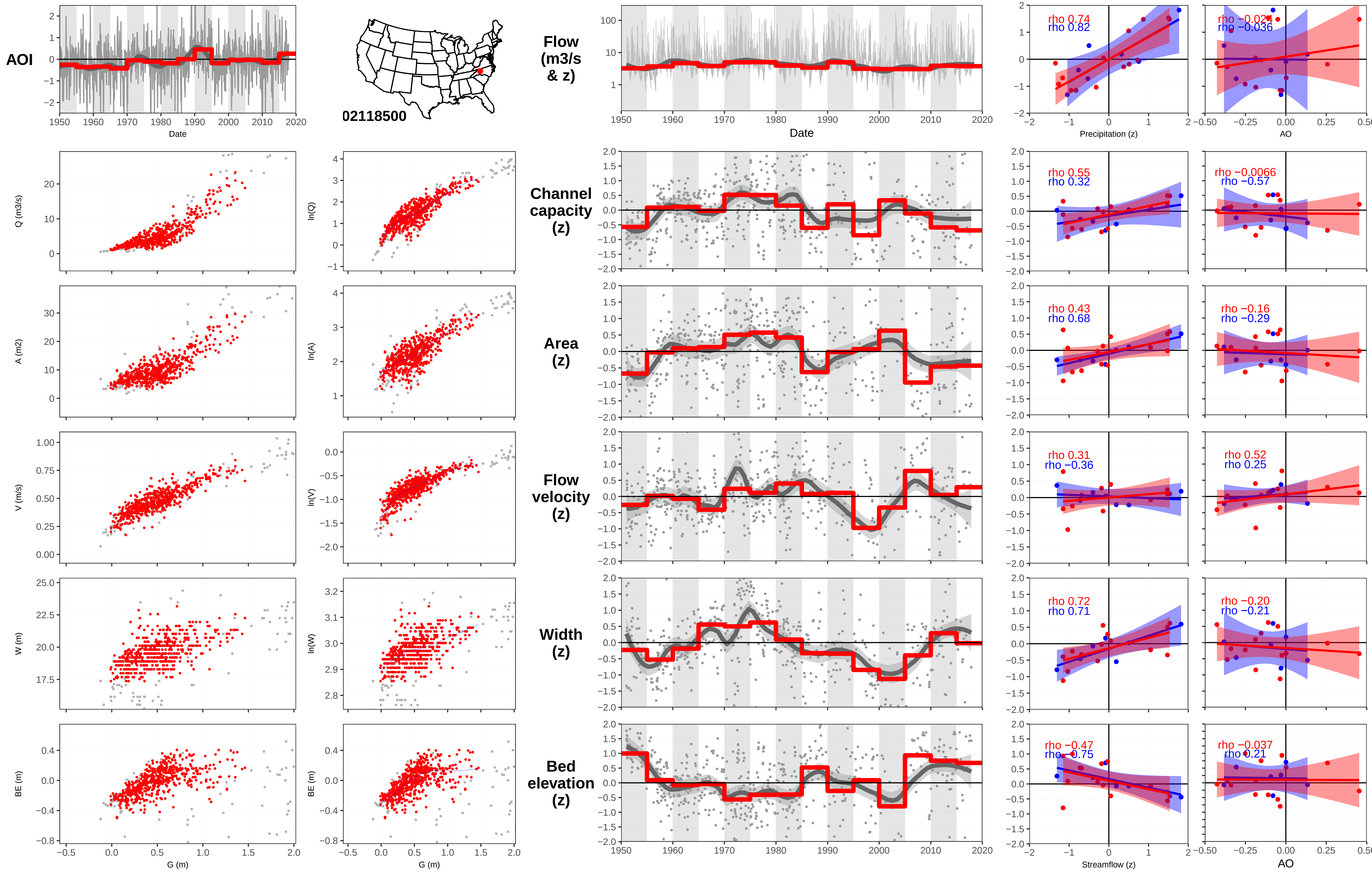

AOI
02118500
Flow (m3/s & z)
Channel capacity (z)
Area (z)
Flow velocity (z)
Width (z)
Bed elevation (z)
Date
Precipitation (z)
AO
Streamflow (z)
G (m)
Q (m3/s)
ln(Q)
A (m2)
ln(A)
V (m/s)
ln(V)
W (m)
ln(W)
BE (m)
rho 0.74
rho 0.82
rho −0.024
rho −0.036
rho 0.55
rho 0.32
rho −0.0066
rho −0.57
rho 0.43
rho 0.68
rho −0.16
rho −0.29
rho 0.31
rho −0.36
rho 0.52
rho 0.25
rho 0.72
rho 0.71
rho −0.20
rho −0.21
rho −0.47
rho −0.75
rho −0.037
rho 0.21

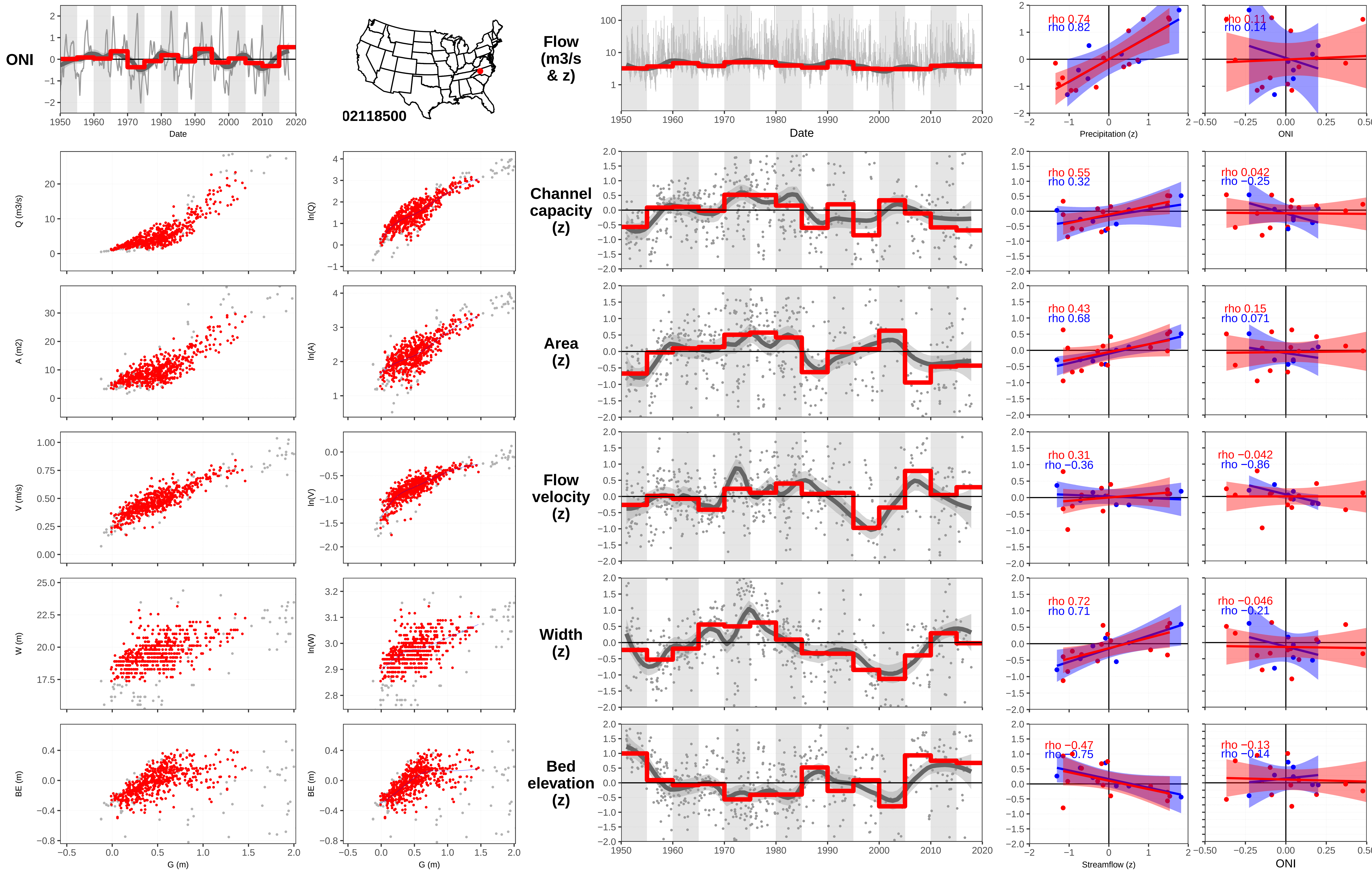
ONI
02118500
Flow (m3/s & z)
Channel capacity (z)
Area (z)
Flow velocity (z)
Width (z)
Bed elevation (z)
Date
Precipitation (z)
Streamflow (z)
ONI
G (m)
Q (m3/s)
ln(Q)
A (m2)
ln(A)
V (m/s)
ln(V)
W (m)
ln(W)
BE (m)
rho 0.74
rho 0.82
rho 0.11
rho 0.14
rho 0.55
rho 0.32
rho 0.042
rho −0.25
rho 0.43
rho 0.68
rho 0.15
rho 0.071
rho 0.31
rho −0.36
rho −0.042
rho −0.86
rho 0.72
rho 0.71
rho −0.046
rho −0.21
rho −0.47
rho −0.75
rho −0.13
rho −0.14

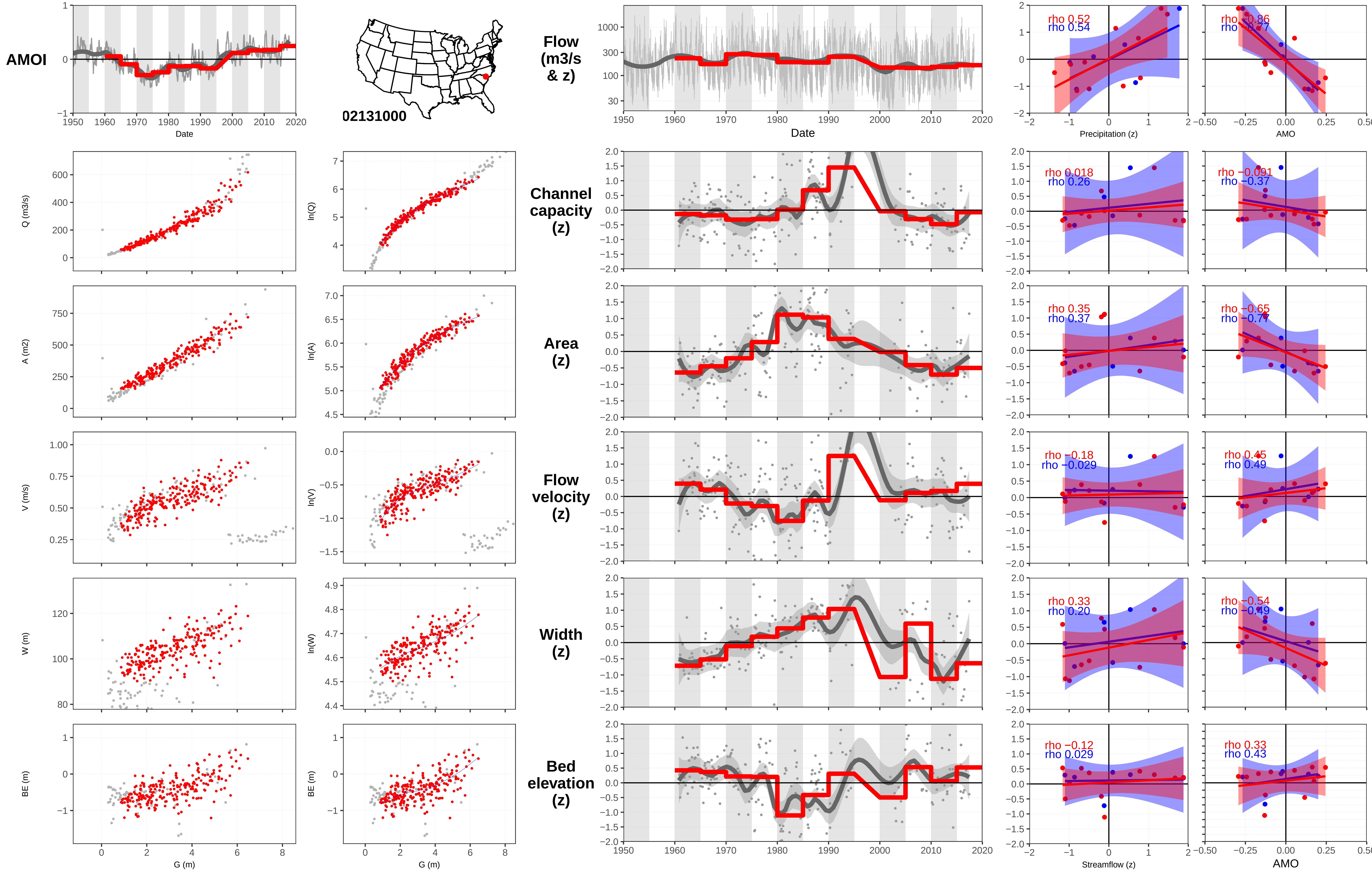

AMOI
02131000
Flow (m3/s & z)
Channel capacity (z)
Area (z)
Flow velocity (z)
Width (z)
Bed elevation (z)
Date
Precipitation (z)
AMO
Streamflow (z)
Q (m3/s)
ln(Q)
A (m2)
ln(A)
V (m/s)
ln(V)
W (m)
ln(W)
BE (m)
G (m)
rho 0.52
rho 0.54
rho −0.86
rho −0.77
rho 0.018
rho 0.26
rho −0.091
rho −0.37
rho 0.35
rho 0.37
rho −0.65
rho −0.77
rho −0.18
rho −0.029
rho 0.45
rho 0.49
rho 0.33
rho 0.20
rho −0.54
rho −0.49
rho −0.12
rho 0.029
rho 0.33
rho 0.43

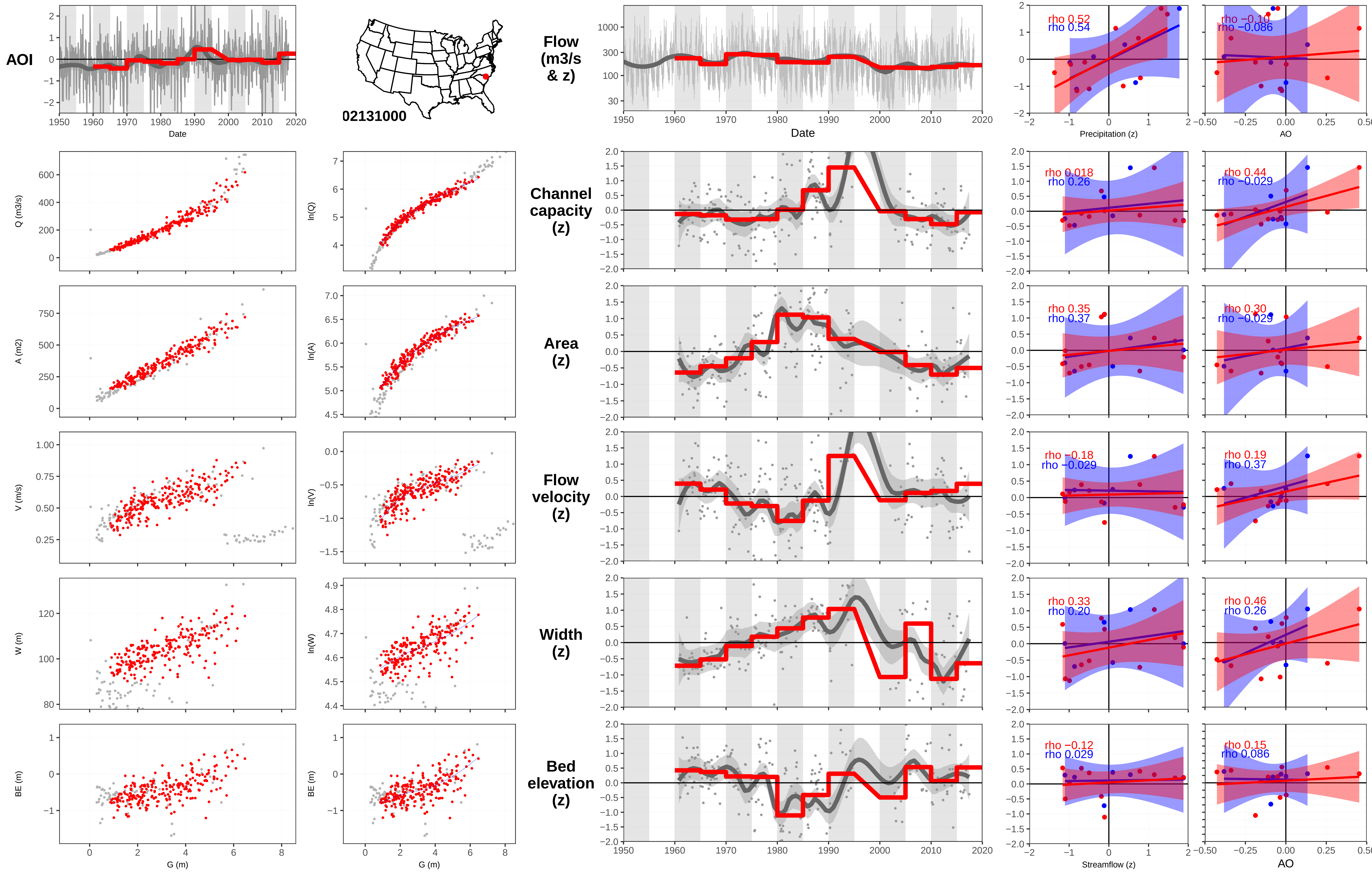

AOI
Date
02131000
Flow (m3/s & z)
Date
Precipitation (z)
AO
rho 0.52
rho 0.54
rho −0.10
rho −0.086
Q (m3/s)
ln(Q)
Channel capacity (z)
rho 0.018
rho 0.26
rho 0.44
rho −0.029
A (m2)
ln(A)
Area (z)
rho 0.35
rho 0.37
rho 0.30
rho −0.029
V (m/s)
ln(V)
Flow velocity (z)
rho −0.18
rho −0.029
rho 0.19
rho 0.37
W (m)
ln(W)
Width (z)
rho 0.33
rho 0.20
rho 0.46
rho 0.26
BE (m)
BE (m)
Bed elevation (z)
rho −0.12
rho 0.029
rho 0.15
rho 0.086
G (m)
G (m)
Streamflow (z)
AO

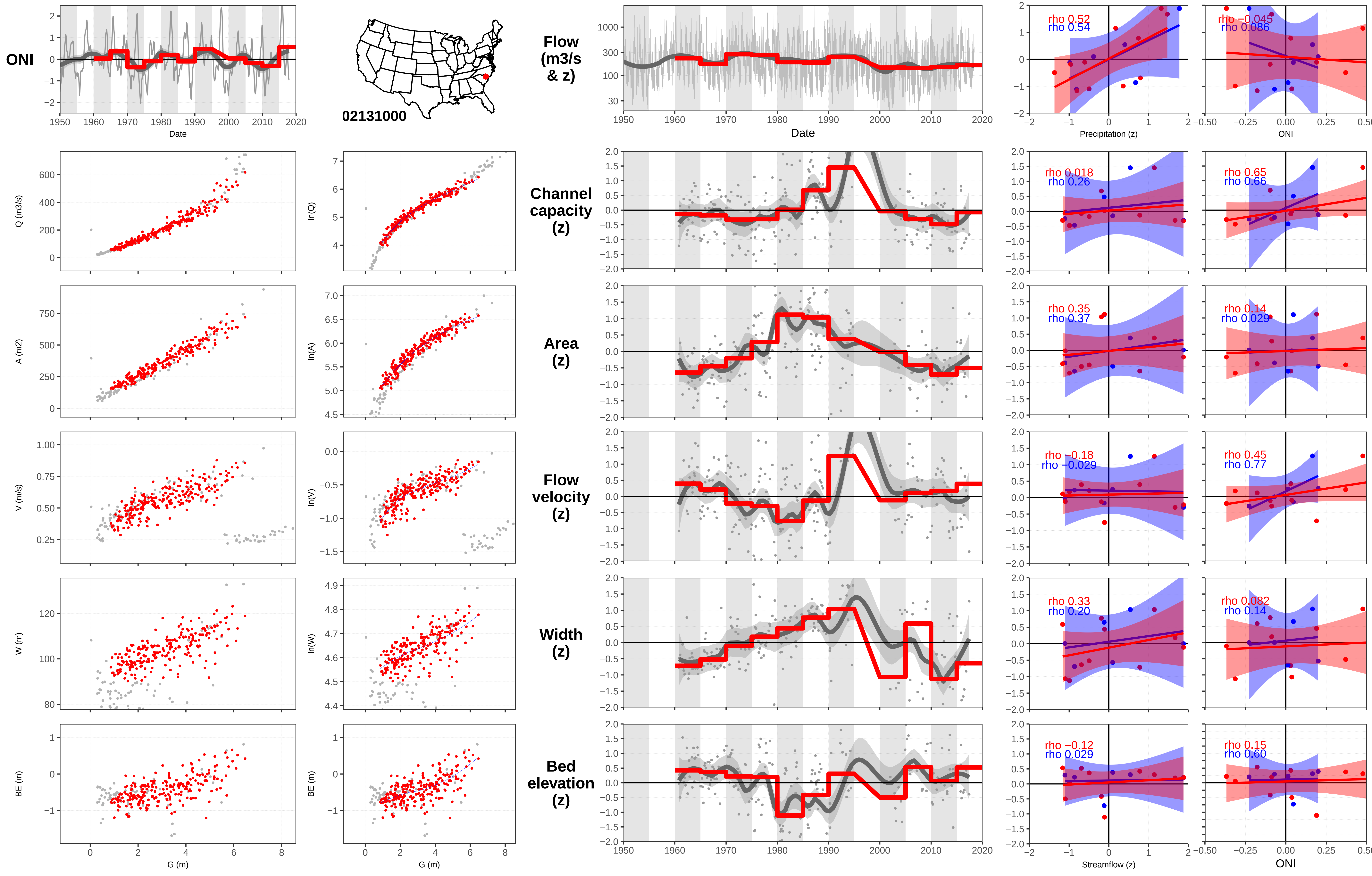

ONI
02131000
Flow (m3/s & z)
Channel capacity (z)
Area (z)
Flow velocity (z)
Width (z)
Bed elevation (z)
Date
Precipitation (z)
Streamflow (z)
ONI
G (m)
Q (m3/s)
ln(Q)
A (m2)
ln(A)
V (m/s)
ln(V)
W (m)
ln(W)
BE (m)
rho 0.52
rho 0.54
rho −0.045
rho 0.086
rho 0.018
rho 0.26
rho 0.65
rho 0.66
rho 0.35
rho 0.37
rho 0.14
rho 0.029
rho −0.18
rho −0.029
rho 0.45
rho 0.77
rho 0.33
rho 0.20
rho 0.082
rho 0.14
rho −0.12
rho 0.029
rho 0.15
rho 0.60

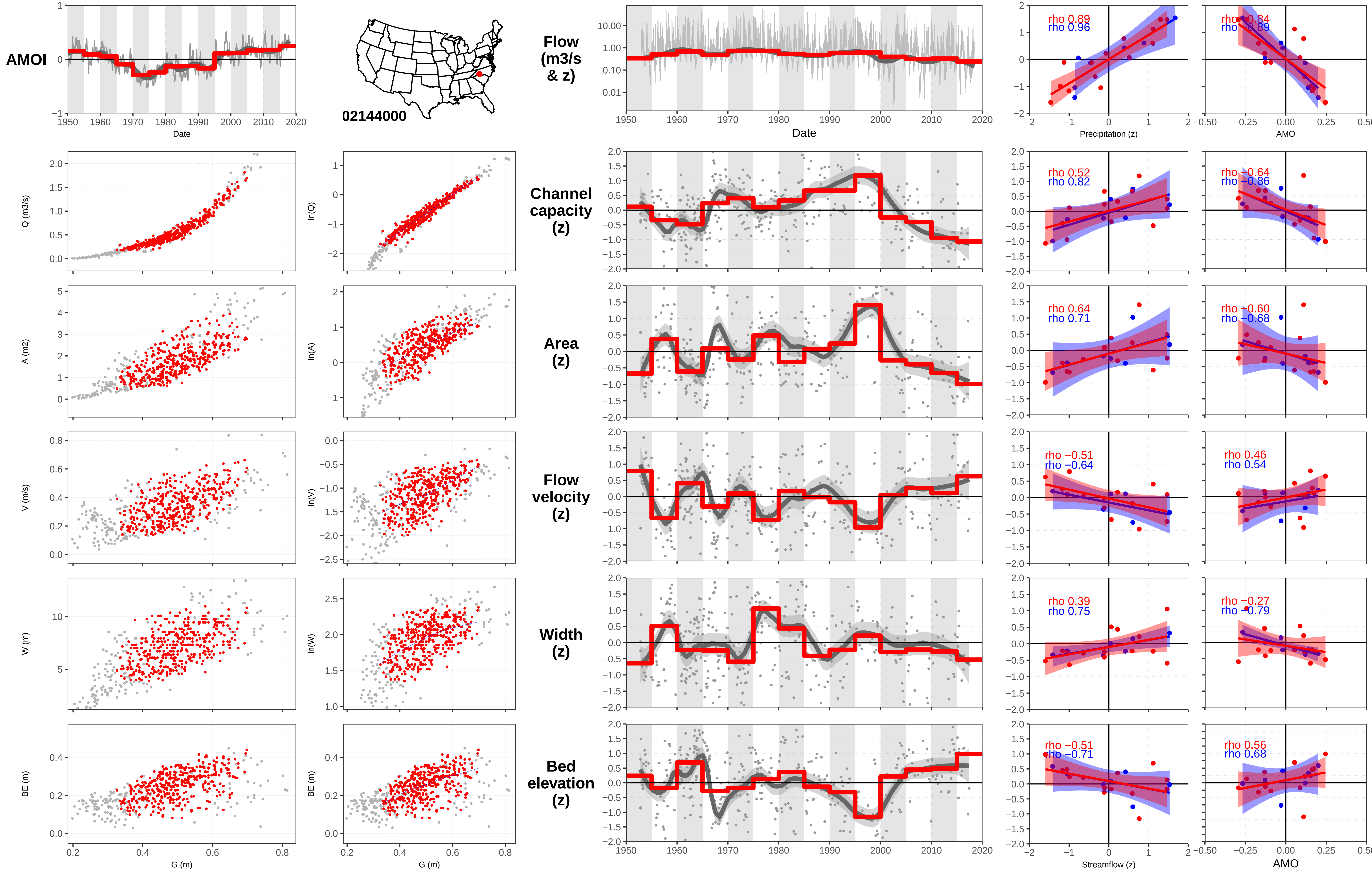

AMOI
02144000
Flow (m3/s & z)
Channel capacity (z)
Area (z)
Flow velocity (z)
Width (z)
Bed elevation (z)
Date
Precipitation (z)
AMO
Streamflow (z)
G (m)
Q (m3/s)
ln(Q)
A (m2)
ln(A)
V (m/s)
ln(V)
W (m)
ln(W)
BE (m)
rho 0.89
rho 0.96
rho −0.84
rho −0.89
rho 0.52
rho 0.82
rho −0.64
rho −0.86
rho 0.64
rho 0.71
rho −0.60
rho −0.68
rho −0.51
rho −0.64
rho 0.46
rho 0.54
rho 0.39
rho 0.75
rho −0.27
rho −0.79
rho −0.51
rho −0.71
rho 0.56
rho 0.68

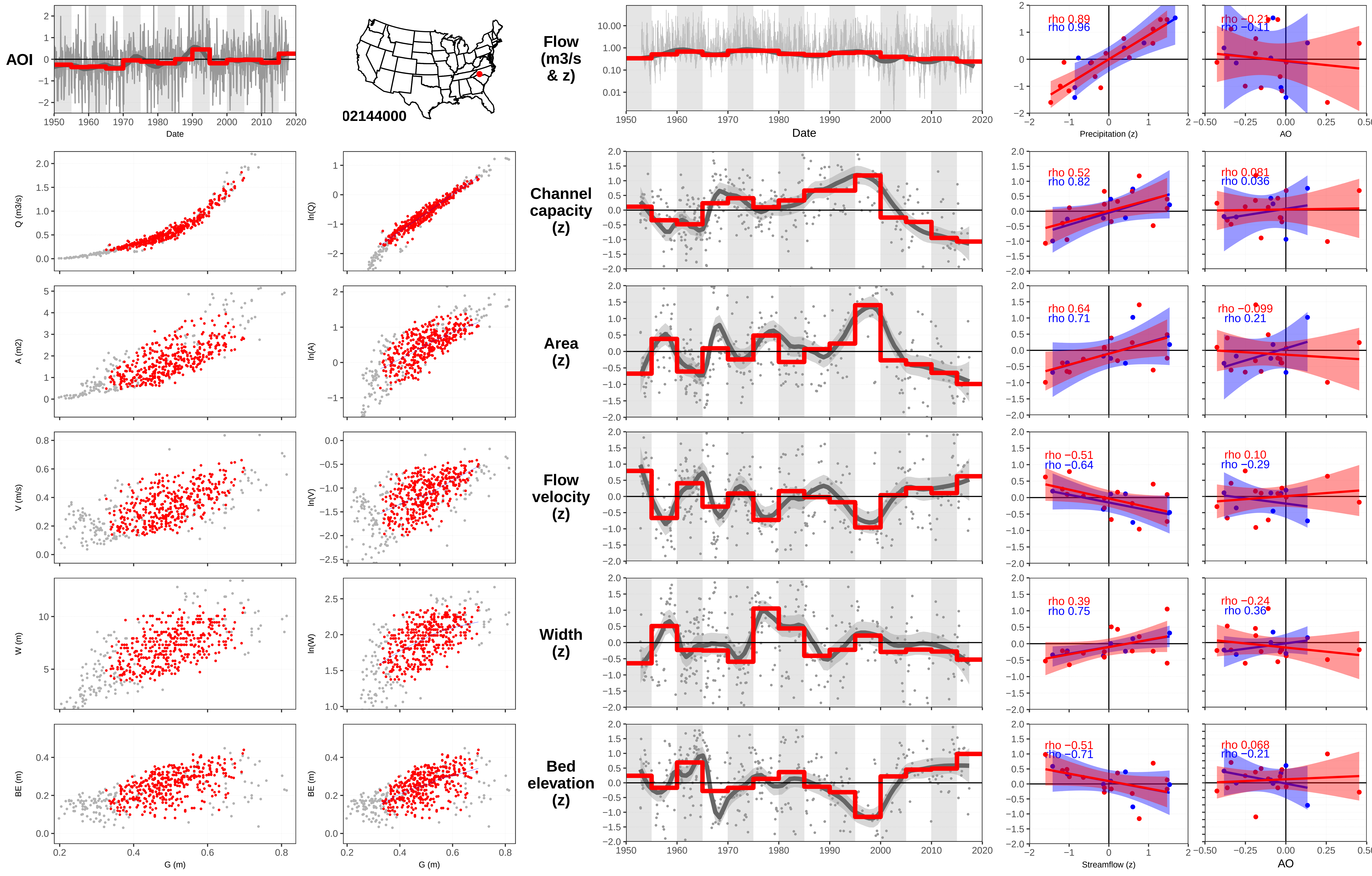

AOI
02144000
Flow (m3/s & z)
Channel capacity (z)
Area (z)
Flow velocity (z)
Width (z)
Bed elevation (z)
Date
Precipitation (z)
Streamflow (z)
AO
G (m)
Q (m3/s)
ln(Q)
A (m2)
ln(A)
V (m/s)
ln(V)
W (m)
ln(W)
BE (m)
rho 0.89
rho 0.96
rho −0.21
rho −0.11
rho 0.52
rho 0.82
rho 0.081
rho 0.036
rho 0.64
rho 0.71
rho −0.099
rho 0.21
rho −0.51
rho −0.64
rho 0.10
rho −0.29
rho 0.39
rho 0.75
rho −0.24
rho 0.36
rho −0.51
rho −0.71
rho 0.068
rho −0.21

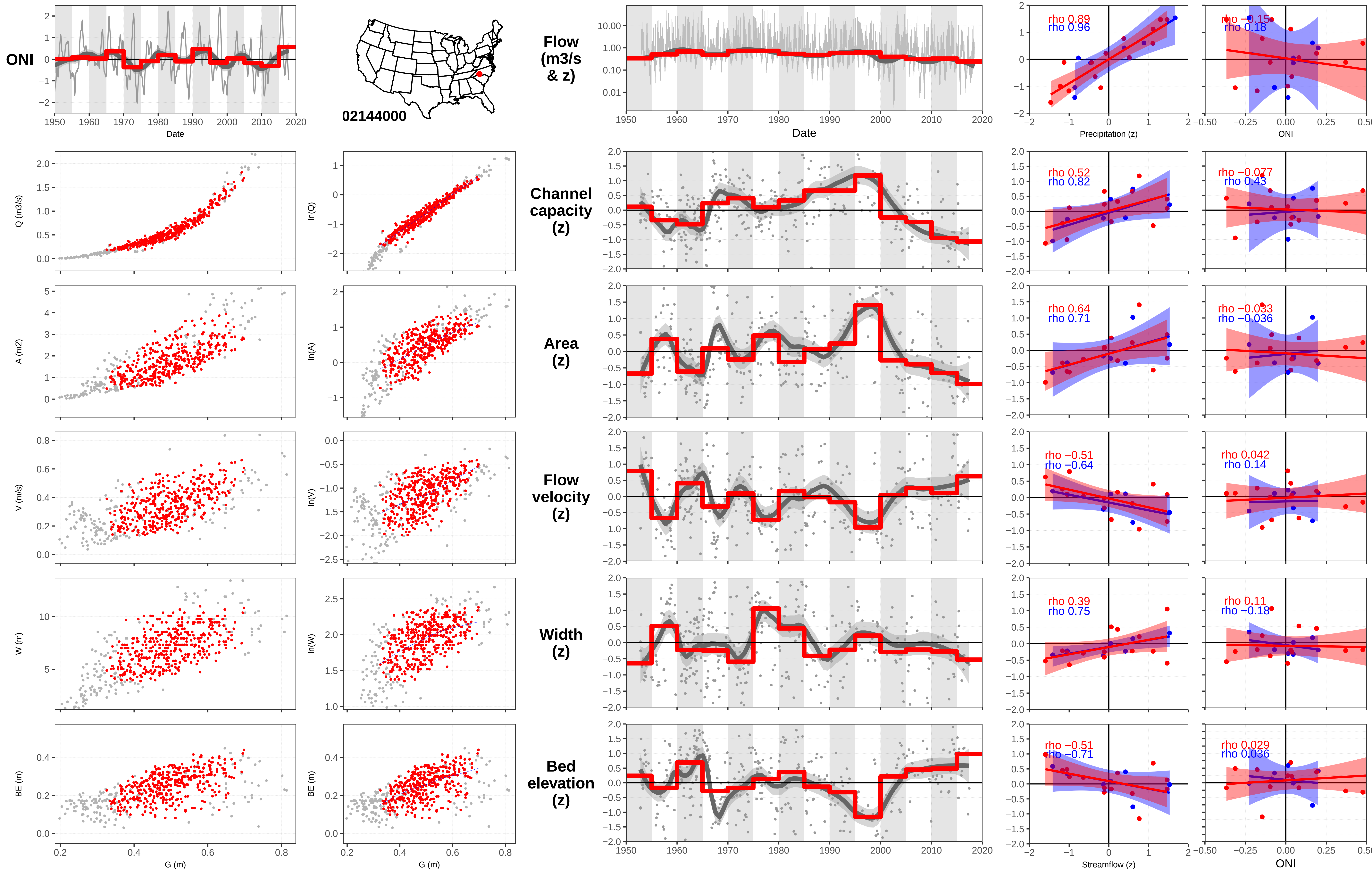

ONI
02144000
Flow (m3/s & z)
Date
Precipitation (z)
ONI
Channel capacity (z)
Area (z)
Flow velocity (z)
Width (z)
Bed elevation (z)
Q (m3/s)
ln(Q)
A (m2)
ln(A)
V (m/s)
ln(V)
W (m)
ln(W)
BE (m)
G (m)
Streamflow (z)
rho 0.89
rho 0.96
rho −0.15
rho 0.18
rho 0.52
rho 0.82
rho −0.077
rho 0.43
rho 0.64
rho 0.71
rho −0.033
rho −0.036
rho −0.51
rho −0.64
rho 0.042
rho 0.14
rho 0.39
rho 0.75
rho 0.11
rho −0.18
rho −0.51
rho −0.71
rho 0.029
rho 0.036

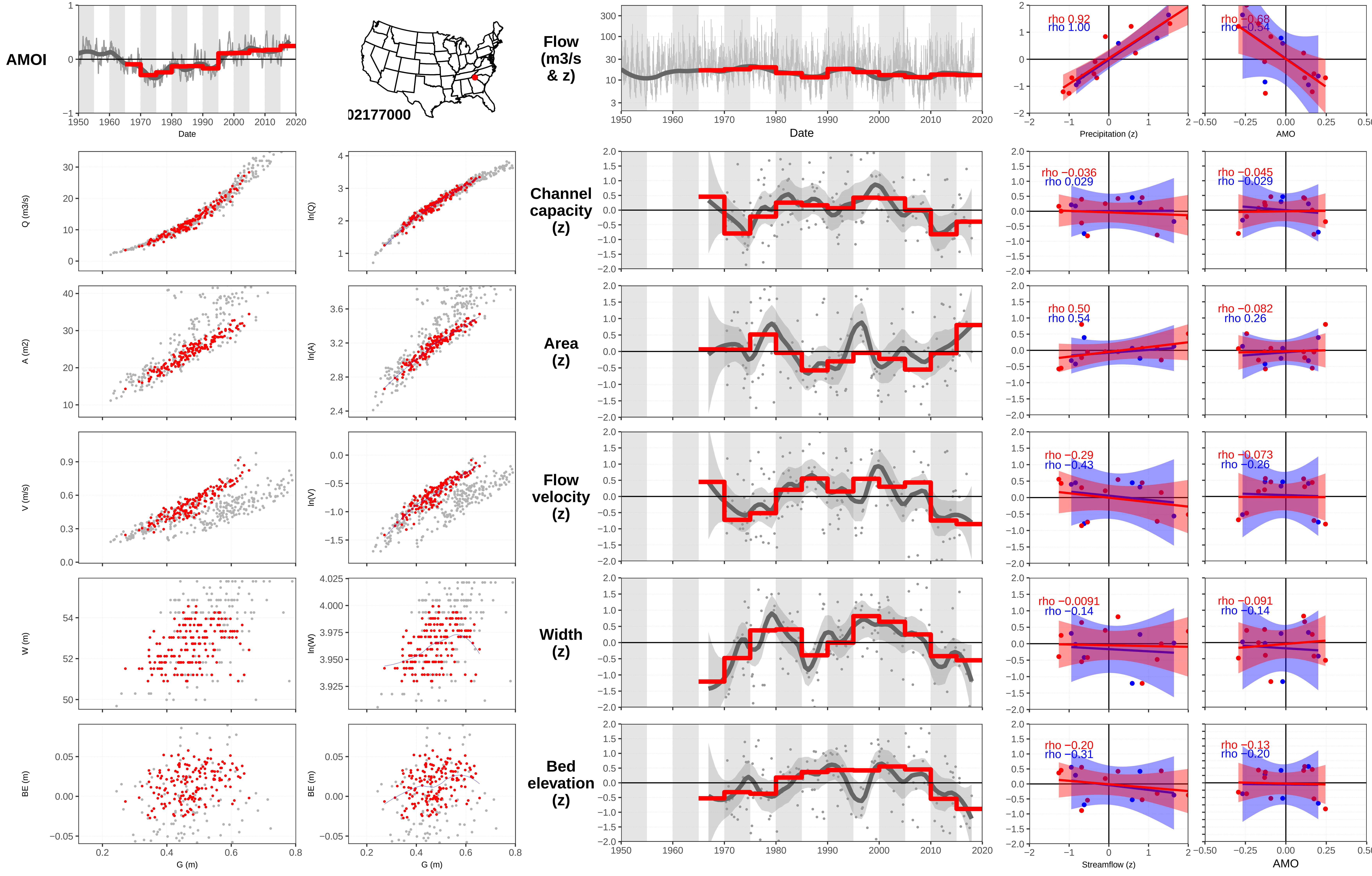

AMOI
Date
02177000
Flow (m3/s & z)
Date
rho 0.92
rho 1.00
Precipitation (z)
rho −0.68
rho −0.54
AMO
Q (m3/s)
ln(Q)
Channel capacity (z)
rho −0.036
rho 0.029
rho −0.045
rho −0.029
A (m2)
ln(A)
Area (z)
rho 0.50
rho 0.54
rho −0.082
rho 0.26
V (m/s)
ln(V)
Flow velocity (z)
rho −0.29
rho −0.43
rho −0.073
rho −0.26
W (m)
ln(W)
Width (z)
rho −0.0091
rho −0.14
rho −0.091
rho −0.14
BE (m)
BE (m)
Bed elevation (z)
rho −0.20
rho −0.31
rho −0.13
rho −0.20
G (m)
G (m)
Streamflow (z)
AMO

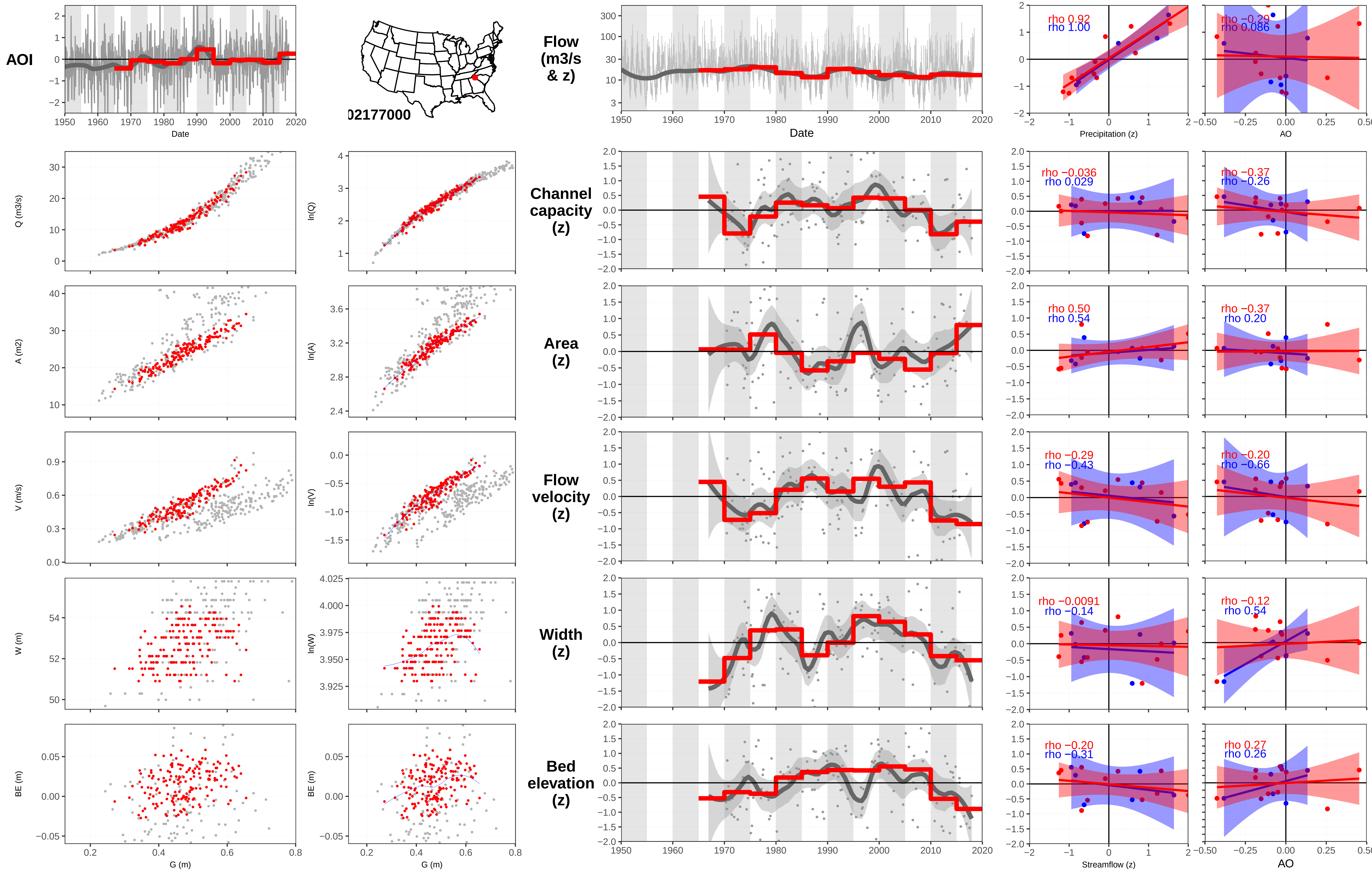
AOI
02177000
Flow (m3/s & z)
Channel capacity (z)
Area (z)
Flow velocity (z)
Width (z)
Bed elevation (z)
Date
Precipitation (z)
Streamflow (z)
AO
G (m)
Q (m3/s)
ln(Q)
A (m2)
ln(A)
V (m/s)
ln(V)
W (m)
ln(W)
BE (m)
rho 0.92
rho 1.00
rho −0.29
rho 0.086
rho −0.036
rho 0.029
rho −0.37
rho −0.26
rho 0.50
rho 0.54
rho −0.37
rho 0.20
rho −0.29
rho −0.43
rho −0.20
rho −0.66
rho −0.0091
rho −0.14
rho −0.12
rho 0.54
rho −0.20
rho −0.31
rho 0.27
rho 0.26

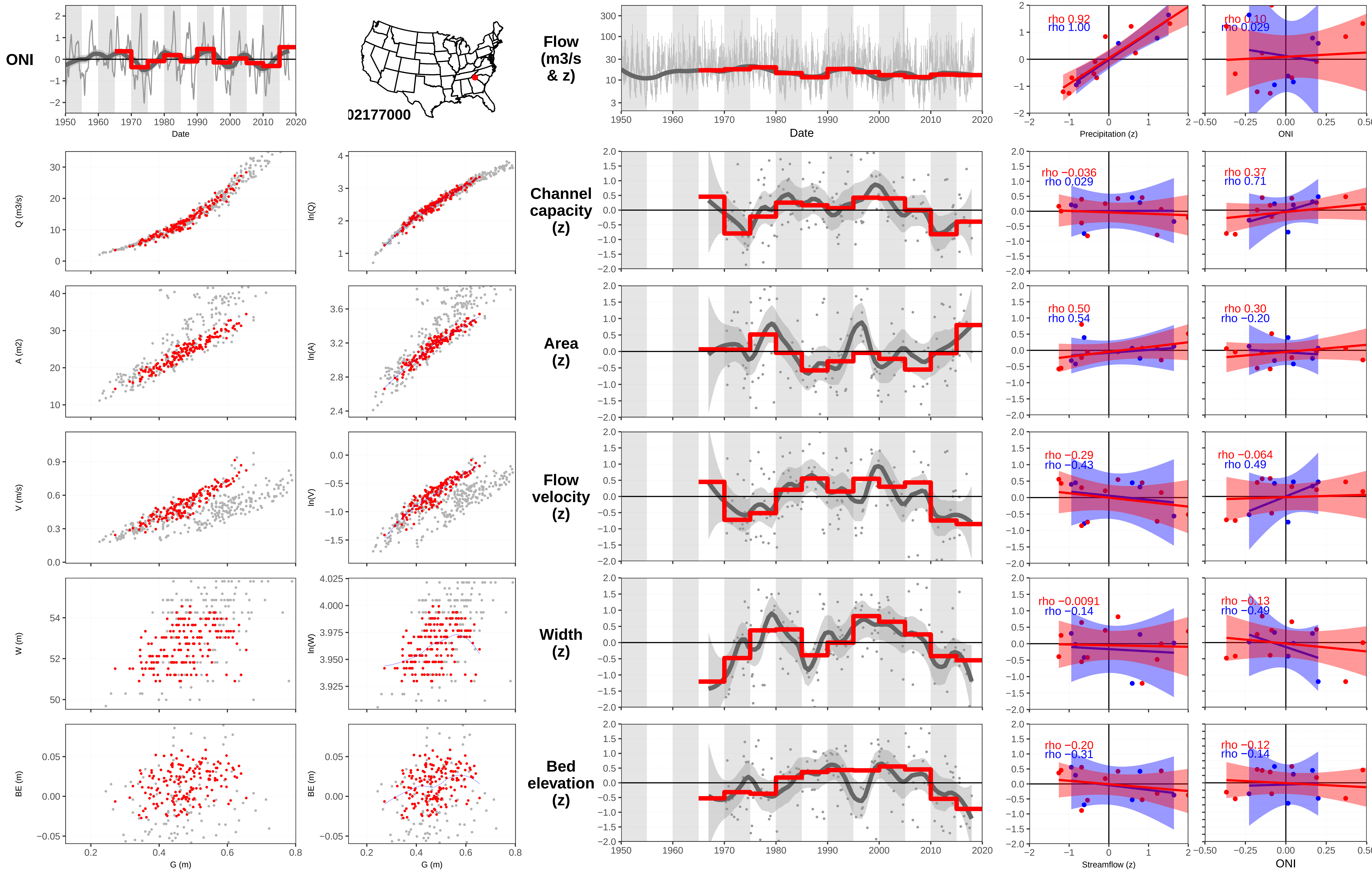

ONI
02177000
Flow (m3/s & z)
Channel capacity (z)
Area (z)
Flow velocity (z)
Width (z)
Bed elevation (z)
Date
Precipitation (z)
Streamflow (z)
ONI
G (m)
Q (m3/s)
ln(Q)
A (m2)
ln(A)
V (m/s)
ln(V)
W (m)
ln(W)
BE (m)
rho 0.92
rho 1.00
rho 0.10
rho 0.029
rho −0.036
rho 0.029
rho 0.37
rho 0.71
rho 0.50
rho 0.54
rho 0.30
rho −0.20
rho −0.29
rho −0.43
rho −0.064
rho 0.49
rho −0.0091
rho −0.14
rho −0.13
rho −0.49
rho −0.20
rho −0.31
rho −0.12
rho −0.14

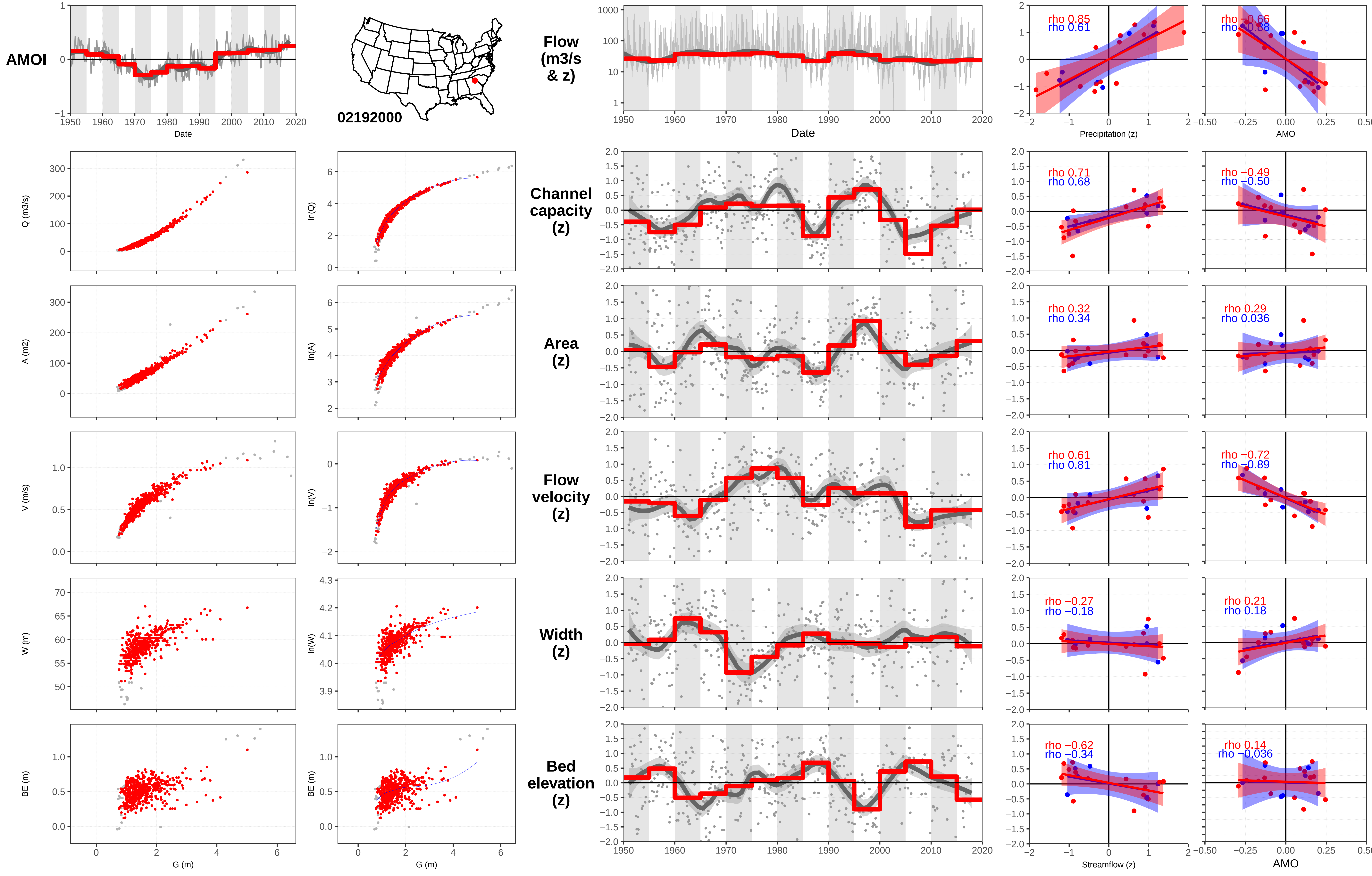

AMOI
02192000
Flow (m3/s & z)
Channel capacity (z)
Area (z)
Flow velocity (z)
Width (z)
Bed elevation (z)
Date
Precipitation (z)
AMO
Streamflow (z)
G (m)
Q (m3/s)
ln(Q)
A (m2)
ln(A)
V (m/s)
ln(V)
W (m)
ln(W)
BE (m)
rho 0.85
rho 0.61
rho −0.66
rho −0.88
rho 0.71
rho 0.68
rho −0.49
rho −0.50
rho 0.32
rho 0.34
rho 0.29
rho 0.036
rho 0.61
rho 0.81
rho −0.72
rho −0.89
rho −0.27
rho −0.18
rho 0.21
rho 0.18
rho −0.62
rho −0.34
rho 0.14
rho −0.036

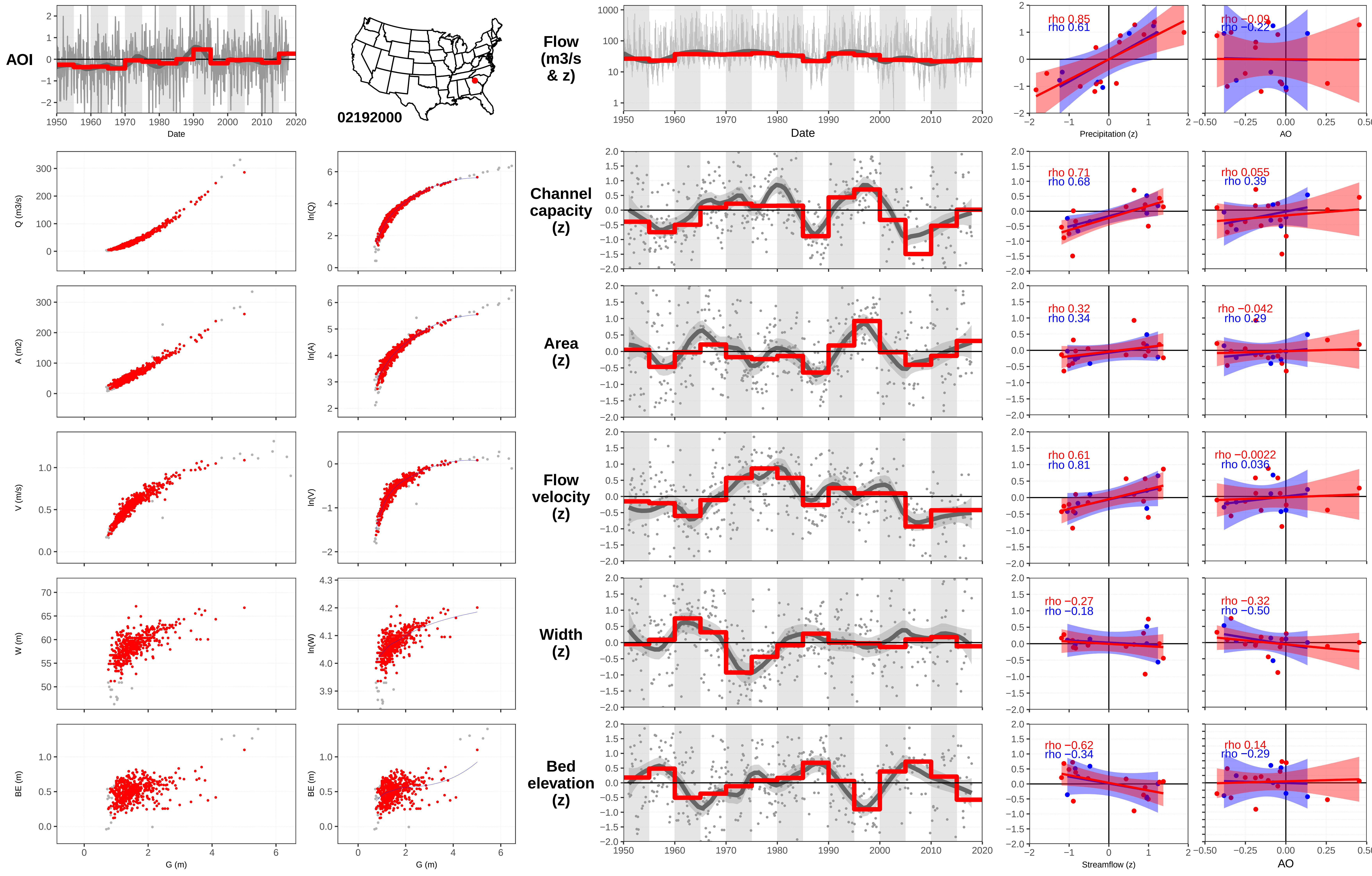

AOI
02192000
Flow (m3/s & z)
Date
rho 0.85
rho 0.61
Precipitation (z)
rho −0.09
rho −0.22
AO
Channel capacity (z)
Q (m3/s)
ln(Q)
rho 0.71
rho 0.68
rho 0.055
rho 0.39
Area (z)
A (m2)
ln(A)
rho 0.32
rho 0.34
rho −0.042
rho 0.29
Flow velocity (z)
V (m/s)
ln(V)
rho 0.61
rho 0.81
rho −0.0022
rho 0.036
Width (z)
W (m)
ln(W)
rho −0.27
rho −0.18
rho −0.32
rho −0.50
Bed elevation (z)
BE (m)
rho −0.62
rho −0.34
rho 0.14
rho −0.29
G (m)
Streamflow (z)

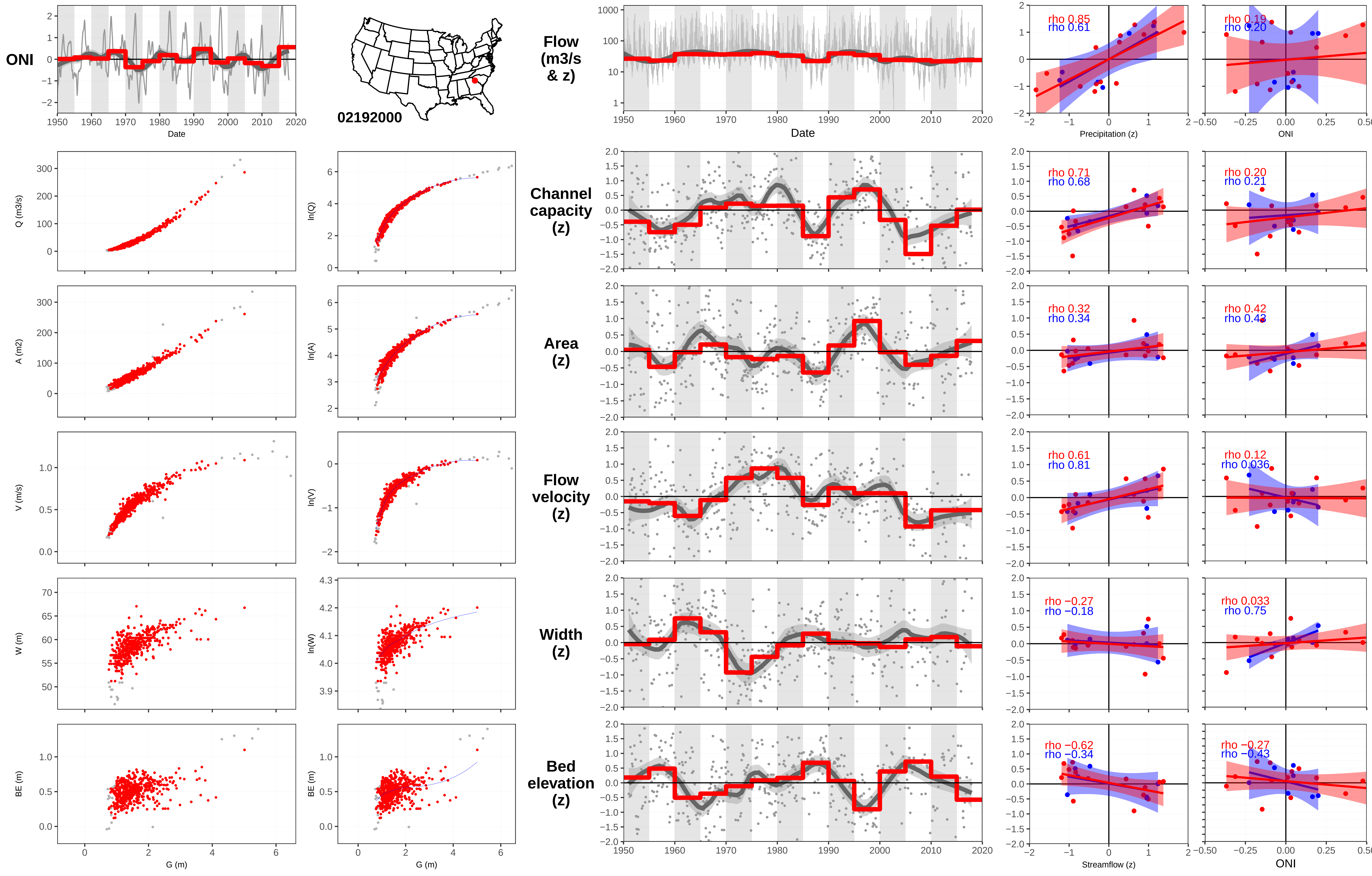

ONI
02192000
Flow (m3/s & z)
Date
Precipitation (z)
ONI
Channel capacity (z)
Area (z)
Flow velocity (z)
Width (z)
Bed elevation (z)
Q (m3/s)
ln(Q)
A (m2)
ln(A)
V (m/s)
ln(V)
W (m)
ln(W)
BE (m)
G (m)
Streamflow (z)
rho 0.85
rho 0.61
rho 0.19
rho 0.20
rho 0.71
rho 0.68
rho 0.20
rho 0.21
rho 0.32
rho 0.34
rho 0.42
rho 0.43
rho 0.61
rho 0.81
rho 0.12
rho 0.036
rho −0.27
rho −0.18
rho 0.033
rho 0.75
rho −0.62
rho −0.34
rho −0.27
rho −0.43

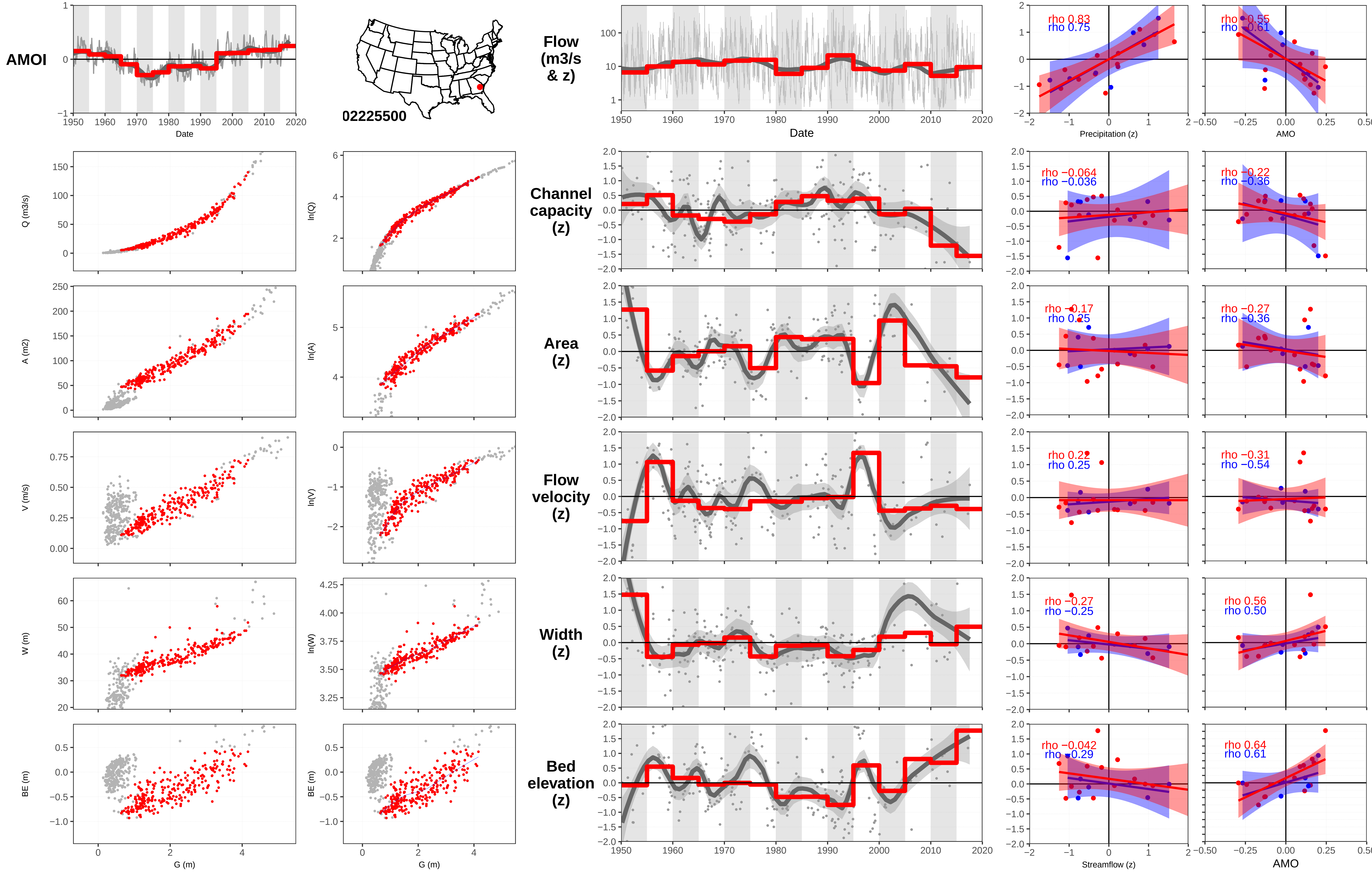

AMOI
02225500
Flow (m3/s & z)
Channel capacity (z)
Area (z)
Flow velocity (z)
Width (z)
Bed elevation (z)
Date
Precipitation (z)
AMO
Streamflow (z)
G (m)
Q (m3/s)
ln(Q)
A (m2)
ln(A)
V (m/s)
ln(V)
W (m)
ln(W)
BE (m)
rho 0.83
rho 0.75
rho −0.55
rho −0.61
rho −0.064
rho −0.036
rho −0.22
rho −0.36
rho −0.17
rho 0.25
rho −0.27
rho −0.36
rho 0.22
rho 0.25
rho −0.31
rho −0.54
rho −0.27
rho −0.25
rho 0.56
rho 0.50
rho −0.042
rho −0.29
rho 0.64
rho 0.61

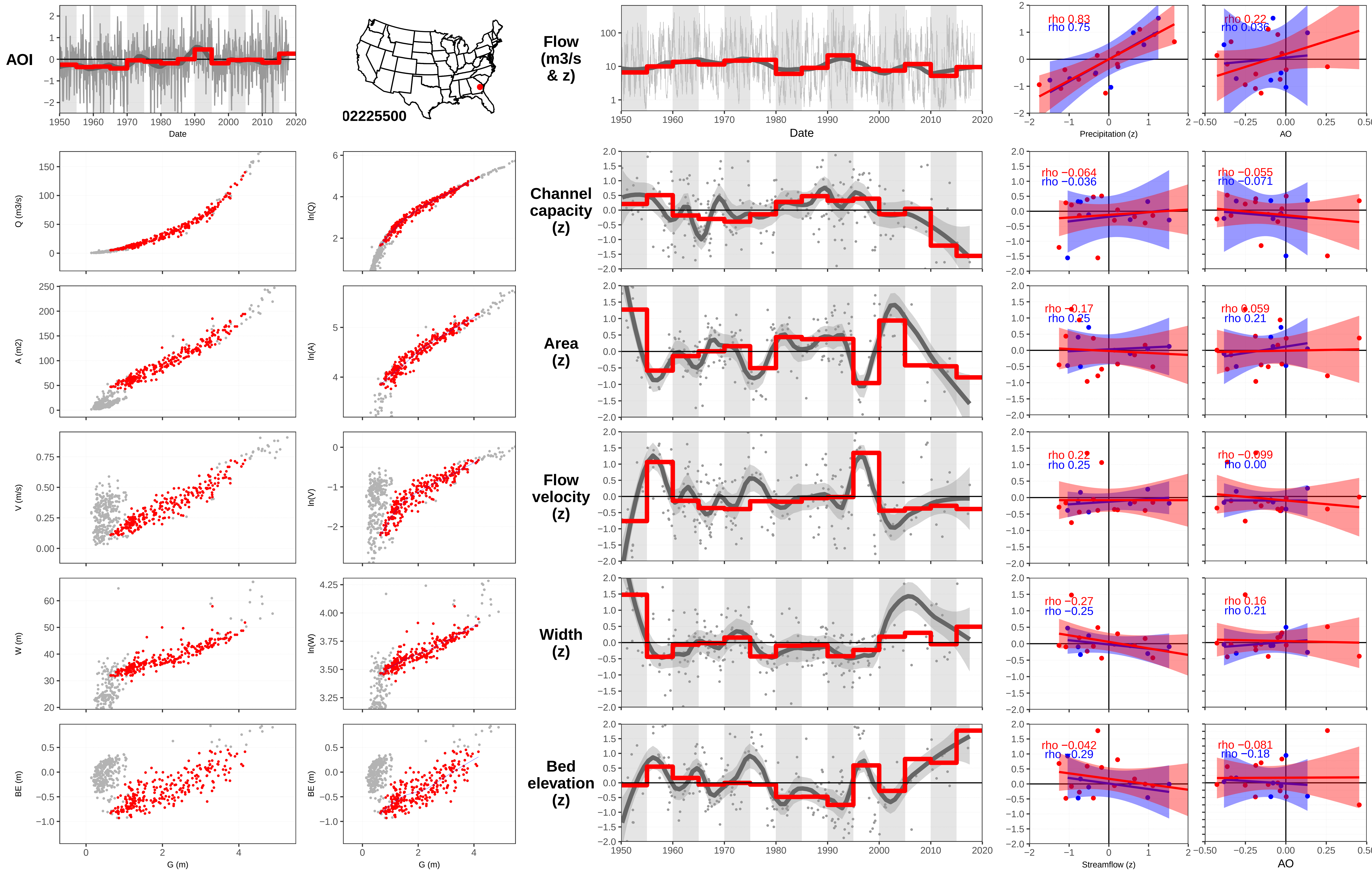
AOI
02225500
Flow (m3/s & z)
Channel capacity (z)
Area (z)
Flow velocity (z)
Width (z)
Bed elevation (z)
Date
Precipitation (z)
AO
Streamflow (z)
Q (m3/s)
ln(Q)
A (m2)
ln(A)
V (m/s)
ln(V)
W (m)
ln(W)
BE (m)
G (m)
rho 0.83
rho 0.75
rho 0.22
rho 0.036
rho −0.064
rho −0.036
rho −0.055
rho −0.071
rho −0.17
rho 0.25
rho 0.059
rho 0.21
rho 0.22
rho 0.25
rho −0.099
rho 0.00
rho −0.27
rho −0.25
rho 0.16
rho 0.21
rho −0.042
rho −0.29
rho −0.081
rho −0.18

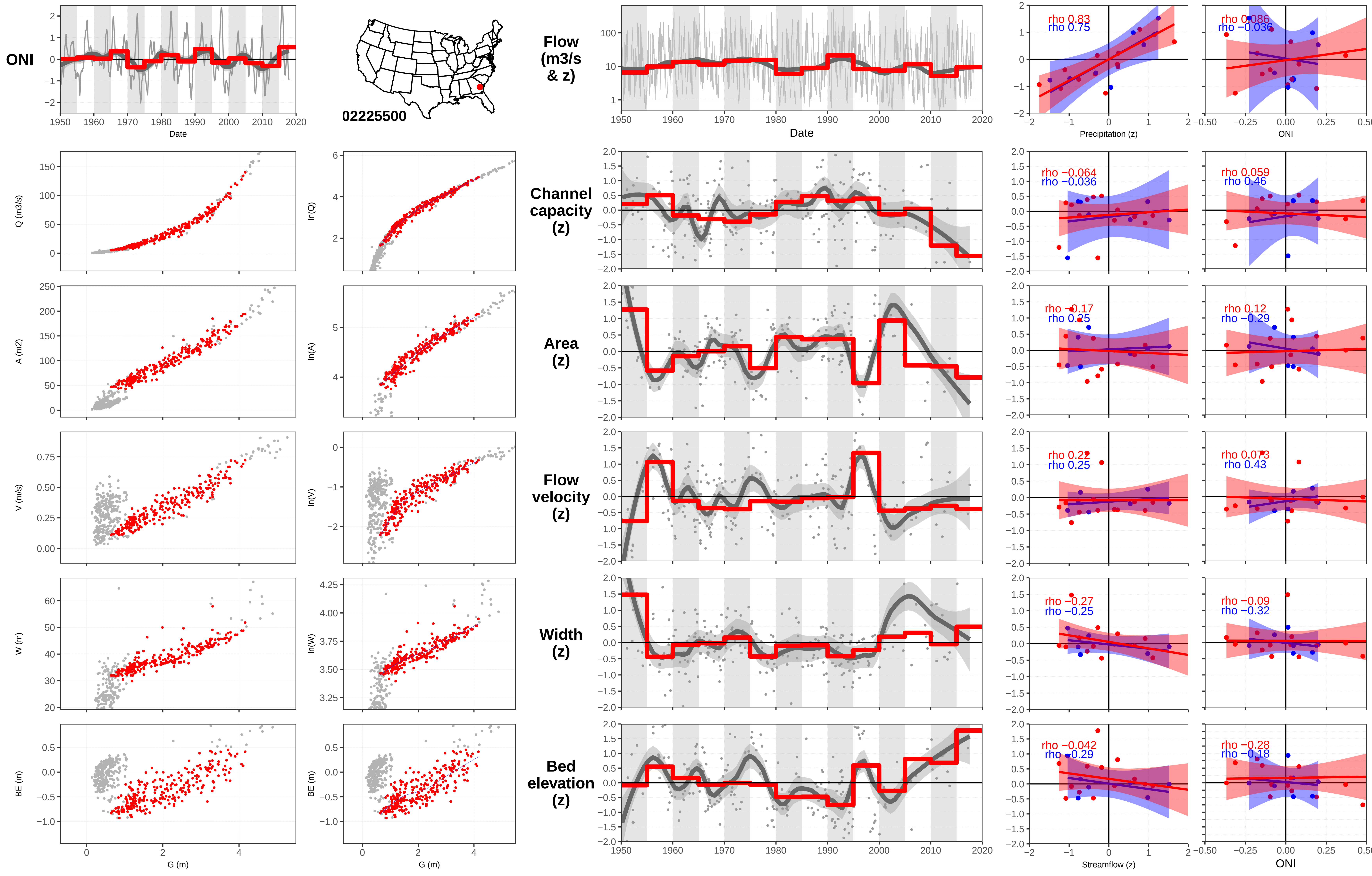

ONI
02225500
Flow (m3/s & z)
Channel capacity (z)
Area (z)
Flow velocity (z)
Width (z)
Bed elevation (z)
Date
Precipitation (z)
Streamflow (z)
ONI
Q (m3/s)
ln(Q)
A (m2)
ln(A)
V (m/s)
ln(V)
W (m)
ln(W)
BE (m)
G (m)
rho 0.83
rho 0.75
rho 0.086
rho −0.036
rho −0.064
rho −0.036
rho 0.059
rho 0.46
rho −0.17
rho 0.25
rho 0.12
rho −0.29
rho 0.22
rho 0.25
rho 0.073
rho 0.43
rho −0.27
rho −0.25
rho −0.09
rho −0.32
rho −0.042
rho −0.29
rho −0.28
rho −0.18

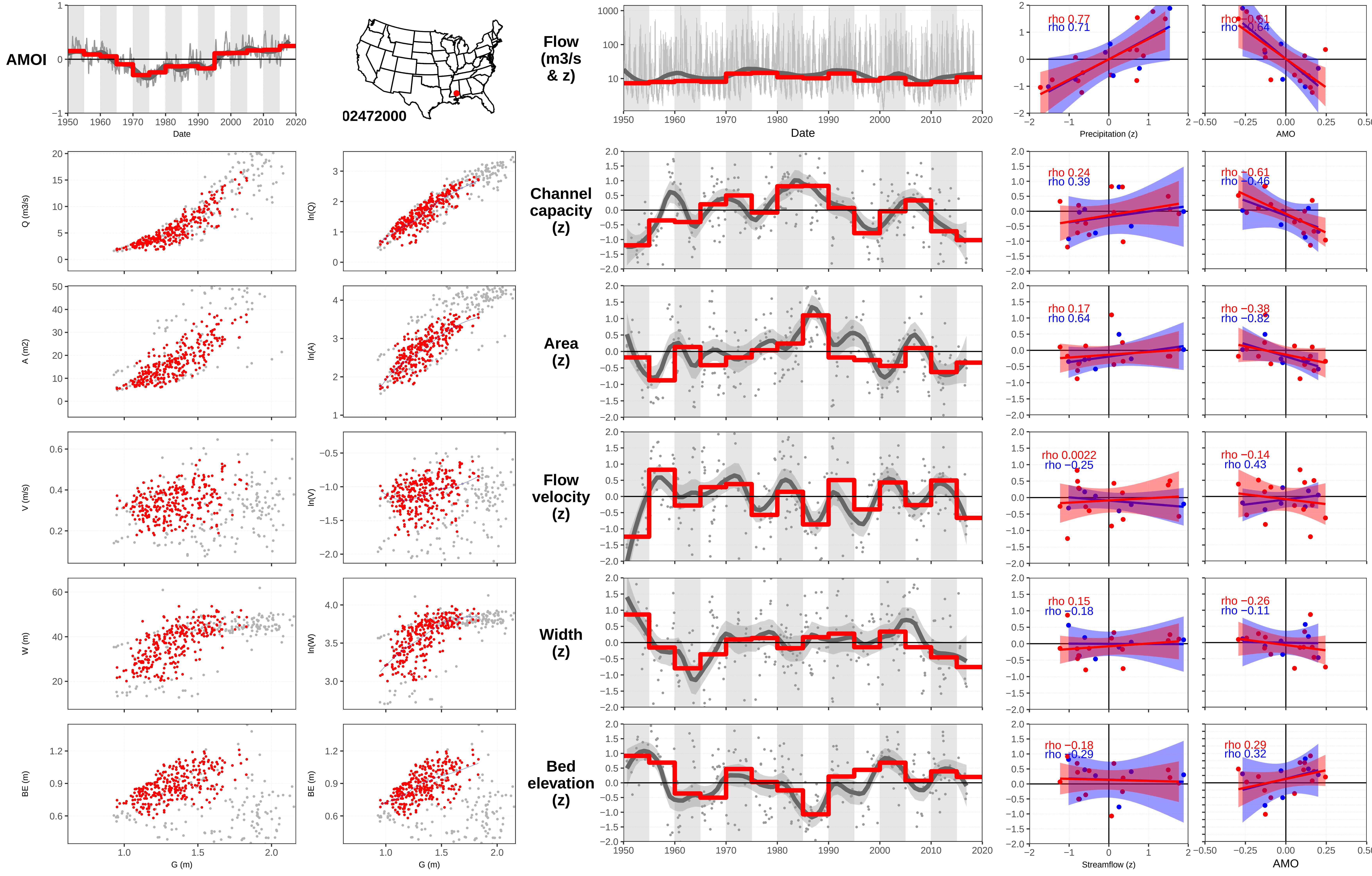
AMOI
02472000
Flow (m3/s & z)
Channel capacity (z)
Area (z)
Flow velocity (z)
Width (z)
Bed elevation (z)
Date
Precipitation (z)
AMO
Streamflow (z)
Q (m3/s)
ln(Q)
A (m2)
ln(A)
V (m/s)
ln(V)
W (m)
ln(W)
BE (m)
G (m)
rho 0.77
rho 0.71
rho −0.61
rho −0.64
rho 0.24
rho 0.39
rho −0.61
rho −0.46
rho 0.17
rho 0.64
rho −0.38
rho −0.82
rho 0.0022
rho −0.25
rho −0.14
rho 0.43
rho 0.15
rho −0.18
rho −0.26
rho −0.11
rho −0.18
rho −0.29
rho 0.29
rho 0.32

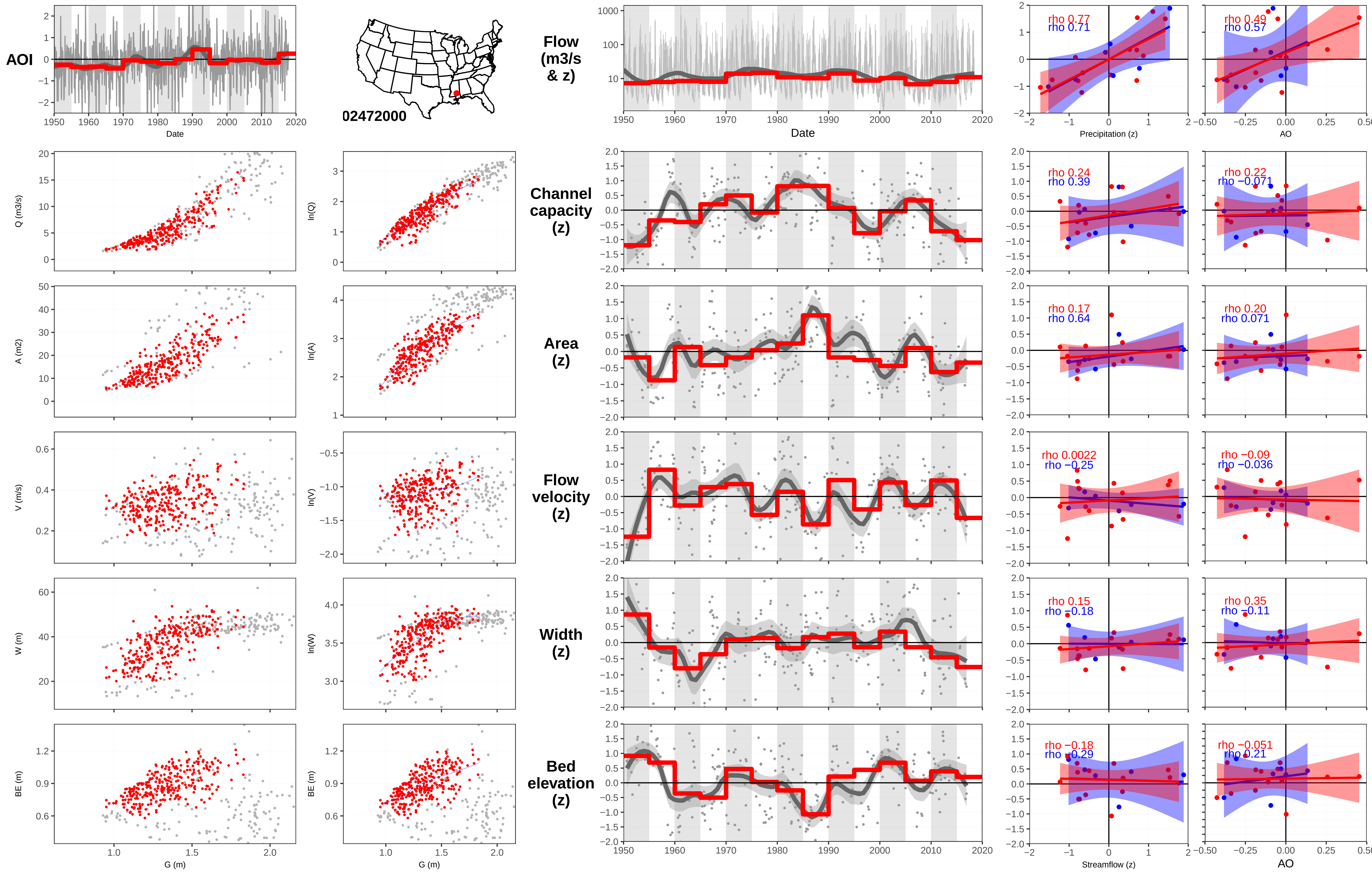
AOI
Date
02472000
Flow (m3/s & z)
Date
rho 0.77
rho 0.71
Precipitation (z)
rho 0.49
rho 0.57
AO
Q (m3/s)
ln(Q)
Channel capacity (z)
rho 0.24
rho 0.39
rho 0.22
rho −0.071
A (m2)
ln(A)
Area (z)
rho 0.17
rho 0.64
rho 0.20
rho 0.071
V (m/s)
ln(V)
Flow velocity (z)
rho 0.0022
rho −0.25
rho −0.09
rho −0.036
W (m)
ln(W)
Width (z)
rho 0.15
rho −0.18
rho 0.35
rho −0.11
BE (m)
Bed elevation (z)
rho −0.18
rho −0.29
rho −0.051
rho 0.21
G (m)
G (m)
Streamflow (z)
AO

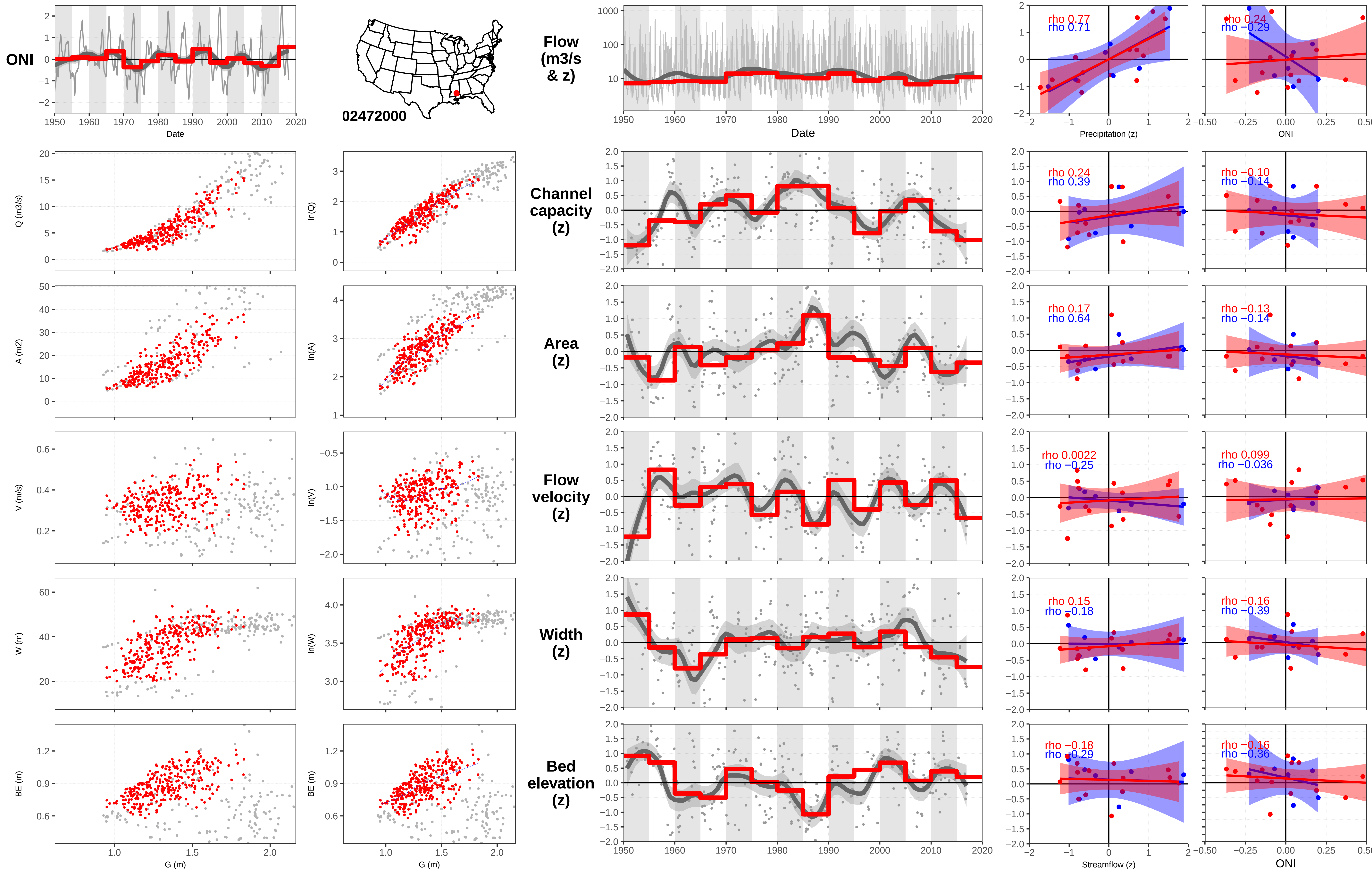

ONI
Date
02472000
Flow (m3/s & z)
Date
rho 0.77
rho 0.71
Precipitation (z)
rho 0.24
rho −0.29
ONI
Q (m3/s)
ln(Q)
Channel capacity (z)
rho 0.24
rho 0.39
rho −0.10
rho −0.14
A (m2)
ln(A)
Area (z)
rho 0.17
rho 0.64
rho −0.13
rho −0.14
V (m/s)
ln(V)
Flow velocity (z)
rho 0.0022
rho −0.25
rho 0.099
rho −0.036
W (m)
ln(W)
Width (z)
rho 0.15
rho −0.18
rho −0.16
rho −0.39
BE (m)
BE (m)
Bed elevation (z)
rho −0.18
rho −0.29
rho −0.16
rho −0.36
G (m)
G (m)
Streamflow (z)
ONI

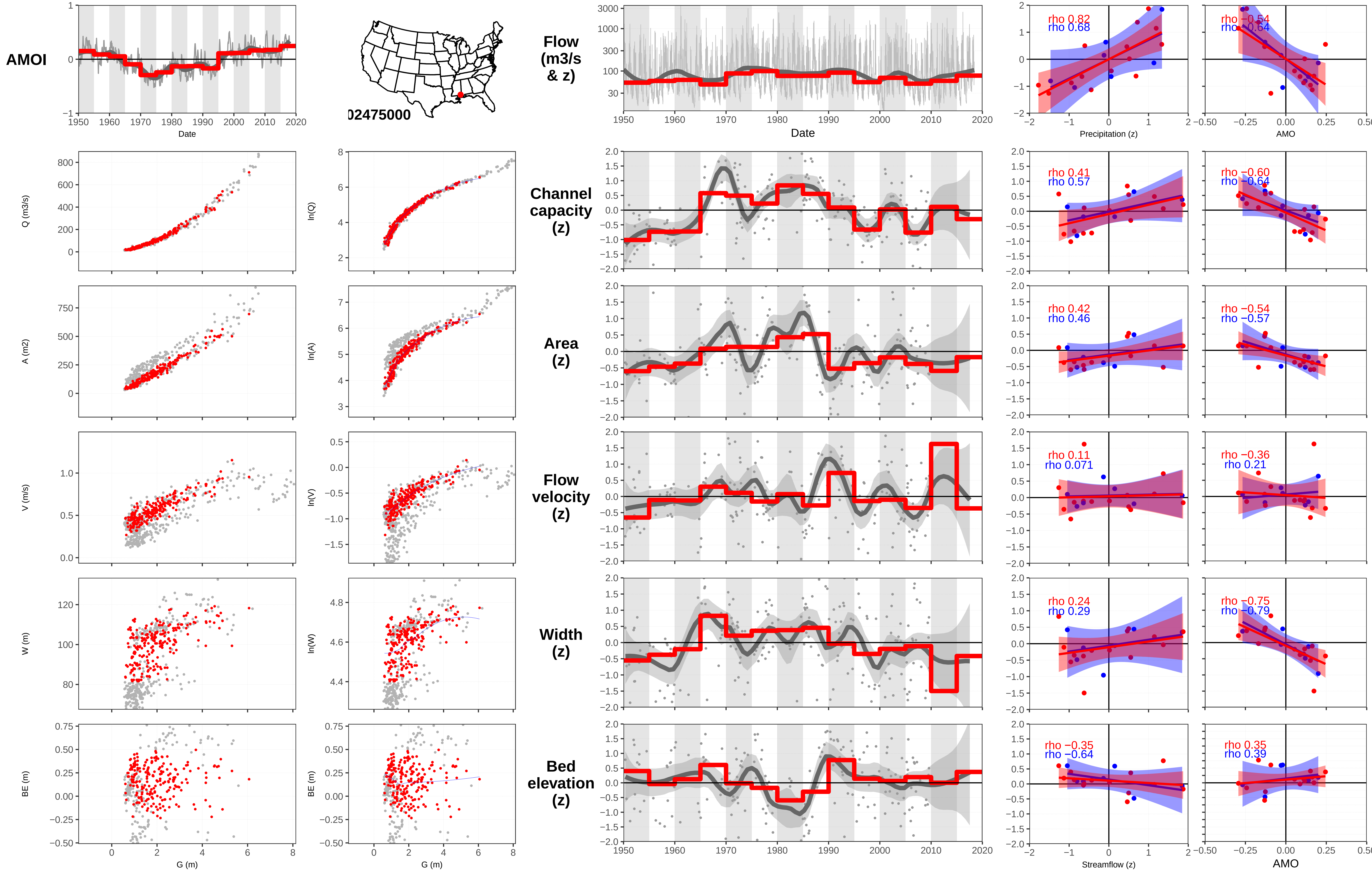

AMOI
Date
02475000
Flow (m3/s & z)
Date
Precipitation (z)
AMO
rho 0.82
rho 0.68
rho −0.54
rho −0.64
Channel capacity (z)
rho 0.41
rho 0.57
rho −0.60
rho −0.64
Q (m3/s)
ln(Q)
Area (z)
rho 0.42
rho 0.46
rho −0.54
rho −0.57
A (m2)
ln(A)
Flow velocity (z)
rho 0.11
rho 0.071
rho −0.36
rho 0.21
V (m/s)
ln(V)
Width (z)
rho 0.24
rho 0.29
rho −0.75
rho −0.79
W (m)
ln(W)
Bed elevation (z)
rho −0.35
rho −0.64
rho 0.35
rho 0.39
BE (m)
G (m)
Streamflow (z)
AMO

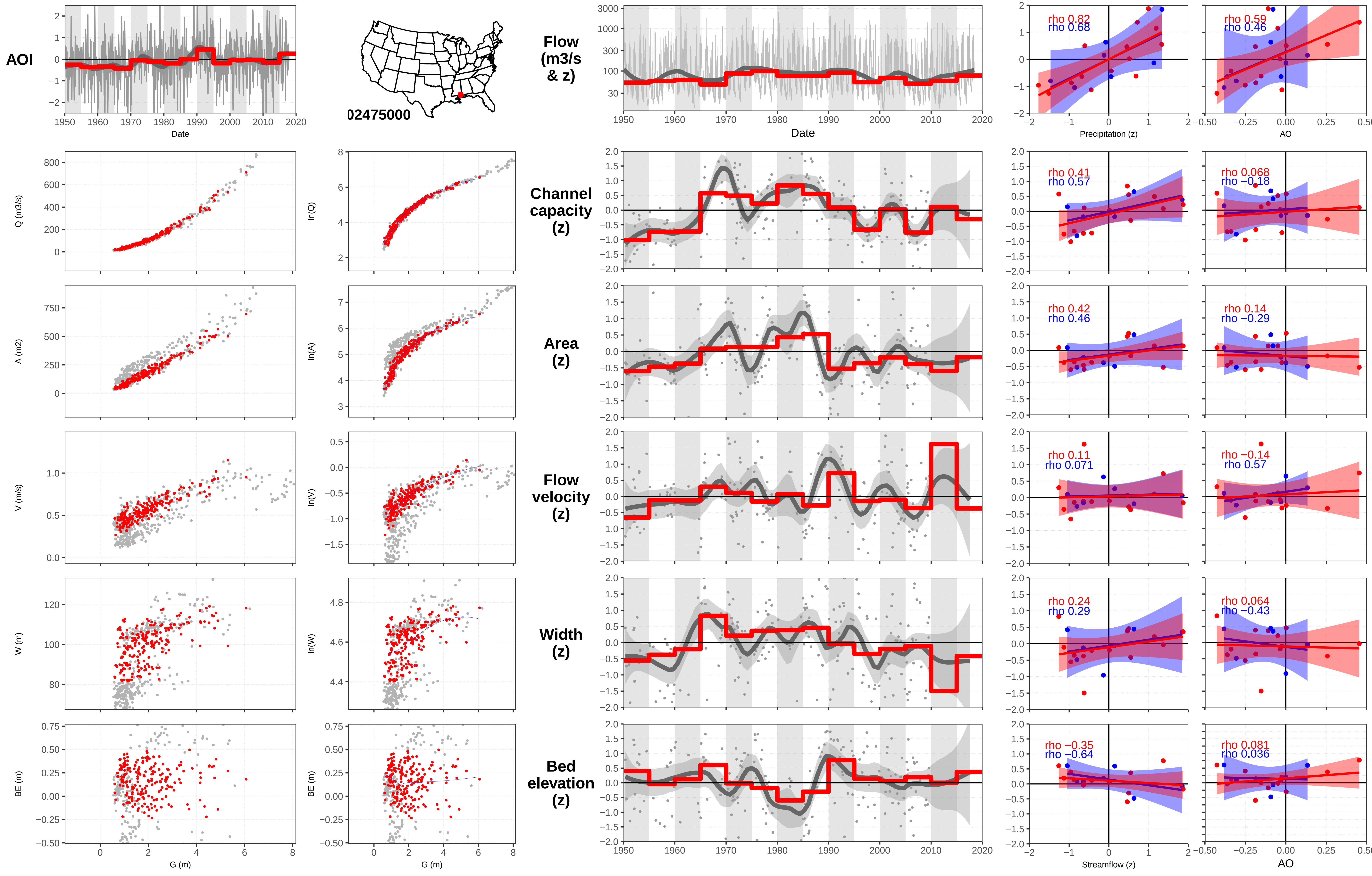

AOI
02475000
Flow (m3/s & z)
Channel capacity (z)
Area (z)
Flow velocity (z)
Width (z)
Bed elevation (z)
Date
Precipitation (z)
AO
Streamflow (z)
G (m)
Q (m3/s)
ln(Q)
A (m2)
ln(A)
V (m/s)
ln(V)
W (m)
ln(W)
BE (m)
rho 0.82
rho 0.68
rho 0.59
rho 0.46
rho 0.41
rho 0.57
rho 0.068
rho −0.18
rho 0.42
rho 0.46
rho 0.14
rho −0.29
rho 0.11
rho 0.071
rho −0.14
rho 0.57
rho 0.24
rho 0.29
rho 0.064
rho −0.43
rho −0.35
rho −0.64
rho 0.081
rho 0.036

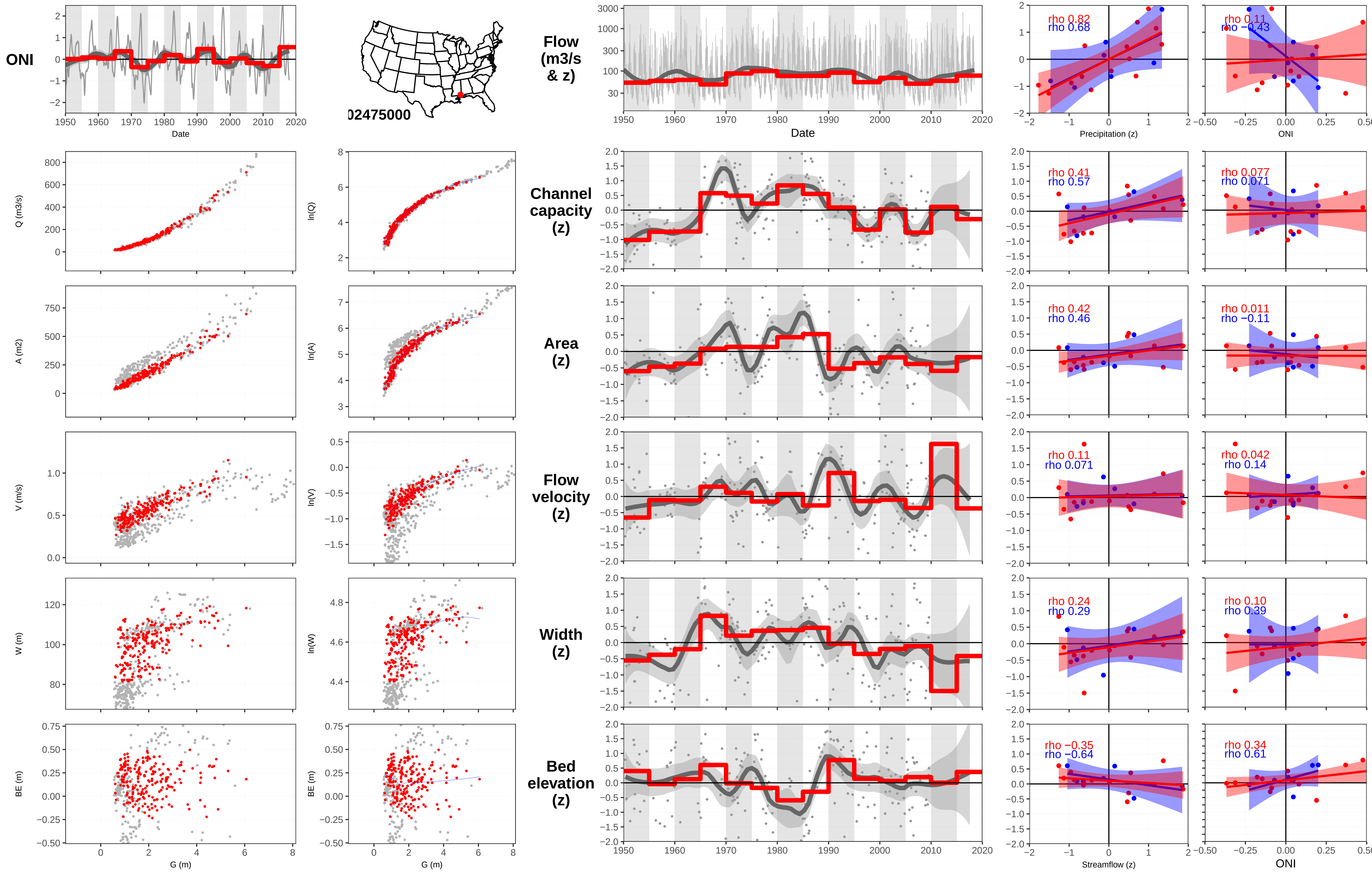

ONI
02475000
Flow (m3/s & z)
Date
Precipitation (z)
ONI
rho 0.82
rho 0.68
rho 0.11
rho −0.43
Q (m3/s)
ln(Q)
Channel capacity (z)
rho 0.41
rho 0.57
rho 0.077
rho 0.071
A (m2)
ln(A)
Area (z)
rho 0.42
rho 0.46
rho 0.011
rho −0.11
V (m/s)
ln(V)
Flow velocity (z)
rho 0.11
rho 0.071
rho 0.042
rho 0.14
W (m)
ln(W)
Width (z)
rho 0.24
rho 0.29
rho 0.10
rho 0.39
BE (m)
Bed elevation (z)
rho −0.35
rho −0.64
rho 0.34
rho 0.61
G (m)
Streamflow (z)

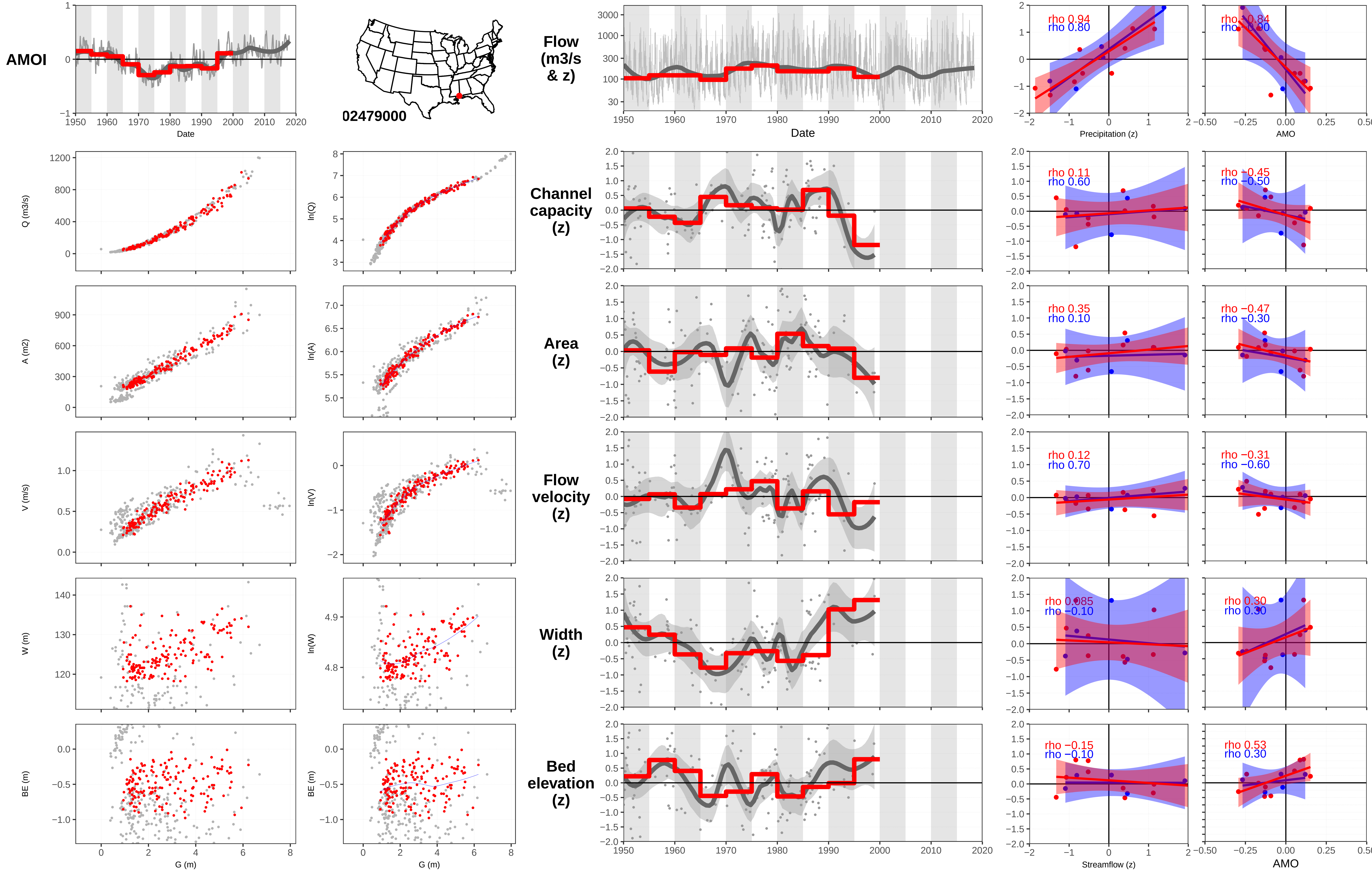

AMOI
02479000
Flow (m3/s & z)
Date
Channel capacity (z)
Area (z)
Flow velocity (z)
Width (z)
Bed elevation (z)
Q (m3/s)
ln(Q)
A (m2)
ln(A)
V (m/s)
ln(V)
W (m)
ln(W)
BE (m)
G (m)
Precipitation (z)
Streamflow (z)
AMO
rho 0.94
rho 0.80
rho −0.84
rho −0.90
rho 0.11
rho 0.60
rho −0.45
rho −0.50
rho 0.35
rho 0.10
rho −0.47
rho −0.30
rho 0.12
rho 0.70
rho −0.31
rho −0.60
rho 0.085
rho −0.10
rho 0.30
rho 0.30
rho −0.15
rho −0.10
rho 0.53
rho 0.30

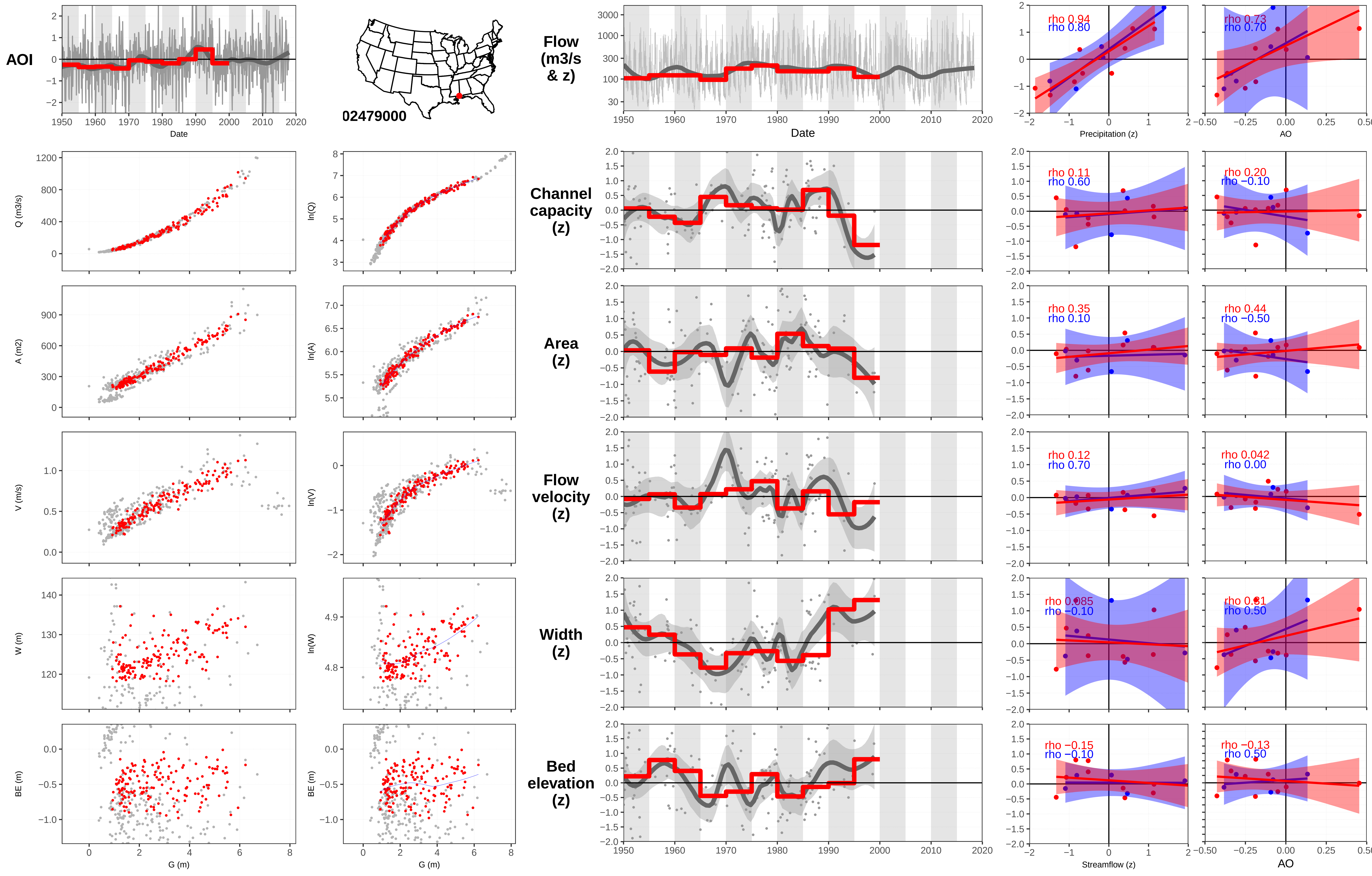

AOI
02479000
Flow (m3/s & z)
Channel capacity (z)
Area (z)
Flow velocity (z)
Width (z)
Bed elevation (z)
Date
Precipitation (z)
AO
Streamflow (z)
Q (m3/s)
ln(Q)
A (m2)
ln(A)
V (m/s)
ln(V)
W (m)
ln(W)
BE (m)
G (m)
rho 0.94
rho 0.80
rho 0.73
rho 0.70
rho 0.11
rho 0.60
rho 0.20
rho −0.10
rho 0.35
rho 0.10
rho 0.44
rho −0.50
rho 0.12
rho 0.70
rho 0.042
rho 0.00
rho 0.085
rho −0.10
rho 0.31
rho 0.50
rho −0.15
rho −0.10
rho −0.13
rho 0.50

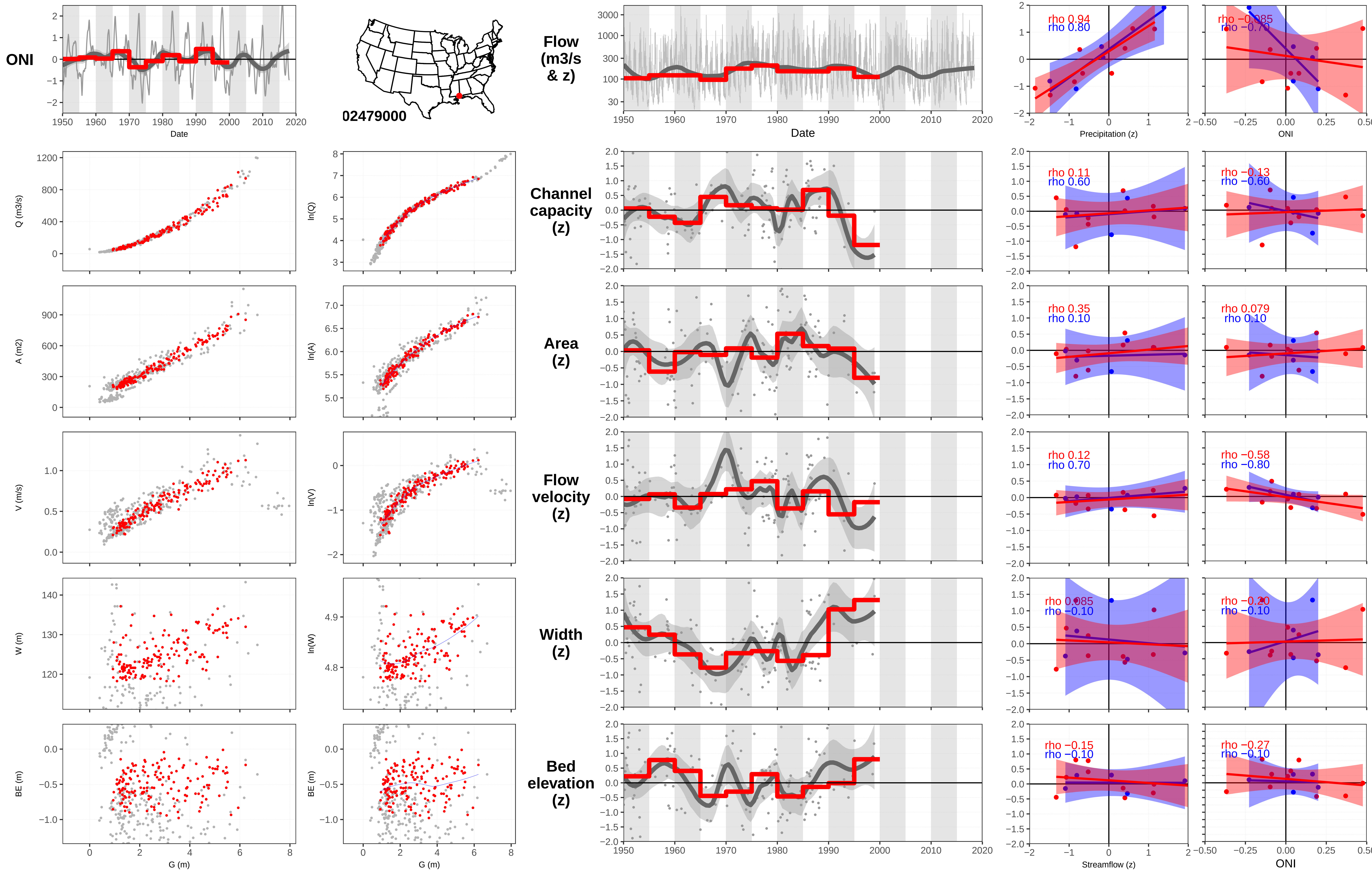

ONI
02479000
Flow (m3/s & z)
Channel capacity (z)
Area (z)
Flow velocity (z)
Width (z)
Bed elevation (z)
Date
Precipitation (z)
Streamflow (z)
ONI
G (m)
Q (m3/s)
ln(Q)
A (m2)
ln(A)
V (m/s)
ln(V)
W (m)
ln(W)
BE (m)
rho 0.94
rho 0.80
rho −0.085
rho −0.70
rho 0.11
rho 0.60
rho −0.13
rho −0.60
rho 0.35
rho 0.10
rho 0.079
rho 0.10
rho 0.12
rho 0.70
rho −0.58
rho −0.80
rho 0.085
rho −0.10
rho −0.20
rho −0.10
rho −0.15
rho −0.10
rho −0.27
rho −0.10

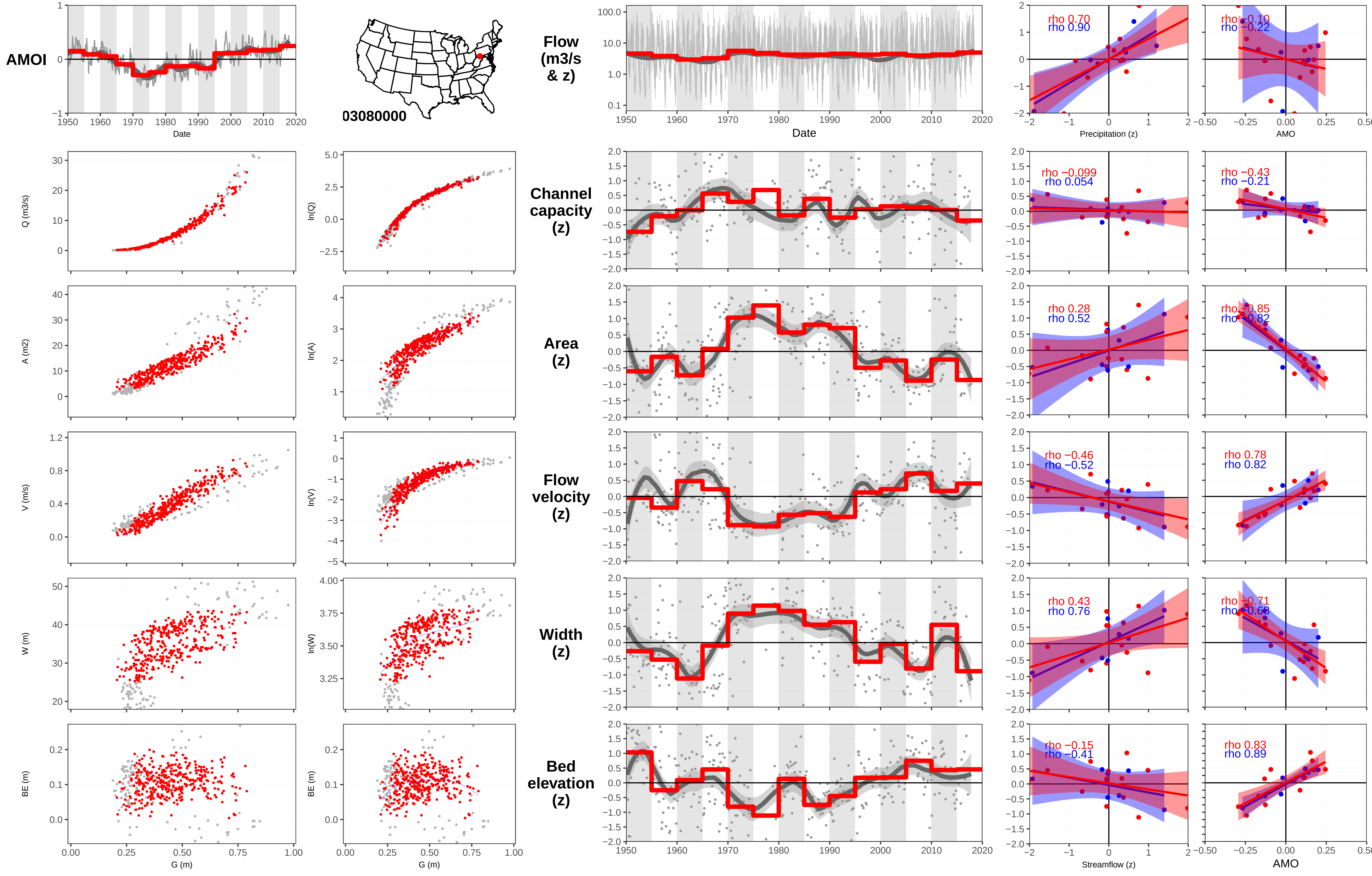

AMOI
03080000
Flow (m3/s & z)
Channel capacity (z)
Area (z)
Flow velocity (z)
Width (z)
Bed elevation (z)
Date
Precipitation (z)
AMO
Streamflow (z)
Q (m3/s)
ln(Q)
A (m2)
ln(A)
V (m/s)
ln(V)
W (m)
ln(W)
BE (m)
G (m)
rho 0.70
rho 0.90
rho −0.10
rho −0.22
rho −0.099
rho 0.054
rho −0.43
rho −0.21
rho 0.28
rho 0.52
rho −0.85
rho −0.82
rho −0.46
rho −0.52
rho 0.78
rho 0.82
rho 0.43
rho 0.76
rho −0.71
rho −0.68
rho −0.15
rho −0.41
rho 0.83
rho 0.89

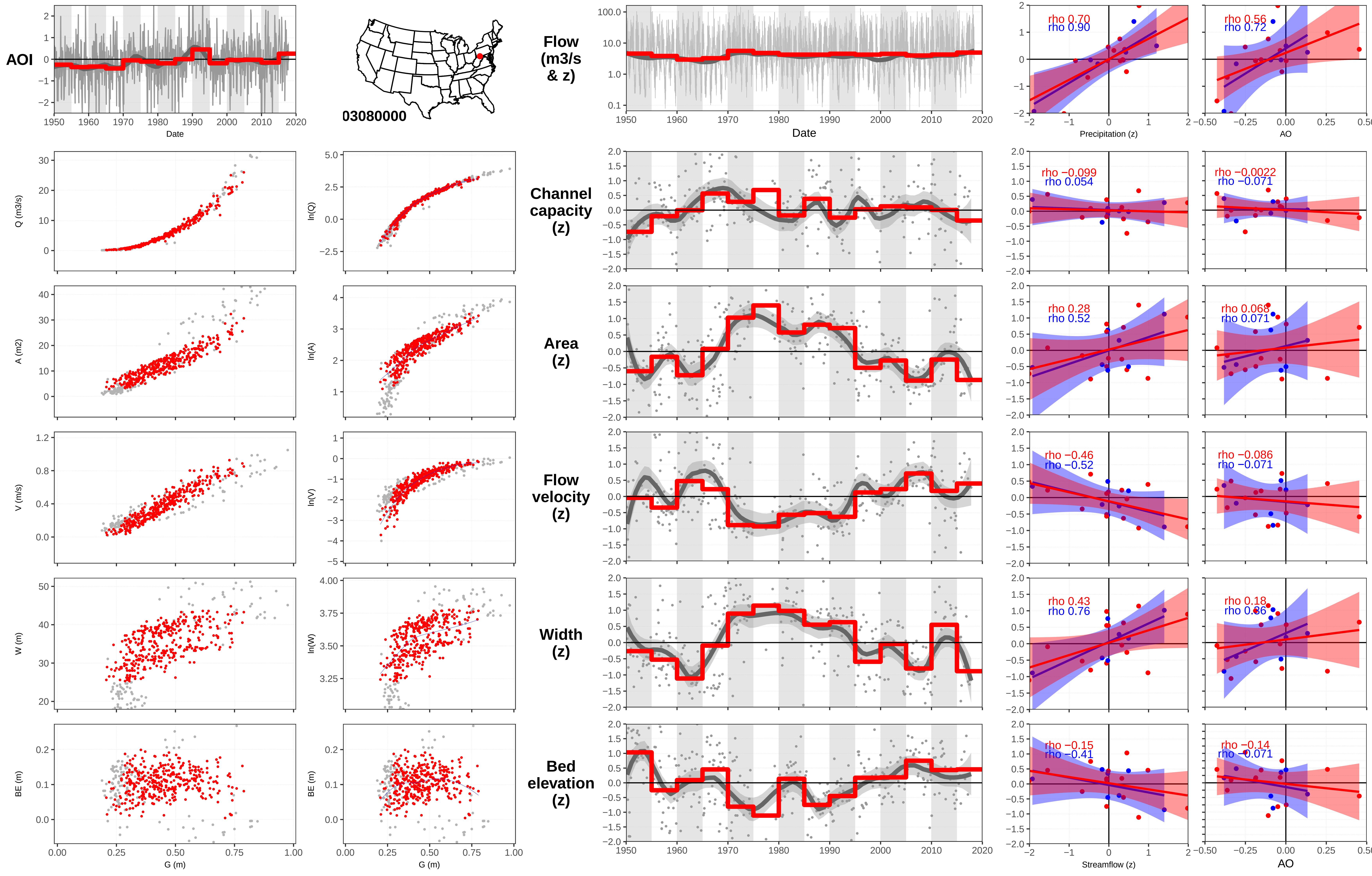
AOI
03080000
Flow (m3/s & z)
Channel capacity (z)
Area (z)
Flow velocity (z)
Width (z)
Bed elevation (z)
Date
Precipitation (z)
AO
Streamflow (z)
G (m)
Q (m3/s)
ln(Q)
A (m2)
ln(A)
V (m/s)
ln(V)
W (m)
ln(W)
BE (m)
rho 0.70
rho 0.90
rho 0.56
rho 0.72
rho −0.099
rho 0.054
rho −0.0022
rho −0.071
rho 0.28
rho 0.52
rho 0.068
rho 0.071
rho −0.46
rho −0.52
rho −0.086
rho −0.071
rho 0.43
rho 0.76
rho 0.18
rho 0.36
rho −0.15
rho −0.41
rho −0.14
rho −0.071

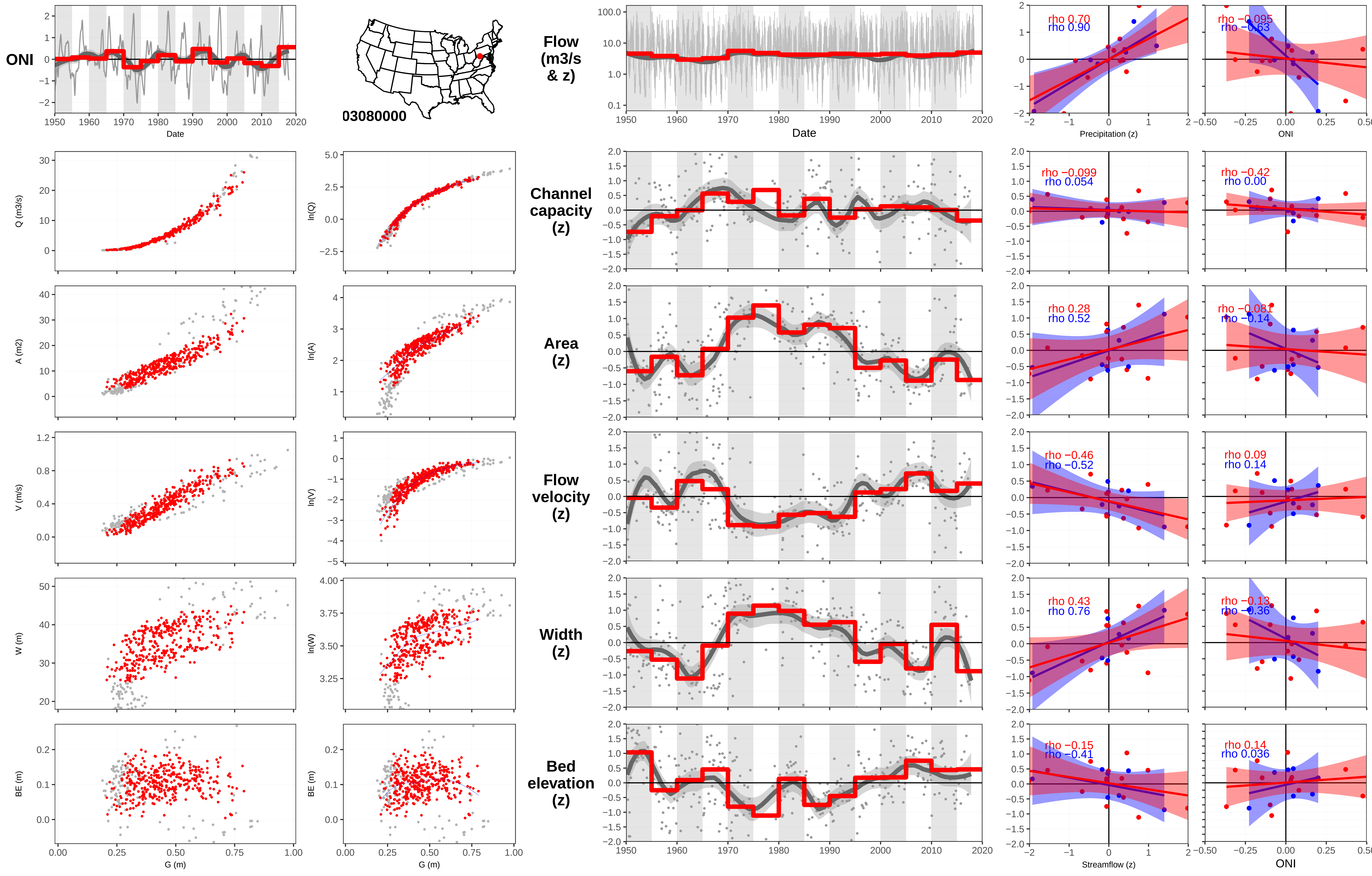
ONI
03080000
Flow (m3/s & z)
Channel capacity (z)
Area (z)
Flow velocity (z)
Width (z)
Bed elevation (z)
Date
Precipitation (z)
Streamflow (z)
ONI
G (m)
Q (m3/s)
ln(Q)
A (m2)
ln(A)
V (m/s)
ln(V)
W (m)
ln(W)
BE (m)
rho 0.70
rho 0.90
rho −0.095
rho −0.63
rho −0.099
rho 0.054
rho −0.42
rho 0.00
rho 0.28
rho 0.52
rho −0.081
rho −0.14
rho −0.46
rho −0.52
rho 0.09
rho 0.14
rho 0.43
rho 0.76
rho −0.13
rho −0.36
rho −0.15
rho −0.41
rho 0.14
rho 0.036

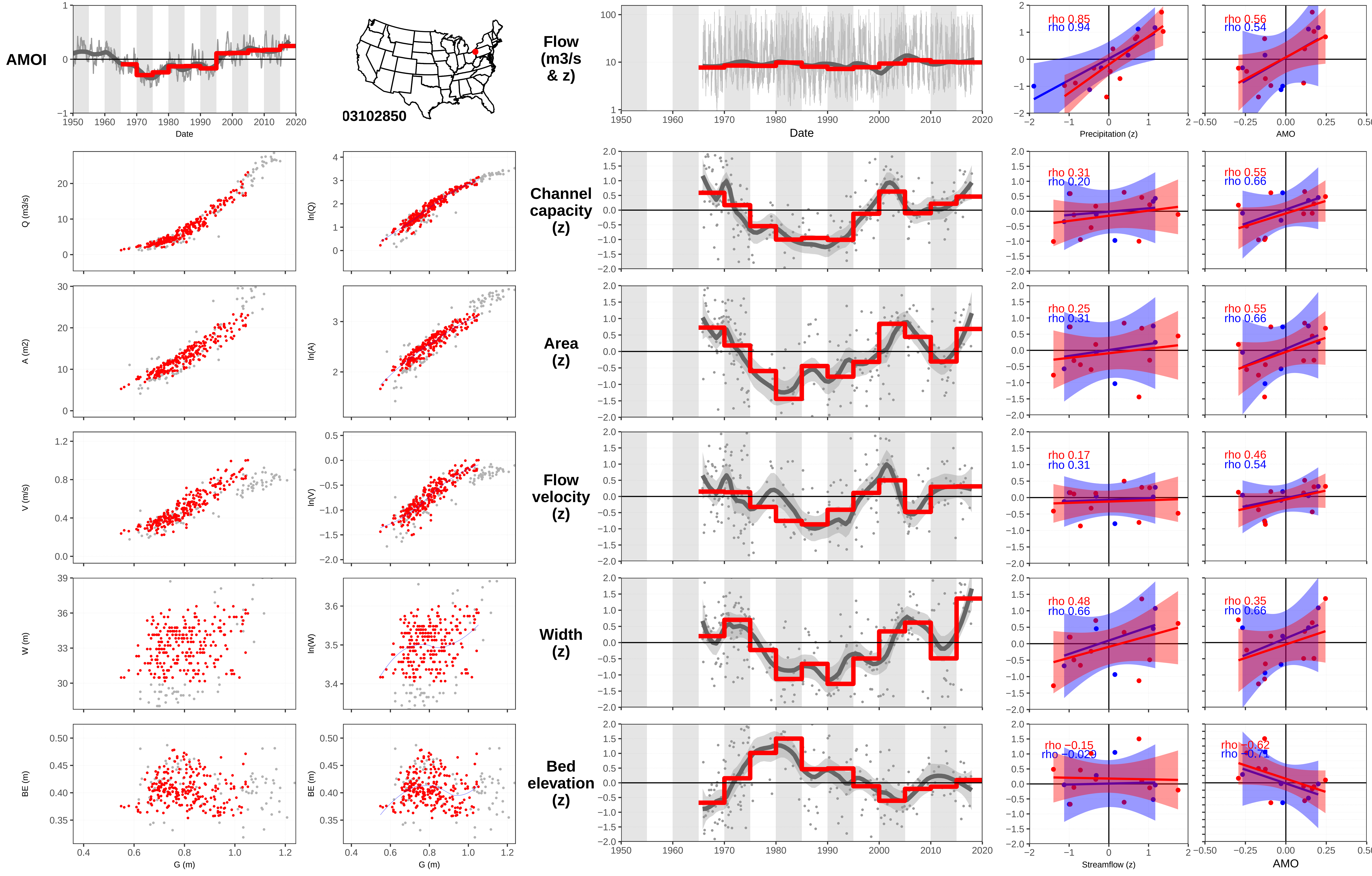

AMOI
03102850
Flow (m3/s & z)
Channel capacity (z)
Area (z)
Flow velocity (z)
Width (z)
Bed elevation (z)
Date
Precipitation (z)
AMO
Streamflow (z)
Q (m3/s)
ln(Q)
A (m2)
ln(A)
V (m/s)
ln(V)
W (m)
ln(W)
BE (m)
G (m)
rho 0.85
rho 0.94
rho 0.56
rho 0.54
rho 0.31
rho 0.20
rho 0.55
rho 0.66
rho 0.25
rho 0.31
rho 0.55
rho 0.66
rho 0.17
rho 0.31
rho 0.46
rho 0.54
rho 0.48
rho 0.66
rho 0.35
rho 0.66
rho −0.15
rho −0.029
rho −0.62
rho −0.71

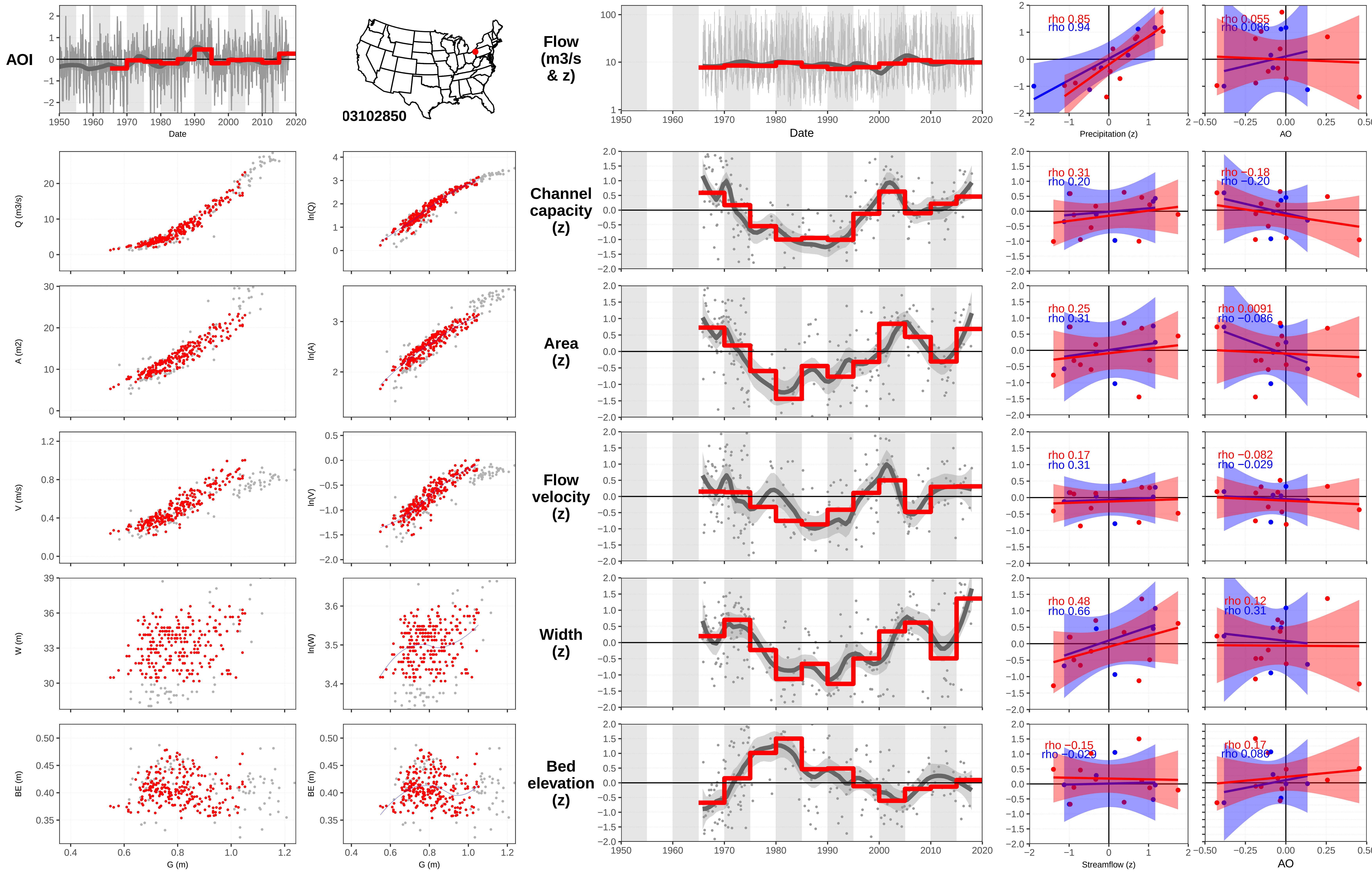

AOI
03102850
Date
Flow (m3/s & z)
Precipitation (z)
AO
rho 0.85
rho 0.94
rho 0.055
rho 0.086
Channel capacity (z)
Q (m3/s)
ln(Q)
rho 0.31
rho 0.20
rho −0.18
rho −0.20
Area (z)
A (m2)
ln(A)
rho 0.25
rho 0.31
rho 0.0091
rho −0.086
Flow velocity (z)
V (m/s)
ln(V)
rho 0.17
rho 0.31
rho −0.082
rho −0.029
Width (z)
W (m)
ln(W)
rho 0.48
rho 0.66
rho 0.12
rho 0.31
Bed elevation (z)
BE (m)
rho −0.15
rho −0.029
rho 0.17
rho 0.080
G (m)
Streamflow (z)

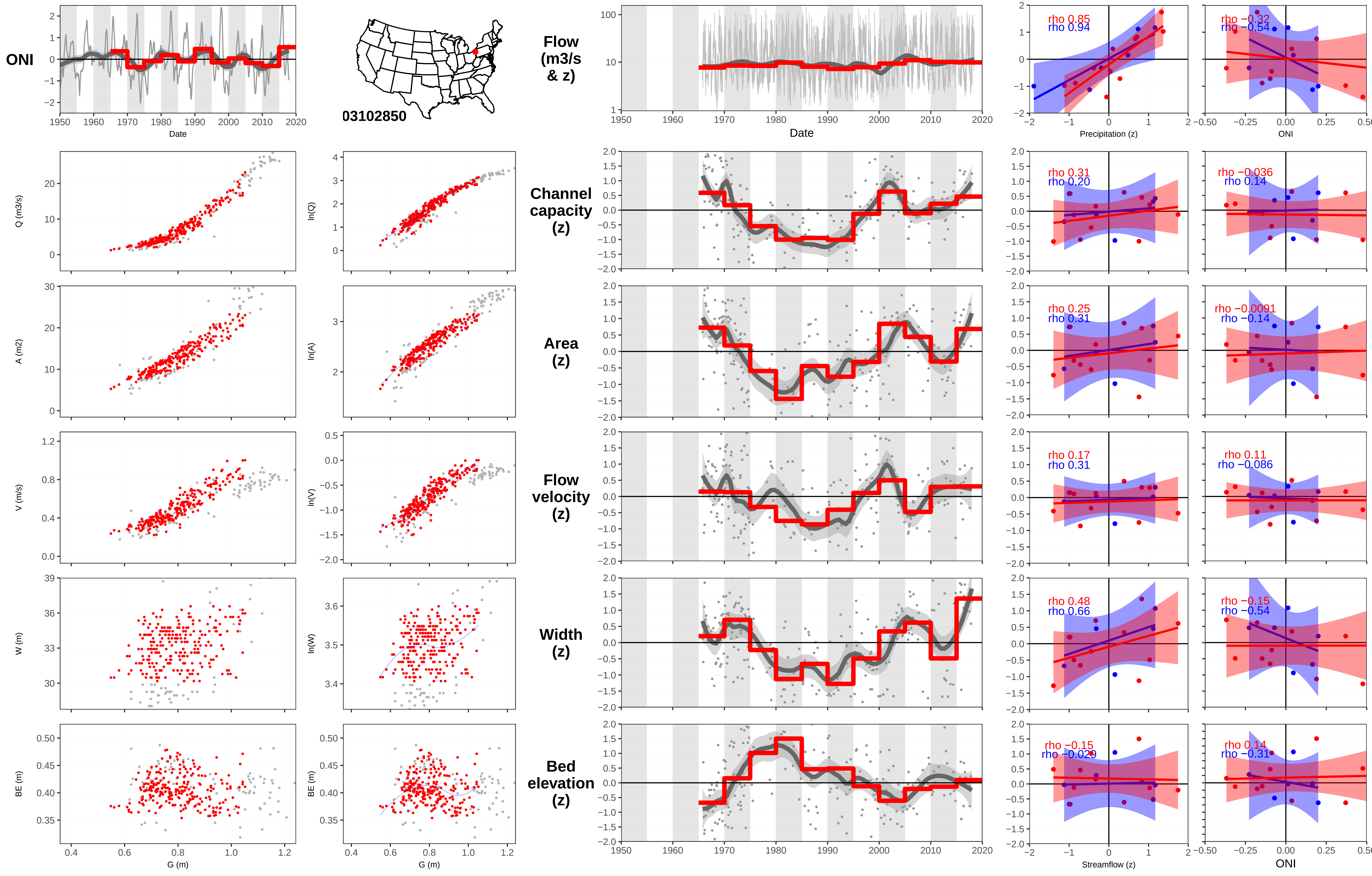

ONI
03102850
Flow (m3/s & z)
Channel capacity (z)
Area (z)
Flow velocity (z)
Width (z)
Bed elevation (z)
Date
Precipitation (z)
ONI
Streamflow (z)
G (m)
Q (m3/s)
ln(Q)
A (m2)
ln(A)
V (m/s)
ln(V)
W (m)
ln(W)
BE (m)
rho 0.85
rho 0.94
rho −0.32
rho −0.54
rho 0.31
rho 0.20
rho −0.036
rho 0.14
rho 0.25
rho 0.31
rho −0.0091
rho −0.14
rho 0.17
rho 0.31
rho 0.11
rho −0.086
rho 0.48
rho 0.66
rho −0.15
rho −0.54
rho −0.15
rho −0.029
rho 0.14
rho −0.31

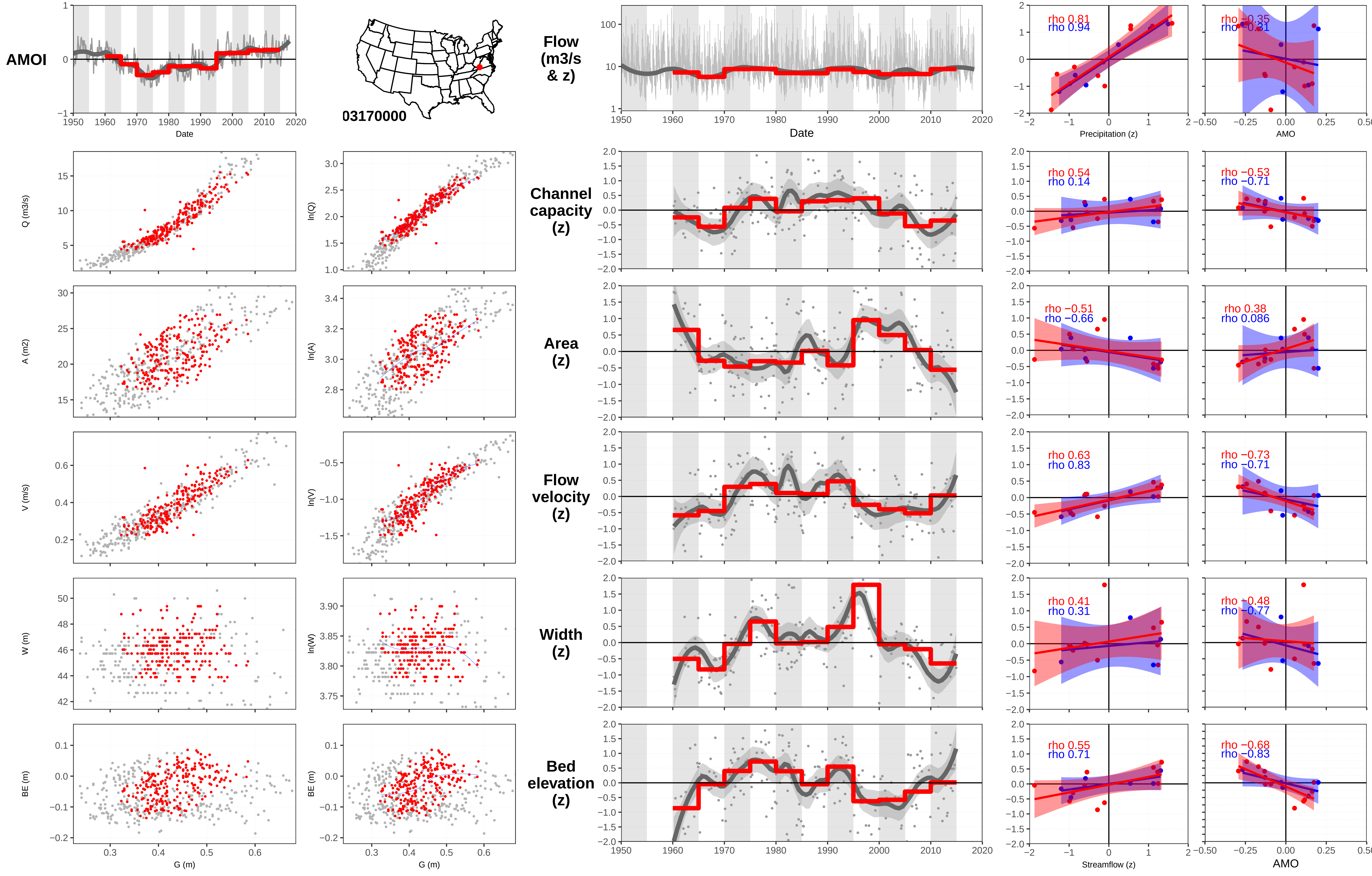

AMOI
Date
03170000
Flow (m3/s & z)
Date
Precipitation (z)
AMO
rho 0.81
rho 0.94
rho −0.35
rho −0.31
Q (m3/s)
ln(Q)
Channel capacity (z)
rho 0.54
rho 0.14
rho −0.53
rho −0.71
A (m2)
ln(A)
Area (z)
rho −0.51
rho −0.66
rho 0.38
rho 0.086
V (m/s)
ln(V)
Flow velocity (z)
rho 0.63
rho 0.83
rho −0.73
rho −0.71
W (m)
ln(W)
Width (z)
rho 0.41
rho 0.31
rho −0.48
rho −0.77
BE (m)
BE (m)
Bed elevation (z)
rho 0.55
rho 0.71
rho −0.68
rho −0.83
G (m)
G (m)
Streamflow (z)
AMO

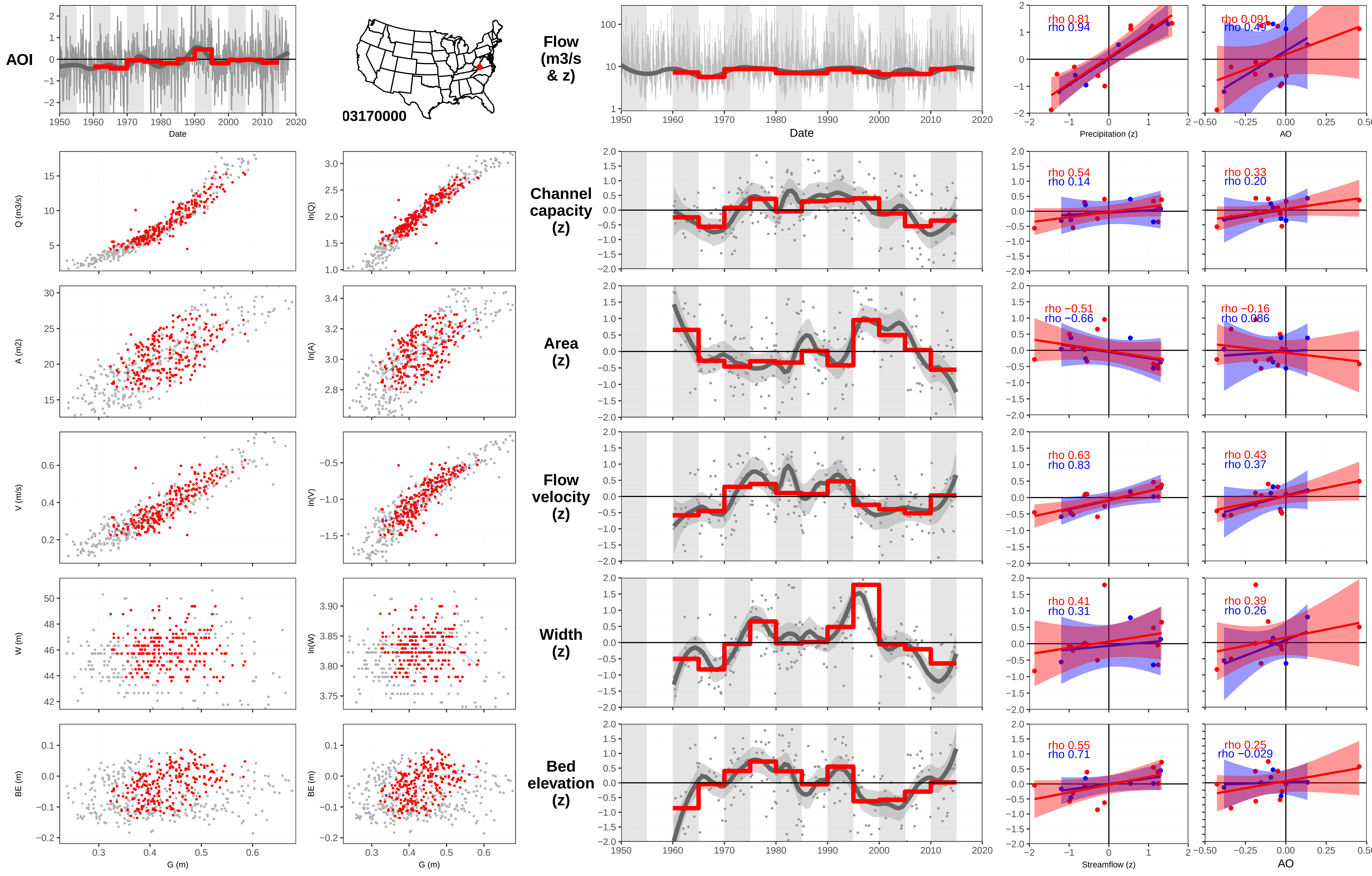

AOI
03170000
Flow (m3/s & z)
Channel capacity (z)
Area (z)
Flow velocity (z)
Width (z)
Bed elevation (z)
Date
Precipitation (z)
AO
Streamflow (z)
G (m)
Q (m3/s)
ln(Q)
A (m2)
ln(A)
V (m/s)
ln(V)
W (m)
ln(W)
BE (m)
rho 0.81
rho 0.94
rho 0.091
rho 0.49
rho 0.54
rho 0.14
rho 0.33
rho 0.20
rho −0.51
rho −0.66
rho −0.16
rho 0.086
rho 0.63
rho 0.83
rho 0.43
rho 0.37
rho 0.41
rho 0.31
rho 0.39
rho 0.26
rho 0.55
rho 0.71
rho 0.25
rho −0.029

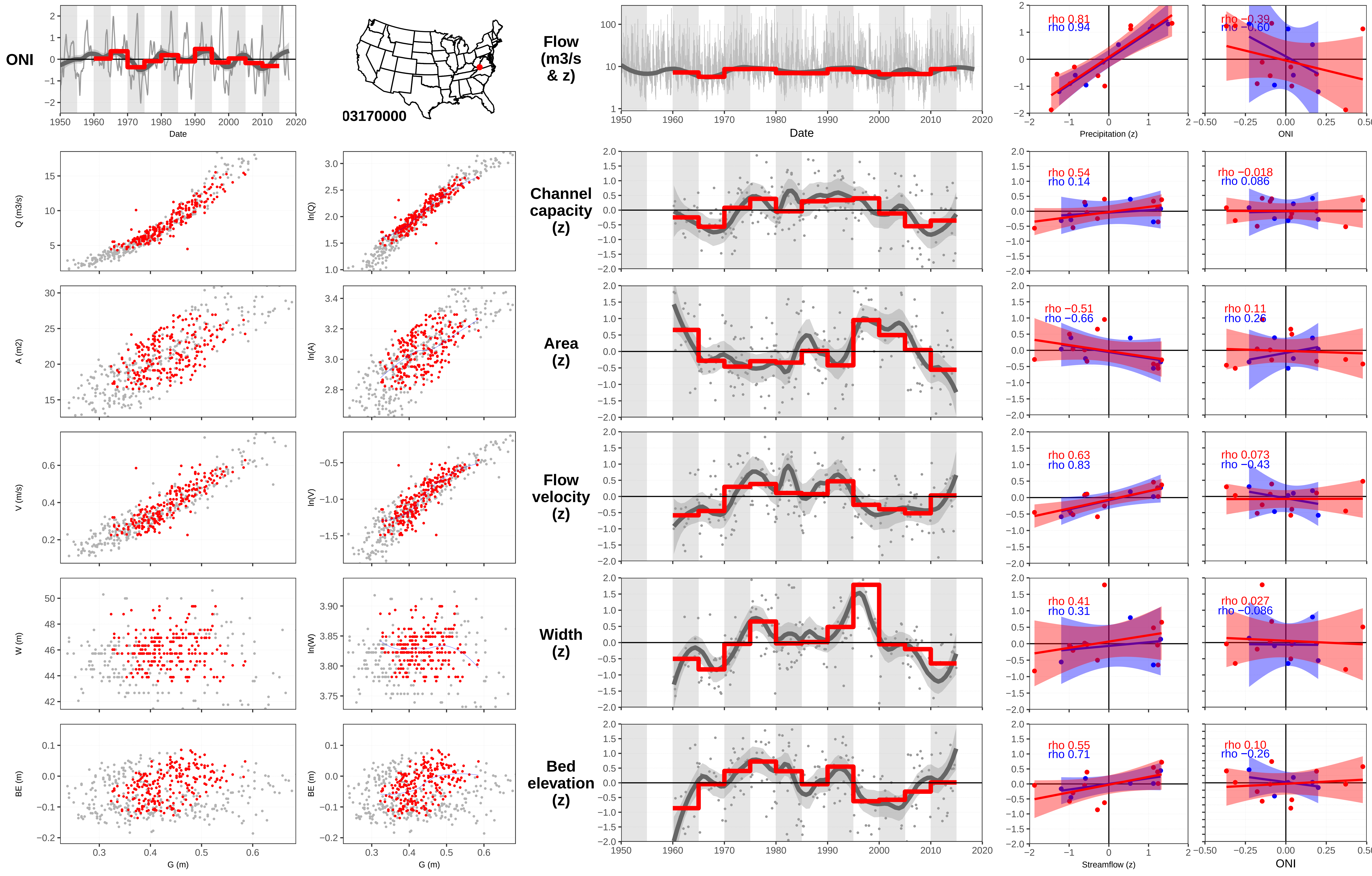

ONI
03170000
Flow (m3/s & z)
Channel capacity (z)
Area (z)
Flow velocity (z)
Width (z)
Bed elevation (z)
Date
Precipitation (z)
Streamflow (z)
ONI
G (m)
Q (m3/s)
ln(Q)
A (m2)
ln(A)
V (m/s)
ln(V)
W (m)
ln(W)
BE (m)
rho 0.81
rho 0.94
rho −0.39
rho −0.60
rho 0.54
rho 0.14
rho −0.018
rho 0.086
rho −0.51
rho −0.66
rho 0.11
rho 0.26
rho 0.63
rho 0.83
rho 0.073
rho −0.43
rho 0.41
rho 0.31
rho 0.027
rho −0.086
rho 0.55
rho 0.71
rho 0.10
rho −0.26

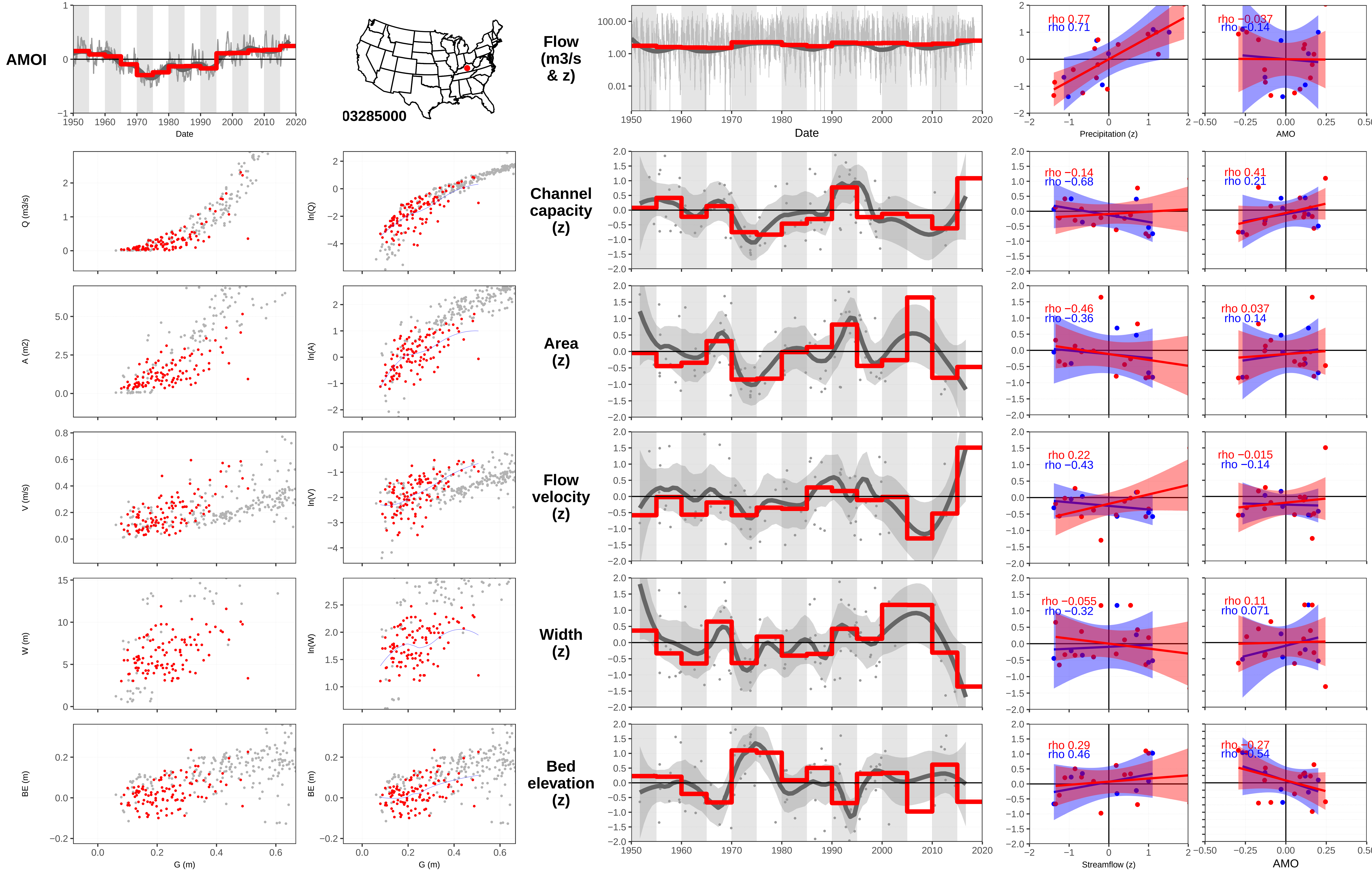
AMOI
03285000
Flow (m3/s & z)
Channel capacity (z)
Area (z)
Flow velocity (z)
Width (z)
Bed elevation (z)
Date
Precipitation (z)
AMO
Streamflow (z)
G (m)
Q (m3/s)
ln(Q)
A (m2)
ln(A)
V (m/s)
ln(V)
W (m)
ln(W)
BE (m)
rho 0.77
rho 0.71
rho −0.037
rho −0.14
rho −0.14
rho −0.68
rho 0.41
rho 0.21
rho −0.46
rho −0.36
rho 0.037
rho 0.14
rho 0.22
rho −0.43
rho −0.015
rho −0.14
rho −0.055
rho −0.32
rho 0.11
rho 0.071
rho 0.29
rho 0.46
rho −0.27
rho −0.54

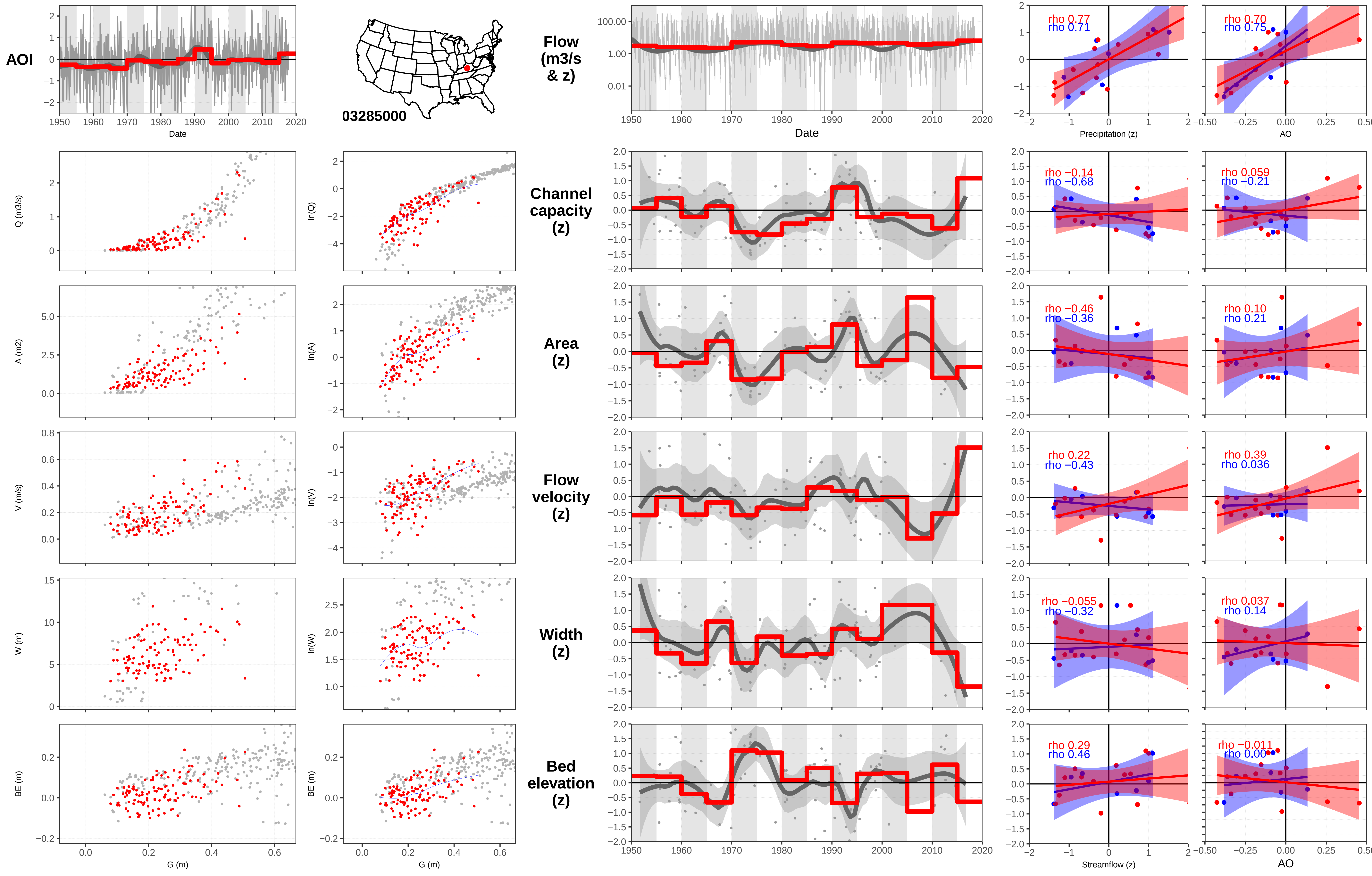

AOI
03285000
Flow (m3/s & z)
Channel capacity (z)
Area (z)
Flow velocity (z)
Width (z)
Bed elevation (z)
Date
Precipitation (z)
AO
Streamflow (z)
G (m)
Q (m3/s)
ln(Q)
A (m2)
ln(A)
V (m/s)
ln(V)
W (m)
ln(W)
BE (m)
rho 0.77
rho 0.71
rho 0.70
rho 0.75
rho −0.14
rho −0.68
rho 0.059
rho −0.21
rho −0.46
rho −0.36
rho 0.10
rho 0.21
rho 0.22
rho −0.43
rho 0.39
rho 0.036
rho −0.055
rho −0.32
rho 0.037
rho 0.14
rho 0.29
rho 0.46
rho −0.011
rho 0.00

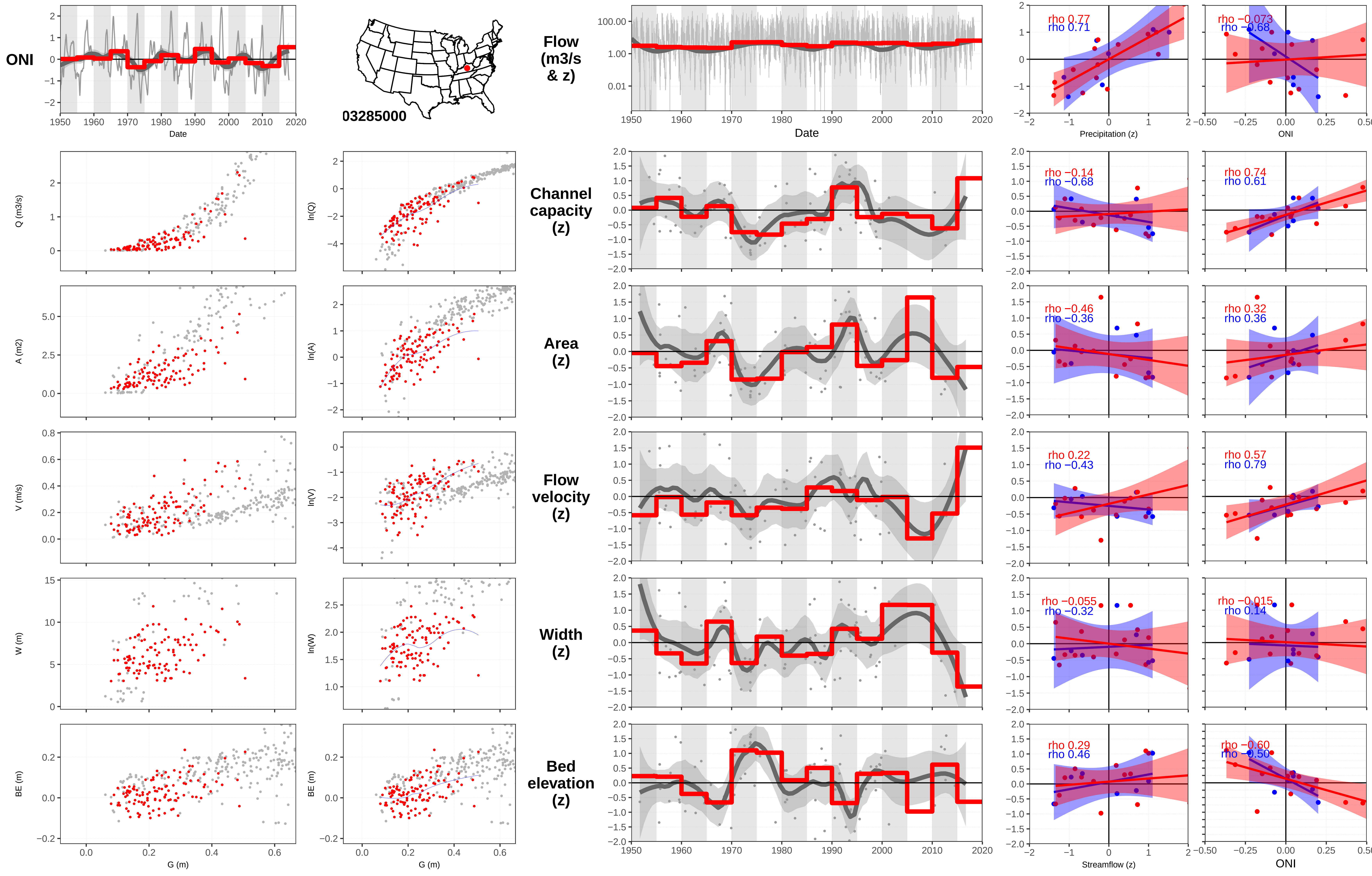

ONI
03285000
Flow (m3/s & z)
Channel capacity (z)
Area (z)
Flow velocity (z)
Width (z)
Bed elevation (z)
Date
Precipitation (z)
Streamflow (z)
ONI
G (m)
Q (m3/s)
ln(Q)
A (m2)
ln(A)
V (m/s)
ln(V)
W (m)
ln(W)
BE (m)
rho 0.77
rho 0.71
rho −0.073
rho −0.68
rho −0.14
rho −0.68
rho 0.74
rho 0.61
rho −0.46
rho −0.36
rho 0.32
rho 0.36
rho 0.22
rho −0.43
rho 0.57
rho 0.79
rho −0.055
rho −0.32
rho −0.015
rho 0.14
rho 0.29
rho 0.46
rho −0.60
rho −0.50

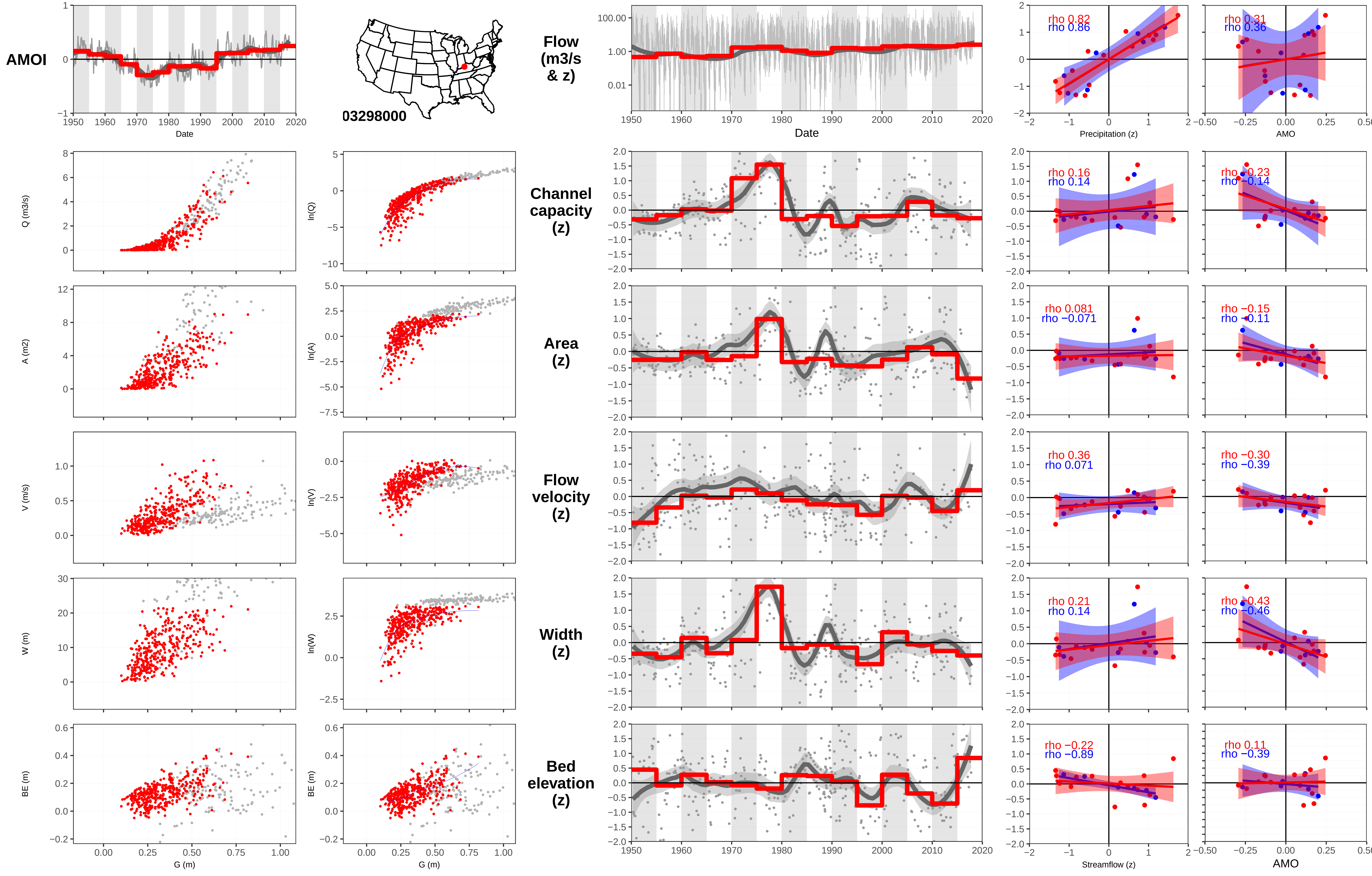

AMOI
03298000
Flow (m3/s & z)
Channel capacity (z)
Area (z)
Flow velocity (z)
Width (z)
Bed elevation (z)
Date
Precipitation (z)
AMO
Streamflow (z)
Q (m3/s)
ln(Q)
A (m2)
ln(A)
V (m/s)
ln(V)
W (m)
ln(W)
BE (m)
G (m)
rho 0.82
rho 0.86
rho 0.31
rho 0.36
rho 0.16
rho 0.14
rho −0.23
rho −0.14
rho 0.081
rho −0.071
rho −0.15
rho −0.11
rho 0.36
rho 0.071
rho −0.30
rho −0.39
rho 0.21
rho 0.14
rho −0.43
rho −0.46
rho −0.22
rho −0.89
rho 0.11
rho −0.39

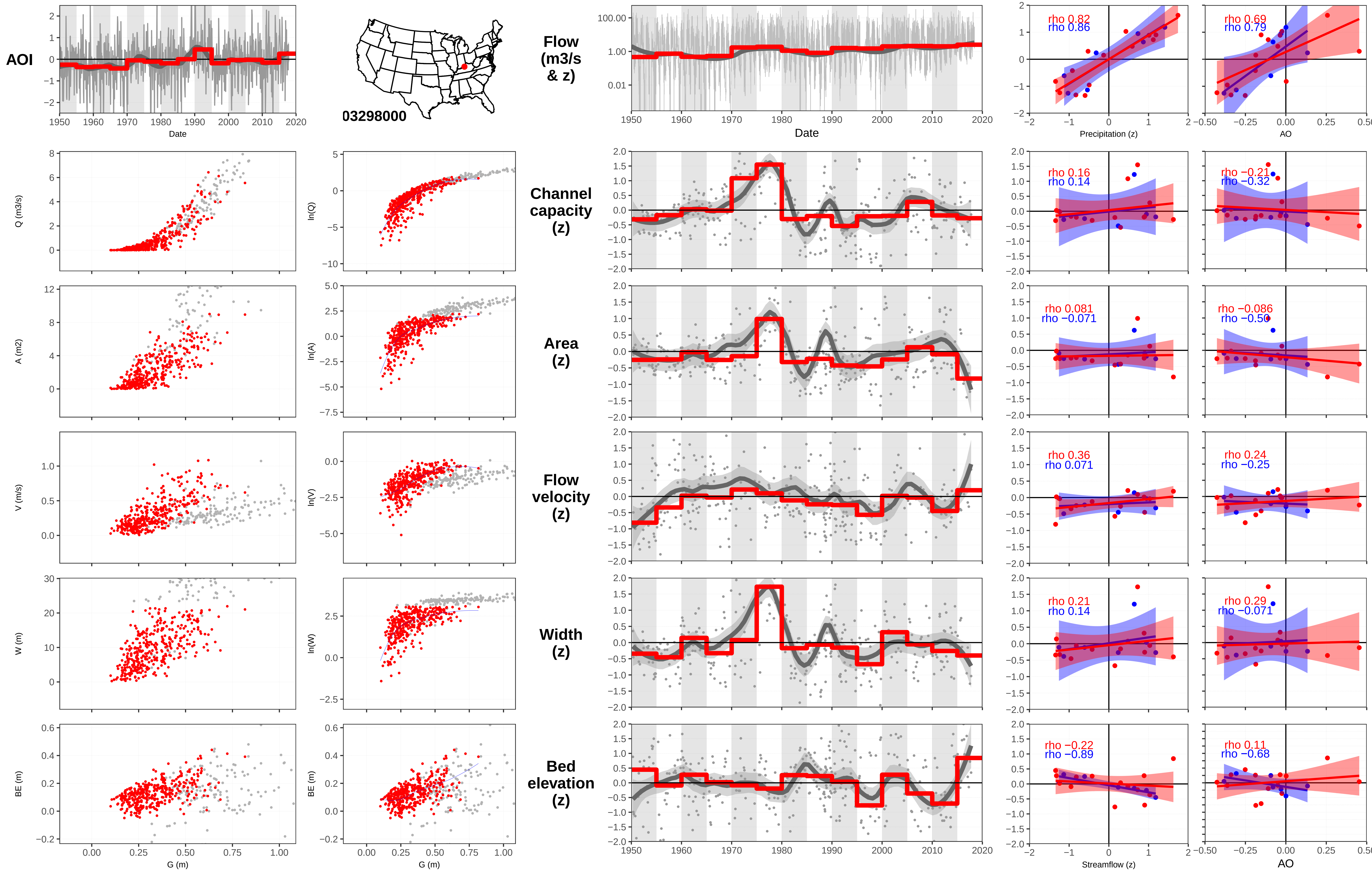

AOI
03298000
Flow (m3/s & z)
Channel capacity (z)
Area (z)
Flow velocity (z)
Width (z)
Bed elevation (z)
Date
Precipitation (z)
AO
Streamflow (z)
G (m)
Q (m3/s)
ln(Q)
A (m2)
ln(A)
V (m/s)
ln(V)
W (m)
ln(W)
BE (m)
rho 0.82
rho 0.86
rho 0.69
rho 0.79
rho 0.16
rho 0.14
rho −0.21
rho −0.32
rho 0.081
rho −0.071
rho −0.086
rho −0.50
rho 0.36
rho 0.071
rho 0.24
rho −0.25
rho 0.21
rho 0.14
rho 0.29
rho −0.071
rho −0.22
rho −0.89
rho 0.11
rho −0.68

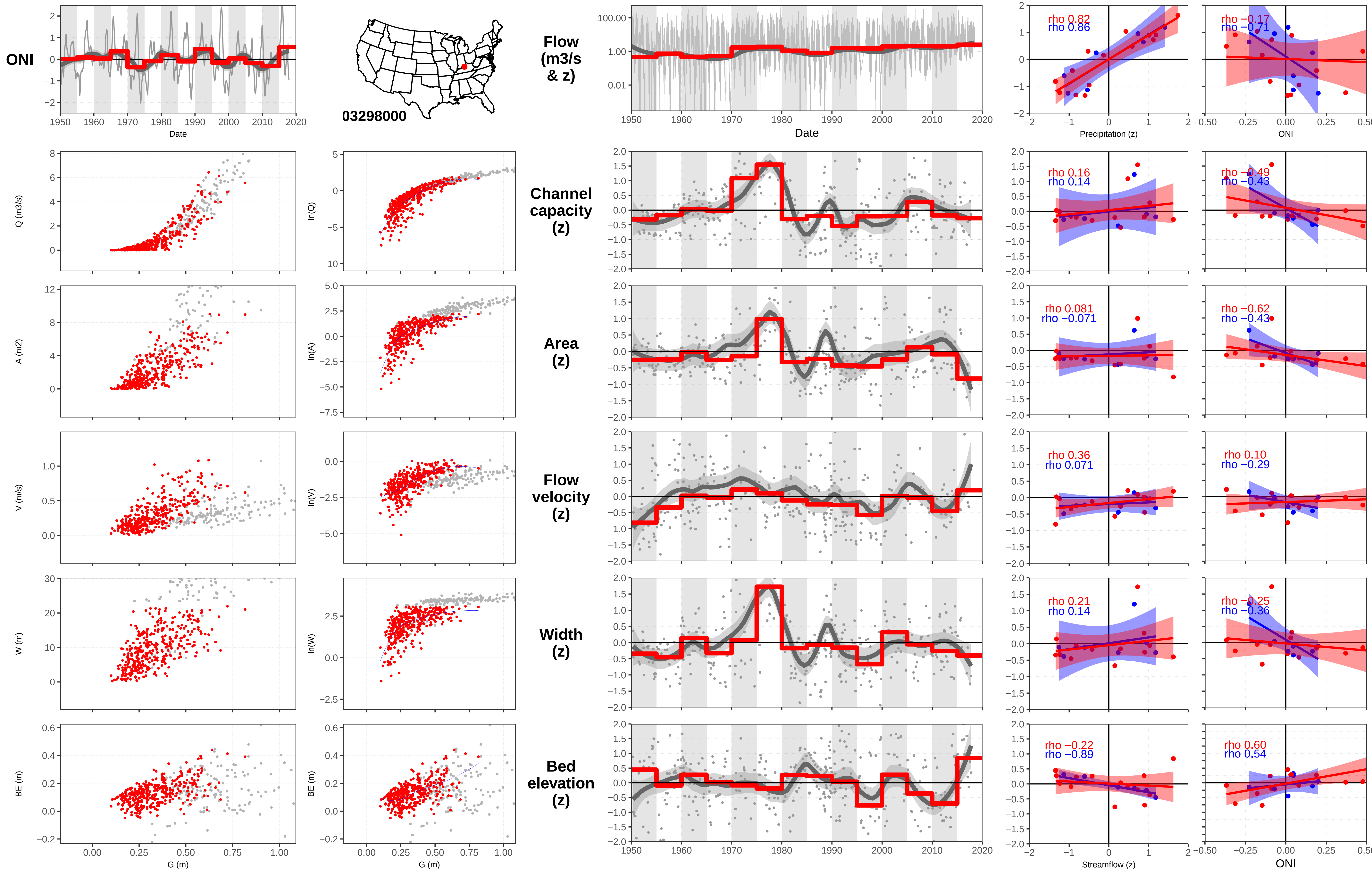

ONI
03298000
Flow (m3/s & z)
Channel capacity (z)
Area (z)
Flow velocity (z)
Width (z)
Bed elevation (z)
Date
Precipitation (z)
Streamflow (z)
ONI
G (m)
Q (m3/s)
ln(Q)
A (m2)
ln(A)
V (m/s)
ln(V)
W (m)
ln(W)
BE (m)
rho 0.82
rho 0.86
rho −0.17
rho −0.71
rho 0.16
rho 0.14
rho −0.49
rho −0.43
rho 0.081
rho −0.071
rho −0.62
rho −0.43
rho 0.36
rho 0.071
rho 0.10
rho −0.29
rho 0.21
rho 0.14
rho −0.25
rho −0.36
rho −0.22
rho −0.89
rho 0.60
rho 0.54

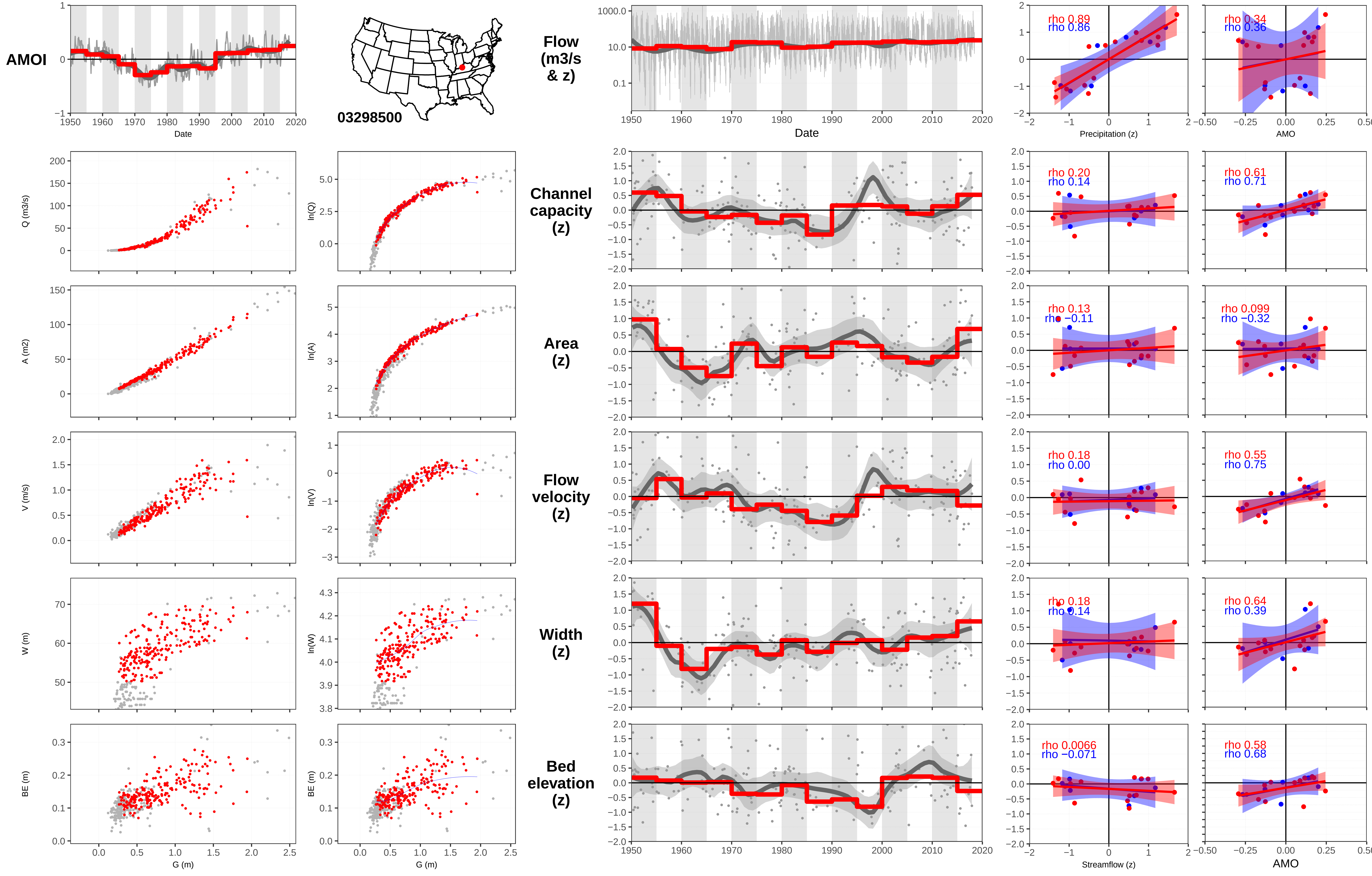

AMOI
03298500
Flow (m3/s & z)
Channel capacity (z)
Area (z)
Flow velocity (z)
Width (z)
Bed elevation (z)
Date
Precipitation (z)
Streamflow (z)
AMO
G (m)
Q (m3/s)
ln(Q)
A (m2)
ln(A)
V (m/s)
ln(V)
W (m)
ln(W)
BE (m)
rho 0.89
rho 0.86
rho 0.34
rho 0.36
rho 0.20
rho 0.14
rho 0.61
rho 0.71
rho 0.13
rho −0.11
rho 0.099
rho −0.32
rho 0.18
rho 0.00
rho 0.55
rho 0.75
rho 0.18
rho 0.14
rho 0.64
rho 0.39
rho 0.0066
rho −0.071
rho 0.58
rho 0.68

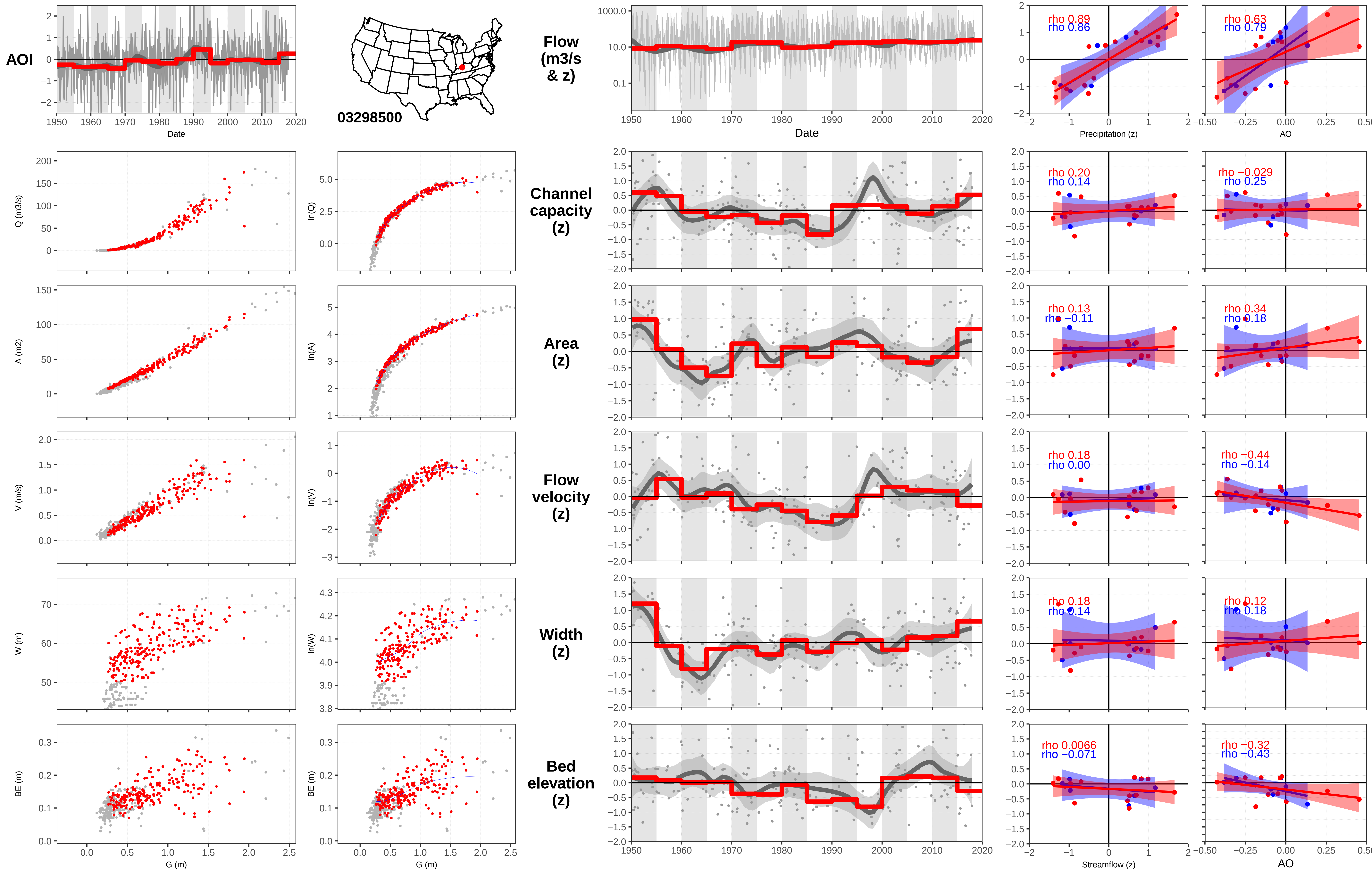

AOI
03298500
Flow (m3/s & z)
Channel capacity (z)
Area (z)
Flow velocity (z)
Width (z)
Bed elevation (z)
Date
Precipitation (z)
AO
Streamflow (z)
G (m)
Q (m3/s)
ln(Q)
A (m2)
ln(A)
V (m/s)
ln(V)
W (m)
ln(W)
BE (m)
rho 0.89
rho 0.86
rho 0.63
rho 0.79
rho 0.20
rho 0.14
rho −0.029
rho 0.25
rho 0.13
rho −0.11
rho 0.34
rho 0.18
rho 0.18
rho 0.00
rho −0.44
rho −0.14
rho 0.18
rho 0.14
rho 0.12
rho 0.18
rho 0.0066
rho −0.071
rho −0.32
rho −0.43

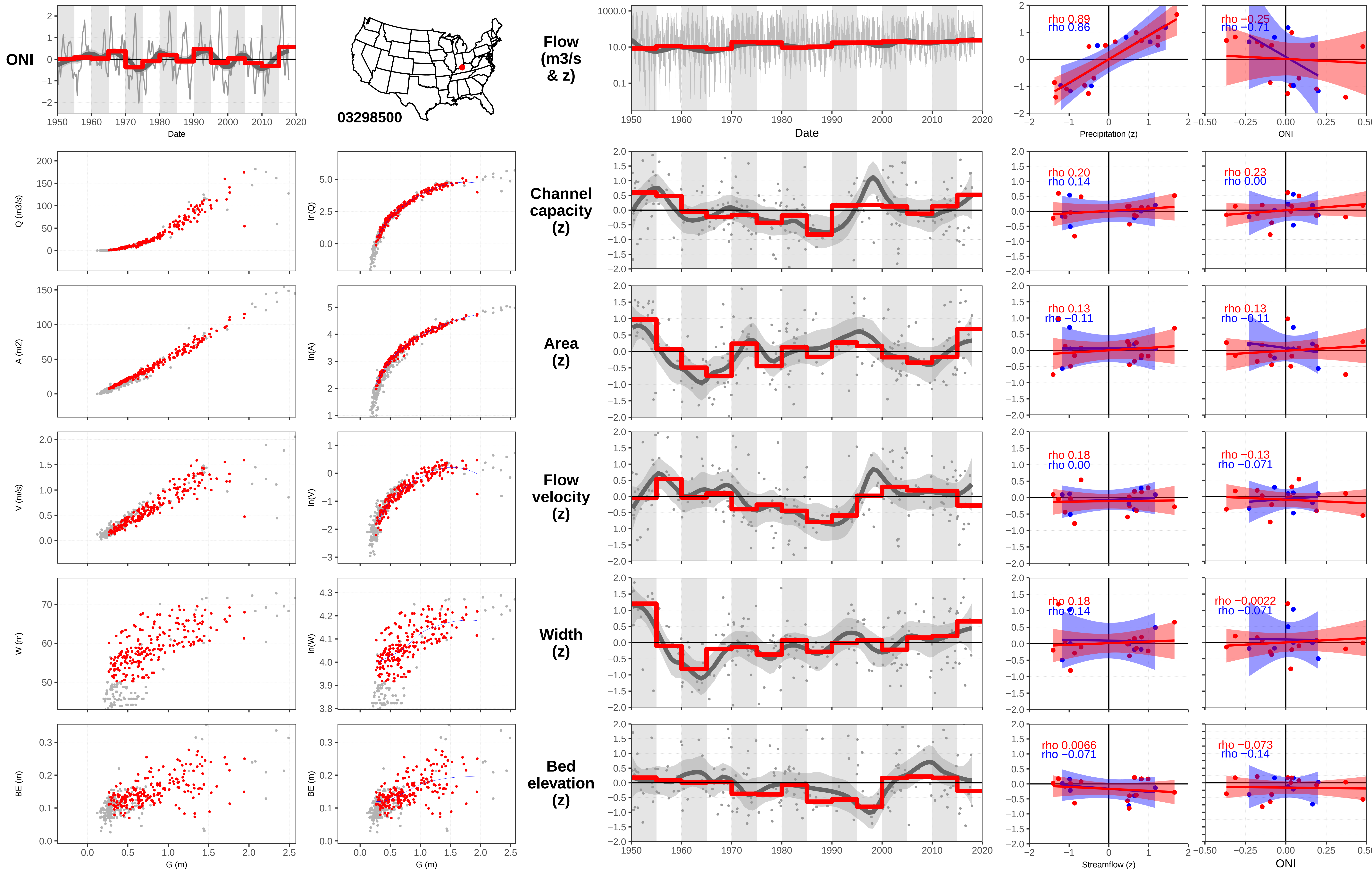
ONI
03298500
Flow (m3/s & z)
Date
Precipitation (z)
ONI
rho 0.89
rho 0.86
rho −0.25
rho −0.71
Channel capacity (z)
Q (m3/s)
ln(Q)
rho 0.20
rho 0.14
rho 0.23
rho 0.00
Area (z)
A (m2)
ln(A)
rho 0.13
rho −0.11
rho 0.13
rho −0.11
Flow velocity (z)
V (m/s)
ln(V)
rho 0.18
rho 0.00
rho −0.13
rho −0.071
Width (z)
W (m)
ln(W)
rho 0.18
rho 0.14
rho −0.0022
rho −0.071
Bed elevation (z)
BE (m)
rho 0.0066
rho −0.071
rho −0.073
rho −0.14
G (m)
Streamflow (z)
ONI

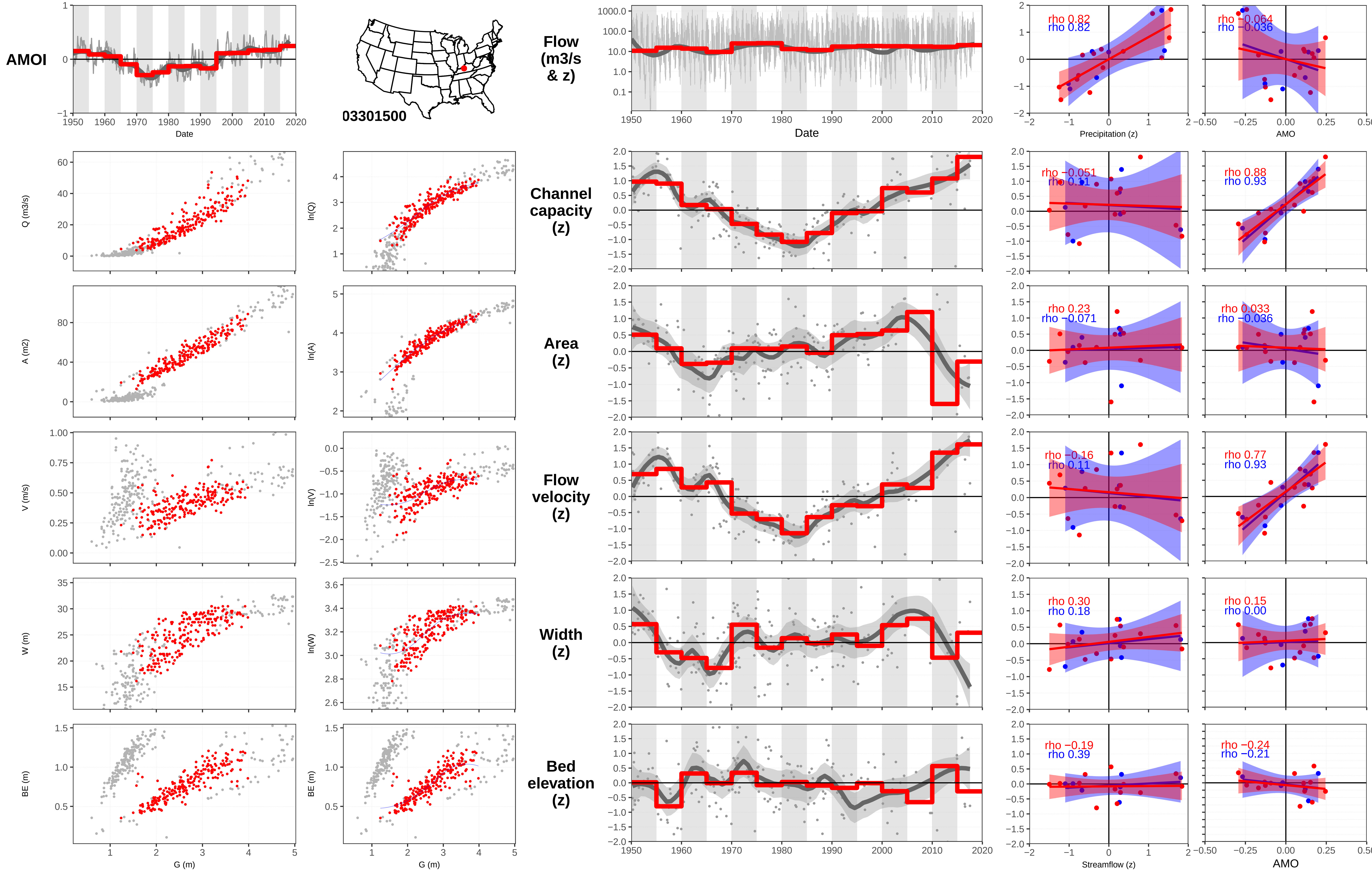

AMOI
03301500
Flow (m3/s & z)
Channel capacity (z)
Area (z)
Flow velocity (z)
Width (z)
Bed elevation (z)
Date
Precipitation (z)
AMO
Streamflow (z)
G (m)
Q (m3/s)
ln(Q)
A (m2)
ln(A)
V (m/s)
ln(V)
W (m)
ln(W)
BE (m)
rho 0.82
rho 0.82
rho −0.064
rho −0.036
rho −0.051
rho 0.11
rho 0.88
rho 0.93
rho 0.23
rho −0.071
rho 0.033
rho −0.036
rho −0.16
rho 0.11
rho 0.77
rho 0.93
rho 0.30
rho 0.18
rho 0.15
rho 0.00
rho −0.19
rho 0.39
rho −0.24
rho −0.21

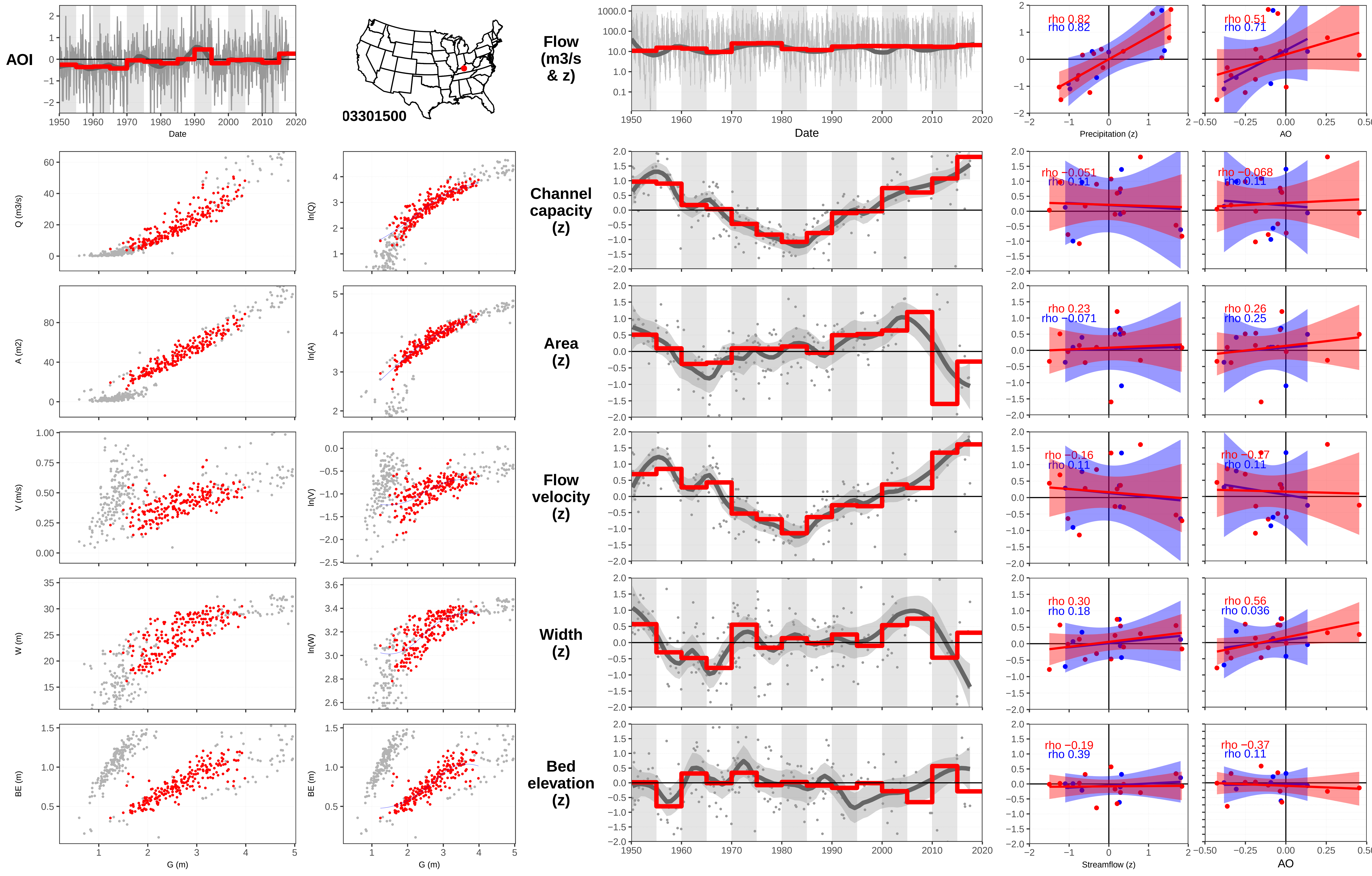

AOI
Date
03301500
Flow (m3/s & z)
Date
rho 0.82
rho 0.82
Precipitation (z)
rho 0.51
rho 0.71
AO
Q (m3/s)
ln(Q)
Channel capacity (z)
rho −0.051
rho 0.11
rho −0.068
rho 0.11
A (m2)
ln(A)
Area (z)
rho 0.23
rho −0.071
rho 0.26
rho 0.25
V (m/s)
ln(V)
Flow velocity (z)
rho −0.16
rho 0.11
rho −0.17
rho 0.11
W (m)
ln(W)
Width (z)
rho 0.30
rho 0.18
rho 0.56
rho 0.036
BE (m)
BE (m)
Bed elevation (z)
rho −0.19
rho 0.39
rho −0.37
rho 0.11
G (m)
G (m)
Streamflow (z)
AO

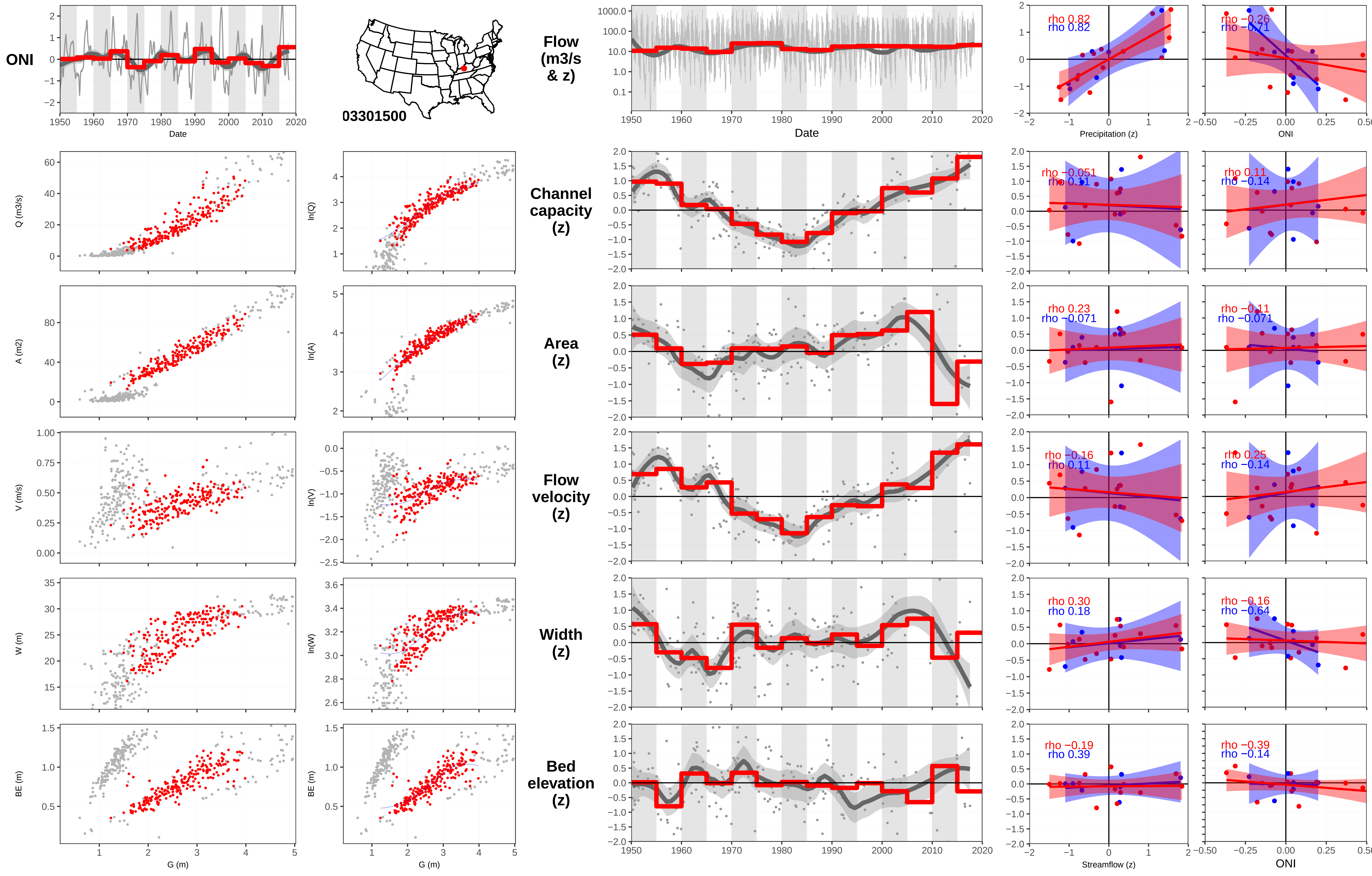
ONI
03301500
Flow (m3/s & z)
Channel capacity (z)
Area (z)
Flow velocity (z)
Width (z)
Bed elevation (z)
Date
Precipitation (z)
Streamflow (z)
ONI
G (m)
Q (m3/s)
ln(Q)
A (m2)
ln(A)
V (m/s)
ln(V)
W (m)
ln(W)
BE (m)
rho 0.82
rho 0.82
rho −0.26
rho −0.71
rho −0.051
rho 0.11
rho 0.11
rho −0.14
rho 0.23
rho −0.071
rho −0.11
rho −0.071
rho −0.16
rho 0.11
rho 0.25
rho −0.14
rho 0.30
rho 0.18
rho −0.16
rho −0.64
rho −0.19
rho 0.39
rho −0.39
rho −0.14

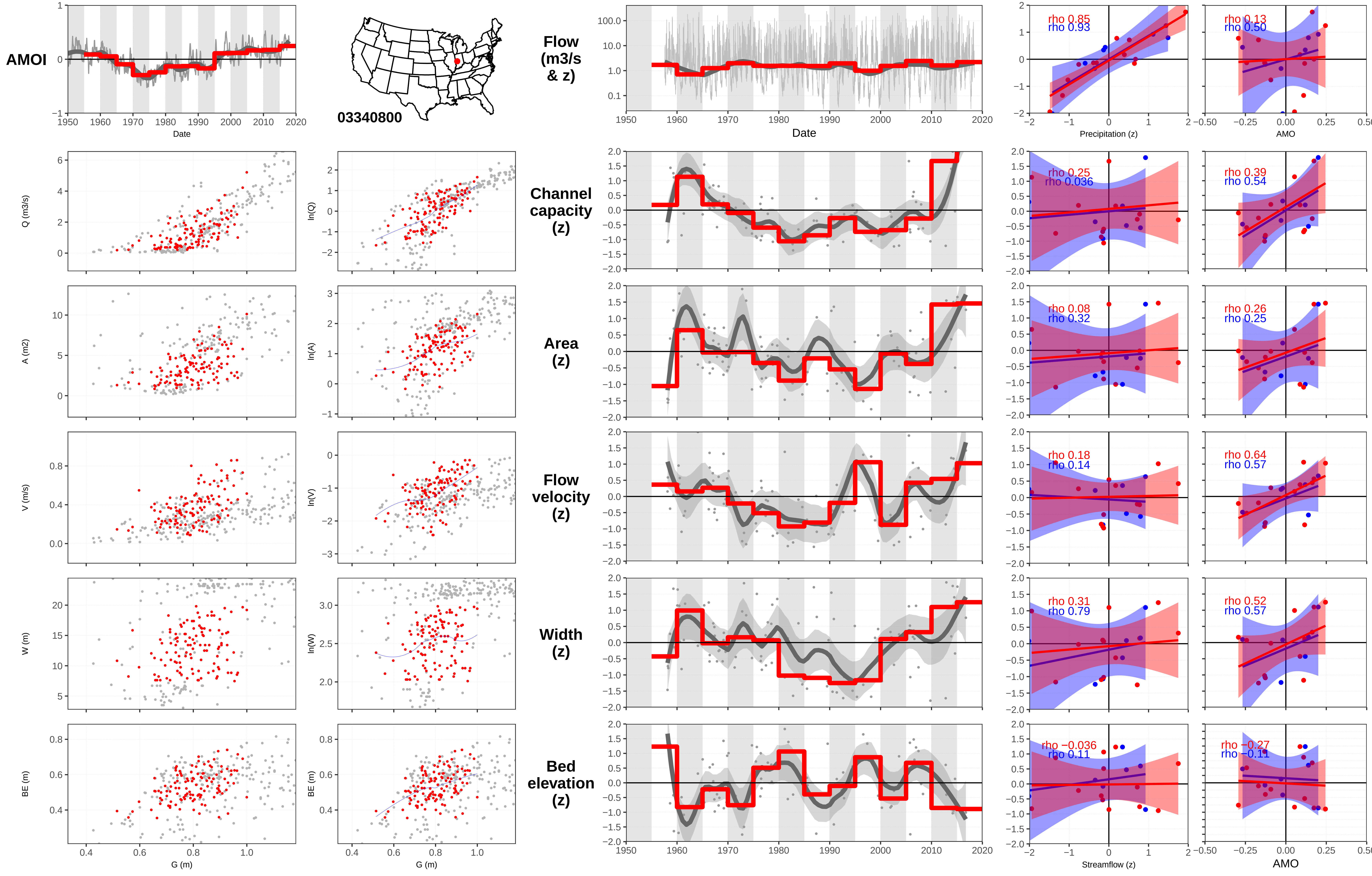

AMOI
03340800
Flow (m3/s & z)
Channel capacity (z)
Area (z)
Flow velocity (z)
Width (z)
Bed elevation (z)
Date
Precipitation (z)
AMO
Streamflow (z)
G (m)
Q (m3/s)
ln(Q)
A (m2)
ln(A)
V (m/s)
ln(V)
W (m)
ln(W)
BE (m)
rho 0.85
rho 0.93
rho 0.13
rho 0.50
rho 0.25
rho 0.036
rho 0.39
rho 0.54
rho 0.08
rho 0.32
rho 0.26
rho 0.25
rho 0.18
rho 0.14
rho 0.64
rho 0.57
rho 0.31
rho 0.79
rho 0.52
rho 0.57
rho −0.036
rho 0.11
rho −0.27
rho −0.11

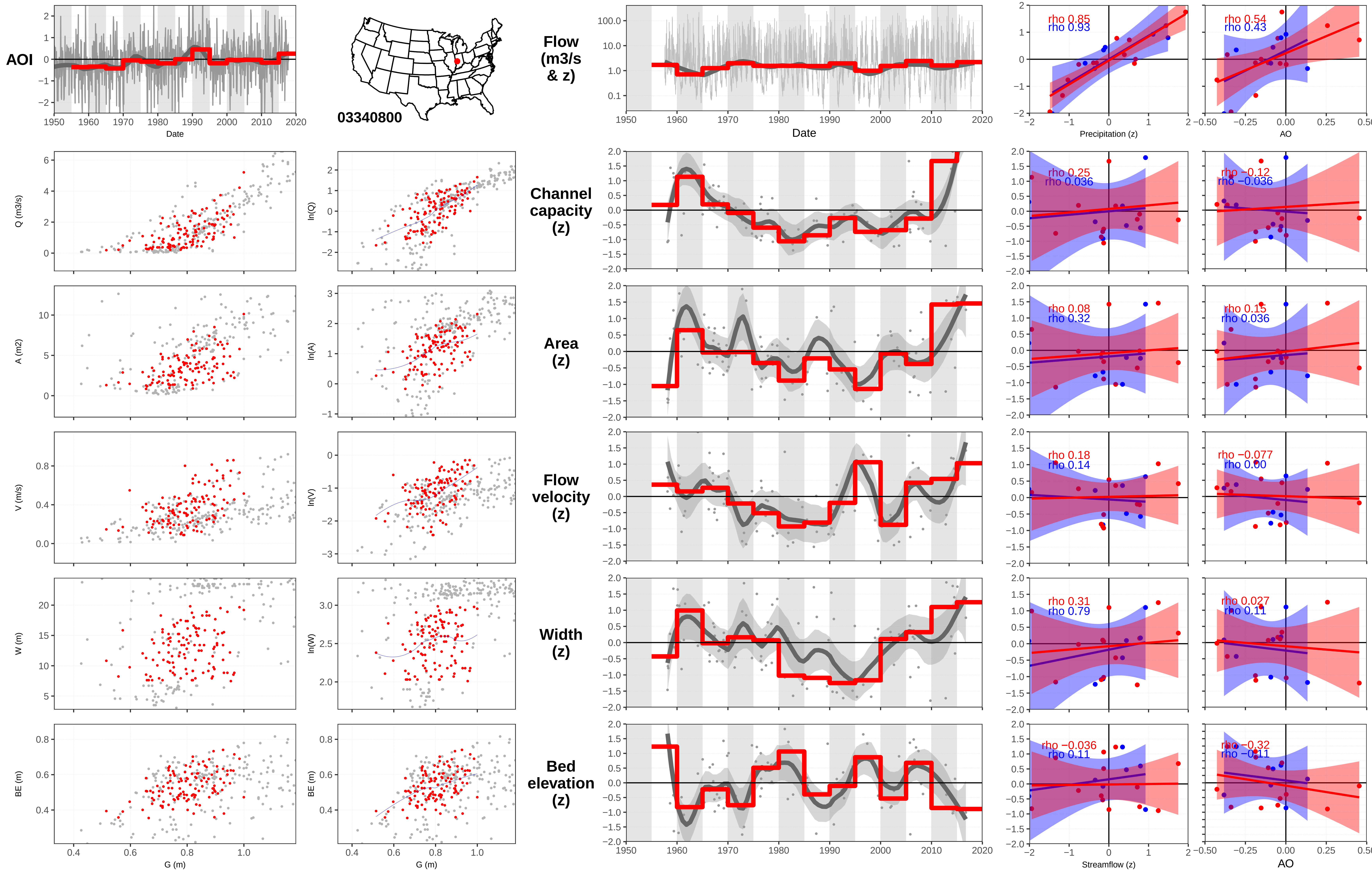

AOI
03340800
Flow (m3/s & z)
Channel capacity (z)
Area (z)
Flow velocity (z)
Width (z)
Bed elevation (z)
Date
Precipitation (z)
AO
Streamflow (z)
G (m)
Q (m3/s)
ln(Q)
A (m2)
ln(A)
V (m/s)
ln(V)
W (m)
ln(W)
BE (m)
rho 0.85
rho 0.93
rho 0.54
rho 0.43
rho 0.25
rho 0.036
rho −0.12
rho −0.036
rho 0.08
rho 0.32
rho 0.15
rho 0.036
rho 0.18
rho 0.14
rho −0.077
rho 0.00
rho 0.31
rho 0.79
rho 0.027
rho 0.11
rho −0.036
rho 0.11
rho −0.32
rho −0.11

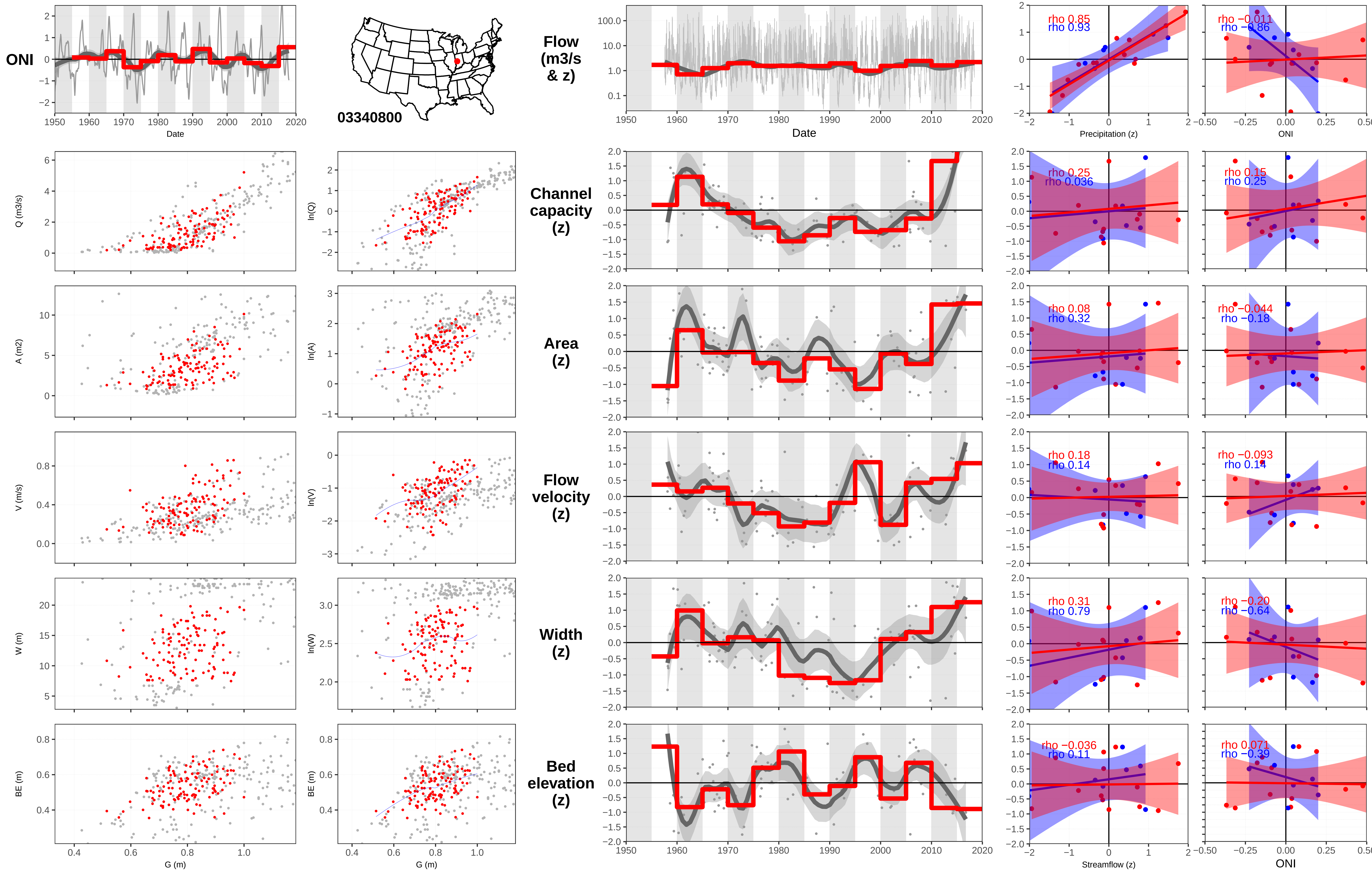

ONI
03340800
Flow (m3/s & z)
Channel capacity (z)
Area (z)
Flow velocity (z)
Width (z)
Bed elevation (z)
Date
Precipitation (z)
Streamflow (z)
ONI
G (m)
Q (m3/s)
ln(Q)
A (m2)
ln(A)
V (m/s)
ln(V)
W (m)
ln(W)
BE (m)
rho 0.85
rho 0.93
rho −0.011
rho −0.86
rho 0.25
rho 0.036
rho 0.15
rho 0.25
rho 0.08
rho 0.32
rho −0.044
rho −0.18
rho 0.18
rho 0.14
rho −0.093
rho 0.14
rho 0.31
rho 0.79
rho −0.20
rho −0.64
rho −0.036
rho 0.11
rho 0.071
rho −0.39

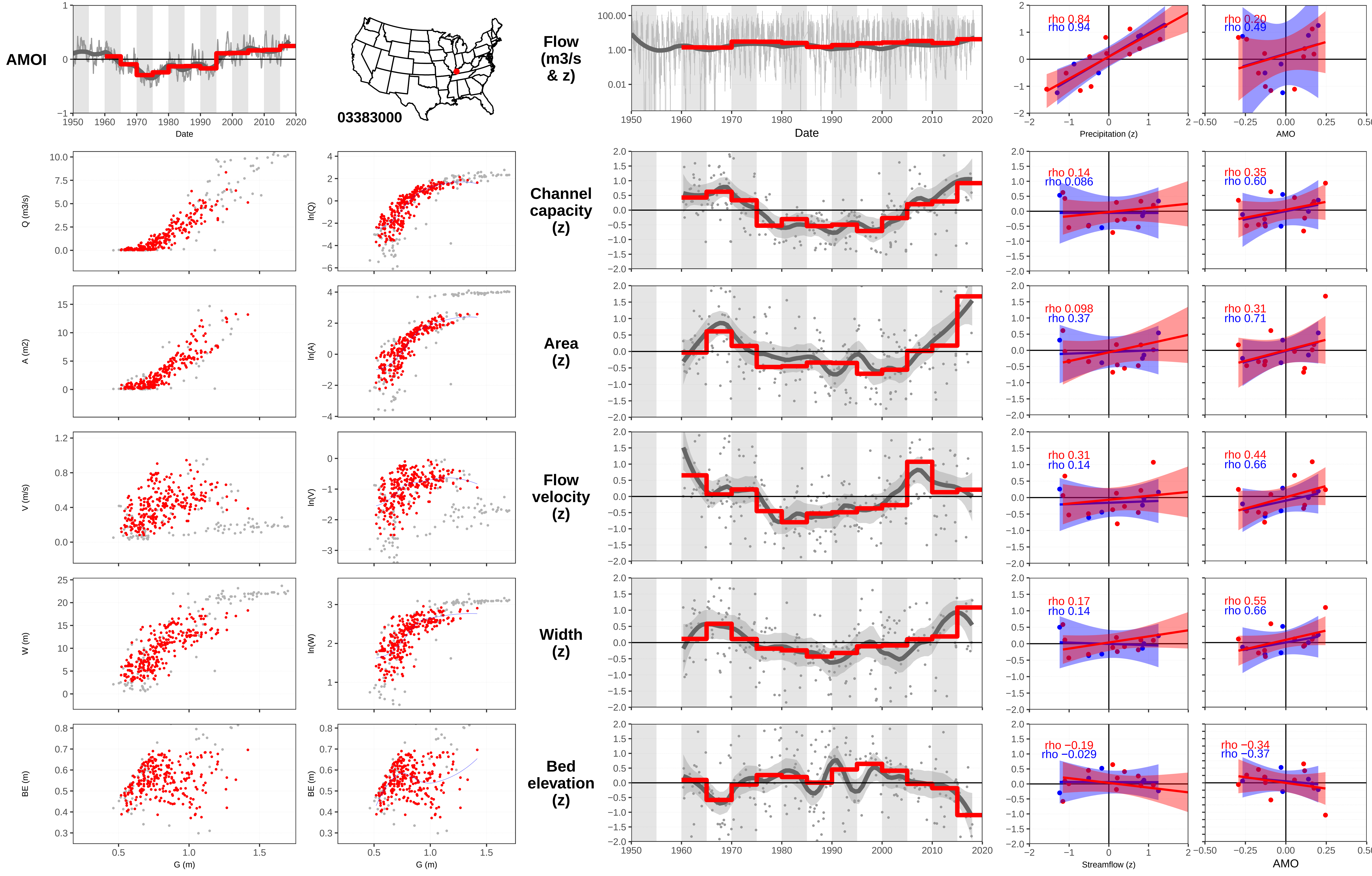

AMOI
03383000
Flow (m3/s & z)
Channel capacity (z)
Area (z)
Flow velocity (z)
Width (z)
Bed elevation (z)
Date
Precipitation (z)
AMO
Streamflow (z)
G (m)
Q (m3/s)
ln(Q)
A (m2)
ln(A)
V (m/s)
ln(V)
W (m)
ln(W)
BE (m)
rho 0.84
rho 0.94
rho 0.20
rho 0.49
rho 0.14
rho 0.086
rho 0.35
rho 0.60
rho 0.098
rho 0.37
rho 0.31
rho 0.71
rho 0.31
rho 0.14
rho 0.44
rho 0.66
rho 0.17
rho 0.14
rho 0.55
rho 0.66
rho −0.19
rho −0.029
rho −0.34
rho −0.37

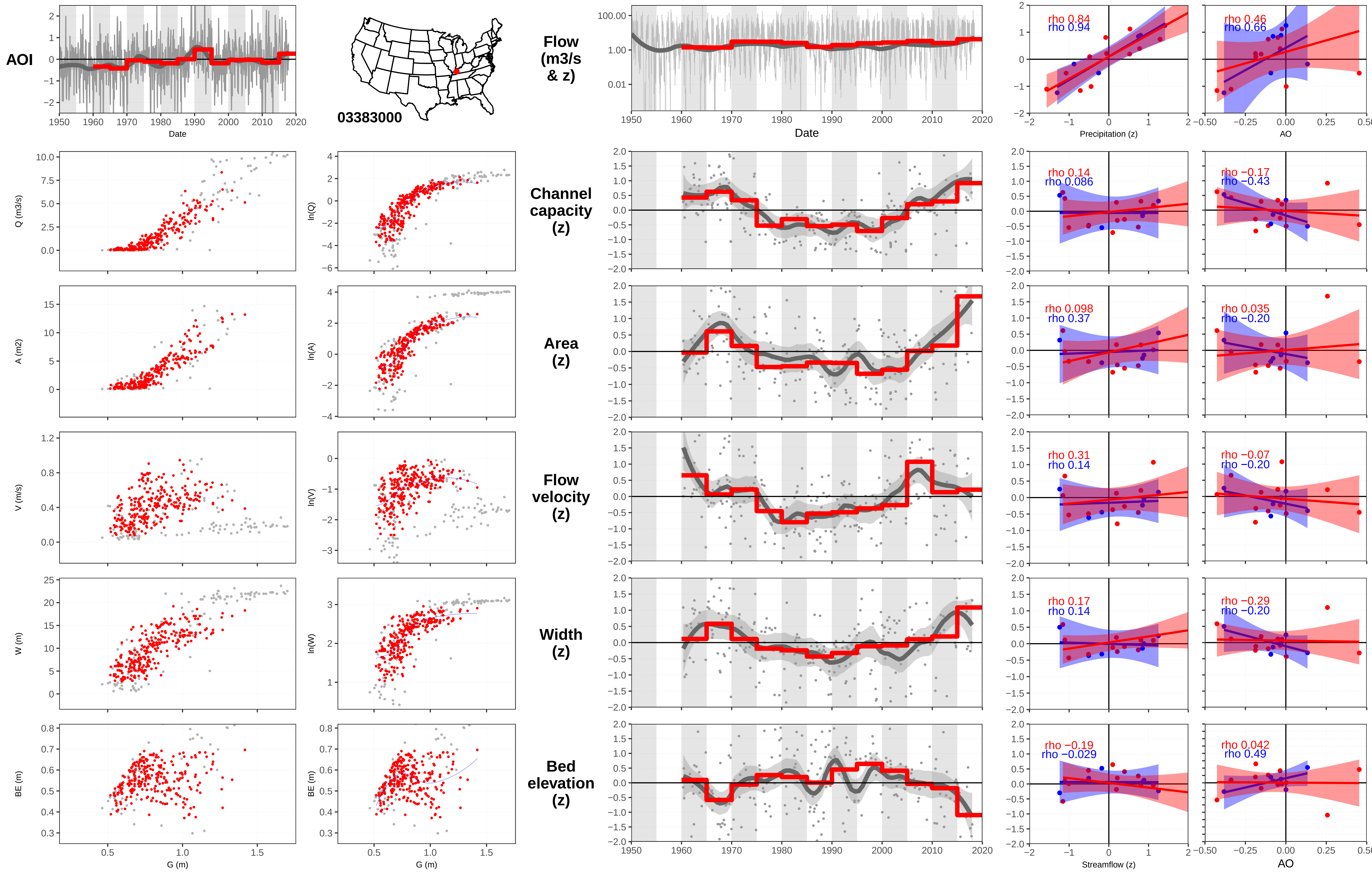

AOI
03383000
Flow (m3/s & z)
Date
Precipitation (z)
AO
rho 0.84
rho 0.94
rho 0.46
rho 0.66
Channel capacity (z)
rho 0.14
rho 0.086
rho −0.17
rho −0.43
Area (z)
rho 0.098
rho 0.37
rho 0.035
rho −0.20
Flow velocity (z)
rho 0.31
rho 0.14
rho −0.07
rho −0.20
Width (z)
rho 0.17
rho 0.14
rho −0.29
rho −0.20
Bed elevation (z)
rho −0.19
rho −0.029
rho 0.042
rho 0.49
Q (m3/s)
ln(Q)
A (m2)
ln(A)
V (m/s)
ln(V)
W (m)
ln(W)
BE (m)
G (m)
Streamflow (z)

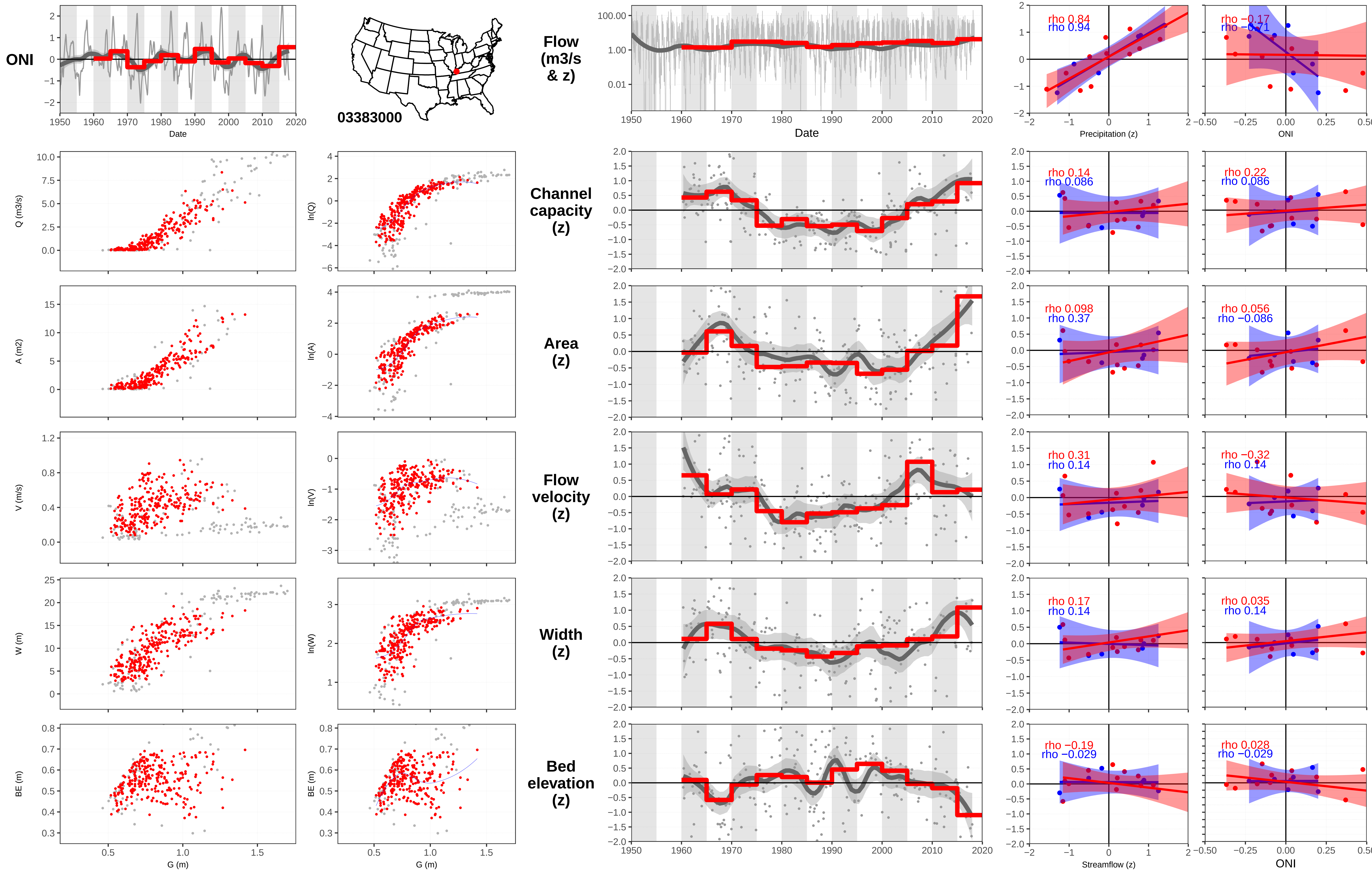

ONI
03383000
Flow (m3/s & z)
Date
Precipitation (z)
ONI
rho 0.84
rho 0.94
rho −0.17
rho −0.71
Channel capacity (z)
Q (m3/s)
ln(Q)
rho 0.14
rho 0.086
rho 0.22
rho 0.086
Area (z)
A (m2)
ln(A)
rho 0.098
rho 0.37
rho 0.056
rho −0.086
Flow velocity (z)
V (m/s)
ln(V)
rho 0.31
rho 0.14
rho −0.32
rho 0.14
Width (z)
W (m)
ln(W)
rho 0.17
rho 0.14
rho 0.035
rho 0.14
Bed elevation (z)
BE (m)
rho −0.19
rho −0.029
rho 0.028
rho −0.029
G (m)
Streamflow (z)

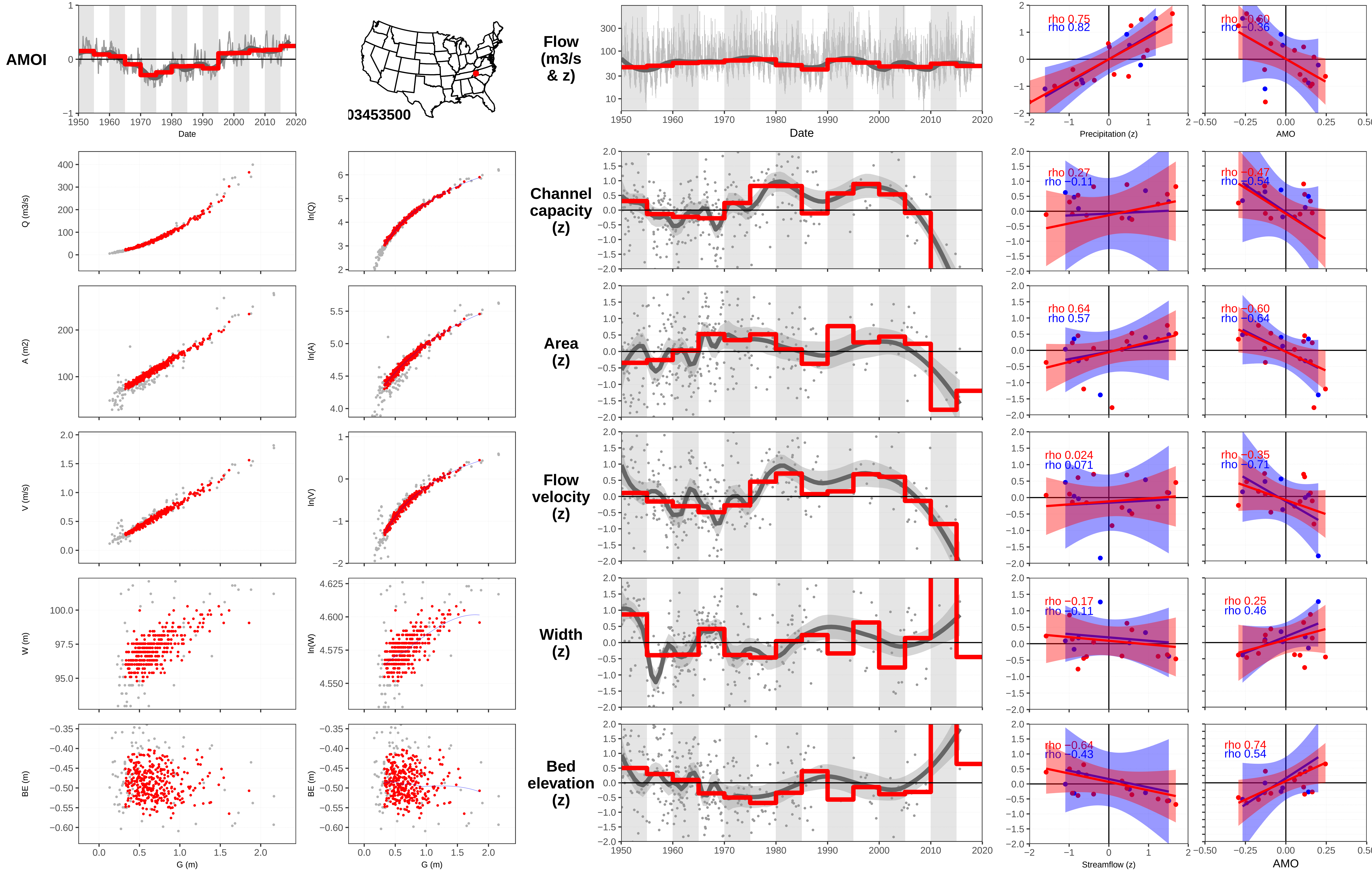

AMOI
03453500
Flow (m3/s & z)
Channel capacity (z)
Area (z)
Flow velocity (z)
Width (z)
Bed elevation (z)
Date
Precipitation (z)
AMO
Streamflow (z)
G (m)
Q (m3/s)
ln(Q)
A (m2)
ln(A)
V (m/s)
ln(V)
W (m)
ln(W)
BE (m)
rho 0.75
rho 0.82
rho −0.60
rho −0.36
rho 0.27
rho −0.11
rho −0.47
rho −0.54
rho 0.64
rho 0.57
rho −0.60
rho −0.64
rho 0.024
rho 0.071
rho −0.35
rho −0.71
rho −0.17
rho −0.11
rho 0.25
rho 0.46
rho −0.64
rho −0.43
rho 0.74
rho 0.54

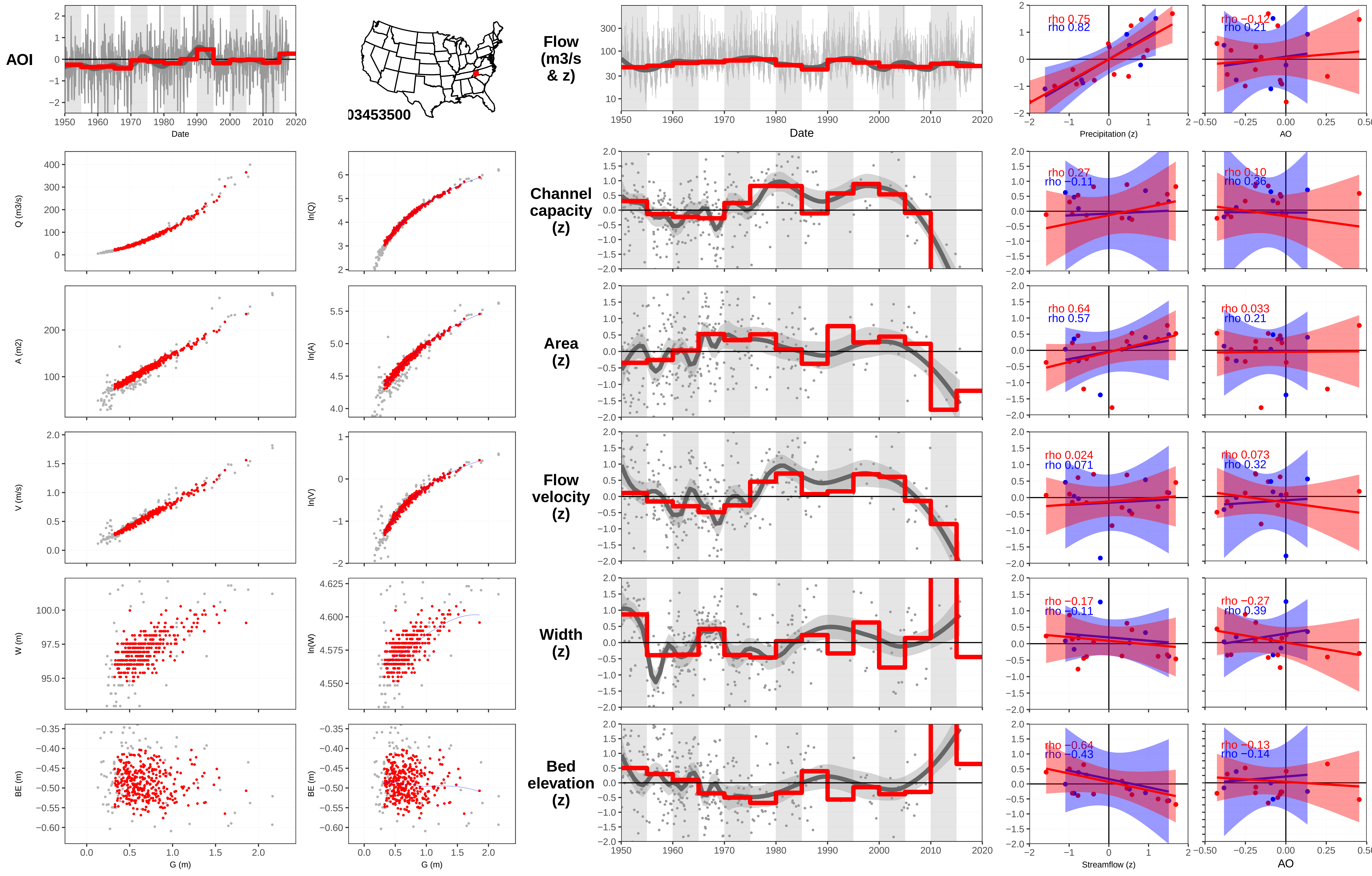

AOI
Date
03453500
Flow (m3/s & z)
Date
rho 0.75
rho 0.82
Precipitation (z)
rho −0.12
rho 0.21
AO
Q (m3/s)
ln(Q)
Channel capacity (z)
rho 0.27
rho −0.11
rho 0.10
rho 0.36
A (m2)
ln(A)
Area (z)
rho 0.64
rho 0.57
rho 0.033
rho 0.21
V (m/s)
ln(V)
Flow velocity (z)
rho 0.024
rho 0.071
rho 0.073
rho 0.32
W (m)
ln(W)
Width (z)
rho −0.17
rho −0.11
rho −0.27
rho 0.39
BE (m)
BE (m)
Bed elevation (z)
rho −0.64
rho −0.43
rho −0.13
rho −0.14
G (m)
G (m)
Streamflow (z)
AO

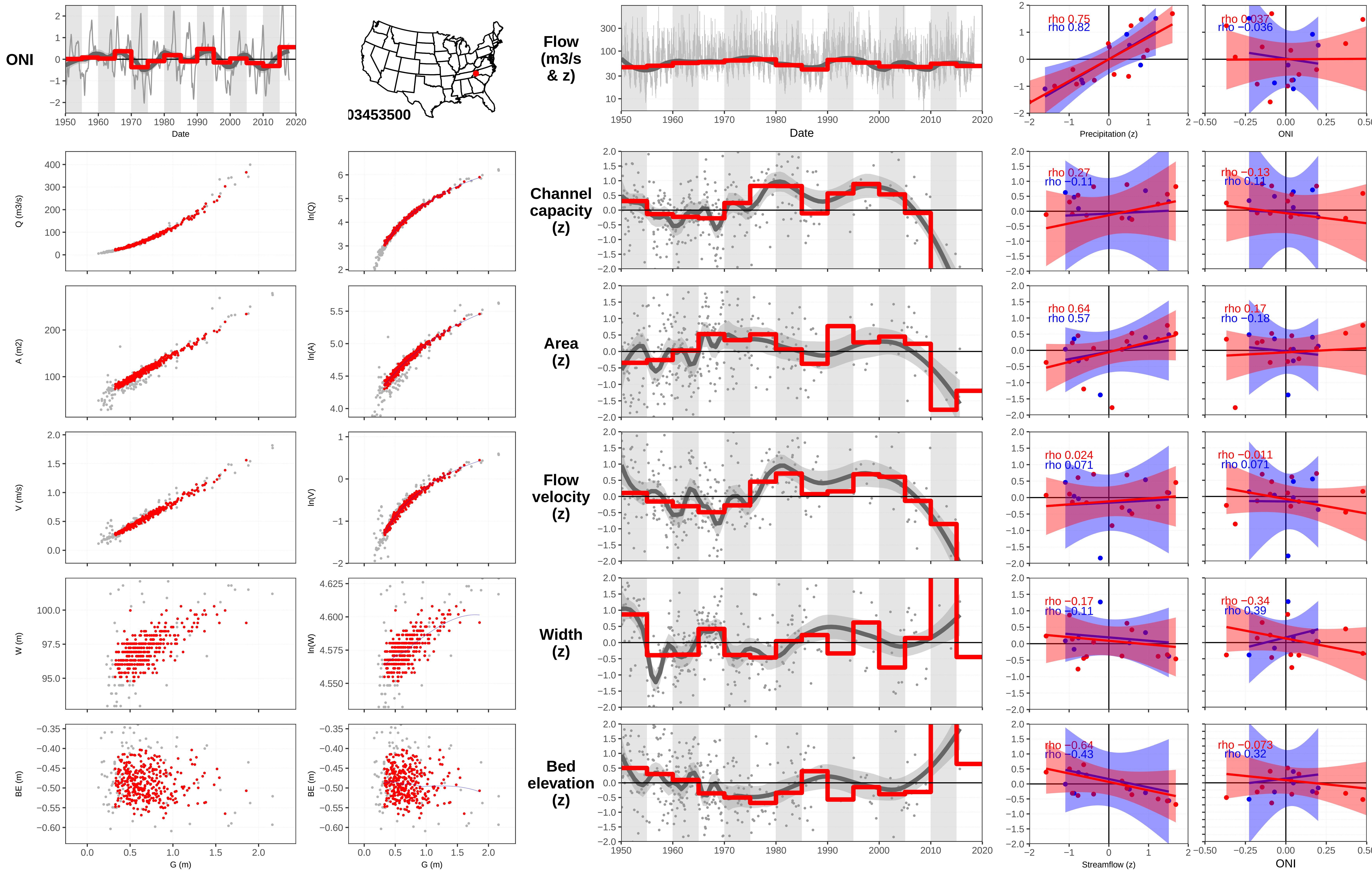
ONI
03453500
Flow (m3/s & z)
Channel capacity (z)
Area (z)
Flow velocity (z)
Width (z)
Bed elevation (z)
Date
Precipitation (z)
Streamflow (z)
ONI
Q (m3/s)
ln(Q)
A (m2)
ln(A)
V (m/s)
ln(V)
W (m)
ln(W)
BE (m)
G (m)
rho 0.75
rho 0.82
rho 0.037
rho −0.036
rho 0.27
rho −0.11
rho −0.13
rho 0.11
rho 0.64
rho 0.57
rho 0.17
rho −0.18
rho 0.024
rho 0.071
rho −0.011
rho 0.071
rho −0.17
rho −0.11
rho −0.34
rho 0.39
rho −0.64
rho −0.43
rho −0.073
rho 0.32

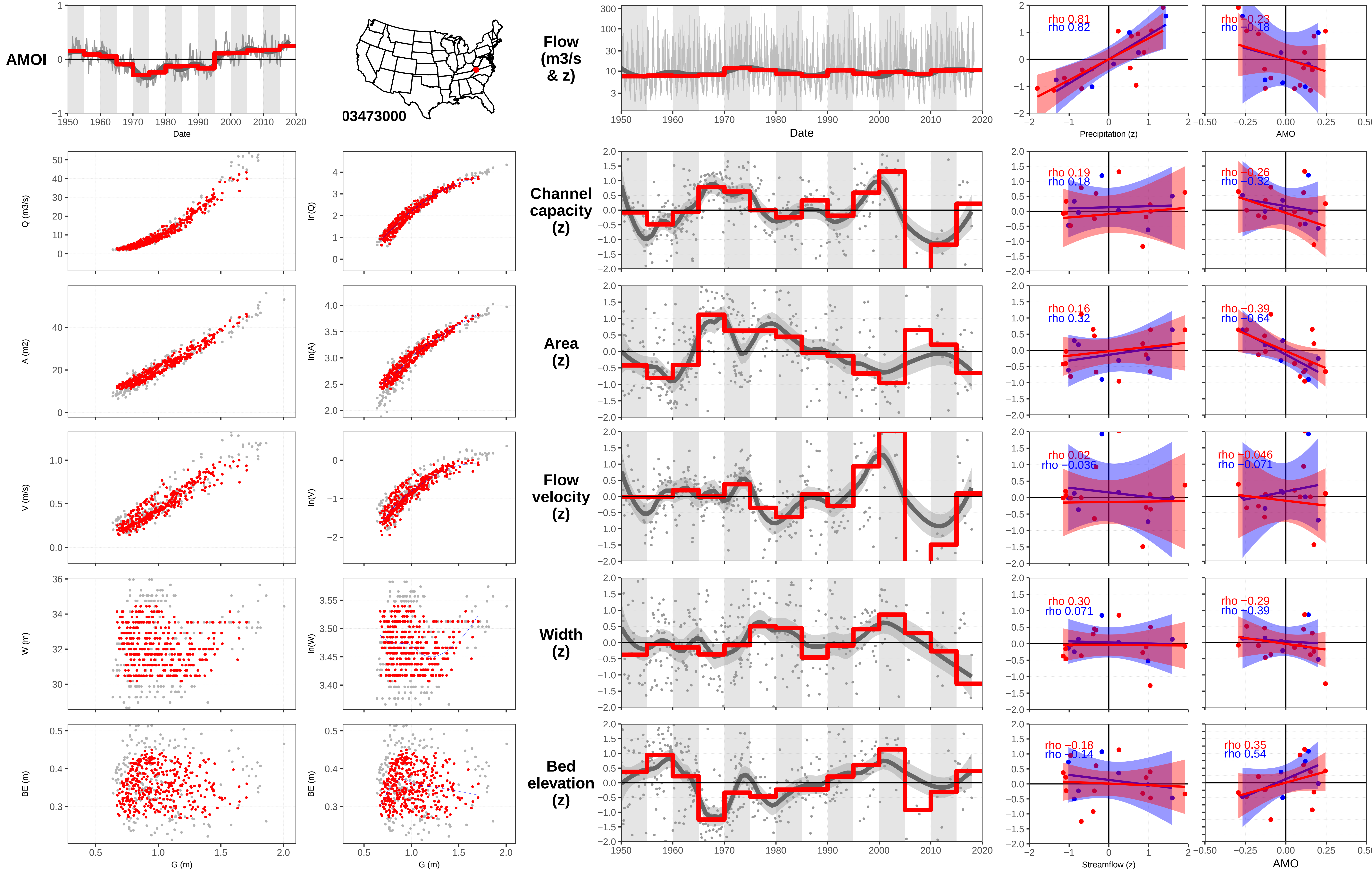

AMOI
03473000
Flow (m3/s & z)
Channel capacity (z)
Area (z)
Flow velocity (z)
Width (z)
Bed elevation (z)
Date
Precipitation (z)
Streamflow (z)
AMO
Q (m3/s)
ln(Q)
A (m2)
ln(A)
V (m/s)
ln(V)
W (m)
ln(W)
BE (m)
G (m)
rho 0.81
rho 0.82
rho −0.23
rho −0.18
rho 0.19
rho 0.18
rho −0.26
rho −0.32
rho 0.16
rho 0.32
rho −0.39
rho −0.64
rho 0.02
rho −0.036
rho −0.046
rho −0.071
rho 0.30
rho 0.071
rho −0.29
rho −0.39
rho −0.18
rho −0.14
rho 0.35
rho 0.54

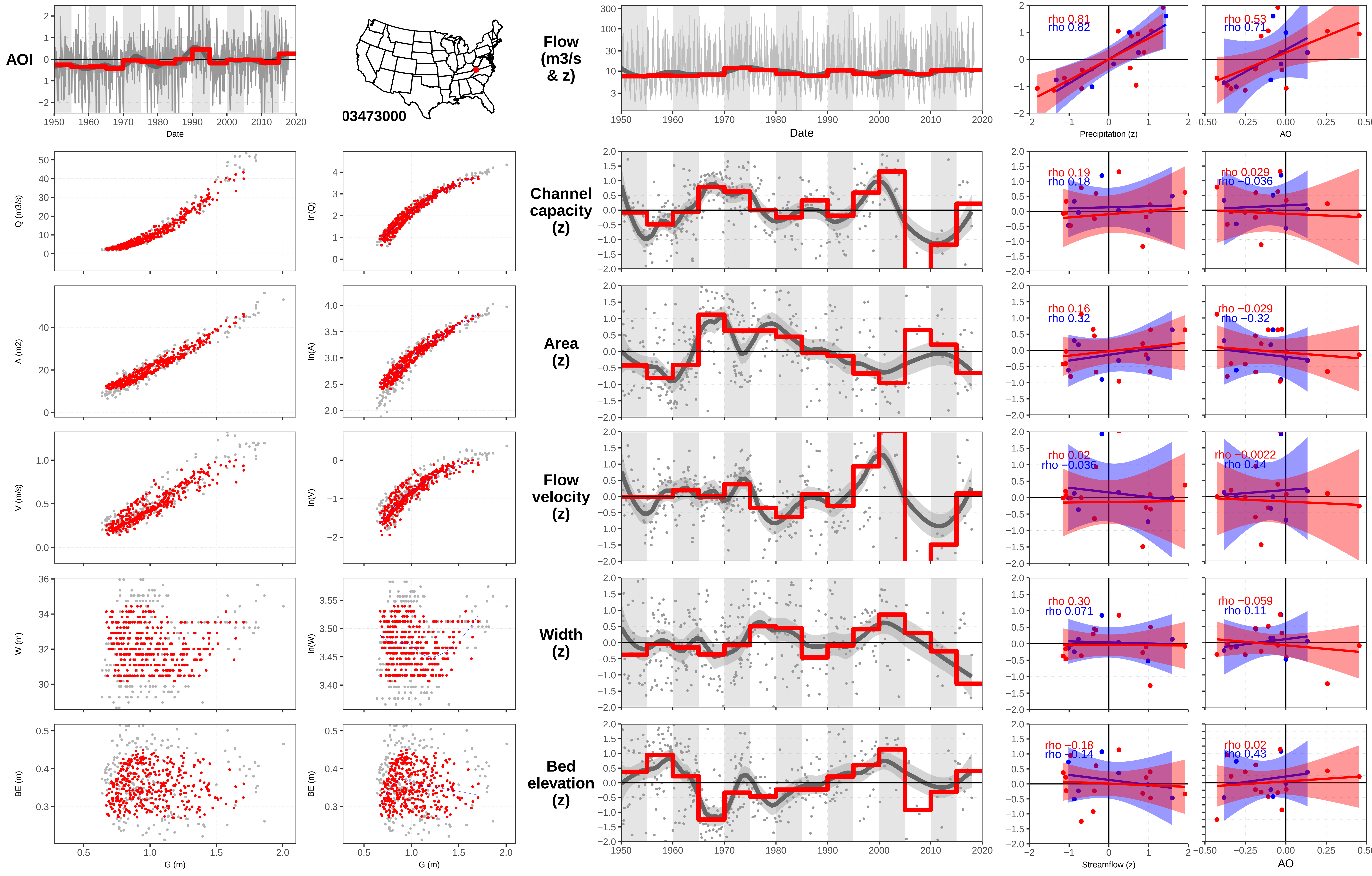
AOI
03473000
Flow (m3/s & z)
Channel capacity (z)
Area (z)
Flow velocity (z)
Width (z)
Bed elevation (z)
Date
Precipitation (z)
AO
Streamflow (z)
Q (m3/s)
ln(Q)
A (m2)
ln(A)
V (m/s)
ln(V)
W (m)
ln(W)
BE (m)
G (m)
rho 0.81
rho 0.82
rho 0.53
rho 0.71
rho 0.19
rho 0.18
rho 0.029
rho −0.036
rho 0.16
rho 0.32
rho −0.029
rho −0.32
rho 0.02
rho −0.036
rho −0.0022
rho 0.14
rho 0.30
rho 0.071
rho −0.059
rho 0.11
rho −0.18
rho −0.14
rho 0.02
rho 0.43

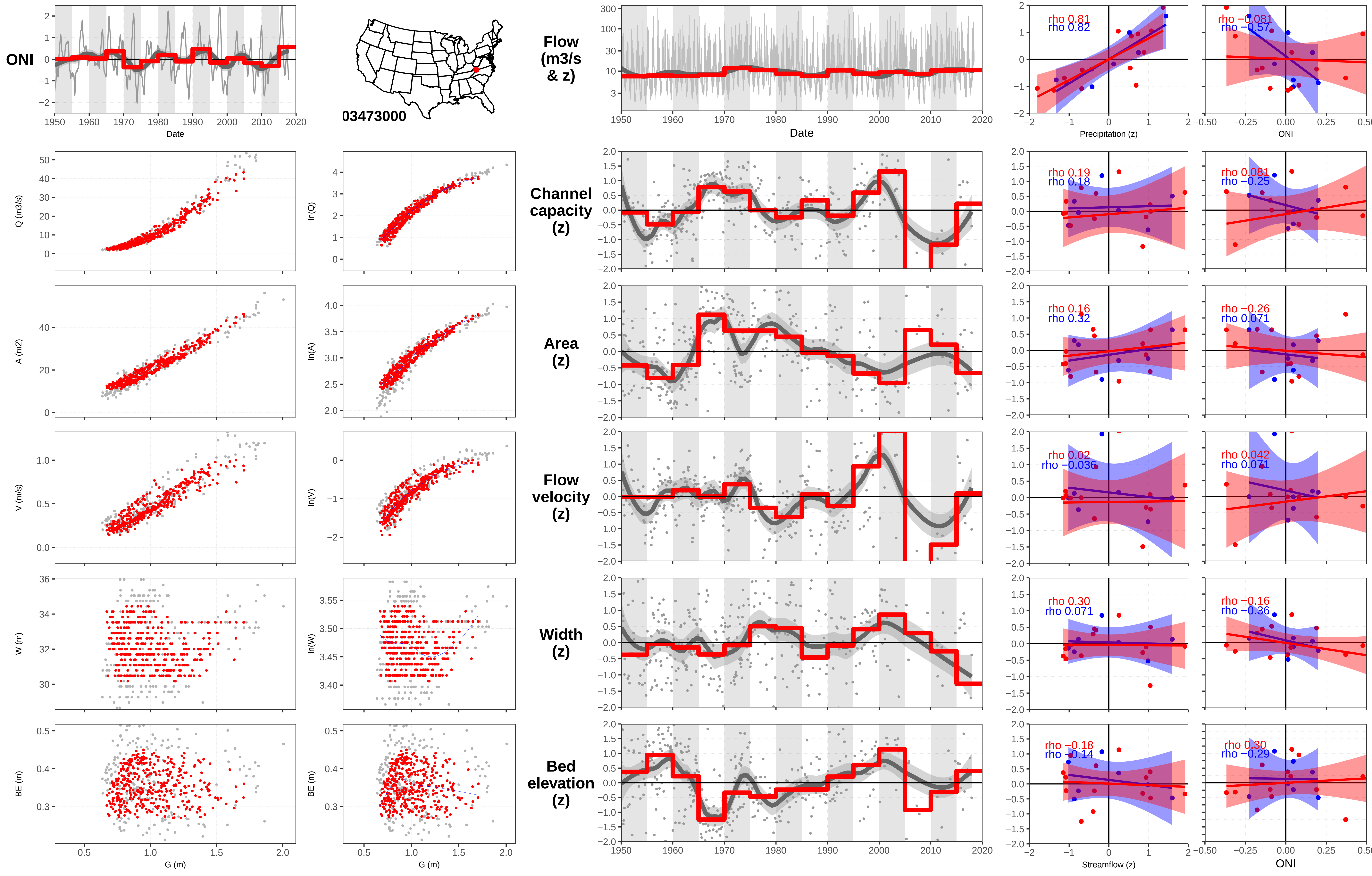

ONI
Date
03473000
Flow (m3/s & z)
Date
Precipitation (z)
ONI
rho 0.81
rho 0.82
rho −0.081
rho −0.57
Channel capacity (z)
rho 0.19
rho 0.18
rho 0.081
rho −0.25
Area (z)
rho 0.16
rho 0.32
rho −0.26
rho 0.071
Flow velocity (z)
rho 0.02
rho −0.036
rho 0.042
rho 0.071
Width (z)
rho 0.30
rho 0.071
rho −0.16
rho −0.36
Bed elevation (z)
rho −0.18
rho −0.14
rho 0.30
rho −0.29
Q (m3/s)
ln(Q)
A (m2)
ln(A)
V (m/s)
ln(V)
W (m)
ln(W)
BE (m)
G (m)
Streamflow (z)

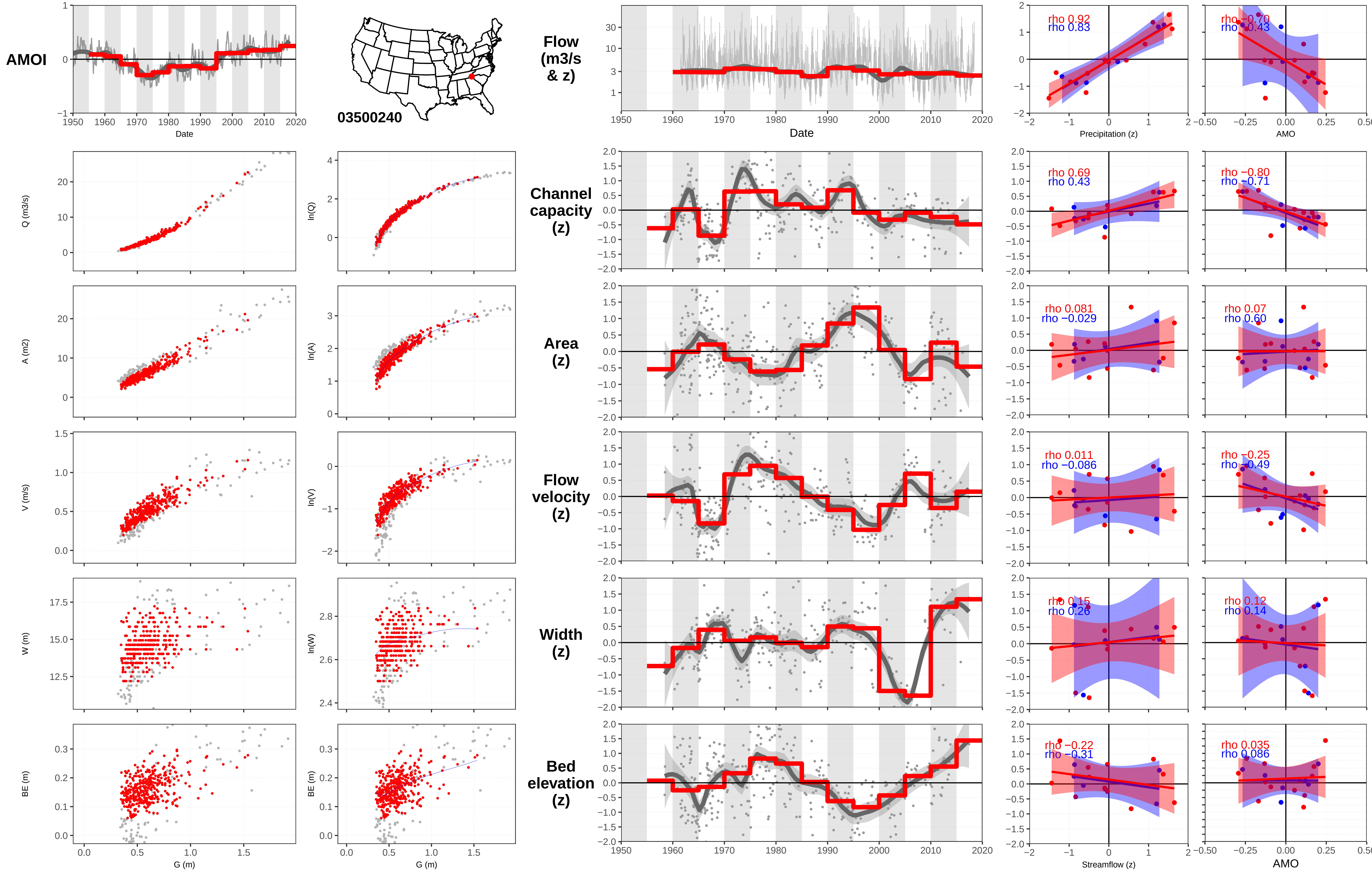
AMOI
03500240
Flow (m3/s & z)
Channel capacity (z)
Area (z)
Flow velocity (z)
Width (z)
Bed elevation (z)
Date
Precipitation (z)
Streamflow (z)
AMO
Q (m3/s)
ln(Q)
A (m2)
ln(A)
V (m/s)
ln(V)
W (m)
ln(W)
BE (m)
G (m)
rho 0.92
rho 0.83
rho −0.70
rho −0.43
rho 0.69
rho 0.43
rho −0.80
rho −0.71
rho 0.081
rho −0.029
rho 0.07
rho 0.60
rho 0.011
rho −0.086
rho −0.25
rho −0.49
rho 0.15
rho 0.26
rho 0.12
rho 0.14
rho −0.22
rho −0.31
rho 0.035
rho 0.086

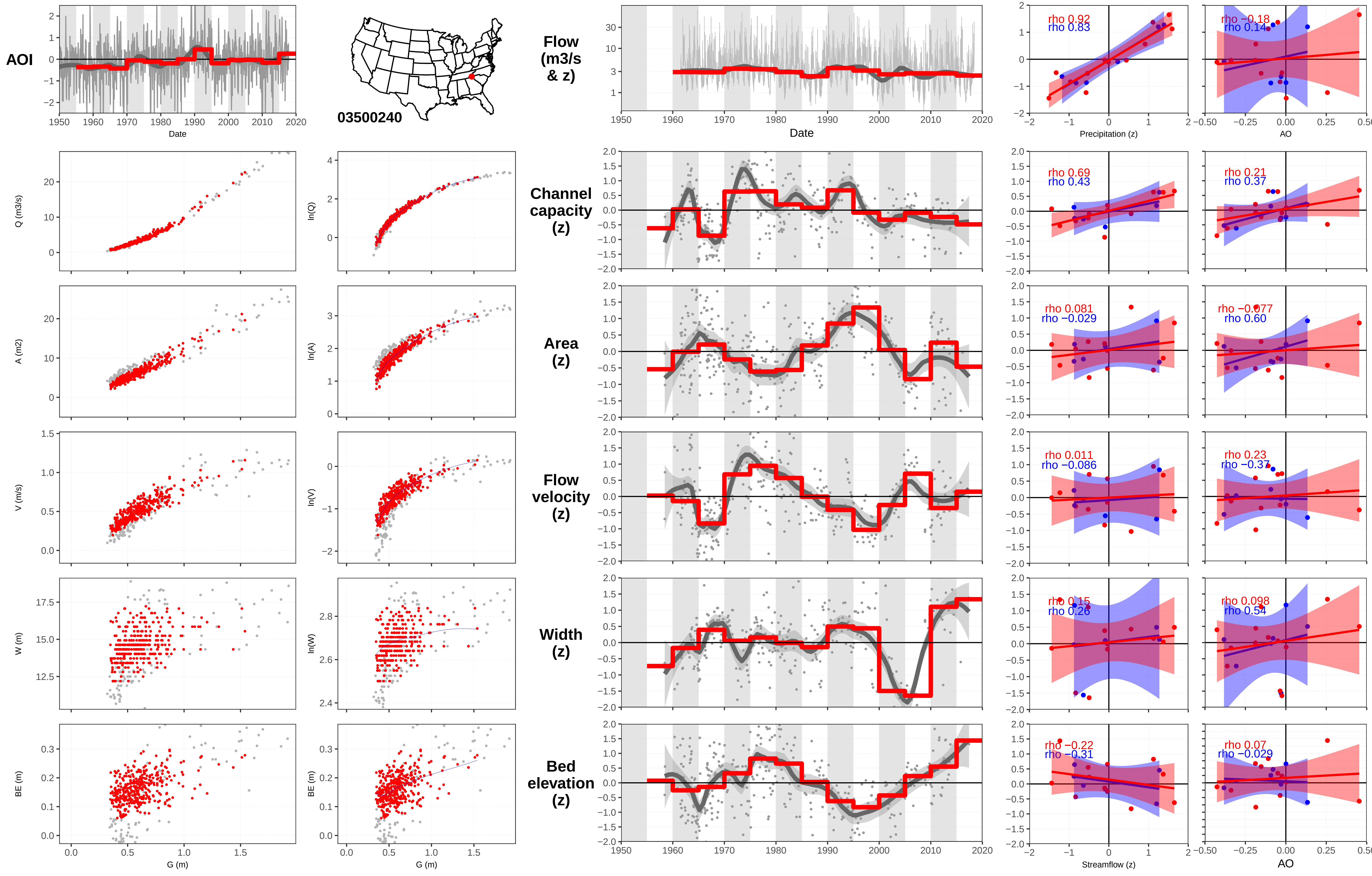
AOI
03500240
Flow (m3/s & z)
Channel capacity (z)
Area (z)
Flow velocity (z)
Width (z)
Bed elevation (z)
Date
Precipitation (z)
Streamflow (z)
AO
Q (m3/s)
ln(Q)
A (m2)
ln(A)
V (m/s)
ln(V)
W (m)
ln(W)
BE (m)
G (m)
rho 0.92
rho 0.83
rho −0.18
rho 0.14
rho 0.69
rho 0.43
rho 0.21
rho 0.37
rho 0.081
rho −0.029
rho −0.077
rho 0.60
rho 0.011
rho −0.086
rho 0.23
rho −0.37
rho 0.15
rho 0.26
rho 0.098
rho 0.54
rho −0.22
rho −0.31
rho 0.07
rho −0.029

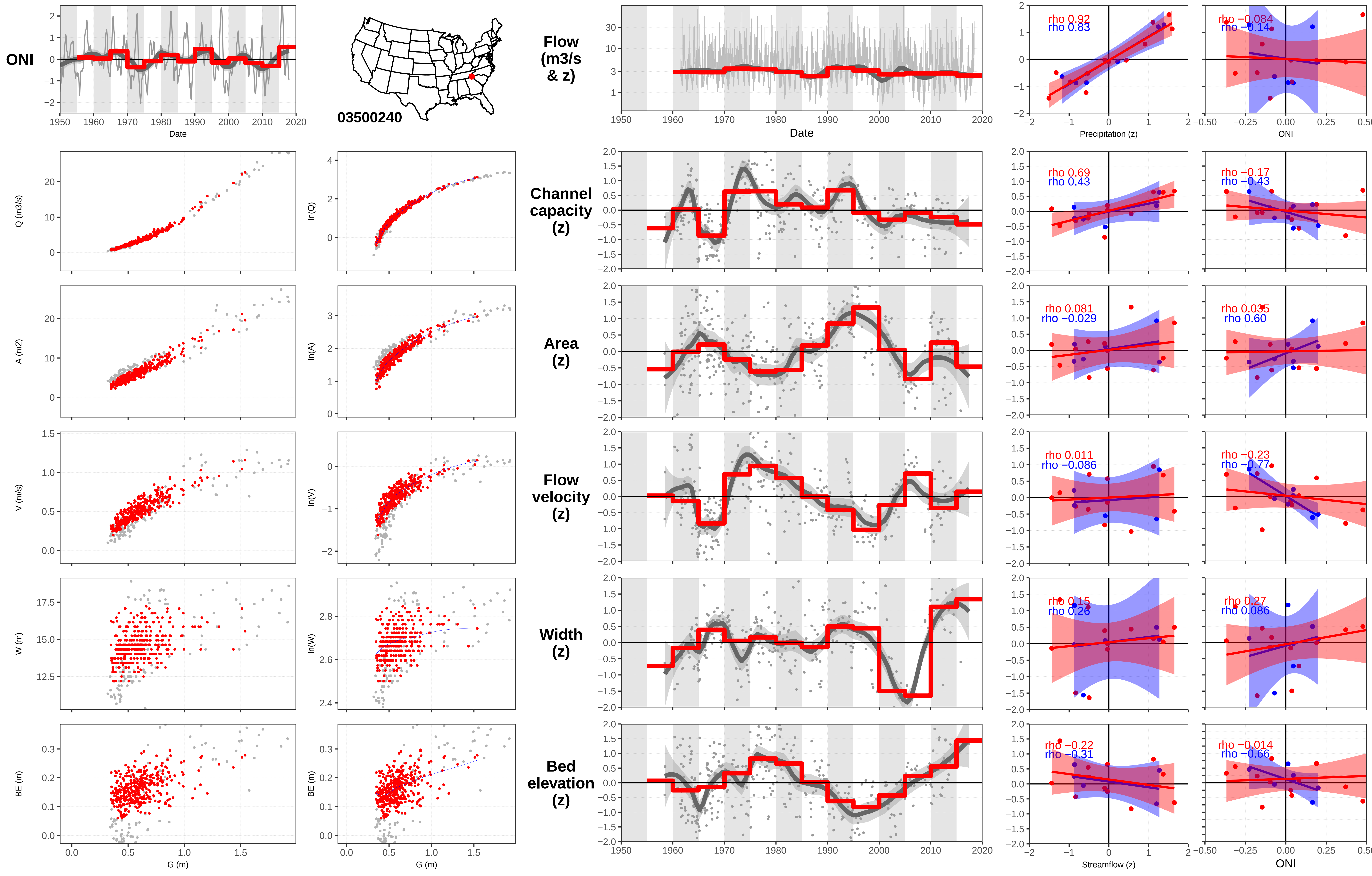

ONI
03500240
Flow (m3/s & z)
Channel capacity (z)
Area (z)
Flow velocity (z)
Width (z)
Bed elevation (z)
Date
Precipitation (z)
Streamflow (z)
ONI
G (m)
Q (m3/s)
ln(Q)
A (m2)
ln(A)
V (m/s)
ln(V)
W (m)
ln(W)
BE (m)
rho 0.92
rho 0.83
rho −0.084
rho −0.14
rho 0.69
rho 0.43
rho −0.17
rho −0.43
rho 0.081
rho −0.029
rho 0.035
rho 0.60
rho 0.011
rho −0.086
rho −0.23
rho −0.77
rho 0.15
rho 0.26
rho 0.27
rho 0.086
rho −0.22
rho −0.31
rho −0.014
rho −0.66

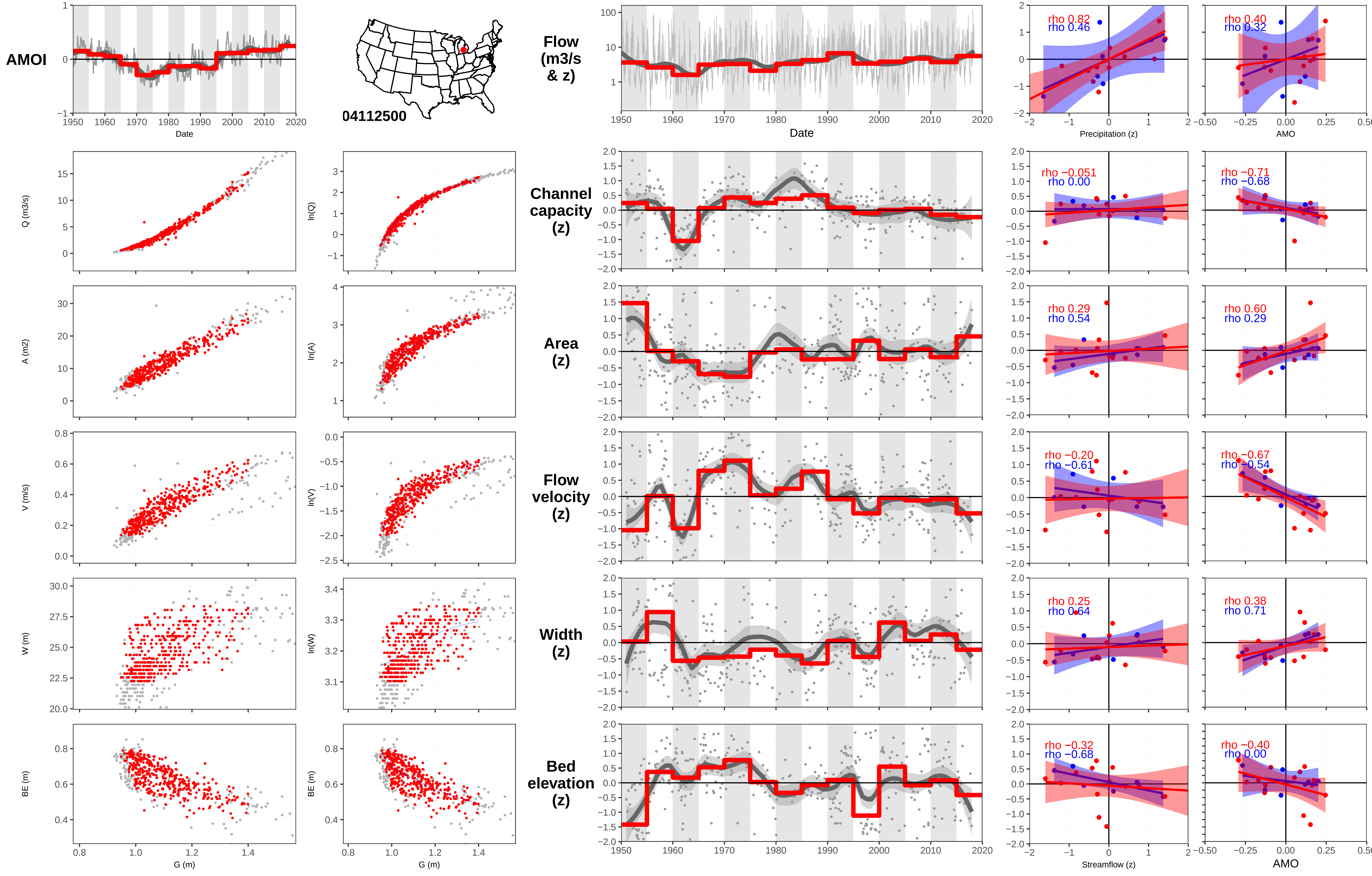

AMOI
04112500
Flow (m3/s & z)
Channel capacity (z)
Area (z)
Flow velocity (z)
Width (z)
Bed elevation (z)
Date
Precipitation (z)
AMO
Streamflow (z)
G (m)
Q (m3/s)
ln(Q)
A (m2)
ln(A)
V (m/s)
ln(V)
W (m)
ln(W)
BE (m)
rho 0.82
rho 0.46
rho 0.40
rho 0.32
rho −0.051
rho 0.00
rho −0.71
rho −0.68
rho 0.29
rho 0.54
rho 0.60
rho 0.29
rho −0.20
rho −0.61
rho −0.67
rho −0.54
rho 0.25
rho 0.64
rho 0.38
rho 0.71
rho −0.32
rho −0.68
rho −0.40
rho 0.00

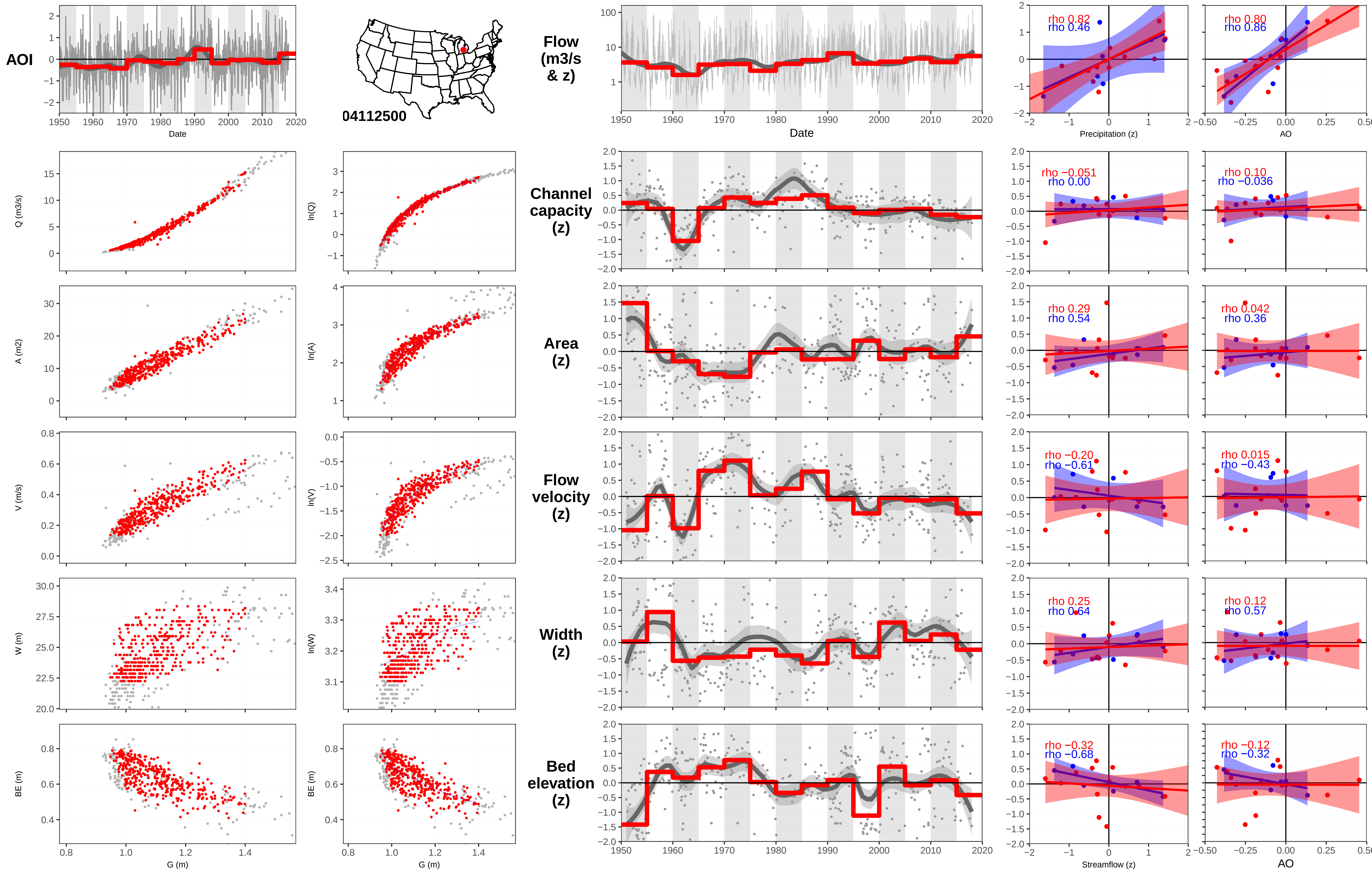

AOI
04112500
Flow (m3/s & z)
Channel capacity (z)
Area (z)
Flow velocity (z)
Width (z)
Bed elevation (z)
Date
Precipitation (z)
Streamflow (z)
AO
G (m)
Q (m3/s)
ln(Q)
A (m2)
ln(A)
V (m/s)
ln(V)
W (m)
ln(W)
BE (m)
rho 0.82
rho 0.46
rho 0.80
rho 0.86
rho −0.051
rho 0.00
rho 0.10
rho −0.036
rho 0.29
rho 0.54
rho 0.042
rho 0.36
rho −0.20
rho −0.61
rho 0.015
rho −0.43
rho 0.25
rho 0.64
rho 0.12
rho 0.57
rho −0.32
rho −0.68
rho −0.12
rho −0.32

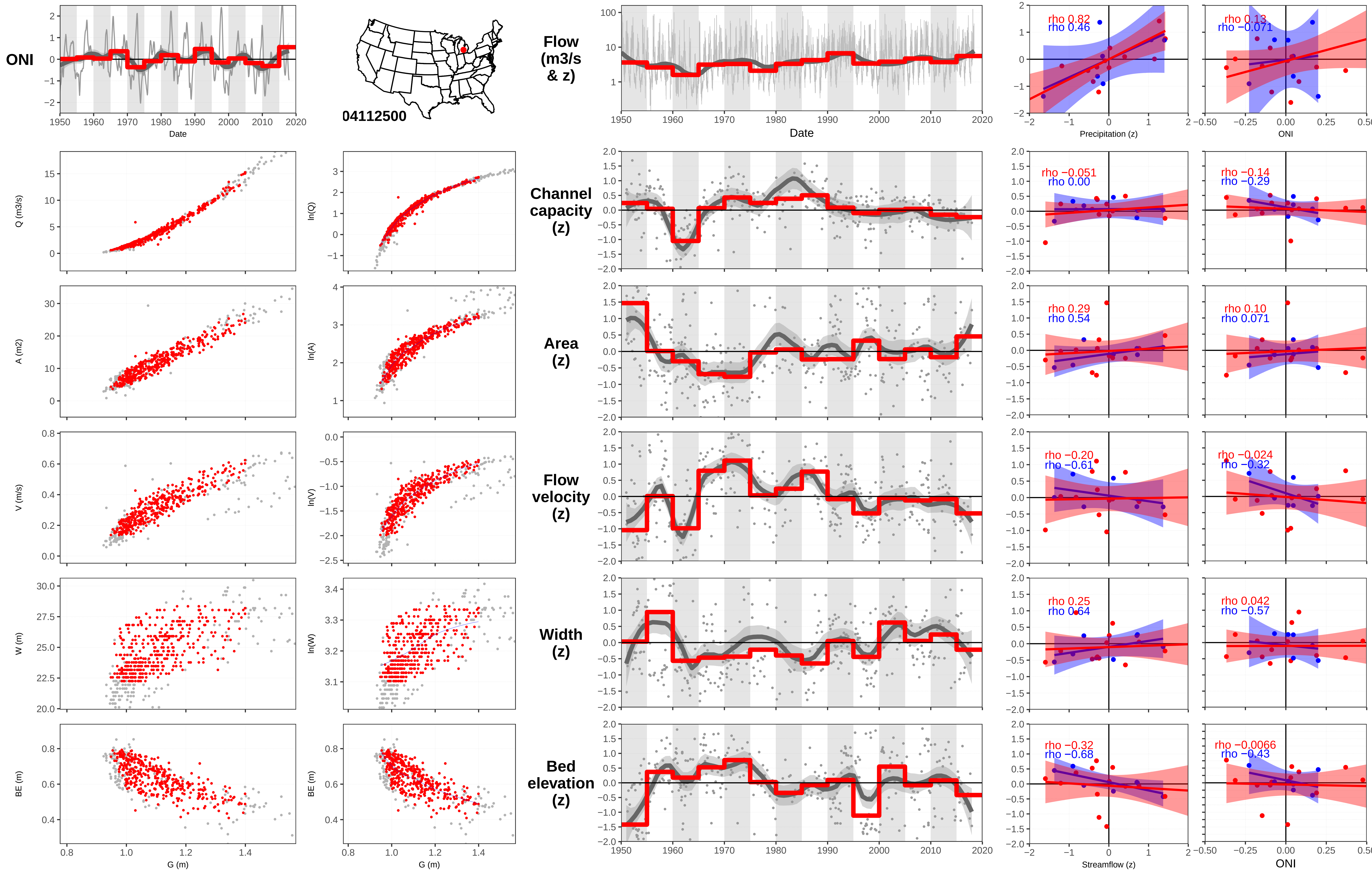

ONI
Date
04112500
Flow (m3/s & z)
Date
Precipitation (z)
ONI
rho 0.82
rho 0.46
rho 0.13
rho −0.071
Q (m3/s)
ln(Q)
Channel capacity (z)
rho −0.051
rho 0.00
rho −0.14
rho −0.29
A (m2)
ln(A)
Area (z)
rho 0.29
rho 0.54
rho 0.10
rho 0.071
V (m/s)
ln(V)
Flow velocity (z)
rho −0.20
rho −0.61
rho −0.024
rho −0.32
W (m)
ln(W)
Width (z)
rho 0.25
rho 0.64
rho 0.042
rho −0.57
BE (m)
BE (m)
Bed elevation (z)
rho −0.32
rho −0.68
rho −0.0066
rho −0.43
G (m)
G (m)
Streamflow (z)
ONI

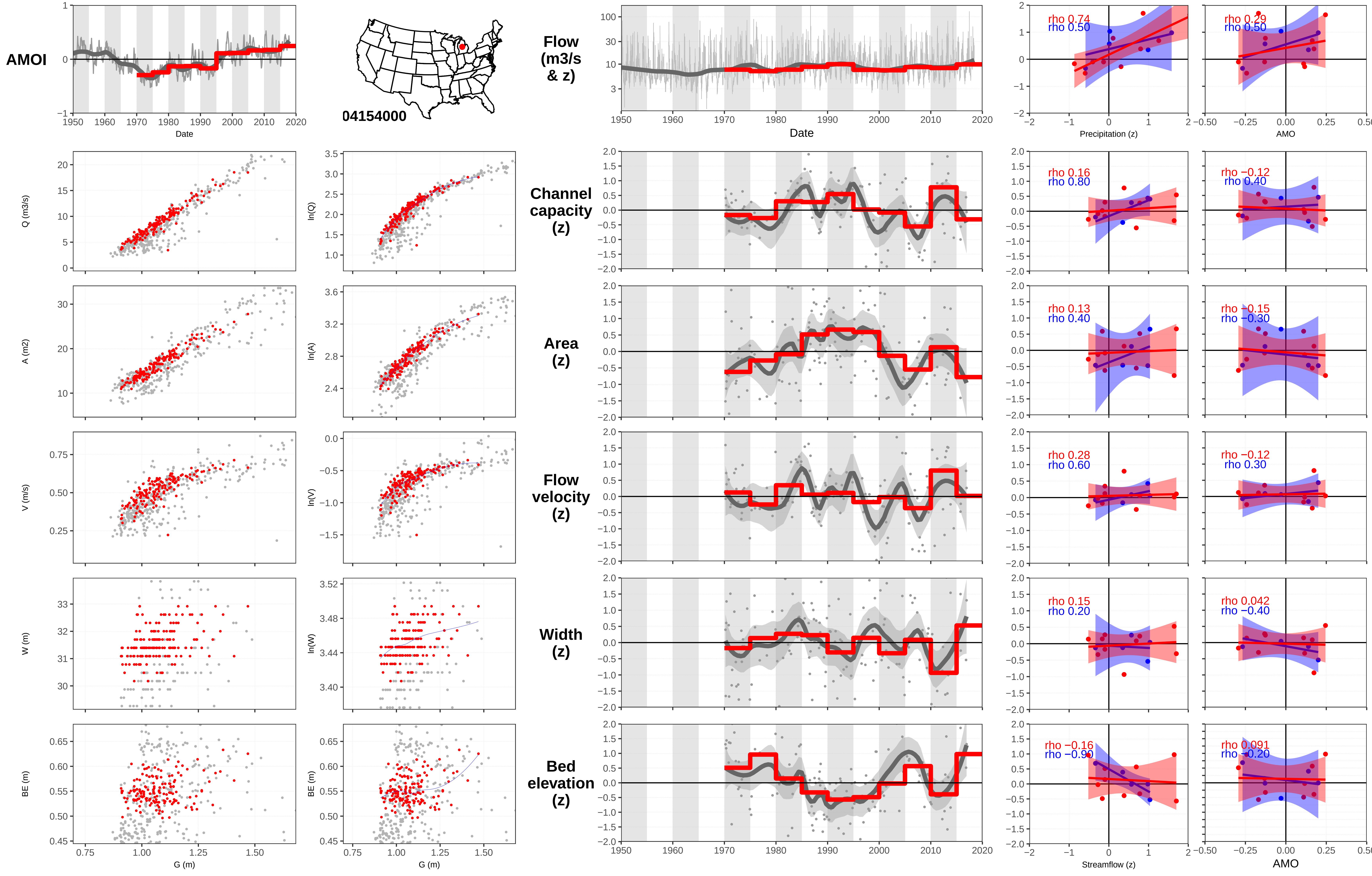

AMOI
04154000
Flow (m3/s & z)
Channel capacity (z)
Area (z)
Flow velocity (z)
Width (z)
Bed elevation (z)
Date
Precipitation (z)
Streamflow (z)
AMO
G (m)
Q (m3/s)
ln(Q)
A (m2)
ln(A)
V (m/s)
ln(V)
W (m)
ln(W)
BE (m)
rho 0.74
rho 0.50
rho 0.29
rho 0.50
rho 0.16
rho 0.80
rho −0.12
rho 0.40
rho 0.13
rho 0.40
rho −0.15
rho −0.30
rho 0.28
rho 0.60
rho −0.12
rho 0.30
rho 0.15
rho 0.20
rho 0.042
rho −0.40
rho −0.16
rho −0.90
rho 0.091
rho −0.20

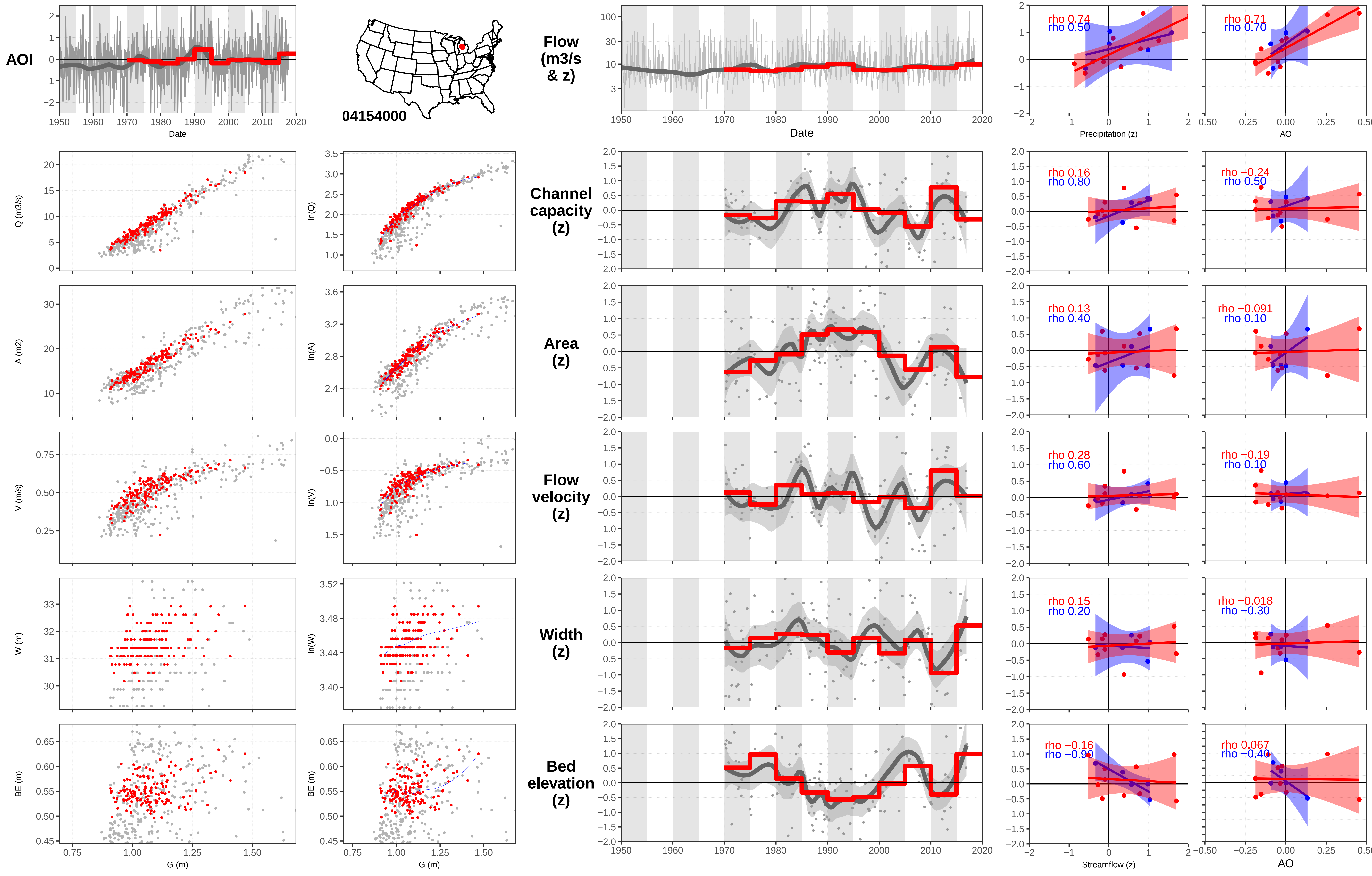

AOI
04154000
Flow (m3/s & z)
Channel capacity (z)
Area (z)
Flow velocity (z)
Width (z)
Bed elevation (z)
Date
Precipitation (z)
AO
Streamflow (z)
G (m)
Q (m3/s)
ln(Q)
A (m2)
ln(A)
V (m/s)
ln(V)
W (m)
ln(W)
BE (m)
rho 0.74
rho 0.50
rho 0.71
rho 0.70
rho 0.16
rho 0.80
rho −0.24
rho 0.50
rho 0.13
rho 0.40
rho −0.091
rho 0.10
rho 0.28
rho 0.60
rho −0.19
rho 0.10
rho 0.15
rho 0.20
rho −0.018
rho −0.30
rho −0.16
rho −0.90
rho 0.067
rho −0.40

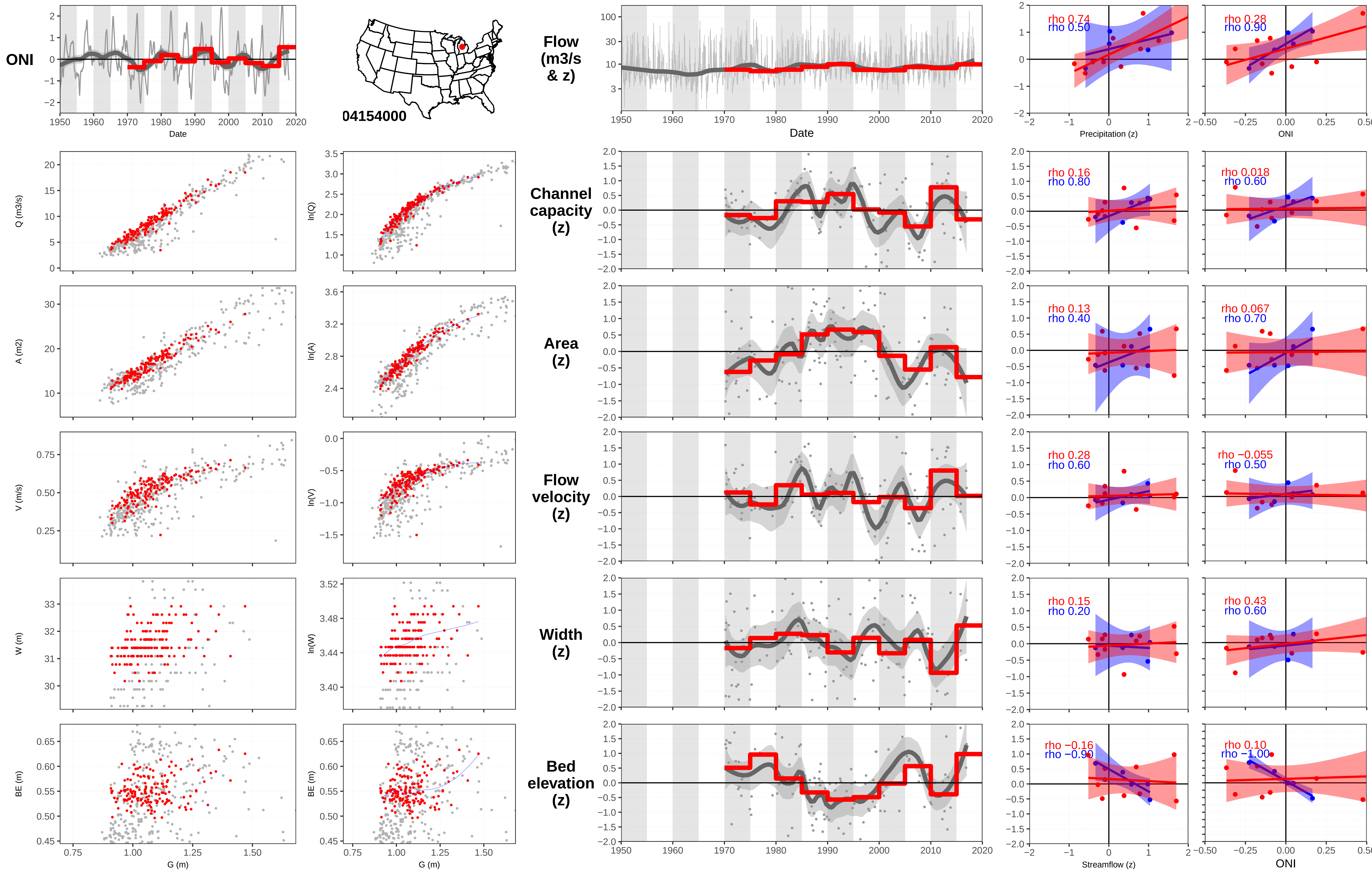
ONI
Date
04154000
Flow (m3/s & z)
Date
rho 0.74
rho 0.50
Precipitation (z)
rho 0.28
rho 0.90
ONI
Q (m3/s)
ln(Q)
Channel capacity (z)
rho 0.16
rho 0.80
rho 0.018
rho 0.60
A (m2)
ln(A)
Area (z)
rho 0.13
rho 0.40
rho 0.067
rho 0.70
V (m/s)
ln(V)
Flow velocity (z)
rho 0.28
rho 0.60
rho −0.055
rho 0.50
W (m)
ln(W)
Width (z)
rho 0.15
rho 0.20
rho 0.43
rho 0.60
BE (m)
BE (m)
Bed elevation (z)
rho −0.16
rho −0.90
rho 0.10
rho −1.00
G (m)
G (m)
Streamflow (z)
ONI

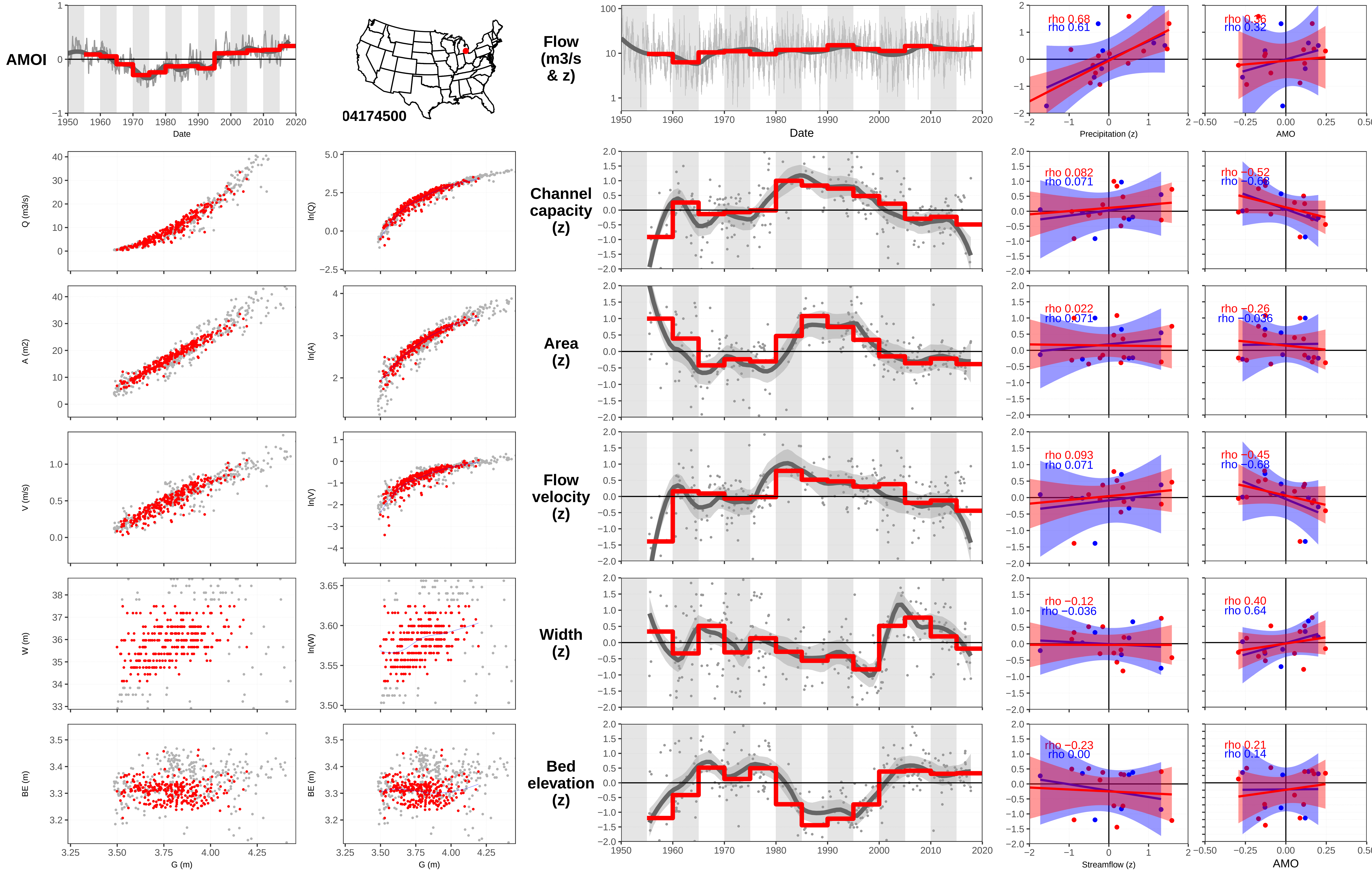

AMOI
04174500
Flow (m3/s & z)
Channel capacity (z)
Area (z)
Flow velocity (z)
Width (z)
Bed elevation (z)
Date
Precipitation (z)
AMO
Streamflow (z)
G (m)
Q (m3/s)
ln(Q)
A (m2)
ln(A)
V (m/s)
ln(V)
W (m)
ln(W)
BE (m)
rho 0.68
rho 0.61
rho 0.36
rho 0.32
rho 0.082
rho 0.071
rho −0.52
rho −0.68
rho 0.022
rho 0.071
rho −0.26
rho −0.036
rho 0.093
rho 0.071
rho −0.45
rho −0.68
rho −0.12
rho −0.036
rho 0.40
rho 0.64
rho −0.23
rho 0.00
rho 0.21
rho 0.14

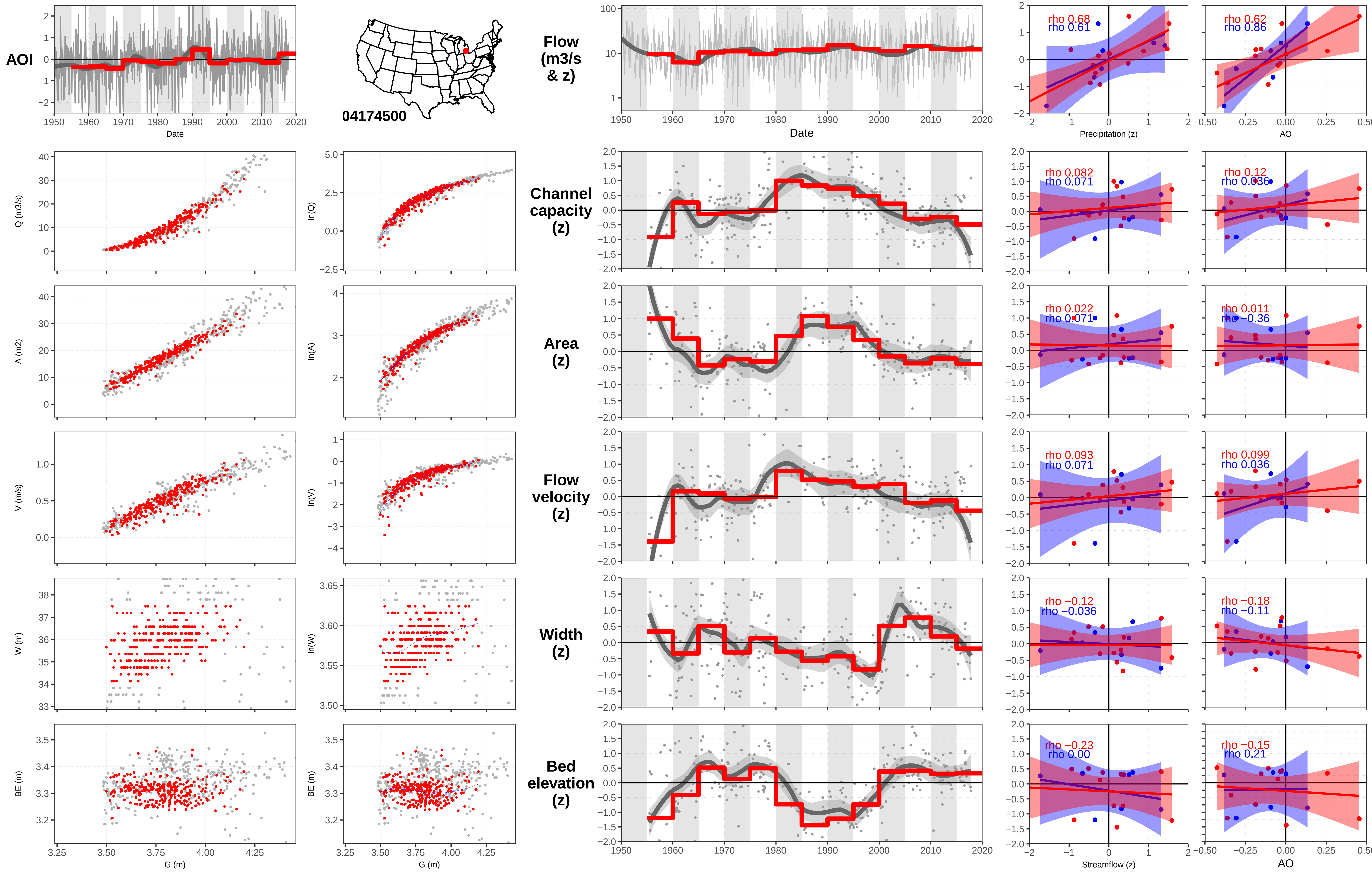

AOI
04174500
Flow (m3/s & z)
Date
Precipitation (z)
AO
rho 0.68
rho 0.61
rho 0.62
rho 0.86
Q (m3/s)
ln(Q)
Channel capacity (z)
rho 0.082
rho 0.071
rho 0.12
rho 0.036
A (m2)
ln(A)
Area (z)
rho 0.022
rho 0.071
rho 0.011
rho −0.36
V (m/s)
ln(V)
Flow velocity (z)
rho 0.093
rho 0.071
rho 0.099
rho 0.036
W (m)
ln(W)
Width (z)
rho −0.12
rho −0.036
rho −0.18
rho −0.11
BE (m)
Bed elevation (z)
rho −0.23
rho 0.00
rho −0.15
rho 0.21
G (m)
Streamflow (z)

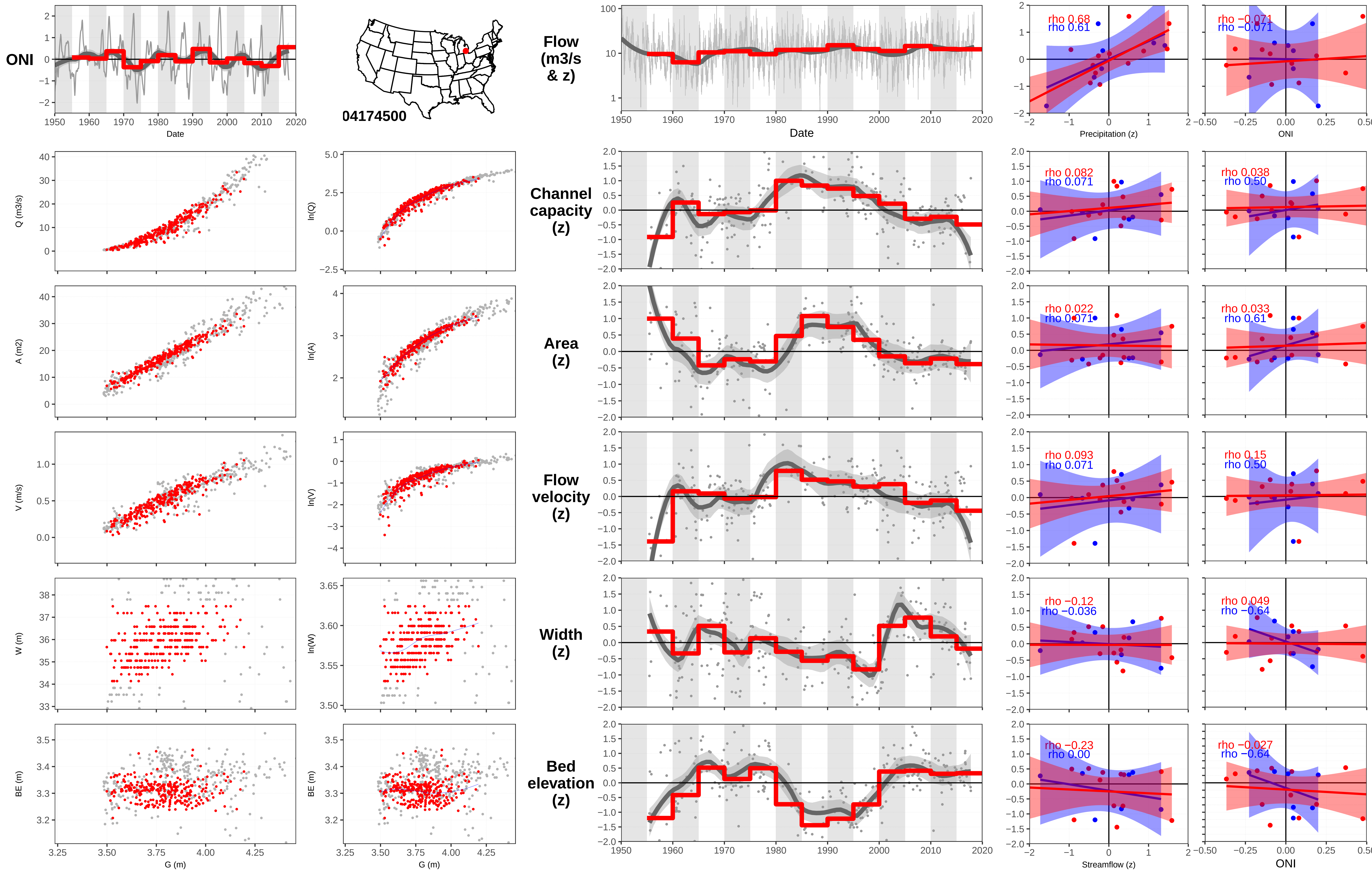

ONI
Date
04174500
Flow (m3/s & z)
Date
Precipitation (z)
ONI
rho 0.68
rho 0.61
rho −0.071
rho −0.071
Q (m3/s)
ln(Q)
Channel capacity (z)
rho 0.082
rho 0.071
rho 0.038
rho 0.50
A (m2)
ln(A)
Area (z)
rho 0.022
rho 0.071
rho 0.033
rho 0.61
V (m/s)
ln(V)
Flow velocity (z)
rho 0.093
rho 0.071
rho 0.15
rho 0.50
W (m)
ln(W)
Width (z)
rho −0.12
rho −0.036
rho 0.049
rho −0.64
BE (m)
Bed elevation (z)
rho −0.23
rho 0.00
rho −0.027
rho −0.64
G (m)
G (m)
Streamflow (z)
ONI

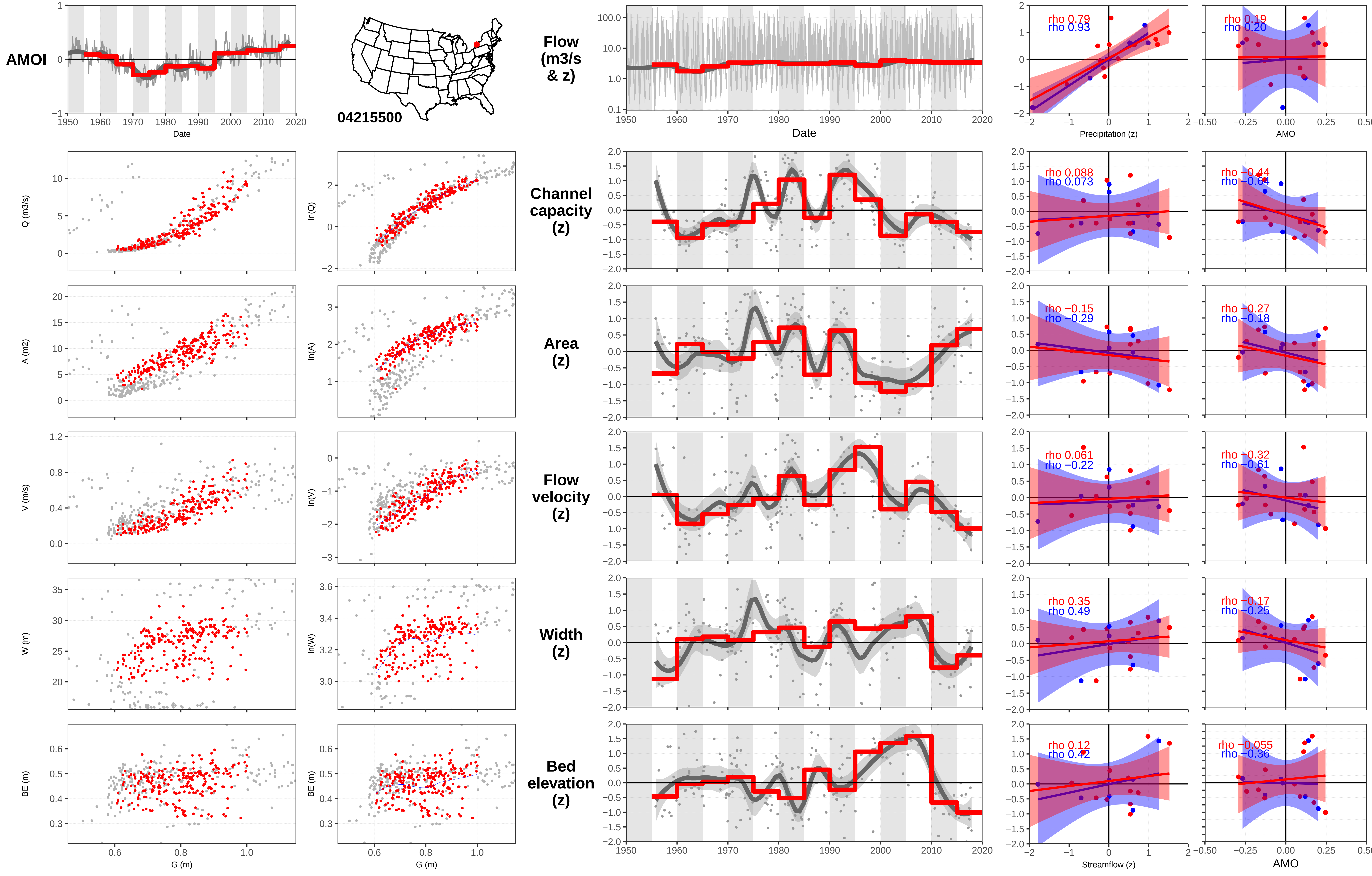

AMOI
04215500
Flow (m3/s & z)
Channel capacity (z)
Area (z)
Flow velocity (z)
Width (z)
Bed elevation (z)
Date
Precipitation (z)
AMO
Streamflow (z)
G (m)
Q (m3/s)
ln(Q)
A (m2)
ln(A)
V (m/s)
ln(V)
W (m)
ln(W)
BE (m)
rho 0.79
rho 0.93
rho 0.19
rho 0.20
rho 0.088
rho 0.073
rho −0.44
rho −0.64
rho −0.15
rho −0.29
rho −0.27
rho −0.18
rho 0.061
rho −0.22
rho −0.32
rho −0.61
rho 0.35
rho 0.49
rho −0.17
rho −0.25
rho 0.12
rho 0.42
rho −0.055
rho −0.36

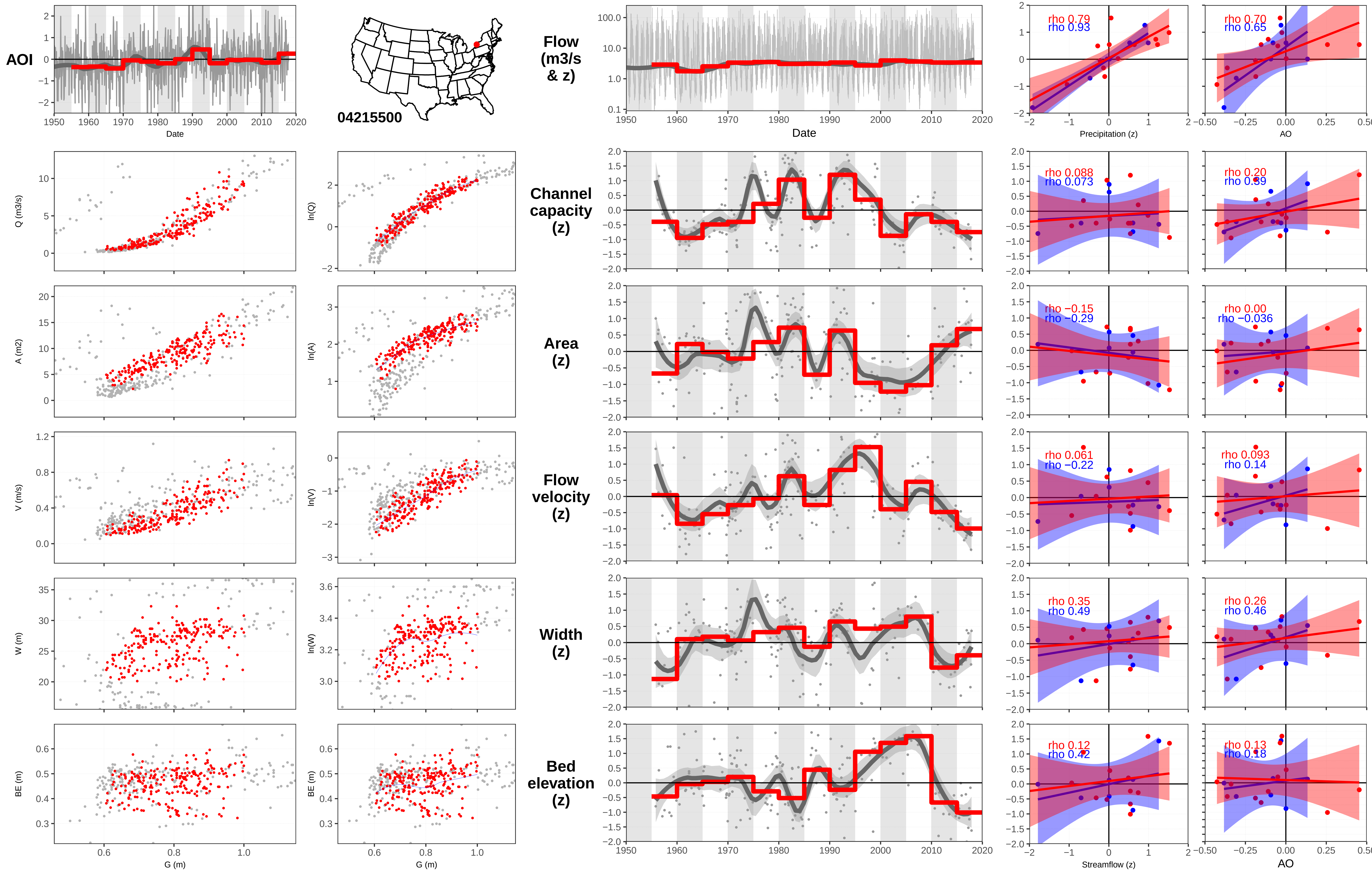

AOI
Date
04215500
Flow (m3/s & z)
Date
Precipitation (z)
AO
rho 0.79
rho 0.93
rho 0.70
rho 0.65
Q (m3/s)
ln(Q)
Channel capacity (z)
rho 0.088
rho 0.073
rho 0.20
rho 0.39
A (m2)
ln(A)
Area (z)
rho −0.15
rho −0.29
rho 0.00
rho −0.036
V (m/s)
ln(V)
Flow velocity (z)
rho 0.061
rho −0.22
rho 0.093
rho 0.14
W (m)
ln(W)
Width (z)
rho 0.35
rho 0.49
rho 0.26
rho 0.46
BE (m)
Bed elevation (z)
rho 0.12
rho 0.42
rho 0.13
rho 0.18
G (m)
G (m)
Streamflow (z)
AO

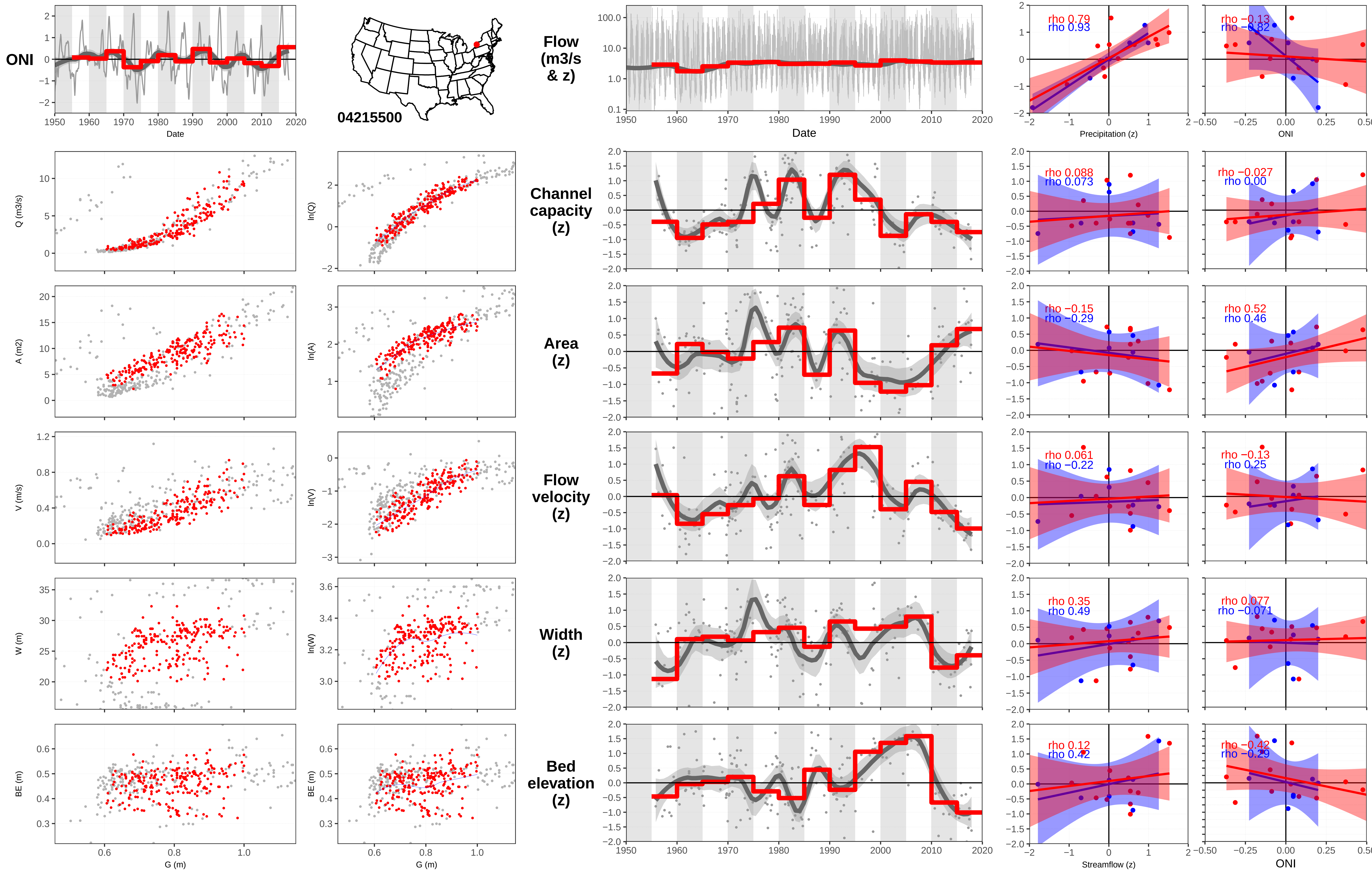

ONI
04215500
Flow (m3/s & z)
Channel capacity (z)
Area (z)
Flow velocity (z)
Width (z)
Bed elevation (z)
Date
Precipitation (z)
Streamflow (z)
ONI
Q (m3/s)
ln(Q)
A (m2)
ln(A)
V (m/s)
ln(V)
W (m)
ln(W)
BE (m)
G (m)
rho 0.79
rho 0.93
rho −0.13
rho −0.82
rho 0.088
rho 0.073
rho −0.027
rho 0.00
rho −0.15
rho −0.29
rho 0.52
rho 0.46
rho 0.061
rho −0.22
rho −0.13
rho 0.25
rho 0.35
rho 0.49
rho 0.077
rho −0.071
rho 0.12
rho 0.42
rho −0.42
rho −0.29

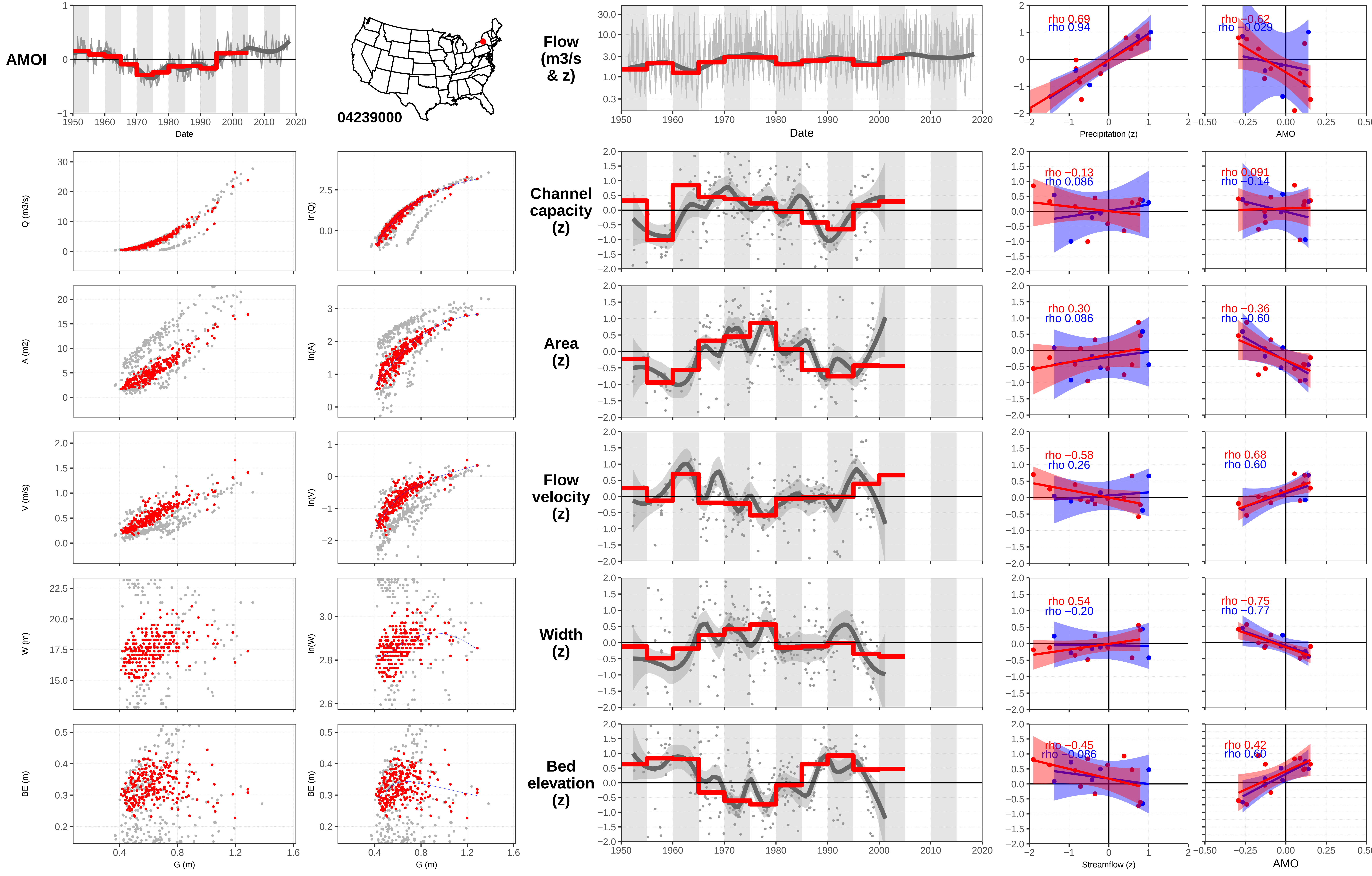

AMOI
04239000
Flow (m3/s & z)
Channel capacity (z)
Area (z)
Flow velocity (z)
Width (z)
Bed elevation (z)
Date
Precipitation (z)
AMO
Streamflow (z)
Q (m3/s)
ln(Q)
A (m2)
ln(A)
V (m/s)
ln(V)
W (m)
ln(W)
BE (m)
G (m)
rho 0.69
rho 0.94
rho −0.62
rho −0.029
rho −0.13
rho 0.086
rho 0.091
rho −0.14
rho 0.30
rho 0.086
rho −0.36
rho −0.60
rho −0.58
rho 0.26
rho 0.68
rho 0.60
rho 0.54
rho −0.20
rho −0.75
rho −0.77
rho −0.45
rho −0.086
rho 0.42
rho 0.60

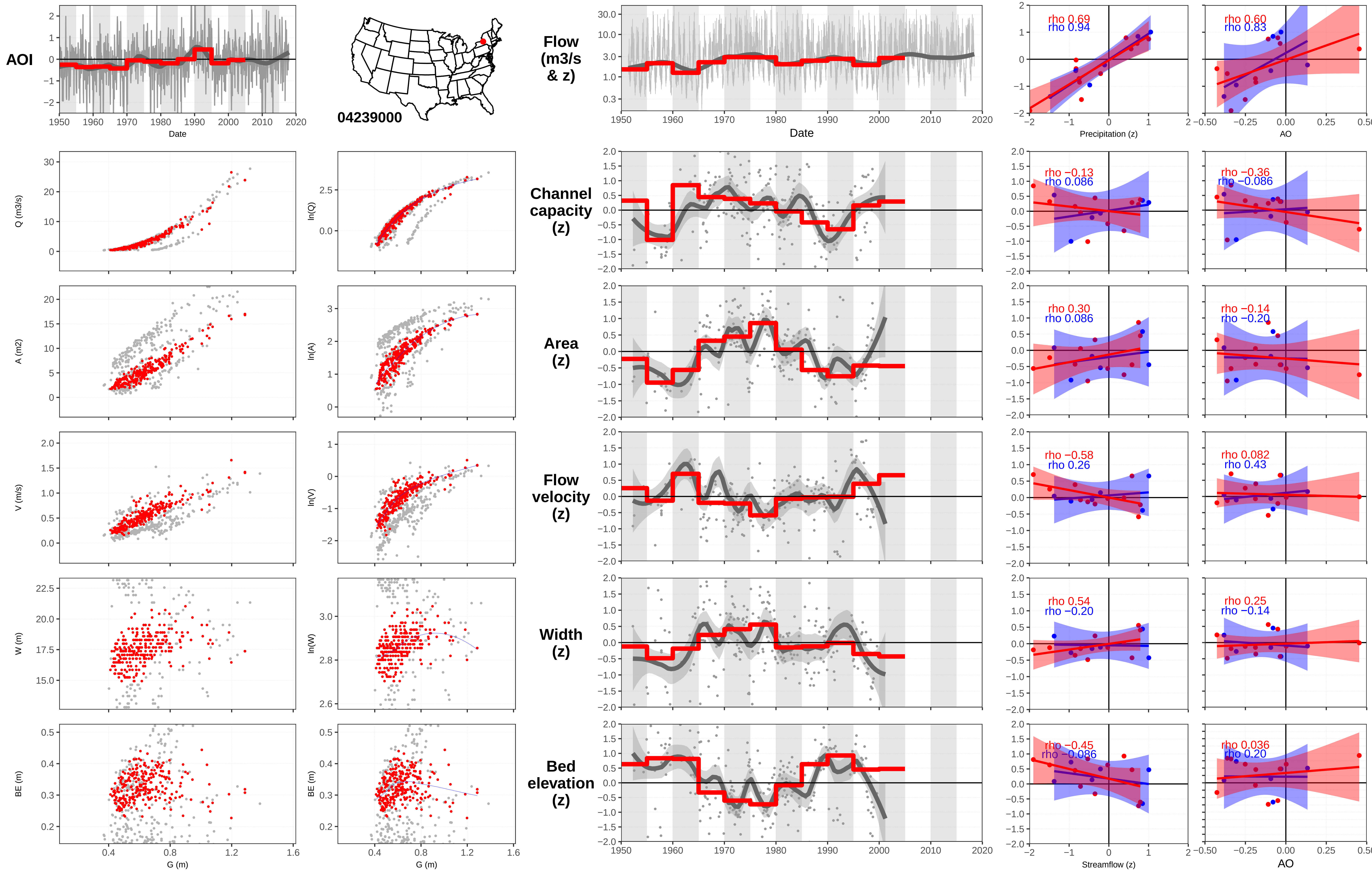

AOI
04239000
Flow (m3/s & z)
Channel capacity (z)
Area (z)
Flow velocity (z)
Width (z)
Bed elevation (z)
Date
Precipitation (z)
AO
Streamflow (z)
G (m)
Q (m3/s)
ln(Q)
A (m2)
ln(A)
V (m/s)
ln(V)
W (m)
ln(W)
BE (m)
rho 0.69
rho 0.94
rho 0.60
rho 0.83
rho −0.13
rho 0.086
rho −0.36
rho −0.086
rho 0.30
rho 0.086
rho −0.14
rho −0.20
rho −0.58
rho 0.26
rho 0.082
rho 0.43
rho 0.54
rho −0.20
rho 0.25
rho −0.14
rho −0.45
rho −0.086
rho 0.036
rho 0.20

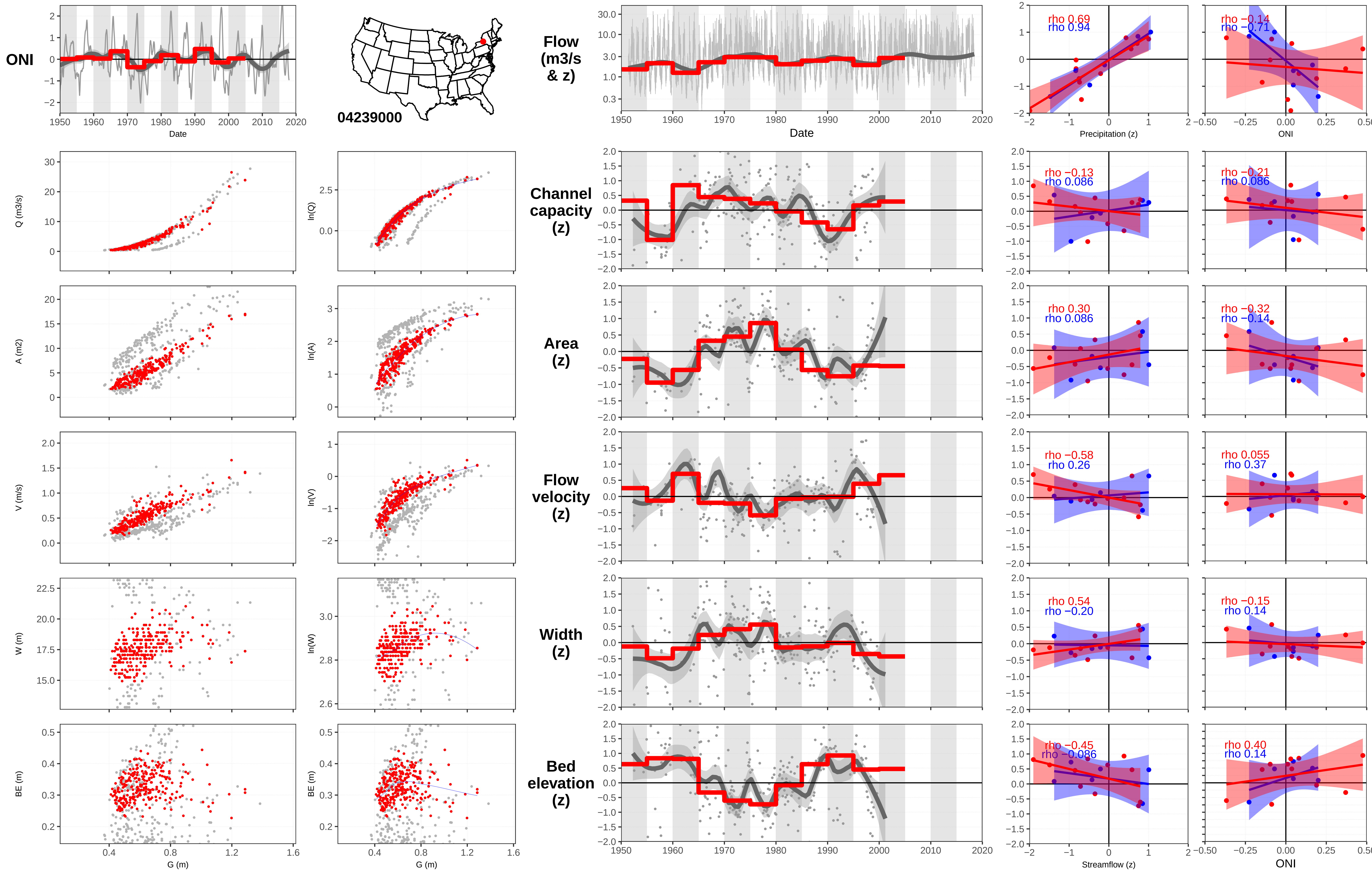
ONI
04239000
Flow (m3/s & z)
Channel capacity (z)
Area (z)
Flow velocity (z)
Width (z)
Bed elevation (z)
Date
Precipitation (z)
Streamflow (z)
ONI
G (m)
Q (m3/s)
ln(Q)
A (m2)
ln(A)
V (m/s)
ln(V)
W (m)
ln(W)
BE (m)
rho 0.69
rho 0.94
rho −0.14
rho −0.71
rho −0.13
rho 0.086
rho −0.21
rho 0.086
rho 0.30
rho 0.086
rho −0.32
rho −0.14
rho −0.58
rho 0.26
rho 0.055
rho 0.37
rho 0.54
rho −0.20
rho −0.15
rho 0.14
rho −0.45
rho −0.086
rho 0.40
rho 0.14

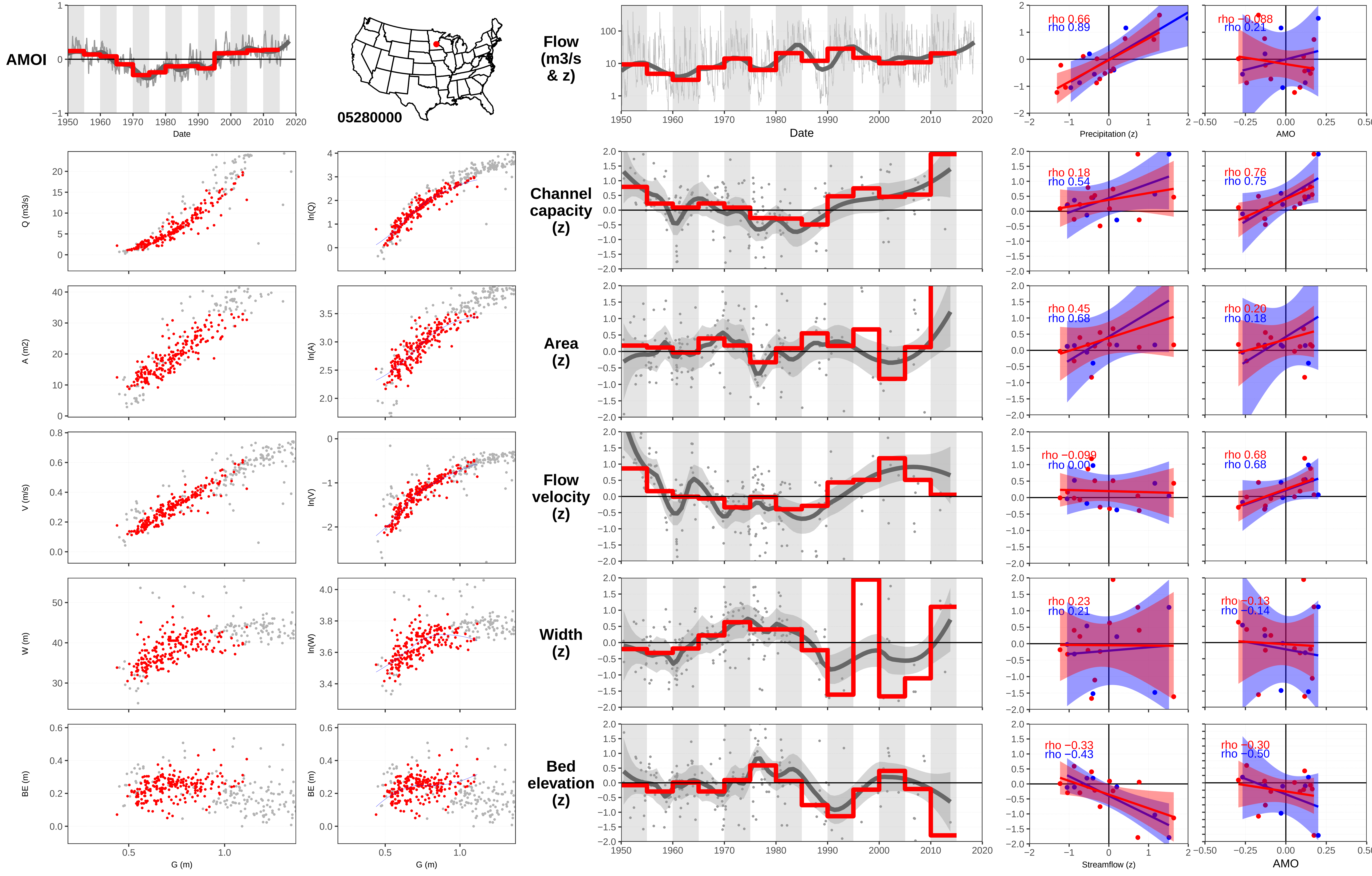
AMOI
05280000
Date
Flow (m3/s & z)
Channel capacity (z)
Area (z)
Flow velocity (z)
Width (z)
Bed elevation (z)
Q (m3/s)
ln(Q)
A (m2)
ln(A)
V (m/s)
ln(V)
W (m)
ln(W)
BE (m)
G (m)
Precipitation (z)
Streamflow (z)
AMO
rho 0.66
rho 0.89
rho −0.088
rho 0.21
rho 0.18
rho 0.54
rho 0.76
rho 0.75
rho 0.45
rho 0.68
rho 0.20
rho 0.18
rho −0.099
rho 0.00
rho 0.68
rho 0.68
rho 0.23
rho 0.21
rho −0.13
rho −0.14
rho −0.33
rho −0.43
rho −0.30
rho −0.50

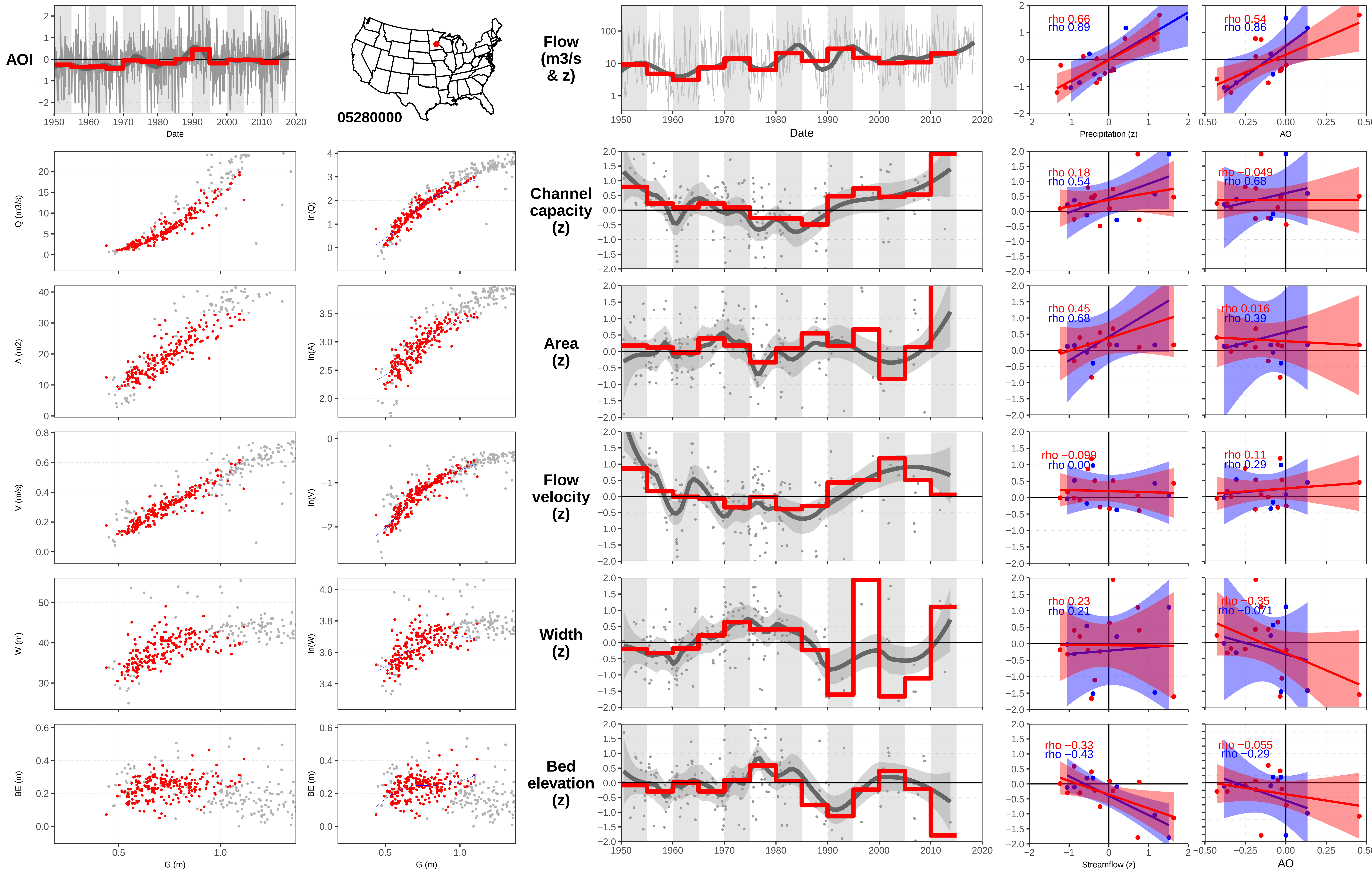

AOI
05280000
Flow (m3/s & z)
Channel capacity (z)
Area (z)
Flow velocity (z)
Width (z)
Bed elevation (z)
Date
Precipitation (z)
AO
Streamflow (z)
G (m)
Q (m3/s)
ln(Q)
A (m2)
ln(A)
V (m/s)
ln(V)
W (m)
ln(W)
BE (m)
rho 0.66
rho 0.89
rho 0.54
rho 0.86
rho 0.18
rho 0.54
rho −0.049
rho 0.68
rho 0.45
rho 0.68
rho 0.016
rho 0.39
rho −0.099
rho 0.00
rho 0.11
rho 0.29
rho 0.23
rho 0.21
rho −0.35
rho −0.071
rho −0.33
rho −0.43
rho −0.055
rho −0.29

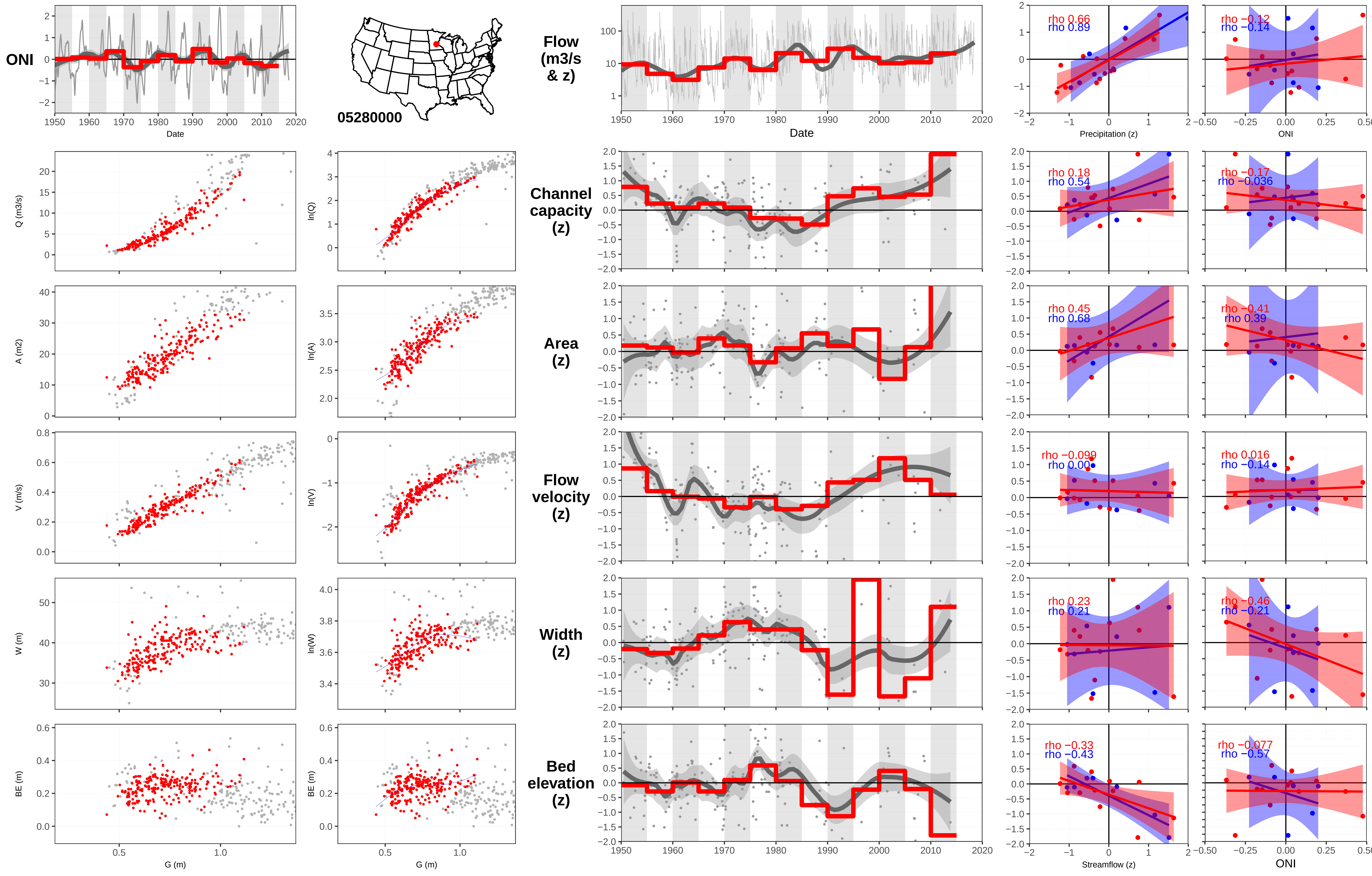

ONI
05280000
Flow (m3/s & z)
Channel capacity (z)
Area (z)
Flow velocity (z)
Width (z)
Bed elevation (z)
Date
Precipitation (z)
Streamflow (z)
ONI
G (m)
Q (m3/s)
ln(Q)
A (m2)
ln(A)
V (m/s)
ln(V)
W (m)
ln(W)
BE (m)
rho 0.66
rho 0.89
rho −0.12
rho −0.14
rho 0.18
rho 0.54
rho −0.17
rho −0.036
rho 0.45
rho 0.68
rho −0.41
rho 0.39
rho −0.099
rho 0.00
rho 0.016
rho −0.14
rho 0.23
rho 0.21
rho −0.46
rho −0.21
rho −0.33
rho −0.43
rho −0.077
rho −0.57

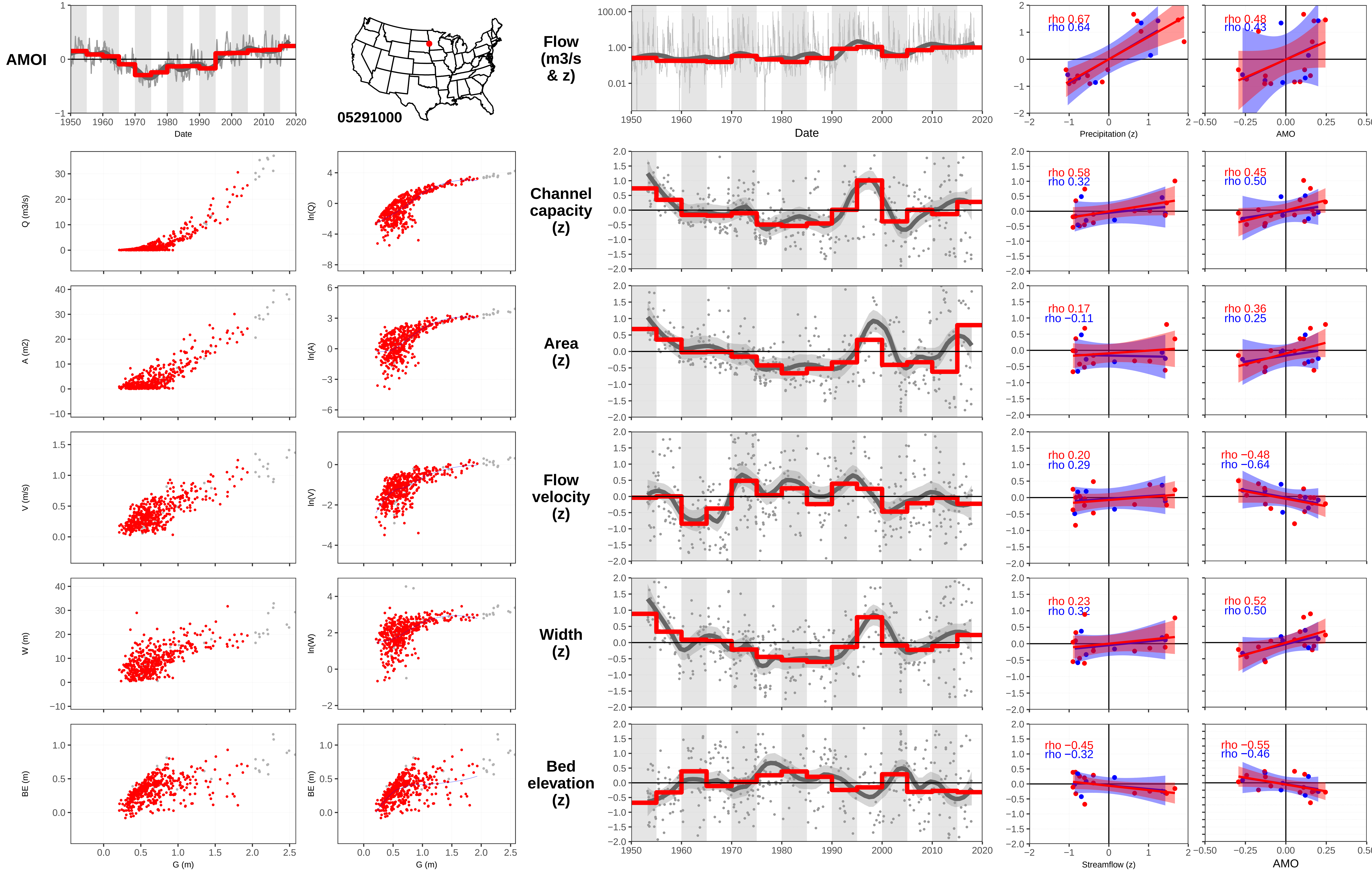

AMOI
05291000
Flow (m3/s & z)
Channel capacity (z)
Area (z)
Flow velocity (z)
Width (z)
Bed elevation (z)
Date
Precipitation (z)
AMO
Streamflow (z)
G (m)
Q (m3/s)
ln(Q)
A (m2)
ln(A)
V (m/s)
ln(V)
W (m)
ln(W)
BE (m)
rho 0.67
rho 0.64
rho 0.48
rho 0.43
rho 0.58
rho 0.32
rho 0.45
rho 0.50
rho 0.17
rho −0.11
rho 0.36
rho 0.25
rho 0.20
rho 0.29
rho −0.48
rho −0.64
rho 0.23
rho 0.32
rho 0.52
rho 0.50
rho −0.45
rho −0.32
rho −0.55
rho −0.46

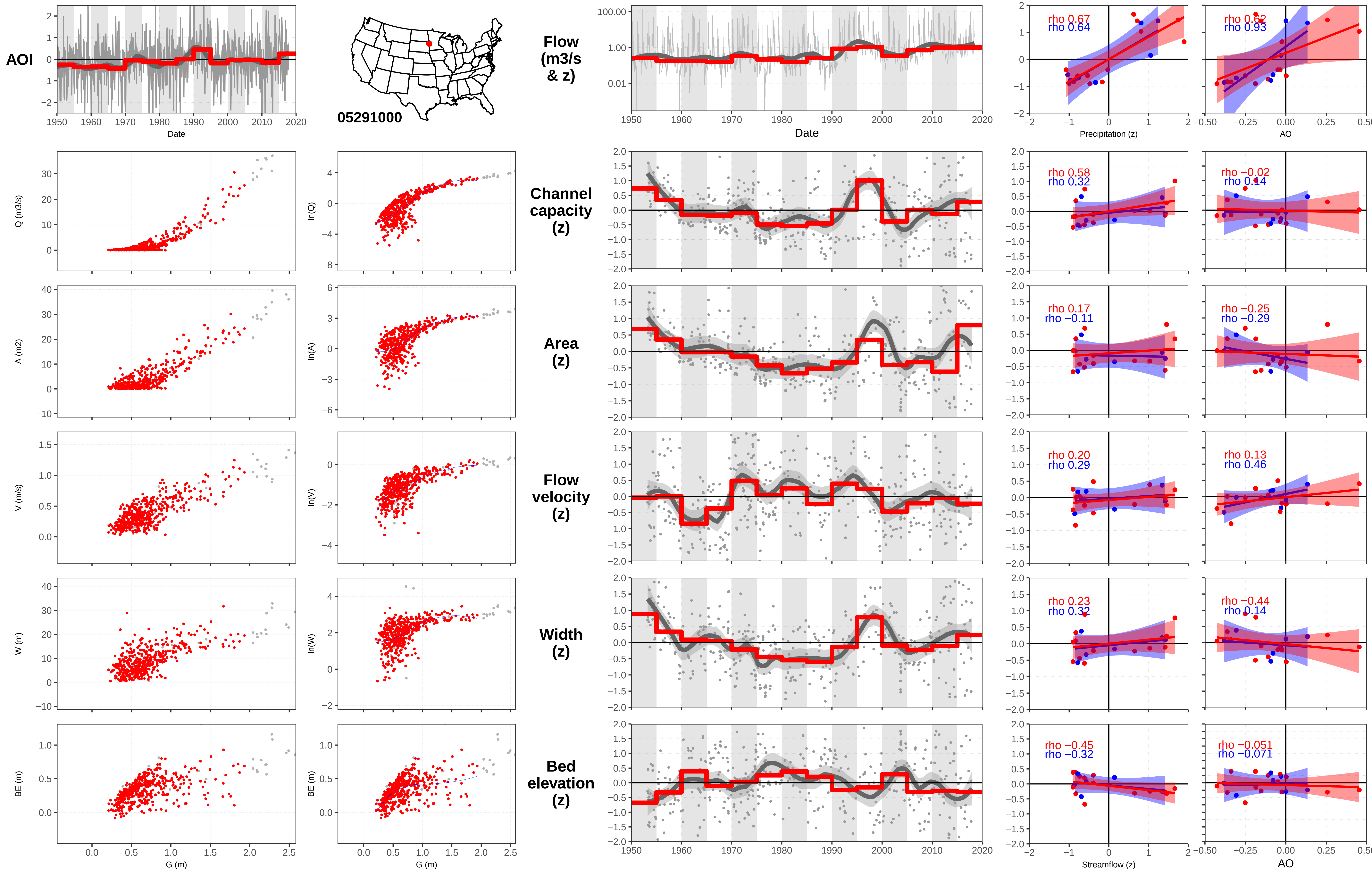

AOI
05291000
Flow (m3/s & z)
Channel capacity (z)
Area (z)
Flow velocity (z)
Width (z)
Bed elevation (z)
Date
Precipitation (z)
AO
Streamflow (z)
G (m)
Q (m3/s)
ln(Q)
A (m2)
ln(A)
V (m/s)
ln(V)
W (m)
ln(W)
BE (m)
rho 0.67
rho 0.64
rho 0.62
rho 0.93
rho 0.58
rho 0.32
rho −0.02
rho 0.14
rho 0.17
rho −0.11
rho −0.25
rho −0.29
rho 0.20
rho 0.29
rho 0.13
rho 0.46
rho 0.23
rho 0.32
rho −0.44
rho 0.14
rho −0.45
rho −0.32
rho −0.051
rho −0.071

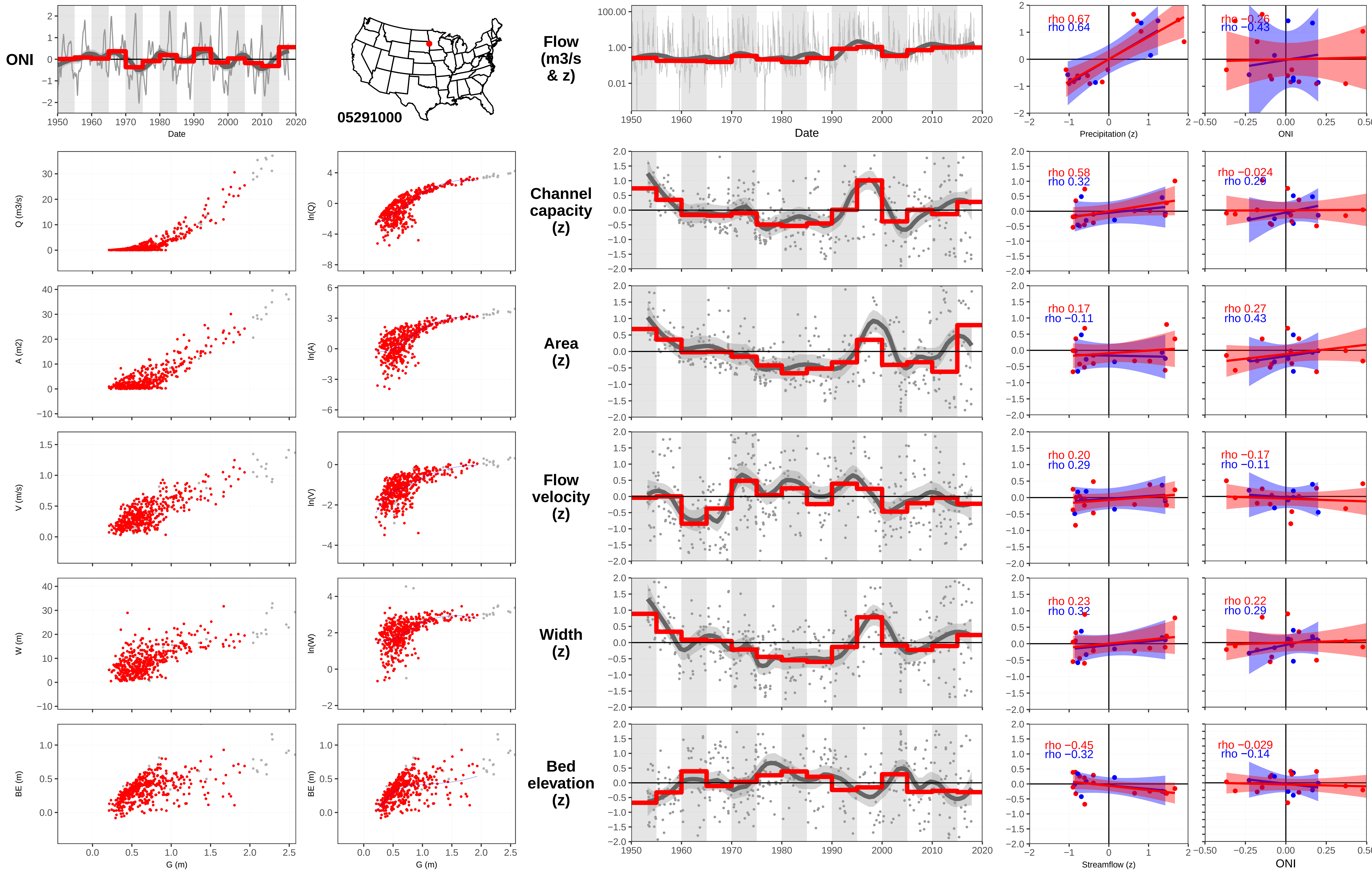

ONI
05291000
Flow (m3/s & z)
Channel capacity (z)
Area (z)
Flow velocity (z)
Width (z)
Bed elevation (z)
Date
Precipitation (z)
Streamflow (z)
ONI
Q (m3/s)
ln(Q)
A (m2)
ln(A)
V (m/s)
ln(V)
W (m)
ln(W)
BE (m)
G (m)
rho 0.67
rho 0.64
rho −0.26
rho −0.43
rho 0.58
rho 0.32
rho −0.024
rho 0.29
rho 0.17
rho −0.11
rho 0.27
rho 0.43
rho 0.20
rho 0.29
rho −0.17
rho −0.11
rho 0.23
rho 0.32
rho 0.22
rho 0.29
rho −0.45
rho −0.32
rho −0.029
rho −0.14

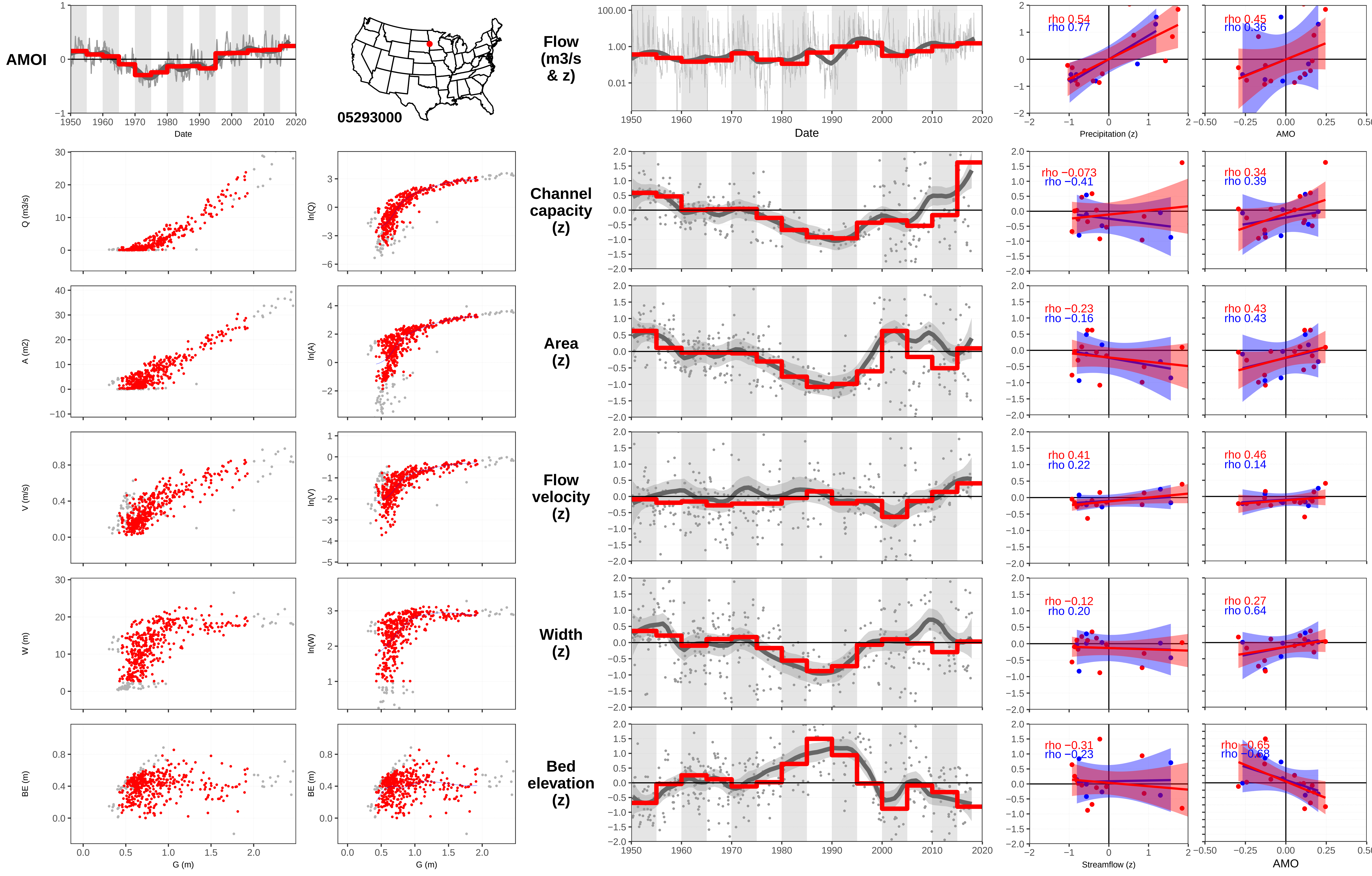

AMOI
05293000
Flow (m3/s & z)
Channel capacity (z)
Area (z)
Flow velocity (z)
Width (z)
Bed elevation (z)
Date
Precipitation (z)
AMO
Streamflow (z)
G (m)
Q (m3/s)
ln(Q)
A (m2)
ln(A)
V (m/s)
ln(V)
W (m)
ln(W)
BE (m)
rho 0.54
rho 0.77
rho 0.45
rho 0.36
rho −0.073
rho −0.41
rho 0.34
rho 0.39
rho −0.23
rho −0.16
rho 0.43
rho 0.43
rho 0.41
rho 0.22
rho 0.46
rho 0.14
rho −0.12
rho 0.20
rho 0.27
rho 0.64
rho −0.31
rho −0.23
rho −0.65
rho −0.68

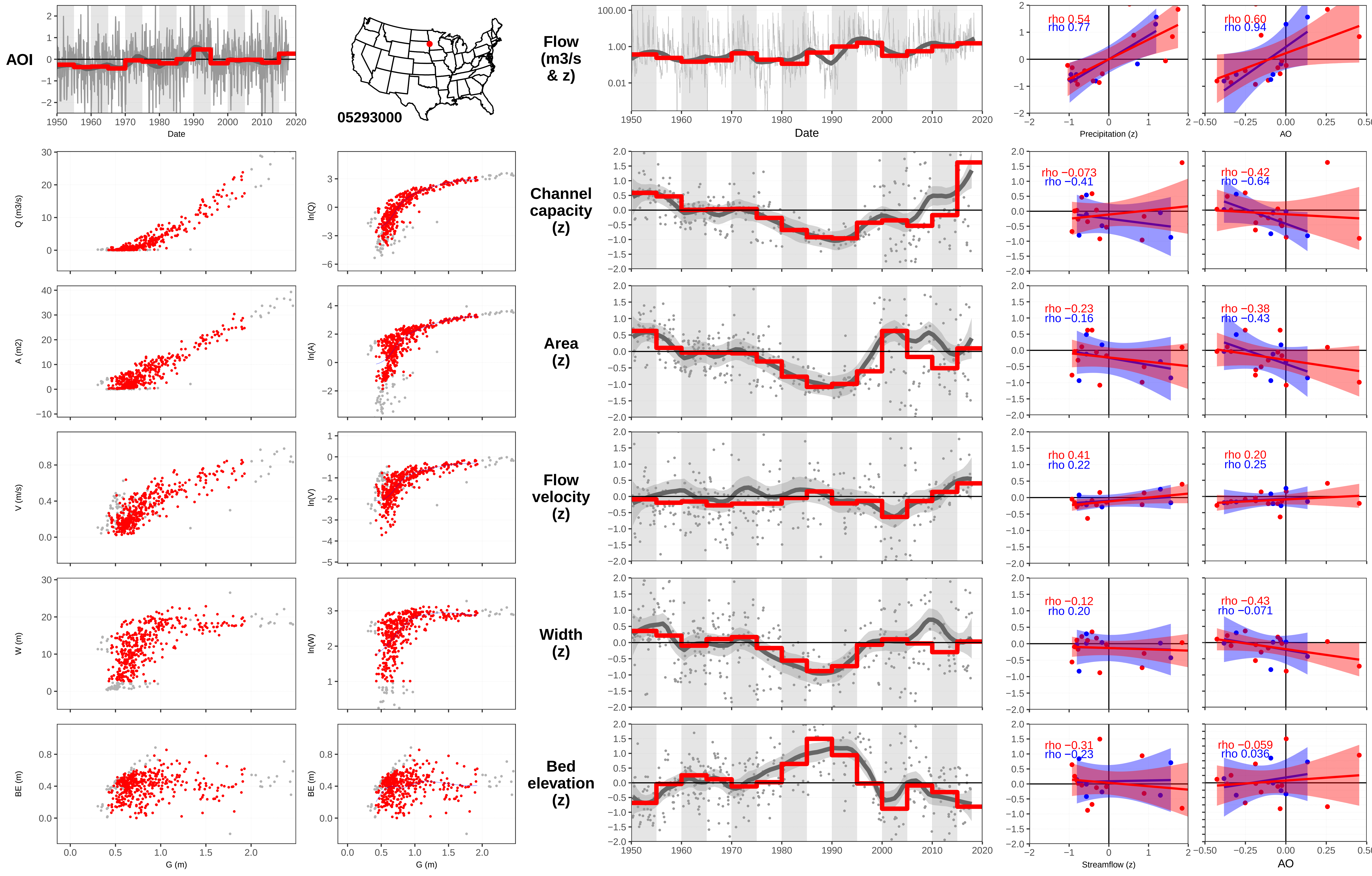

AOI
05293000
Flow (m3/s & z)
Channel capacity (z)
Area (z)
Flow velocity (z)
Width (z)
Bed elevation (z)
Date
Precipitation (z)
AO
Streamflow (z)
G (m)
Q (m3/s)
ln(Q)
A (m2)
ln(A)
V (m/s)
ln(V)
W (m)
ln(W)
BE (m)
rho 0.54
rho 0.77
rho 0.60
rho 0.94
rho −0.073
rho −0.41
rho −0.42
rho −0.64
rho −0.23
rho −0.16
rho −0.38
rho −0.43
rho 0.41
rho 0.22
rho 0.20
rho 0.25
rho −0.12
rho 0.20
rho −0.43
rho −0.071
rho −0.31
rho −0.23
rho −0.059
rho 0.036

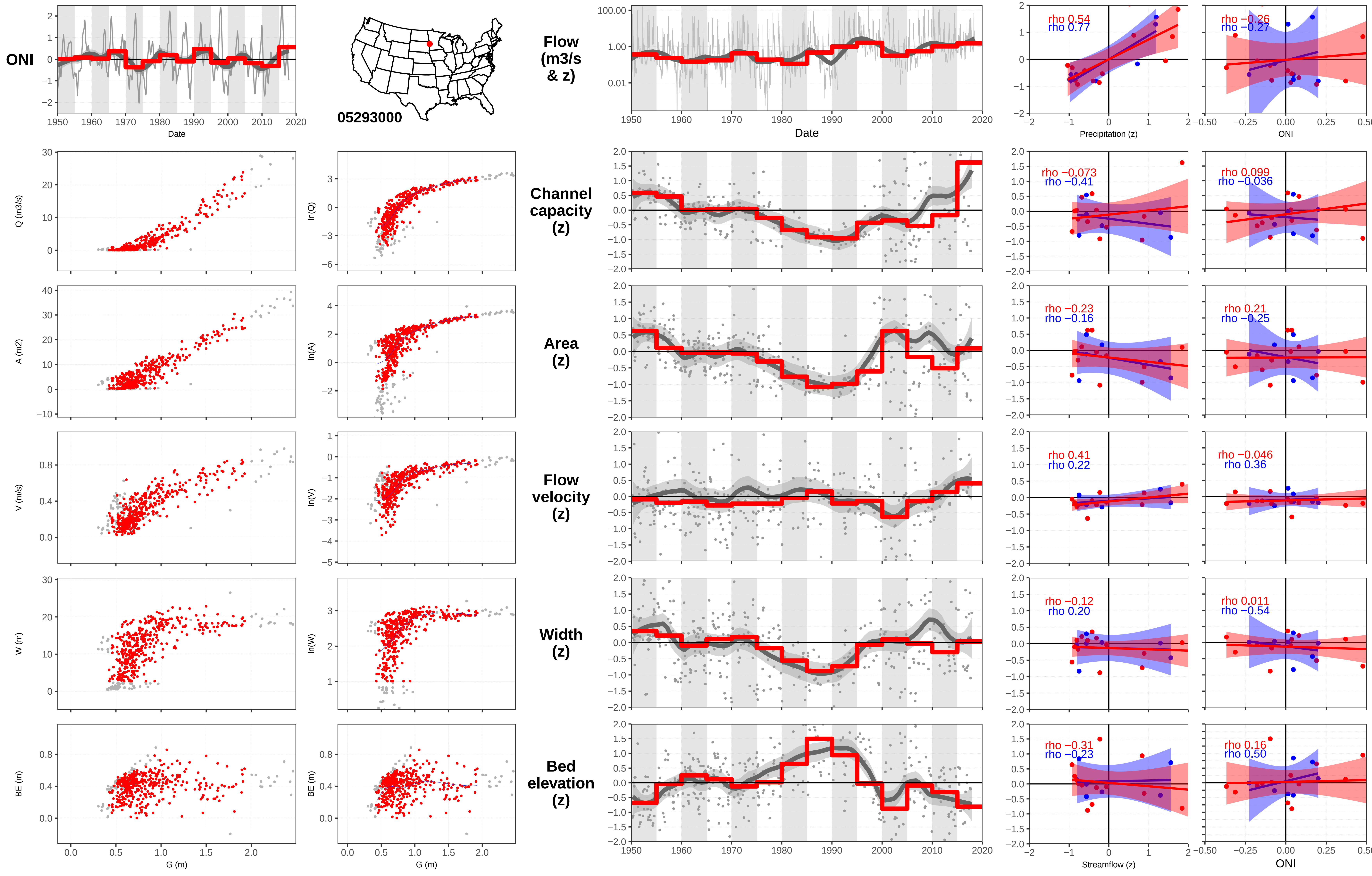

ONI
05293000
Flow (m3/s & z)
Channel capacity (z)
Area (z)
Flow velocity (z)
Width (z)
Bed elevation (z)
Date
Precipitation (z)
Streamflow (z)
G (m)
Q (m3/s)
ln(Q)
A (m2)
ln(A)
V (m/s)
ln(V)
W (m)
ln(W)
BE (m)
rho 0.54
rho 0.77
rho −0.26
rho −0.27
rho −0.073
rho −0.41
rho 0.099
rho −0.036
rho −0.23
rho −0.16
rho 0.21
rho −0.25
rho 0.41
rho 0.22
rho −0.046
rho 0.36
rho −0.12
rho 0.20
rho 0.011
rho −0.54
rho −0.31
rho −0.23
rho 0.16
rho 0.50

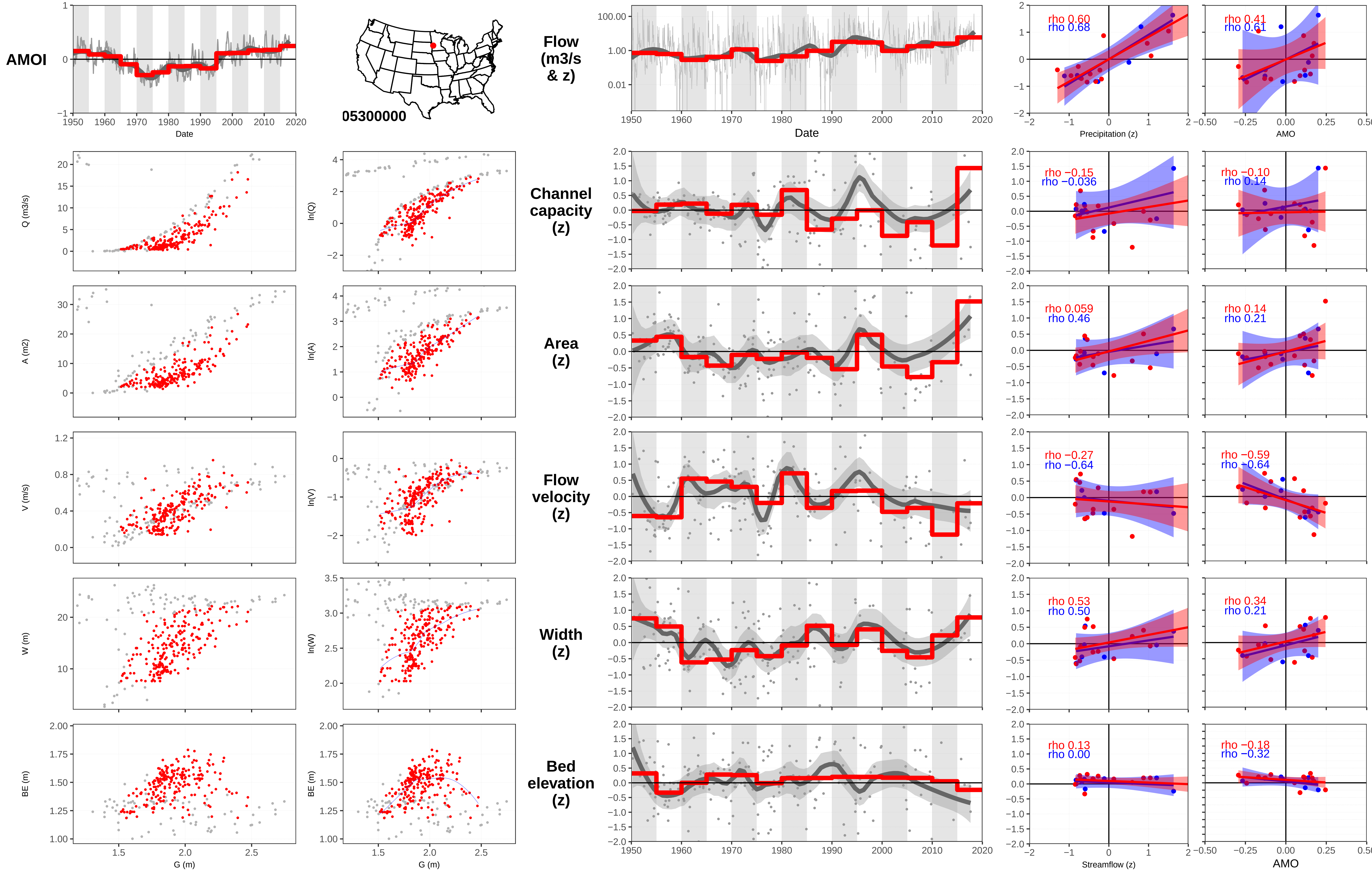
AMOI
05300000
Flow (m3/s & z)
Channel capacity (z)
Area (z)
Flow velocity (z)
Width (z)
Bed elevation (z)
Date
Precipitation (z)
AMO
Streamflow (z)
G (m)
Q (m3/s)
ln(Q)
A (m2)
ln(A)
V (m/s)
ln(V)
W (m)
ln(W)
BE (m)
rho 0.60
rho 0.68
rho 0.41
rho 0.61
rho −0.15
rho −0.036
rho −0.10
rho 0.14
rho 0.059
rho 0.46
rho 0.14
rho 0.21
rho −0.27
rho −0.64
rho −0.59
rho −0.64
rho 0.53
rho 0.50
rho 0.34
rho 0.21
rho 0.13
rho 0.00
rho −0.18
rho −0.32

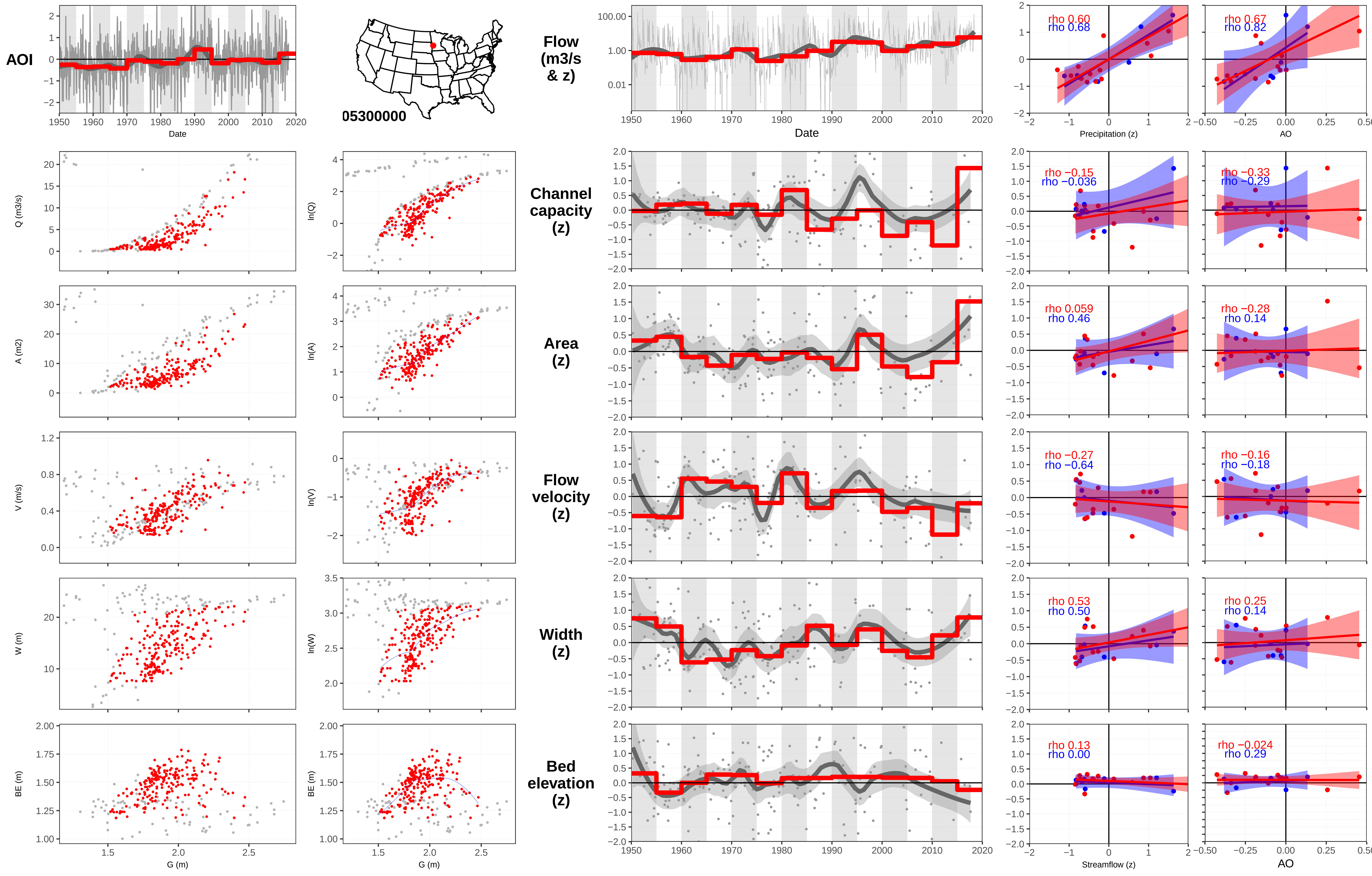
AOI
05300000
Flow (m3/s & z)
Channel capacity (z)
Area (z)
Flow velocity (z)
Width (z)
Bed elevation (z)
Date
Precipitation (z)
AO
Streamflow (z)
Q (m3/s)
ln(Q)
A (m2)
ln(A)
V (m/s)
ln(V)
W (m)
ln(W)
BE (m)
G (m)
rho 0.60
rho 0.68
rho 0.67
rho 0.82
rho −0.15
rho −0.036
rho −0.33
rho −0.29
rho 0.059
rho 0.46
rho −0.28
rho 0.14
rho −0.27
rho −0.64
rho −0.16
rho −0.18
rho 0.53
rho 0.50
rho 0.25
rho 0.14
rho 0.13
rho 0.00
rho −0.024
rho 0.29

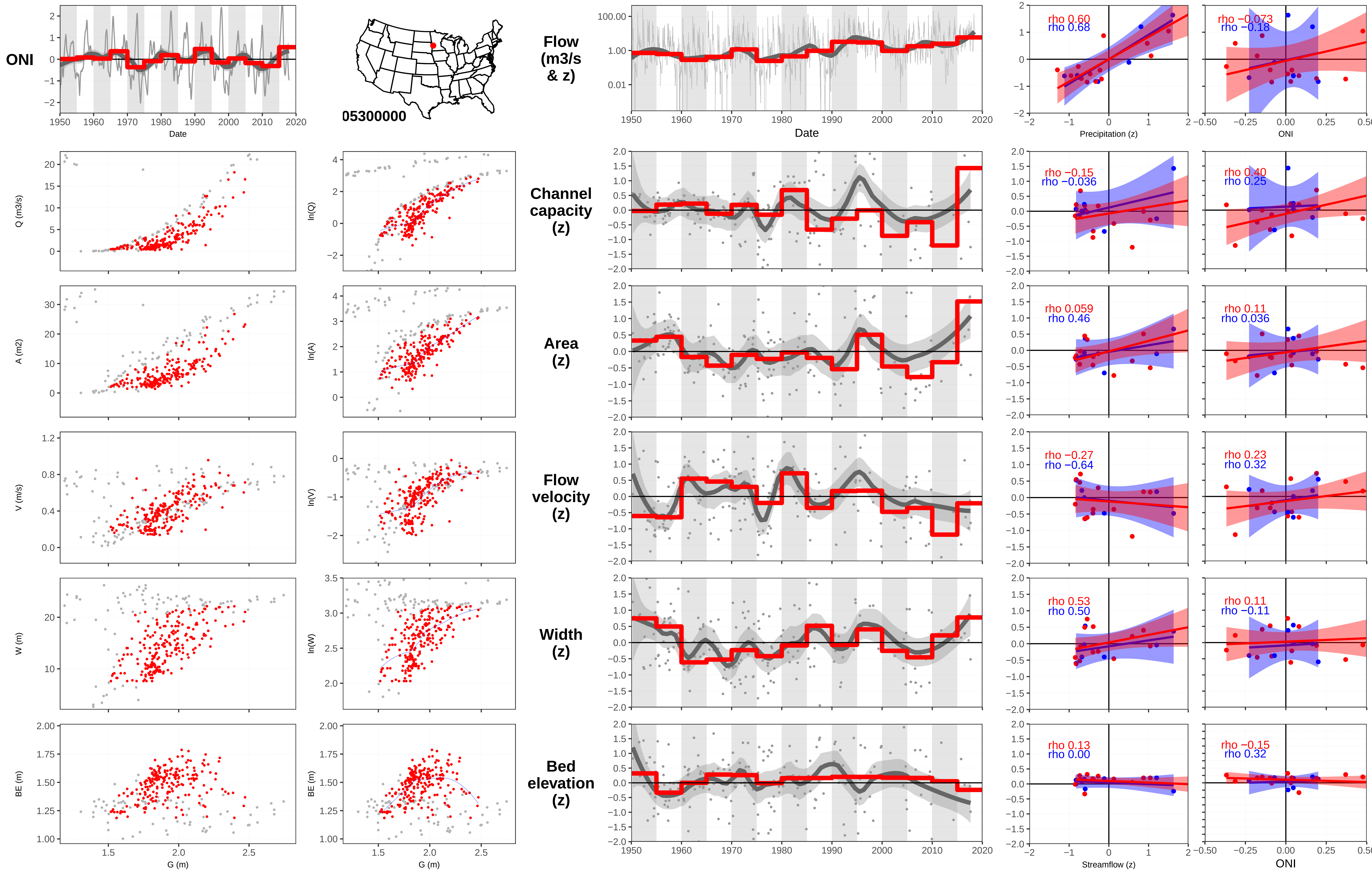

ONI
05300000
Flow (m3/s & z)
Channel capacity (z)
Area (z)
Flow velocity (z)
Width (z)
Bed elevation (z)
Date
Precipitation (z)
Streamflow (z)
ONI
Q (m3/s)
ln(Q)
A (m2)
ln(A)
V (m/s)
ln(V)
W (m)
ln(W)
BE (m)
G (m)
rho 0.60
rho 0.68
rho −0.073
rho −0.18
rho −0.15
rho −0.036
rho 0.40
rho 0.25
rho 0.059
rho 0.46
rho 0.11
rho 0.036
rho −0.27
rho −0.64
rho 0.23
rho 0.32
rho 0.53
rho 0.50
rho 0.11
rho −0.11
rho 0.13
rho 0.00
rho −0.15
rho 0.32

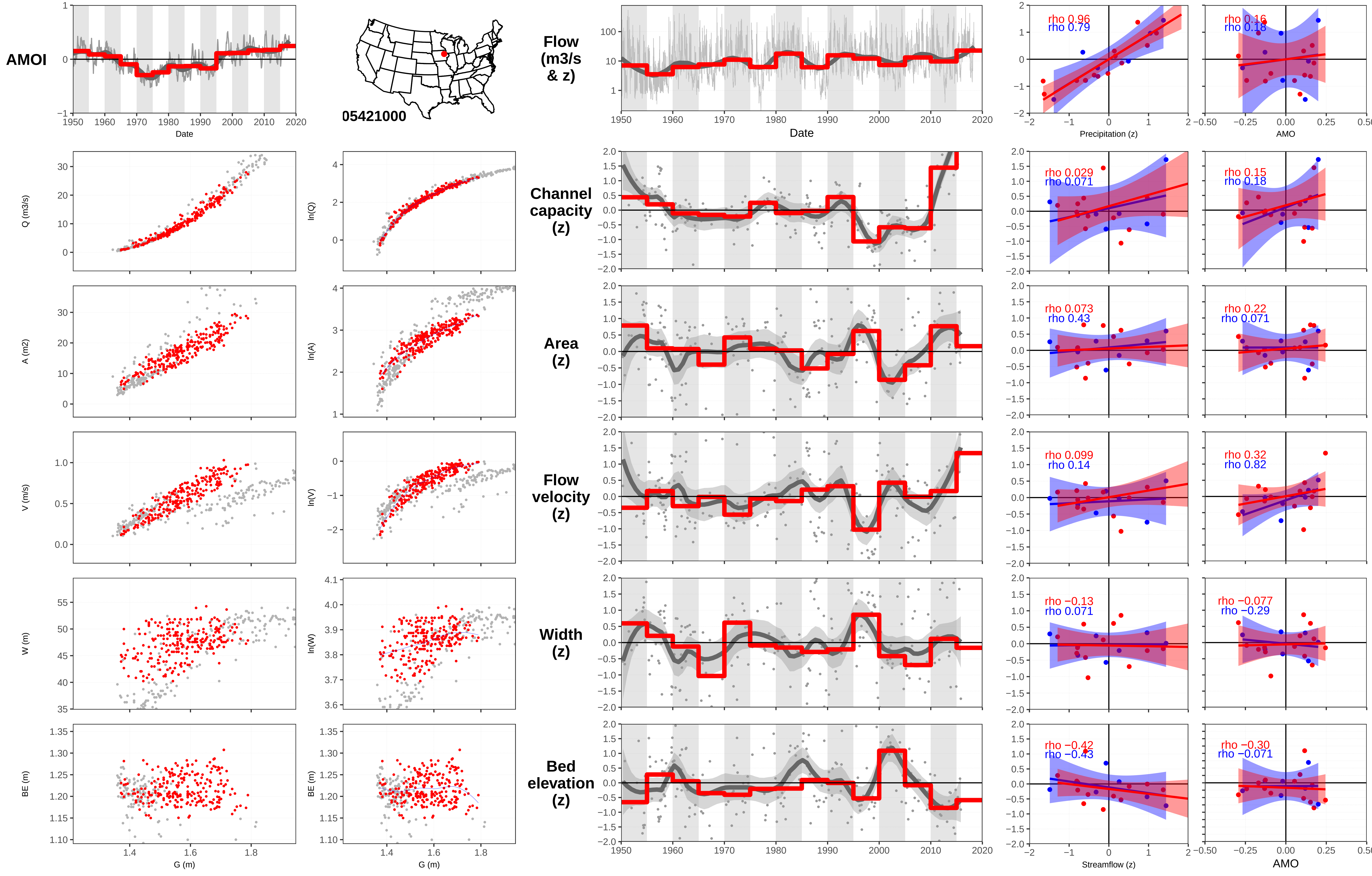

AMOI
Date
05421000
Flow (m3/s & z)
Date
rho 0.96
rho 0.79
Precipitation (z)
rho 0.16
rho 0.18
AMO
Q (m3/s)
ln(Q)
Channel capacity (z)
rho 0.029
rho 0.071
rho 0.15
rho 0.18
A (m2)
ln(A)
Area (z)
rho 0.073
rho 0.43
rho 0.22
rho 0.071
V (m/s)
ln(V)
Flow velocity (z)
rho 0.099
rho 0.14
rho 0.32
rho 0.82
W (m)
ln(W)
Width (z)
rho −0.13
rho 0.071
rho −0.077
rho −0.29
BE (m)
BE (m)
Bed elevation (z)
rho −0.42
rho −0.43
rho −0.30
rho −0.071
G (m)
G (m)
Streamflow (z)
AMO

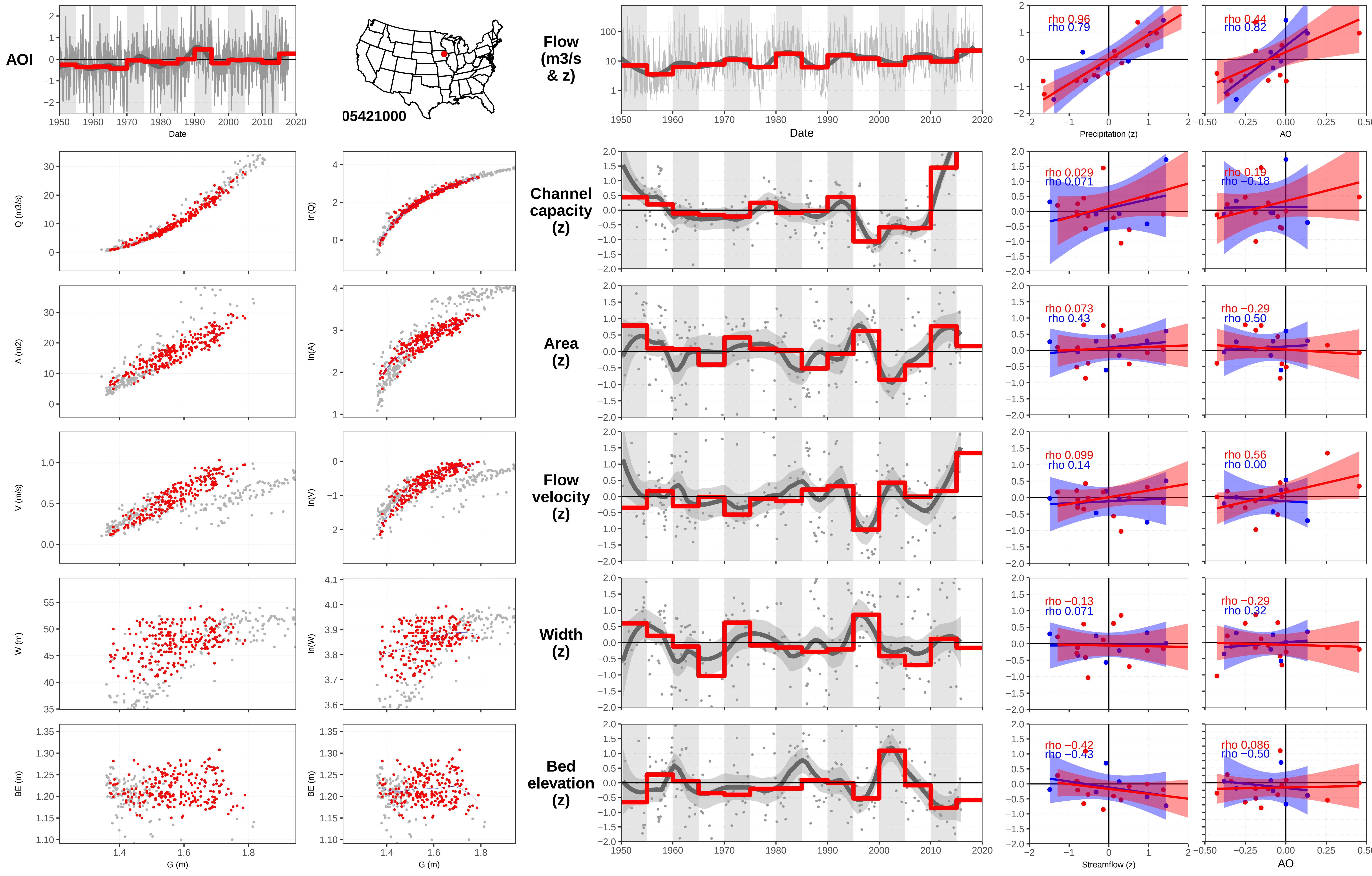

AOI
05421000
Flow (m3/s & z)
Channel capacity (z)
Area (z)
Flow velocity (z)
Width (z)
Bed elevation (z)
Date
Precipitation (z)
AO
Streamflow (z)
G (m)
Q (m3/s)
ln(Q)
A (m2)
ln(A)
V (m/s)
ln(V)
W (m)
ln(W)
BE (m)
rho 0.96
rho 0.79
rho 0.44
rho 0.82
rho 0.029
rho 0.071
rho 0.19
rho −0.18
rho 0.073
rho 0.43
rho −0.29
rho 0.50
rho 0.099
rho 0.14
rho 0.56
rho 0.00
rho −0.13
rho 0.071
rho −0.29
rho 0.32
rho −0.42
rho −0.43
rho 0.086
rho −0.50

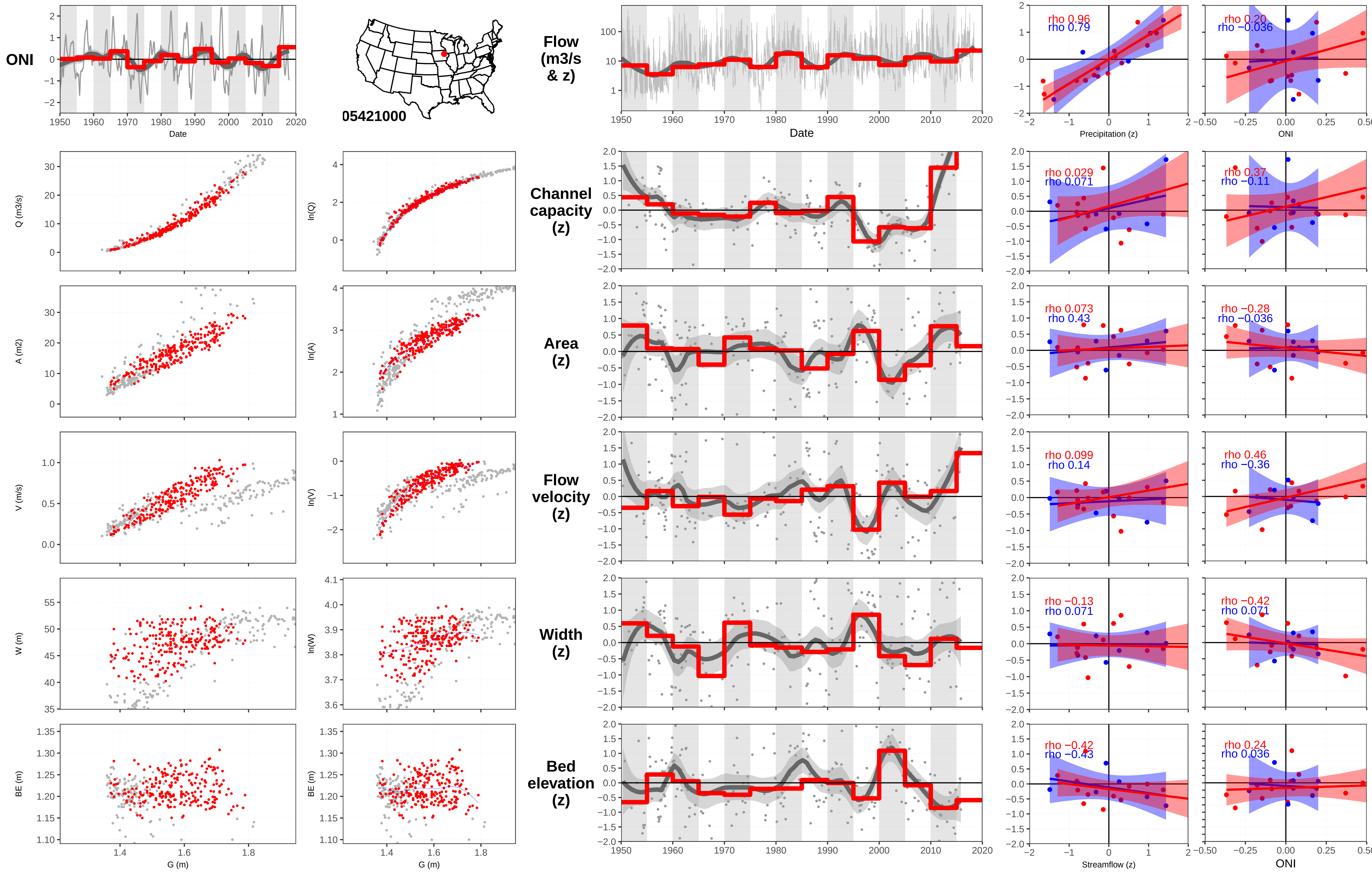

ONI
Date
05421000
Flow (m3/s & z)
Date
rho 0.96
rho 0.79
Precipitation (z)
rho 0.20
rho −0.036
ONI
Q (m3/s)
ln(Q)
Channel capacity (z)
rho 0.029
rho 0.071
rho 0.37
rho −0.11
A (m2)
ln(A)
Area (z)
rho 0.073
rho 0.43
rho −0.28
rho −0.036
V (m/s)
ln(V)
Flow velocity (z)
rho 0.099
rho 0.14
rho 0.46
rho −0.36
W (m)
ln(W)
Width (z)
rho −0.13
rho 0.071
rho −0.42
rho 0.071
BE (m)
BE (m)
Bed elevation (z)
rho −0.42
rho −0.43
rho 0.24
rho 0.036
G (m)
G (m)
Streamflow (z)
ONI

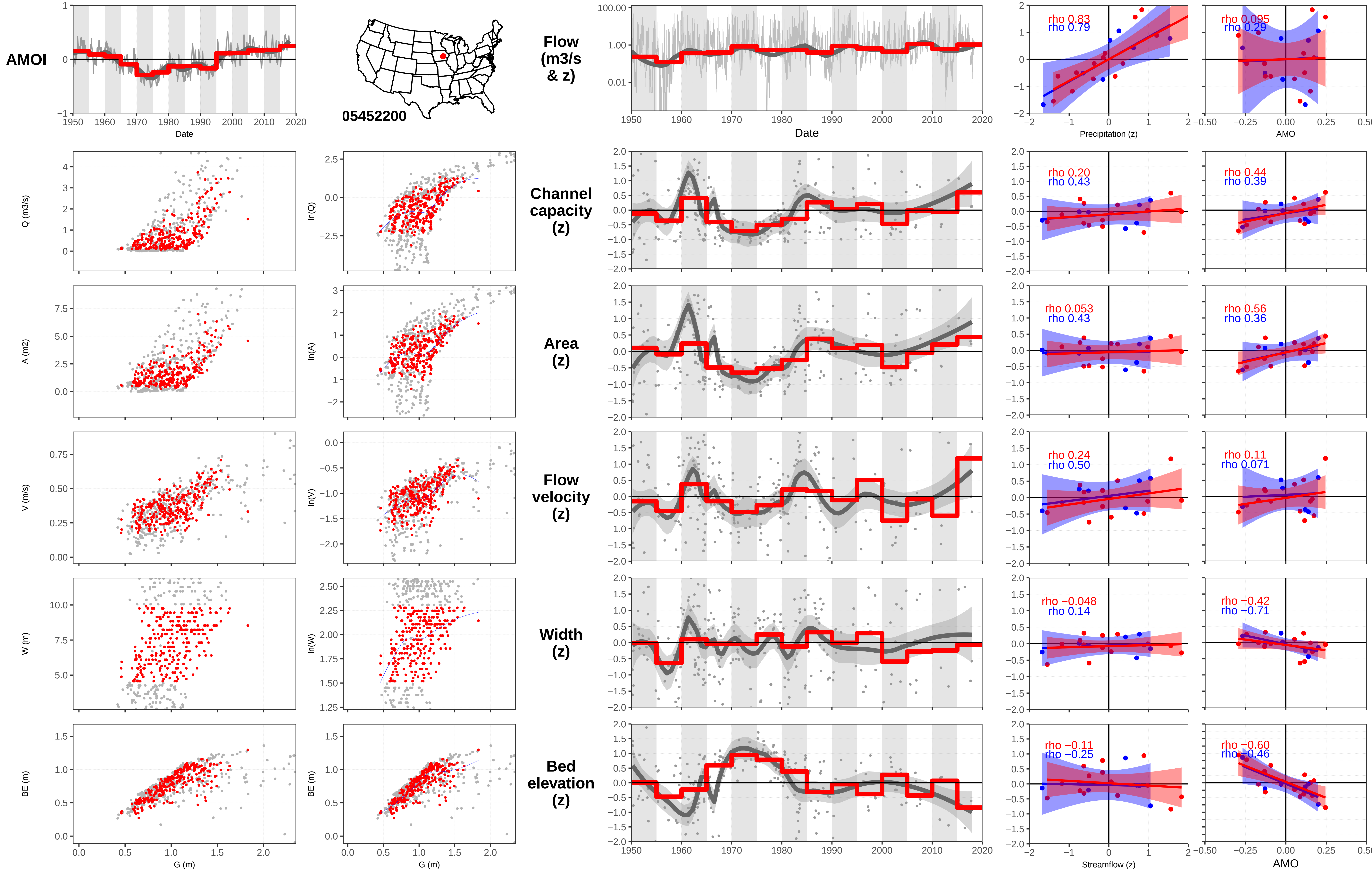

AMOI
05452200
Flow (m3/s & z)
Channel capacity (z)
Area (z)
Flow velocity (z)
Width (z)
Bed elevation (z)
Date
Precipitation (z)
AMO
Streamflow (z)
G (m)
Q (m3/s)
ln(Q)
A (m2)
ln(A)
V (m/s)
ln(V)
W (m)
ln(W)
BE (m)
rho 0.83
rho 0.79
rho 0.095
rho 0.29
rho 0.20
rho 0.43
rho 0.44
rho 0.39
rho 0.053
rho 0.43
rho 0.56
rho 0.36
rho 0.24
rho 0.50
rho 0.11
rho 0.071
rho −0.048
rho 0.14
rho −0.42
rho −0.71
rho −0.11
rho −0.25
rho −0.60
rho −0.46

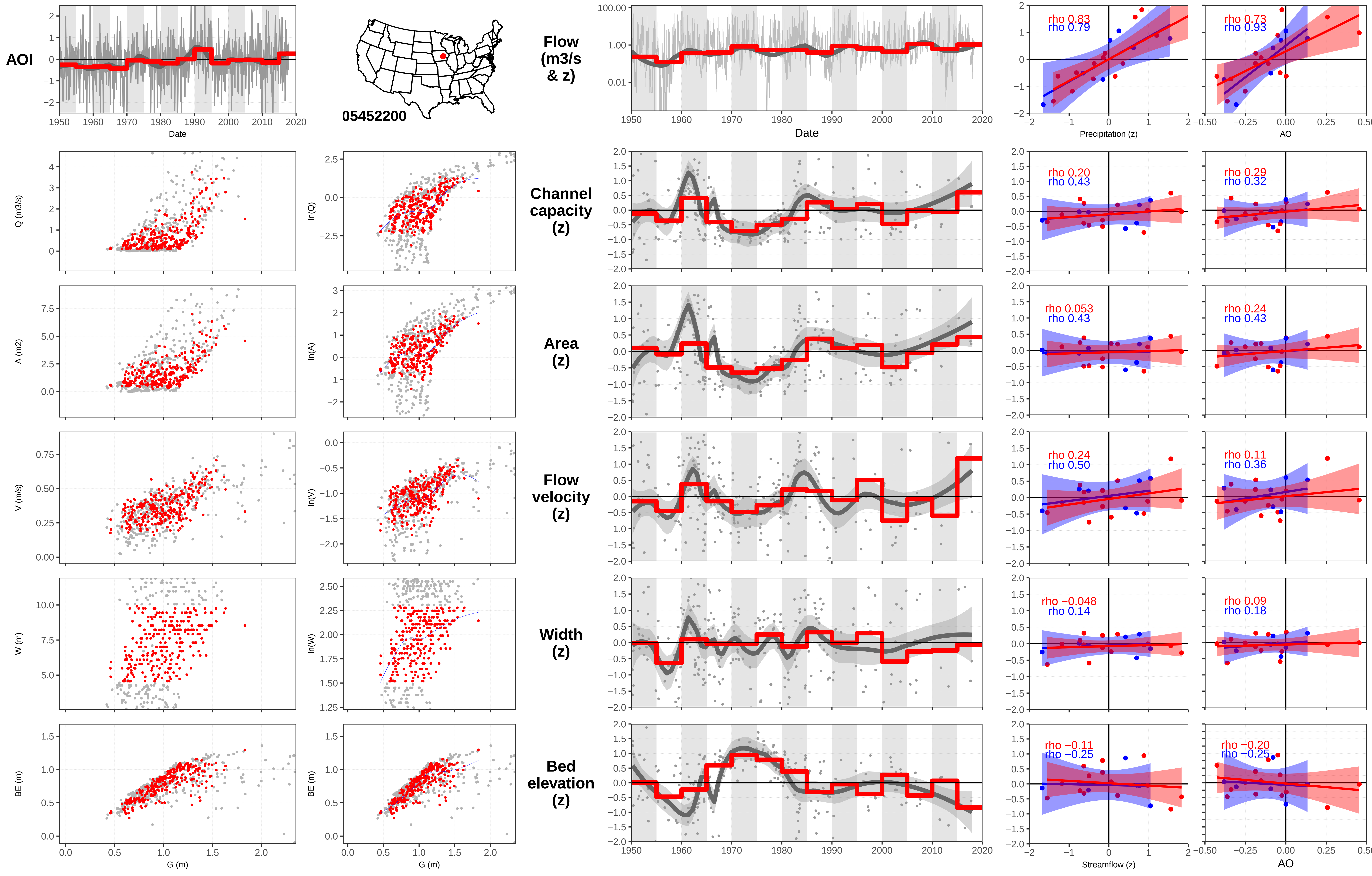

AOI
05452200
Flow (m3/s & z)
Channel capacity (z)
Area (z)
Flow velocity (z)
Width (z)
Bed elevation (z)
Date
Precipitation (z)
AO
Streamflow (z)
G (m)
Q (m3/s)
ln(Q)
A (m2)
ln(A)
V (m/s)
ln(V)
W (m)
ln(W)
BE (m)
rho 0.83
rho 0.79
rho 0.73
rho 0.93
rho 0.20
rho 0.43
rho 0.29
rho 0.32
rho 0.053
rho 0.43
rho 0.24
rho 0.43
rho 0.24
rho 0.50
rho 0.11
rho 0.36
rho −0.048
rho 0.14
rho 0.09
rho 0.18
rho −0.11
rho −0.25
rho −0.20
rho −0.25

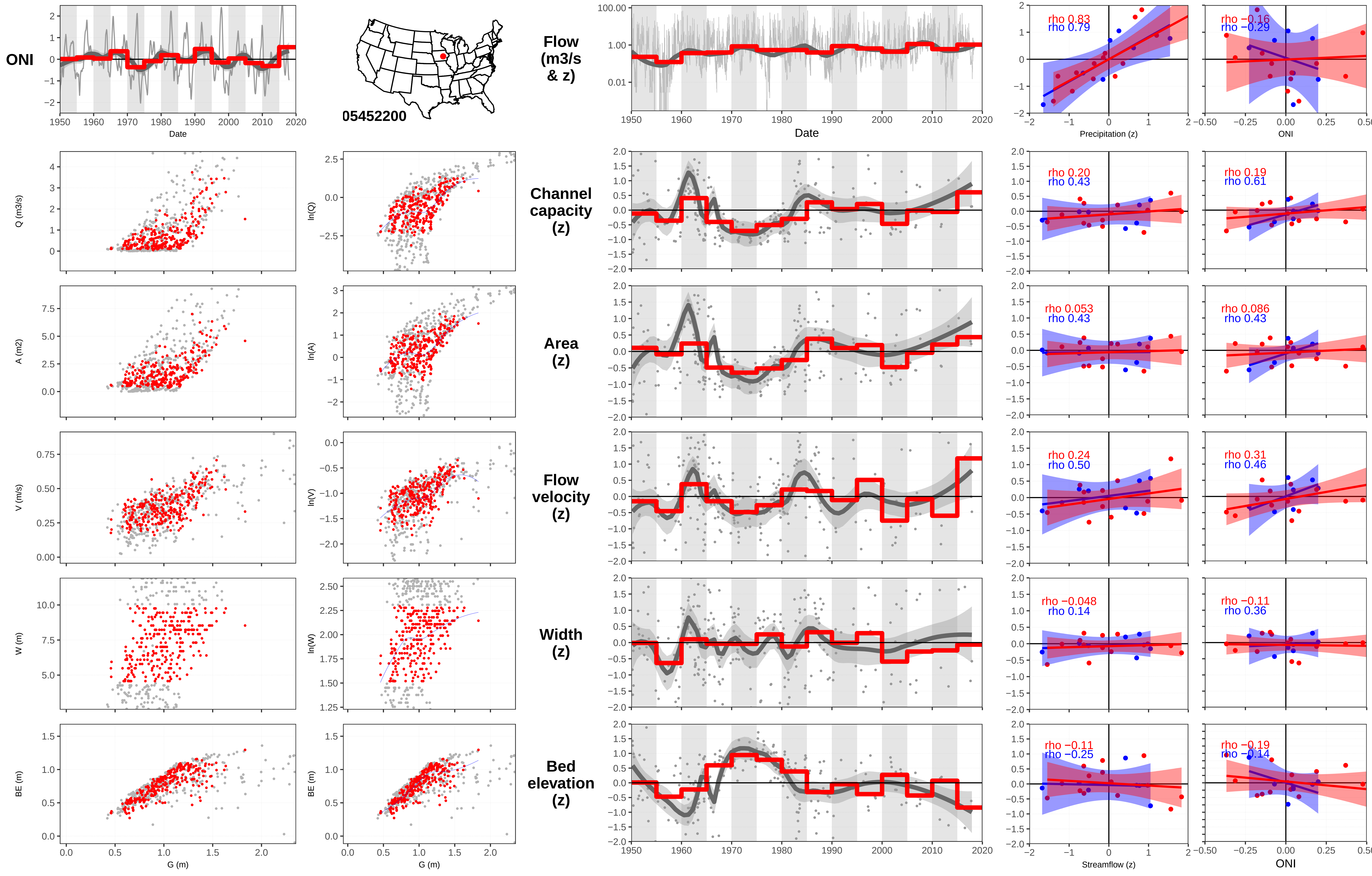

ONI
05452200
Flow (m3/s & z)
Channel capacity (z)
Area (z)
Flow velocity (z)
Width (z)
Bed elevation (z)
Date
Precipitation (z)
Streamflow (z)
G (m)
Q (m3/s)
ln(Q)
A (m2)
ln(A)
V (m/s)
ln(V)
W (m)
ln(W)
BE (m)
rho 0.83
rho 0.79
rho −0.16
rho −0.29
rho 0.20
rho 0.43
rho 0.19
rho 0.61
rho 0.053
rho 0.43
rho 0.086
rho 0.43
rho 0.24
rho 0.50
rho 0.31
rho 0.46
rho −0.048
rho 0.14
rho −0.11
rho 0.36
rho −0.11
rho −0.25
rho −0.19
rho −0.14

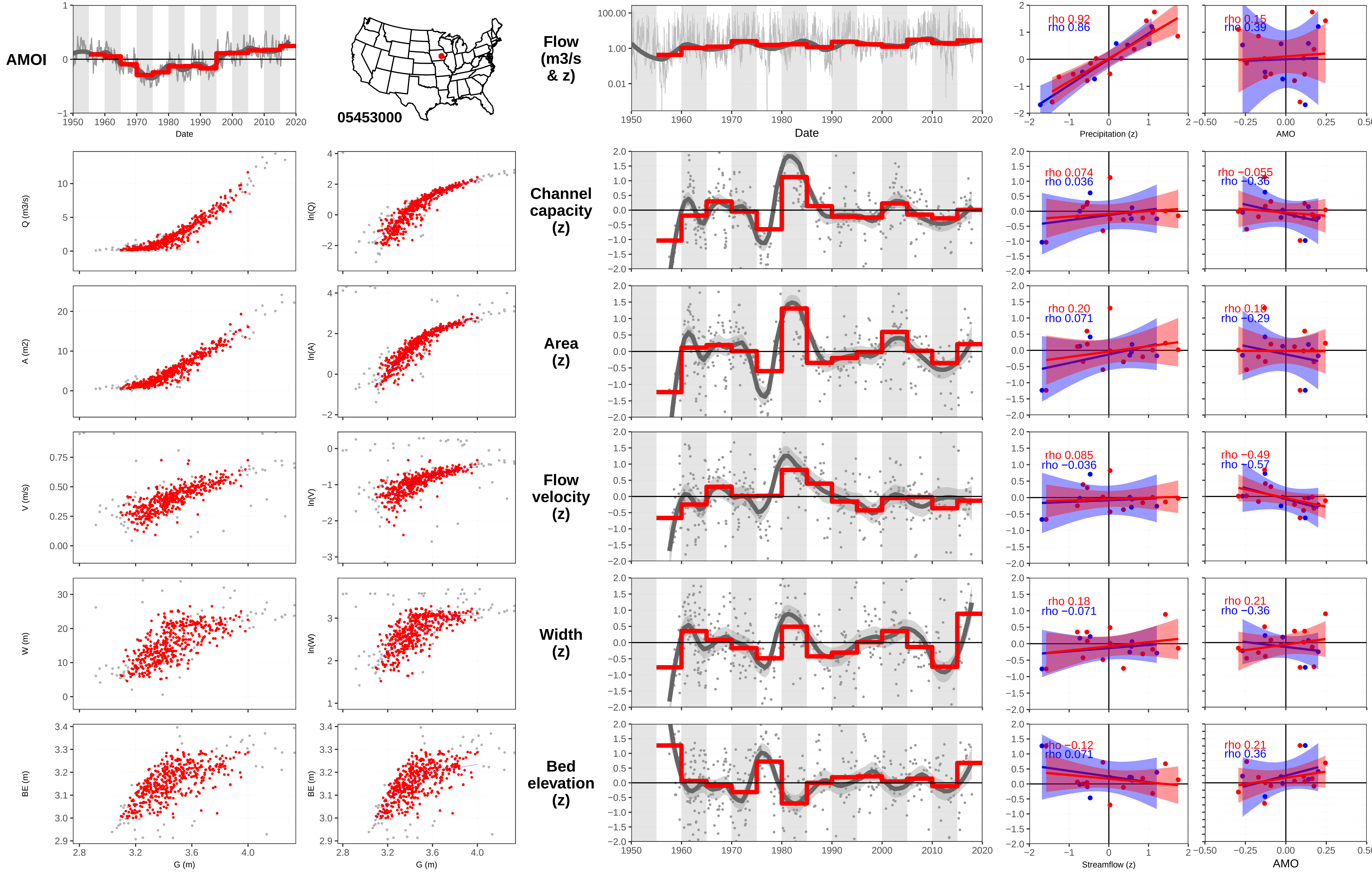

AMOI
05453000
Flow (m3/s & z)
Channel capacity (z)
Area (z)
Flow velocity (z)
Width (z)
Bed elevation (z)
Date
Precipitation (z)
Streamflow (z)
AMO
Q (m3/s)
ln(Q)
A (m2)
ln(A)
V (m/s)
ln(V)
W (m)
ln(W)
BE (m)
G (m)
rho 0.92
rho 0.86
rho 0.15
rho 0.39
rho 0.074
rho 0.036
rho −0.055
rho −0.36
rho 0.20
rho 0.071
rho 0.18
rho −0.29
rho 0.085
rho −0.036
rho −0.49
rho −0.57
rho 0.18
rho −0.071
rho 0.21
rho −0.36
rho −0.12
rho 0.071
rho 0.21
rho 0.36

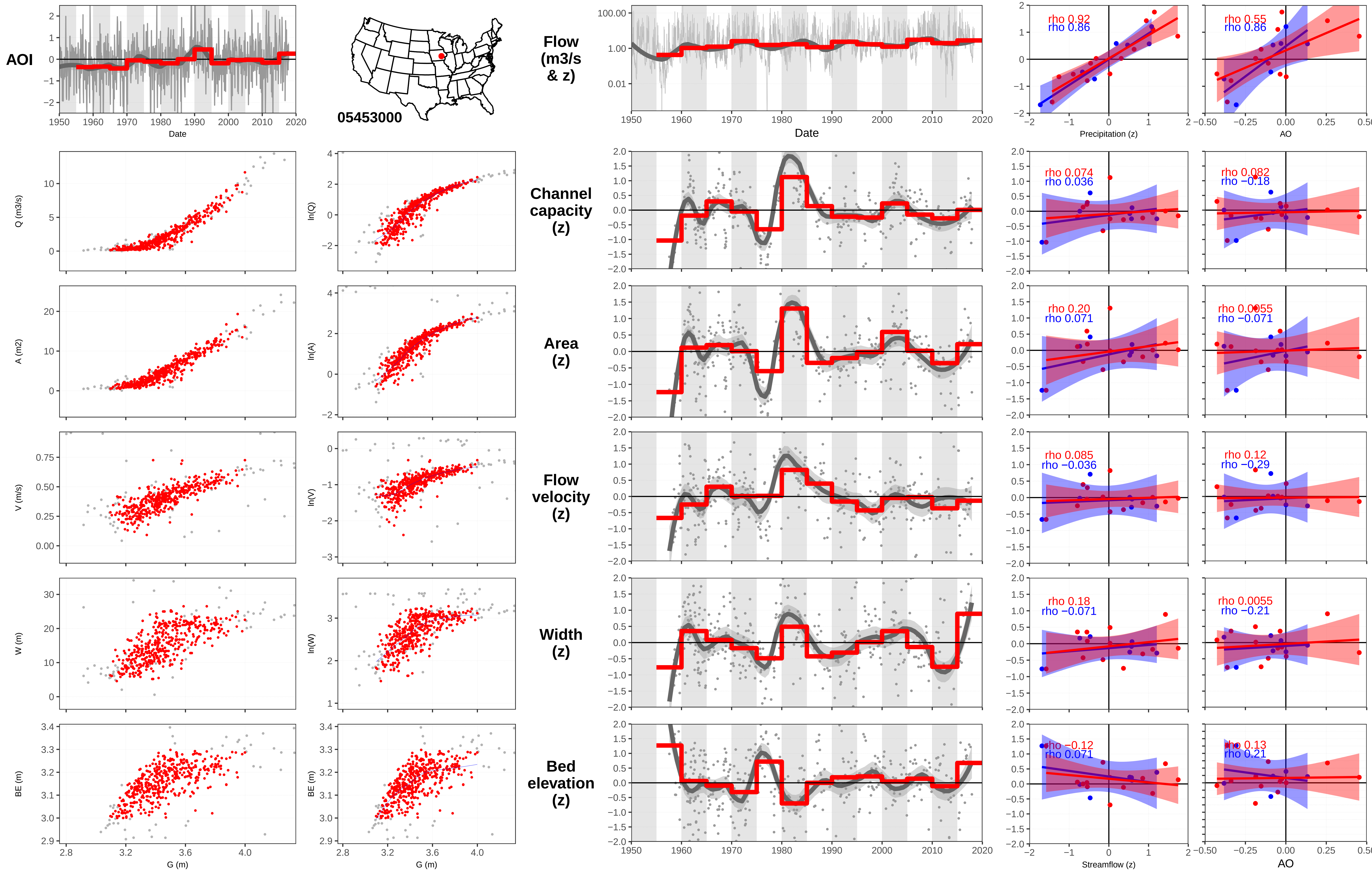

AOI
05453000
Flow (m3/s & z)
Channel capacity (z)
Area (z)
Flow velocity (z)
Width (z)
Bed elevation (z)
Date
Precipitation (z)
AO
Streamflow (z)
G (m)
Q (m3/s)
ln(Q)
A (m2)
ln(A)
V (m/s)
ln(V)
W (m)
ln(W)
BE (m)
rho 0.92
rho 0.86
rho 0.55
rho 0.86
rho 0.074
rho 0.036
rho 0.082
rho −0.18
rho 0.20
rho 0.071
rho 0.0055
rho −0.071
rho 0.085
rho −0.036
rho 0.12
rho −0.29
rho 0.18
rho −0.071
rho 0.0055
rho −0.21
rho −0.12
rho 0.071
rho 0.13
rho 0.21

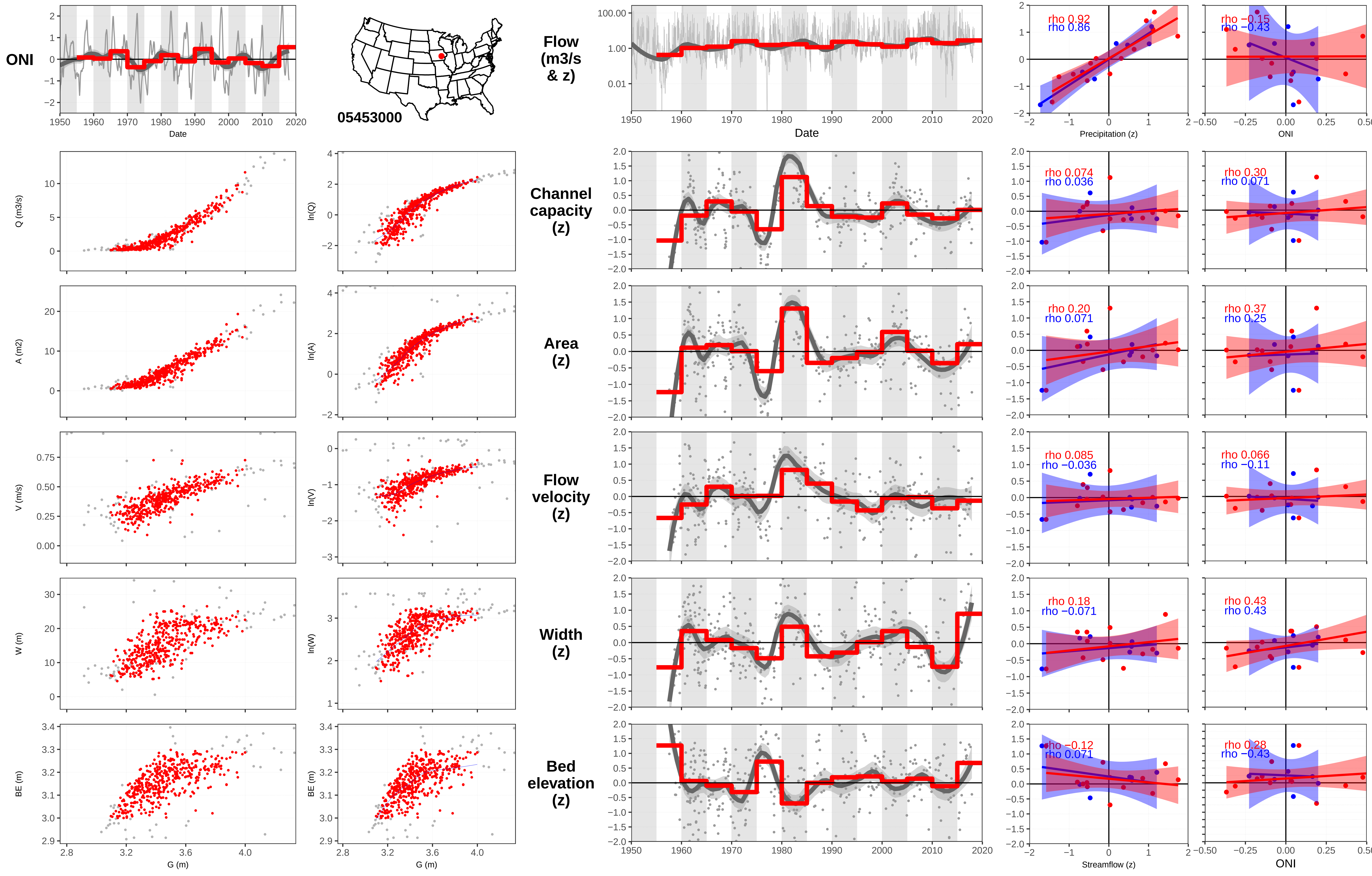

ONI
05453000
Flow (m3/s & z)
Channel capacity (z)
Area (z)
Flow velocity (z)
Width (z)
Bed elevation (z)
Date
Precipitation (z)
Streamflow (z)
G (m)
Q (m3/s)
ln(Q)
A (m2)
ln(A)
V (m/s)
ln(V)
W (m)
ln(W)
BE (m)
rho 0.92
rho 0.86
rho −0.15
rho −0.43
rho 0.074
rho 0.036
rho 0.30
rho 0.071
rho 0.20
rho 0.071
rho 0.37
rho 0.25
rho 0.085
rho −0.036
rho 0.066
rho −0.11
rho 0.18
rho −0.071
rho 0.43
rho 0.43
rho −0.12
rho 0.071
rho 0.28
rho −0.43

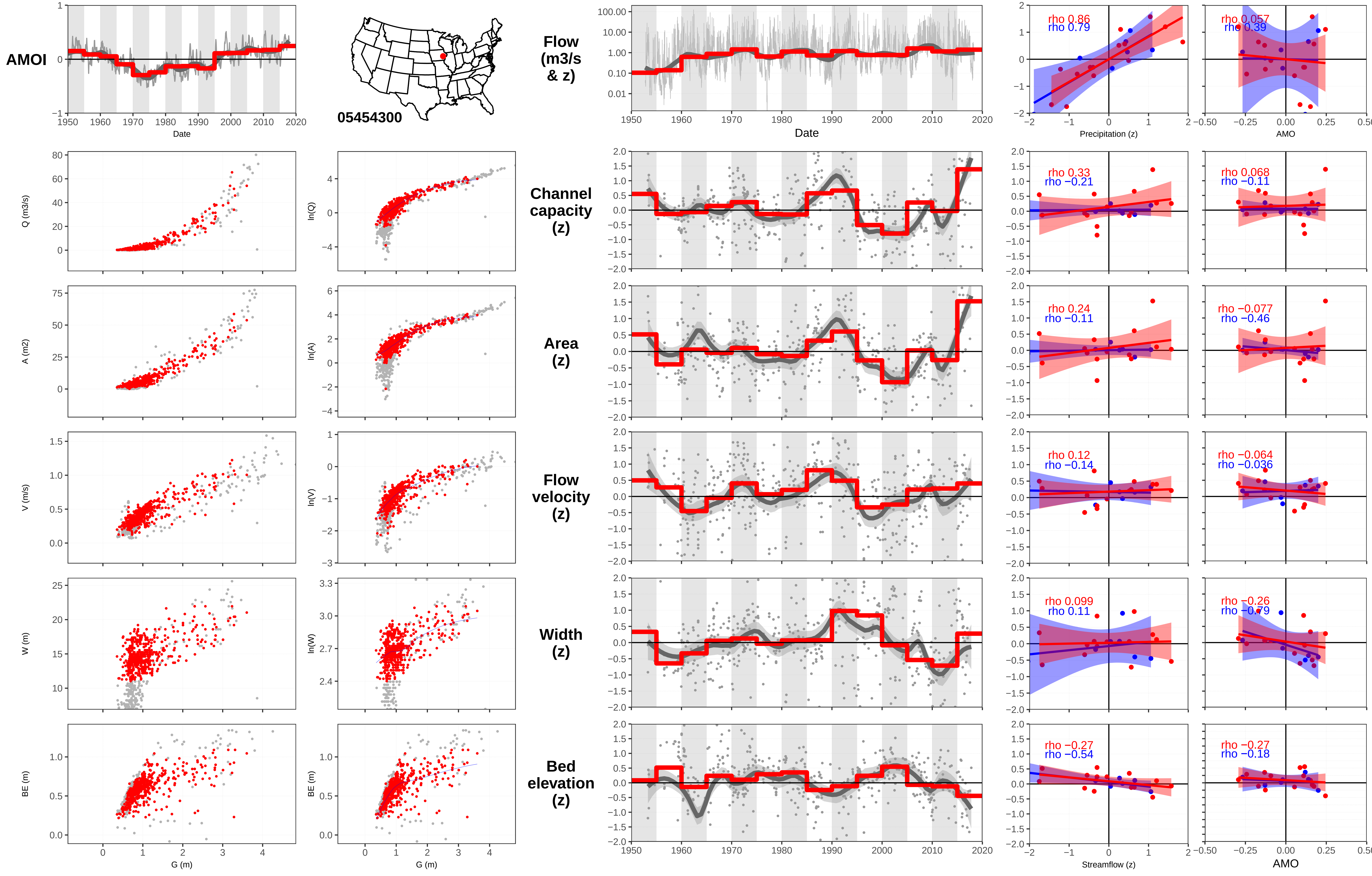
AMOI
05454300
Flow (m3/s & z)
Date
Precipitation (z)
AMO
rho 0.86
rho 0.79
rho 0.057
rho 0.39
Channel capacity (z)
Q (m3/s)
ln(Q)
rho 0.33
rho −0.21
rho 0.068
rho −0.11
Area (z)
A (m2)
ln(A)
rho 0.24
rho −0.11
rho −0.077
rho −0.46
Flow velocity (z)
V (m/s)
ln(V)
rho 0.12
rho −0.14
rho −0.064
rho −0.036
Width (z)
W (m)
ln(W)
rho 0.099
rho 0.11
rho −0.26
rho −0.79
Bed elevation (z)
BE (m)
rho −0.27
rho −0.54
rho −0.27
rho −0.18
G (m)
Streamflow (z)
AMO

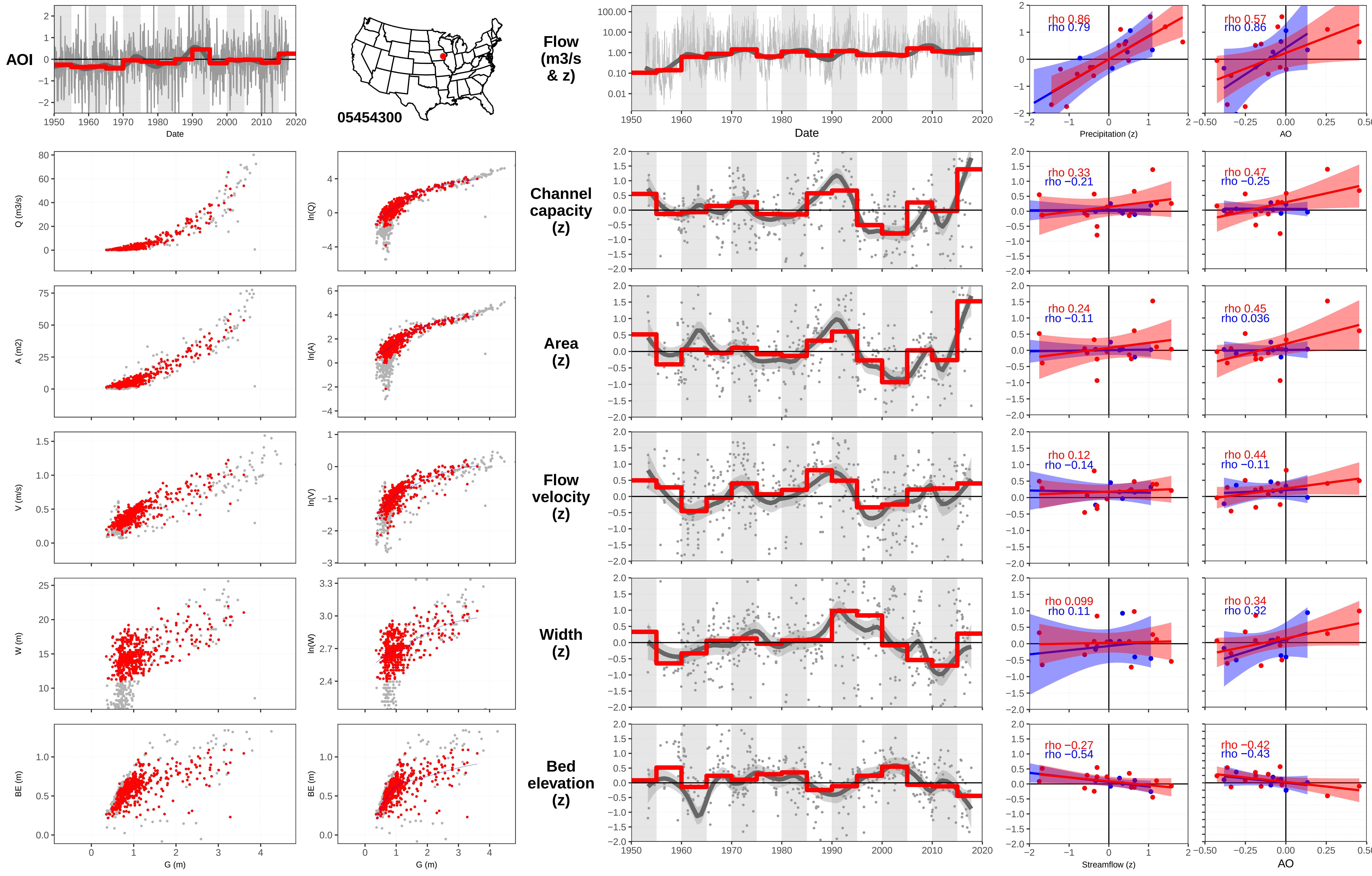

AOI
05454300
Flow (m3/s & z)
Channel capacity (z)
Area (z)
Flow velocity (z)
Width (z)
Bed elevation (z)
Date
Precipitation (z)
AO
Streamflow (z)
G (m)
Q (m3/s)
ln(Q)
A (m2)
ln(A)
V (m/s)
ln(V)
W (m)
ln(W)
BE (m)
rho 0.86
rho 0.79
rho 0.57
rho 0.86
rho 0.33
rho −0.21
rho 0.47
rho −0.25
rho 0.24
rho −0.11
rho 0.45
rho 0.036
rho 0.12
rho −0.14
rho 0.44
rho −0.11
rho 0.099
rho 0.11
rho 0.34
rho 0.32
rho −0.27
rho −0.54
rho −0.42
rho −0.43

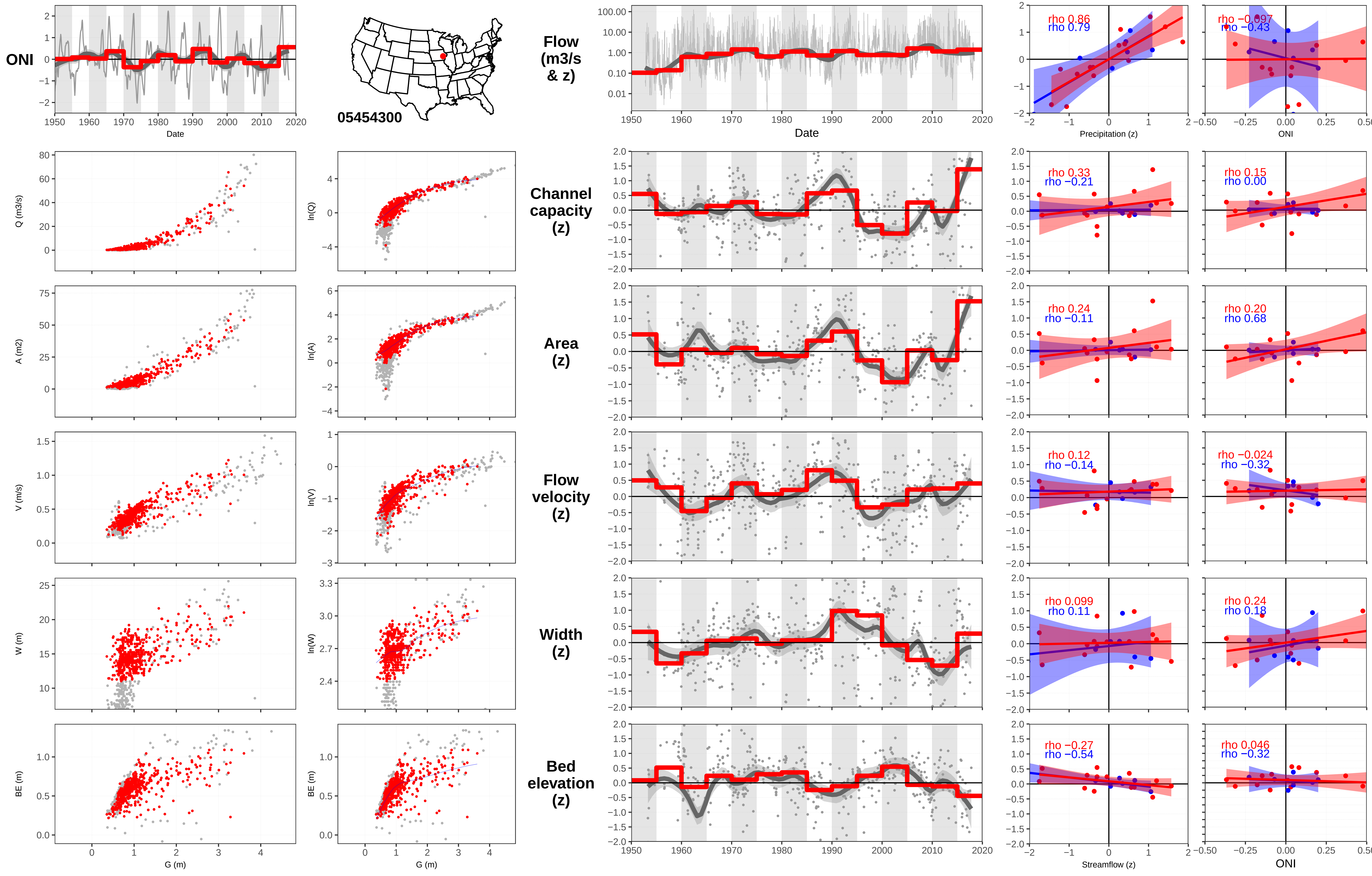

ONI
05454300
Flow (m3/s & z)
Channel capacity (z)
Area (z)
Flow velocity (z)
Width (z)
Bed elevation (z)
Date
Precipitation (z)
Streamflow (z)
ONI
G (m)
Q (m3/s)
ln(Q)
A (m2)
ln(A)
V (m/s)
ln(V)
W (m)
ln(W)
BE (m)
rho 0.86
rho 0.79
rho −0.097
rho −0.43
rho 0.33
rho −0.21
rho 0.15
rho 0.00
rho 0.24
rho −0.11
rho 0.20
rho 0.68
rho 0.12
rho −0.14
rho −0.024
rho −0.32
rho 0.099
rho 0.11
rho 0.24
rho 0.18
rho −0.27
rho −0.54
rho 0.046
rho −0.32

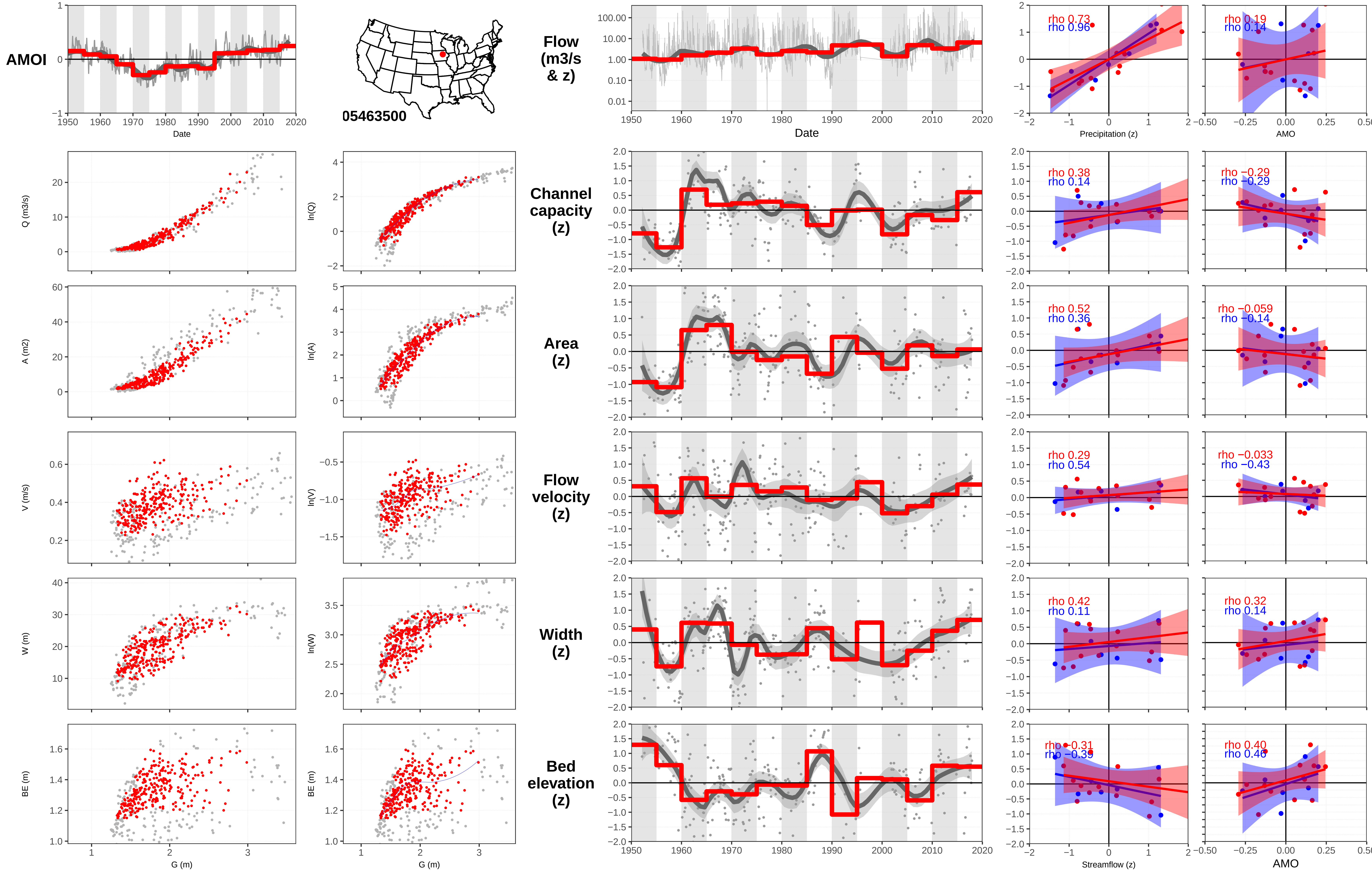
AMOI
05463500
Flow (m3/s & z)
Channel capacity (z)
Area (z)
Flow velocity (z)
Width (z)
Bed elevation (z)
Date
Precipitation (z)
AMO
Streamflow (z)
Q (m3/s)
ln(Q)
A (m2)
ln(A)
V (m/s)
ln(V)
W (m)
ln(W)
BE (m)
G (m)
rho 0.73
rho 0.96
rho 0.19
rho 0.14
rho 0.38
rho 0.14
rho −0.29
rho −0.29
rho 0.52
rho 0.36
rho −0.059
rho −0.14
rho 0.29
rho 0.54
rho −0.033
rho −0.43
rho 0.42
rho 0.11
rho 0.32
rho 0.14
rho −0.31
rho −0.39
rho 0.40
rho 0.46

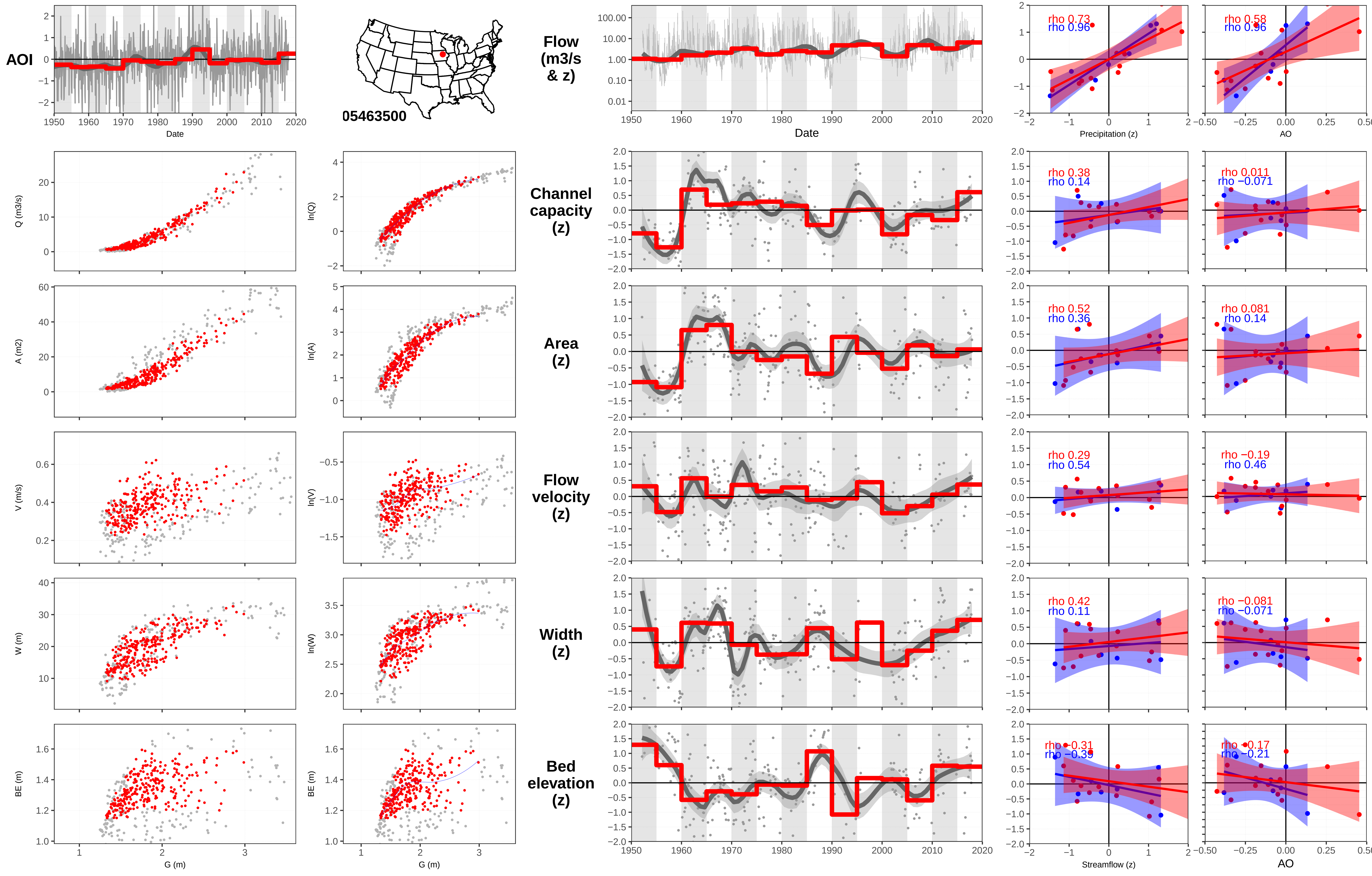

AOI
05463500
Flow (m3/s & z)
Channel capacity (z)
Area (z)
Flow velocity (z)
Width (z)
Bed elevation (z)
Date
Precipitation (z)
AO
Streamflow (z)
G (m)
Q (m3/s)
ln(Q)
A (m2)
ln(A)
V (m/s)
ln(V)
W (m)
ln(W)
BE (m)
rho 0.73
rho 0.96
rho 0.58
rho 0.96
rho 0.38
rho 0.14
rho 0.011
rho −0.071
rho 0.52
rho 0.36
rho 0.081
rho 0.14
rho 0.29
rho 0.54
rho −0.19
rho 0.46
rho 0.42
rho 0.11
rho −0.081
rho −0.071
rho −0.31
rho −0.39
rho −0.17
rho −0.21

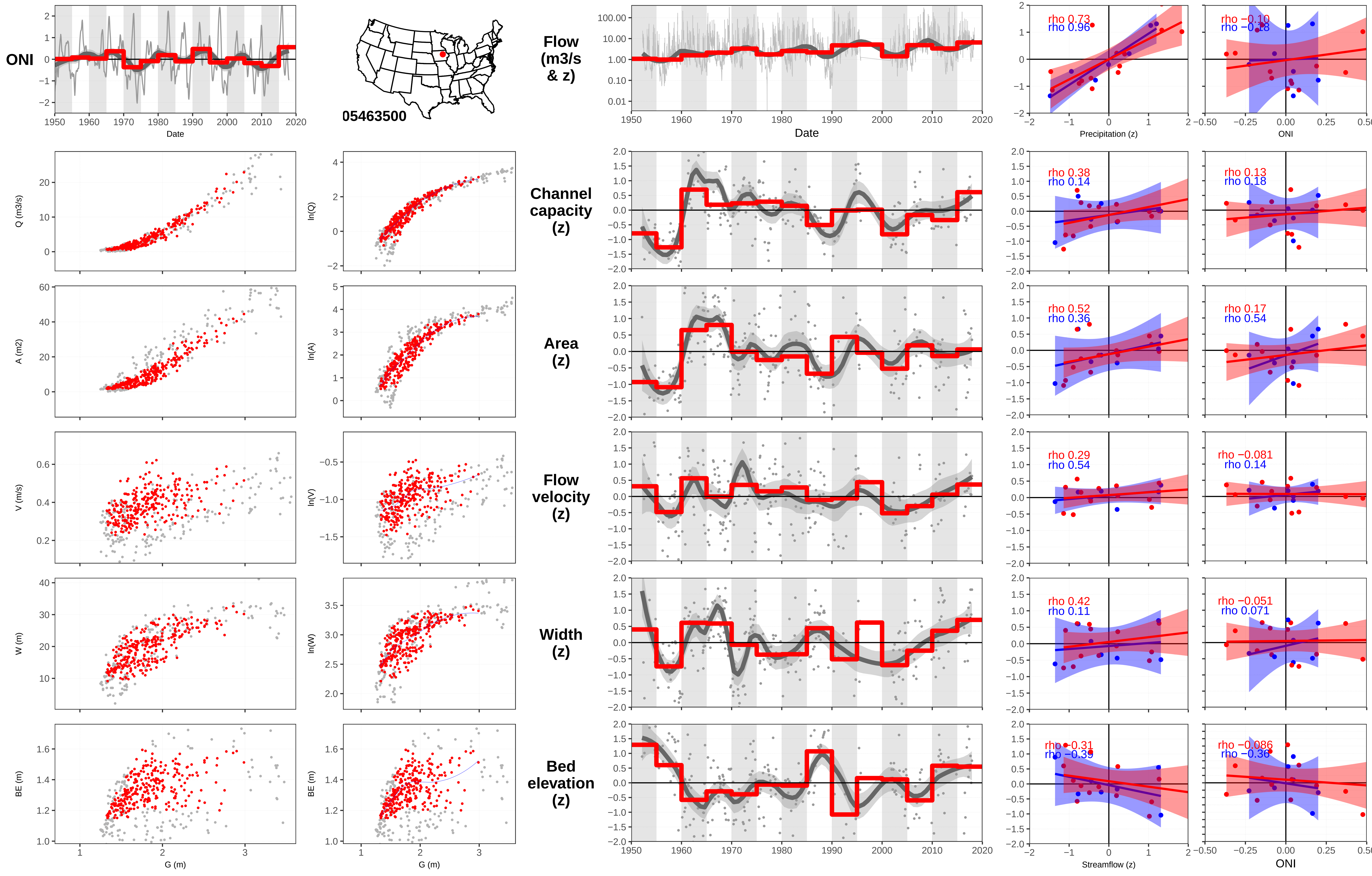
ONI
05463500
Flow (m3/s & z)
Channel capacity (z)
Area (z)
Flow velocity (z)
Width (z)
Bed elevation (z)
Date
Precipitation (z)
Streamflow (z)
ONI
G (m)
Q (m3/s)
ln(Q)
A (m2)
ln(A)
V (m/s)
ln(V)
W (m)
ln(W)
BE (m)
rho 0.73
rho 0.96
rho −0.10
rho −0.18
rho 0.38
rho 0.14
rho 0.13
rho 0.18
rho 0.52
rho 0.36
rho 0.17
rho 0.54
rho 0.29
rho 0.54
rho −0.081
rho 0.14
rho 0.42
rho 0.11
rho −0.051
rho 0.071
rho −0.31
rho −0.39
rho −0.086
rho −0.36

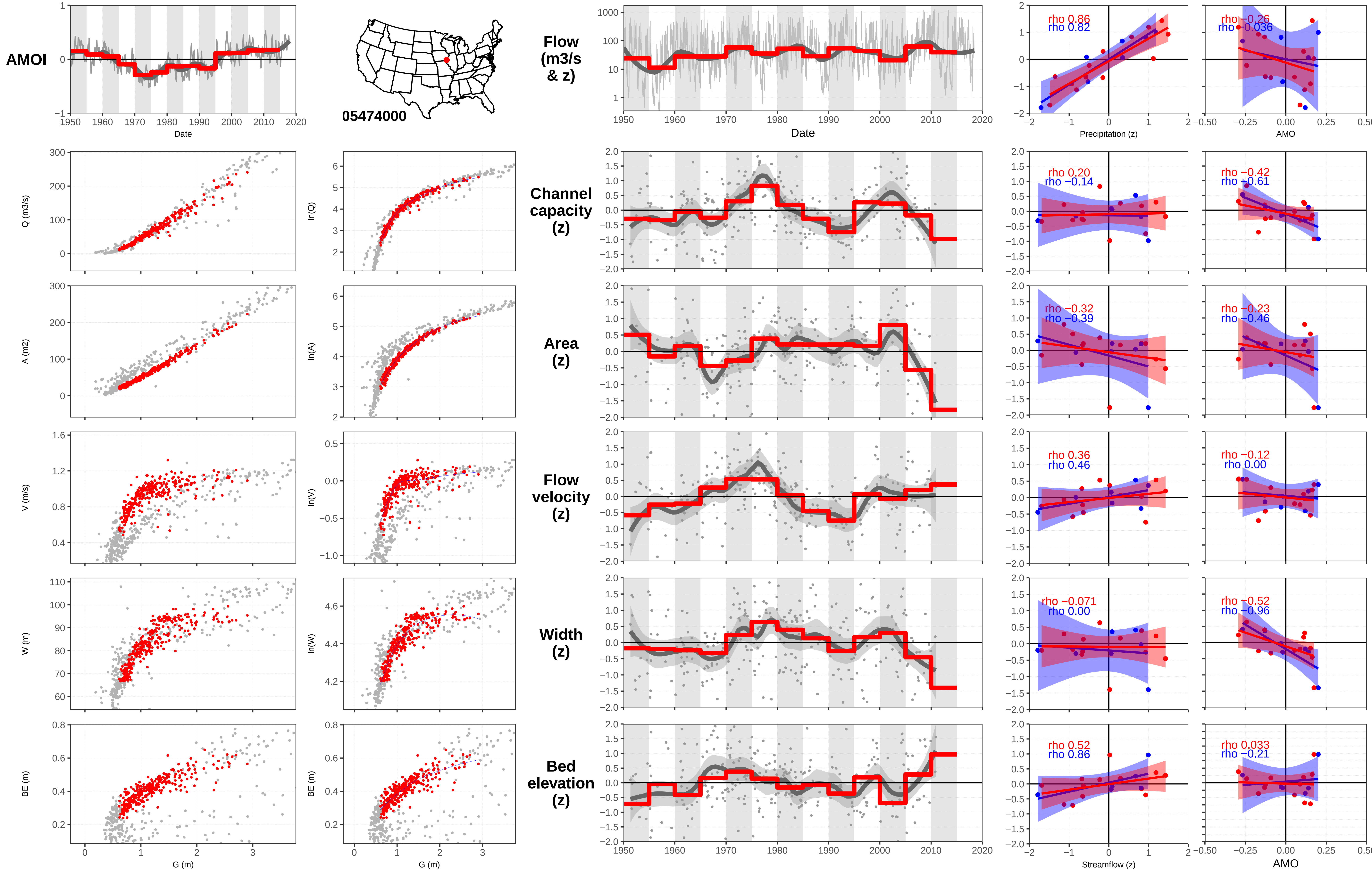

AMOI
05474000
Flow (m3/s & z)
Channel capacity (z)
Area (z)
Flow velocity (z)
Width (z)
Bed elevation (z)
Date
Precipitation (z)
Streamflow (z)
AMO
G (m)
Q (m3/s)
ln(Q)
A (m2)
ln(A)
V (m/s)
ln(V)
W (m)
ln(W)
BE (m)
rho 0.86
rho 0.82
rho −0.26
rho −0.036
rho 0.20
rho −0.14
rho −0.42
rho −0.61
rho −0.32
rho −0.39
rho −0.23
rho −0.46
rho 0.36
rho 0.46
rho −0.12
rho 0.00
rho −0.071
rho 0.00
rho −0.52
rho −0.96
rho 0.52
rho 0.86
rho 0.033
rho −0.21

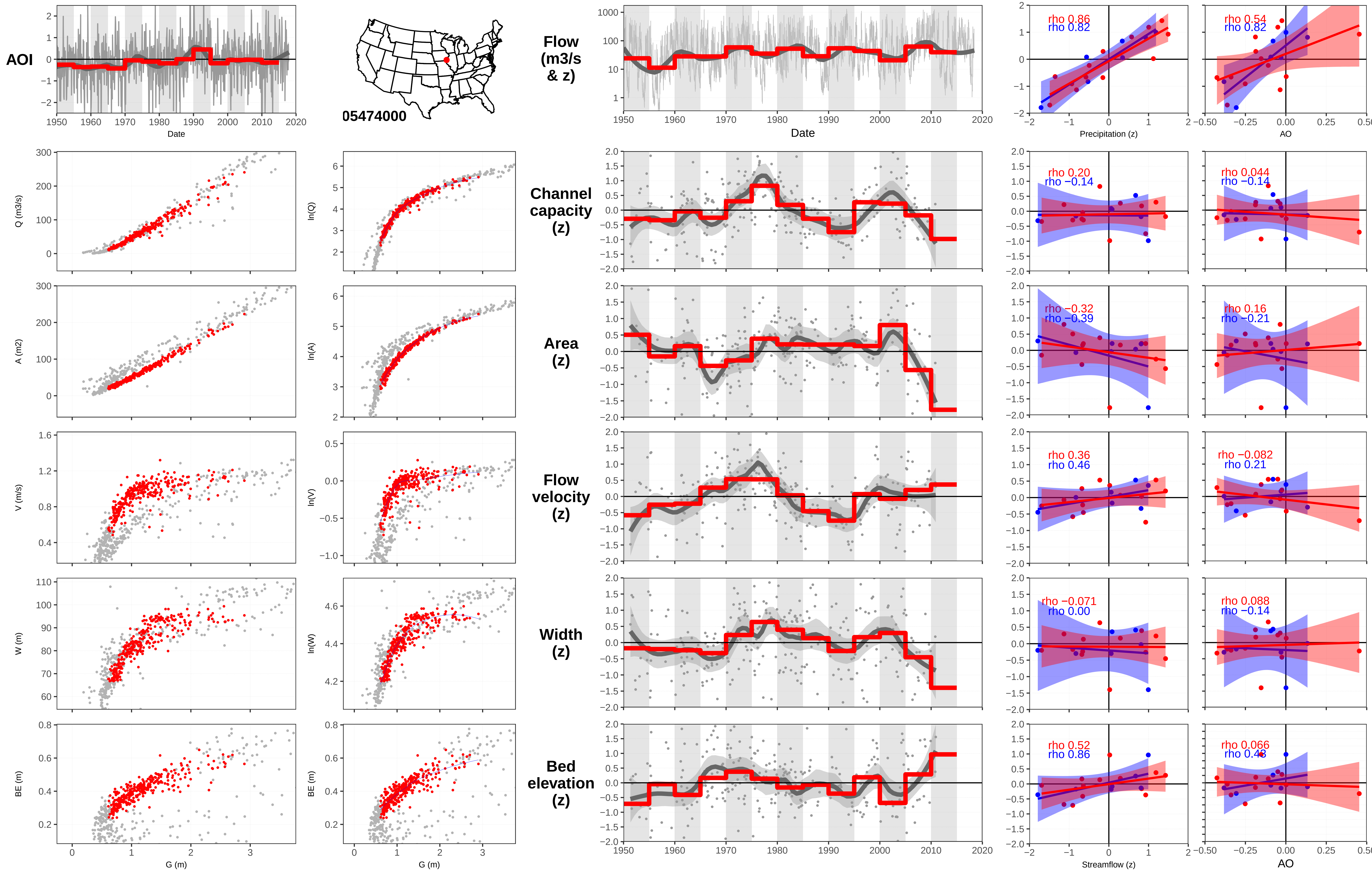

AOI
05474000
Flow (m3/s & z)
Channel capacity (z)
Area (z)
Flow velocity (z)
Width (z)
Bed elevation (z)
Date
Precipitation (z)
Streamflow (z)
AO
Q (m3/s)
ln(Q)
A (m2)
ln(A)
V (m/s)
ln(V)
W (m)
ln(W)
BE (m)
G (m)
rho 0.86
rho 0.82
rho 0.54
rho 0.82
rho 0.20
rho −0.14
rho 0.044
rho −0.14
rho −0.32
rho −0.39
rho 0.16
rho −0.21
rho 0.36
rho 0.46
rho −0.082
rho 0.21
rho −0.071
rho 0.00
rho 0.088
rho −0.14
rho 0.52
rho 0.86
rho 0.066
rho 0.43

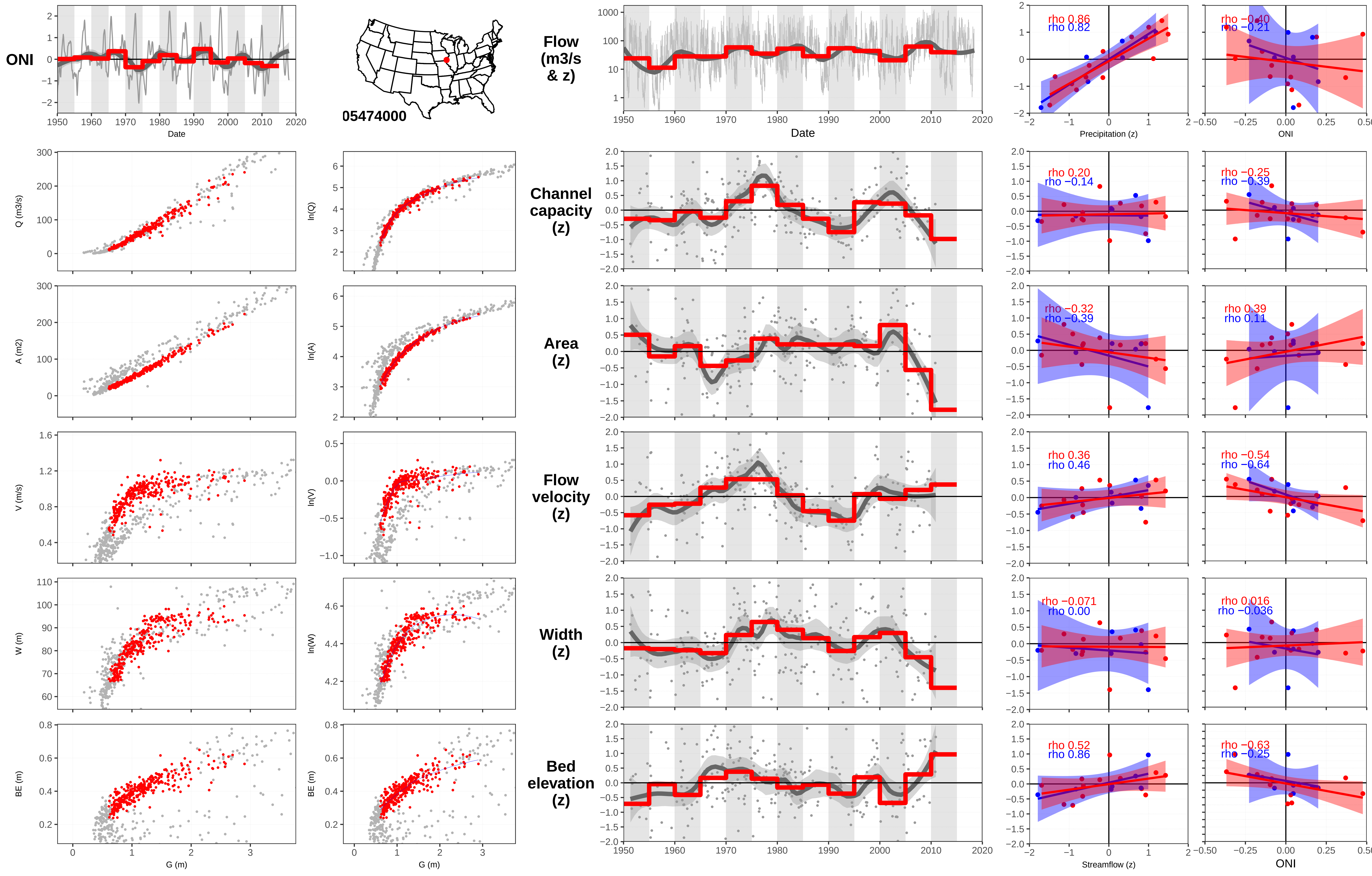

ONI
Date
05474000
Flow (m3/s & z)
Date
rho 0.86
rho 0.82
Precipitation (z)
rho −0.40
rho −0.21
ONI
Q (m3/s)
ln(Q)
Channel capacity (z)
rho 0.20
rho −0.14
rho −0.25
rho −0.39
A (m2)
ln(A)
Area (z)
rho −0.32
rho −0.39
rho 0.39
rho 0.11
V (m/s)
ln(V)
Flow velocity (z)
rho 0.36
rho 0.46
rho −0.54
rho −0.64
W (m)
ln(W)
Width (z)
rho −0.071
rho 0.00
rho 0.016
rho −0.036
BE (m)
BE (m)
Bed elevation (z)
rho 0.52
rho 0.86
rho −0.63
rho −0.25
G (m)
G (m)
Streamflow (z)
ONI

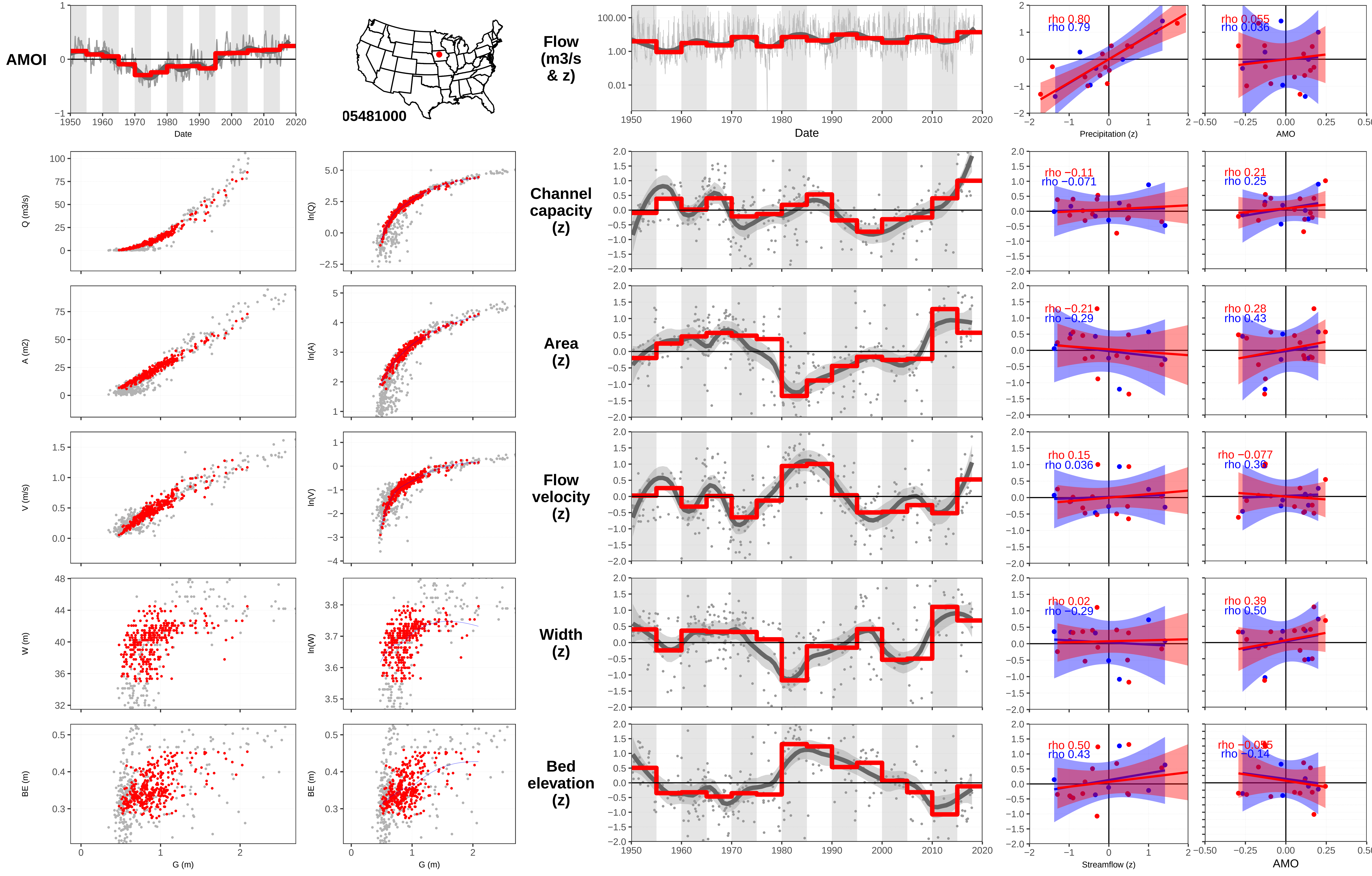

AMOI
05481000
Flow (m3/s & z)
Channel capacity (z)
Area (z)
Flow velocity (z)
Width (z)
Bed elevation (z)
Date
Precipitation (z)
Streamflow (z)
AMO
G (m)
Q (m3/s)
ln(Q)
A (m2)
ln(A)
V (m/s)
ln(V)
W (m)
ln(W)
BE (m)
rho 0.80
rho 0.79
rho 0.055
rho 0.036
rho −0.11
rho −0.071
rho 0.21
rho 0.25
rho −0.21
rho −0.29
rho 0.28
rho 0.43
rho 0.15
rho 0.036
rho −0.077
rho 0.36
rho 0.02
rho −0.29
rho 0.39
rho 0.50
rho 0.50
rho 0.43
rho −0.055
rho −0.14

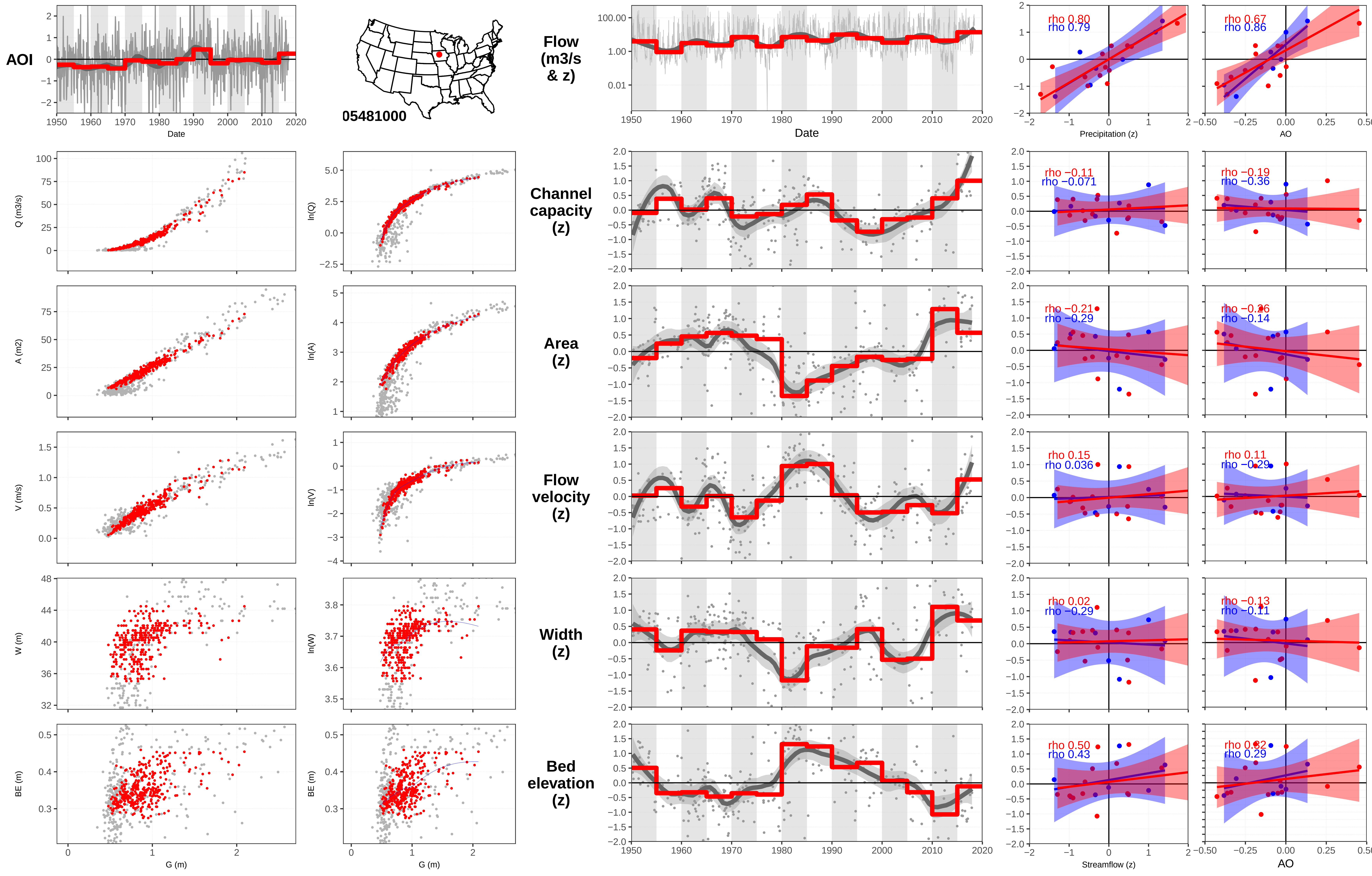

AOI
05481000
Date
Flow (m3/s & z)
Precipitation (z)
AO
rho 0.80
rho 0.79
rho 0.67
rho 0.86
Channel capacity (z)
Q (m3/s)
ln(Q)
rho −0.11
rho −0.071
rho −0.19
rho −0.36
Area (z)
A (m2)
ln(A)
rho −0.21
rho −0.29
rho −0.26
rho −0.14
Flow velocity (z)
V (m/s)
ln(V)
rho 0.15
rho 0.036
rho 0.11
rho −0.29
Width (z)
W (m)
ln(W)
rho 0.02
rho −0.29
rho −0.13
rho −0.11
Bed elevation (z)
BE (m)
rho 0.50
rho 0.43
rho 0.32
rho 0.29
G (m)
Streamflow (z)
AO

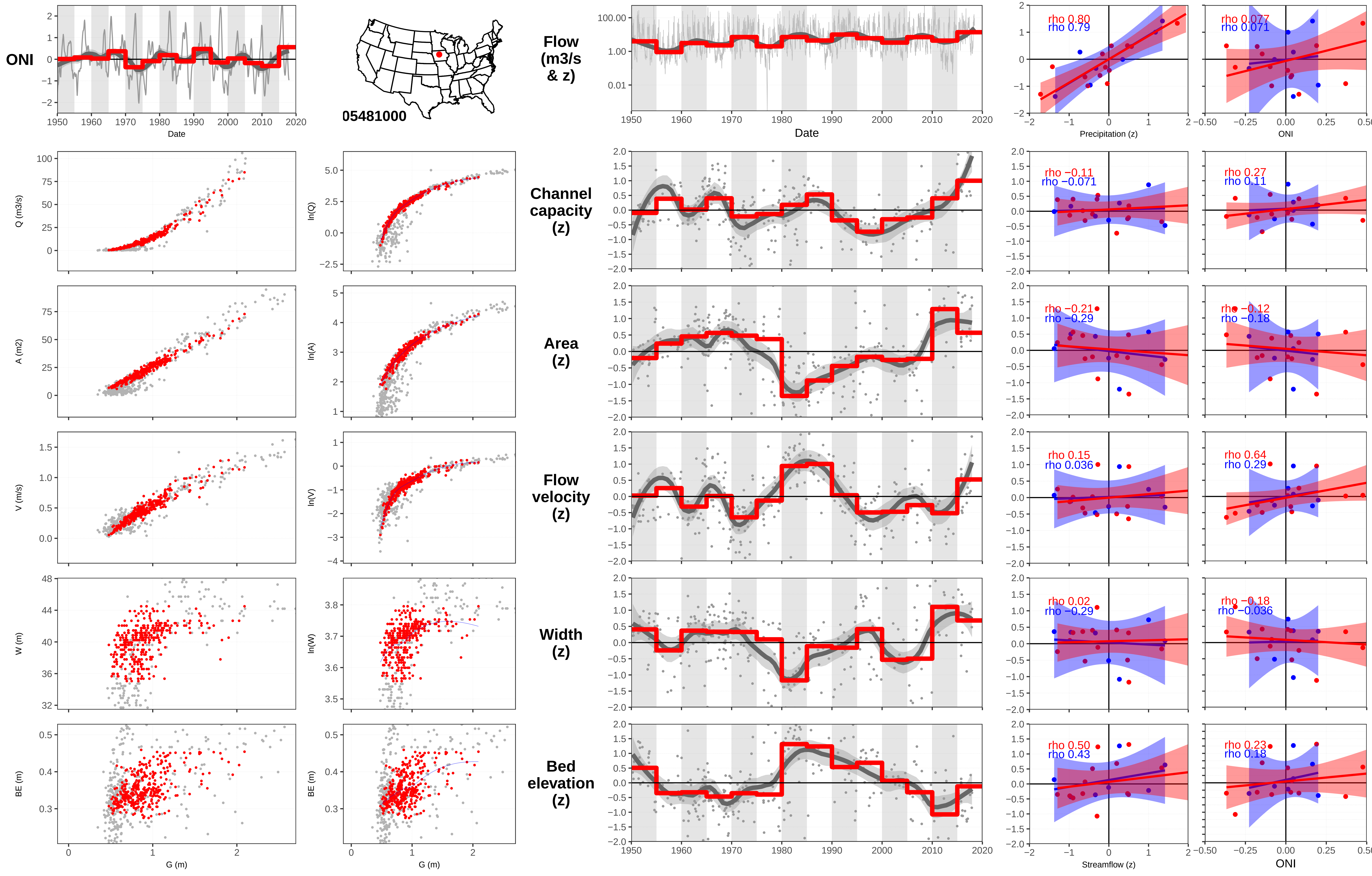
ONI
05481000
Flow (m3/s & z)
Channel capacity (z)
Area (z)
Flow velocity (z)
Width (z)
Bed elevation (z)
Date
Precipitation (z)
Streamflow (z)
ONI
G (m)
Q (m3/s)
ln(Q)
A (m2)
ln(A)
V (m/s)
ln(V)
W (m)
ln(W)
BE (m)
rho 0.80
rho 0.79
rho 0.077
rho 0.071
rho −0.11
rho −0.071
rho 0.27
rho 0.11
rho −0.21
rho −0.29
rho −0.12
rho −0.18
rho 0.15
rho 0.036
rho 0.64
rho 0.29
rho 0.02
rho −0.29
rho −0.18
rho −0.036
rho 0.50
rho 0.43
rho 0.23
rho 0.18

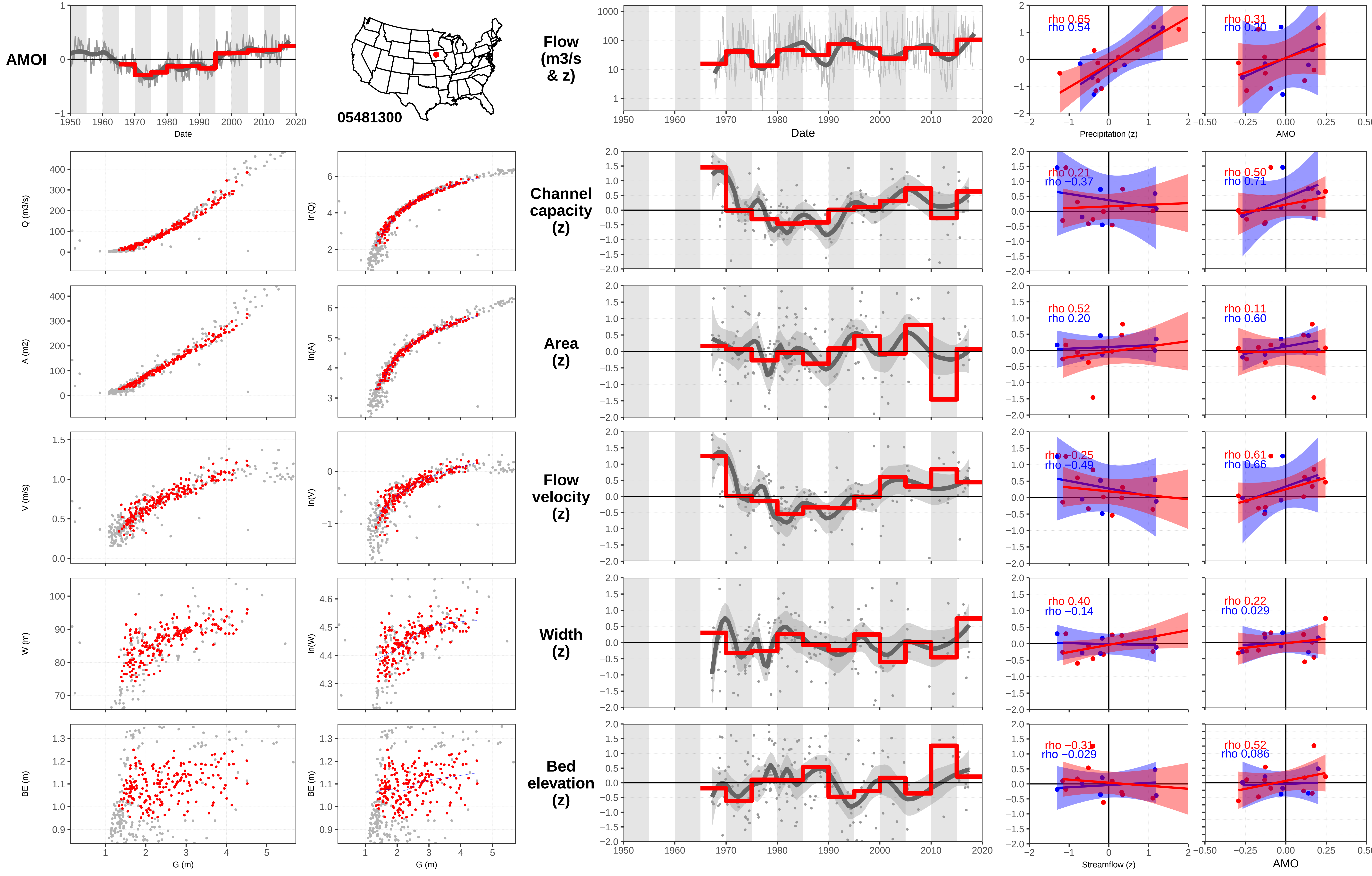
AMOI
Date
05481300
Flow (m3/s & z)
Date
Channel capacity (z)
Area (z)
Flow velocity (z)
Width (z)
Bed elevation (z)
Q (m3/s)
ln(Q)
A (m2)
ln(A)
V (m/s)
ln(V)
W (m)
ln(W)
BE (m)
G (m)
Precipitation (z)
Streamflow (z)
AMO
rho 0.65
rho 0.54
rho 0.31
rho 0.20
rho 0.21
rho −0.37
rho 0.50
rho 0.71
rho 0.52
rho 0.20
rho 0.11
rho 0.60
rho −0.25
rho −0.49
rho 0.61
rho 0.66
rho 0.40
rho −0.14
rho 0.22
rho 0.029
rho −0.31
rho −0.029
rho 0.52
rho 0.086

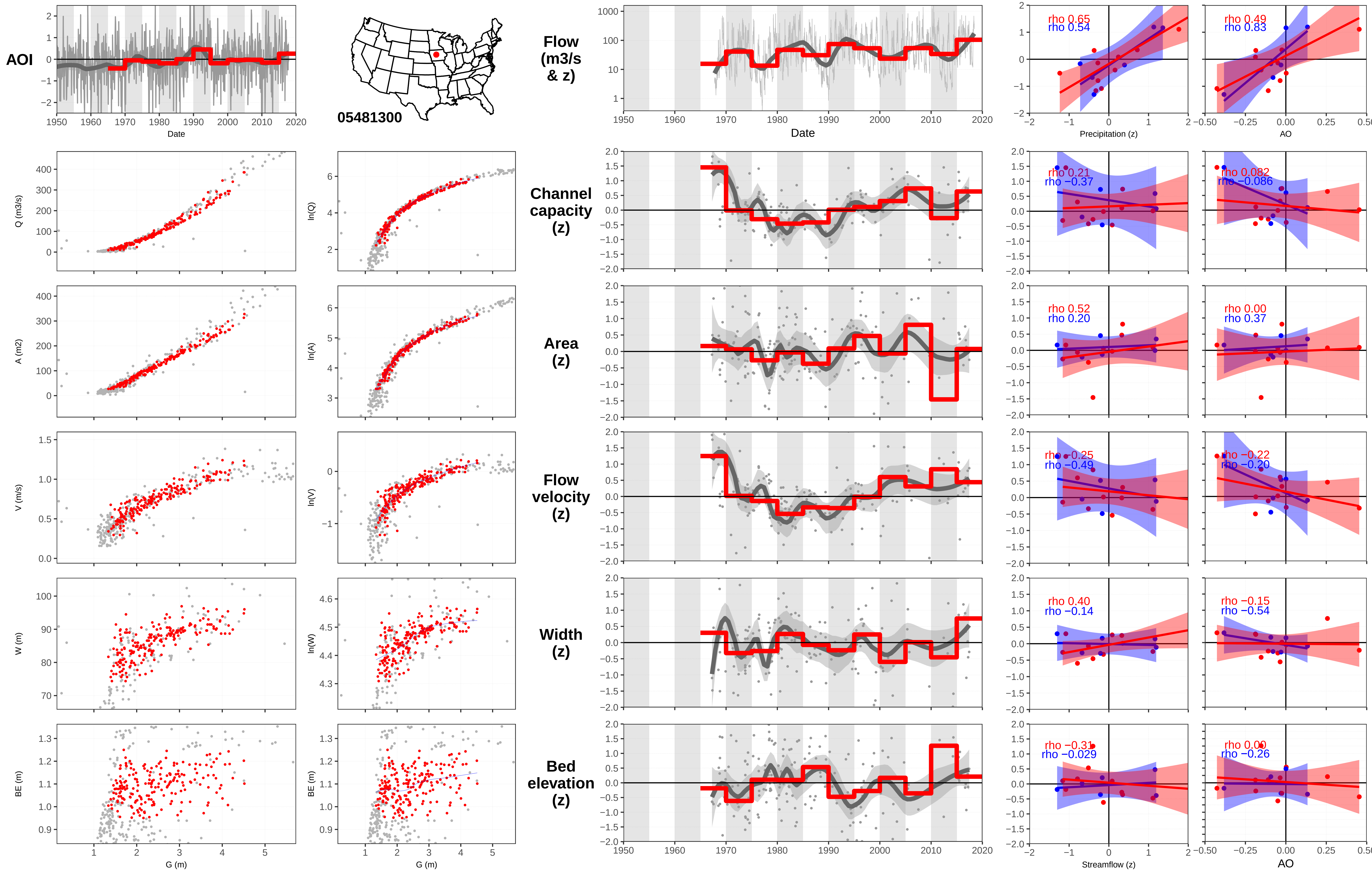

AOI
05481300
Flow (m3/s & z)
Channel capacity (z)
Area (z)
Flow velocity (z)
Width (z)
Bed elevation (z)
Date
Precipitation (z)
AO
Streamflow (z)
G (m)
Q (m3/s)
ln(Q)
A (m2)
ln(A)
V (m/s)
ln(V)
W (m)
ln(W)
BE (m)
rho 0.65
rho 0.54
rho 0.49
rho 0.83
rho 0.21
rho −0.37
rho 0.082
rho −0.086
rho 0.52
rho 0.20
rho 0.00
rho 0.37
rho −0.25
rho −0.49
rho −0.22
rho −0.20
rho 0.40
rho −0.14
rho −0.15
rho −0.54
rho −0.31
rho −0.029
rho 0.00
rho −0.26

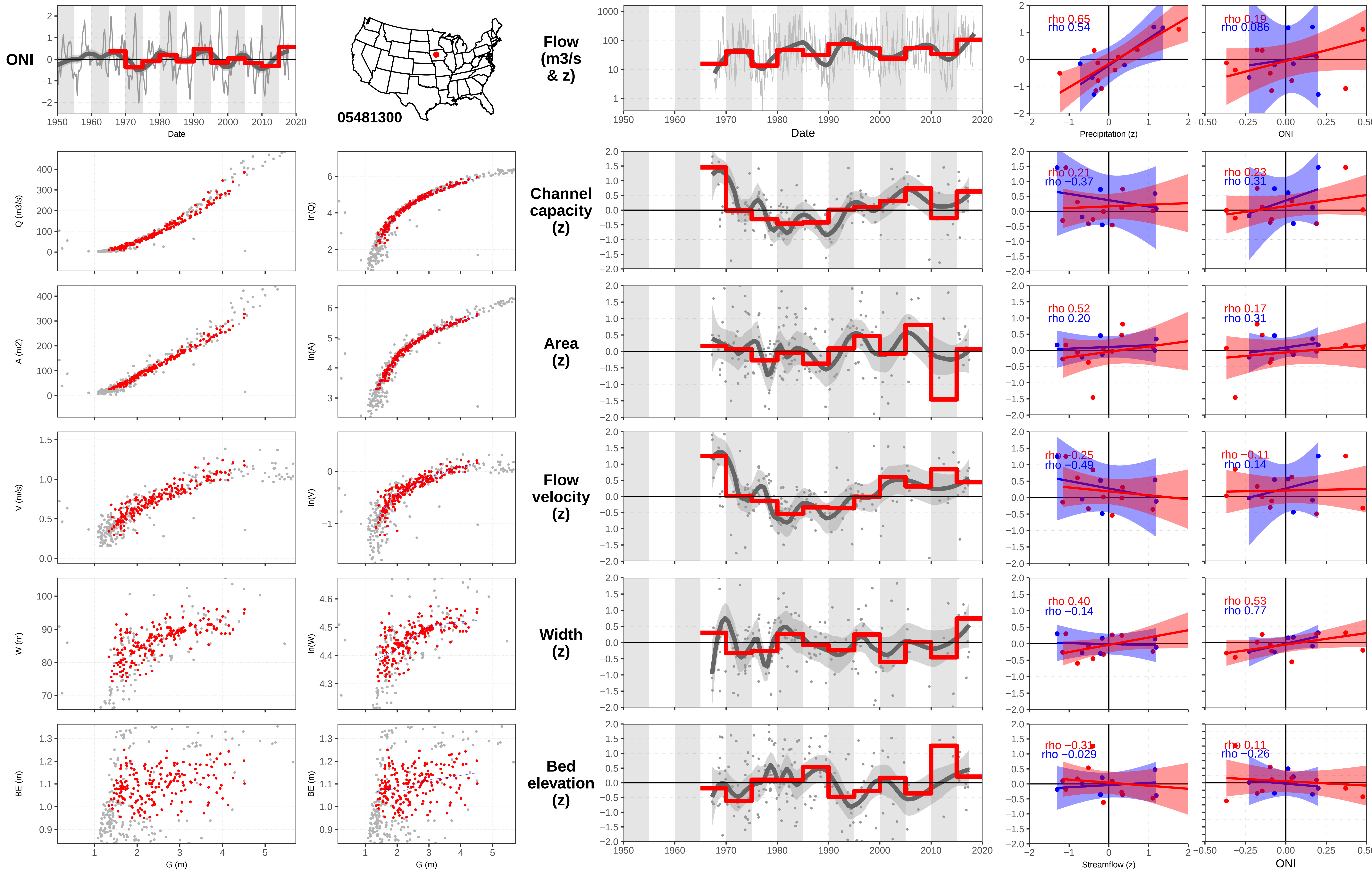
ONI
05481300
Flow (m3/s & z)
Channel capacity (z)
Area (z)
Flow velocity (z)
Width (z)
Bed elevation (z)
Date
Precipitation (z)
Streamflow (z)
ONI
G (m)
Q (m3/s)
ln(Q)
A (m2)
ln(A)
V (m/s)
ln(V)
W (m)
ln(W)
BE (m)
rho 0.65
rho 0.54
rho 0.19
rho 0.086
rho 0.21
rho −0.37
rho 0.23
rho 0.31
rho 0.52
rho 0.20
rho 0.17
rho 0.31
rho −0.25
rho −0.49
rho −0.11
rho 0.14
rho 0.40
rho −0.14
rho 0.53
rho 0.77
rho −0.31
rho −0.029
rho 0.11
rho −0.26

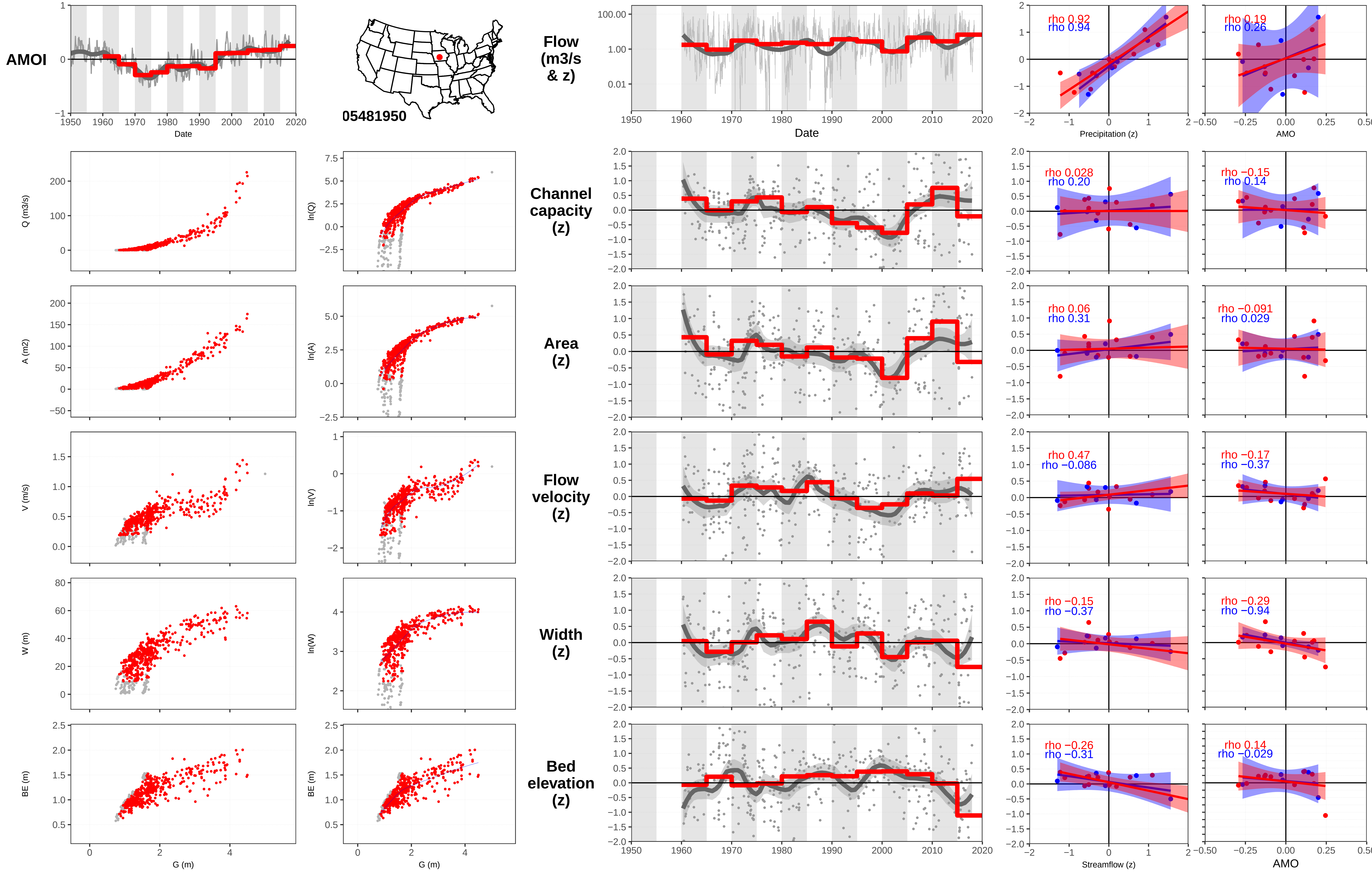

AMOI
05481950
Flow (m3/s & z)
Channel capacity (z)
Area (z)
Flow velocity (z)
Width (z)
Bed elevation (z)
Date
Precipitation (z)
AMO
Streamflow (z)
G (m)
Q (m3/s)
ln(Q)
A (m2)
ln(A)
V (m/s)
ln(V)
W (m)
ln(W)
BE (m)
rho 0.92
rho 0.94
rho 0.19
rho 0.26
rho 0.028
rho 0.20
rho −0.15
rho 0.14
rho 0.06
rho 0.31
rho −0.091
rho 0.029
rho 0.47
rho −0.086
rho −0.17
rho −0.37
rho −0.15
rho −0.37
rho −0.29
rho −0.94
rho −0.26
rho −0.31
rho 0.14
rho −0.029

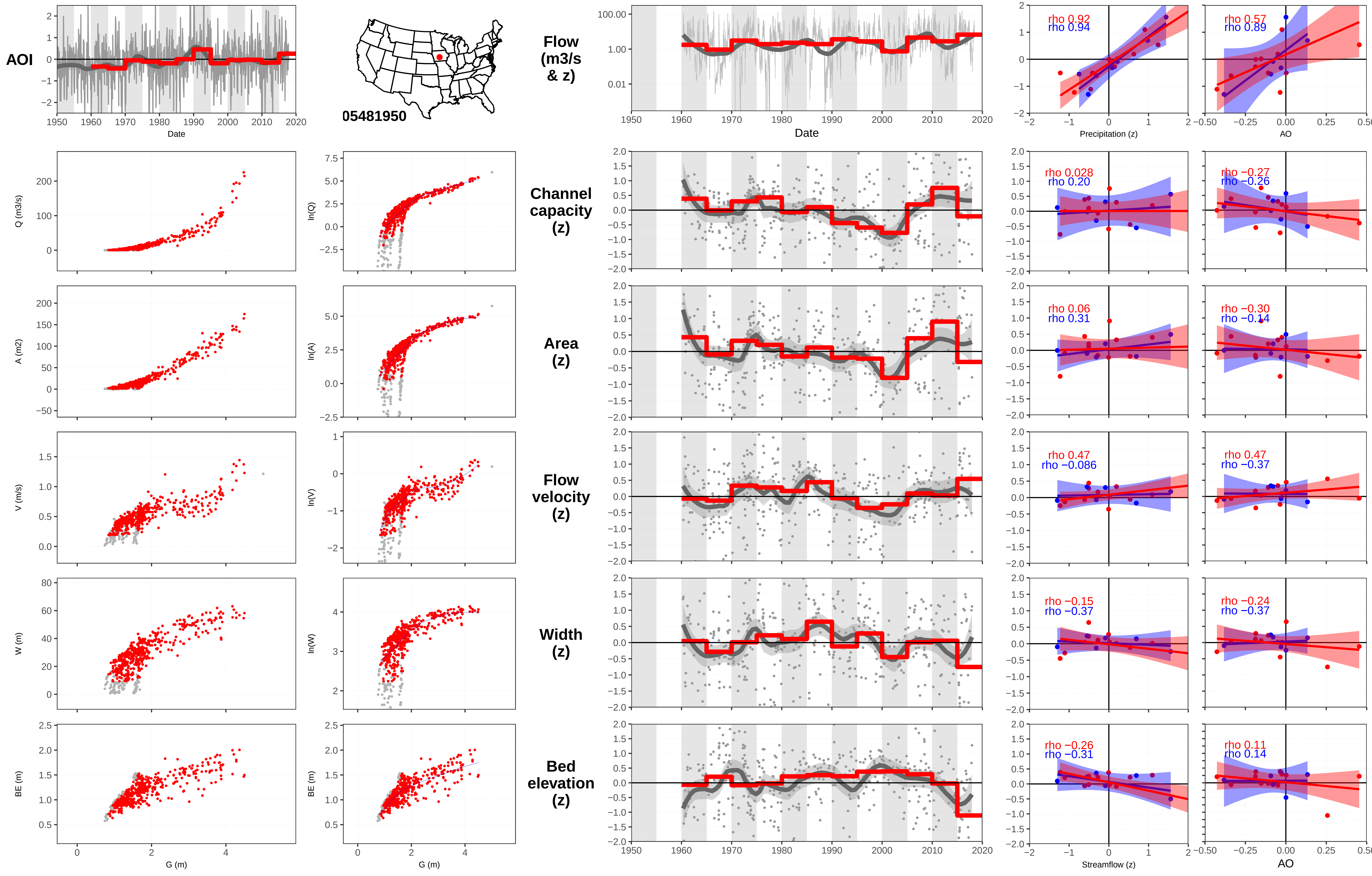

AOI
05481950
Flow (m3/s & z)
Channel capacity (z)
Area (z)
Flow velocity (z)
Width (z)
Bed elevation (z)
Date
Precipitation (z)
AO
Streamflow (z)
G (m)
Q (m3/s)
ln(Q)
A (m2)
ln(A)
V (m/s)
ln(V)
W (m)
ln(W)
BE (m)
rho 0.92
rho 0.94
rho 0.57
rho 0.89
rho 0.028
rho 0.20
rho −0.27
rho −0.26
rho 0.06
rho 0.31
rho −0.30
rho −0.14
rho 0.47
rho −0.086
rho 0.47
rho −0.37
rho −0.15
rho −0.37
rho −0.24
rho −0.37
rho −0.26
rho −0.31
rho 0.11
rho 0.14

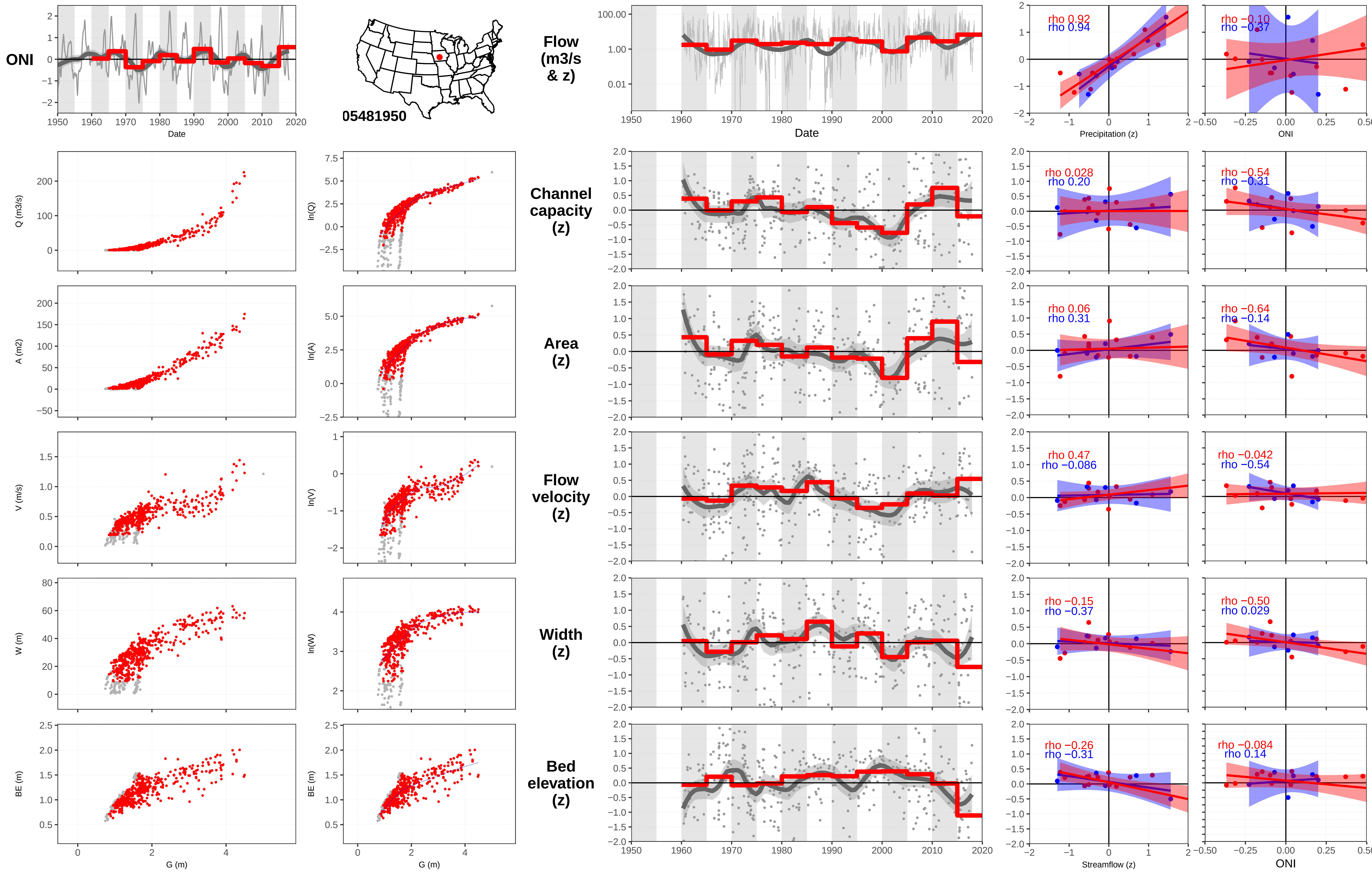

ONI
Date
05481950
Flow (m3/s & z)
Date
rho 0.92
rho 0.94
Precipitation (z)
rho −0.10
rho −0.37
ONI
Q (m3/s)
ln(Q)
Channel capacity (z)
rho 0.028
rho 0.20
rho −0.54
rho −0.31
A (m2)
ln(A)
Area (z)
rho 0.06
rho 0.31
rho −0.64
rho −0.14
V (m/s)
ln(V)
Flow velocity (z)
rho 0.47
rho −0.086
rho −0.042
rho −0.54
W (m)
ln(W)
Width (z)
rho −0.15
rho −0.37
rho −0.50
rho 0.029
BE (m)
BE (m)
Bed elevation (z)
rho −0.26
rho −0.31
rho −0.084
rho 0.14
G (m)
G (m)
Streamflow (z)
ONI

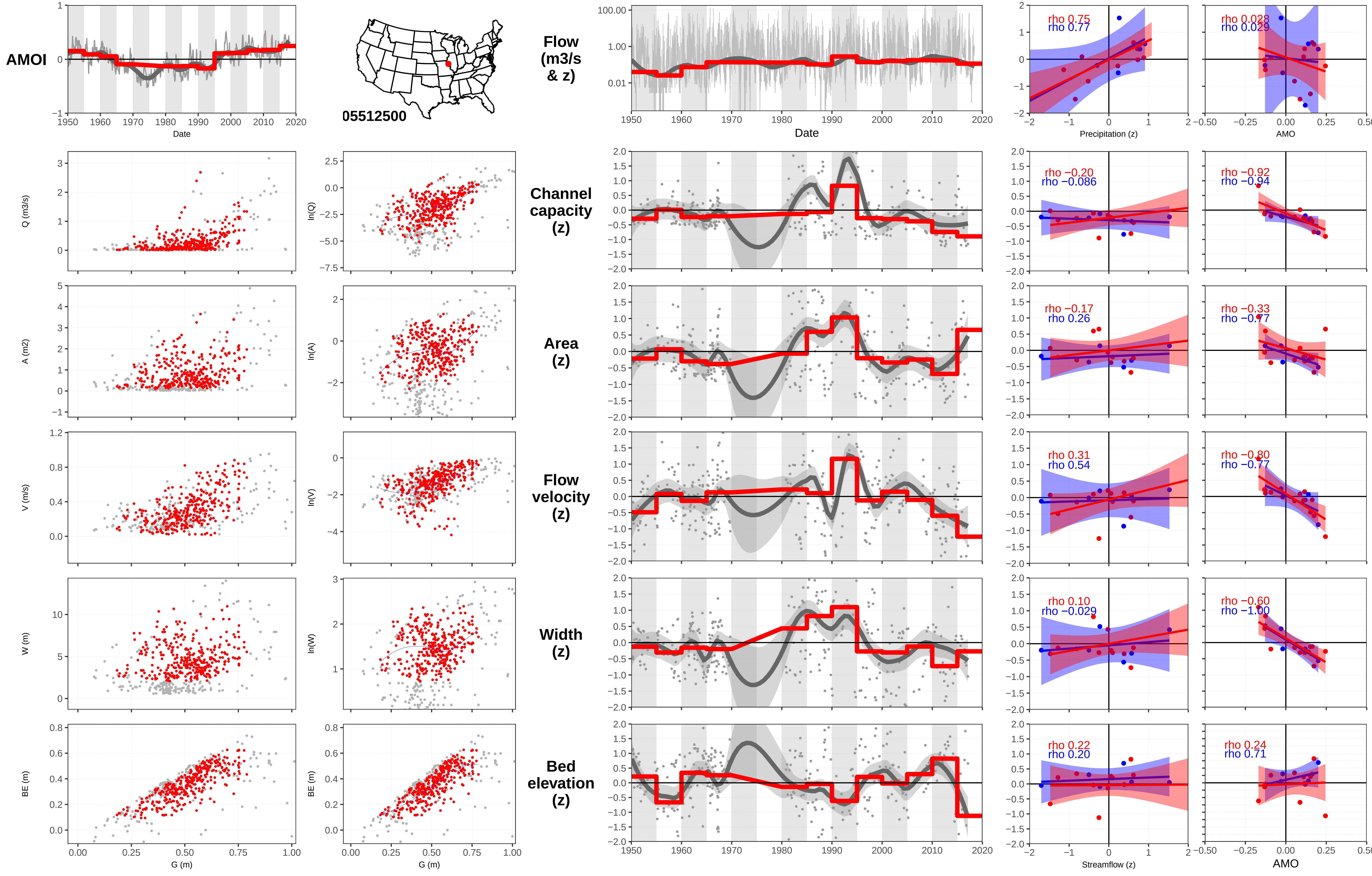

AMOI
05512500
Flow (m3/s & z)
Channel capacity (z)
Area (z)
Flow velocity (z)
Width (z)
Bed elevation (z)
Date
Precipitation (z)
AMO
Streamflow (z)
G (m)
Q (m3/s)
ln(Q)
A (m2)
ln(A)
V (m/s)
ln(V)
W (m)
ln(W)
BE (m)
rho 0.75
rho 0.77
rho 0.028
rho 0.029
rho −0.20
rho −0.086
rho −0.92
rho −0.94
rho −0.17
rho 0.26
rho −0.33
rho −0.77
rho 0.31
rho 0.54
rho −0.80
rho −0.77
rho 0.10
rho −0.029
rho −0.60
rho −1.00
rho 0.22
rho 0.20
rho 0.24
rho 0.71

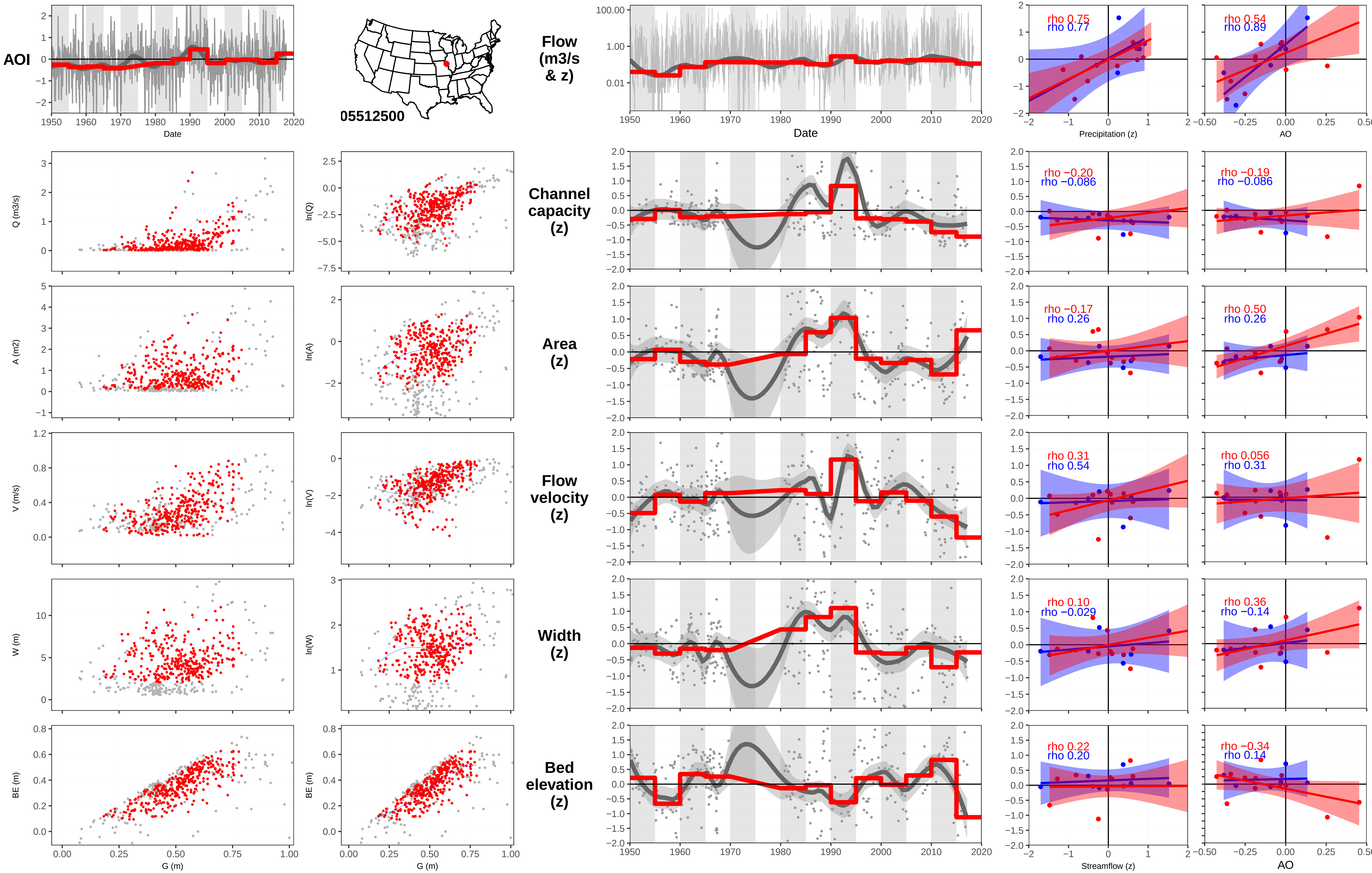

AOI
05512500
Flow (m3/s & z)
Channel capacity (z)
Area (z)
Flow velocity (z)
Width (z)
Bed elevation (z)
Date
Precipitation (z)
Streamflow (z)
AO
G (m)
Q (m3/s)
ln(Q)
A (m2)
ln(A)
V (m/s)
ln(V)
W (m)
ln(W)
BE (m)
rho 0.75
rho 0.77
rho 0.54
rho 0.89
rho −0.20
rho −0.086
rho −0.19
rho −0.086
rho −0.17
rho 0.26
rho 0.50
rho 0.26
rho 0.31
rho 0.54
rho 0.056
rho 0.31
rho 0.10
rho −0.029
rho 0.36
rho −0.14
rho 0.22
rho 0.20
rho −0.34
rho 0.14

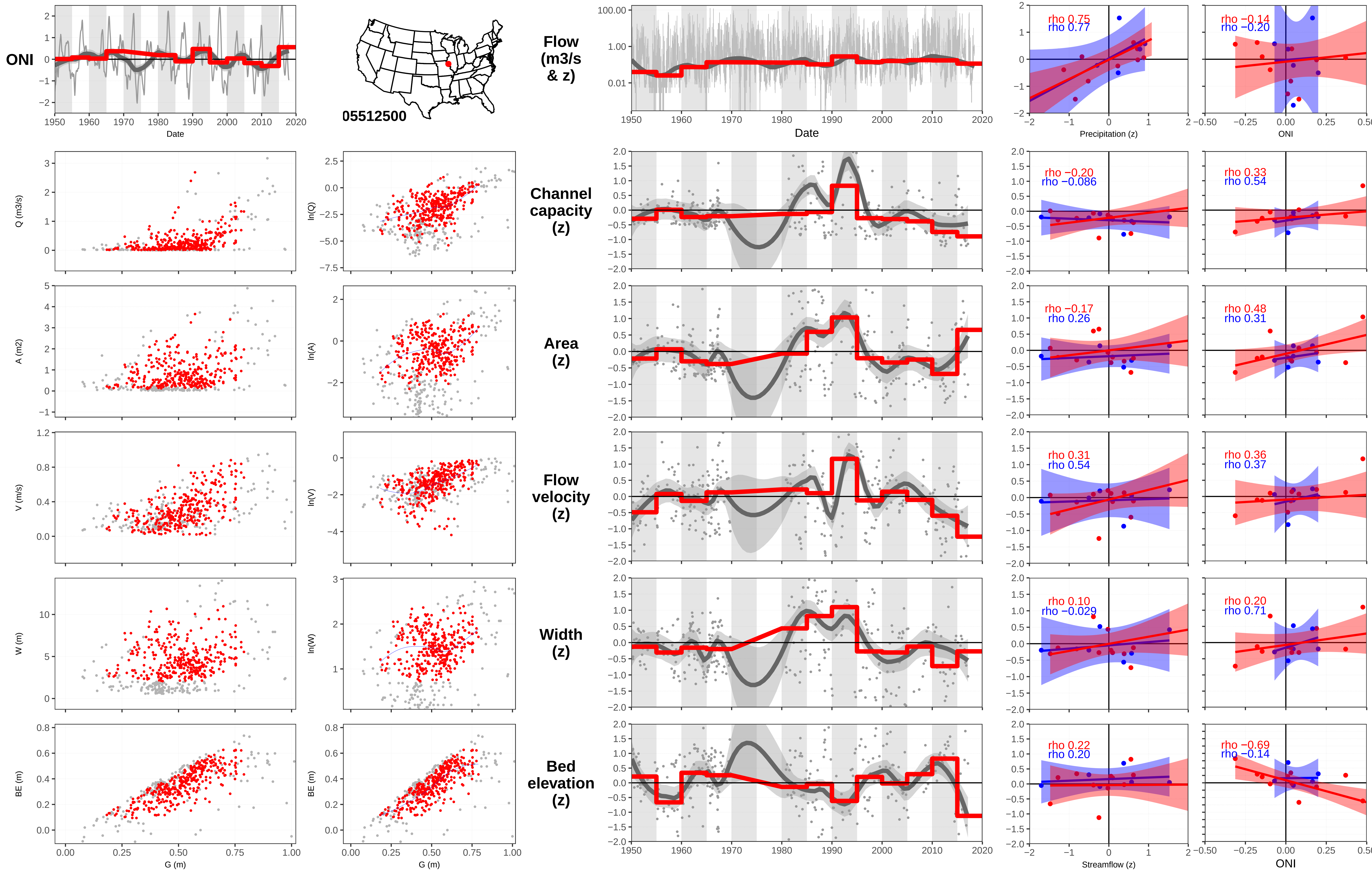
ONI
Date
05512500
Flow (m3/s & z)
Date
rho 0.75
rho 0.77
Precipitation (z)
rho −0.14
rho −0.20
ONI
Q (m3/s)
ln(Q)
Channel capacity (z)
rho −0.20
rho −0.086
rho 0.33
rho 0.54
A (m2)
ln(A)
Area (z)
rho −0.17
rho 0.26
rho 0.48
rho 0.31
V (m/s)
ln(V)
Flow velocity (z)
rho 0.31
rho 0.54
rho 0.36
rho 0.37
W (m)
ln(W)
Width (z)
rho 0.10
rho −0.029
rho 0.20
rho 0.71
BE (m)
G (m)
Bed elevation (z)
rho 0.22
rho 0.20
rho −0.69
rho −0.14
Streamflow (z)
ONI

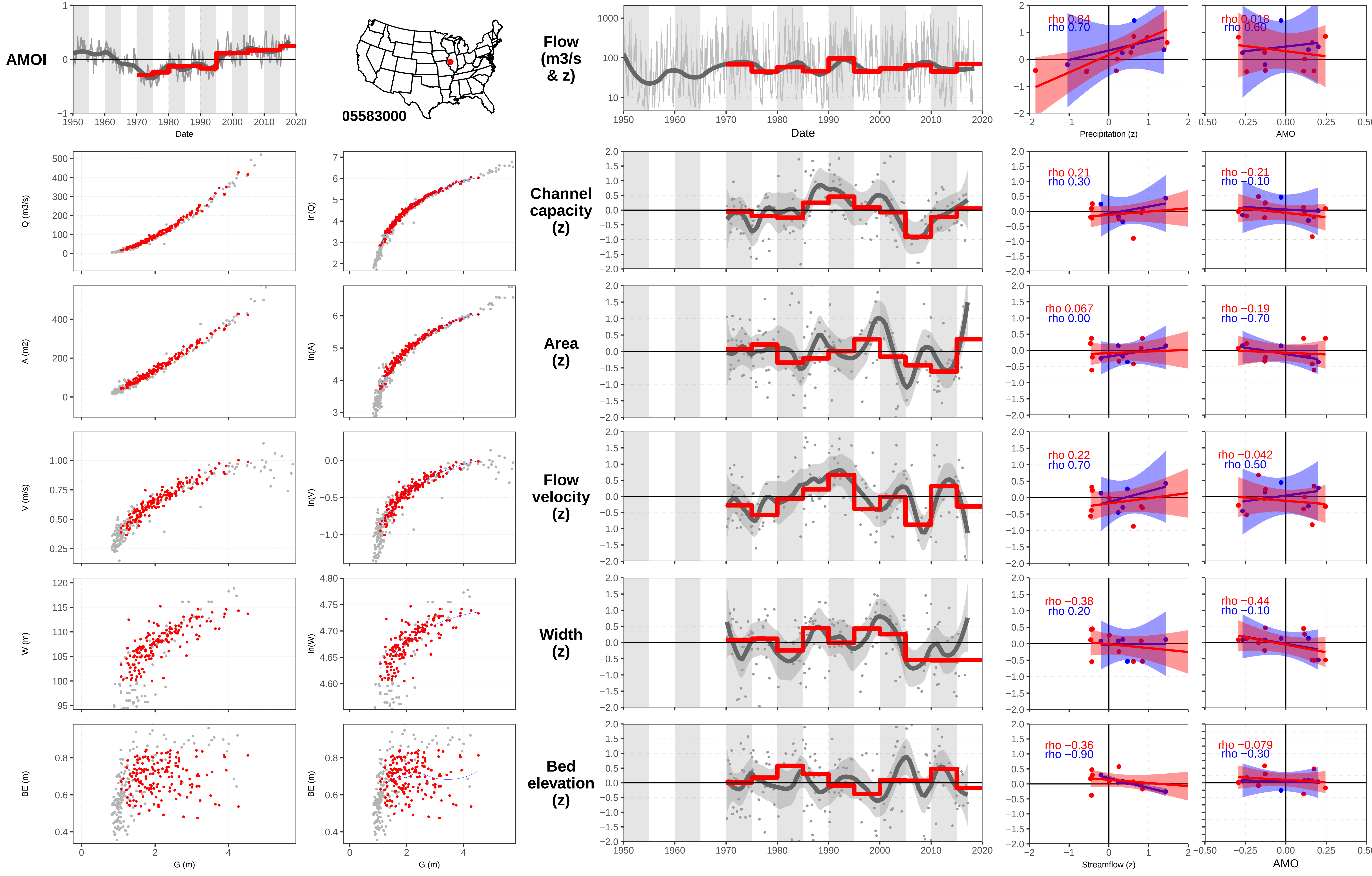

AMOI
Date
05583000
Flow (m3/s & z)
Date
Precipitation (z)
AMO
rho 0.84
rho 0.70
rho 0.018
rho 0.60
Channel capacity (z)
rho 0.21
rho 0.30
rho −0.21
rho −0.10
Q (m3/s)
ln(Q)
Area (z)
rho 0.067
rho 0.00
rho −0.19
rho −0.70
A (m2)
ln(A)
Flow velocity (z)
rho 0.22
rho 0.70
rho −0.042
rho 0.50
V (m/s)
ln(V)
Width (z)
rho −0.38
rho 0.20
rho −0.44
rho −0.10
W (m)
ln(W)
Bed elevation (z)
rho −0.36
rho −0.90
rho −0.079
rho −0.30
BE (m)
G (m)
Streamflow (z)

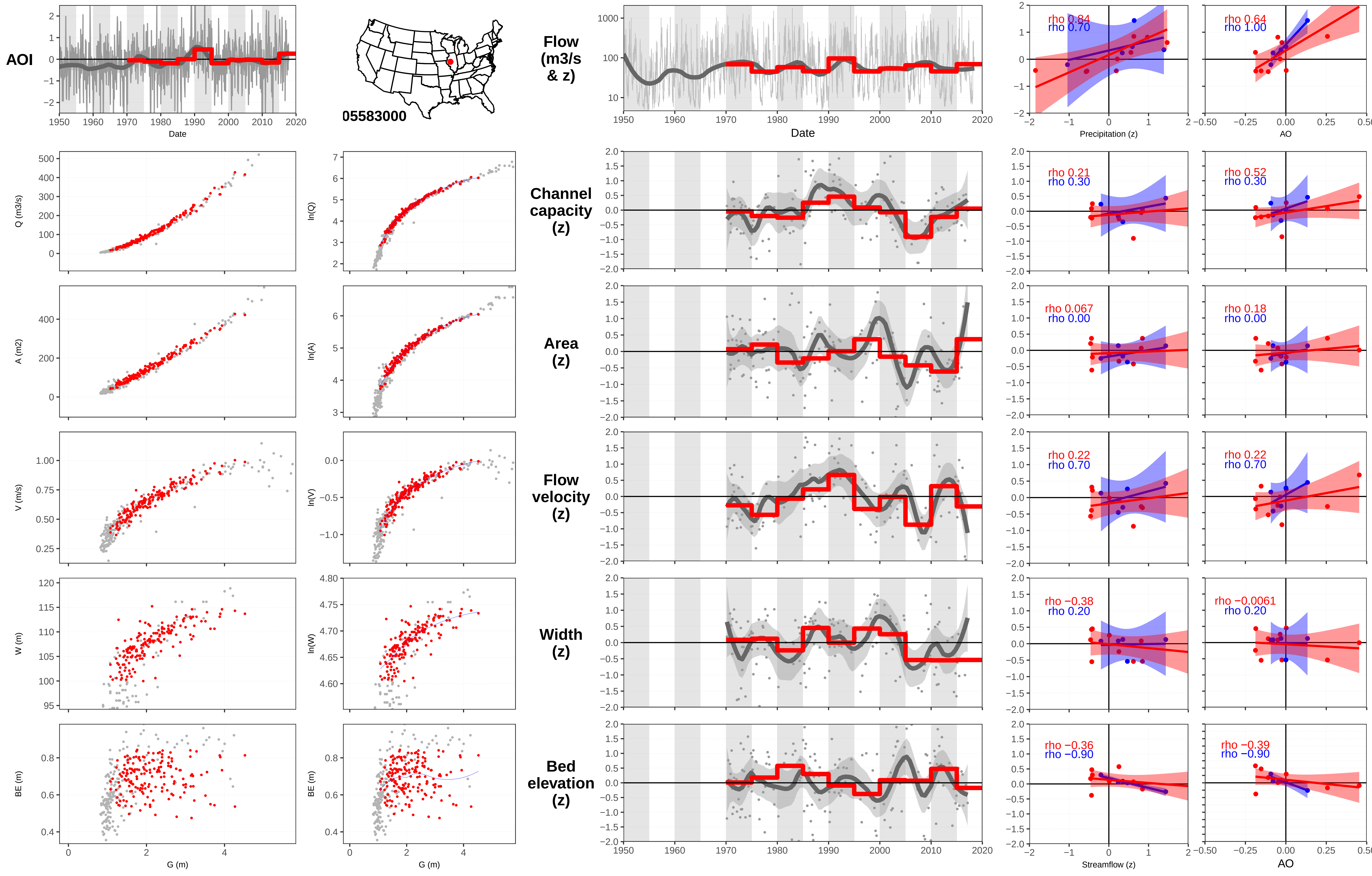

AOI
05583000
Flow (m3/s & z)
Channel capacity (z)
Area (z)
Flow velocity (z)
Width (z)
Bed elevation (z)
Date
Precipitation (z)
AO
Streamflow (z)
G (m)
Q (m3/s)
ln(Q)
A (m2)
ln(A)
V (m/s)
ln(V)
W (m)
ln(W)
BE (m)
rho 0.84
rho 0.70
rho 0.64
rho 1.00
rho 0.21
rho 0.30
rho 0.52
rho 0.30
rho 0.067
rho 0.00
rho 0.18
rho 0.00
rho 0.22
rho 0.70
rho 0.22
rho 0.70
rho −0.38
rho 0.20
rho −0.0061
rho 0.20
rho −0.36
rho −0.90
rho −0.39
rho −0.90

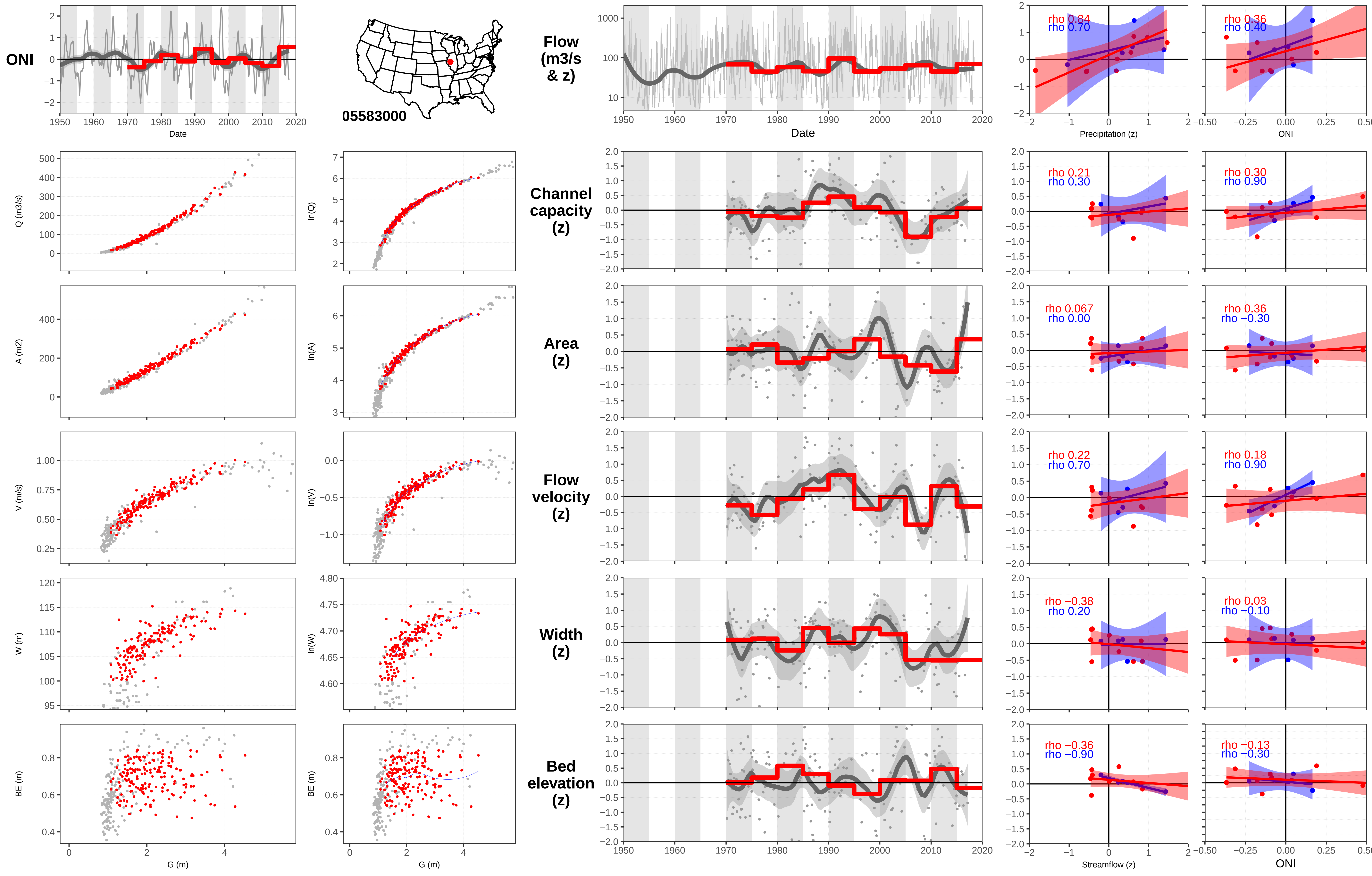

ONI
05583000
Flow (m3/s & z)
Channel capacity (z)
Area (z)
Flow velocity (z)
Width (z)
Bed elevation (z)
Date
Precipitation (z)
ONI
Streamflow (z)
G (m)
Q (m3/s)
ln(Q)
A (m2)
ln(A)
V (m/s)
ln(V)
W (m)
ln(W)
BE (m)
rho 0.84
rho 0.70
rho 0.36
rho 0.40
rho 0.21
rho 0.30
rho 0.30
rho 0.90
rho 0.067
rho 0.00
rho 0.36
rho −0.30
rho 0.22
rho 0.70
rho 0.18
rho 0.90
rho −0.38
rho 0.20
rho 0.03
rho −0.10
rho −0.36
rho −0.90
rho −0.13
rho −0.30

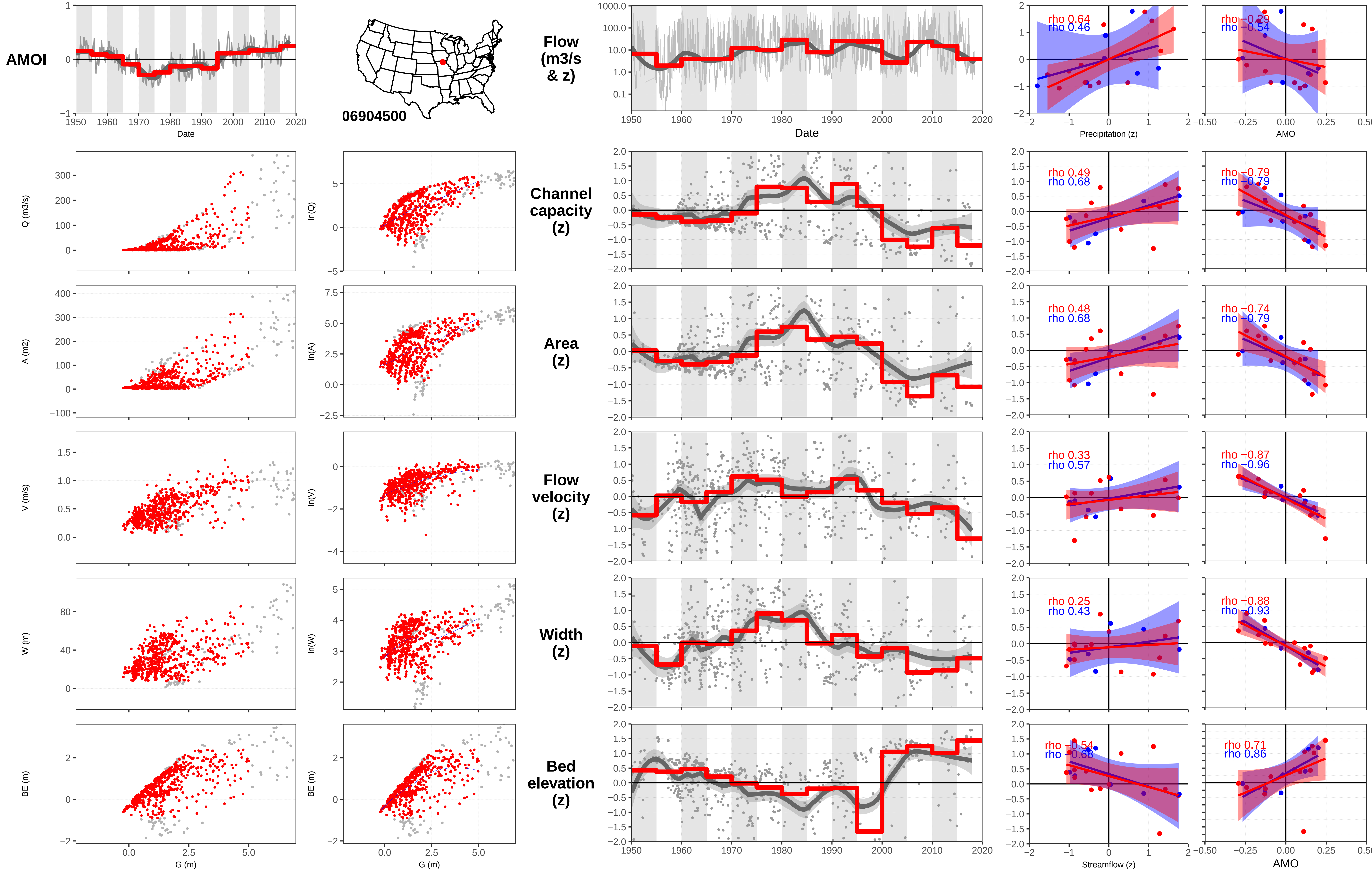

AMOI
06904500
Flow (m3/s & z)
Channel capacity (z)
Area (z)
Flow velocity (z)
Width (z)
Bed elevation (z)
Date
Precipitation (z)
Streamflow (z)
AMO
G (m)
Q (m3/s)
ln(Q)
A (m2)
ln(A)
V (m/s)
ln(V)
W (m)
ln(W)
BE (m)
rho 0.64
rho 0.46
rho −0.29
rho −0.54
rho 0.49
rho 0.68
rho −0.79
rho −0.79
rho 0.48
rho 0.68
rho −0.74
rho −0.79
rho 0.33
rho 0.57
rho −0.87
rho −0.96
rho 0.25
rho 0.43
rho −0.88
rho −0.93
rho −0.54
rho −0.68
rho 0.71
rho 0.86

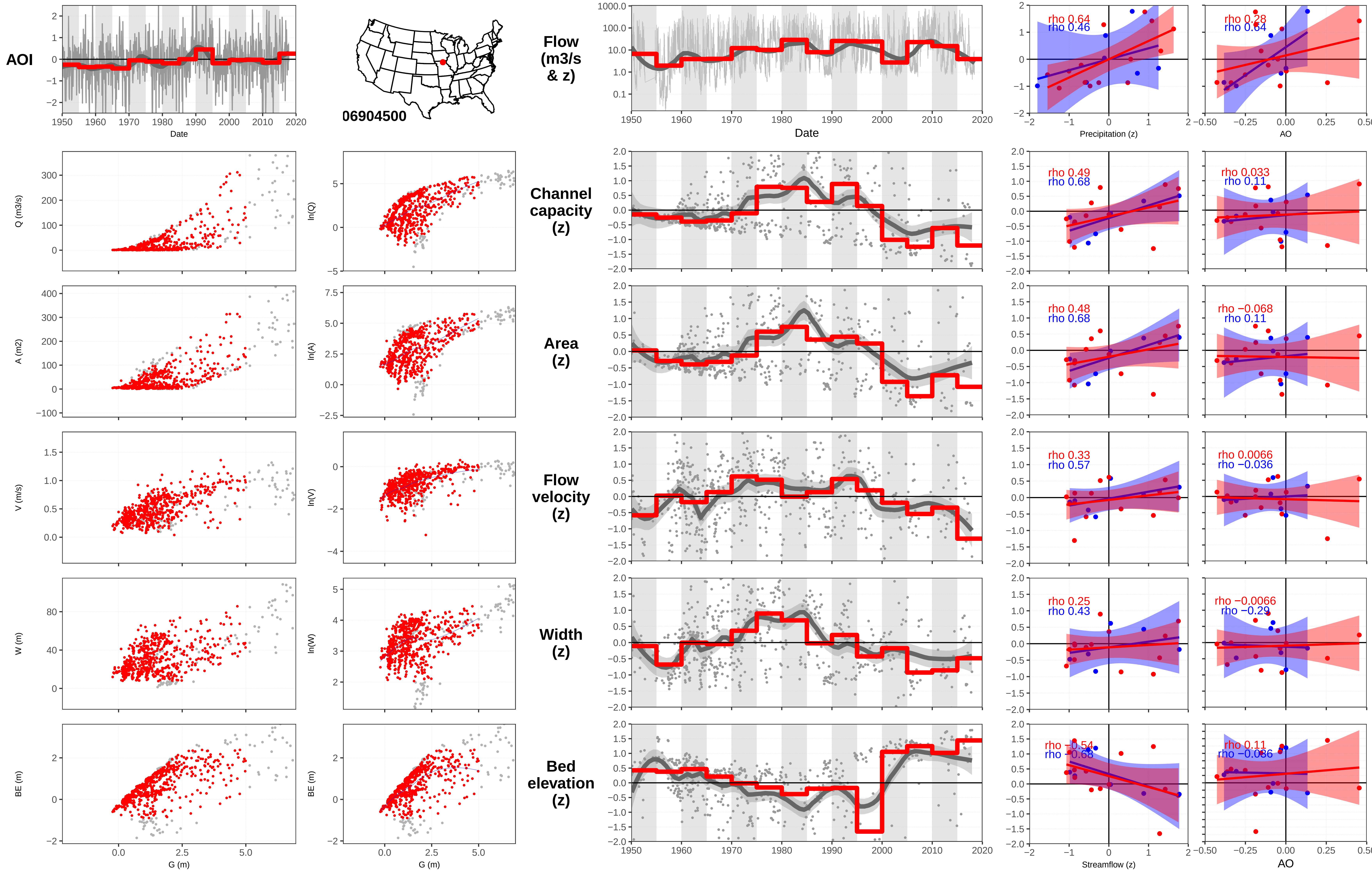

AOI
06904500
Flow (m3/s & z)
Channel capacity (z)
Area (z)
Flow velocity (z)
Width (z)
Bed elevation (z)
Date
Precipitation (z)
AO
Streamflow (z)
G (m)
Q (m3/s)
ln(Q)
A (m2)
ln(A)
V (m/s)
ln(V)
W (m)
ln(W)
BE (m)
rho 0.64
rho 0.46
rho 0.28
rho 0.64
rho 0.49
rho 0.68
rho 0.033
rho 0.11
rho 0.48
rho 0.68
rho −0.068
rho 0.11
rho 0.33
rho 0.57
rho 0.0066
rho −0.036
rho 0.25
rho 0.43
rho −0.0066
rho −0.29
rho −0.54
rho −0.68
rho 0.11
rho −0.086

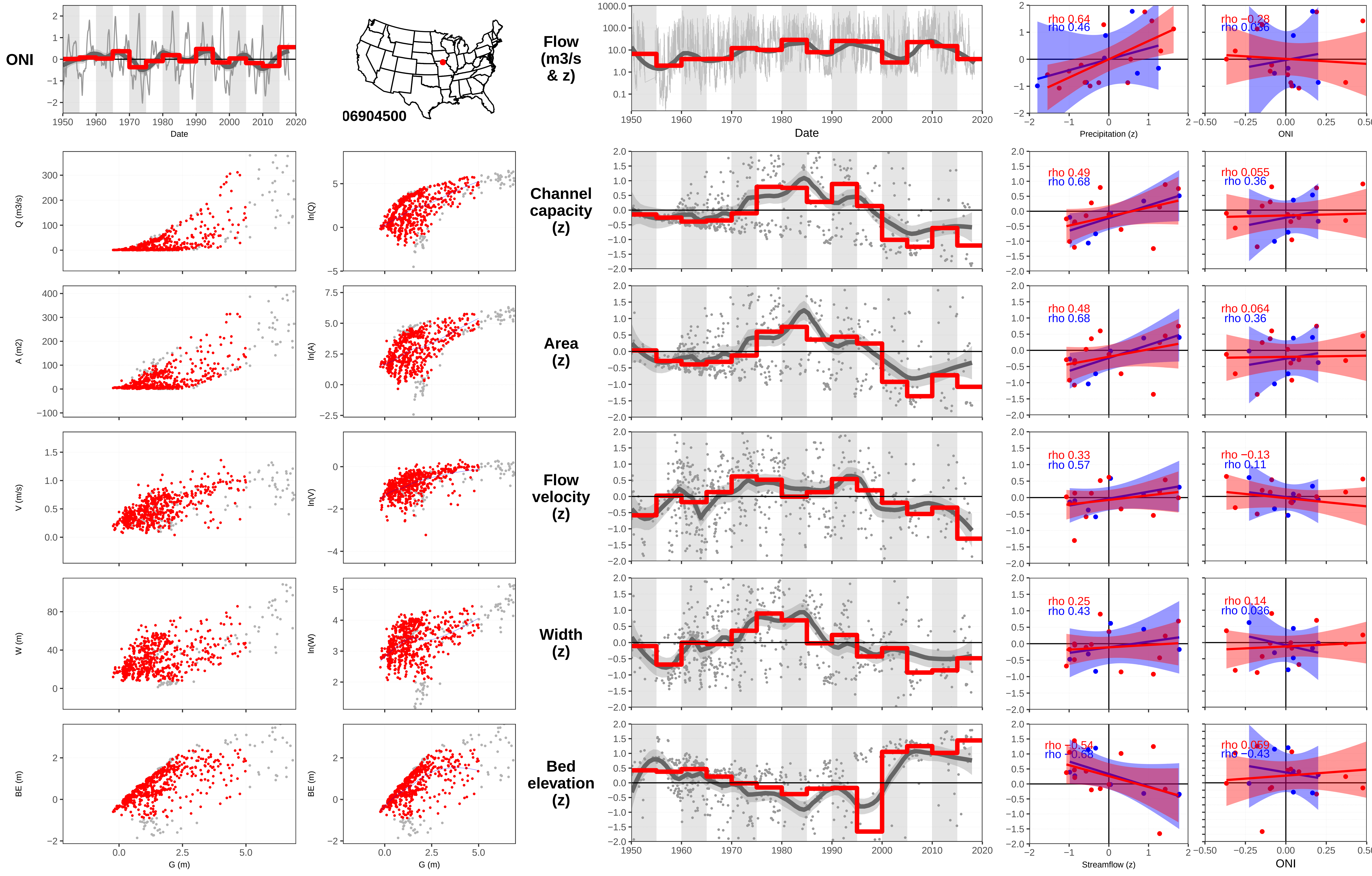

ONI
06904500
Flow (m3/s & z)
Channel capacity (z)
Area (z)
Flow velocity (z)
Width (z)
Bed elevation (z)
Date
Precipitation (z)
Streamflow (z)
G (m)
rho 0.64
rho 0.46
rho −0.28
rho 0.036
rho 0.49
rho 0.68
rho 0.055
rho 0.36
rho 0.48
rho 0.68
rho 0.064
rho 0.36
rho 0.33
rho 0.57
rho −0.13
rho 0.11
rho 0.25
rho 0.43
rho 0.14
rho 0.036
rho −0.54
rho −0.68
rho 0.059
rho −0.43

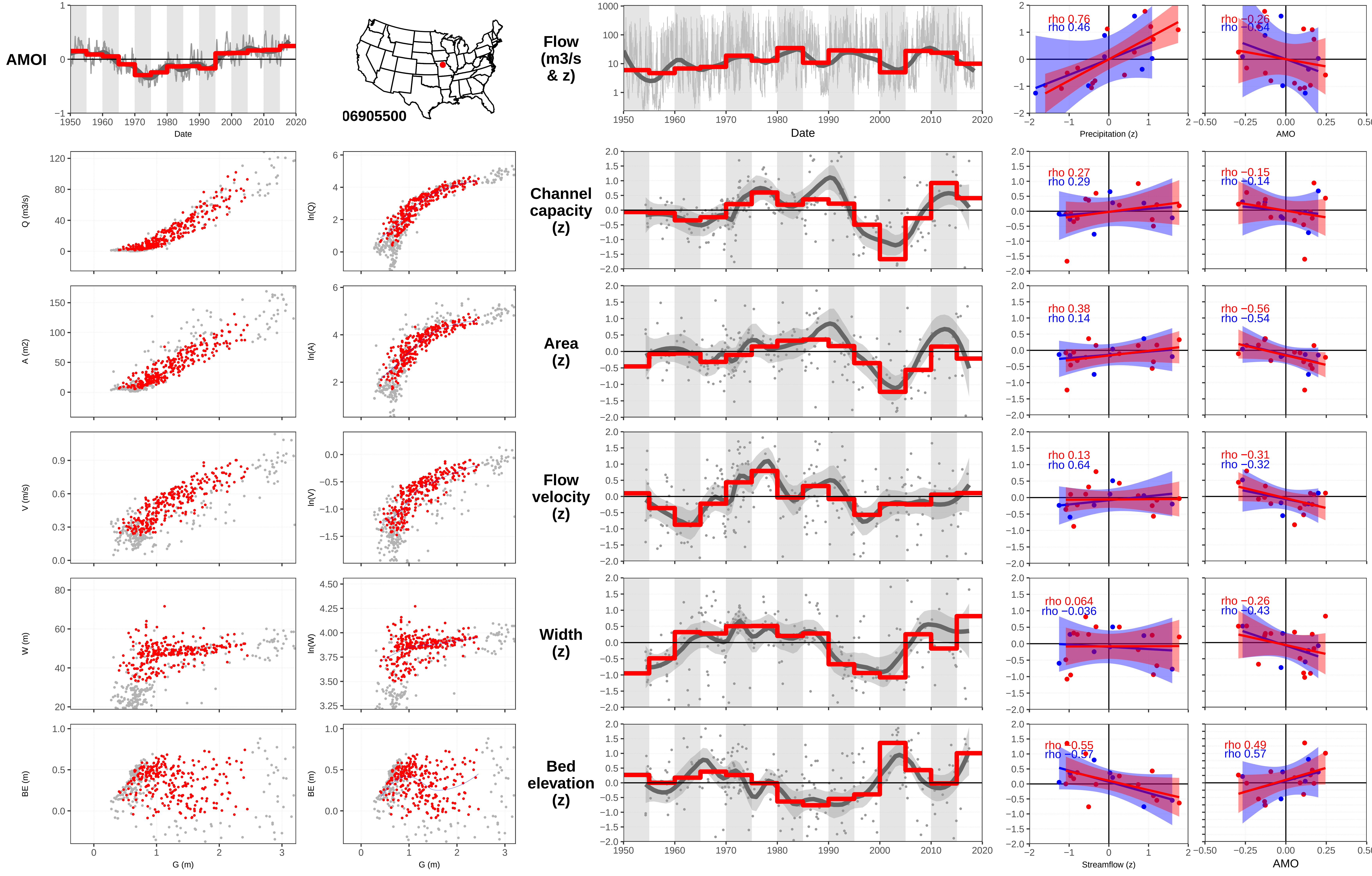

AMOI
06905500
Flow (m3/s & z)
Channel capacity (z)
Area (z)
Flow velocity (z)
Width (z)
Bed elevation (z)
Date
Precipitation (z)
AMO
Streamflow (z)
G (m)
Q (m3/s)
ln(Q)
A (m2)
ln(A)
V (m/s)
ln(V)
W (m)
ln(W)
BE (m)
rho 0.76
rho 0.46
rho −0.26
rho −0.54
rho 0.27
rho 0.29
rho −0.15
rho −0.14
rho 0.38
rho 0.14
rho −0.56
rho −0.54
rho 0.13
rho 0.64
rho −0.31
rho −0.32
rho 0.064
rho −0.036
rho −0.26
rho −0.43
rho −0.55
rho −0.57
rho 0.49
rho 0.57

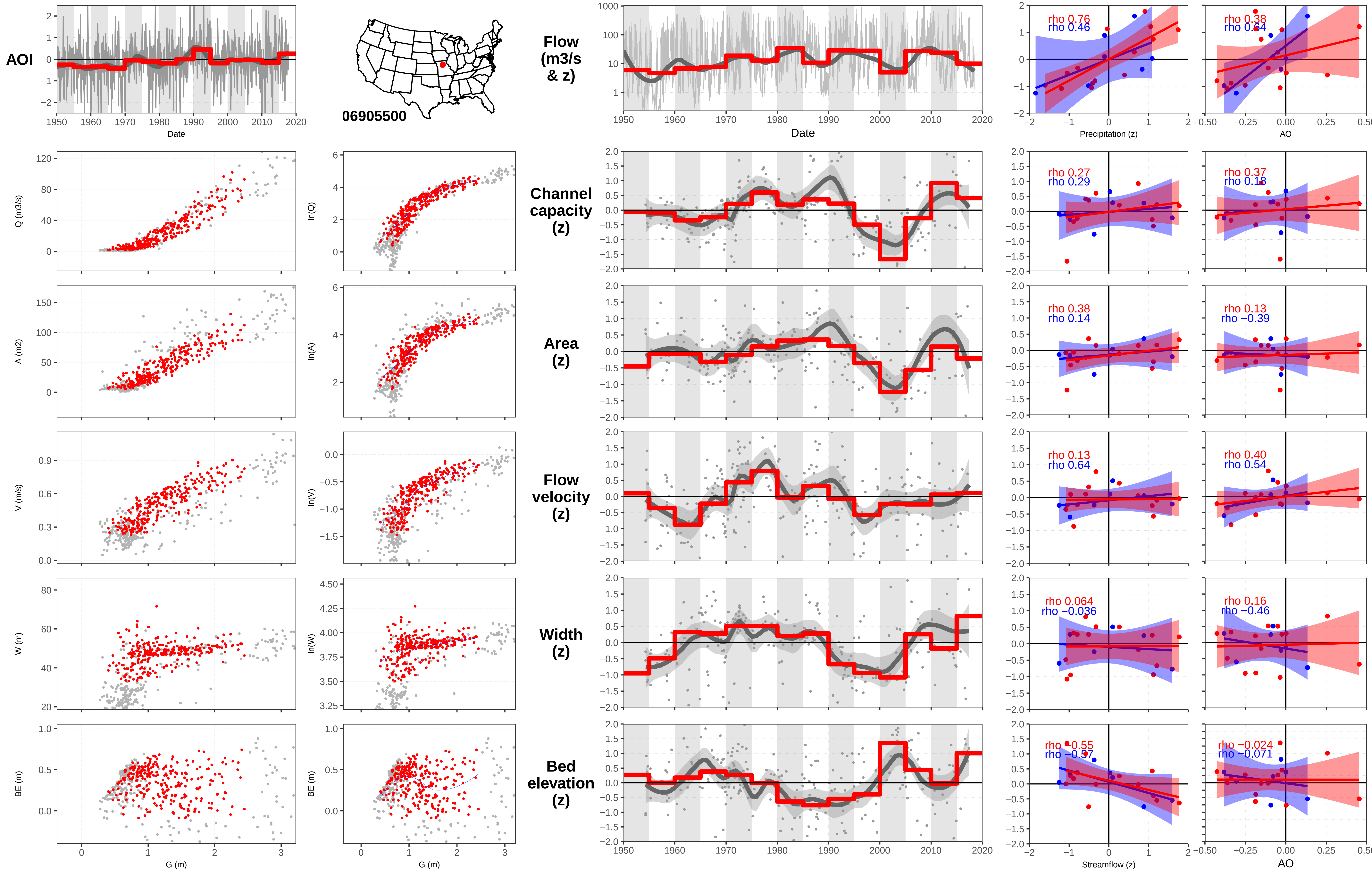

AOI
06905500
Flow (m3/s & z)
Channel capacity (z)
Area (z)
Flow velocity (z)
Width (z)
Bed elevation (z)
Date
Precipitation (z)
AO
Streamflow (z)
G (m)
Q (m3/s)
ln(Q)
A (m2)
ln(A)
V (m/s)
ln(V)
W (m)
ln(W)
BE (m)
rho 0.76
rho 0.46
rho 0.38
rho 0.64
rho 0.27
rho 0.29
rho 0.37
rho 0.18
rho 0.38
rho 0.14
rho 0.13
rho −0.39
rho 0.13
rho 0.64
rho 0.40
rho 0.54
rho 0.064
rho −0.036
rho 0.16
rho −0.46
rho −0.55
rho −0.57
rho −0.024
rho −0.071

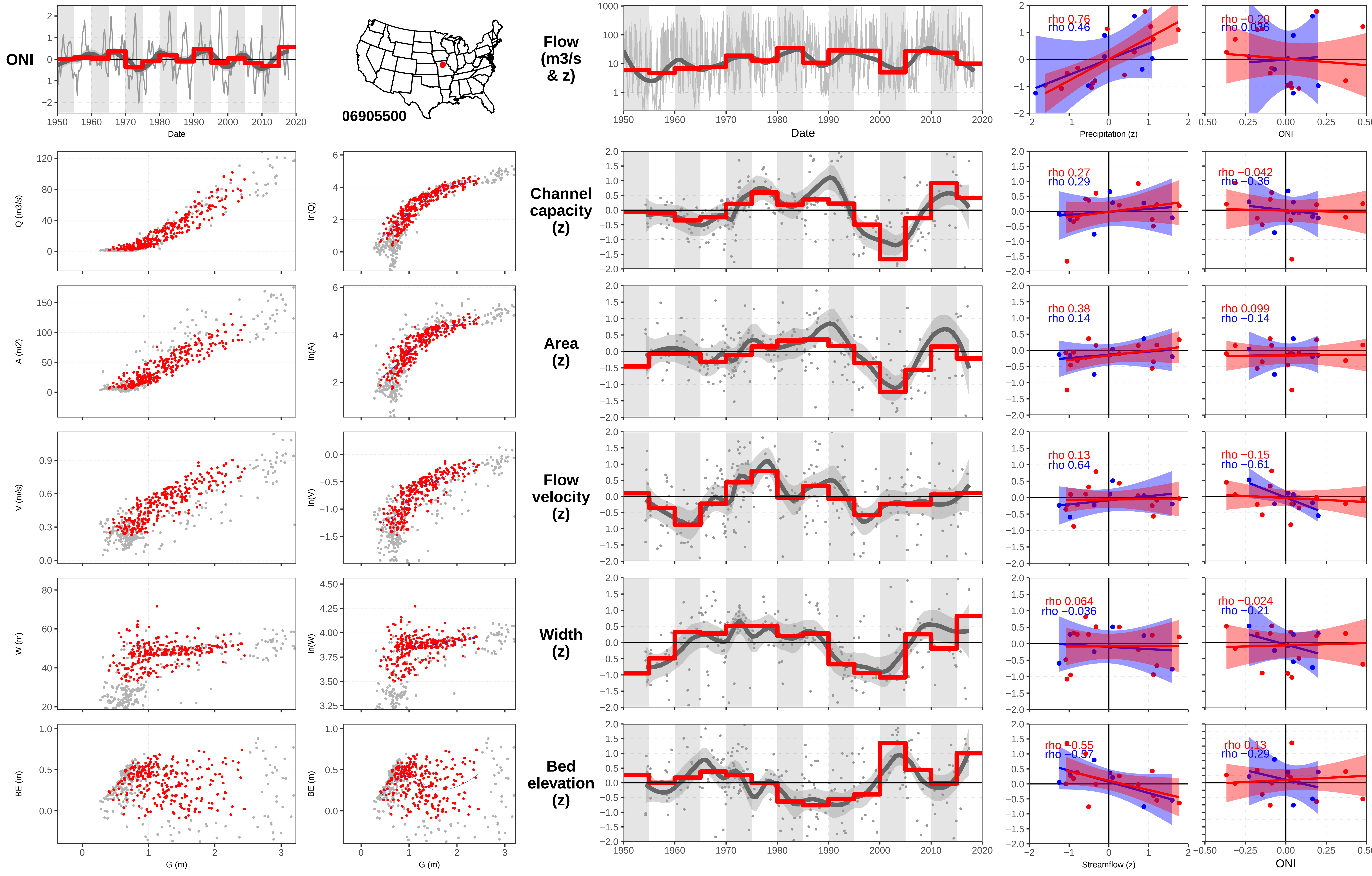

ONI
06905500
Flow (m3/s & z)
Channel capacity (z)
Area (z)
Flow velocity (z)
Width (z)
Bed elevation (z)
Date
Precipitation (z)
Streamflow (z)
ONI
G (m)
Q (m3/s)
ln(Q)
A (m2)
ln(A)
V (m/s)
ln(V)
W (m)
ln(W)
BE (m)
rho 0.76
rho 0.46
rho −0.20
rho 0.036
rho 0.27
rho 0.29
rho −0.042
rho −0.36
rho 0.38
rho 0.14
rho 0.099
rho −0.14
rho 0.13
rho 0.64
rho −0.15
rho −0.61
rho 0.064
rho −0.036
rho −0.024
rho −0.21
rho −0.55
rho −0.57
rho 0.13
rho −0.29

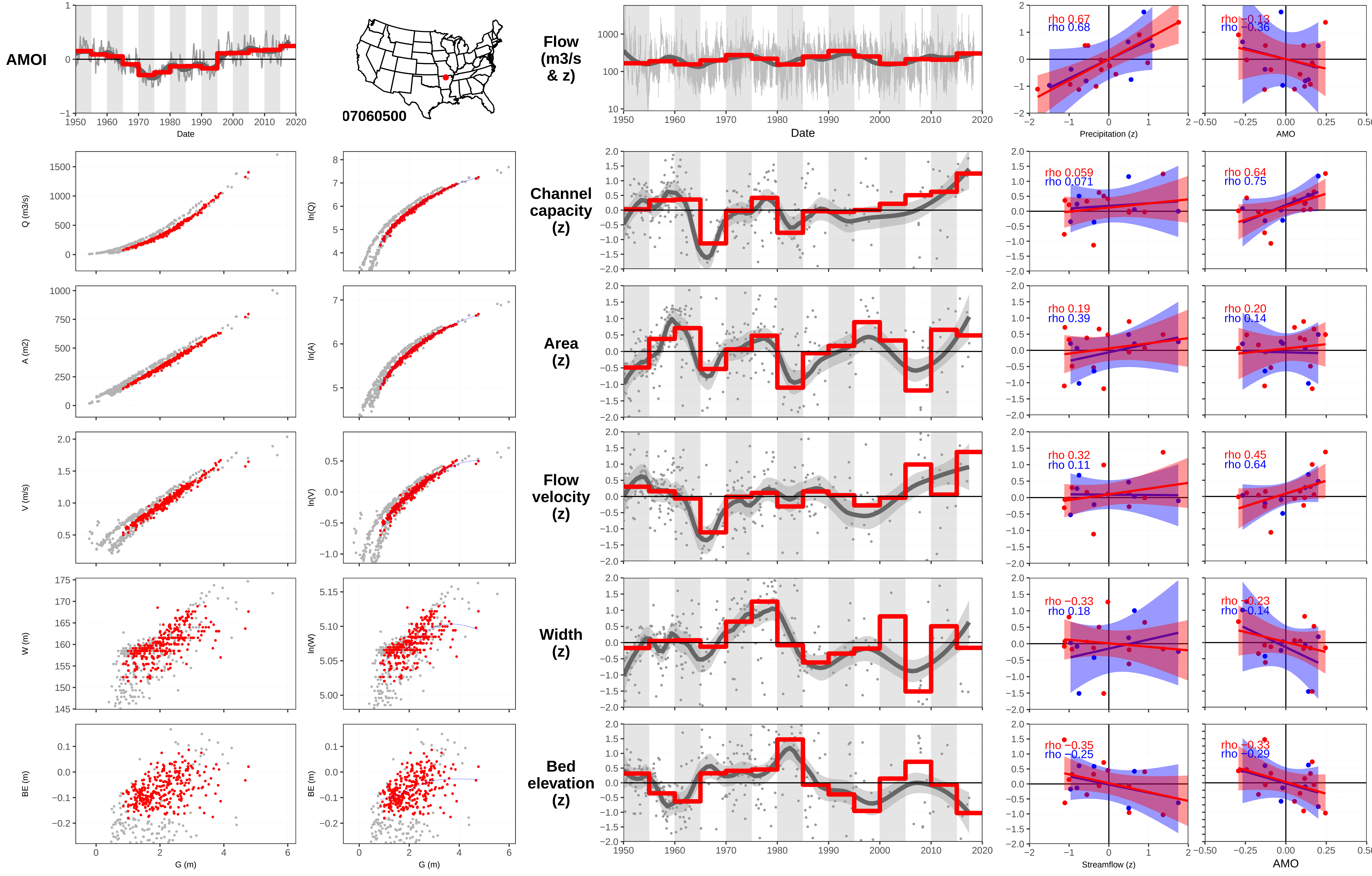

AMOI
07060500
Flow (m3/s & z)
Channel capacity (z)
Area (z)
Flow velocity (z)
Width (z)
Bed elevation (z)
Date
Precipitation (z)
AMO
Streamflow (z)
Q (m3/s)
ln(Q)
A (m2)
ln(A)
V (m/s)
ln(V)
W (m)
ln(W)
BE (m)
G (m)
rho 0.67
rho 0.68
rho −0.13
rho −0.36
rho 0.059
rho 0.071
rho 0.64
rho 0.75
rho 0.19
rho 0.39
rho 0.20
rho 0.14
rho 0.32
rho 0.11
rho 0.45
rho 0.64
rho −0.33
rho 0.18
rho −0.23
rho −0.14
rho −0.35
rho −0.25
rho −0.33
rho −0.29

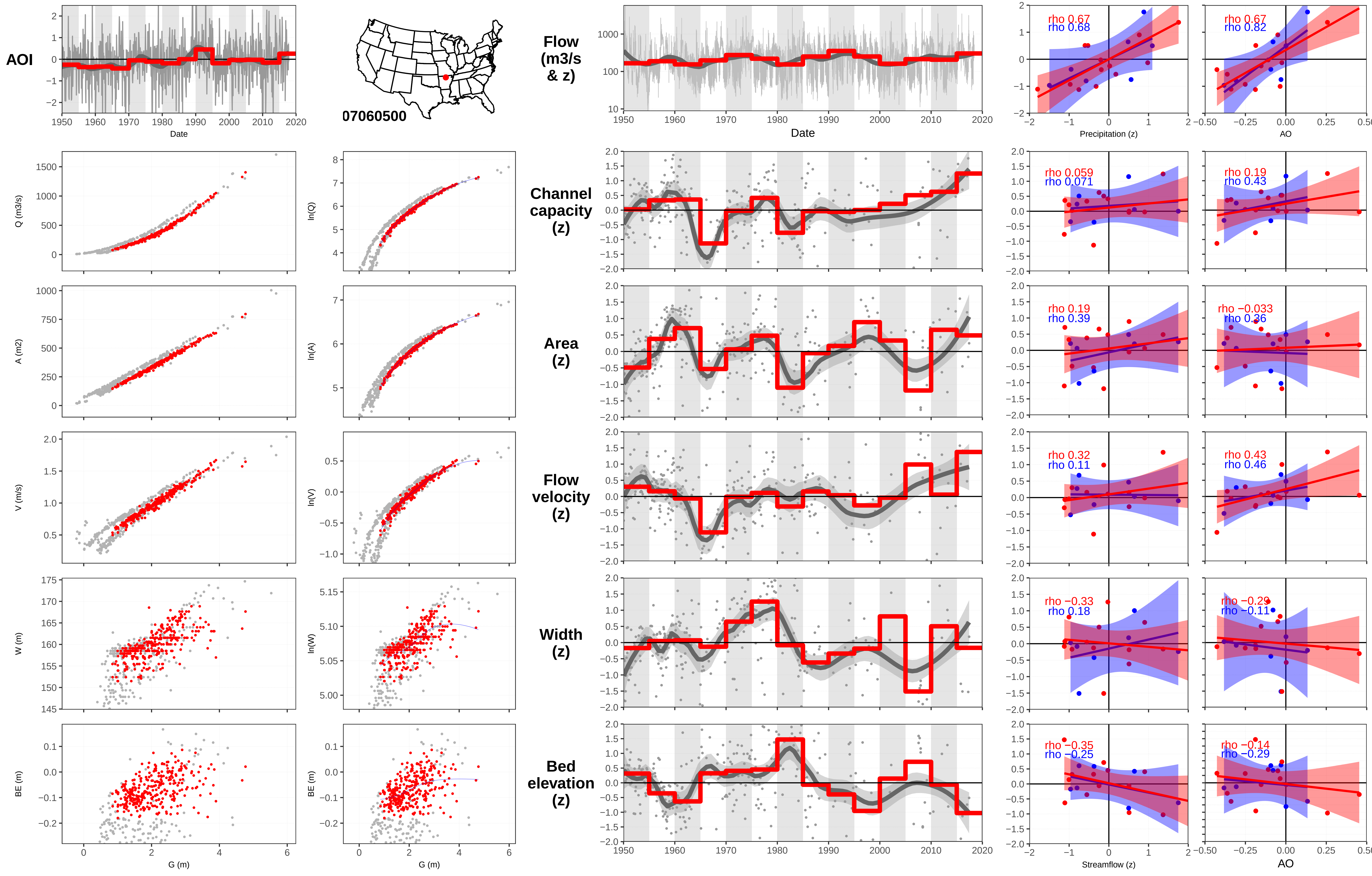

AOI
07060500
Flow (m3/s & z)
Channel capacity (z)
Area (z)
Flow velocity (z)
Width (z)
Bed elevation (z)
Date
Precipitation (z)
Streamflow (z)
AO
G (m)
Q (m3/s)
ln(Q)
A (m2)
ln(A)
V (m/s)
ln(V)
W (m)
ln(W)
BE (m)
rho 0.67
rho 0.68
rho 0.67
rho 0.82
rho 0.059
rho 0.071
rho 0.19
rho 0.43
rho 0.19
rho 0.39
rho −0.033
rho 0.36
rho 0.32
rho 0.11
rho 0.43
rho 0.46
rho −0.33
rho 0.18
rho −0.29
rho −0.11
rho −0.35
rho −0.25
rho −0.14
rho −0.29

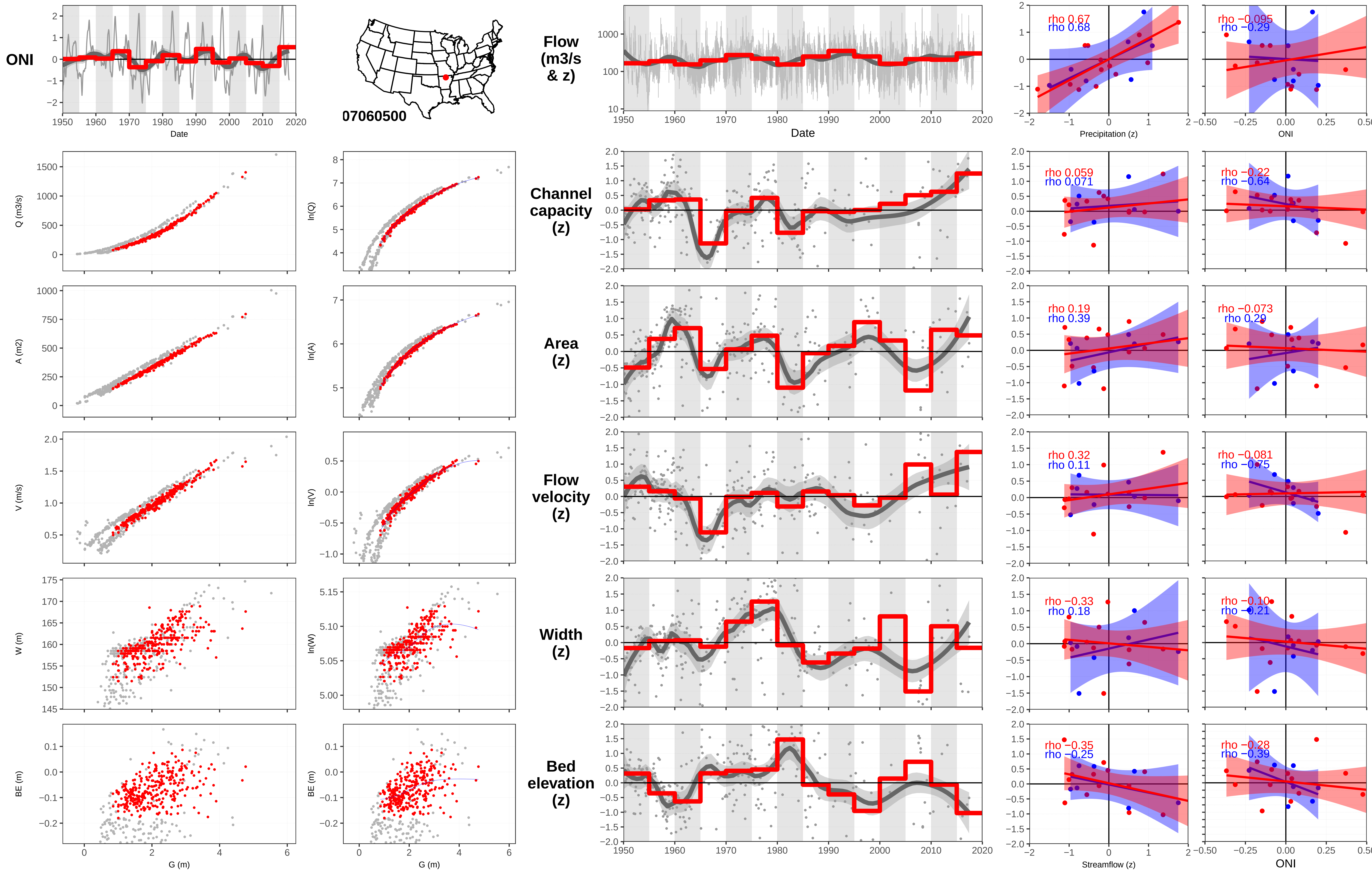

ONI
07060500
Flow (m3/s & z)
Channel capacity (z)
Area (z)
Flow velocity (z)
Width (z)
Bed elevation (z)
Date
Precipitation (z)
Streamflow (z)
ONI
Q (m3/s)
ln(Q)
A (m2)
ln(A)
V (m/s)
ln(V)
W (m)
ln(W)
BE (m)
G (m)
rho 0.67
rho 0.68
rho −0.095
rho −0.29
rho 0.059
rho 0.071
rho −0.22
rho −0.64
rho 0.19
rho 0.39
rho −0.073
rho 0.29
rho 0.32
rho 0.11
rho −0.081
rho −0.75
rho −0.33
rho 0.18
rho −0.10
rho −0.21
rho −0.35
rho −0.25
rho −0.28
rho −0.39

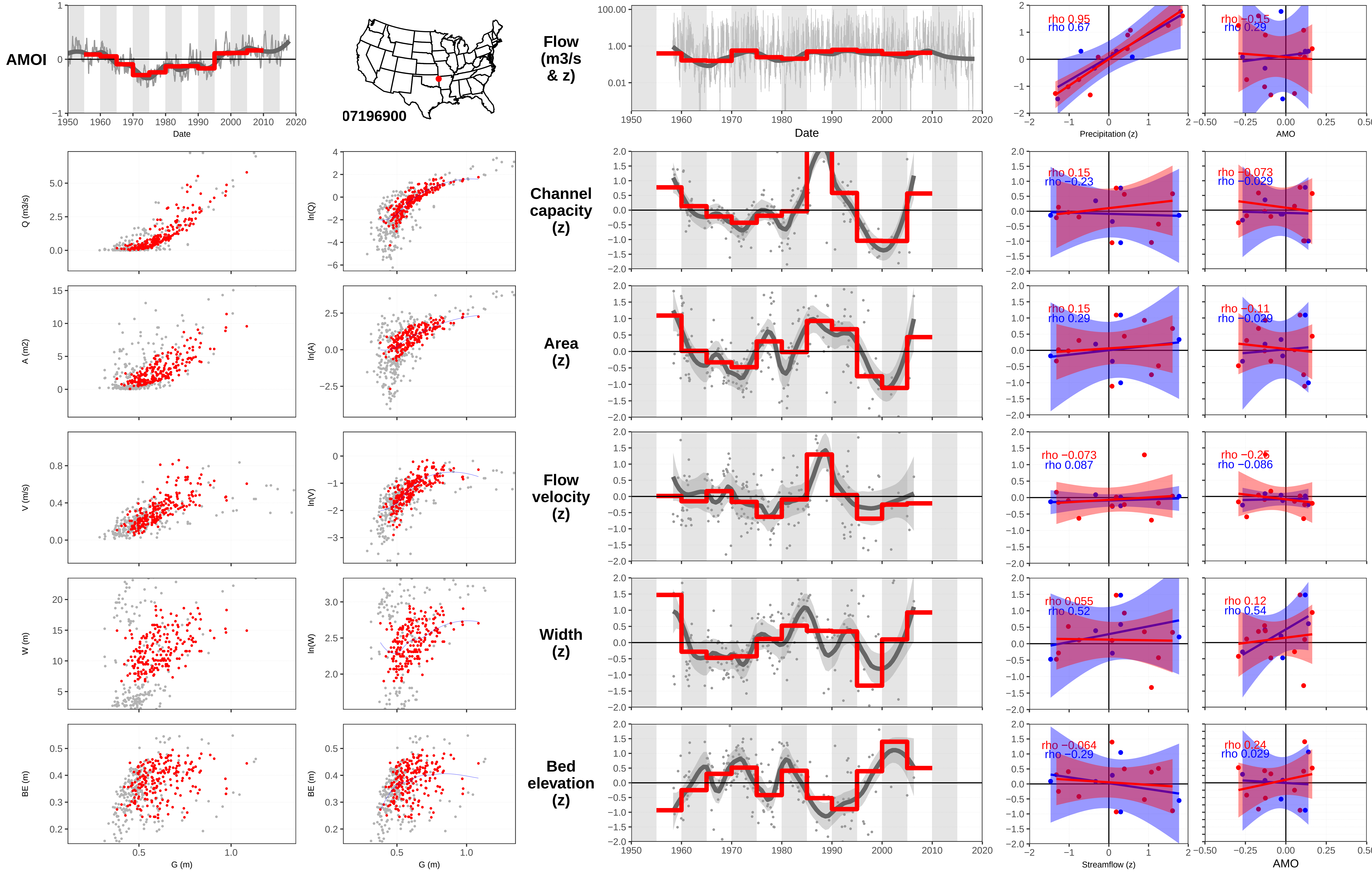

AMOI
Date
07196900
Flow (m3/s & z)
Date
Precipitation (z)
AMO
rho 0.95
rho 0.67
rho −0.15
rho 0.29
Q (m3/s)
ln(Q)
Channel capacity (z)
rho 0.15
rho −0.23
rho −0.073
rho −0.029
A (m2)
ln(A)
Area (z)
rho 0.15
rho 0.29
rho −0.11
rho −0.029
V (m/s)
ln(V)
Flow velocity (z)
rho −0.073
rho 0.087
rho −0.25
rho −0.086
W (m)
ln(W)
Width (z)
rho 0.055
rho 0.52
rho 0.12
rho 0.54
BE (m)
BE (m)
Bed elevation (z)
rho −0.064
rho −0.29
rho 0.24
rho 0.029
G (m)
G (m)
Streamflow (z)
AMO

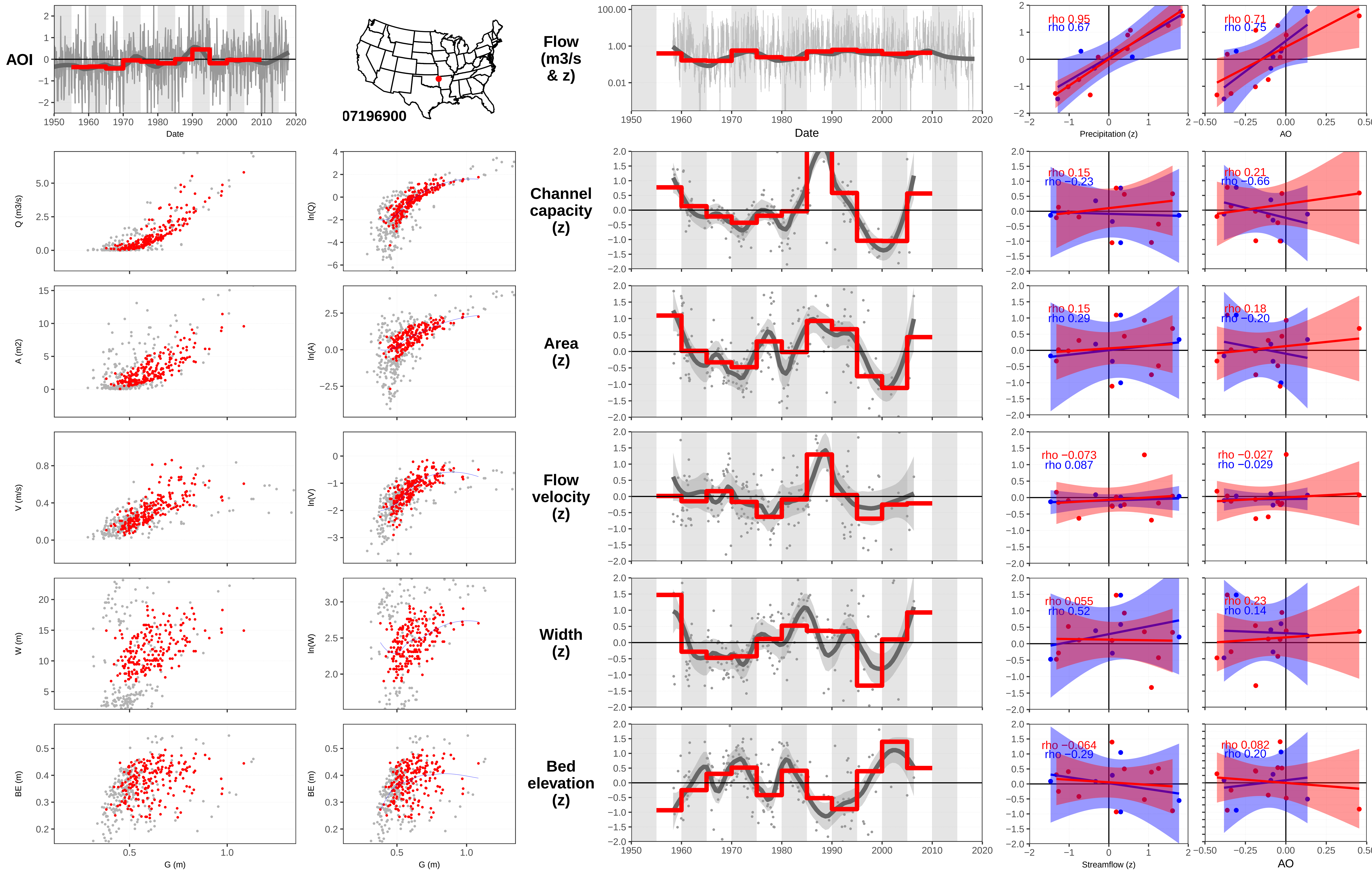

AOI
Date
07196900
Flow (m3/s & z)
Date
Precipitation (z)
AO
rho 0.95
rho 0.67
rho 0.71
rho 0.75
Q (m3/s)
ln(Q)
Channel capacity (z)
rho 0.15
rho −0.23
rho 0.21
rho −0.66
A (m2)
ln(A)
Area (z)
rho 0.15
rho 0.29
rho 0.18
rho −0.20
V (m/s)
ln(V)
Flow velocity (z)
rho −0.073
rho 0.087
rho −0.027
rho −0.029
W (m)
ln(W)
Width (z)
rho 0.055
rho 0.52
rho 0.23
rho 0.14
BE (m)
BE (m)
Bed elevation (z)
rho −0.064
rho −0.29
rho 0.082
rho 0.20
G (m)
G (m)
Streamflow (z)
AO

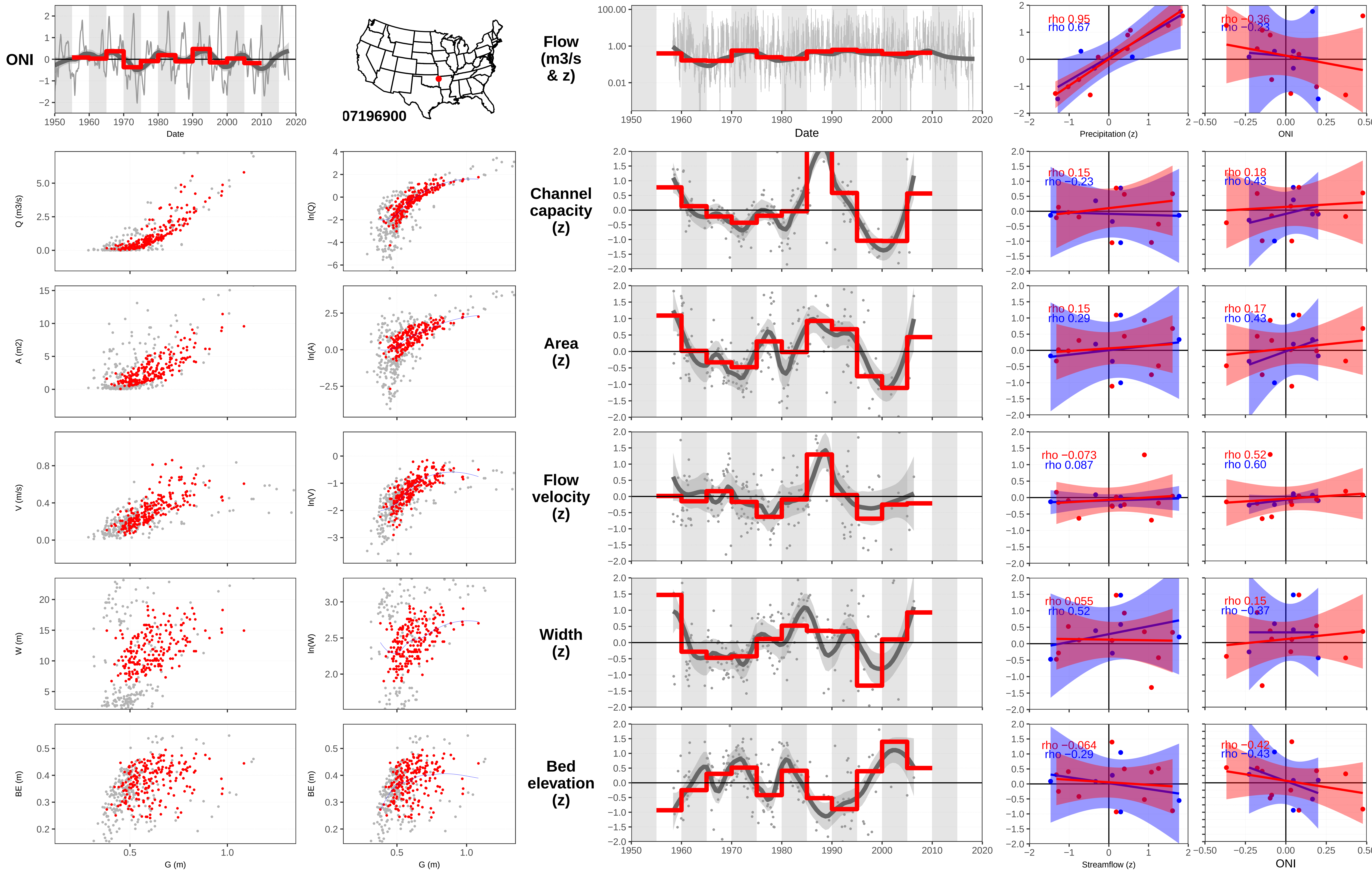
ONI
07196900
Flow (m3/s & z)
Channel capacity (z)
Area (z)
Flow velocity (z)
Width (z)
Bed elevation (z)
Date
Precipitation (z)
Streamflow (z)
ONI
Q (m3/s)
ln(Q)
A (m2)
ln(A)
V (m/s)
ln(V)
W (m)
ln(W)
BE (m)
G (m)
rho 0.95
rho 0.67
rho −0.36
rho −0.23
rho 0.15
rho −0.23
rho 0.18
rho 0.43
rho 0.15
rho 0.29
rho 0.17
rho 0.43
rho −0.073
rho 0.087
rho 0.52
rho 0.60
rho 0.055
rho 0.52
rho 0.15
rho −0.37
rho −0.064
rho −0.29
rho −0.42
rho −0.43

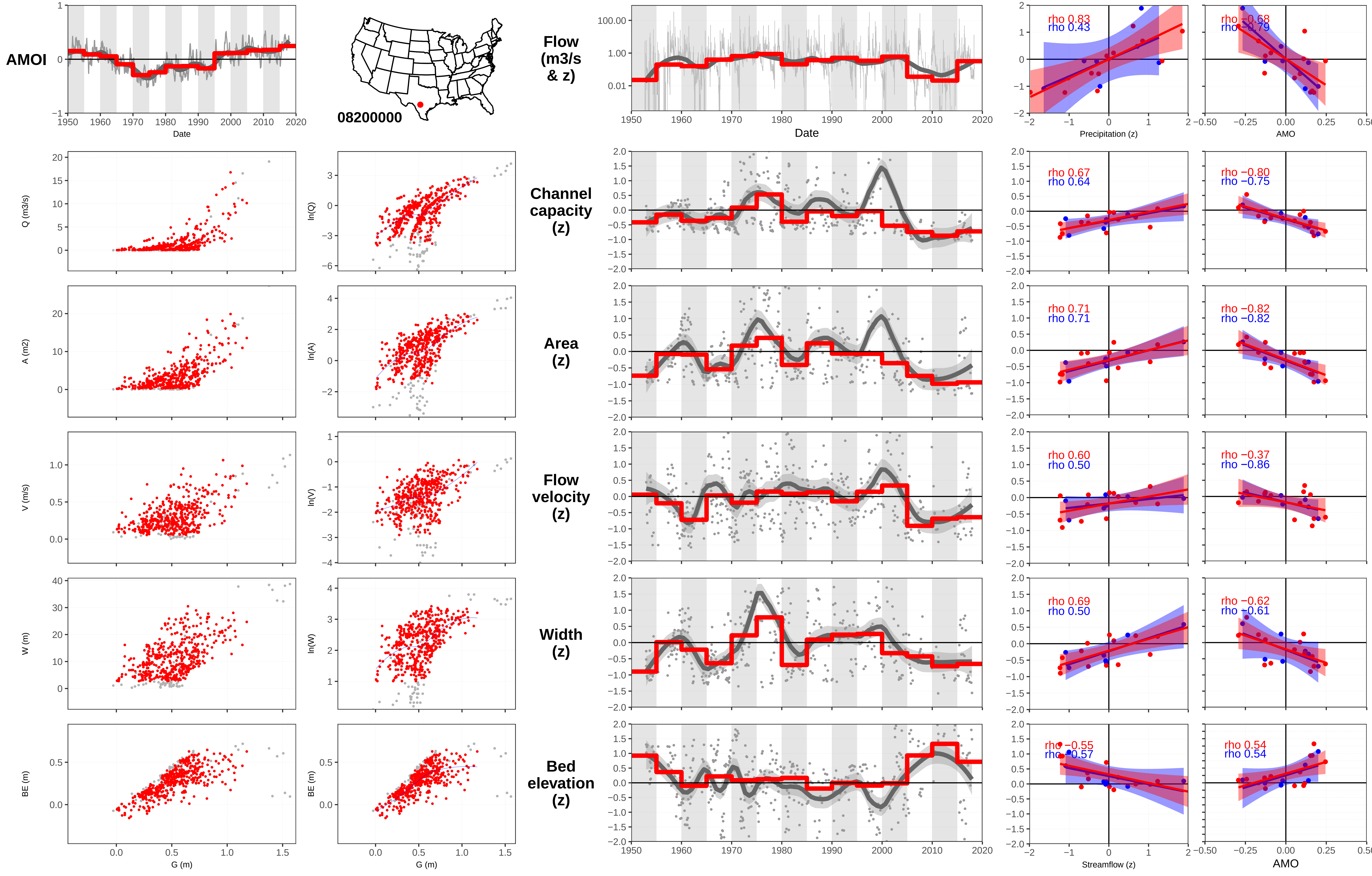

AMOI
08200000
Flow (m3/s & z)
Channel capacity (z)
Area (z)
Flow velocity (z)
Width (z)
Bed elevation (z)
Date
Precipitation (z)
AMO
Streamflow (z)
G (m)
Q (m3/s)
ln(Q)
A (m2)
ln(A)
V (m/s)
ln(V)
W (m)
ln(W)
BE (m)
rho 0.83
rho 0.43
rho −0.68
rho −0.79
rho 0.67
rho 0.64
rho −0.80
rho −0.75
rho 0.71
rho 0.71
rho −0.82
rho −0.82
rho 0.60
rho 0.50
rho −0.37
rho −0.86
rho 0.69
rho 0.50
rho −0.62
rho −0.61
rho −0.55
rho −0.57
rho 0.54
rho 0.54

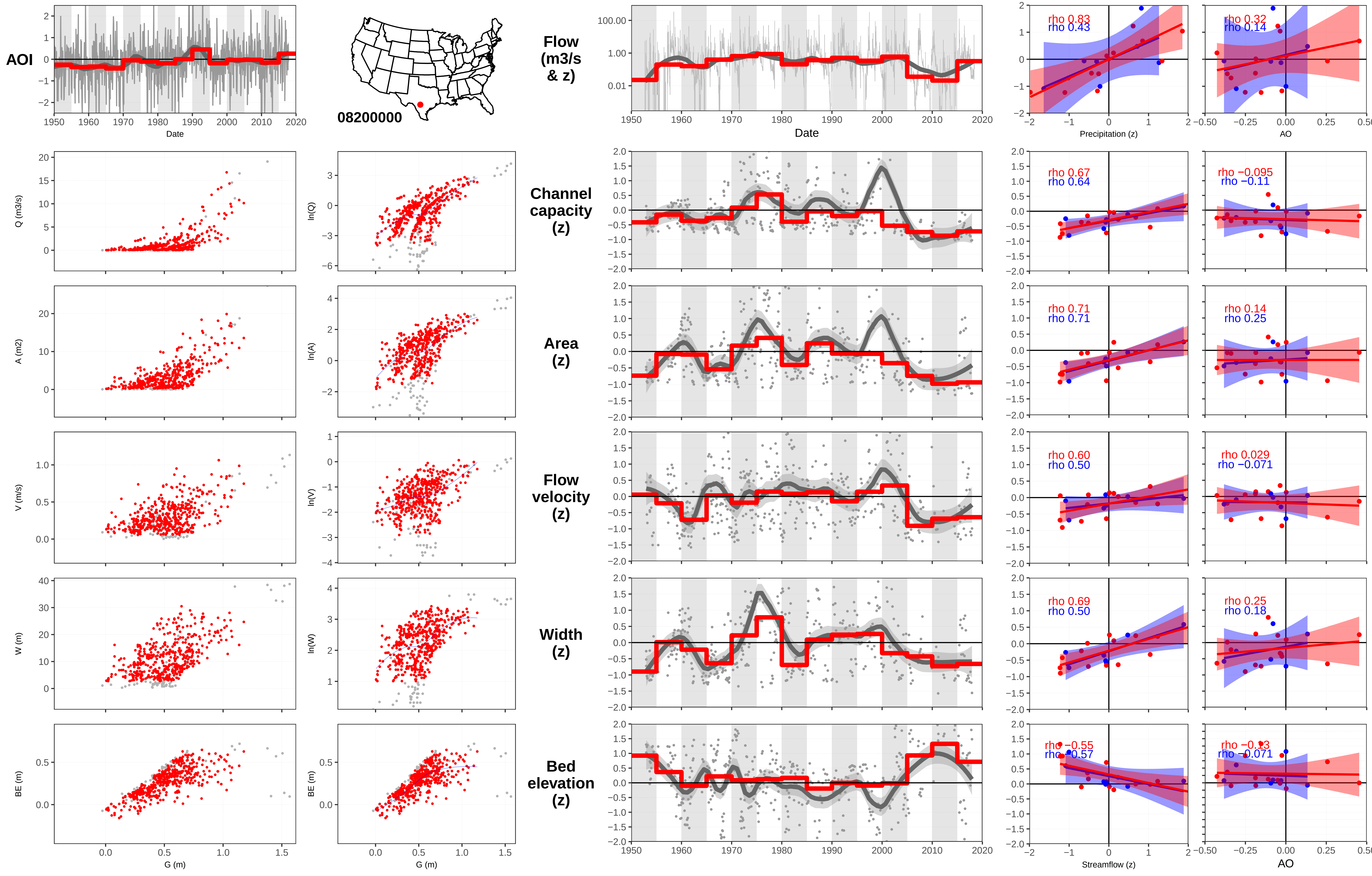

AOI
08200000
Flow (m3/s & z)
Channel capacity (z)
Area (z)
Flow velocity (z)
Width (z)
Bed elevation (z)
Date
Precipitation (z)
AO
Streamflow (z)
G (m)
Q (m3/s)
ln(Q)
A (m2)
ln(A)
V (m/s)
ln(V)
W (m)
ln(W)
BE (m)
rho 0.83
rho 0.43
rho 0.32
rho 0.14
rho 0.67
rho 0.64
rho −0.095
rho −0.11
rho 0.71
rho 0.71
rho 0.14
rho 0.25
rho 0.60
rho 0.50
rho 0.029
rho −0.071
rho 0.69
rho 0.50
rho 0.25
rho 0.18
rho −0.55
rho −0.57
rho −0.13
rho −0.071

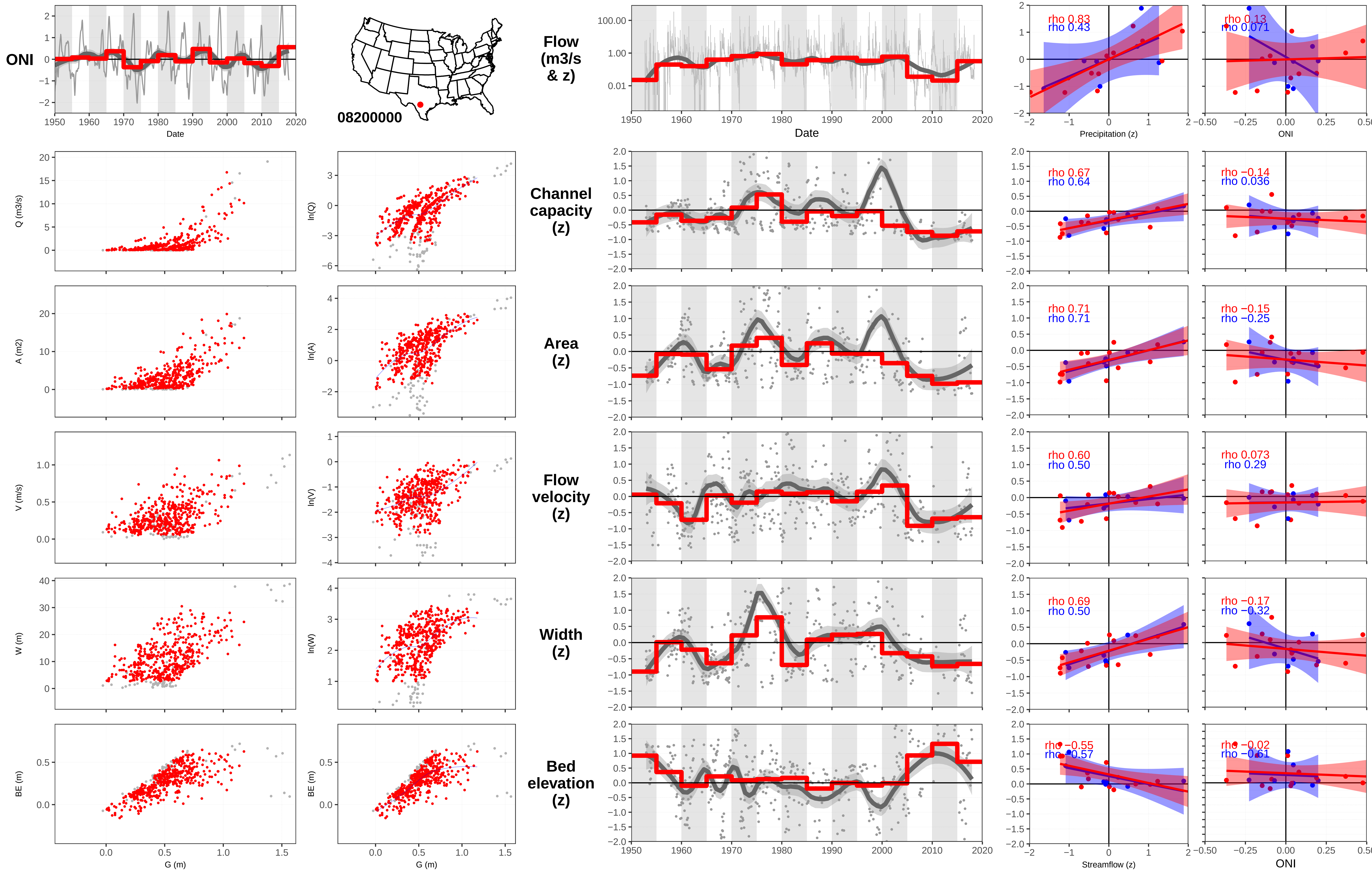

ONI
08200000
Flow (m3/s & z)
Channel capacity (z)
Area (z)
Flow velocity (z)
Width (z)
Bed elevation (z)
Date
Precipitation (z)
Streamflow (z)
ONI
G (m)
Q (m3/s)
ln(Q)
A (m2)
ln(A)
V (m/s)
ln(V)
W (m)
ln(W)
BE (m)
rho 0.83
rho 0.43
rho 0.13
rho 0.071
rho 0.67
rho 0.64
rho −0.14
rho 0.036
rho 0.71
rho 0.71
rho −0.15
rho −0.25
rho 0.60
rho 0.50
rho 0.073
rho 0.29
rho 0.69
rho 0.50
rho −0.17
rho −0.32
rho −0.55
rho −0.57
rho −0.02
rho −0.61

Summary statistics for every site

VARIABLES

Site: USGS site identification number
Index: The climate mode index defined in the manuscript
DA(km2): Drainage area in square kilometres
Elev(m): The site elevation in metres
The following metrics indicate the Spearman's rank correlation coefficient (r) between the 5-year median values of each of the variables:
r.CC.Q50: Spearman's rank correlation coefficient between the channel conveyance capacity and streamflow
r.A.Q50: Spearman's rank correlation coefficient between the cross-sectional flow area and streamflow
r.V.Q50: Spearman's rank correlation coefficient between the cross-sectional mean flow velocity and streamflow
r.W.Q50: Spearman's rank correlation coefficient between the channel width and streamflow
r.B.Q50: Spearman's rank correlation coefficient between the channel mean bed elevation and streamflow
r.Q50.P: Spearman's rank correlation coefficient between streamflow and precipitation
r.Q50.Ind: Spearman's rank correlation coefficient between streamflow and the climate index
r.CC.Ind: Spearman's rank correlation coefficient between channel conveyance capacity and the climate index
r.A.Ind: Spearman's rank correlation coefficient between cross-sectional flow area and the climate index
r.V.Ind: Spearman's rank correlation coefficient between cross-sectional mean flow velocity and the climate index
r.W.Ind: Spearman's rank correlation coefficient between channel width and the climate index
r.B.Ind: Spearman's rank correlation coefficient between channel mean bed elevation and the climate index

Site	Index	DA(km2)	Elev(m)	r.CC.Q50	r.A.Q50	r.V.Q50	r.W.Q50	r.B.Q50	r.Q50.P	r.Q50.Ind	r.CC.Ind	r.A.Ind	r.V.Ind	r.W.Ind	r.B.Ind
01452500	AMOI	115.25	247.24	0.39	0.42	0.30	0.43	-0.10	0.81	-0.26	0.46	0.01	0.43	-0.05	0.21
01452500	AOI	115.25	247.24	0.39	0.42	0.30	0.43	-0.10	0.81	0.24	-0.01	-0.04	0.05	-0.50	-0.18
01452500	ONI	115.25	247.24	0.39	0.42	0.30	0.43	-0.10	0.81	-0.71	-0.40	-0.55	-0.19	-0.55	0.23
01469500	AMOI	111.11	817.48	0.18	0.44	-0.23	0.15	-0.63	0.90	-0.17	-0.22	0.08	0.07	-0.15	-0.04
01469500	AOI	111.11	817.48	0.18	0.44	-0.23	0.15	-0.63	0.90	0.25	0.43	-0.33	0.60	-0.33	-0.03
01469500	ONI	111.11	817.48	0.18	0.44	-0.23	0.15	-0.63	0.90	-0.30	-0.02	-0.03	0.35	-0.28	0.13
01480617	AMOI	142.45	262.52	0.52	0.65	-0.01	0.21	-0.59	0.81	-0.26	0.19	-0.04	0.02	-0.07	0.02
01480617	AOI	142.45	262.52	0.52	0.65	-0.01	0.21	-0.59	0.81	-0.53	-0.09	-0.15	-0.43	0.21	0.13
01480617	ONI	142.45	262.52	0.52	0.65	-0.01	0.21	-0.59	0.81	-0.66	-0.38	-0.55	0.04	-0.16	0.47
01541000	AMOI	815.85	1207.14	-0.04	0.30	-0.26	0.16	-0.20	0.92	-0.24	-0.20	0.17	-0.30	-0.19	-0.28
01541000	AOI	815.85	1207.14	-0.04	0.30	-0.26	0.16	-0.20	0.92	0.44	-0.11	0.62	-0.77	-0.21	-0.41
01541000	ONI	815.85	1207.14	-0.04	0.30	-0.26	0.16	-0.20	0.92	0.05	-0.10	0.09	0.15	-0.38	-0.25
01544500	AMOI	352.24	1027.12	0.23	0.18	0.27	0.06	-0.26	0.72	0.09	-0.58	-0.48	-0.64	-0.45	0.35
01544500	AOI	352.24	1027.12	0.23	0.18	0.27	0.06	-0.26	0.72	0.75	0.31	0.22	0.31	-0.05	-0.32
01544500	ONI	352.24	1027.12	0.23	0.18	0.27	0.06	-0.26	0.72	0.07	-0.00	0.00	0.06	0.45	0.18
01580000	AMOI	244.50	249.60	0.22	0.10	0.11	0.13	-0.03	0.92	-0.03	-0.86	-0.80	0.06	0.48	0.88
01580000	AOI	244.50	249.60	0.22	0.10	0.11	0.13	-0.03	0.92	0.31	-0.06	-0.16	0.10	-0.40	-0.02
01580000	ONI	244.50	249.60	0.22	0.10	0.11	0.13	-0.03	0.92	-0.71	-0.33	-0.17	-0.24	-0.36	0.05
01581700	AMOI	90.13	188.28	0.68	0.32	0.56	0.11	-0.24	0.87	-0.35	0.10	0.23	-0.06	0.23	-0.22
01581700	AOI	90.13	188.28	0.68	0.32	0.56	0.11	-0.24	0.87	0.11	0.28	-0.26	0.26	-0.35	-0.15
01581700	ONI	90.13	188.28	0.68	0.32	0.56	0.11	-0.24	0.87	-0.68	-0.40	-0.16	-0.29	-0.22	0.06
01582000	AMOI	137.01	302.32	0.45	0.22	0.12	0.39	0.03	0.87	-0.20	0.04	-0.26	0.09	0.17	0.52
01582000	AOI	137.01	302.32	0.45	0.22	0.12	0.39	0.03	0.87	0.27	0.42	0.61	-0.06	0.06	-0.29
01582000	ONI	137.01	302.32	0.45	0.22	0.12	0.39	0.03	0.87	-0.70	-0.41	-0.04	-0.34	-0.27	-0.06
01583500	AMOI	154.88	262.08	0.27	0.17	0.31	0.01	-0.36	0.91	0.08	-0.02	-0.34	-0.09	-0.10	0.23
01583500	AOI	154.88	262.08	0.27	0.17	0.31	0.01	-0.36	0.91	0.33	0.06	-0.06	-0.05	-0.10	-0.09
01583500	ONI	154.88	262.08	0.27	0.17	0.31	0.01	-0.36	0.91	-0.58	-0.18	-0.10	-0.28	0.04	0.38
01593500	AMOI	98.42	258.56	0.08	0.47	-0.10	0.26	-0.53	0.91	-0.03	0.42	-0.01	0.35	-0.05	0.13
01593500	AOI	98.42	258.56	0.08	0.47	-0.10	0.26	-0.53	0.91	0.38	-0.02	-0.01	0.02	-0.01	-0.25
01593500	ONI	98.42	258.56	0.08	0.47	-0.10	0.26	-0.53	0.91	-0.50	-0.10	-0.10	-0.17	0.11	0.45
01643000	AMOI	2116.02	231.21	0.31	0.39	-0.02	0.32	-0.24	0.96	-0.13	-0.54	0.10	-0.61	0.32	-0.05
01643000	AOI	2116.02	231.21	0.31	0.39	-0.02	0.32	-0.24	0.96	0.46	0.09	0.31	-0.01	0.30	-0.27
01643000	ONI	2116.02	231.21	0.31	0.39	-0.02	0.32	-0.24	0.96	-0.44	0.09	0.06	-0.01	0.30	0.09
01645000	AMOI	261.59	213.31	0.21	-0.14	0.22	0.03	0.36	0.97	0.11	-0.12	0.26	-0.29	0.71	0.21
01645000	AOI	261.59	213.31	0.21	-0.14	0.22	0.03	0.36	0.97	0.49	0.24	-0.16	0.14	-0.23	0.09
01645000	ONI	261.59	213.31	0.21	-0.14	0.22	0.03	0.36	0.97	-0.59	-0.44	-0.00	-0.23	-0.35	-0.43
01666500	AMOI	463.61	283.70	-0.01	-0.12	-0.05	-0.06	0.12	0.81	-0.32	0.15	0.05	0.20	-0.06	-0.40
01666500	AOI	463.61	283.70	-0.01	-0.12	-0.05	-0.06	0.12	0.81	0.47	0.27	-0.13	0.12	-0.24	-0.05

Site	Index	DA(km2)	Elev(m)	r.CC.Q50	r.A.Q50	r.V.Q50	r.W.Q50	r.B.Q50	r.Q50.P	r.Q50.Ind	r.CC.Ind	r.A.Ind	r.V.Ind	r.W.Ind	r.B.Ind
01666500	ONI	463.61	283.70	-0.01	-0.12	-0.05	-0.06	0.12	0.81	-0.43	0.09	-0.21	0.08	0.09	0.09
01667500	AMOI	1212.12	241.36	0.25	-0.03	-0.13	0.03	-0.25	0.86	-0.28	-0.27	-0.24	-0.18	0.75	0.61
01667500	AOI	1212.12	241.36	0.25	-0.03	-0.13	0.03	-0.25	0.86	0.48	0.24	0.26	-0.13	0.25	-0.11
01667500	ONI	1212.12	241.36	0.25	-0.03	-0.13	0.03	-0.25	0.86	-0.51	0.14	0.42	0.11	0.12	-0.09
02021500	AMOI	852.11	1099.78	0.19	-0.20	0.37	0.25	0.07	0.74	-0.08	0.57	0.20	0.25	-0.26	-0.27
02021500	AOI	852.11	1099.78	0.19	-0.20	0.37	0.25	0.07	0.74	0.46	-0.16	-0.05	0.02	-0.11	-0.15
02021500	ONI	852.11	1099.78	0.19	-0.20	0.37	0.25	0.07	0.74	-0.29	-0.08	-0.07	0.12	-0.13	0.09
02029000	AMOI	11864.74	252.47	0.17	0.42	-0.38	0.27	-0.24	0.83	-0.30	0.04	-0.71	0.32	-0.02	0.55
02029000	AOI	11864.74	252.47	0.17	0.42	-0.38	0.27	-0.24	0.83	0.55	-0.18	0.22	-0.42	0.18	-0.09
02029000	ONI	11864.74	252.47	0.17	0.42	-0.38	0.27	-0.24	0.83	-0.17	0.06	-0.42	0.35	-0.85	0.53
02034000	AMOI	1717.16	210.39	0.21	0.05	0.06	-0.26	-0.60	0.78	-0.42	-0.06	-0.37	0.09	-0.27	0.36
02034000	AOI	1717.16	210.39	0.21	0.05	0.06	-0.26	-0.60	0.78	0.30	-0.00	0.16	-0.20	-0.12	0.00
02034000	ONI	1717.16	210.39	0.21	0.05	0.06	-0.26	-0.60	0.78	-0.55	-0.41	0.40	-0.45	0.36	0.17
02039500	AMOI	782.18	280.94	0.54	0.05	0.55	0.63	0.20	0.34	-0.63	-0.82	0.20	-0.83	-0.32	-0.33
02039500	AOI	782.18	280.94	0.54	0.05	0.55	0.63	0.20	0.34	0.18	0.19	0.21	0.18	0.08	-0.26
02039500	ONI	782.18	280.94	0.54	0.05	0.55	0.63	0.20	0.34	-0.34	-0.02	0.11	0.10	-0.20	-0.15
02073000	AMOI	981.61	657.22	0.30	0.33	0.17	0.34	0.01	0.80	-0.40	-0.57	-0.28	-0.52	0.12	0.37
02073000	AOI	981.61	657.22	0.30	0.33	0.17	0.34	0.01	0.80	0.45	0.07	-0.10	0.01	0.17	0.21
02073000	ONI	981.61	657.22	0.30	0.33	0.17	0.34	0.01	0.80	-0.32	-0.24	-0.29	-0.20	-0.16	0.50
02074000	AMOI	1393.41	539.56	0.01	0.04	0.00	-0.40	-0.17	0.76	-0.29	-0.76	-0.60	-0.45	0.36	0.69
02074000	AOI	1393.41	539.56	0.01	0.04	0.00	-0.40	-0.17	0.76	0.51	0.20	0.17	0.20	-0.13	-0.32
02074000	ONI	1393.41	539.56	0.01	0.04	0.00	-0.40	-0.17	0.76	-0.38	0.09	0.18	-0.28	0.16	-0.18
02083500	AMOI	5653.95	9.32	-0.26	0.13	-0.65	0.29	0.01	0.52	-0.18	0.19	0.05	-0.06	-0.05	-0.28
02083500	AOI	5653.95	9.32	-0.26	0.13	-0.65	0.29	0.01	0.52	-0.17	-0.21	-0.23	0.21	-0.35	0.42
02083500	ONI	5653.95	9.32	-0.26	0.13	-0.65	0.29	0.01	0.52	-0.14	-0.38	-0.16	-0.23	-0.33	0.21
02118500	AMOI	401.45	734.78	0.55	0.43	0.31	0.72	-0.47	0.74	-0.67	-0.68	-0.57	-0.14	-0.36	0.70
02118500	AOI	401.45	734.78	0.55	0.43	0.31	0.72	-0.47	0.74	-0.02	-0.01	-0.16	0.52	-0.20	-0.04
02118500	ONI	401.45	734.78	0.55	0.43	0.31	0.72	-0.47	0.74	0.11	0.04	0.15	-0.04	-0.05	-0.13
02131000	AMOI	22869.61	23.54	0.02	0.35	-0.18	0.33	-0.12	0.52	-0.86	-0.09	-0.65	0.45	-0.54	0.33
02131000	AOI	22869.61	23.54	0.02	0.35	-0.18	0.33	-0.12	0.52	-0.10	0.44	0.30	0.19	0.46	0.15
02131000	ONI	22869.61	23.54	0.02	0.35	-0.18	0.33	-0.12	0.52	-0.05	0.65	0.14	0.45	0.08	0.15
02144000	AMOI	82.36	705.86	0.52	0.64	-0.51	0.39	-0.51	0.89	-0.84	-0.64	-0.60	0.46	-0.27	0.56
02144000	AOI	82.36	705.86	0.52	0.64	-0.51	0.39	-0.51	0.89	-0.21	0.08	-0.10	0.10	-0.24	0.07
02144000	ONI	82.36	705.86	0.52	0.64	-0.51	0.39	-0.51	0.89	-0.15	-0.08	-0.03	0.04	0.11	0.03
02177000	AMOI	536.13	1165.50	-0.04	0.50	-0.29	-0.01	-0.20	0.92	-0.68	-0.05	-0.08	-0.07	-0.09	-0.13
02177000	AOI	536.13	1165.50	-0.04	0.50	-0.29	-0.01	-0.20	0.92	-0.29	-0.37	-0.37	-0.20	-0.12	0.27
02177000	ONI	536.13	1165.50	-0.04	0.50	-0.29	-0.01	-0.20	0.92	0.10	0.37	0.30	-0.06	-0.13	-0.12
02192000	AMOI	3677.79	356.80	0.71	0.32	0.61	-0.27	-0.62	0.85	-0.66	-0.49	0.29	-0.72	0.21	0.14
02192000	AOI	3677.79	356.80	0.71	0.32	0.61	-0.27	-0.62	0.85	-0.09	0.05	-0.04	-0.00	-0.32	0.14
02192000	ONI	3677.79	356.80	0.71	0.32	0.61	-0.27	-0.62	0.85	0.19	0.20	0.42	0.12	0.03	-0.27
02225500	AMOI	2874.89	73.10	-0.06	-0.17	0.22	-0.27	-0.04	0.83	-0.55	-0.22	-0.27	-0.31	0.56	0.64
02225500	AOI	2874.89	73.10	-0.06	-0.17	0.22	-0.27	-0.04	0.83	0.22	-0.05	0.06	-0.10	0.16	-0.08
02225500	ONI	2874.89	73.10	-0.06	-0.17	0.22	-0.27	-0.04	0.83	0.09	0.06	0.12	0.07	-0.09	-0.28
02472000	AMOI	1924.36	197.01	0.24	0.17	0.00	0.15	-0.18	0.77	-0.61	-0.61	-0.38	-0.14	-0.26	0.29
02472000	AOI	1924.36	197.01	0.24	0.17	0.00	0.15	-0.18	0.77	0.49	0.22	0.20	-0.09	0.35	-0.05
02472000	ONI	1924.36	197.01	0.24	0.17	0.00	0.15	-0.18	0.77	0.24	-0.10	-0.13	0.10	-0.16	-0.16
02475000	AMOI	9052.02	42.15	0.41	0.42	0.11	0.24	-0.35	0.82	-0.54	-0.60	-0.54	-0.36	-0.75	0.35
02475000	AOI	9052.02	42.15	0.41	0.42	0.11	0.24	-0.35	0.82	0.59	0.07	0.14	-0.14	0.06	0.08
02475000	ONI	9052.02	42.15	0.41	0.42	0.11	0.24	-0.35	0.82	0.11	0.08	0.01	0.04	0.10	0.34
02479000	AMOI	17068.03	26.25	0.11	0.35	0.12	0.09	-0.15	0.94	-0.84	-0.45	-0.47	-0.31	0.30	0.53
02479000	AOI	17068.03	26.25	0.11	0.35	0.12	0.09	-0.15	0.94	0.73	0.20	0.44	0.04	0.31	-0.13
02479000	ONI	17068.03	26.25	0.11	0.35	0.12	0.09	-0.15	0.94	-0.09	-0.13	0.08	-0.58	-0.20	-0.27
03080000	AMOI	313.39	1335.26	-0.10	0.28	-0.46	0.43	-0.15	0.70	-0.10	-0.43	-0.85	0.78	-0.71	0.83
03080000	AOI	313.39	1335.26	-0.10	0.28	-0.46	0.43	-0.15	0.70	0.56	-0.00	0.07	-0.09	0.18	-0.14
03080000	ONI	313.39	1335.26	-0.10	0.28	-0.46	0.43	-0.15	0.70	-0.09	-0.42	-0.08	0.09	-0.13	0.14
03102850	AMOI	872.83	913.94	0.31	0.25	0.17	0.48	-0.15	0.85	0.56	0.55	0.55	0.46	0.35	-0.62
03102850	AOI	872.83	913.94	0.31	0.25	0.17	0.48	-0.15	0.85	0.05	-0.18	0.01	-0.08	0.12	0.17
03102850	ONI	872.83	913.94	0.31	0.25	0.17	0.48	-0.15	0.85	-0.32	-0.04	-0.01	0.11	-0.15	0.14

Site	Index	DA(km2)	Elev(m)	r.CC.Q50	r.A.Q50	r.V.Q50	r.W.Q50	r.B.Q50	r.Q50.P	r.Q50.Ind	r.CC.Ind	r.A.Ind	r.V.Ind	r.W.Ind	r.B.Ind
03170000	AMOI	800.31	1815.62	0.54	-0.51	0.63	0.41	0.55	0.81	-0.35	-0.53	0.38	-0.73	-0.48	-0.68
03170000	AOI	800.31	1815.62	0.54	-0.51	0.63	0.41	0.55	0.81	0.09	0.33	-0.16	0.43	0.39	0.25
03170000	ONI	800.31	1815.62	0.54	-0.51	0.63	0.41	0.55	0.81	-0.39	-0.02	0.11	0.07	0.03	0.10
03285000	AMOI	823.62	750.10	-0.14	-0.46	0.22	-0.05	0.29	0.77	-0.04	0.41	0.04	-0.02	0.11	-0.27
03285000	AOI	823.62	750.10	-0.14	-0.46	0.22	-0.05	0.29	0.77	0.70	0.06	0.10	0.39	0.04	-0.01
03285000	ONI	823.62	750.10	-0.14	-0.46	0.22	-0.05	0.29	0.77	-0.07	0.74	0.32	0.57	-0.02	-0.60
03298000	AMOI	357.42	542.60	0.16	0.08	0.36	0.21	-0.22	0.82	0.31	-0.23	-0.15	-0.30	-0.43	0.11
03298000	AOI	357.42	542.60	0.16	0.08	0.36	0.21	-0.22	0.82	0.69	-0.21	-0.09	0.24	0.29	0.11
03298000	ONI	357.42	542.60	0.16	0.08	0.36	0.21	-0.22	0.82	-0.17	-0.49	-0.62	0.10	-0.25	0.60
03298500	AMOI	3100.22	406.62	0.20	0.13	0.18	0.18	0.01	0.89	0.34	0.61	0.10	0.55	0.64	0.58
03298500	AOI	3100.22	406.62	0.20	0.13	0.18	0.18	0.01	0.89	0.63	-0.03	0.34	-0.44	0.12	-0.32
03298500	ONI	3100.22	406.62	0.20	0.13	0.18	0.18	0.01	0.89	-0.25	0.23	0.13	-0.13	-0.00	-0.07
03301500	AMOI	3364.40	400.42	-0.05	0.23	-0.16	0.30	-0.19	0.82	-0.06	0.88	0.03	0.77	0.15	-0.24
03301500	AOI	3364.40	400.42	-0.05	0.23	-0.16	0.30	-0.19	0.82	0.51	-0.07	0.26	-0.17	0.56	-0.37
03301500	ONI	3364.40	400.42	-0.05	0.23	-0.16	0.30	-0.19	0.82	-0.26	0.11	-0.11	0.25	-0.16	-0.39
03340800	AMOI	360.01	686.03	0.25	0.08	0.18	0.31	-0.04	0.85	0.13	0.39	0.26	0.64	0.52	-0.27
03340800	AOI	360.01	686.03	0.25	0.08	0.18	0.31	-0.04	0.85	0.54	-0.12	0.15	-0.08	0.03	-0.32
03340800	ONI	360.01	686.03	0.25	0.08	0.18	0.31	-0.04	0.85	-0.01	0.15	-0.04	-0.09	-0.20	0.07
03383000	AMOI	660.45	362.80	0.14	0.10	0.31	0.17	-0.19	0.84	0.20	0.35	0.31	0.44	0.55	-0.34
03383000	AOI	660.45	362.80	0.14	0.10	0.31	0.17	-0.19	0.84	0.46	-0.17	0.03	-0.07	-0.29	0.04
03383000	ONI	660.45	362.80	0.14	0.10	0.31	0.17	-0.19	0.84	-0.17	0.22	0.06	-0.32	0.03	0.03
03453500	AMOI	3449.87	1646.79	0.27	0.64	0.02	-0.17	-0.64	0.75	-0.60	-0.47	-0.60	-0.35	0.25	0.74
03453500	AOI	3449.87	1646.79	0.27	0.64	0.02	-0.17	-0.64	0.75	-0.12	0.10	0.03	0.07	-0.27	-0.13
03453500	ONI	3449.87	1646.79	0.27	0.64	0.02	-0.17	-0.64	0.75	0.04	-0.13	0.17	-0.01	-0.34	-0.07
03473000	AMOI	784.77	1792.30	0.19	0.16	0.02	0.30	-0.18	0.81	-0.23	-0.26	-0.39	-0.05	-0.29	0.35
03473000	AOI	784.77	1792.30	0.19	0.16	0.02	0.30	-0.18	0.81	0.53	0.03	-0.03	-0.00	-0.06	0.02
03473000	ONI	784.77	1792.30	0.19	0.16	0.02	0.30	-0.18	0.81	-0.08	0.08	-0.26	0.04	-0.16	0.30
03500240	AMOI	147.89	2017.18	0.69	0.08	0.01	0.15	-0.22	0.92	-0.70	-0.80	0.07	-0.25	0.12	0.03
03500240	AOI	147.89	2017.18	0.69	0.08	0.01	0.15	-0.22	0.92	-0.18	0.21	-0.08	0.23	0.10	0.07
03500240	ONI	147.89	2017.18	0.69	0.08	0.01	0.15	-0.22	0.92	-0.08	-0.17	0.03	-0.23	0.27	-0.01
04112500	AMOI	919.45	823.96	-0.05	0.29	-0.20	0.25	-0.32	0.82	0.40	-0.71	0.60	-0.67	0.38	-0.40
04112500	AOI	919.45	823.96	-0.05	0.29	-0.20	0.25	-0.32	0.82	0.80	0.10	0.04	0.02	0.12	-0.12
04112500	ONI	919.45	823.96	-0.05	0.29	-0.20	0.25	-0.32	0.82	0.13	-0.14	0.10	-0.02	0.04	-0.01
04154000	AMOI	1077.44	710.38	0.16	0.13	0.28	0.15	-0.16	0.74	0.29	-0.12	-0.15	-0.12	0.04	0.09
04154000	AOI	1077.44	710.38	0.16	0.13	0.28	0.15	-0.16	0.74	0.71	-0.24	-0.09	-0.19	-0.02	0.07
04154000	ONI	1077.44	710.38	0.16	0.13	0.28	0.15	-0.16	0.74	0.28	0.02	0.07	-0.05	0.43	0.10
04174500	AMOI	1888.10	744.81	0.08	0.02	0.09	-0.12	-0.23	0.68	0.36	-0.52	-0.26	-0.45	0.40	0.21
04174500	AOI	1888.10	744.81	0.08	0.02	0.09	-0.12	-0.23	0.68	0.62	0.12	0.01	0.10	-0.18	-0.15
04174500	ONI	1888.10	744.81	0.08	0.02	0.09	-0.12	-0.23	0.68	-0.07	0.04	0.03	0.15	0.05	-0.03
04215500	AMOI	349.65	604.86	0.09	-0.15	0.06	0.35	0.12	0.79	0.19	-0.44	-0.27	-0.32	-0.17	-0.05
04215500	AOI	349.65	604.86	0.09	-0.15	0.06	0.35	0.12	0.79	0.70	0.20	0.00	0.09	0.26	0.13
04215500	ONI	349.65	604.86	0.09	-0.15	0.06	0.35	0.12	0.79	-0.13	-0.03	0.52	-0.13	0.08	-0.42
04239000	AMOI	229.21	414.19	-0.13	0.30	-0.58	0.54	-0.45	0.69	-0.62	0.09	-0.36	0.68	-0.75	0.42
04239000	AOI	229.21	414.19	-0.13	0.30	-0.58	0.54	-0.45	0.69	0.60	-0.36	-0.14	0.08	0.25	0.04
04239000	ONI	229.21	414.19	-0.13	0.30	-0.58	0.54	-0.45	0.69	-0.14	-0.21	-0.32	0.05	-0.15	0.40
05280000	AMOI	6837.57	893.08	0.18	0.45	-0.10	0.23	-0.33	0.66	-0.09	0.76	0.20	0.68	-0.13	-0.30
05280000	AOI	6837.57	893.08	0.18	0.45	-0.10	0.23	-0.33	0.66	0.54	-0.05	0.02	0.11	-0.35	-0.05
05280000	ONI	6837.57	893.08	0.18	0.45	-0.10	0.23	-0.33	0.66	-0.12	-0.17	-0.41	0.02	-0.46	-0.08
05291000	AMOI	1030.82	996.96	0.58	0.17	0.20	0.23	-0.45	0.67	0.48	0.45	0.36	-0.48	0.52	-0.55
05291000	AOI	1030.82	996.96	0.58	0.17	0.20	0.23	-0.45	0.67	0.62	-0.02	-0.25	0.13	-0.44	-0.05
05291000	ONI	1030.82	996.96	0.58	0.17	0.20	0.23	-0.45	0.67	-0.26	-0.02	0.27	-0.17	0.22	-0.03
05293000	AMOI	1188.81	953.34	-0.07	-0.23	0.41	-0.12	-0.31	0.54	0.45	0.34	0.43	0.46	0.27	-0.65
05293000	AOI	1188.81	953.34	-0.07	-0.23	0.41	-0.12	-0.31	0.54	0.60	-0.42	-0.38	0.20	-0.43	-0.06
05293000	ONI	1188.81	953.34	-0.07	-0.23	0.41	-0.12	-0.31	0.54	-0.26	0.10	0.21	-0.05	0.01	0.16
05300000	AMOI	2486.39	946.98	-0.15	0.06	-0.27	0.53	0.13	0.60	0.41	-0.10	0.14	-0.59	0.34	-0.18
05300000	AOI	2486.39	946.98	-0.15	0.06	-0.27	0.53	0.13	0.60	0.67	-0.33	-0.28	-0.16	0.25	-0.02
05300000	ONI	2486.39	946.98	-0.15	0.06	-0.27	0.53	0.13	0.60	-0.07	0.40	0.11	0.23	0.11	-0.15
05421000	AMOI	2714.31	882.85	0.03	0.07	0.10	-0.13	-0.42	0.96	0.16	0.15	0.22	0.32	-0.08	-0.30

Site	Index	DA(km2)	Elev(m)	r.CC.Q50	r.A.Q50	r.V.Q50	r.W.Q50	r.B.Q50	r.Q50.P	r.Q50.Ind	r.CC.Ind	r.A.Ind	r.V.Ind	r.W.Ind	r.B.Ind
05421000	AOI	2714.31	882.85	0.03	0.07	0.10	-0.13	-0.42	0.96	0.44	0.19	-0.29	0.56	-0.29	0.09
05421000	ONI	2714.31	882.85	0.03	0.07	0.10	-0.13	-0.42	0.96	0.20	0.37	-0.28	0.46	-0.42	0.24
05452200	AMOI	183.63	786.59	0.20	0.05	0.24	-0.05	-0.11	0.83	0.09	0.44	0.56	0.11	-0.42	-0.60
05452200	AOI	183.63	786.59	0.20	0.05	0.24	-0.05	-0.11	0.83	0.73	0.29	0.24	0.11	0.09	-0.20
05452200	ONI	183.63	786.59	0.20	0.05	0.24	-0.05	-0.11	0.83	-0.16	0.19	0.09	0.31	-0.11	-0.19
05453000	AMOI	489.51	744.94	0.07	0.20	0.09	0.18	-0.12	0.92	0.15	-0.05	0.18	-0.49	0.21	0.21
05453000	AOI	489.51	744.94	0.07	0.20	0.09	0.18	-0.12	0.92	0.55	0.08	0.01	0.12	0.01	0.13
05453000	ONI	489.51	744.94	0.07	0.20	0.09	0.18	-0.12	0.92	-0.15	0.30	0.37	0.07	0.43	0.28
05454300	AMOI	254.08	647.48	0.33	0.24	0.12	0.10	-0.27	0.86	0.06	0.07	-0.08	-0.06	-0.26	-0.27
05454300	AOI	254.08	647.48	0.33	0.24	0.12	0.10	-0.27	0.86	0.57	0.47	0.45	0.44	0.34	-0.42
05454300	ONI	254.08	647.48	0.33	0.24	0.12	0.10	-0.27	0.86	-0.10	0.15	0.20	-0.02	0.24	0.05
05463500	AMOI	784.77	865.03	0.38	0.52	0.29	0.42	-0.31	0.73	0.19	-0.29	-0.06	-0.03	0.32	0.40
05463500	AOI	784.77	865.03	0.38	0.52	0.29	0.42	-0.31	0.73	0.58	0.01	0.08	-0.19	-0.08	-0.17
05463500	ONI	784.77	865.03	0.38	0.52	0.29	0.42	-0.31	0.73	-0.10	0.13	0.17	-0.08	-0.05	-0.09
05474000	AMOI	11168.04	521.24	0.20	-0.32	0.36	-0.07	0.52	0.86	-0.26	-0.42	-0.23	-0.12	-0.52	0.03
05474000	AOI	11168.04	521.24	0.20	-0.32	0.36	-0.07	0.52	0.86	0.54	0.04	0.16	-0.08	0.09	0.07
05474000	ONI	11168.04	521.24	0.20	-0.32	0.36	-0.07	0.52	0.86	-0.40	-0.25	0.39	-0.54	0.02	-0.63
05481000	AMOI	2185.95	989.57	-0.11	-0.21	0.15	0.02	0.50	0.80	0.05	0.21	0.28	-0.08	0.39	-0.05
05481000	AOI	2185.95	989.57	-0.11	-0.21	0.15	0.02	0.50	0.80	0.67	-0.19	-0.26	0.11	-0.13	0.32
05481000	ONI	2185.95	989.57	-0.11	-0.21	0.15	0.02	0.50	0.80	0.08	0.27	-0.12	0.64	-0.18	0.23
05481300	AMOI	14120.63	894.00	0.21	0.52	-0.25	0.40	-0.31	0.65	0.31	0.50	0.11	0.61	0.22	0.52
05481300	AOI	14120.63	894.00	0.21	0.52	-0.25	0.40	-0.31	0.65	0.49	0.08	0.00	-0.22	-0.15	0.00
05481300	ONI	14120.63	894.00	0.21	0.52	-0.25	0.40	-0.31	0.65	0.19	0.23	0.17	-0.11	0.53	0.11
05481950	AMOI	927.22	806.98	0.03	0.06	0.47	-0.15	-0.26	0.92	0.19	-0.15	-0.09	-0.17	-0.29	0.14
05481950	AOI	927.22	806.98	0.03	0.06	0.47	-0.15	-0.26	0.92	0.57	-0.27	-0.30	0.47	-0.24	0.11
05481950	ONI	927.22	806.98	0.03	0.06	0.47	-0.15	-0.26	0.92	-0.10	-0.54	-0.64	-0.04	-0.50	-0.08
05512500	AMOI	102.05	638.48	-0.20	-0.17	0.31	0.10	0.22	0.75	0.03	-0.92	-0.33	-0.80	-0.60	0.24
05512500	AOI	102.05	638.48	-0.20	-0.17	0.31	0.10	0.22	0.75	0.54	-0.19	0.50	0.06	0.36	-0.34
05512500	ONI	102.05	638.48	-0.20	-0.17	0.31	0.10	0.22	0.75	-0.14	0.33	0.48	0.36	0.20	-0.69
05583000	AMOI	13190.82	452.88	0.21	0.07	0.22	-0.38	-0.36	0.84	0.02	-0.21	-0.19	-0.04	-0.44	-0.08
05583000	AOI	13190.82	452.88	0.21	0.07	0.22	-0.38	-0.36	0.84	0.64	0.52	0.18	0.22	-0.01	-0.39
05583000	ONI	13190.82	452.88	0.21	0.07	0.22	-0.38	-0.36	0.84	0.36	0.30	0.36	0.18	0.03	-0.13
06904500	AMOI	3548.29	737.65	0.49	0.48	0.33	0.25	-0.54	0.64	-0.29	-0.79	-0.74	-0.87	-0.88	0.71
06904500	AOI	3548.29	737.65	0.49	0.48	0.33	0.25	-0.54	0.64	0.28	0.03	-0.07	0.01	-0.01	0.11
06904500	ONI	3548.29	737.65	0.49	0.48	0.33	0.25	-0.54	0.64	-0.28	0.05	0.06	-0.13	0.14	0.06
06905500	AMOI	4843.28	632.66	0.27	0.38	0.13	0.06	-0.55	0.76	-0.26	-0.15	-0.56	-0.31	-0.26	0.49
06905500	AOI	4843.28	632.66	0.27	0.38	0.13	0.06	-0.55	0.76	0.38	0.37	0.13	0.40	0.16	-0.02
06905500	ONI	4843.28	632.66	0.27	0.38	0.13	0.06	-0.55	0.76	-0.20	-0.04	0.10	-0.15	-0.02	0.13
07060500	AMOI	25848.10	316.38	0.06	0.19	0.32	-0.33	-0.35	0.67	-0.13	0.64	0.20	0.45	-0.23	-0.33
07060500	AOI	25848.10	316.38	0.06	0.19	0.32	-0.33	-0.35	0.67	0.67	0.19	-0.03	0.43	-0.29	-0.14
07060500	ONI	25848.10	316.38	0.06	0.19	0.32	-0.33	-0.35	0.67	-0.09	-0.22	-0.07	-0.08	-0.10	-0.28
07196900	AMOI	105.15	986.47	0.15	0.15	-0.07	0.05	-0.06	0.95	-0.15	-0.07	-0.11	-0.25	0.12	0.24
07196900	AOI	105.15	986.47	0.15	0.15	-0.07	0.05	-0.06	0.95	0.71	0.21	0.18	-0.03	0.23	0.08
07196900	ONI	105.15	986.47	0.15	0.15	-0.07	0.05	-0.06	0.95	-0.36	0.18	0.17	0.52	0.15	-0.42
08200000	AMOI	247.60	1169.10	0.67	0.71	0.60	0.69	-0.55	0.83	-0.68	-0.80	-0.82	-0.37	-0.62	0.54
08200000	AOI	247.60	1169.10	0.67	0.71	0.60	0.69	-0.55	0.83	0.32	-0.09	0.14	0.03	0.25	-0.13
08200000	ONI	247.60	1169.10	0.67	0.71	0.60	0.69	-0.55	0.83	0.13	-0.14	-0.15	0.07	-0.17	-0.02
